# Supplementary material for: Genome Wild Analysis and Molecular Understanding of the Aquaporin Diversity in Olive Trees (Olea Europaea L.)
Source: Int J Mol Sci. 2020 Jun 11;21(11):4183. doi: 10.3390/ijms21114183 (PMC7312470; doi:10.3390/ijms21114183)
Supplement: Supplementary file 1 [file ijms-21-04183-s001.zip › ijms-821530_Supplementary_files/Figure S1_Faize_OeuMIP_sequences.pdf]

Figure S1 Detailed *Oeu*MIP sequences (genomic, CDS and protein sequences) from the wild olive species *Olea europaea* var. *sylvestris*.

All sequences are presented in their original version, and if need be, in their corrected versions. Corrections are explained in the "***Observation***" item). Only the "corrected" sequences were used for analysis.
For the genomic sequences, the introns are in blue.
Codons "Stop" and "Start" are in red.
All sequences are classified according to their subfamily.

PIP1 – Plasmalemic Intrinsic Protein type 1

*Oeu*PIP1;1a Oeu038800.1

Observation: OeuPIP1;1a, 100% homolog to OePIP1.1 DQ202708 previously cloned by Secchi et al., 2007, and to PIP1.1 [A0A385A1M4 / A0A385A1N7] from UniProtKB
Corrected as follow : Exon 1 upstream extended with 63n

>*Oeu*PIP1;1a_Oeu038800.1 | chr19:7203994..7205679 forward Original version
ATGGAGAGCAAAGAGGAGGATGTACGAGTGGGAGCCAACAAATTCGCCGAGAGGCAGCCGATTGGCACGGCTGCT
CAAAGCCAGGACAAGGACTACAAGGAACCACCACCGGCGCCACTTTTCGAGCCGGGTGAGCTGTCCTCGTGGTCG
TTCTACAGAGCTGGCATAGCAGAGTTCATAGCCACTTTCTTGTTCCTTTATATTACAGTTTTGACAGTGATGGGA
GTTAGTAAGTCTGATTCCAAGTGCAAAACTGTTGGAATCCAAGGTATTGCTTGGGCATTTGGTGGCATGATCTTT
GCCCTTGTTTACTGCACTGCAGGAATTTCAGGCAAGTTCTTTTCTATATATATAATTTGCAAATTTTGCTTGTAT
TTGCTATGATCTTCAACAGTAACGATCCGTCTTAAAGCTCAAAGAGGGAGTCGTCAGATTTGAAGATTGGTTTGT
GGGGTAGATATGAAAATACTAACCAACATATATATTGGATTATGGTCTCTGTCTCTGCAGTTTAGTCTTTGATAG
TATATGGAATTTAGGTTTTGCCATTAAAATTAGAAATTACCTAATTCAAAATTGAGTTGATTTTTCATGAATTTC
AGTTATAAGGTTTTAATTCCATTTGATATTAACATGGCAATTCAGTAAACCAAATCGTTTCAGCAATCAAATTTA
TGATGAACAATTTTAACAGTACTACTAACCTATTTTCTTCAAAGAAAAGGGCAAAATTTTAATGAACTGCTTGTT
TTGCAGGGGGGCATATAAATCCGGCAGTGACTTTCGGGCTGTTTTTGGGGAGGAAATTGTCGTTGACCCGGGCTG
TGTTCTACATGGTGATGCAGTGTCTTGGAGCCATATGTGGTGCTGGTGTGGTTAAGGGATTCGGAAAGACCCTGT
ACATGACCAAGGGTGGTGGTGCTAATGTAGTAGCCCACGGTTACACCAAGGGCGATGGCCTAGGTGCTGAAATAA
TCGGAACTTTTGTGCTCGTTTACACTGTCTTCTCTGCTACTGATGCCAAACGTAGCGCCAGAGACTCGCACGTTC
CTGTAAGCACTCCTCTTGTCTTTGTAGATTGAGATTGAACAAGAATTTGCTTGTGTATCTGTAATCAAATTGTCT
TTTCGTTTTGTAAGAACTGATGAATCAAAAAATGTCATTGTTGATCAAGAAAAAGTTTGATTAATTGGATTCTCA
TATGCTGTAATTCAATGGTTTTCAGATATTGGCCCCCTTGCCTATTGGGTTTGCTGTGTTCTTGGTGCACTTGGC
AACCATCCCTATTACCGGTACAGGTATTAACCCCGCCAGGAGTCTTGGTGCAGCAATCATCTACAACAAAGACAA
GGCATGGGATGATCACGTAAGCTAAAAATTCAACTGCCTGAATTCAACAATTGAAATATTTGAGTTAAGGGGGGC
ATACCTAGACAATTATATCCTAACAAATTTTTACGTTGTTCACTTATCACAATCCTCCTTTTATCTATTTTGGGA
CTACGACGAAATTTTTTAATAATCTGGCAGATGAGATGATAAGATTGTCTAAAGTTTGTTCTCACTCTTATCTTG
CTCTGATATGATAATATGCAGTGGATATTCTGGGTTGGACCGTTCATTGGGGCAGCACTTGCAGCTCTATACCAT
GTGGTGGTGATCCGAGCCATCCCATTCAAAAAATAA

>*Oeu*PIP1;1a_*Oeu*038800.1 Corrected version
ATGGAGAGCAAAGAGGAGGATGTACGAGTGGGAGCCAACAAATTCGCCGAGAGGCAGCCGATTGGCACGGCTGCT
CAAAGCCAGGACAAGGACTACAAGGAACCACCACCGGCGCCACTTTTCGAGCCGGGTGAGCTGTCCTCGTGGTCG
TTCTACAGAGCTGGCATAGCAGAGTTCATAGCCACTTTCTTGTTCCTTTATATTACAGTTTTGACAGTGATGGGA
GTTAGTAAGTCTGATTCCAAGTGCAAAACTGTTGGAATCCAAGGTATTGCTTGGGCATTTGGTGGCATGATCTTT
GCCCTTGTTTACTGCACTGCAGGAATTTCAGGCAAGTTCTTTTCTATATATATAATTTGCAAATTTTGCTTGTAT
TTGCTATGATCTTCAACAGTAACGATCCGTCTTAAAGCTCAAAGAGGGAGTCGTCAGATTTGAAGATTGGTTTGT
GGGGTAGATATGAAAATACTAACCAACATATATATTGGATTATGGTCTCTGTCTCTGCAGTTTAGTCTTTGATAG
TATATGGAATTTAGGTTTTGCCATTAAAATTAGAAATTACCTAATTCAAAATTGAGTTGATTTTTCATGAATTTC
AGTTATAAGGTTTTAATTCCATTTGATATTAACATGGCAATTCAGTAAACCAAATCGTTTCAGCAATCAAATTTA
TGATGAACAATTTTAACAGTACTACTAACCTATTTTCTTCAAAGAAAAGGGCAAAATTTTAATGAACTGCTTGTT
TTGCAGGGGGGCATATAAATCCGGCAGTGACTTTCGGGCTGTTTTTGGGGAGGAAATTGTCGTTGACCCGGGCTG
TGTTCTACATGGTGATGCAGTGTCTTGGAGCCATATGTGGTGCTGGTGTGGTTAAGGGATTCGGAAAGACCCTGT
ACATGACCAAGGGTGGTGGTGCTAATGTAGTAGCCCACGGTTACACCAAGGGCGATGGCCTAGGTGCTGAAATAA

TCGGAACTTTTGTGCTCGTTTACACTGTCTTCTCTGCTACTGATGCCAAACGTAGCGCCAGAGACTCGCACGTTC
CTGTAAGCACTCCTCTTGTCTTTGTAGATTGAGATTGAACAAGAATTTGCTTGTGTATCTGTAATCAAATTGTCT
TTTCGTTTTGTAAGAACTGATGAATCAAAAAATGTCATTGTTGATCAAGAAAAAGTTTGATTAATTGGATTCTCA
TATGCTGTAATTCAATGGTTTTCAGATATTGGCCCCCTTGCCTATTGGGTTTGCTGTGTTCTTGGTGCACTTGGC
AACCATCCCTATTACCGGTACAGGTATTAACCCCGCCAGGAGTCTTGGTGCAGCAATCATCTACAACAAAGACAA
GGCATGGGATGATCACGTAAGCTAAAAATTCAACTGCCTGAATTCAACAATTGAAATATTTGAGTTAAGGGGGGC
ATACCTAGACAATTATATCCTAACAAATTTTTACGTTGTTCACTTATCACAATCCTCCTTTTATCTATTTTGGGA
CTACGACGAAATTTTTTAATAATCTGGCAGATGAGATGATAAGATTGTCTAAAGTTTGTTCTCACTCTTATCTTG
CTCTGATATGATAATATGCAGTGGATATTCTGGGTTGGACCGTTCATTGGGGCAGCACTTGCAGCTCTATACCAT
GTGGTGGTGATCCGAGCCATCCCATTCAAAAAATAA

>*Oeu*PIP1;1a_*Oeu*038800.1

ATGGAGAGCAAAGAGGAGGATGTACGAGTGGGAGCCAACAAATTCGCCGAGAGGCAGCCGATTGGCACGGCTGCT
CAAAGCCAGGACAAGGACTACAAGGAACCACCACCGGCGCCACTTTTCGAGCCGGGTGAGCTGTCCTCGTGGTCG
TTCTACAGAGCTGGCATAGCAGAGTTCATAGCCACTTTCTTGTTCCTTTATATTACAGTTTTGACAGTGATGGGA
GTTAGTAAGTCTGATTCCAAGTGCAAAACTGTTGGAATCCAAGGTATTGCTTGGGCATTTGGTGGCATGATCTTT
GCCCTTGTTTACTGCACTGCAGGAATTTCAGGGGGGCATATAAATCCGGCAGTGACTTTCGGGCTGTTTTTGGGG
AGGAAATTGTCGTTGACCCGGGCTGTGTTCTACATGGTGATGCAGTGTCTTGGAGCCATATGTGGTGCTGGTGTG
GTTAAGGGATTCGGAAAGACCCTGTACATGACCAAGGGTGGTGGTGCTAATGTAGTAGCCCACGGTTACACCAAG
GGCGATGGCCTAGGTGCTGAAATAATCGGAACTTTTGTGCTCGTTTACACTGTCTTCTCTGCTACTGATGCCAAA
CGTAGCGCCAGAGACTCGCACGTTCCTATATTGGCCCCCTTGCCTATTGGGTTTGCTGTGTTCTTGGTGCACTTG
GCAACCATCCCTATTACCGGTACAGGTATTAACCCCGCCAGGAGTCTTGGTGCAGCAATCATCTACAACAAAGAC
AAGGCATGGGATGATCACTGGATATTCTGGGTTGGACCGTTCATTGGGGCAGCACTTGCAGCTCTATACCATGTG
GTGGTGATCCGAGCCATCCCATTCAAAAAATAA

>*Oeu*PIP1;1a_*Oeu*038800.1

MESKEEDVRVGANKFAERQPIGTAAQSQDKDYKEPPPAPLFEPGELSSWSFYRAGIAEFIATFLFLYITVLTVMG
VSKSDSKCKTVGIQGIAWAFGGMIFALVYCTAGISGGHINPAVTFGLFLGRKLSLTRAVFYMVMQCLGAICGAGV
VKGFGKTLYMTKGGGANVVAHGYTKGDGLGAEIIGTFVLVYTVFSATDAKRSARDSHVPILAPLPIGFAVFLVHL
ATIPITGTGINPARSLGAAIIYNKDKAWDDHWIFWVGPFIGAALAALYHVVVIRAIPFKK*

*Oeu*PIP1;1b *Oeu*038801.1

Observation: Protein 100% Homolog to *Oeu*038800.1
Corrected as follow : Exon 1 upstream extended with 63n

>*Oeu*PIP1;1b_*Oeu*038801.1 | chr19:7208573..7210258 reverse Original version

ATGGAGAGCAAAGAGGAGGATGTACGAGTGGGAGCCAACAAATTCGCCGAGAGGCAGCCGATTGGCACGGCTGCT
CAAAGCCAGGACAAGGACTACAAGGAACCACCACCGGCGCCACTTTTCGAGCCGGGTGAGCTGTCCTCGTGGTCG
TTCTACAGAGCTGGCATAGCAGAGTTCATAGCCACTTTCTTGTTCCTTTATATTACAGTTTTGACAGTGATGGGA
GTTAGTAAGTCTGATTCCAAGTGCAAAACTGTTGGAATCCAAGGTATTGCTTGGGCATTTGGTGGCATGATCTTT
GCCCTTGTTTACTGCACTGCAGGAATTTCAGGCAAGTTCTTTTCTATATATATAATTTGCAAATTTTGCTTGTAT
TTGCTATGATCTTCAACAGTAACGATCCGTCTTAAAGCTCAAAGAGGGAGTCGTCAGATTTGAAGATTGGTTTGT
GGGGTAGATATGAAAATACTAACCAACATATATATTGGATTATGGTCTCTGTCTCTGCAGTTTAGTCTTTGATAG
TATATGGAATTTAGGTTTTGCCATTAAAATTAGAAATTACCTAATTCAAAATTGAGTTGATTTTTCATGAATTTC
AGTTATAAGGTTTTAATTCCATTTGATATTAACATGGCAATTCAGTAAACCAAATCGTTTCAGCAATCAAATTTA
TGATGAACAATTTTAACAGTACTACTAACCTATTTTCTTCAAAGAAAAGGGCAAAATTTTAATGAACTGCTTGTT
TTGCAGGGGGGCATATAAATCCGGCAGTGACTTTCGGGCTGTTTTTGGGGAGGAAATTGTCGTTGACCCGGGCTG
TGTTCTACATGGTGATGCAGTGTCTTGGAGCCATATGTGGTGCTGGTGTGGTTAAGGGATTCGGAAAGACCCTGT
ACATGACCAAGGGTGGTGGTGCTAATGTAGTAGCCCACGGTTACACCAAGGGCGATGGCCTAGGTGCTGAAATAA
TCGGAACTTTTGTGCTCGTTTACACTGTCTTCTCTGCTACTGATGCCAAACGTAGCGCCAGAGACTCGCACGTTC
CTGTAAGCACTCCTCTTGTCTTTGTAGATTGAGATTGAACAAGAATTTGCTTGTGTATCTGTAATCAAATTGTCT
TTTCGTTTTGTAAGAACTGATGAATCAAAAAATGTCATTGTTGATCAAGAAAAAGTTTGATTAATTGGATTCTCA
TATGCTGTAATTCAATGGTTTTCAGATATTGGCCCCCTTGCCTATTGGGTTTGCTGTGTTCTTGGTGCACTTGGC
AACCATCCCTATTACCGGTACAGGTATTAACCCCGCCAGGAGTCTTGGTGCAGCAATCATCTACAACAAAGACAA
GGCATGGGATGATCACGTAAGCTAAAAATTCAACTGCCTGAATTCAACAATTGAAATATTTGAGTTAAGGGGGGC
ATACCTAGACAATTATATCCTAACAAATTTTTACGTTGTTCACTTATCACAATCCTCCTTTTATCTATTTTGGGA
CTACGACGAAATTTTTTAATAATCTGGCAGATGAGATGATAAGATTGTCTAAAGTTTGTTCTCACTCTTATCTTG
CTCTGATATGATAATATGCAGTGGATATTCTGGGTTGGACCGTTCATTGGGGCAGCACTTGCAGCTCTATACCAT
GTGGTGGTGATCCGAGCCATCCCATTCAAAAAATAA

>*Oeu*PIP1;1b_*Oeu*038801.1 **Corrected version**
ATGGAGAGCAAAGAGGAGGATGTACGAGTGGGAGCCAACAAATTCGCCGAGAGGCAGCCGATTGGCACGGCTGCT
CAAAGCCAGGACAAGGACTACAAGGAACCACCACCGGCGCCACTTTTCGAGCCGGGTGAGCTGTCCTCGTGGTCG
TTCTACAGAGCTGGCATAGCAGAGTTCATAGCCACTTTCTTGTTCCTTTATATTACAGTTTTGACAGTGATGGGA
GTTAGTAAGTCTGATTCCAAGTGCAAAACTGTTGGAATCCAAGGTATTGCTTGGGCATTTGGTGGCATGATCTTT
GCCCTTGTTTACTGCACTGCAGGAATTTCAGGCAAGTTCTTTTCTATATATATAATTTGCAAATTTTGCTTGTAT
TTGCTATGATCTTCAACAGTAACGATCCGTCTTAAAGCTCAAAGAGGGAGTCGTCAGATTTGAAGATTGGTTTGT
GGGGTAGATATGAAAATACTAACCAACATATATATTGGATTATGGTCTCTGTCTCTGCAGTTTAGTCTTTGATAG
TATATGGAATTTAGGTTTTGCCATTAAAATTAGAAATTACCTAATTCAAAATTGAGTTGATTTTTCATGAATTTC
AGTTATAAGGTTTTAATTCCATTTGATATTAACATGGCAATTCAGTAAACCAAATCGTTTCAGCAATCAAATTTA
TGATGAACAATTTTAACAGTACTACTAACCTATTTTCTTCAAAGAAAAGGGCAAAATTTTAATGAACTGCTTGTT
TTGCAGGGGGGCATATAAATCCGGCAGTGACTTTCGGGCTGTTTTTGGGGAGGAAATTGTCGTTGACCCGGGCTG
TGTTCTACATGGTGATGCAGTGTCTTGGAGCCATATGTGGTGCTGGTGTGGTTAAGGGATTCGGAAAGACCCTGT
ACATGACCAAGGGTGGTGGTGCTAATGTAGTAGCCCACGGTTACACCAAGGGCGATGGCCTAGGTGCTGAAATAA
TCGGAACTTTTGTGCTCGTTTACACTGTCTTCTCTGCTACTGATGCCAAACGTAGCGCCAGAGACTCGCACGTTC
CTGTAAGCACTCCTCTTGTCTTTGTAGATTGAGATTGAACAAGAATTTGCTTGTGTATCTGTAATCAAATTGTCT
TTTCGTTTTGTAAGAACTGATGAATCAAAAAATGTCATTGTTGATCAAGAAAAAGTTTGATTAATTGGATTCTCA
TATGCTGTAATTCAATGGTTTTCAGATATTGGCCCCCTTGCCTATTGGGTTTGCTGTGTTCTTGGTGCACTTGGC
AACCATCCCTATTACCGGTACAGGTATTAACCCCGCCAGGAGTCTTGGTGCAGCAATCATCTACAACAAAGACAA
GGCATGGGATGATCACGTAAGCTAAAAATTCAACTGCCTGAATTCAACAATTGAAATATTTGAGTTAAGGGGGGC
ATACCTAGACAATTATATCCTAACAAATTTTTACGTTGTTCACTTATCACAATCCTCCTTTTATCTATTTTGGGA
CTACGACGAAATTTTTTAATAATCTGGCAGATGAGATGATAAGATTGTCTAAAGTTTGTTCTCACTCTTATCTTG
CTCTGATATGATAATATGCAGTGGATATTCTGGGTTGGACCGTTCATTGGGGCAGCACTTGCAGCTCTATACCAT
GTGGTGGTGATCCGAGCCATCCCATTCAAAAAA**TAA**
>*Oeu*PIP1;1b_*Oeu*038801.1
ATGGAGAGCAAAGAGGAGGATGTACGAGTGGGAGCCAACAAATTCGCCGAGAGGCAGCCGATTGGCACGGCTGCT
CAAAGCCAGGACAAGGACTACAAGGAACCACCACCGGCGCCACTTTTCGAGCCGGGTGAGCTGTCCTCGTGGTCG
TTCTACAGAGCTGGCATAGCAGAGTTCATAGCCACTTTCTTGTTCCTTTATATTACAGTTTTGACAGTGATGGGA
GTTAGTAAGTCTGATTCCAAGTGCAAAACTGTTGGAATCCAAGGTATTGCTTGGGCATTTGGTGGCATGATCTTT
GCCCTTGTTTACTGCACTGCAGGAATTTCAGGGGGGCATATAAATCCGGCAGTGACTTTCGGGCTGTTTTTGGGG
AGGAAATTGTCGTTGACCCGGGCTGTGTTCTACATGGTGATGCAGTGTCTTGGAGCCATATGTGGTGCTGGTGTG
GTTAAGGGATTCGGAAAGACCCTGTACATGACCAAGGGTGGTGGTGCTAATGTAGTAGCCCACGGTTACACCAAG
GGCGATGGCCTAGGTGCTGAAATAATCGGAACTTTTGTGCTCGTTTACACTGTCTTCTCTGCTACTGATGCCAAA
CGTAGCGCCAGAGACTCGCACGTTCCTATATTGGCCCCCTTGCCTATTGGGTTTGCTGTGTTCTTGGTGCACTTG
GCAACCATCCCTATTACCGGTACAGGTATTAACCCCGCCAGGAGTCTTGGTGCAGCAATCATCTACAACAAAGAC
AAGGCATGGGATGATCACTGGATATTCTGGGTTGGACCGTTCATTGGGGCAGCACTTGCAGCTCTATACCATGTG
GTGGTGATCCGAGCCATCCCATTCAAAAAA**TAA**
>*Oeu*PIP1;1b_*Oeu*038801.1
MESKEEDVRVGANKFAERQPIGTAAQSQDKDYKEPPPAPLFEPGELSSWSFYRAGIAEFIATFLFLYITVLTVMG
VSKSDSKCKTVGIQGIAWAFGGMIFALVYCTAGISGGHINPAVTFGLFLGRKLSLTRAVFYMVMQCLGAICGAGV
VKGFGKTLYMTKGGGANVVAHGYTKGDGLGAEIIGTFVLVYTVFSATDAKRSARDSHVPILAPLPIGFAVFLVHL
ATIPITGTGINPARSLGAAIIYNKDKAWDDHWIFWVGPFIGAALAALYHVVVIRAIPFKK*

***Oeu*PIP1;1c *Oeu*038802.2**

Observation: Protein 100% Homolog to *Oeu*038800.1
Corrected as follow : Exon 1 upstream extended with 63n

>*Oeu*PIP1;1c_*Oeu*038802.2 | chr19:7217774..7219459 forward **Original version**
ATGGAGAGCAAGGAGGAGGATGTACGAGTGGGAGCCAACAAATTCGCCGAGAGGCAGCCGATTGGCACGGCTGCT
CAAAGCCAGGACAAGGACTACAAGGAACCACCACCGGCGCCACTTTTCGAGCCGGGTGAGCTGTCCTCGTGGTCG
TTCTACAGAGCTGGCATAGCAGAGTTCATAGCCACTTTCTTGTTCCTTTATATTACAGTTTTGACAGTGATGGGA
GTTAGTAAGTCTGATTCCAAGTGCAAAACTGTTGGAATCCAAGGTATTGCTTGGGCATTTGGTGGCATGATCTTT
GCCCTTGTTTACTGCACTGCAGGAATTTCAGGCAAGTTCTTTTCTATATATATAATTTGCAAATTTTGCTTGTAT
TTGCTATGATCTTCAACAGTAACGATCCGTCTTAAAGCTCAAAGAGGGAGTCGTCAGATTTGAAGATTGGTTTGT
GGGGTAGATATGAAAATACTAACCAACATATATATTGGATTATGGTCTCTGTCTCTGCAGTTTAGTCTTTGATAG
TATATGGAATTTAGGTTTTGCCATTAAAATTAGAAATTACCTAATTCAAAATTGAGTTGATTTTTCATGAATTTC
AGTTATAAGGTTTTAATTCCATTTGATATTAACATGGCAATTCAGAAAACCAAATCGTTTCAGCAATCAAATTTA
TGATGAACAATTTTAACAGTACTACTAACCTATTTTCTTCAAAGAAAAGGGCAAAATTTTAATGAACTGCTTGTT
TTGCAGGGGGGCATATAAATCCGGCAGTGACTTTCGGGCTGTTTTTGGGGAGGAAATTGTCGTTGACCCGGGCTG
TGTTCTACATGGTGATGCAGTGTCTTGGAGCCATATGTGGTGCTGGTGTGGTTAAGGGATTCGGAAAGACCCTGT
ACATGACCAAGGGTGGTGGTGCTAATGTAGTAGCCCACGGTTACACCAAGGGCGATGGCCTAGGTGCTGAAATAA

TCGGAACTTTTGTGCTCGTTTACACTGTCTTCTCTGCTACTGATGCCAAACGTAGCGCCAGAGACTCGCACGTTC
CTGTAAGCACTCCTCTTGTCTTTGTAGATTGAGATTGAACAAGAATTTGCTTGTGTATCTGTAATCAAATTGTCT
TTTCGTTTTGTAAGAACTGATGAATCAAAAAATGTCATTGTTGATCAAGAAAAAGTTTGATTAATTGGATTCTCA
TATGCTGTAATTCAATGGTTTTCAGATATTGGCCCCCTTGCCTATTGGGTTTGCTGTGTTCTTGGTGCACTTGGC
AACCATCCCTATTACCGGTACAGGTATTAACCCCGCCAGGAGTCTTGGTGCAGCAATCATCTACAACAAAGACAA
GGCATGGGATGATCACGTAAGCTAAAAATTCAACTGCCTGAATTCAACAATTGAAATATTTGAGTTAAGGGGGGC
ATACCTAGACAATTATATCCTAACAAATTTTTACGTTGTTCACTTATCACAATCCTCCTTTTATCTATTTTGGGA
CTACGACGAAATTTTTTAATAATCTGGCAGATGAGATGATAAGATTGTCTAAAGTTTGTTCTCACTCTTATCTTG
CTCTGATATGATAATATGCAGTGGATATTCTGGGTTGGACCGTTCATTGGGGCAGCACTTGCAGCTCTATACCAT
GTGGTGGTGATCCGAGCCATCCCATTCAAAAAATAA

>*Oeu*PIP1;1c_*Oeu*038802.2 Corrected version

ATGGAGAGCAAGGAGGAGGATGTACGAGTGGGAGCCAACAAATTCGCCGAGAGGCAGCCGATTGGCACGGCTGCT
CAAAGCCAGGACAAGGACTACAAGGAACCACCACCGGCGCCACTTTTCGAGCCGGGTGAGCTGTCCTCGTGGTCG
TTCTACAGAGCTGGCATAGCAGAGTTCATAGCCACTTTCTTGTTCCTTTATATTACAGTTTTGACAGTGATGGGA
GTTAGTAAGTCTGATTCCAAGTGCAAAACTGTTGGAATCCAAGGTATTGCTTGGGCATTTGGTGGCATGATCTTT
GCCCTTGTTTACTGCACTGCAGGAATTTCAGGCAAGTTCTTTTCTATATATATAATTTGCAAATTTTGCTTGTAT
TTGCTATGATCTTCAACAGTAACGATCCGTCTTAAAGCTCAAAGAGGGAGTCGTCAGATTTGAAGATTGGTTTGT
GGGGTAGATATGAAAATACTAACCAACATATATATTGGATTATGGTCTCTGTCTCTGCAGTTTAGTCTTTGATAG
TATATGGAATTTAGGTTTTGCCATTAAAATTAGAAATTACCTAATTCAAAATTGAGTTGATTTTTCATGAATTTC
AGTTATAAGGTTTTAATTCCATTTGATATTAACATGGCAATTCAGAAAACCAAATCGTTTCAGCAATCAAATTTA
TGATGAACAATTTTAACAGTACTACTAACCTATTTTCTTCAAAGAAAAGGGCAAAATTTTAATGAACTGCTTGTT
TTGCAGGGGGGCATATAAATCCGGCAGTGACTTTCGGGCTGTTTTTGGGGAGGAAATTGTCGTTGACCCGGGCTG
TGTTCTACATGGTGATGCAGTGTCTTGGAGCCATATGTGGTGCTGGTGTGGTTAAGGGATTCGGAAAGACCCTGT
ACATGACCAAGGGTGGTGGTGCTAATGTAGTAGCCCACGGTTACACCAAGGGCGATGGCCTAGGTGCTGAAATAA
TCGGAACTTTTGTGCTCGTTTACACTGTCTTCTCTGCTACTGATGCCAAACGTAGCGCCAGAGACTCGCACGTTC
CTGTAAGCACTCCTCTTGTCTTTGTAGATTGAGATTGAACAAGAATTTGCTTGTGTATCTGTAATCAAATTGTCT
TTTCGTTTTGTAAGAACTGATGAATCAAAAAATGTCATTGTTGATCAAGAAAAAGTTTGATTAATTGGATTCTCA
TATGCTGTAATTCAATGGTTTTCAGATATTGGCCCCCTTGCCTATTGGGTTTGCTGTGTTCTTGGTGCACTTGGC
AACCATCCCTATTACCGGTACAGGTATTAACCCCGCCAGGAGTCTTGGTGCAGCAATCATCTACAACAAAGACAA
GGCATGGGATGATCACGTAAGCTAAAAATTCAACTGCCTGAATTCAACAATTGAAATATTTGAGTTAAGGGGGGC
ATACCTAGACAATTATATCCTAACAAATTTTTACGTTGTTCACTTATCACAATCCTCCTTTTATCTATTTTGGGA
CTACGACGAAATTTTTTAATAATCTGGCAGATGAGATGATAAGATTGTCTAAAGTTTGTTCTCACTCTTATCTTG
CTCTGATATGATAATATGCAGTGGATATTCTGGGTTGGACCGTTCATTGGGGCAGCACTTGCAGCTCTATACCAT
GTGGTGGTGATCCGAGCCATCCCATTCAAAAAATAA

>*Oeu*PIP1;1c_*Oeu*038802.2

ATGGAGAGCAAGGAGGAGGATGTACGAGTGGGAGCCAACAAATTCGCCGAGAGGCAGCCGATTGGCACGGCTGCT
CAAAGCCAGGACAAGGACTACAAGGAACCACCACCGGCGCCACTTTTCGAGCCGGGTGAGCTGTCCTCGTGGTCG
TTCTACAGAGCTGGCATAGCAGAGTTCATAGCCACTTTCTTGTTCCTTTATATTACAGTTTTGACAGTGATGGGA
GTTAGTAAGTCTGATTCCAAGTGCAAAACTGTTGGAATCCAAGGTATTGCTTGGGCATTTGGTGGCATGATCTTT
GCCCTTGTTTACTGCACTGCAGGAATTTCAGGGGGGCATATAAATCCGGCAGTGACTTTCGGGCTGTTTTTGGGG
AGGAAATTGTCGTTGACCCGGGCTGTGTTCTACATGGTGATGCAGTGTCTTGGAGCCATATGTGGTGCTGGTGTG
GTTAAGGGATTCGGAAAGACCCTGTACATGACCAAGGGTGGTGGTGCTAATGTAGTAGCCCACGGTTACACCAAG
GGCGATGGCCTAGGTGCTGAAATAATCGGAACTTTTGTGCTCGTTTACACTGTCTTCTCTGCTACTGATGCCAAA
CGTAGCGCCAGAGACTCGCACGTTCCTATATTGGCCCCCTTGCCTATTGGGTTTGCTGTGTTCTTGGTGCACTTG
GCAACCATCCCTATTACCGGTACAGGTATTAACCCCGCCAGGAGTCTTGGTGCAGCAATCATCTACAACAAAGAC
AAGGCATGGGATGATCACTGGATATTCTGGGTTGGACCGTTCATTGGGGCAGCACTTGCAGCTCTATACCATGTG
GTGGTGATCCGAGCCATCCCATTCAAAAAATAA

>OeuPIP1;1c_Oeu038802.2

MESKEEDVRVGANKFAERQPIGTAAQSQDKDYKEPPPAPLFEPGELSSWSFYRAGIAEFIATFLFLYITVLTVMG
VSKSDSKCKTVGIQGIAWAFGGMIFALVYCTAGISGGHINPAVTFGLFLGRKLSLTRAVFYMVMQCLGAICGAGV
VKGFGKTLYMTKGGGANVVAHGYTKGDGLGAEIIGTFVLVYTVFSATDAKRSARDSHVPILAPLPIGFAVFLVHL
ATIPITGTGINPARSLGAAIIYNKDKAWDDHWIFWVGPFIGAALAALYHVVVIRAIPFKK*

***Oeu*PIP1;2 *Oeu*018133.1**

Observation: Protein 100% Homolog to PIP1.2 A0A1D5AHZ6 from UniProtKB

>*Oeu*PIP1;2_*Oeu*018133.1 | Scaffold17619:410..2065 forward

ATGGAGAGCAAAGAGGAAGATGTACGAGTGGGAGCCAACAAATTCAACGAGAGGCAGCCGATTGGCACGGCGGCG
CAATCCCAAGACAAGGACTACAAGGAGCCACCACCGGCGCCGCTTTTCGAGCCCGGCGAGCTGTCCTCATGGTCG
TTCTACAGAGCTGGCATTGCCGAGTTCATAGCCACATTCCTGTTCCTGTATATTACAATTTTGACAGTGATGGGA
GTTAGCAAGTCTGATTCTAAGTGCTCTACTGTTGGAATCCAAGGTATTGCTTGGGCATTCGGTGGCATGATCTTT

GCCCTTGTTTACTGCACCGCTGGAATTTCAGGCAAGTTCTTTACCTATAAATGGTTCGCATATTTGGCTTCTATA
TACCTTGATCTTTAACCTTAATATCTCATACGAGTAATCGATCTGTCTAAAACTCTAAAATTGAGTTAGATTTCA
GGATTGGCGTTCTCAGTTGGGTTGCTCCGTCTTGATGATGAGGTTGAAGTTTAACATGGAATCGTCTGTGTCTCT
GTAGTTAGTCTTTGATAGTAGCTTTGGGAATCAAAATAAGAAATTACCTAATTCAAAATTGAGTTGATTTTATAT
TAATTTCTGTTCTGAATAACAATTATAACAATATTAACTTATTTTCTTCAAAGAAAATGGCATAATTATTACTGG
AAAAGAAAGCAAGTTGTATGACCAGTAACTTATTGACGAGTTGTTTGCTGTGCAGGGGGGCATATAAACCCGGCA
GTGACATTTGGGCTGTTTTTGGCCAGGAAATTGTCATTGACTCGGGCTATATTCTACATGGTGATGCAGTGCCTA
GGAGCCATATGTGGTGCTGGTGTGGTTAAGGGGTTCGGAAAGACCCTGTACATGTCCAAGGGTGGTGGTGCTAAT
GTTGTAGCTCACGGTTACACCAAGGGTAGTGGCCTAGGTGCTGAAATTATAGGTACCTTCGTTCTCGTTTACACC
GTTTTCTCTGCTACTGATGCCAAACGTAGCGCCAGAGACTCTCACGTTCCAGTAAGCACTCCTCTTTTCTTTTTT
GGCTAGTTTCTCTTGAAATTTTTCATTTGTACAGGTTGTAGATATTGATCAATCAAAGCACAACATTATTGACTC
AAAAAAGTTTGATTAATTGGACTCCCTAATTCTGTAATTCTATGGTTTTCAGATATTAGCCCCCTTGCCTATTGG
GTTTGCCGTGTTCTTGGTGCACTTGGCGACCATCCCTATTACAGGTACTGGTATTAACCCCGCCAGGAGTCTTGG
TGCAGCAATCATCTACAACAAAGACAAGGCATGGGATGATCACGTAAGCTAAAATTCAACTGTATCTGAATTCAG
TATAATCAGGATTCTACTATTGAAATATTCGAGTTAAGGACTCACTCAGACAATTATATCCTAACTTACACTACA
AGTCGTGTTAAATTTGGCGTGTATAAGGTGAATGGTAAAATGTGACATATTTTTACGTTGTTAACTTACCACAAC
CCTCCTATTTTAGGATGATAAGGTTGGATAAAGTTAGTTATCTTTTTTCCTGATAATGTGCAGTGGATATTCTGG
GTTGGACCATTCATTGGGGCAGCACTTGCAGCTCTCTACCATGTGGTTGTGATAAGAGCCATCCCATTCAAGAAT
AAG**TGA**

>*Oeu*PIP1;2_*Oeu*018133.1

ATGGAGAGCAAAGAGGAAGATGTACGAGTGGGAGCCAACAAATTCAACGAGAGGCAGCCGATTGGCACGGCGGCG
CAATCCCAAGACAAGGACTACAAGGAGCCACCACCGGCGCCGCTTTTCGAGCCCGGCGAGCTGTCCTCATGGTCG
TTCTACAGAGCTGGCATTGCCGAGTTCATAGCCACATTCCTGTTCCTGTATATTACAATTTTGACAGTGATGGGA
GTTAGCAAGTCTGATTCTAAGTGCTCTACTGTTGGAATCCAAGGTATTGCTTGGGCATTCGGTGGCATGATCTTT
GCCCTTGTTTACTGCACCGCTGGAATTTCAGGGGGGCATATAAACCCGGCAGTGACATTTGGGCTGTTTTTGGCC
AGGAAATTGTCATTGACTCGGGCTATATTCTACATGGTGATGCAGTGCCTAGGAGCCATATGTGGTGCTGGTGTG
GTTAAGGGGTTCGGAAAGACCCTGTACATGTCCAAGGGTGGTGGTGCTAATGTTGTAGCTCACGGTTACACCAAG
GGTAGTGGCCTAGGTGCTGAAATTATAGGTACCTTCGTTCTCGTTTACACCGTTTTCTCTGCTACTGATGCCAAA
CGTAGCGCCAGAGACTCTCACGTTCCAATATTAGCCCCCTTGCCTATTGGGTTTGCCGTGTTCTTGGTGCACTTG
GCGACCATCCCTATTACAGGTACTGGTATTAACCCCGCCAGGAGTCTTGGTGCAGCAATCATCTACAACAAAGAC
AAGGCATGGGATGATCACTGGATATTCTGGGTTGGACCATTCATTGGGGCAGCACTTGCAGCTCTCTACCATGTG
GTTGTGATAAGAGCCATCCCATTCAAGAATAAG**TGA**

>*Oeu*PIP1;2_*Oeu*018133.1

MESKEEDVRVGANKFNERQPIGTAAQSQDKDYKEPPPAPLFEPGELSSWSFYRAGIAEFIATFLFLYITILTVMG
VSKSDSKCSTVGIQGIAWAFGGMIFALVYCTAGISGGHINPAVTFGLFLARKLSLTRAIFYMVMQCLGAICGAGV
VKGFGKTLYMSKGGGANVVAHGYTKGSGLGAEIIGTFVLVYTVFSATDAKRSARDSHVPILAPLPIGFAVFLVHL
ATIPITGTGINPARSLGAAIIYNKDKAWDDHWIFWVGPFIGAALAALYHVVVIRAIPFKNK*

***Oeu*PIP1;3 *Oeu*042329.1**

Observation: Protein 100% Homolog to PIP1.3 A0A1D5AHZ7 from UniProtKB

>*Oeu*PIP1;3_*Oeu*042329.1 | chr13:11012950..11015602 forward

ATGGCAGAAAGCAAGGAAGAAGATGTTAAACTTGGAGCCAACAAGTTCACAGAGAAGCAGCCATTAGGCACAGCG
GCTCAAACAGACAAGGACTATAAGGAGCCACCACCAGCACCTTTATTTGAGCCTGGAGAATTGACATCATGGTCC
TTTTACAGGGCTGGAATTGCAGAATTCATAGCCACTTTCTTGTTCTTGTACATATCTATTTTGACTGTTATGGGT
GTTTTCAGTGCAAAGAACAAGTGTGCCTCTGTGGGCGTCCAGGGAATTGCTTGGGCCTTTGGGGGTATGATCTTT
GCACTTGTCTACTGTACTGCTGGTATCTCAGGTCAGTCCCTAAAACCCCTTTCCTATCTTTACTTTTAATTTACC
CTTTTTTTTTTCAAATTGCTTAATTTTTCTTTTTCTTTTTTATTTTTGGACTTCTCTCTGTATATCTTTTTTTT
TAGTCCTTCTTTTTACGATCTTTGACTCAAAAAAGTTCTTCAATCGGTTTGTGGGTCTTTAGAGATTCTTGAGGA
AATTTATTCAGTTGAGTAGTAGTGATTTGTGTGTTCCTTCTTTGTATTGGAAAATTTTGTGGCTTTTTATTGTTC
TGTTTATCTGTATAAGTGCTTAGTTTAACCACGGGCTAGCTACATCGATGATATTTGGAACTAAAGATTGTATCT
TTGTGATTTCTTGAAATTTTTTTGACTTTTTTCAGTGTAAGCTGTTTGGCTAAGCTTAAAATGTTTGCTTATAAG
CAAATAAGTATTAAAATAATTACTAGCTTAGTAAGGTAAAGTGTATTAGGTATATACACTAACGTTTTTCTTCTA
CTTTGATGGTAAGGTAAAAATTGTTATGACCATGTTAACAAGGTTGATCTTATTAAGAGGAATGACTCGATATCT
ATCTAAGTTGGTCAATATTTCGATATTTAATCAATACGTGATTTATTCTCTTAACATGCTCCCGCAAAATAATGG
CTCTTTTTTAGCAGACCAATTTTGAAATTCGTGTTCTTATTTTTGAGACTTAATCGGATCGAATAGCTCGTTGGA
GTTACTGGGCTCTGATATCGTGTTAACAACCTTACTAGGAGTGGCCCAAAATCTATTTAAGTTGGTCAATATTTC
GGTATTTAACCGACGTGTGATTTATTCCCTTAACAAAAATTGTCAAAATATGTAAAAAAATTTATTTTTATAGCT
TAGCTAGACATAAGTGAGGGAAAAGTAAGATATTGAAACATTTTTGAAACGGAAATTTACTTATGAGCAACTTTT
AAACTACTTTGATGAAGCATATTTAAATTTTTCTTGTTTGCATGTTAAAACAGGAGGGCACATCAACCCAGCTGT
GACCTTTGGCTTGTTCTTGGCAAGGAAACTTTCTTTGATCAGGGCTTTATTTTACATGGTGATGCAGTGCCTGGG

TGCCATATGTGGTGCTGGTGTTGTTAAGGGGTTCATGAAGGGTCAATACGAGAGACTTCAAGGCGGTGCTAACAT
TGTGCAGCATGGCTACACCAAAGGCGATGCCCTTGGTGCGGAGATAGTTGGCACCTTTATTCTCGTCTACACTGT
CTTCTCGGCTACTGATGCCAAGAGAAATGCTAGAGACTCACACGTCCCGGTATGATTACCCATTCTTCGCTAATT
ACTTCGACTAACATCACTATTATTGACTAACATCACTATTATTGCTCGCTTAAAATTAATTAATTAATTAATTTT
TTATTATTATTATTATTATTATTATTATTATTGTTTTAATTTTATTTTTTTTAATTATTATTTTTTTTTTTT
TATGAAATTGCTGCAAAAGCTTAAGTTGAGAGGGCATATTGACTGATTTAAATTTAGAAGGCTGGTATATGCATG
ATTTTAGCGAAGGCTTGGCGATGAGACTTGAACTTATACCCCTGCAATTTAACATTGAGTGTTTTGACAAATTTC
ATTATTTAGTGCGTTGTGGATGCTAGGGGATCTATTATTGGTATGATGAATGTGCCAAAAACTTTTCGAAAATAT
AAGATAATACCGAAGCTGATCCTGTGAAATTTCTTGATTTTAAAACTTGGGATTTCATTATAAAATGTCAATCTT
GTTAAGAAGAATAGTCAAGCCCTTAACGTGCTCATTTTTGGAGTCTTAATTGGATCAAATGTCTCGTTGGAGCTT
AATAGGTTTATACCATATTAAAACTTGGGATTTCATTTTAAAAGGTTTATCTTGTTAAGAGATGCAGTTCTAACC
ATTATGACGTCCATGTTTTATGTGGAATTTTATCCCTTGACATACTGGTTCTTAAATATGTCTTCGTGATTGTTG
CAGCTTTTGGCTCCACTCCCCATTGGATTTGCAGTATTTTTGGTTCATTTGGCCACCATCCCCATTACTGGAACA
GGTATCAACCCTGCTAGGAGTCTCGGAGCTGCCATAATTTACAATAGAGACCACGCATGGGACGACCAGGTAAGA
TTCTTGCTATACATTACATATAACACCTGTATCTATTCCAATGTTTTTTCTTGACTAAATTACGCGATTTCTTTA
TTTACAGTGGATCTTCTGGGTTGGTCCCTTCATTGGAGCTGCTCTTGCTGCAATTTACCACCAGATTGTCATCAG
AGCCATTCCTTTCAAGAGCAGGGCT**TAA**

>*Oeu*PIP1;3_*Oeu*042329.1

ATGGCAGAAAGCAAGGAAGAAGATGTTAAACTTGGAGCCAACAAGTTCACAGAGAAGCAGCCATTAGGCACAGCG
GCTCAAACAGACAAGGACTATAAGGAGCCACCACCAGCACCTTTATTTGAGCCTGGAGAATTGACATCATGGTCC
TTTTACAGGGCTGGAATTGCAGAATTCATAGCCACTTTCTTGTTCTTGTACATATCTATTTTGACTGTTATGGGT
GTTTTCAGTGCAAAGAACAAGTGTGCCTCTGTGGGCGTCCAGGGAATTGCTTGGGCCTTTGGGGGTATGATCTTT
GCACTTGTCTACTGTACTGCTGGTATCTCAGGAGGGCACATCAACCCAGCTGTGACCTTTGGCTTGTTCTTGGCA
AGGAAACTTTCTTTGATCAGGGCTTTATTTTACATGGTGATGCAGTGCCTGGGTGCCATATGTGGTGCTGGTGTT
GTTAAGGGGTTCATGAAGGGTCAATACGAGAGACTTCAAGGCGGTGCTAACATTGTGCAGCATGGCTACACCAAA
GGCGATGCCCTTGGTGCGGAGATAGTTGGCACCTTTATTCTCGTCTACACTGTCTTCTCGGCTACTGATGCCAAG
AGAAATGCTAGAGACTCACACGTCCCGCTTTTGGCTCCACTCCCCATTGGATTTGCAGTATTTTTGGTTCATTTG
GCCACCATCCCCATTACTGGAACAGGTATCAACCCTGCTAGGAGTCTCGGAGCTGCCATAATTTACAATAGAGAC
CACGCATGGGACGACCAGTGGATCTTCTGGGTTGGTCCCTTCATTGGAGCTGCTCTTGCTGCAATTTACCACCAG
ATTGTCATCAGAGCCATTCCTTTCAAGAGCAGGGCT**TAA**

>*Oeu*PIP1;3_*Oeu*042329.1

MAESKEEDVKLGANKFTEKQPLGTAAQTDKDYKEPPPAPLFEPGELTSWSFYRAGIAEFIATFLFLYISILTVMG
VFSAKNKCASVGVQGIAWAFGGMIFALVYCTAGISGGHINPAVTFGLFLARKLSLIRALFYMVMQCLGAICGAGV
VKGFMKGQYERLQGGANIVQHGYTKGDALGAEIVGTFILVYTVFSATDAKRNARDSHVPLLAPLPIGFAVFLVHL
ATIPITGTGINPARSLGAAIIYNRDHAWDDQWIFWVGPFIGAALAAIYHQIVIRAIPFKSRA*

*Oeu*PIP1;4 Oeu052954.1

>*Oeu*PIP1;4_*Oeu*052954.1 | chr3:21471493..21473698 reverse

ATGGAGGGTAAAGAGGAGGATGTGAGGCTAGGAGCCAACAAGTTCACAGAGAGGCAACCATTGGGTACCTCAGCA
CAGAGCAAGGACTACAAGGAGCCACCTCCAGCTCCACTTTTTGAGCCTGGTGAACTCCAGTCATGGTCCTTTTGG
AGAGCTGGGATTGCTGAGTTTATAGCTACTTTCTTGTTTCTTTATATCACAATCTTGACTGTAATGGGTGTCAGC
AGGTCTGGCAGCAAGTGTGCTTCTGTGGGTATTCAGGGCATTGCTTGGGCCTTTGGTGGCATGATTTTTGCCCTT
GTCTACTGCACTGCTGGCATCTCAGGTCCTTCTCTCTCTCTCTCTCTCTCTCTCTCTCTCTCTCTCTCTCTCT
CAATATCTCTGTGTTAGTGTGTTCTATATTCTGTTGCTTAAGTAAATTTCGAGTCCGTTGTGTTTGGTGTGCAAG
ATTTTAACTCCTAAACATGCCAAGAATGTACTACTAAACTAATTTTAAGTCTTCATTTTCATGGGATCTTGGAAA
ATATGAAGTATGAAGCTTCTGTTAGTGTTCTATATTCTGTTCTCAAGTAAATATAGAGTTTAGTCTGTGTTTAGT
ATGGAGTATTGGATGGAAGTGAACTGTATAATGAACAAGATTTTATCTTCGAACATACCAAGCAATACTACTAGA
TCTGGGTCCTCATTTTCATGGGATCTTGGAAAGATGTAAATTATGTAGCTTCTTTTTGTGTTCGGTAATCTGTTG
CTTAAGTAACTCTAGAGTTTTGACTAGTATAATGAGCAAGATTTTATTTTCTATATCTAACTAGCCACAAATGTA
CTAGTTGAGGATTTTCTTTCTTTCTTTCCTTTTTTTTTTTTTTTTGGTTGGAATTCATTGTTGTTTTTCAGGTTT
TAAATCTTGAATGTGGTCTTGTAATGTAGTTCAAACCCTGCTCTTCTCTTCAAAATCTGCACCTTGTGTTAGCAG
CTTATTAATTTAATGGGATGTAGGGTTTTGTGCTGACATTTCTTTATTGTGATAATGATTAATGCCCTTGTTTGA
AAATTATTGAACTTTGTTTATGTACTGATGTTGGAATTTTTTATGGGTGATTTCAGGTGGACACATAAATCCTGC
AGTAACATTTGGTCTGTTTCTAGCAAGAAAATTGTCTCTAACCAGAGCAATTTTTTACATCGTGATGCAATGTCT
CGGAGCAATCTGTGGTGCTGGTGTCGTAAAGGGCTTCCAGCCTTCTTTTTACAAGGCTAATGGTGGTGGTGCTAA
TGTTGTTTCCCATGGTTACACCAAGGGCGATGGACTTGGTGCTGAGATTATTGGCACTTTTGTTCTTGTTTACAC
TGTCTTTTCTGCTACTGATGCCAAGAGAAATGCCAGAGACTCACATGTCCCTGTAAGTATTTCTTTGCAATCTTA
TTTTTCTTGCCTGTTTTGTACTCATAGCATTTTGTTTATTGGTATTATTTTCGCTTTCTGTATGATTGCCCTTT
CTTACACACAAGGCATGTGCCTTATCTGTAATGTGTTCGAATGTCTCATGAATTTGGTTATACTTTTCTAATTGA
AAAAAGGATTGCAATGCTGGAAGTCCATATAGTCTTGGCATGGTTAGGACTTAGGAGCAATATCAATTTCTGCGC
ACGTCTTTTTTGTGATTCAGGAAGATGGAAATGAATTTCGAGGTTTTTCCGTAGTGTATGATGCTTTACATGATA

TATTATGGAGCCTTACGATAGGCGTAGCCTTTGATTTTTCTTCTAGTACATAGTTGGGCTAGAAATCAGTAATTT
ATGCCAAAACTTGAATCTTAACGTGGGATGCCAAATTGATGACTCCTTTGATCTTCCAAATTTCACAGATTTTGG
CTCCACTCCCAATTGGATTTGCCGTCTTTTTGGTTCACTTGGCAACCATCCCCATCACCGGAACTGGCATCAACC
CAGCCAGAAGCCTTGGCGCCGCCATCATTTACAACAAAGATCATGCTTGGGATGATCACGTAAGTTTACTTCATT
AACTTCCACAGACAATCTTCTTACCTGTTCATGACATTTTTGGCGTCCATACATAAACATTTTTGTCACATGTGC
CTTTCTTCAGTGGATCTTCTGGGTCGGACCTTTCATTGGAGCTGCCCTTGCTGCACTTTACCATCAGGTTATCAT
TCGAGCCATCCCATTTAAGAGTAGCAGC**TGA**

>*Oeu*PIP1;4_*Oeu*052954.1

ATGGAGGGTAAAGAGGAGGATGTGAGGCTAGGAGCCAACAAGTTCACAGAGAGGCAACCATTGGGTACCTCAGCA
CAGAGCAAGGACTACAAGGAGCCACCTCCAGCTCCACTTTTTGAGCCTGGTGAACTCCAGTCATGGTCCTTTTGG
AGAGCTGGGATTGCTGAGTTTATAGCTACTTTCTTGTTTCTTTATATCACAATCTTGACTGTAATGGGTGTCAGC
AGGTCTGGCAGCAAGTGTGCTTCTGTGGGTATTCAGGGCATTGCTTGGGCCTTTGGTGGCATGATTTTTGCCCTT
GTCTACTGCACTGCTGGCATCTCAGGTGGACACATAAATCCTGCAGTAACATTTGGTCTGTTTCTAGCAAGAAAA
TTGTCTCTAACCAGAGCAATTTTTTACATCGTGATGCAATGTCTCGGAGCAATCTGTGGTGCTGGTGTCGTAAAG
GGCTTCCAGCCTTCTTTTTACAAGGCTAATGGTGGTGGTGCTAATGTTGTTTCCCATGGTTACACCAAGGGCGAT
GGACTTGGTGCTGAGATTATTGGCACTTTTGTTCTTGTTTACACTGTCTTTTCTGCTACTGATGCCAAGAGAAAT
GCCAGAGACTCACATGTCCCTATTTTGGCTCCACTCCCAATTGGATTTGCCGTCTTTTTGGTTCACTTGGCAACC
ATCCCCATCACCGGAACTGGCATCAACCCAGCCAGAAGCCTTGGCGCCGCCATCATTTACAACAAAGATCATGCT
TGGGATGATCACTGGATCTTCTGGGTCGGACCTTTCATTGGAGCTGCCCTTGCTGCACTTTACCATCAGGTTATC
ATTCGAGCCATCCCATTTAAGAGTAGCAGC**TGA**

>*Oeu*PIP1;4_*Oeu*052954.1

MEGKEEDVRLGANKFTERQPLGTSAQSKDYKEPPPAPLFEPGELQSWSFWRAGIAEFIATFLFLYITILTVMGVS
RSGSKCASVGIQGIAWAFGGMIFALVYCTAGISGGHINPAVTFGLFLARKLSLTRAIFYIVMQCLGAICGAGVVK
GFQPSFYKANGGGANVVSHGYTKGDGLGAEIIGTFVLVYTVFSATDAKRNARDSHVPILAPLPIGFAVFLVHLAT
IPITGTGINPARSLGAAIIYNKDHAWDDHWIFWVGPFIGAALAALYHQVIIRAIPFKSSS*

***Oeu*PIP1;5 *Oeu*061384.2**

Corrected as follow : Third predicted Exon as part of the intron, generating introns 2 and 3 as one. Ultimately, this gene including only 4 introns.

>*Oeu*PIP1;5_*Oeu*061384.2 | Scaffold87:524214..526575 reverse Original version

ATGGCAGAAAACAGGGAAGAGGATGTTAGGCTTGGAGCCAACAAGTTTACTGAGAAGCAGCCTTTGGGTACAGCT
GCTCAAACAGACAAGGACTACAAGGAGCCACCTCCAGCCCCTTTGTTTGAGCCTGGGGAGATTAAATCATGGTCT
TTTTACAGGGCTGGTATTGCAGAGTTTGTGGCTACCTTCTTGTTCTTGTACATTACCATTTTGACTGTCATGGGG
GTTGGTAGATCAAATAGTAAGTGTGCCTCTGTGGGCATCCAGGGGATTGCTTGGTCCTTTGGTGGCATGATTTTT
GCACTTGTATACTGCACTGCTGGGATTTCAGGTTAGTTTATTTTTGTTTTTTTAATCTCACTTAATCTTGTTTTT
TCTTTGCATGTTTCTGTTTGTTTCTTTTACTGTGAGTCTTGGCAAAATTGTGTATTTCCTTTGTTTTTATGGGTT
TCTGGGGTTTCGTTTTGCCAGATTGATTATGAATTATTGTTACTGGATCTTGTTAAATAAGTTTTTAATTGATCC
ATTTAGATATTATTTGCGATAAAAGCTAGGATCTTGGTGATTTTTTCAGGAAACTCCAGTTTTAGATTATATGCT
ATGGCTTAGGGTACCCGCACCCTAACCCCTCTTAGACCATTGTCTATTATATGTAGCTTCAAAACTGATTTTCTG
TATTGGCCGTTGAACAGGAGGGCACATCAACCCTGCTGTGACCTTCGGTTTGTTCTTGGCAAGGAAGCTTTCCTT
GACCAGAGCTATCTTCTACATAGTGATGCAGTGCCTTGGTGCAATCTGTGGTGCTGGTGTTGTCAAGGGATTCAT
GGAGGGTCCATACCAGAGGCTACAAGGTGGGGCCAACGTTGTGCAGCATGGCTACACCAAGGGTGATGGCCTTGG
TGCTGAGATTGTTGGCACTTTTATTCTTGTTTACACTGTCTTCTCTGCCACTGATGCCAAGAGAAACGCCAGAGA
CTCGCATGTTCCAGTAGGTCTTCCTTTGTCGGTTATGCACTGATATTAACTTTTGGTTGCTTAAACTTGTTTGGA
AAGGCTGGATTTGGTGCCTTGATTTGTTTTTTGTTACTTCTAATTAAGCGTTCATACCCAAGAAAGGAAGAATCG
ATAATTTTATTATGTTTTGTGTAGCTGGAATGCTACATTTCTCTGATTTCATTTCGCTAATATTTGGTTTATATT
TATAAACTGTTTAGAAACGCTCAATTCACTTTATGTCCACACATTTCTAAGGACCCACAACCACAAGTACTGCCC
ACTCCAACGCGACTCATTTCTACCTATTTATAATTTAAACCATCTTATAAACAACTTTCCAAACATGCTTTTAGA
TGTTGAGCAATTTTGCTTATTTAGCCTCAGTTCATTCAATGCAATCTTGACATGATATGGGCCAATATCTTTAGT
TACTGAAATTCCGTTTAGACCAAAACTGACCCTATACTGAATGTCATGTATCATAGCACAAGGTTTGTCTTTCCA
AAGTATCGCCCCACGCAAAATTTTCTGTTTTGCTTTGATGATGCATTCAGCTTATTTGTTGGTTTATAAATCTAT
TGCCTGTCGTTAAGCAGGGGTGGAAGATGGGGTTAGGATGATGTGCAGTTTTGATAATTTTGATGTGCTTTTGGG
TAATGTGGCTTTTTAGCTGTCACATGGAACCCACAACTTAAAATATTTATTCTCATTCTATCTTTTGAGAAGAAA
GAGTTGTGAAGCAGTTGCATTAAGTGGTTTTATTCTCATCATAGGGAATGTTGGCAGCTCCAATTGGTCTTTTAG
CACCATAGGCACATAACACCATCAATTAATTGAGCTATTGATTTGGTCGAAGTCTCAAATTTTATTGTTATCACA
TGGACATTAATGTAGACTACGATTCTTGGTAATATAATTGTGTGATTGTACATAATGATTCGGAGAAACAACAAA
AGCTCATGACGATTAGCTTCTTGATTTTGATAAGAGAATTCATGTAGTAGTTCTAAAATTCTTTTATGTTTCAGC
TTTTGGCTCCTCTCCCCATTGGGTTTGCAGTATTTTTGGTTCATCTGGCCACCATCCCCATCACTGGAACCAGCA

TCAACCCTGCCAGGAGTCTTGGAGCTGCCATTATTTACAACAGAGATAACGCATGGGACGACCATGTAAGAATCT
CTCTATGCAGATCCTTTATAACTCTCGTAATTATTAATCAGTAGTACATTTTCAACTAAAACCATTGTTTCTTTA
CGTCGTCCAGTGGATCTTCTGGGTCGGACCCTTCATTGGAGCTGCTCTTGCTGCTATTTACCACCAGATTGTGAT
CAGAGCCATTCCTTTCAAGAGCAGCCAGGCCTGT**TAA**
>*Oeu*PIP1;5_*Oeu*061384.2 Corrected version
ATGGCAGAAAACAGGGAAGAGGATGTTAGGCTTGGAGCCAACAAGTTTACTGAGAAGCAGCCTTTGGGTACAGCT
GCTCAAACAGACAAGGACTACAAGGAGCCACCTCCAGCCCCTTTGTTTGAGCCTGGGGAGATTAAATCATGGTCT
TTTTACAGGGCTGGTATTGCAGAGTTTGTGGCTACCTTCTTGTTCTTGTACATTACCATTTTGACTGTCATGGGG
GTTGGTAGATCAAATAGTAAGTGTGCCTCTGTGGGCATCCAGGGGATTGCTTGGTCCTTTGGTGGCATGATTTTT
GCACTTGTATACTGCACTGCTGGGATTTCAGGTTAGTTTATTTTTGTTTTTTTAATCTCACTTAATCTTGTTTTT
TCTTTGCATGTTTCTGTTTGTTTCTTTTACTGTGAGTCTTGGCAAAATTGTGTATTTCCTTTGTTTTTATGGGTT
TCTGGGGTTTCGTTTTGCCAGATTGATTATGAATTATTGTTACTGGATCTTGTTAAATAAGTTTTTAATTGATCC
ATTTAGATATTATTTGCGATAAAAGCTAGGATCTTGGTGATTTTTTCAGGAAACTCCAGTTTTAGATTATATGCT
ATGGCTTAGGGTACCCGCACCCTAACCCCTCTTAGACCATTGTCTATTATATGTAGCTTCAAAACTGATTTTCTG
TATTGGCCGTTGAACAGGAGGGCACATCAACCCTGCTGTGACCTTCGGTTTGTTCTTGGCAAGGAAGCTTTCCTT
GACCAGAGCTATCTTCTACATAGTGATGCAGTGCCTTGGTGCAATCTGTGGTGCTGGTGTTGTCAAGGGATTCAT
GGAGGGTCCATACCAGAGGCTACAAGGTGGGGCCAACGTTGTGCAGCATGGCTACACCAAGGGTGATGGCCTTGG
TGCTGAGATTGTTGGCACTTTTATTCTTGTTTACACTGTCTTCTCTGCCACTGATGCCAAGAGAAACGCCAGAGA
CTCGCATGTTCCAGTAGGTCTTCCTTTGTCGGTTATGCACTGATATTAACTTTTGGTTGCTTAAACTTGTTTGGA
AAGGCTGGATTTGGTGCCTTGATTTGTTTTTTGTTACTTCTAATTAAGCGTTCATACCCAAGAAAGGAAGAATCG
ATAATTTTATTATGTTTTGTGTAGCTGGAATGCTACATTTCTCTGATTTCATTTCGCTAATATTTGGTTTATATT
TATAAACTGTTTAGAAACGCTCAATTCACTTTATGTCCACACATTTCTAAGGACCCACAACCACAAGTACTGCCC
ACTCCAACGCGACTCATTTCTACCTATTTATAATTTAAACCATCTTATAAACAACTTTCCAAACATGCTTTTAGA
TGTTGAGCAATTTTGCTTATTTAGCCTCAGTTCATTCAATGCAATCTTGACATGATATGGGCCAATATCTTTAGT
TACTGAAATTCCGTTTAGACCAAAACTGACCCTATACTGAATGTCATGTATCATAGCACAAGGTTTGTCTTTCCA
AAGTATCGCCCCACGCAAAATTTTCTGTTTTGCTTTGATGATGCATTCAGCTTATTTGTTGGTTTATAAATCTAT
TGCCTGTCGTTAAGCAGGGGTGGAAGATGGGGTTAGGATGATGTGCAGTTTTGATAATTTTGATGTGCTTTTGGG
TAATGTGGCTTTTTAGCTGTCACATGGAACCCACAACTTAAAATATTTATTCTCATTCTATCTTTTGAGAAGAAA
GAGTTGTGAAGCAGTTGCATTAAGTGGTTTTATTCTCATCATAGGGAATGTTGGCAGCTCCAATTGGTCTTTTAG
CACCATAGGCACATAACACCATCAATTAATTGAGCTATTGATTTGGTCGAAGTCTCAAATTTTATTGTTATCACA
TGGACATTAATGTAGACTACGATTCTTGGTAATATAATTGTGTGATTGTACATAATGATTCGGAGAAACAACAAA
AGCTCATGACGATTAGCTTCTTGATTTTGATAAGAGAATTCATGTAGTAGTTCTAAAATTCTTTTATGTTTCAGC
TTTTGGCTCCTCTCCCCATTGGGTTTGCAGTATTTTTGGTTCATCTGGCCACCATCCCCATCACTGGAACCAGCA
TCAACCCTGCCAGGAGTCTTGGAGCTGCCATTATTTACAACAGAGATAACGCATGGGACGACCATGTAAGAATCT
CTCTATGCAGATCCTTTATAACTCTCGTAATTATTAATCAGTAGTACATTTTCAACTAAAACCATTGTTTCTTTA
CGTCGTCCAGTGGATCTTCTGGGTCGGACCCTTCATTGGAGCTGCTCTTGCTGCTATTTACCACCAGATTGTGAT
CAGAGCCATTCCTTTCAAGAGCAGCCAGGCCTGT**TAA**
>*Oeu*PIP1;5_*Oeu*061384.2
ATGGCAGAAAACAGGGAAGAGGATGTTAGGCTTGGAGCCAACAAGTTTACTGAGAAGCAGCCTTTGGGTACAGCT
GCTCAAACAGACAAGGACTACAAGGAGCCACCTCCAGCCCCTTTGTTTGAGCCTGGGGAGATTAAATCATGGTCT
TTTTACAGGGCTGGTATTGCAGAGTTTGTGGCTACCTTCTTGTTCTTGTACATTACCATTTTGACTGTCATGGGG
GTTGGTAGATCAAATAGTAAGTGTGCCTCTGTGGGCATCCAGGGGATTGCTTGGTCCTTTGGTGGCATGATTTTT
GCACTTGTATACTGCACTGCTGGGATTTCAGGAGGGCACATCAACCCTGCTGTGACCTTCGGTTTGTTCTTGGCA
AGGAAGCTTTCCTTGACCAGAGCTATCTTCTACATAGTGATGCAGTGCCTTGGTGCAATCTGTGGTGCTGGTGTT
GTCAAGGGATTCATGGAGGGTCCATACCAGAGGCTACAAGGTGGGGCCAACGTTGTGCAGCATGGCTACACCAAG
GGTGATGGCCTTGGTGCTGAGATTGTTGGCACTTTTATTCTTGTTTACACTGTCTTCTCTGCCACTGATGCCAAG
AGAAACGCCAGAGACTCGCATGTTCCACTTTTGGCTCCTCTCCCCATTGGGTTTGCAGTATTTTTGGTTCATCTG
GCCACCATCCCCATCACTGGAACCAGCATCAACCCTGCCAGGAGTCTTGGAGCTGCCATTATTTACAACAGAGAT
AACGCATGGGACGACCATTGGATCTTCTGGGTCGGACCCTTCATTGGAGCTGCTCTTGCTGCTATTTACCACCAG
ATTGTGATCAGAGCCATTCCTTTCAAGAGCAGCCAGGCCTGT**TAA**
>*Oeu*PIP1;5_*Oeu*061384.2
MAENREEDVRLGANKFTEKQPLGTAAQTDKDYKEPPPAPLFEPGEIKSWSFYRAGIAEFVATFLFLYITILTVMG
VGRSNSKCASVGIQGIAWSFGGMIFALVYCTAGISGGHINPAVTFGLFLARKLSLTRAIFYIVMQCLGAICGAGV
VKGFMEGPYQRLQGGANVVQHGYTKGDGLGAEIVGTFILVYTVFSATDAKRNARDSHVPLLAPLPIGFAVFLVHL
ATIPITGTSINPARSLGAAIIYNRDNAWDDHWIFWVGPFIGAALAAIYHQIVIRAIPFKSSQAC*

_Oeu_PIP1;6 _Oeu_046350.1

>*Oeu*PIP1;6_*Oeu*046350.1 | Scaffold4751:4496..7173 forward
ATGGAGGGTAAAGAGGAGGATGTAAGGCTAGGAGCCAACAAATTCACAGAGAGGCAGCCATTGGGTACCTCAGCA
CAGAGCAAGGACTACAAGGAGCCACCTCCAGCTCCACTTTTTGAGCCTGGTGAACTCCAATCATGGTCCTTTTGG
AGGGCTGGGATTGCTGAGTTTATGGCCACTTTCTTGTTCCTTTATATCACGATCTTGACTGTAATGGGTGTGAGC
AGGTCTAACAGTAAGTGTACTTCTGTGGGTATTCAGGGCATTGCTTGGGCCTTTGGTGGCATGATTTTTGCCCTT
GTCTACTGCACTGCCGGCATCTCAGGTCTCTGTCTCTCAATTTGTGTGTGTTCTGTTCTCTGTTACTTAAATAAA
TTTAGGGTCTGCTGTTTGGTAGTGGACTGGATTTATAATGAACAAGATTTAACTTCGAACTTGCCAAGAATGTAT
TAGTACTACTAATATTGAGTTCTCATTTTCATGGGATCTTGGAAAATGTAAAGCATGCAGCTTCTGTCTAAGTTC
TACTACCTGTTGCTTAAGTGAATCGAGAGTCTTGCTAGTTTGGTATGCAGTATTAGATGGAACTAGTAGTATAAA
GAACAAGATTATATCTTCGAACATGCCAAGAATGTTAATGCACCACTAATTATGAGTCCATATTTTCATGGGATC
TTGAAAAATGTAAGTATGCGGCTTCTTTTTGTTTTTTATAATCTGTTGCTTAAGTAAATATCGAGTCTTGTGTGT
TTCGTGTGTAGAAGTGGATGGAATTATATCACATAATGTACAAGTATGATCTTCAAACATGCCAAGAATGTACTA
CTAGTTTAAAAAAATCTCGAGTCTTGCATGCTGGATGGAACTATATAGTATAATGAACAAAATTTGACAACATGC
CAAGAATGTACCACTGGTTTAAGTAAATCTAGATTCTTGGTAAGCAATACCAGACAACATTGAATAGTGAACAAG
ATTTGATCTTTAAACATGACAAGAACGTCCTATTACTAGAGTCTTGCCTGTTTAATACGTAGTATACGATGAGAC
TAAATAATATAATGAACAAGATTGATCTTCTAACATGCCAAGAATATACTACTAAGTAAATCCAGAGTCTCGACT
GTTTGGTAGCAATATTGGACAAGACGATTTTATCCTCCAAAAAAGACAAGAATGTACTAATACTTTTGAACCCTC
ATTTTCATGGGATTTTGGCAAATGGAAAGTAGAGTAATTTGTTATTGTTTGTGATAATTTGCTGTTGTTTTTTGA
GTTTTAAGTCTTGAATGTCACAGAATTTAAACCCTGCTTCTTCTTTTTTCAAATCCTGCTAAAATTTAATGGTTT
TTGTTCTTTTAGTTCTACTCTCCTTGATGCTAAGCAAAAAATATTTCTTCAAATATGAGTTCAACTTATAATTCA
TACAAAAAAAGGCCAAATCTTTGATATAATTTCTGGAAAAGAACTTATGATAAGTGATTATAATGCCCTTGTTTA
AAGATTATTGGAGCTTTTCATGTGCTGATATTTGAAATTTTTTATGGGTAATTTCAGGTGGACACATAAATCCTG
CAGTAACATTTGGTCTATTCGTAGCAAGGAAATTGTCTCTGACCAGGGCAATTTTCTACATTGTGATGCAATGCC
TTGGCGCAATCTGCGGCGCGGGTGTCGTAAAGGGCTTCGGGCCTTCTTTTTACAAGGCTAACGGTGGTGGTGCTA
ATGTTGTTGCCCATGGTTACACTAAGGGTGATGGACTTGGTGCTGAGATTATTGGCACCTTTGTTCTTGTTTACA
CTGTCTTCTCTGCTACTGATGCCAAGAGAAATGCCAGAGACTCGCATGTCCCTGTGAGTATTTCTTTTTAATCGT
GATTTTGAAAAGTGTTTTACTTTCAATGCAGTTTAGTATTAGTATTATTTTTTATGCTGAGGAAGGTATCATAGA
TATTTTAACCATGTATTCTTGCCTCGTCGGCTATAGATTATGTTTGTGTTGGGAAAATTGGACTTTCTTCATTGA
GAGTGGGCAAGACCCGGTCTGCATTCTGTTTGATTGTGCTCGATCTGAAATTCGAGTTCTTGAAAAGATATGTTG
GGCTACGATGGAATGATTTGGTCAATCAATAGTGTGCTCGAGCACTTTGATTCGAAATTCTGAAGTGCTGATTCG
ACTTTCTTTTGCACTTTGATTCTTCTTCTATTTCATGGTCAAACTAGAAACGAGTAATTTTGCAGATTTAACTTG
GCCTCAACTGTATGATTTGTGCCATAACTAGAATCTCATTGTGGGATGCCATCTTGATGACTACTAATTTTTCGA
ATTTCATAGATTTTAGCTCCACTCCCAATTGGGTTTGCCGTCTTTTTGGTTCACTTGGCAACAATCCCCATCACT
GGAACCGGTATCAACCCAGCCCGAAGCCTTGGTGCTGCCATCATTTACAACAAAGATCACGCTTGGGACGACCAT
GTAAGTTTTACGTCTGTAACTTCCATAGACTATGTTCTTACCTGTTCATGACATATGATTAGGCGCCCGGCCGTA
CTTATATTTGTGTCACTTCTGTCTTTCTTCAGTGGATCTTTTGGGTCGGACCTTTCATCGGAGCTGCACTCGCTG
CCCTTTACCACCAGGTTATCATTAGAGCTATCCCATTCAAGAGTGGCAAC**TGA**

>*Oeu*PIP1;6_*Oeu*046350.1
ATGGAGGGTAAAGAGGAGGATGTAAGGCTAGGAGCCAACAAATTCACAGAGAGGCAGCCATTGGGTACCTCAGCA
CAGAGCAAGGACTACAAGGAGCCACCTCCAGCTCCACTTTTTGAGCCTGGTGAACTCCAATCATGGTCCTTTTGG
AGGGCTGGGATTGCTGAGTTTATGGCCACTTTCTTGTTCCTTTATATCACGATCTTGACTGTAATGGGTGTGAGC
AGGTCTAACAGTAAGTGTACTTCTGTGGGTATTCAGGGCATTGCTTGGGCCTTTGGTGGCATGATTTTTGCCCTT
GTCTACTGCACTGCCGGCATCTCAGGTGGACACATAAATCCTGCAGTAACATTTGGTCTATTCGTAGCAAGGAAA
TTGTCTCTGACCAGGGCAATTTTCTACATTGTGATGCAATGCCTTGGCGCAATCTGCGGCGCGGGTGTCGTAAAG
GGCTTCGGGCCTTCTTTTTACAAGGCTAACGGTGGTGGTGCTAATGTTGTTGCCCATGGTTACACTAAGGGTGAT
GGACTTGGTGCTGAGATTATTGGCACCTTTGTTCTTGTTTACACTGTCTTCTCTGCTACTGATGCCAAGAGAAAT
GCCAGAGACTCGCATGTCCCTATTTTAGCTCCACTCCCAATTGGGTTTGCCGTCTTTTTGGTTCACTTGGCAACA
ATCCCCATCACTGGAACCGGTATCAACCCAGCCCGAAGCCTTGGTGCTGCCATCATTTACAACAAAGATCACGCT
TGGGACGACCATTGGATCTTTTGGGTCGGACCTTTCATCGGAGCTGCACTCGCTGCCCTTTACCACAGGTTATCA
TTAGAGCTATCCCATTCAAGAGTGGCAAC**TGA**

>*Oeu*PIP1;6_*Oeu*046350.1
MEGKEEDVRLGANKFTERQPLGTSAQSKDYKEPPPAPLFEPGELQSWSFWRAGIAEFMATFLFLYITILTVMGVS
RSNSKCTSVGIQGIAWAFGGMIFALVYCTAGISGGHINPAVTFGLFVARKLSLTRAIFYIVMQCLGAICGAGVVK
GFGPSFYKANGGGANVVAHGYTKGDGLGAEIIGTFVLVYTVFSATDAKRNARDSHVPILAPLPIGFAVFLVHLAT
IPITGTGINPARSLGAAIIYNKDHAWDDHWIFWVGPFIGAALAALYHQVIIRAIPFKSGN*

PIP1-related sequences. Considered as Pseudogenes

*Oeu*022396.1 *Oeu*PIP1-related

>*Oeu*022396.1 scaffold1994:48682..48948 forward
Incomplete sequence

>*Oeu*022396.1
ATGAGAGCCATATGTGATGTTGGTATGGTTAAGGGATTCAGAAAGACCCTATACATGACCAAGGGTGGTGGTGCT
AATGTAGTAGCCTACAGTTACACCAAGGGTGATGCCCTAGGTGCTGAAATTGTCGTTACCTCTGCGCTCGTTTAC
AATATTTTCTCTTCTACTGATGCCAAACATAAAGCTAGAGACTCACAAGTTCCTGTAAGCATTCCACCTGTCTTT
GCAAGCCTGTTTCTCTTGACATTATTGATTAGACTTAAG**TGA**
>*Oeu*022396.1
ATGAGAGCCATATGTGATGTTGGTATGGTTAAGGGATTCAGAAAGACCCTATACATGACCAAGGGTGGTGGTGCT
AATGTAGTAGCCTACAGTTACACCAAGGGTGATGCCCTAGGTGCTGAAATTGTCGTTACCTCTGCGCTCGTTTAC
AATATTTTCTCTTCTACTGATGCCAAACATAAAGCTAGAGACTCACAAGTTCCTGTAAGCATTCCACCTGTCTTT
GCAAGCCTGTTTCTCTTGACATTATTGATTAGACTTAAG**TGA**
>*Oeu*022396.1
MRAICDVGMVKGFRKTLYMTKGGGANVVAYSYTKGDALGAEIVVTSALVYNIFSSTDAKHKARDSQVPVSIPPVF
ASLFLLTLLIRLK*

*Oeu*010785.1 *Oeu*PIP1-related

>*Oeu*010785.1 | chr3:8511236..8513098 reverse
Incomplete sequence

>*Oeu*010785.1
ATGGGAGCCATATGTGGCGTTGGTGTGGTCAAGGGATTTGGAAAGAACCTGTACATGACCGAAGGTGTTGGTGCT
AATGTTGTAGCCTACGGTTGCACCAAGGGTGATGCCCTAGGTGCTGAAATTGTTGGTACCTCTACACTCGTTTAC
ATTGTTTTCTCTACTACTGATGCCAAACATAGAGCCAGGGACTCACACGTTCCTGTAAGCACTCTTGTCTTTGTA
GGCTGTTTCTCTTGACATTATTGATTAGACTTAAGCGAATATAAATCATTCTTCATATTAGTTATGATTAAATGT
GATTGAGCTTGAACAAGAATTATCTTCTGTATCTGCAAGAATTGATAAATCAAAACATGATATTATTGATAACGA
AAAAGTTTGATTAATTGGATTTCCTAATACTCTAATTCAATGGTTTTCAAATATTGGCCTCCTTGCCCATTGGGT
TTGCCGTGTTCTTACCAATACAGGTATTAGTCGTGCCAAGAGTCTTGGTGCAGCAAACATCTACATTCAAGAGCA
GGCATGGGATGATCACGTAAGCTAGAAATTGAACTACTTACCTAGACAATTATATCCTAACAACCACTACATGTC
GTGTTAACTTTGACAGGGACGGAGCTAGACATTTGGTGTTGTGGGGTCAAAATTAAAATACAATTTCTTTTAGAA
CAACATATCATAATATGTTATTTGAACACTCGCTTCAACTACATTTGCTCTGCACTCGAAAATAAGGGTTTTT
ATTTAAAATATTTCTCCATGAATGTTACAAAATCAAATTCAATATTAGCAAGATATGAGCACGGCATCTAGATGA
GAGAATTTTCTATTTCAATTGAATAAATAGTAAGAAGAAACAAGATGACATCAACTTTTTTTTTTTTTTTTTAA
TACTATTAGAGACCAAAATCATACTTTCCAGATTCACACAAAATTTTATAGTTGTCTTCTTGATTTCTTATTTAT
AATTGCAACAATTAGATCCAAACCCCATAATTTAACCGGATAAGGATAAATGCATGATTCTCCAAAGGCATCAAA
TAATCACAAAAAAAATTATCCGAATTAGATATTGGTAATTTCCATAATATGGCAATGCCGACTTGTTTAGATGTT
TATGTTTTTTTCCTGTAGACTAGATAATAACTAATAAGCGATGGGGAATTGGGGATTATAGTTGAAAATATTTCT
CAAATCTCAAATTCAATTATTCAAATGAATTTTAGAATAAAAATATGTAAAATCAAACAAGTAGGGTATCGATTT
GTTATCTAAATGGAAGAAAGATCACAAAGTTAGCACAAGAAAGAAAATCTCAAAACGTGAAGGAATCGAAAGAAT
GGAGTTGGATAGGTGAAATAAAAATTAATGGGCCTGTGCATTTGGGCTTGCCAATAAACAATTGGACAATGTTAT
ATTTTAATCTTTAGTGTACCAAAAAAAAAATCCAGAAATTTGTGTGGTGTCAACGGACCCCACATGACCCCATGC
GGGTCCATCAGCGAACTTTAACATGTATAAAGGTTCGCATGGTAAGATGCGACAAATTTTTACGTTGTTCACTTA
CCACAACCCTCTTTTATTTATTTTTAGACAACTTTGAAAATTTTTGTAAAAATTGGCAAATGGGATTGATAAGAT
TGGCTAAAGTTTGTTCTCGCTCCCATCCTGCTCTGATAATATGCAATGGATATTCTGGGTTATTCCATTCATTAG
GGCTAGACTTTTAGCTCTGTAACATATGGTTTTGGTGGAAGAGACGATGACAGTGGAATGAAGAGAGGCGGTTAT
AGAAGTGGTGGAAGAGGGGGAAGGGAAGGGAGCAAGGTTATAGATGAGCCACATCGAAGT**TAG**
>*Oeu*010785.1
ATGGGAGCCATATGTGGCGTTGGTGTGGTCAAGGGATTTGGAAAGAACCTGTACATGACCGAAGGTGTTGGTGCT
AATGTTGTAGCCTACGGTTGCACCAAGGGTGATGCCCTAGGTGCTGAAATTGTTGGTACCTCTACACTCGTTTAC
ATTGTTTTCTCTACTACTGATGCCAAACATAGAGCCAGGGACTCACACGTTCCTGTATTAGTCGTGCCAAGAGTC
TTGGTGCAGCAAACATCTACATTCAAGAGCAGGCATGGGATGATCACAGACGATGACAGTGGAATGAAGAGAGGC
GGTTATAGAAGTGGTGGAAGAGGGGGAAGGGAAGGGAGCAAGGTTATAGATGAGCCACATCGAAGT**TAG**
>*Oeu*010785.1
MGAICGVGVVKGFGKNLYMTEGVGANVVAYGCTKGDALGAEIVGTSTLVYIVFSTTDAKHRARDSHVPVLVVPRV
LVQQTSTFKSRHGMITDDDSGMKRGGYRSGGRGGREGSKVIDEPHRS*

***Oeu*019177.1 *Oeu*PIP1-related**

>*Oeu*019177.1 | chr8:2118675..2120753 reverse
Incomplete sequence

>*Oeu*019177.1
ATGAATGTCAGAGGGCATTTGCTAGGTAACTCGAGCATCGCTTTAAAAGCAGTGTTTTAAAAGGCGTAATACATG
ACAATATGTAAAGGCCAAGCTATGGGTTAGATGAGGCAGAGGTGTTAAAAATCATAAAAAGACGTTTTTTTATTT
TAATATTATTTTAATTTTTAATCAATCGAAAAATCGTTAATAATTAAAAATAATACTCGCCAAACATCTAAATCA
AATTTATATATTTTTCCAAAGTTTTCAATAAAGGATAAGTTTCAAATGTAAATATTAAATAGTAAATAAATAGGA
ATTTTTGGGAGATTTTCTTTGTTCAAGGCTTCAAGCGGGTATTTTGAATTTAGAATCGACGAAGTGAATAAATCA
ACATTTAAATTTTAACGTAGAAGAATTGTAAAAGAAAAAAAAAAGATAATTATCCGCGCCCAAGCGAGCATTAGC
GGTCAATGATTATTATTGACCGCTATATAAGGCTTACGCCGCACATCACGCATTGGCAGCGCCTAAAAGCTGTGA
ATGCGCGCCTTTTAGAACAATGTTTAAACATGCGTTATCTTACAGGTCGGCTCCCAAATTAACATCATTTGGTTC
AATCTTTTCGATAATGAATACAGCTACTCAAACGGAAAAAACGGTTTAGTGATTGGCTGTTTTTTTTTCCATAAC
TATCCAATGGAAAAATGACTCGTGTCCCTCAATTTTAAATTCTGTAACTAAACTTGTTAATGTGGCAGGATTCTT
GGATAATTGCTTNNNNNNNNNNNNNNNNNNNNNNNNNNNNNNNNNNNNNNNNNNNNNNNNNNNNNNNNNNNNNNNNN
NNNNNNNNNNNNNNNNNNNNNNNNNNNNNNNNNNNNNNNNNNNNNNNNNNNNNNNNNNNNNNNNNNNNNNNNNNN
NNNNNNNNNNNNNNNNNNNNNNNNNNNNNNNNNNNNNNNNNNNNNNNNNNNNNNNNNNNNNNNNNNNNNNNNNNN
NNNNNNNNNNNNNNNNNNNNNNNNNNNNNNNNNNNNNNNNNNNNNNNNNNNNNNNNNNNNNNNNNNNNNNNNNNN
NNNNNNNNNNNNNNNNNNNNNNNNNNNNNNNNNNNNNNNNNNNNNNNNNNNNNNNNNNNNNNNNNNNNNNNNNNN
NNNNNNNNNNNNNNNNNNNNNNNNNNNNNNNNNNNNNNNNNNNNNNNNNNNNNNNNNNNNNNNNNNNNNNNNNNN
NNNNNNNNNNNNNNNNNNNNNNNNNNNNNNNNNNNNNNNNNNNNNNNNNNNNNNNNNNNNNNNNNNNNNNNNNNN
NNNNNNNNNNNNNNNNNNNNNNNNNNNNNNNNNNNNNNGATTTAACTATAATTTGTGAAGCTAGAATGCTTGGTT
TCTCTAATTTCATTCAGACATTGAGCAAATATGCTTATTTAGCTCCACTTTCTTCAAGTCATTCAATGCAATCTT
GACACGATACGAGGCTCATATGTTTAGTTACCGAGGTGCTGTTCAGACGAAAACTGACCTATACCGAATGTCACC
AGACATGGATGTATCAACTCACTTAGTTTTTATCAAAATTTTCCGTTTATGTTTGATGATGTACTGTTTGGCATT
TTTTTATGATTGATCAAACATACTTTTTATTCACATATTTTTGGGTGACCCACAAGTACTATTCGAGAACCTGTA
AAAAAGCTCGTGAGTCGTGACTGTTTATCATTTTGCTATGTGAAGAGACAATCCACGCAGTAGAGTCCTAAAATT
CTTAAATCTTGCAGATTTTGGCTCCTCTCCCCATTGGGTTTGCTGTATTTTTGGTTCATTTGGCCACCATCCCCA
TCACTGGAACTGGCATCAACCCGGCCAGGAGTCTTGGAGCTGCCATCATTTACAACAAAGAGCATGCATGGAACG
ACCACGTGAGAATCTCTCTAGTCTTAGATTCATTATAATTTATCGTAATTATGAATCAGTTGTAAATTTCAATTG
AAACTCATGTTTGTTTACATCGTTCAGTGGATCTTCTGGGTCGGACCCTTCATCGGAGCTGCTCTTGCTGCAATC
TACCACCAGATTGTCATCAGAGCCATTCCTTTCAAGAGCAGCAGGGCTTGT**TAA**
>*Oeu*019177.1
ATGAATGTCAGAGGGCATTTGCTAGACATTGAGCAAATATGCTTATTTAGCTCCACTTTCTTCAAGTCATTCAAT
GCAATCTTGACACGATACGAGGCTCATATGTTTAGTTACCGAGGTGCTGTTCAGACGAAAACTGACCTATACCGA
ATGTCACCAGACATGGATATTTTGGCTCCTCTCCCCATTGGGTTTGCTGTATTTTTGGTTCATTTGGCCACCATC
CCCATCACTGGAACTGGCATCAACCCGGCCAGGAGTCTTGGAGCTGCCATCATTTACAACAAAGAGCATGCATGG
AACGACCACTGGATCTTCTGGGTCGGACCCTTCATCGGAGCTGCTCTTGCTGCAATCTACCACCAGATTGTCATC
AGAGCCATTCCTTTCAAGAGCAGCAGGGCTTGT**TAA**
>*Oeu*019177.1
MNVRGHLLDIEQICLFSSTFFKSFNAILTRYEAHMFSYRGAVQTKTDLYRMSPDMDILAPLPIGFAVFLVHLATI
PITGTGINPARSLGAAIIYNKEHAWNDHWIFWVGPFIGAALAAIYHQIVIRAIPFKSSRAC*

***Oeu*018831.2 *Oeu*PIP1-related**

>*Oeu*018831.2 | chr4:15354575..15359491 forward
Pseudogene

>*Oeu*018831.2
ATGGGAGCCATATGTGGTGTTGGTGTGGTTAAGGGATTCGGAAAGACCCTGTACATGACCAAGGGTGGTGGTGGT
ACTAATGTAGTAGCCGACGGTTACACCAAGGGTGATGCCCTAGGTGCTGAAATTGTTGGTACCTCTGTGCCCTTT
AAAAAACTTCTTGGGTCTGAGGGAGGGACTACAACCCAACCCAATCCCCTCCCAAATCCGTCCCTAGTCTTGTGC
TCGAGAAAAGCATTGGAAAGAACATGGTGCTCTTTATGGCAATGGTGCTAGAGTAGGCTGCGAACGAGCGGAGGT
TGAAAAATGAAAATCGAATTGGAAATTTCAAATTGATTGAATAATTTCAACTGAAATGGCAGAATTGGTTGTATT
TTGTATTTATAAATGGGTCATGAGTTTTGGGTAATCATGTTGGGCTAATGGGTTAATGGGCTAGTGGGTTTCGCT
TGGTAGTTGGGCTGGAGCTTGGAGGGAATTAAAAGATAAAAAACATTTGGGCCGTAGGGTTGGGCCCAAAAGTTT
AGTGCGGGCATGGACTCCATTGGGCTCTCCCCAGGTGGCGACGTTGGTCACGATGGTAAGTGGCAAAAGAATCGA
GGACTGCTAAACTTCTTTGATGAGGAAGTACAGATAGACAGTGATGAAGAAGAGGAAGGCGAAAATGGTTAGGTT
TTTTTCTTCTTTTTTTCTTCTTTTTTTTTTTTTTGCGGTCTTATTTTATGTTATCAATTACTTAGTTTATTGAT
TTTGAGTTTTATAATCTGTTGAAAATTTTCGTTTGAGAAGAAAGTTCTGTGATTTAGTGAAAATTAAAGTGAATG

AGGCAAACCTATAATTTTTTTATTATCAATTTGACAAATGAAACAAAAAAAGAAAGTTGAAATTTCTTACTTTAA
AGGAAAAGCAATTATGACAAGTTTCAAGTTATTATAGTTGTTGTGGATAAAACTTGGGGGAAATTTTGCTCTGTG
AATATGTCAAGTTCTTGTGAGTACATCGAAATTACCAGAGATTGGAGACTAGGAGTACTCCTAAAAATTTTGGCA
GCTGCAGCTGAAGAGAAAGAAATACATTCATTTGAAATTGACCCTGCACAAGTAGGTTAATTAGTTCTGTCAAAA
TAATCTCCTATTTTCTTCATTTTTAATGTAGACAGCAGTTGTGATGATGATCTTTCATTTCATTTAATCTGGACT
TTACTATGACTGGGCTTCAACATATATGTGTAATTGTTATTCCTTCAATGGACAATTCTAAGTGTACATAATTTG
GTTTACCAAATCATTTACCTACTCTCACAAGCACGTGGACCCCGCGTGAATAAAATAATTCCAACATGTGGTCCC
CACACCCCTATTGTAAGTGTATGTAAAACATTATGTAAAGAAAATTAGGTACATCTAGCATGGTCTTCCTTCAAT
GTGTGTATAGTATTCTGTGCATTGTCTTGGAAGCTAGGTCTAAAATATGTATATCCAATAGGTTTCTTTGGTTTG
GATATATTTCTTCACTAAAGAATATTTAATGTCGTTACTTTTCAAGAAATAATTGGAACCATCTTCCTTTATGAC
ACATCACGTGTCGAATTAACCCATTTGTACCATTTAAGACCAATAATTAACCCCCCAACCCCCCACCACCACAAT
ATACATACACGTCATTTCTTAACTAATCATTTGACCAGCTAGCTTATTGATGTCATGTATATGCATATGTATAAA
TGCTATAGCTGGTCAGTATGAAACTCGATTGTGTACTGTTTTTTATATGGAAGATCAAGGCTTTCTGTCAGAATT
TTGATAGCAGTGATTCTTCTTGTTTCTTTGGCAAATTTCTTAAAATCTTTCTGAATGTTGGTAGTGTATACTGTT
TTTACAATTTCAGAAACTAAATTCTTCTTGGGTTGTTTGCTAAATTAATAATATATAATACAGCAAGCCAAAAAT
GAGAAATAATTGCTTGTTCTCTAAGTAACTTTCACAATATTGCCTAACTGATTAGCTGATCATATTCTATTAACT
ATTTATGTGTTTACAGGTTGAGAATGTTAAGCAACATTGTTTCCCTAATGTTCTGAATTATTCCATGCTGGAAGA
GAATCACTTCAGTAACGACACTGTGAATTGCTTTTGATTTCTTTTTATCATTAGGGCTAGAGATACCTATTATTT
CACACAATTATAAAATTCTATAGTCCTATGGGAGCCATATGTGGTGTTGGTGTGGTTAAGGGATTCGGAAAGACC
CTGTACATGACCAAGGGTGGTGGTGGTACTAATGTAGTAGCCGACGGTTACACCAAGGGTGATGCCCTAGGTGCT
GAAATTGTTGGTACCTCTGTGCTCGTTTACACGGTTTTCTCTGCTTCTGATGCCAAACATAGAGCCAGAGACACA
CACATTCCTGTAAGTACTACTCTTGTCTTTGTAGGCCTATTTCTCTTGACATTATTAATTAGACTTAAGCCAATA
TAAATCATTCTACATAATAGGTATGATTAAATGTGATTAAGTTTGAAAAAGAATTGTCTTCTATATCTGTAAGAA
TTGGTAAATCAAAACCTGATATTATTGATCATGAAAAAGTTTGATTAATTGGATTTCGTAATGCTATAATTCAAT
GGTTTTCAAATATTGGCCCCCTTGCCCATTAGGTTTGCTATGTTCTTGGTGCACTTGGCTACCATCCCTATTATC
GATACAAGTATTAACCGTGCCAGAAGTCTTGGTGCAGCAAACATCTACAATAAAGAGCAGGCATGGGATGATCAT
GTAAGCTAAAAATTGAACTATATCTAAATACAATATAATCAGTATTCTACTATTGAAATATTTGTGATAAGGAAC
CACTACATGTCGTGTTAACTTTTACATGTGTAAAGGTTCGCATGGTAAAACAGAATAAATTTTTATGTTTTTCAC
TTACCACAGCCCTCTTTTATTTATTTTCATACAACTATGAATTTTTTTAAAAAAATTGACAAATGGGATGATAAG
ATTGGCTAAAGTTTGTTCTTGCTCTCATCTTGCTCTGATAATATGCAATGGATATTCTGGGTTGTACCATATGGT
TTTGGTGGAAGAGGCGACAGGGGGAAATTTTGCTCTGTGAATATGTCAAGTTCTTGTGAGTACATCGAAATTACC
AGAGATTGGAGACTAGGAGTACTCCTAAAAATTTTGGCAGCTGCAGCTGAAGAGAAAGAAATACATTCATTTGAA
ATTGACCCTGCACAAGTAGGTTAATTAGTTCTGTCAAAATAATCTCCTATTTTCTTCATTTTTAATGTAGACAGC
AGTTGTGATGATGATCTTTCATTTCATTTAATCTGGACTTTACTATGACTGGGCTTCAACATATATGTGTAATTG
TTATTCCTTCAATGGACAATTCTAAGTGTACATAATTTGGTTTACCAAATCATTTACCTACTCTCACAAGCACGT
GGACCCCGCGTGAATAAAATAATTCCAACATGTGGTCCCCACACCCCTATTGTAAGTGTATGTAAAACATTATGT
AAAGAAAATTAGGTACATCTAGCATGGTCTTCCTTCAATGTGTGTATAGTATTCTGTGCATTGTCTTGGAAGCTA
GGTCTAAAATATGTATATCCAATAGGTTTCTTTGGTTTGGATATATTTCTTCACTAAAGAATATTTAATGTCGTT
ACTTTTCAAGAAATAATTGGAACCATCTTCCTTTATGACACATCACGTGTCGAATTAACCCATTTGTACCATTTA
AGACCAATAATTAACCCCCCAACCCCCCACCACCACAATATACATACACGTCATTTCTTAACTAATCATTTGACC
AGCTAGCTTATTGATGTCATGTATATGCATATGTATAAATGCTATAGCTGGTCAGTATGAAACTCGATTGTGTAC
TGTTTTTTATATGGAAGATCAAGGCTTTCTGTCAGAATTTTGATAGCAGTGATTCTTCTTGTTTCTTTGGCAAAT
TTCTTAAAATCTTTCTGAATGTTGGTAGTGTATACTGTTTTTACAATTTCAGAAACTAAATTCTTCTTGGGTTGT
TTGCTAAATTAATAATATATAATACAGCAAGCCAAAAATGAGAAATAATTGCTTGTTCTCTAAGTAACTTTCACA
ATATTGCCTAACTGATTAGCTGATCATATTCTATTAACTATTTATGTGTTTACAGGTTGAGAATGTTAAGCAACA
TTGTTTCCCTAATGTTCTGAATTATTCCATGCTGGAAGAGAATCACTTCAGTAACGACACTGTGAATTGCTTTTG
ATTTCTTTTTATCATTAGGGCTAGAGATACCTATTGTTTCACACAATTATAAAATTCTATAGTCCAATGGGAGCC
ATATGTGGTGTTGGTGTGGTTAAGGGATTCGGAAAGACTCTGTACATGAACTAGGGTGGTGGTGGTACTAATGTA
GTAGCCGACGGTTACACCAAGGGTGATGCCCTAGGTGCTGAAATTGTTGGTACCTCTGTGCTCGTTTACACGGTT
TTCTCTGCTTCTGATGCCAAACATAGAGCCAGAGACACACACATTCCTGTAAGTACTACTCGTCTTTGTAGGCCT
ATTTCTCTTGACATTATTAATTAGACTTAAGCCAATATAAATCATTCTACATAATAGGTATGATTAAATGTGATT
AAGTTTGAACAAGAATTGTCTTCTGTATCTGTAAGAATTGATAAAAACCTGATATTATTGATCATGAAAAAGTTT
GATTAATTGGATTTCGTAATACTATAATTCAATGGTTTTCAAATATTGGTCCCCTTGCCCATTAGGTTTGCTGTG
TTCTTGGCGCACTTGGCTACCATCCCTATTATCGATACAGGTATTAACCGTGCCAGAAGTCTTGGTGCAGCAAAC
ATCTACAATAAAGAGCAGGCATGGGATGATCATGTAAGC**TAA**

>*Oeu*018831.2

ATGGGAGCCATATGTGGTGTTGGTGTGGTTAAGGGATTCGGAAAGACCCTGTACATGACCAAGGGTGGTGGTGGT
ACTAATGTAGTAGCCGACGGTTACACCAAGGGTGATGCCCTAGGTGCTGAAATTGTTGGTGGCGACGTTGGTCAC
GATGGTAAGTGGCAAAAGAATCGAGGACTGCTAAACTTCTTTGATGAGGAAGTACAGATAGACAGTGATGAAGAA
GAGGAAGGCGAAAATGTTGTTGTGGATAAAACTTGGGGGAAATTTTGCTCTGTGAATATGTCAAGTTCTTGTGAG
TACATCGAAATTACCAGAGATTGGAGACTAGGAGTACTCCTAAAAATTTTGGCAGCTGCAGCTGAAGAGAAAGAA
ATACATTCATTTGAAATTGACCCTGCACAAGTTGAGAATGTTAAGCAACATTGTTTCCCTAATGTTCTGAATTAT
TCCATGCTGGAAGAGAATCACTTCAGTAACGACACTGGATTCGGAAAGACCCTGTACATGACCAAGGGTGGTGGT

GGTACTAATGTAGTAGCCGACGGTTACACCAAGGGTGATGCCCTAGGTGCTGAAATTGTTGGTACCTCTGTGCTC
GTTTACACGGTTTTCTCTGCTTCTGATGCCAAACATAGAGCCAGAGACACACACATTCCTAATTGGTTTGCTATG
TTCTTGGTGCACTTGGCTACCATCCCTATTATCGATACAAGTATTAACCGTGCCAGAAGTCTTGGTGCAGCAAAC
ATCTACAATAAAGAGCAGGCATGGGATGATCATGTTGAGAATGTTAAGCAACATTGTTTCCCTAATGTTCTGAAT
TATTCCATGCTGGAAGAGAATCACTTCAGTAACGACACTGGTGGTGGTGGTACTAATGTAGTAGCCGACGGTTAC
ACCAAGGGTGATGCCCTAGGTGCTGAAATTGTTGGTACCTCTGTGCTCGTTTACACGGTTTTCTCTGCTTCTGAT
GCCAAACATAGAGCCAGAGACACACACATTCCTATATTGGTCCCCTTGCCCATTAGGTTTGCTGTGTTCTTGGCG
CACTTGGCTACCATCCCTATTATCGATACAGGTATTAACCGTGCCAGAAGTCTTGGTGCAGCAAACATCTACAAT
AAAGAGCAGGCATGGGATGATCATGTAAGC**TAA**

>*Oeu*018831.2

MGAICGVGVVKGFGKTLYMTKGGGGTNVVADGYTKGDALGAEIVGGDVGHDGKWQKNRGLLNFFDEEVQIDSDEE
EEGENVVVDKTWGKFCSVNMSSSCEYIEITRDWRLGVLLKILAAAAEEKEIHSFEIDPAQVENVKQHCFPNVLNY
SMLEENHFSNDTGFGKTLYMTKGGGGTNVVADGYTKGDALGAEIVGTSVLVYTVFSASDAKHRARDTHIPNWFAM
FLVHLATIPIIDTSINRARSLGAANIYNKEQAWDDHVENVKQHCFPNVLNYSMLEENHFSNDTGGGGTNVVADGY
TKGDALGAEIVGTSVLVYTVFSASDAKHRARDTHIPILVPLPIRFAVFLAHLATIPIIDTGINRARSLGAANIYN
KEQAWDDHVS*

PIP2 – Plasmalemic Intrinsic Protein type 2

*Oeu*PIP2;1 *Oeu*001271.1

>*Oeu*PIP2;1_*Oeu*001271.1 | scaffold1031:177815..179775 reverse
ATGACGAAAGACGTCGAATCTCATGTGGAGCCAGCGGAGGAATACTCCGCCAAGGACTACCATGATCCGCCACCG
GCGCCTCTCATTGATTTTGATGAGCTGAGCAGCTGGTCGTTTTACAGAGCACTTATTGCTGAGTTCGTTGCCACT
TTGCTGTTCCTTTACGTCACAGTTTTGACCGTTATAGGCTACAAAAGCCAGATTGACCCAGCTCACGGCGGCGAC
GACTGCGGCGGCGTTGGGATTCTTGGCATTGCATGGGCTTTCGGCGGCATGATTTTCATTCTTGTCTACTGCACC
GCCGGTATCTCTGGTCAGTTTTACAGGTTTCAGCCAGCTTTTCCTTGATTGGTACCTGCACCATAATAAGTAAAT
TACTTGTGAAAATAAGTAACTTTTTCATATTGTGACAGGAGGACATATTAACCCAGCAGTGACATTCGGGCTGTT
CTTGGGAAGAAAGGTGTCACTGATCAGAGCAGTGCTTTACATGGTAGCACAATGTTTGGGTGCAATCTGTGGTGT
GGGATTGGTGAAAGCATTCCAAAAATCGTACTATCATAAGTATGGTGGTGGAGCAAATGAACTTGCTCCAGGGTA
CAACAAAGGGGTTGGTTTGGGTGCTGAAATTATTGGCACCTTTGTTTTGGTCTACACCGTTTTCTCAGCCACTGA
TCCTAAGAGGAATGCCAGAGACTCCCATGTCCCTGTAAGCACTTTTTTTCTCTGCTAACGCACCCACACCCCCAA
GTCTTATTATCCCAAGGGAGGAGTTCTCCTCGGAATAACAACCCCTTGATTGGATGTTTTAAATTTGTTTTTTGG
GCAAAATCCAATGAAGTTTTGCCATTTGACAACCTGTAACTCCTCTGGCAAATTTGTTGTACATATAACATGGTT
CATTCTCCACAGAATCATTTTTTTGCCACTAATCTGTCGTACTAAAGGTGTCATAAAATCGCGTGGGGTTCATCA
GGGTCCTAAATTAATTTGTACACGGGTCAACGGGCAATAATAAGTTCTTGACCCAAAGTCCATCAATGAACTTGA
ATTTATAGAAAATTTTGGATTATATCCTTGCAAATATTGATTTAATCTGATACATATGAAGGTCTTGGCTCCACT
TCCCATCGGATTTGCTGTGTTCATGGTTCACCTTGCCACCATCCCCATCACCGGAACTGGCATCAACCCTGCTAG
GAGCTTTGGAGCTGCTGTCATCTACAACGAAGACAAGCCCTGGGATGACCATGTATGCCTCCCTTTTACCTCTTT
TTTACTGATTTTACACGCACAATAAGTTGNNNNNNNNNNNNNNNNNNNNNNNNNNNNNNNNNNNNNNNNNNNNNN
NNNACCACCCCCCCCCCCCCCCCCCCTTTTTTTAAATTGTAATTATTTTTATAGAATTTAATGACATAATTCAT
AAAAGCTAGTCAAGTCCCCAATTTATCTTTTTAGTTTTTGGACAAGGGTATCTCAATTTGATACAAAAACAAGTA
GGAGGTCCCGAGTTCAAGTTGCACTTCCACTCATTTATAAAAATATGCACGTCTCATGTCTGCGCATGAGGACAT
GTGTTCAAGACGTAATTTAACACATTCCTCTCCCGTTAACACATTAAGCCCCTGTTTGATAGGGATTAAAATCAG
ATTTATAATAGATTTTGATAAAAGTTGATGAAATGTGTACTTGAAAAGTTGTGGGTCCCCTAAAATATGTTGTTA
GAATTAATTATTAAATTTGACTTAGATTAAAATCTGAATTTTAATTTTTACCAAACATGGACTAAGCTTTTAAAC
GAGTTATTAAAAACGAAGATAGTACTAATATATTTGTTAATATTCAGTGGATTTTTTGGGTTGGACCATTCGTTG
GAGCTCTAATTGCTGCAATCTACCACCAATACATCCTAAGGGCAGGAGCCATCAAGGCACTGGGTTCTTTCAGGA
GCAACGCT**TAA**
>*Oeu*PIP2;1_*Oeu*001271.1
ATGACGAAAGACGTCGAATCTCATGTGGAGCCAGCGGAGGAATACTCCGCCAAGGACTACCATGATCCGCCACCG
GCGCCTCTCATTGATTTTGATGAGCTGAGCAGCTGGTCGTTTTACAGAGCACTTATTGCTGAGTTCGTTGCCACT
TTGCTGTTCCTTTACGTCACAGTTTTGACCGTTATAGGCTACAAAAGCCAGATTGACCCAGCTCACGGCGGCGAC
GACTGCGGCGGCGTTGGGATTCTTGGCATTGCATGGGCTTTCGGCGGCATGATTTTCATTCTTGTCTACTGCACC
GCCGGTATCTCTGGAGGACATATTAACCCAGCAGTGACATTCGGGCTGTTCTTGGGAAGAAAGGTGTCACTGATC
AGAGCAGTGCTTTACATGGTAGCACAATGTTTGGGTGCAATCTGTGGTGTGGGATTGGTGAAAGCATTCCAAAAA
TCGTACTATCATAAGTATGGTGGTGGAGCAAATGAACTTGCTCCAGGGTACAACAAAGGGGTTGGTTTGGGTGCT
GAAATTATTGGCACCTTTGTTTTGGTCTACACCGTTTTCTCAGCCACTGATCCTAAGAGGAATGCCAGAGACTCC
CATGTCCCTGTCTTGGCTCCACTTCCCATCGGATTTGCTGTGTTCATGGTTCACCTTGCCACCATCCCCATCACC
GGAACTGGCATCAACCCTGCTAGGAGCTTTGGAGCTGCTGTCATCTACAACGAAGACAAGCCCTGGGATGACCAT
TGGATTTTTTGGGTTGGACCATTCGTTGGAGCTCTAATTGCTGCAATCTACCACCAATACATCCTAAGGGCAGGA
GCCATCAAGGCACTGGGTTCTTTCAGGAGCAACGCT**TAA**
>*Oeu*PIP2;1_*Oeu*001271.1
MTKDVESHVEPAEEYSAKDYHDPPPAPLIDFDELSSWSFYRALIAEFVATLLFLYVTVLTVIGYKSQIDPAHGGD
DCGGVGILGIAWAFGGMIFILVYCTAGISGGHINPAVTFGLFLGRKVSLIRAVLYMVAQCLGAICGVGLVKAFQK
SYYHKYGGGANELAPGYNKGVGLGAEIIGTFVLVYTVFSATDPKRNARDSHVPVLAPLPIGFAVFMVHLATIPIT
GTGINPARSFGAAVIYNEDKPWDDHWIFWVGPFVGALIAAIYHQYILRAGAIKALGSFRSNA*

*Oeu*PIP2;2 *Oeu*030183.1

>*Oeu*PIP2;2_*Oeu*030183.1 | chr4:8070191..8071649 reverse
ATGACGAAAGACGTTGAATCTCACGGGGAGCCGGCGGAATTCTCGGCCAAGGACTACCATGATCCACCACCGGCG
CCGCTCATTGATTTTGAAGAGCTGAGCAGCTGGTCATTTTACAGAGCACTTATTGCTGAGTTCATTGCCACTTTA
CTGTTCCTCTACGTCACAGTTTTGACTGTTATTGGATACAAAAGCCAGATTGACCCCGCTCACGGCGGCGATCAG
TGCGGCGGCGTCGGGATTCTAGGCATTGCATGGGCTTTCGGTGGCATGATTTTCATTCTTGTTTACTGCACCGCC
GGTATCTCTGGTCAGTTTTAAAAGTCTTTACAAGCTTTTTTCCTTATAGATTTTGATGAGTGACTTACTGGTGAA
ATAAGTAACTTTTTGATAATGTGACAGGAGGACACATTAACCCGGCTGTGACATTCGGGCTGTTCTTGGGACGAA
AGGTGTCACTGATCAGAGCAGTGATGTACATGGTAGCACAGTGTTTGGGTGCAATCTGTGGTGTGGGTTTGGTGA

AAGCATTCCAAAAATCTTATTACAACCTGTACGGTGGTGGAGCAAATGAGCTTGCTCCAGGTTACAACAAAGGTG
TTGGATTGGGTGCTGAAATTATTGGCACATTTGTTTTGGTTTACACTGTCTTCTCAGCCACTGATCCTAAGAGGA
ATGCCAGAGATTCCCATGTTCCCGTAAGCAATTTTTTTTCTCTTCTAACCCAAACTCTGGTAAAATTTTTGGTTA
TAGTCTTGAAAATATTGATTTAAATCTTACATATATGAAGGTGTTGGCTCCACTTCCCATTGGATTTGCTGTTTT
CATGGTTCACCTGGCCACCATCCCCATCACTGGAACTGGCATCAACCCTGCTAGGAGCTTTGGAGCTGCTGTCAT
CTACAACAAAGACAAGCCCTGGGATGACCATGTATGCTCTTTTTTACTTCTTTAGGCCCTGTTTTGACGGTCTTT
TTTTTTTTTTTTTGGAAAGAATGTATTTTTGAATAGTAACTTTTTGGAGAATGCGTGATTAAAAAGTAAAAAATG
TCTTGGGTTTATATGAATAATATTTTATTGTGAGAATGTATGCTCGAAAATGAATATGGGAAAGTAGAGTAATG
AAAGAATTACCGAAGAGGGGCTTATATAAATAATTATTTCATGTCTTGTTTTCACTTGTTTATGAAGGGTAGATG
TGTGGAATATTTGATAAAAAAGATAAACGAGGTTGTGTTGAATACATAATTTAAAGAAGTTTATCTGTTGTTTAA
CAACTTGAACTTTTGGATAAGGTTTTTGAGACTAACATATGTTATTACTTTTGTTTAAATAAATATGCAGTGGAT
TTTCTGGGTTGGGCCATTTATTGGAGCTGCCATTGCTGCATTCTACCACCAATACATTCTAAGGGCAGCAGCCAT
TAAGGCACTGGGATCTTTCAGGAGCAATAAT**TAA**
>*Oeu*PIP2;2_*Oeu*030183.1
ATGACGAAAGACGTTGAATCTCACGGGGAGCCGGCGGAATTCTCGGCCAAGGACTACCATGATCCACCACCGGCG
CCGCTCATTGATTTTGAAGAGCTGAGCAGCTGGTCATTTTACAGAGCACTTATTGCTGAGTTCATTGCCACTTTA
CTGTTCCTCTACGTCACAGTTTTGACTGTTATTGGATACAAAAGCCAGATTGACCCCGCTCACGGCGGCGATCAG
TGCGGCGGCGTCGGGATTCTAGGCATTGCATGGGCTTTCGGTGGCATGATTTTCATTCTTGTTTACTGCACCGCC
GGTATCTCTGGAGGACACATTAACCCGGCTGTGACATTCGGGCTGTTCTTGGGACGAAAGGTGTCACTGATCAGA
GCAGTGATGTACATGGTAGCACAGTGTTTGGGTGCAATCTGTGGTGTGGGTTTGGTGAAAGCATTCCAAAAATCT
TATTACAACCTGTACGGTGGTGGAGCAAATGAGCTTGCTCCAGGTTACAACAAAGGTGTTGGATTGGGTGCTGAA
ATTATTGGCACATTTGTTTTGGTTTACACTGTCTTCTCAGCCACTGATCCTAAGAGGAATGCCAGAGATTCCCAT
GTTCCCGTGTTGGCTCCACTTCCCATTGGATTTGCTGTTTTCATGGTTCACCTGGCCACCATCCCCATCACTGGA
ACTGGCATCAACCCTGCTAGGAGCTTTGGAGCTGCTGTCATCTACAACAAAGACAAGCCCTGGGATGACCATTGG
ATTTTCTGGGTTGGGCCATTTATTGGAGCTGCCATTGCTGCATTCTACCACCAATACATTCTAAGGGCAGCAGCC
ATTAAGGCACTGGGATCTTTCAGGAGCAATAAT**TAA**
>*Oeu*PIP2;2_*Oeu*030183.1
MTKDVESHGEPAEFSAKDYHDPPPAPLIDFEELSSWSFYRALIAEFIATLLFLYVTVLTVIGYKSQIDPAHGGDQ
CGGVGILGIAWAFGGMIFILVYCTAGISGGHINPAVTFGLFLGRKVSLIRAVMYMVAQCLGAICGVGLVKAFQKS
YYNLYGGGANELAPGYNKGVGLGAEIIGTFVLVYTVFSATDPKRNARDSHVPVLAPLPIGFAVFMVHLATIPITG
TGINPARSFGAAVIYNKDKPWDDHWIFWVGPFIGAAIAAFYHQYILRAAAIKALGSFRSNN*

*Oeu*PIP2;3 *Oeu*033842.1

>*Oeu*PIP2;3_*Oeu*033842.1 | chr12:7848155..7849789 reverse
ATGGTAAAAGATGGTGGAGTGGCTGAGCATGGATCATTCACAGTCAAAGACTACCACGATCCACCACCGGCGCCG
TTGATTGATGCGGCGGAGCTTACAAAGTGGTCATTTTACAGAGCTATTATAGCTGAGTTTATAGCCACCTTGCTT
TTCCTTTACATTACGGTTTTGACTGTGATAGGGTACAAGAGCCAGACTGATACCAACGCCGGCCACGGCGGTGAT
CAGTGCGGTGGCGTTGGAGTTCTTGGTATTGCATGGGCCTTTGGTGGCATGATCTTCGTTCTTGTCTACTGCACT
GCTGGAATTTCCGGTGAGCTTTGTTTTATATAAAATTATGGATCAGAAAACAAGTTTATATATATATTGATTAAA
TGGTTTATCATCATGTGCAGGGGGGCACATTAATCCAGCAGTGACATTCGGGCTTTTCTTGGCCCGGAAGGTGTC
GATGATCAGAGCCATTATGTATATGGTGGCTCAGTGTTTGGGGGCCATTTGTGGCGTCGGACTGGTGAAGGCATT
CCAGAAAGCATATTACAAGAAGTACGGCGGCGGCGCCAATGAACTCGCAGACGGCTACAGCACCGGCGTGGGATT
GTCGGCTGAGATCATCGGAACATTCGTTCTTGTTTACACAGTTTTCTCAGCCACCGATCCCAAGAGAAGTGCCAG
GGATTCCCATGTTCCTGTGAGTTGCACATATACAATTCCCCATCTTTTTTTCTTCTGAGAAGTGCATGTTTGGTT
AAGTTATAAGGCTTACTTATAAGGAATAAGTATTTAGAAAAATTGGATCCATGAAATAACTTAAATATTTCGAAA
ACAAAGAAAGCACCTACTAGTGATAAGTACAAGTTAAAATTACTTTAAACTTGACTTTGTTCGACTTCAAAATTG
ATCCTAAACTCTTTACTTGAGTTTGAGCCCGGCTAGAATTCAGTGGTTTTATCTTTATGAAACGATTCGATATTG
ACACTACTTGAATCTGGACTTGAATCTCAGGTTTTGGCACCTCTCCCAATTGGATTTGCAGTTTTCATGGTTCAC
TTGGCAACCATCCCAATCACTGGTACTGGCATCAACCCTGCTAGAAGTTTTGGAGCTGCAGTAATCTACAACCAG
GAAAAGGCTTGGGATGACCAGGTTAGCATTTTTGGAAAGTAATTATGAATTAGAGTTGACTATATGAATCGTGTT
TTGCACCATTTAATTCACTTTGCTGCTAATTATACTCGAAGAGCCCTCATGCAATGATTAGTAGGATTTGTGTCA
TAAGATGTGATTAAATTTCAAGTTCAATGTCAATATTCTACCTCAACCCTTCGGGTTTTTATCTAAACATGTGAC
AAGATCCCTTTTTTTTTTCTTTTTTCTTTTTTCTTTTTTTTTTTCTTTTTTTAAGTCTAAACATGAGACGAGCTA
CGAAAAAACATGAGTTACATCCCTGCATTTACATTGAAAAAACACCTACAATCACAAGTAATAATTTATGTATAC
TTTTGTCTCCTTCAGTGGATCTTCTGGGTAGGACCCTTCATTGGTGCAGCCATTGCAGCCTTCTACCACCAGTTT
ATTTTGAGAGCGGGAGCAGCTAAAGCTCTTGGTTCATTCAGAAGCAGCTACCATGTT**TGA**
>*Oeu*PIP2;3_*Oeu*033842.1
ATGGTAAAAGATGGTGGAGTGGCTGAGCATGGATCATTCACAGTCAAAGACTACCACGATCCACCACCGGCGCCG
TTGATTGATGCGGCGGAGCTTACAAAGTGGTCATTTTACAGAGCTATTATAGCTGAGTTTATAGCCACCTTGCTT
TTCCTTTACATTACGGTTTTGACTGTGATAGGGTACAAGAGCCAGACTGATACCAACGCCGGCCACGGCGGTGAT
CAGTGCGGTGGCGTTGGAGTTCTTGGTATTGCATGGGCCTTTGGTGGCATGATCTTCGTTCTTGTCTACTGCACT

GCTGGAATTTCCGGGGGGCACATTAATCCAGCAGTGACATTCGGGCTTTTCTTGGCCCGGAAGGTGTCGATGATC
AGAGCCATTATGTATATGGTGGCTCAGTGTTTGGGGGCCATTTGTGGCGTCGGACTGGTGAAGGCATTCCAGAAA
GCATATTACAAGAAGTACGGCGGCGGCGCCAATGAACTCGCAGACGGCTACAGCACCGGCGTGGGATTGTCGGCT
GAGATCATCGGAACATTCGTTCTTGTTTACACAGTTTTCTCAGCCACCGATCCCAAGAGAAGTGCCAGGGATTCC
CATGTTCCTGTTTTGGCACCTCTCCCAATTGGATTTGCAGTTTTCATGGTTCACTTGGCAACCATCCCAATCACT
GGTACTGGCATCAACCCTGCTAGAAGTTTTGGAGCTGCAGTAATCTACAACCAGGAAAAGGCTTGGGATGACCAG
TGGATCTTCTGGGTAGGACCCTTCATTGGTGCAGCCATTGCAGCCTTCTACCACCAGTTTATTTTGAGAGCGGGA
GCAGCTAAAGCTCTTGGTTCATTCAGAAGCAGCTACCATGTT**TGA**
>*Oeu*PIP2;3_*Oeu*033842.1
MVKDGGVAEHGSFTVKDYHDPPPAPLIDAAELTKWSFYRAIIAEFIATLLFLYITVLTVIGYKSQTDTNAGHGGD
QCGGVGVLGIAWAFGGMIFVLVYCTAGISGGHINPAVTFGLFLARKVSMIRAIMYMVAQCLGAICGVGLVKAFQK
AYYKKYGGGANELADGYSTGVGLSAEIIGTFVLVYTVFSATDPKRSARDSHVPVLAPLPIGFAVFMVHLATIPIT
GTGINPARSFGAAVIYNQEKAWDDQWIFWVGPFIGAAIAAFYHQFILRAGAAKALGSFRSSYHV*

*Oeu*PIP2;4 *Oeu*030439.1

>*Oeu*PIP2;4_*Oeu*030439.1 | scaffold2530:66057..67614 forward
ATGGCAAAGGACGCTGGAGTGGCGGAGCATGGATCGTTCGCGGCAAAGGACTACCAAGATCCACCACCAGCTCCG
CTGATTGACGCGGAGGAGCTTACAAAGTGGTCATTTTACAGAGCTGTTATAGCCGAGTTTATAGCCACCTTGCTC
TTCCTTTACATTACGGTTTTGACTGTGATTGGGTACAAGAGCCAGATTGATACCACGGTTGGCCACGGCGGTGAT
CAGTGCGGTGGCGTTGGAATTCTTGGTATTGCATGGGCCTTTGGTGGAATGATCTTCGTTCTTGTCTACTGCACC
GCTGGAATTTCCGGTGAGTTTTGTTTATACGCACACAGATACATGCATACATGTATGGTTTGATTTTAATGGCAT
TTCTCATGTCATGTCATGTGCATCTCAGGGGGACACATTAATCCAGCAGTGACATTCGGGCTTTTCTTGGCCCGA
AAGGTGTCAATGATCAGAGCCATTATGTATATGGTGGCTCAGTGTTTGGGGGCCATTTGTGGCGTCGCGCTGGTG
AAGGCGTTACAGAAGTCACACTACAAGAAATATGGCGGCGGCGCAAATGAGCTCGCCGATGGCTACAGCACCGGC
GTCGGATTATCCGCGGAGATAATCGGAACATTCGTCCTTGTTTACACAGTTTTCTCTGCCACTGATCCCAAGAGA
AGTGCCAGGGATTCCCATGTCCCTGTAAGTTGACATATACAATTCTCTATTTTTTTTCTTTTGAGAAGTGCATGT
TTGGTTAACCTTAAAAAATTTACTTATAATTGAATAAGTATTAAAATAATTTTTTTGGGGGTCCAAGTAGAAAAT
AACTTATTTAGTAGCTTTACTGACAATATTACTTAAAAGTAATTTAATATTGACAATTTTTGCATTTGGACTTGA
ATCTTAGGTATTGGCACCTCTTCCAATTGGATTTGCAGTTTTCATGGTTCATTTGGCCACCATCCCCATCACTGG
TACTGGTATCAACCCTGCTAGGAGTTTAGGGGCTGCAGTGATCTACAACCAGGAAAAAGCTTGGGATGATCAGGT
TAGCATTTTTCCCAAAGTATTACAAATTAGATTTAACTATACTCATAATGTTTTGCGCCATTTCATTCACTTTAT
TTATTGTGAATTCTTCTCGAAGATCCCCGATAAATTTCAAGTTCATTGTCAACATACCAGCTCTCGTGTTTTTAT
GAATTTTTTCGATTATGACAGCCTAGTCAAAAAGTATCGTCGAAATCATGTTGAAGAAGTGAGAAAATTTTATAC
TTCGCTGTCAACCCCTTTCTTTATTCGAACTTGAGACTGACTATAAACAAAAATAGCAAAAAGTTATATTTCAAA
AGCTCTAAACATATAGTTTTCAATATTATTTATACCCCCACCCAAAATCACAAGTAGTAATTTATCTATTTACTT
TCGTGTCCTTCAGTGGATCTTCTGGGTTGGACCCTTCTTTGGTGCAGCCATTGCAGCCTTCTACCACCAGTTTAT
TTTGAGAGCAGGAGCAGCTAAAGCCCTTGGTTCATTCAGAAGCAGCTCCCATGTT**TAA**
>*Oeu*PIP2;4_*Oeu*030439.1
ATGGCAAAGGACGCTGGAGTGGCGGAGCATGGATCGTTCGCGGCAAAGGACTACCAAGATCCACCACCAGCTCCG
CTGATTGACGCGGAGGAGCTTACAAAGTGGTCATTTTACAGAGCTGTTATAGCCGAGTTTATAGCCACCTTGCTC
TTCCTTTACATTACGGTTTTGACTGTGATTGGGTACAAGAGCCAGATTGATACCACGGTTGGCCACGGCGGTGAT
CAGTGCGGTGGCGTTGGAATTCTTGGTATTGCATGGGCCTTTGGTGGAATGATCTTCGTTCTTGTCTACTGCACC
GCTGGAATTTCCGGGGGACACATTAATCCAGCAGTGACATTCGGGCTTTTCTTGGCCCGAAAGGTGTCAATGATC
AGAGCCATTATGTATATGGTGGCTCAGTGTTTGGGGGCCATTTGTGGCGTCGCGCTGGTGAAGGCGTTACAGAAG
TCACACTACAAGAAATATGGCGGCGGCGCAAATGAGCTCGCCGATGGCTACAGCACCGGCGTCGGATTATCCGCG
GAGATAATCGGAACATTCGTCCTTGTTTACACAGTTTTCTCTGCCACTGATCCCAAGAGAAGTGCCAGGGATTCC
CATGTCCCTGTATTGGCACCTCTTCCAATTGGATTTGCAGTTTTCATGGTTCATTTGGCCACCATCCCCATCACT
GGTACTGGTATCAACCCTGCTAGGAGTTTAGGGGCTGCAGTGATCTACAACCAGGAAAAAGCTTGGGATGATCAG
TGGATCTTCTGGGTTGGACCCTTCTTTGGTGCAGCCATTGCAGCCTTCTACCACCAGTTTATTTTGAGAGCAGGA
GCAGCTAAAGCCCTTGGTTCATTCAGAAGCAGCTCCCATGTT**TAA**
>*Oeu*PIP2;4_*Oeu*030439.1
MAKDAGVAEHGSFAAKDYQDPPPAPLIDAEELTKWSFYRAVIAEFIATLLFLYITVLTVIGYKSQIDTTVGHGGD
QCGGVGILGIAWAFGGMIFVLVYCTAGISGGHINPAVTFGLFLARKVSMIRAIMYMVAQCLGAICGVALVKALQK
SHYKKYGGGANELADGYSTGVGLSAEIIGTFVLVYTVFSATDPKRSARDSHVPVLAPLPIGFAVFMVHLATIPIT
GTGINPARSLGAAVIYNQEKAWDDQWIFWVGPFFGAAIAAFYHQFILRAGAAKALGSFRSSSHV*

***Oeu*PIP2;5 *Oeu*027987.1**

>*Oeu*PIP2;5_*Oeu*027987.1 | scaffold2335:58720..59930 reverse
ATGGGCAAGGACATTGAGGTTGGCGCCGAATACACCACCAAGGACTACCAAGATCCGCCACCGGCTCCGCTGATT
GACCCGGAGGAGCTAACCAAGTGGTCGTTCTACAGGGCCATTATAGCTGAATTCATCGCCACGCTCTTGTTCCTG
TACATAACTGTTCTGACTGTGATTGGGTACAAGAGCGAAAGCGCCGCCGATAAATGCGGCGGCGTCGGGATTCTT
GGCATAGCTTGGGCTTTTGGTGGCATGATTTTTGTCCTCGTTTACTGCACTGCTGGCATTTCCGGTAAGTACCTT
AATGTCTAGACTTCGACCGACTAAATTCCATTTAGAGGGAGCTAGAAATTTTGTTACATGTACGAATACTCAATA
TAATTCCAATGGGTGCAGGAGGGCACATCAACCCGGCAGTGACATTAGGCCTATTCTTGGCCCGTAAGGTGTCAC
TGGTTCGGGCTATCATGTACATGGTGGCGCAATGTTTAGGTGCCATTTGCGGTTGCGGCTTGGTAAAGGCATTCC
AGAAGTCTTACTATGTAAAATTCGGCGGCGGTGCCAATGAACTGGCGGATGGGTACAGTAACGGCACCGGATTGG
CGGCGGAGATCATTGGCACATTCGTCCTTGTTTACACCGTATTCTCTGCCACTGACCCCAAGAGAAATGCTAGAG
ACTCCCACGTACCTGTAAGTGCATTTGACTCCCCTAGTTTCTTCCATTTTTTATACGGACTTGTTAAAATCCCGG
TTCGACTCGGCCGGGTATGAATGAAAGGCTGACTCTGTTGGACCCTGTTGACACCCCTATCTAGGTCAAGAATTT
GTTTCCTCTGTTCGTGTTGATTAATCCTAATTGAAATTTTTGACAGGTTTTGGCACCTCTTCCAATTGGATTTGC
AGTGTTCATGGTTCATTTAGCCACAATCCCAATTACCGGCACGGGTATCAACCCGGCCCGAAGTTTAGGAGCTGC
TGTTATCTATGGCAAAGACAAGGCCTGGGATGATCAAGTACGCATTATTTCAATGTTTTACAATTTGTACCTTAT
TTCCAGGAAAAAAAAATTAACGTTAATTCTCATATATTTTGCAGTGGATTTTCTGGGTGGGACCATTCATTGGGG
CAGCCATTGCTGCATTTTACTACCAGTATATTCTGAGGGCAGGAGCTGTAAAAGCTCTGGGATCATTCAGGAGCA
CTGCTCAT**TGA**
>*Oeu*PIP2;5_*Oeu*027987.1
ATGGGCAAGGACATTGAGGTTGGCGCCGAATACACCACCAAGGACTACCAAGATCCGCCACCGGCTCCGCTGATT
GACCCGGAGGAGCTAACCAAGTGGTCGTTCTACAGGGCCATTATAGCTGAATTCATCGCCACGCTCTTGTTCCTG
TACATAACTGTTCTGACTGTGATTGGGTACAAGAGCGAAAGCGCCGCCGATAAATGCGGCGGCGTCGGGATTCTT
GGCATAGCTTGGGCTTTTGGTGGCATGATTTTTGTCCTCGTTTACTGCACTGCTGGCATTTCCGGAGGGCACATC
AACCCGGCAGTGACATTAGGCCTATTCTTGGCCCGTAAGGTGTCACTGGTTCGGGCTATCATGTACATGGTGGCG
CAATGTTTAGGTGCCATTTGCGGTTGCGGCTTGGTAAAGGCATTCCAGAAGTCTTACTATGTAAAATTCGGCGGC
GGTGCCAATGAACTGGCGGATGGGTACAGTAACGGCACCGGATTGGCGGCGGAGATCATTGGCACATTCGTCCTT
GTTTACACCGTATTCTCTGCCACTGACCCCAAGAGAAATGCTAGAGACTCCCACGTACCTGTTTTGGCACCTCTT
CCAATTGGATTTGCAGTGTTCATGGTTCATTTAGCCACAATCCCAATTACCGGCACGGGTATCAACCCGGCCCGA
AGTTTAGGAGCTGCTGTTATCTATGGCAAAGACAAGGCCTGGGATGATCAATGGATTTTCTGGGTGGGACCATTC
ATTGGGGCAGCCATTGCTGCATTTTACTACCAGTATATTCTGAGGGCAGGAGCTGTAAAAGCTCTGGGATCATTC
AGGAGCACTGCTCAT**TGA**
>*Oeu*PIP2;5_*Oeu*027987.1
MGKDIEVGAEYTTKDYQDPPPAPLIDPEELTKWSFYRAIIAEFIATLLFLYITVLTVIGYKSESAADKCGGVGIL
GIAWAFGGMIFVLVYCTAGISGGHINPAVTLGLFLARKVSLVRAIMYMVAQCLGAICGCGLVKAFQKSYYVKFGG
GANELADGYSNGTGLAAEIIGTFVLVYTVFSATDPKRNARDSHVPVLAPLPIGFAVFMVHLATIPITGTGINPAR
SLGAAVIYGKDKAWDDQWIFWVGPFIGAAIAAFYYQYILRAGAVKALGSFRSTAH*

***Oeu*PIP2;6 *Oeu*017257.1**

>*Oeu*PIP2;6_*Oeu*017257.1 | chr6:11700750..11703098 forward
ATGACAAAGGACGCAGGTGTGGCGGAGCACGGGTCGTTCACGACCAAGGACTACCATGATCCGCCTCCGGCGCCG
CTCATCGACGCGGAGGAGCTGACAAAATGGTCATTTTACAGGGCTATAATAGCTGAGTTCATAGCCACTTTGCTG
TTTCTTTACATTACAGTTTTGACTGTAATTGGGTACAAAAGCCAGACTGATCCCGACTTGAAGGGTGACCAGTGT
GATGGGGTTGGAATTCTTGGTATTGCATGGGCCTTTGGTGGCATGATCTTCATTCTGGTTTACTGCACTGCTGGA
ATTTCTGGTCAGTTTTATTTTGTTGAGACTCGAGATTCCATCTTAAGAGTTAATTTTTTTGTTACTATTTTCATA
TGTAACCATTTTGATTTACCCTTTAACATGATCAGCAAGATGGTGGCTTATGTGCCACAATTAATCTTGATATCT
GACATGATAAAGTTACATGCTCAATTAATTTTTCCAATAAAAATGGTGTAATTTGAGTGATCTGTGTGCATGCAG
GGGGGCACATAAACCCAGCAGTGACCTTTGGCCTGTTCTTGGCCCGGAAAGTGTCTTTGATCCGGGCAGTGATGT
ACATGGTGGCACAGTGTTTGGGAGCCATCTGCGGCGTTGGTTTGGTCAAGGCATTTCAGAAATCATACTACAAGA
AATACGGTGGCGGCGCCAACGTGCTGGCCGATGGGATCAGCACCGGCGTCGGGTTAGCTGCCGAAATCATCGGAA
CATTCGTCCTTGTTTACACTGTTTTCTCCGCCACTGATCCAAAGCGTAGTGCCAGAGATTCTCACGTCCCTGTAA
GTTTCTATGAGCTCAATTTTTGCTCAAGTGATTAAGCCTTAGAATATTGTGAAATATGTGATTAAAAATTAATTG
TTGACTTGACTTAAAATCTAACTTGATATAGATTTAAATTCCCTTTGAGTTTATTATAATTAGATAAGTAAAGAT
GTATCTTTTTTTGCTGTCTCGATTCAAGTTCGGTAAAGATTAGTAGTAGGGGTCAGCAGGGGCAGACCTACTTGA
GACCCAGCGGGTCGGCCGCCCCCACTCGATTTTCAAATTTTTAGTTTTTCTCCAAGTTTTTGTAGGTTTTCAATT
TTTTTCCCACGGTGTTTTGGTTTTGTCCCCCACTGCACTTCCGTCCTGAGTCCACCATTGAATGTGACCCATACC
AAATTGCAATATGCTGTACAACTGGGGCACTTGCCAGTATTATGTGGTTGTAAGTGAAATGAAGAAATTAATGTA
CCAAAGGGGACTTTACCTTGGTGACAGATGCAATGAATCAAGAAAAGTACAAGAGATATTATTGGTTTATCTAAT
TCGTTATGGACCATGAAACTTTGGATTATACCACTTTGTGAGAACGATTTTGACCGATGCAGTGAAAGAGGTATT

GGTGACAGCAGATGATTTGACGAGAAAGTTGTTCGTATTTTAAACAATTTAATGATAGAAAATTATATGGTATTA
TGTCAACCTGTCAACATATCTAGCACTATAACACCATTGTAAAATCTTACCTTGGGGACATGGATTCGTAGATTA
GATAGCAATATTAGTAGAAAACTTTTGTGGGCCCTATTAGAACTAAAAAAAATTCCACGCTTGTGGGGGATATCG
TGTGATATACAACAGTAGACAATGATACATGAATTTTGCTAAACGACTCGCACCGTTCATTACACCGATGACTGC
ACCCTCACATGATGCACGGGTCCCAATTGAATAAAAAAATGTCGATGCCATGTGTATGTGTGTGCGGTCACCTGC
GCGGGTCGTGTAGCGTGGTTCATGATACATTAGTGGATAAATTTTTTACCAAAAAAAAATATTCTTCTATAGTGA
TGTCCATTAATGTATTTAAATTCAACATACTAATTATGCCGAAGCCATTATTATTTCGAAAATACTAGGTATTGG
CACCTCTTCCAATTGGATTTGCAGTGTTCATGGTTCATTTGGCCACCATTCCAGTCACCGGAACGGGCATCAACC
CAGCTCGGAGTCTTGGAGCCGCAGTAATCTACGACCAGCACAAGGCTTGGGACGATCACGTTAGTACATATATTT
CAAATTAAATTTAGTTACTTCTCATTAAACGTATACTATAACGAAAATTTCTGTCTTGAAAAGGTCTTTTGATGA
CCCATAATTGGTGCTATTTAATCTAATAATTACATGTATATGTTTTTTCAGTGGATTTTCTGGGTCGGACCGTTT
ATCGGTGCAGCCGTTGCAGCATTCTACCACCAGTTTATATTGAGAGCTGGAGCAGCAAAGGCCCTTGGTTCATTC
AGGAGTAGTGCTTCTCATCAT**TGA**

>*Oeu*PIP2;6_*Oeu*017257.1

ATGACAAAGGACGCAGGTGTGGCGGAGCACGGGTCGTTCACGACCAAGGACTACCATGATCCGCCTCCGGCGCCG
CTCATCGACGCGGAGGAGCTGACAAAATGGTCATTTTACAGGGCTATAATAGCTGAGTTCATAGCCACTTTGCTG
TTTCTTTACATTACAGTTTTGACTGTAATTGGGTACAAAAGCCAGACTGATCCCGACTTGAAGGGTGACCAGTGT
GATGGGGTTGGAATTCTTGGTATTGCATGGGCCTTTGGTGGCATGATCTTCATTCTGGTTTACTGCACTGCTGGA
ATTTCTGGGGGGCACATAAACCCAGCAGTGACCTTTGGCCTGTTCTTGGCCCGGAAAGTGTCTTTGATCCGGGCA
GTGATGTACATGGTGGCACAGTGTTTGGGAGCCATCTGCGGCGTTGGTTTGGTCAAGGCATTTCAGAAATCATAC
TACAAGAAATACGGTGGCGGCGCCAACGTGCTGGCCGATGGGATCAGCACCGGCGTCGGGTTAGCTGCCGAAATC
ATCGGAACATTCGTCCTTGTTTACACTGTTTTCTCCGCCACTGATCCAAAGCGTAGTGCCAGAGATTCTCACGTC
CCTGTATTGGCACCTCTTCCAATTGGATTTGCAGTGTTCATGGTTCATTTGGCCACCATTCCAGTCACCGGAACG
GGCATCAACCCAGCTCGGAGTCTTGGAGCCGCAGTAATCTACGACCAGCACAAGGCTTGGGACGATCACTGGATT
TTCTGGGTCGGACCGTTTATCGGTGCAGCCGTTGCAGCATTCTACCACCAGTTTATATTGAGAGCTGGAGCAGCA
AAGGCCCTTGGTTCATTCAGGAGTAGTGCTTCTCATCAT**TGA**

>*Oeu*PIP2;6_*Oeu*017257.1

MTKDAGVAEHGSFTTKDYHDPPPAPLIDAEELTKWSFYRAIIAEFIATLLFLYITVLTVIGYKSQTDPDLKGDQC
DGVGILGIAWAFGGMIFILVYCTAGISGGHINPAVTFGLFLARKVSLIRAVMYMVAQCLGAICGVGLVKAFQKSY
YKKYGGGANVLADGISTGVGLAAEIIGTFVLVYTVFSATDPKRSARDSHVPVLAPLPIGFAVFMVHLATIPVTGT
GINPARSLGAAVIYDQHKAWDDHWIFWVGPFIGAAVAAFYHQFILRAGAAKALGSFRSSASHH*

*Oeu*PIP2;7 *Oeu*018111.1

>*Oeu*PIP2;7_*Oeu*018111.1 | scaffold176:1251179..1252336 forward

ATGGTGAAGGATGTTGAGGTCGGAGGCCCTGAATTTATTACAAAAGACTATCAAGACCCTCCTCCAGCACCGTTG
ATTGATCCGGAAGAACTGACTAAATGGTCATTTTATAGAGCTATCATTGCTGAATTCGTAGCTACATTACTGTTC
TTATACATTACTGTTTTGACTGTGATTGGGTACAAGAGCCAGAGCGCCACCGATGAATGCGGCGGCGTTGGCATT
TTGGGCATTGCTTGGGCCTTTGGTGGCATGATTTTCGTACTTGTTTACTGCACTGCTGGTATTTCCGGTGAGTTG
CACATCTTAAATATGATTCAAATCATAAAATTGACAAAGTTTTAACACAGATTCCTCTTTCTTTGTGATGATATT
TACGACGTTAATGGTGCAGGAGGGCACATTAACCCGGCAGTGACATTCGGGTTGTTTTTAGCCCGAAAAGTTTCA
TTGATTCGGGCCATTATGTACATGGTGGCTCAGTGTGTAGGGGCCATTTGTGGGTGTGGATTGGTGAAGGCATTC
CAGAGGGCTTACTACGTTAGATACGGTGGTGGCGCCAATGAACTTGGAGATGGATATAGCACAGGCACCGGATTG
GCGGCCGAGATTATCGGCACTTTTGTGCTTGTTTACACTGTCTTCTCCGCCACGGACCCTAAAAGAAGTGCCAGA
GACTCCCATGTTCCGGTAATATCCGTGCTTAAGTTTTTAGCCAAACAAACTCCATAATTTTAGCAAGTCAAGATT
TTCTTCAATCAATTTTGACTGTTGATTAATCCTACTTGAAAATTAATCTAAAGGTATTGGCACCACTGCCTATTG
GATTTGCTGTGTTCATGGTTCATTTGGCTACAATTCCGATCACCGGCACTGGTATCAACCCTGCCCGAAGTTTTG
GAGCTGCAGTGATCTATGGCAAGGATAAGGCCTGGGACGATCAAGTATTGAACATTATTTAAAAATTTGTTTATT
TTATTGTTAAGTGTTGTCGAGTACGGCTGCTGACTGACCCTTTTGATCGATTTTTGACAGTGGATCTTCTGGGCT
GGACCTTTCATTGGGGCAGCCATTGCTGCATTGTACCATCAGTACATTCTTAGAGCAGGTGCAGTTAAAGCTCTA
GGATCATTCAGGAGTAGTGCTTCTGCCTAT**TGA**

>*Oeu*PIP2;7_*Oeu*018111.1

ATGGTGAAGGATGTTGAGGTCGGAGGCCCTGAATTTATTACAAAAGACTATCAAGACCCTCCTCCAGCACCGTTG
ATTGATCCGGAAGAACTGACTAAATGGTCATTTTATAGAGCTATCATTGCTGAATTCGTAGCTACATTACTGTTC
TTATACATTACTGTTTTGACTGTGATTGGGTACAAGAGCCAGAGCGCCACCGATGAATGCGGCGGCGTTGGCATT
TTGGGCATTGCTTGGGCCTTTGGTGGCATGATTTTCGTACTTGTTTACTGCACTGCTGGTATTTCCGGAGGGCAC
ATTAACCCGGCAGTGACATTCGGGTTGTTTTTAGCCCGAAAAGTTTCATTGATTCGGGCCATTATGTACATGGTG
GCTCAGTGTGTAGGGGCCATTTGTGGGTGTGGATTGGTGAAGGCATTCCAGAGGGCTTACTACGTTAGATACGGT
GGTGGCGCCAATGAACTTGGAGATGGATATAGCACAGGCACCGGATTGGCGGCCGAGATTATCGGCACTTTTGTG
CTTGTTTACACTGTCTTCTCCGCCACGGACCCTAAAAGAAGTGCCAGAGACTCCCATGTTCCGGTATTGGCACCA
CTGCCTATTGGATTTGCTGTGTTCATGGTTCATTTGGCTACAATTCCGATCACCGGCACTGGTATCAACCCTGCC
CGAAGTTTTGGAGCTGCAGTGATCTATGGCAAGGATAAGGCCTGGGACGATCAATGGATCTTCTGGGCTGGACCT

TTCATTGGGGCAGCCATTGCTGCATTGTACCATCAGTACATTCTTAGAGCAGGTGCAGTTAAAGCTCTAGGATCA
TTCAGGAGTAGTGCTTCTGCCTAT**TGA**
>*Oeu*PIP2;7_*Oeu*018111.1
MVKDVEVGGPEFITKDYQDPPPAPLIDPEELTKWSFYRAIIAEFVATLLFLYITVLTVIGYKSQSATDECGGVGI
LGIAWAFGGMIFVLVYCTAGISGGHINPAVTFGLFLARKVSLIRAIMYMVAQCVGAICGCGLVKAFQRAYYVRYG
GGANELGDGYSTGTGLAAEIIGTFVLVYTVFSATDPKRSARDSHVPVLAPLPIGFAVFMVHLATIPITGTGINPA
RSFGAAVIYGKDKAWDDQWIFWAGPFIGAAIAALYHQYILRAGAVKALGSFRSSASAY*

*Oeu*PIP2;8 *Oeu*023232.1

Corrected as follow : Exon 1 extended in its 5'end with 84n

>*Oeu*PIP2;8_*Oeu*023232.1 | scaffold20321:54..1081 reverse original version
ATGGGTGAGCTCATGCTCTGGTCCTTTTACAGAGCCATAATTGCCGAGTTCATTGCCACCCTGCTCTTCCTTTAC
GTCACCGTCGCCACCGTCATCGGCCACAAGAAGCTCAACGCCGCCGACCAATGCGATGGCGTTGGCATCCTCGGC
ATCGCTTGGGCCTTTGGTGGCATGATCTTCATCCTTGTTTACTGCACCGCCGGCATCTCAGGTACATGATTCACT
GCCTCAACTTCTTCAAGACAAATCACCACTTATTTCTTGAAATTTTACCGGAATTTTTCTCTGCTGTTCTACAG
GTGGTCACATAAACCCGGCTGTGACTTTCGGGTTGTTCTTGGCCAGGAAGGTGTCGTTGATCCGTGCAATTATGT
ACATGATTGCACAGTGTTTGGGTGCCGTCTGTGGAGTTGGCCTGGTGAAGGCCTTCATGAAGAGCTACTACAACC
GTCTGGGCGGCGGCGCTAACGTGGTGTCTCATGGGTACAATATCGGCACAGCTTTGGGTGCTGAAATTATCGGTA
CCTTTGTTCTTGTATACACCGTCTTCTCGGCCACTGACCCCAAGAGAAGTGCACGTGACTCTCACGTCCCTGTAA
GTAGAAAAAAATAGCTAGCTTATTTGTGGGTTTTTCCTTGTCTTTTCTAAGTTGACTTATTTTTGGATTGTTT
TAGGTTTTGGCTCCTCTTCCAATTGGGTTTGCAGTTTTCATGGTGCACTTAGCGACCATCCCCATCACTGGAACT
GGTATTAACCCTGCAAGGAGTTTTGGAGCTGCTGTGATCTACAACAATGACAAAGTCTGGGATGACCATGTAAGT
TGGTTTGGATAAATTACTTTCTTTTTTTTCGTTTCTTACTTTTGTTGAACTTAAAAGTAAAAAAATTTGGATGGT
TCATGCAGTGGATTTTCTGGGTAGGACCATTTGTGGGAGCTTTGGCGGCAGCAGCATATCATCAGTACATCTTGA
GAGCTGCAGCTATTAAGGCAATTGGATCATTCCGCAGCAATCCCACAAAC**TAA**
>*Oeu*PIP2;8_*Oeu*023232.1 | scaffold20321:54..1165 reverse Corrected version
ATGTCGAAAGAAGTGACTGAAGAAGGACAAGTACACCCCCATGGAAAGGACTATGAGGACCCACCGCCAGCACCA
TTACTGGACATGGGTGAGCTCATGCTCTGGTCCTTTTACAGAGCCATAATTGCCGAGTTCATTGCCACCCTGCTC
TTCCTTTACGTCACCGTCGCCACCGTCATCGGCCACAAGAAGCTCAACGCCGCCGACCAATGCGATGGCGTTGGC
ATCCTCGGCATCGCTTGGGCCTTTGGTGGCATGATCTTCATCCTTGTTTACTGCACCGCCGGCATCTCAGGTACA
TGATTCACTGCCTCAACTTCTTCAAGACAAATCACCACTTATTTCTTGAAATTTTACCGGAATTTTTCTCTGCT
GTTCTACAGGTGGTCACATAAACCCGGCTGTGACTTTCGGGTTGTTCTTGGCCAGGAAGGTGTCGTTGATCCGTG
CAATTATGTACATGATTGCACAGTGTTTGGGTGCCGTCTGTGGAGTTGGCCTGGTGAAGGCCTTCATGAAGAGCT
ACTACAACCGTCTGGGCGGCGGCGCTAACGTGGTGTCTCATGGGTACAATATCGGCACAGCTTTGGGTGCTGAAA
TTATCGGTACCTTTGTTCTTGTATACACCGTCTTCTCGGCCACTGACCCCAAGAGAAGTGCACGTGACTCTCACG
TCCCTGTAAGTAGAAAAAAATAGCTAGCTTATTTGTGGGTTTTTCCTTGTCTTTTCTAAGTTGACTTATTTTTT
GGATTGTTTTAGGTTTTGGCTCCTCTTCCAATTGGGTTTGCAGTTTTCATGGTGCACTTAGCGACCATCCCCATC
ACTGGAACTGGTATTAACCCTGCAAGGAGTTTTGGAGCTGCTGTGATCTACAACAATGACAAAGTCTGGGATGAC
CATGTAAGTTGGTTTGGATAAATTACTTTCTTTTTTTTCGTTTCTTACTTTTGTTGAACTTAAAAGTAAAAAAAT
TTGGATGGTTCATGCAGTGGATTTTCTGGGTAGGACCATTTGTGGGAGCTTTGGCGGCAGCAGCATATCATCAGT
ACATCTTGAGAGCTGCAGCTATTAAGGCAATTGGATCATTCCGCAGCAATCCCACAAAC**TAA**
>*Oeu*PIP2;8_*Oeu*023232.1
ATGTCGAAAGAAGTGACTGAAGAAGGACAAGTACACCCCCATGGAAAGGACTATGAGGACCCACCGCCAGCACCA
TTACTGGACATGGGTGAGCTCATGCTCTGGTCCTTTTACAGAGCCATAATTGCCGAGTTCATTGCCACCCTGCTC
TTCCTTTACGTCACCGTCGCCACCGTCATCGGCCACAAGAAGCTCAACGCCGCCGACCAATGCGATGGCGTTGGC
ATCCTCGGCATCGCTTGGGCCTTTGGTGGCATGATCTTCATCCTTGTTTACTGCACCGCCGGCATCTCAGGTGGT
CACATAAACCCGGCTGTGACTTTCGGGTTGTTCTTGGCCAGGAAGGTGTCGTTGATCCGTGCAATTATGTACATG
ATTGCACAGTGTTTGGGTGCCGTCTGTGGAGTTGGCCTGGTGAAGGCCTTCATGAAGAGCTACTACAACCGTCTG
GGCGGCGGCGCTAACGTGGTGTCTCATGGGTACAATATCGGCACAGCTTTGGGTGCTGAAATTATCGGTACCTTT
GTTCTTGTATACACCGTCTTCTCGGCCACTGACCCCAAGAGAAGTGCACGTGACTCTCACGTCCCTGTTTTGGCT
CCTCTTCCAATTGGGTTTGCAGTTTTCATGGTGCACTTAGCGACCATCCCCATCACTGGAACTGGTATTAACCCT
GCAAGGAGTTTTGGAGCTGCTGTGATCTACAACAATGACAAAGTCTGGGATGACCATTGGATTTTCTGGGTAGGA
CCATTTGTGGGAGCTTTGGCGGCAGCAGCATATCATCAGTACATCTTGAGAGCTGCAGCTATTAAGGCAATTGGA
TCATTCCGCAGCAATCCCACAAAC**TAA**
>*Oeu*PIP2;8_*Oeu*023232.1
MSKEVTEEGQVHPHGKDYEDPPPAPLLDMGELMLWSFYRAIIAEFIATLLFLYVTVATVIGHKKLNAADQCDGVG
ILGIAWAFGGMIFILVYCTAGISGGHINPAVTFGLFLARKVSLIRAIMYMIAQCLGAVCGVGLVKAFMKSYYNRL
GGGANVVSHGYNIGTALGAEIIGTFVLVYTVFSATDPKRSARDSHVPVLAPLPIGFAVFMVHLATIPITGTGINP
ARSFGAAVIYNNDKVWDDHWIFWVGPFVGALAAAAYHQYILRAAAIKAIGSFRSNPTN*

***Oeu*PIP2;9 *Oeu*020106.1/scaffold870**

Observation: Two scaffolds have been linked to generate *Oeu*PIP2;9. The scaffold Oeu020106.1 is underlined
>Oeu020106.1 | Scaffold18654:1671..2018 forward
>Scaffold870 | Scaffold870:312817..313578 forward

>*Oeu*PIP2;9_*Oeu*020106.1/Scaffold870
ATGACGAAGGAAGTGAGTGAAGAAGGACAGGTACATCACCATGGAAAGGACTACGTGGACCCACCACCAGCACCA
TTGCTAGACATGGGTGAGCTCAAGCTCTGGTCCTTTTACAGAGCTCTTATCGCCGAGTTCATCGCCACCCTTCTC
TTCCTCTACGTCACCGTCGCTACCGTCATCGGCCACAAGAAGCTCAACGCGGCCGACCAATGTGATGGCGTTGGC
ATTCTCGGTATCGCCTGGGCCTTTGGTGGCATGATCTTCATCCTCGTTTACTGTACCGCCGGCATCTCAGGTGGT
CACATAAACCCGGCTGTGACTTTTGGTTTGTTCTTGGCAAGGAAGGTATCGTTGATCCGTGCTGTTATGTACATG
ATTGCACAGTGTTTGGGTGCCATTTGTGGAGTTGGGCTGGTGAAGGCCTTCATGAAGGGGTACTACAACCGTCTA
GGCGGCGGCGCTAACTCGGTGTCTCATGGTTACAACACAGGCACAGCTTTGGGTGCTGAAATCATCGGCACCTTT
GTGCTTGTTTACACCGTCTTCTCCGCCACTGACCCCAAGAGAAGTGCACGTGACTCTCATGTCCCTGTAAGTAGA
AGCAATTTCTAGCTTATTTTGTGTTTGCTTTTTCTAAAGTGAACTTGAACTTATTTTGGATTATTTTCAGGTTTT
GGCTCCTTTACCAATTGGATTTGCAGTGTTCGTGGTGCACTTGGCCACCATCCCTATTACTGGAACTGGTATCAA
CCCTGCTAGGAGTTTTGGAGCTGCCGTCATCTTCAACAATGATAAAGTCTGGGATGACCATGTAAGTTCGTCTAG
TAAGTTACTTTTTTTTCAAGTATATATAATTTATTTTCTTGAACTAATAAGTATAATTTTGATGATGCATGTAGT
GGATTTTCTGGGTTGGACCATTTGTGGGAGCATTGGCAGCAGCAGCATATCATCAGTACATCTTAAGGGCTGCAG
CTATTAAGGCATTGGGATCATTCCGCAGCAACCCCACAAAC**TAA**
>*Oeu*PIP2;9_*Oeu*020106.1/Scaffold870
ATGACGAAGGAAGTGAGTGAAGAAGGACAGGTACATCACCATGGAAAGGACTACGTGGACCCACCACCAGCACCA
TTGCTAGACATGGGTGAGCTCAAGCTCTGGTCCTTTTACAGAGCTCTTATCGCCGAGTTCATCGCCACCCTTCTC
TTCCTCTACGTCACCGTCGCTACCGTCATCGGCCACAAGAAGCTCAACGCGGCCGACCAATGTGATGGCGTTGGC
ATTCTCGGTATCGCCTGGGCCTTTGGTGGCATGATCTTCATCCTCGTTTACTGTACCGCCGGCATCTCAGGTGGT
CACATAAACCCGGCTGTGACTTTTGGTTTGTTCTTGGCAAGGAAGGTATCGTTGATCCGTGCTGTTATGTACATG
ATTGCACAGTGTTTGGGTGCCATTTGTGGAGTTGGGCTGGTGAAGGCCTTCATGAAGGGGTACTACAACCGTCTA
GGCGGCGGCGCTAACTCGGTGTCTCATGGTTACAACACAGGCACAGCTTTGGGTGCTGAAATCATCGGCACCTTT
GTGCTTGTTTACACCGTCTTCTCCGCCACTGACCCCAAGAGAAGTGCACGTGACTCTCATGTCCCTGTTTTGGCT
CCTTTACCAATTGGATTTGCAGTGTTCGTGGTGCACTTGGCCACCATCCCTATTACTGGAACTGGTATCAACCCT
GCTAGGAGTTTTGGAGCTGCCGTCATCTTCAACAATGATAAAGTCTGGGATGACCATTGGATTTTCTGGGTTGGA
CCATTTGTGGGAGCATTGGCAGCAGCAGCATATCATCAGTACATCTTAAGGGCTGCAGCTATTAAGGCATTGGGA
TCATTCCGCAGCAACCCCACAAAC**TAA**
>*Oeu*PIP2;9_*Oeu*020106.1/Scaffold870
MTKEVSEEGQVHHHGKDYVDPPPAPLLDMGELKLWSFYRALIAEFIATLLFLYVTVATVIGHKKLNAADQCDGVG
ILGIAWAFGGMIFILVYCTAGISGGHINPAVTFGLFLARKVSLIRAVMYMIAQCLGAICGVGLVKAFMKGYYNRL
GGGANSVSHGYNTGTALGAEIIGTFVLVYTVFSATDPKRSARDSHVPVLAPLPIGFAVFVVHLATIPITGTGINP
ARSFGAAVIFNNDKVWDDHWIFWVGPFVGALAAAAYHQYILRAAAIKALGSFRSNPTN*

***Oeu*PIP2;10 *Oeu*022438.1/scaffold2530**

Observation: corrected by elongating the 5′ end with 66n, and elongated in the 3′ end by linking the Scaffold Scaffold2530 (this latter is underlined)

>*Oeu*PIP2;10_*Oeu*022438.1 | scaffold19979:146..1262 reverse original version
ATGGAGCTTACAAAGTGGTCATTTTATAGAGCTGTTATAGCTGAGTTTATAGCCACCTTGCTCTTCCTTTACATT
ACGGTTTTGACTGTGATTGGGTACAAGAGCCAGAATGATACTCATTGCGGCGGCGGCGGCGTCGGAATTCTTGGT
ATTGCATGGGCATTTGGTGGCATGATCTTCGTTCTTGTCTACTGCACTGCTGGAATCTCCGGTGAGTCTTGTTTA
TACATATACATACATACATCAGATGCATAAATGTAATGGCATATTTCATGTCATGTCCATCTCAGGGGGACACAT
TAATCCAGCAGTGACATTCGGGCTTTTCTTGGCCCGACAGGTGTCAATGATCAAAGCCATTATGTATATGGTGGC
TCAGTGTTTGGGGGCCATTTGTGGCGTCGCGCTGGTGAAGGCGGTACAGAAGTCACACTACAAGAAATATGGCGG
CGGCGTAAATGAGCTCTCCGATGGCTACAGCACCGGCGGCGGATTGGCAGCAGAGATCATCGGAACATTCGTTCT
TGTCTACACAGTTTTCTCTGCCATTTATGCCAAGAGAAAAGCCAGGGATTCCCATGTCCCTGTAAGTGCATATAT
ACAATTCTCCATTTTTGACCTGCTCGATAATCTACTTATAAGTACATTTAGAGCATGTGTTTTTCAATTAAACAC
TTGGTAAGTGAATGCCATGCATTATGATACCATACACGTGAAACTGCAAAACTTCGTGGTTCAATAATGATACCA
TGCACGTTGTACTGTAAAACCATATTGTCCATGCATCATTATTACATCAATATGGAGTTCCAATTTTTTTTTACG
ATAAGATTTGTATTGCTTGAGTTTGAAATCGAACTTGTCACAGTTAATCTTATTTTGAGCTCGACTTAATTCAGT
TCTTTTATCTATACGAAAGGAGCGAATATTGATATTTTTTGTATTTGAACTTGAATCATAGGTTTTGGCACCTCT
CTCAATTGGATTTGCAGTTTTTATGGTTCACTTGGCCACTATCCCGATCACTGGTACTAGTATTAATCCAGCTAG
AAGCTTTGGACCTGCAGTGATCTACAACCAGAAAAAGGCTTGGGATGATCAGGTTAACATAGCC**TAA**

>*Oeu*PIP2;10_*Oeu*022438.1 Corrected version
ATGGAAAACCACGTTGGAGTGGCGAAGGACTACCGCGATCCACTGCCGGCCCCGCTGATTGGCTTGATGGAGCTT
ACAAAGTGGTCATTTTATAGAGCTGTTATAGCTGAGTTTATAGCCACCTTGCTCTTCCTTTACATTACGGTTTTG
ACTGTGATTGGGTACAAGAGCCAGAATGATACTCATTGCGGCGGCGGCGGCGTCGGAATTCTTGGTATTGCATGG
GCATTTGGTGGCATGATCTTCGTTCTTGTCTACTGCACTGCTGGAATCTCCGGTGAGTCTTGTTTATACATATAC
ATACATACATCAGATGCATAAATGTAATGGCATATTTCATGTCATGTCCATCTCAGGGGGACACATTAATCCAGC
AGTGACATTCGGGCTTTTCTTGGCCCGACAGGTGTCAATGATCAAAGCCATTATGTATATGGTGGCTCAGTGTTT
GGGGGCCATTTGTGGCGTCGCGCTGGTGAAGGCGGTACAGAAGTCACACTACAAGAAATATGGCGGCGGCGTAAA
TGAGCTCTCCGATGGCTACAGCACCGGCGGCGGATTGGCAGCAGAGATCATCGGAACATTCGTTCTTGTCTACAC
AGTTTTCTCTGCCATTTATGCCAAGAGAAAAGCCAGGGATTCCCATGTCCCTGTAAGTGCATATATACAATTCTC
CATTTTTGACCTGCTCGATAATCTACTTATAAGTACATTTAGAGCATGTGTTTTTCAATTAAACACTTGGTAAGT
GAATGCCATGCATTATGATACCATACACGTGAAACTGCAAAACTTCGTGGTTCAATAATGATACCATGCACGTTG
TACTGTAAAACCATATTGTCCATGCATCATTATTACATCAATATGGAGTTCCAATTTTTTTTACGATAAGATTT
GTATTGCTTGAGTTTGAAATCGAACTTGTCACAGTTAATCTTATTTTGAGCTCGACTTAATTCAGTTCTTTTATC
TATACGAAAGGAGCGAATATTGATATTTTTTGTATTTGAACTTGAATCATAGGTTTTGGCACCTCTCTCAATTGG
ATTTGCAGTTTTTATGGTTCACTTGGCCACTATCCCGATCACTGGTACTAGTATTAATCCAGCTAGAAGCTTTGG
ACCTGCAGTGATCTACAACCAGAAAAAGGCTTGGGATGATCAGGTTAACATAGCC**TAA**AAAGATTTATAACGAAC
TAAGGGTGCTTTTGGTTTGTCATTTTCAGTTTTGCAAAATACTGAAAACGTGTTTGATCTGTTATTTTAGAAAAT
GCAGTTTTGAAAATAAGCCGTTTGGTATCCATTTTTTTCAAAATGAATTTTTCTCTTTTTTCTTTTCAACCACAC
ATTTTTAATTAATATAATATTACACATATATCAAAAACTTCTCCTTATTTTTCAACTACACATTTTTCATCAATT
TACTATTCAAAAATGCATTTTTACATAAAAATATCTCCCAAACGCCCTCTAAATTTATAATAAAAACAACGTTAT
ATATATAGAATAATTTTATGGAACTAAATGTCATACAATAAATTTTCAAAATTTTAAACCAAACACGTTTTCTAA
TTTTTTGTTTTGAGATTTTTTTTTTTAAAATGACAAATCAAACGAGTTTTTACCAATATTGAATAGATCGTCAAA
ACAGAGACCTAAAATGAACTCAAATTGAAAATTGAAACATAAACCAAACAGACCTAAATCTTACGATGAAATTTG
GATCTACAACTTGAATATTTTTTCTCTCCTCTATTCAATTCATTGTTGATGCTATTGGGGTGTCCAAATGAAAGA
TGTTAGTAAGACTAAAGTCTTACTAAAGTCAGGGAGAATATTTTATTTCAATTACTCCTAATTATTTATCCGACA
AGAAGAATGATTTTTAAAACGCCACACTAGTTAACTAAAAGTTACATGGATCCCTTTCTATAAGTACTTTGTTCC
TTTATTGCCGAAATAGTCCTAATGAAATGCCTAGTAGGATTCATATTATAAAATATGAGAAAATTAATATGCGTT
TAATAATAACCTAGCTCAATCTTTATGATCCTTAATATATATGAACTCGAGATTGAGTATGGAAAAAATATTTAT
ATCCCAACACTAGTTGGAACGACTATGAACAAAATACAATATGAGTACCATTCCTTTTAGTCATAAAATTTATAA
ATCCCTGTTTGGTTCCTATTCTTTTCTCATTAAAATGAAAAATATGATAGTTATTCATCATATCAAGTCACCAAA
TTTGAATTTATATTTCTTGTGTGGGGTCCGTAACGACTATATTTTTCATTCTCATTCTCCCAAAAATGAGAAACA
AACGGGGGCTAATTTTAACATTATCTACCCCACCAGCCCACAAGTGATAATTTATTTGCTTATTTTTGTTCTCTT
AATTCCAGTGGATATTCTGGGTAGGACCCTTCATTGGTGCAGCCATGGCAGCCTTGTACCACCAGTGTATTTTGA
GAGCTAAAGCCTTTCGTTCATATAGAAGCAGCTCCCGTATT**TAA**

>*Oeu*PIP2;10_*Oeu*022438.1/Scaffold2530
ATGGAAAACCACGTTGGAGTGGCGAAGGACTACCGCGATCCACTGCCGGCCCCGCTGATTGGCTTGATGGAGCTT
ACAAAGTGGTCATTTTATAGAGCTGTTATAGCTGAGTTTATAGCCACCTTGCTCTTCCTTTACATTACGGTTTTG
ACTGTGATTGGGTACAAGAGCCAGAATGATACTCATTGCGGCGGCGGCGGCGTCGGAATTCTTGGTATTGCATGG
GCATTTGGTGGCATGATCTTCGTTCTTGTCTACTGCACTGCTGGAATCTCCGGGGGACACATTAATCCAGCAGTG
ACATTCGGGCTTTTCTTGGCCCGACAGGTGTCAATGATCAAAGCCATTATGTATATGGTGGCTCAGTGTTTGGGG
GCCATTTGTGGCGTCGCGCTGGTGAAGGCGGTACAGAAGTCACACTACAAGAAATATGGCGGCGGCGTAAATGAG
CTCTCCGATGGCTACAGCACCGGCGGCGGATTGGCAGCAGAGATCATCGGAACATTCGTTCTTGTCTACACAGTT
TTCTCTGCCATTTATGCCAAGAGAAAAGCCAGGGATTCCCATGTCCCTGTTTTGGCACCTCTCTCAATTGGATTT
GCAGTTTTTATGGTTCACTTGGCCACTATCCCGATCACTGGTACTAGTATTAATCCAGCTAGAAGCTTTGGACCT
GCAGTGATCTACAACCAGAAAAAGGCTTGGGATGATCAGTGGATATTCTGGGTAGGACCCTTCATTGGTGCAGCC
ATGGCAGCCTTGTACCACCAGTGTATTTTGAGAGCTAAAGCCTTTCGTTCATATAGAAGCAGCTCCCGTATT**TAA**

>*Oeu*PIP2;10_*Oeu*022438.1/Scaffold2530
MENHVGVAKDYRDPLPAPLIGLMELTKWSFYRAVIAEFIATLLFLYITVLTVIGYKSQNDTHCGGGGVGILGIAW
AFGGMIFVLVYCTAGISGGHINPAVTFGLFLARQVSMIKAIMYMVAQCLGAICGVALVKAVQKSHYKKYGGGVNE
LSDGYSTGGGLAAEIIGTFVLVYTVFSAIYAKRKARDSHVPVLAPLSIGFAVFMVHLATIPITGTSINPARSFGP
AVIYNQKKAWDDQWIFWVGPFIGAAMAALYHQCILRAKAFRSYRSSSRI*

_Oeu_PIP2;11 _Oeu_017254.1/_Oeu_017256.1

>_Oeu_017254.1 | chr6:11625929..11647223 forward

***Observation*:**
5′ end extended with 36n,
3′ located 21268n after TAA
Intron 2 truncated for the analysis // (19421n)
PIP2 sequence considered as pseudogene but keept for the *in silico* analysis with its hotologous *Oleur*PIP2;11.

>_Oeu_PIP2;11_Oeu017254.1/_Oeu_017256.1 corrected version
ATGAAGGACGTAGGGGTGGCGAATCACGAGTCGTTCATGGCCAAGGACTACCATGATCCACCGCCGGCGCCGCTC
ATCGACACAGAGGAGCTGACGAAGTGGTCGTTTTACAGAGCTTTAATAGCTGAGTTCATAGCCACTTTACTATTT
CTTTACATTACAGTTTTGACTGTAATTGGGTATAAGAGCCATCTTGATACTGACTTGAAGGGTGACCAGTGTGGT
GGTGTGCGAATTCTTGGTATTGCATGGGCCTTTGGAGGCATGATCTTCATTCTTGTCTACTGCACTGCTGGAATT
TCTGGTTAGTTTTTCTTTCTTTACATTGAGCATCAAGATTCTATCTTAAAACGTCAACATTATAATTAAGAAGAT
TAGGTGAATGTTTGGTATTGATTTTTCACTCCAAAAAATCAATTTTTTGATCGTTATTTCACTATTCAAGCTGTC
ATTTTTTACTTCCACTCTCACGGGTAGGACCCACTTTTTTTTCCGACGGTCAAAAAATCAATTTTCAAAATCACT
ACCAAACAAGCCCTAGATCAATCTCTTGGTTCTTATTTTTATATGTTAAGTGACGTGAGATTTATTGTCAACATA
TTTCGTGCAATGTTGGAAATTGAATGCTCCTTTAAGCTTTAAATAGATCGAATTATTCACATGAACTTCTTGTGC
TTGTATATTAAATCCATGTTCGACAATCAATAAGAAAACTCATTTTTTTCTCGAGCTCATTTTCAACAAAATAAT
TTGTACGTAAATTCTTAACTTTTTTCATACTTTTTAACCAGTAATCACAAAATCTCAAAAAAAATCAGTATTTCG
AAACTCATTTTTCTGAAAAGCTCAGTTTAATGATTGCCAAACAGGTAGCCTAAAGGATCAAATAACTCTGTGAGC
ATGTTGGGGTTTGGAAAAATCTTGGGGTTTCATTTTAAAATCTTTACTTTGTTAGAGTAGTATCACAATTTTTAA
TAAAGTGATTAACGCCATCATCATATTTAACTGAACATTTAGAACAAGAATAAAATTCCCTCTAAACATGCAGAT
GCTTAATTTGTTCACCAAAAATAGTGTAATTTGAGTGATCTGTGTGCATGCAGGGGGGCATATAAACCCAGCAGT
GACTTTTGGCCTGTTTTTGGCCCGAAAAGTGTCTTTGATCCGGGCAGTAATGTACATGGTGGCACAATGTTTGGG
GGCCATTTGCGGTGTCGGTTTGGTCAAATCCTTTCAAAAATCATACTACAGGAAATACGGCGGTGGCTCCAATGA
GCTGGCAGAAGGGTACAGCACCAGCGTCGGGCTATCTGTCGAATGTAAGTATTATGCCCTAGGTCCAATCGACTT
TGAACATAAATACTTTTATATATAATTAATGCAATTAAATGGCAGATCTATTATTTAATTAATAGACAATTAAAT
AAATAATAGAGTCCGTAGATTCAAATATTTGTAATTTATTAATCACAAGGAGTTGTGATTGTGAGATACAAATTA
CATCTGATTGAATCTTAAATTATCCGTGGTCGTAAGTAATAACGAGATACGGATATCGTTATTACGGATAGACCA
TCACGTGTGGCATTTCTTACTTGTGAAGTAAGTAGCTGATCTCACAAGTTAAAGTGTGGTAACACTTAAGTGAAC
ACGTGGATGTTTGTTATAGAACAAAACATTGGAGACCCGTCGAGAGAATCCACATGGATAACTATCTGAGTGTCG
GTGGTTAATCTCTAACGTTCGGTAGTGAAATTGGTCCTTTGACTTGAGGTCGTCATGGTACCTTATATGAGGACA
GTCATACTTTGGCGGTGTTGGATGTTGCTCTGAAAAGGGCCGCTAATACGACATCGTTCGGGTACGTCAAGAATC
GTATGAAGGTTGTGATTGATCAATAAAGGAT//TCGTCACTCTTAGATAGTAGGAGATTATGATCTGTGTTTCAT
CCAATTAAGTCAATGATCAAATCCTTGGCCAAGGTGTACTCATAAGTCGAAAAGGAGTTTTCGATTTATATTGAC
TTAATAGTGGAGTGAAAGATATGATTGATGATCGAGATTGACACAACTCCATATCTCGTAATTGATCGGATCATA
TAAATAAACAAAATTTAACTTATTTTTCTTCTATAATACTACATAgataaaaatatttgtacaaaatcttgttga
taaaatggtgtactcactctcacaaaattagcggactccacatgaatttaaaaaatgtcgacttataatctccac
acctattgtgagtagtgagtTTGTACATGTAGCATGATCCATACTACTCAGACTAATCATGCCATCAACTATTTG
TTGTACATAGGTTTGAGTACCCTACGAATTTTGAGTCTAGAAATTTTAACGTATGAAACAAAACACATTATTACA
ACCAAAATTTGAGTTTTGAATTTGTATTTTTTTATTTTTCATAACGTTAGACTCAAGAAGACTCTTTGACGTTTG
CATTACTCAAGCTTTTAAACATGGGTTGAAATGCTAGATCATCGGAACATTCGTCCTTGTTTACACTGTTTTCTC
CGCCACTGATCCAAAGCGTAGTGCCAGAGATTCTCACGTCGTATTGGCACCTCTTCCAATTGGATTTGCAGTTTT
CATGATTCATTTGGCCACTATTCCAATCACTGGAACTGGCATTAACCCGGCAAGGAGCCTTGGAGCTGCAGTAAT
CTACAACCAAGACAAGGCTTGGGATGATCTTGTTAGTATCTATATTCCTAATTATATTTAATTATATCCATTTAA
ATATACACTATACATAGAAATTTGATTTTTAACGATAAAAACTTGTCTCGAAAAGTCTCTTGATGACCTATGTTC
GGTCAATCGAACAATGACTTCTCATGGAAAATATTTTCATTGTAAAAATTGAATTTTCTAGTTTGCAATTTTTCT
AATACTTGCATTATATGTTTTCCAGTGGATTTTCTGGGTTGGTCCCTTTATTGGTGCAGCCATTGCAGCATTCTA
CCACCAGTTTATATTGAGAGCTGGGGCAGTGAAAGCCCTTGGTTCATTCAGGAGTAGTGCTTCTCATGTTTAA

>_Oeu_PIP2;11_Oeu017254.1/_Oeu_017256.1
ATGAAGGACGTAGGGGTGGCGAATCACGAGTCGTTCATGGCCAAGGACTACCATGATCCACCGCCGGCGCCGCTC
ATCGACACAGAGGAGCTGACGAAGTGGTCGTTTTACAGAGCTTTAATAGCTGAGTTCATAGCCACTTTACTATTT
CTTTACATTACAGTTTTGACTGTAATTGGGTATAAGAGCCATCTTGATACTGACTTGAAGGGTGACCAGTGTGGT
GGTGTGCGAATTCTTGGTATTGCATGGGCCTTTGGAGGCATGATCTTCATTCTTGTCTACTGCACTGCTGGAATT
TCTGGGGGGCATATAAACCCAGCAGTGACTTTTGGCCTGTTTTTGGCCCGAAAAGTGTCTTTGATCCGGGCAGTA
ATGTACATGGTGGCACAATGTTTGGGGGCCATTTGCGGTGTCGGTTTGGTCAAATCCTTTCAAAAATCATACTAC
AGGAAATACGGCGGTGGCTCCAATGAGCTGGCAGAAGGGTACAGCACCAGCGTCGGGCTATCTATCATCGGAACA
TTCGTCCTTGTTTACACTGTTTTCTCCGCCACTGATCCAAAGCGTAGTGCCAGAGATTCTCACGTCGTATTGGCA
CCTCTTCCAATTGGATTTGCAGTTTTCATGATTCATTTGGCCACTATTCCAATCACTGGAACTGGCATTAACCCG

GCAAGGAGCCTTGGAGCTGCAGTAATCTACAACCAAGACAAGGCTTGGGATGATCTTTGGATTTTCTGGGTTGGT
CCCTTTATTGGTGCAGCCATTGCAGCATTCTACCACCAGTTTATATTGAGAGCTGGGGCAGTGAAAGCCCCTTGGT
TCATTCAGGAGTAGTGCTTCTCATGTT**TAA**
>*Oeu*PIP2;11_*Oeu*017254.1/*Oeu*017256.1
MKDVGVANHESFMAKDYHDPPPAPLIDTEELTKWSFYRALIAEFIATLLFLYITVLTVIGYKSHLDTDLKGDQCG
GVRILGIAWAFGGMIFILVYCTAGISGGHINPAVTFGLFLARKVSLIRAVMYMVAQCLGAICGVGLVKSFQKSYY
RKYGGGSNELAEGYSTSVGLSIIGTFVLVYTVFSATDPKRSARDSHVVLAPLPIGFAVFMIHLATIPITGTGINP
ARSLGAAVIYNQDKAWDDLWIFWVGPFIGAAIAAFYHQFILRAGAVKALGSFRSSASHV*

*Oeu*PIP2-related, considered as Pseudogenes

*Oeu*059136.1 *Oeu*PIP2-related

>*Oeu*059136.1 | chr10:15785080..15785518 reverse
Incomplete sequence

>*Oeu*059136.1
ATGGTTCACCTTGTGACCATCCCCATTACTGGAACTAGCATCAAACCTACTAGGAGCTTTGGAGCTGCTATAATC
TACAACAAAGACAATCTCTGTGATGACCATATATGTCGACTTTTGCCTCTTTATATACATAATTAGTTCAAGTCT
TATCTTCACTTGTTTATGAAATTTAGACATGTGGAATATTTGATATAAAAAAAAATAAACAAGGGTTTTTTAAAT
AATAATTTAAATAATTTTATCAGCCATGTAATCGGTTCAACTTTTAAAAAGGGTTATTCAGACTAATATTTTTTA
CCACTTTTGTTTAAATAAATATGCAATGGATTCTCTAGGTTGGACCATTCATTGGAGTTGCTATTGTTGCATTCT
ACCACCAATACATTCTAAAATAGCAACCATTAAGGCATTGGGATCTTTCAGGAGCAATGCA**TAA**
>*Oeu*059136.1
ATGGTTCACCTTGTGACCATCCCCATTACTGGAACTAGCATCAAACCTACTAGGAGCTTTGGAGCTGCTATAATC
TACAACAAAGACAATCTCTGTGATGACCATATATGTGCAACCATTAAGGCATTGGGATCTTTCAGGAGCAATGCA
TAA
>*Oeu*059136.1
MVHLVTIPITGTSIKPTRSFGAAIIYNKDNLCDDHICATIKALGSFRSNA*

TIP – Tonoplastic Intrinsic Protein

*Oeu*TIP1;1 *Oeu*058531.1

>*Oeu*TIP1;1_*Oeu*058531.1 | chr22:3077808..3078742 forward
ATGCCTATTTCAAGAGTCGCTATTGGATCTCCGGCGGAGTTCAGCCAACTGGATGCCATCAAGGCTGCCGTGGCT
GAGTTCATTTCCATGTTGATTTTTGTTTTTGCTGGTCAAGGCTCTGGCATGGCTTTCGGTAAGTAGTTCTGCTGA
ACAAACTCATTTAAAAGTTTTAAGTTGTTAGAAATCGATAATTACATCATTGGTTTTTTATTTTTTCACTTTTTC
AACAGCTAAGCTTACGGACAATGAATCAACAACGCCGGCTGGCCTCATTGCTGCATCGATAGCACATGCTTTTGC
GCTTTTTGTGGCGGTTTCGGTCGGTGCCAACATCTCCGGCGGCCACGTAAACCCTGCTGTCACCTTAGGTGCATT
TGTTGGTGGTCACATCACATTGTTTAGAAGCATCATGTACTGGATTGCCCAATTGCTTGGATCTGTTATTGCATG
CTTGCTTCTTAAGTTTGCCACTGGTGGACTCGTAAGTTGAATCTTATATACTCTGTCCAGTTGAGTCCATTACTG
TAACATATTGGCTAATGTTCAATCCTTGATTGTAGGAAACATCAGCCTTTGCACTTTCATCGGGAGTGACAGTTT
GGAATGCAGTCATTTTCGAGATAGTGATGACCTTTGGACTTGTTTACACAGTTTATGCCACTGCAGTGGATCCAA
AGAAGGGTAATTTAGGGATCATTGCTCCCATTGCCATTGGCTTCATTGTTGGTGCCAACATCTTAGCCGGTGGCG
CATTCGATGGTGCATCCATGAACCCAGCCGTGTCTTTTGGCCCTGCTGTCGTCAGCTGGACATGGGACAGCCACT
GGGTCTACTGGCTCGGCCCATTTGTCGGTGCTGGAATTGCTGCTCTGGTCTATGAAATCTTGTTCATTAACCAGA
GCCATCAACAGCTCCCTACCAGTGCAGAATAC**TAA**
>*Oeu*TIP1;1_*Oeu*058531.1
ATGCCTATTTCAAGAGTCGCTATTGGATCTCCGGCGGAGTTCAGCCAACTGGATGCCATCAAGGCTGCCGTGGCT
GAGTTCATTTCCATGTTGATTTTTGTTTTTGCTGGTCAAGGCTCTGGCATGGCTTTCGCTAAGCTTACGGACAAT
GAATCAACAACGCCGGCTGGCCTCATTGCTGCATCGATAGCACATGCTTTTGCGCTTTTTGTGGCGGTTTCGGTC
GGTGCCAACATCTCCGGCGGCCACGTAAACCCTGCTGTCACCTTAGGTGCATTTGTTGGTGGTCACATCACATTG
TTTAGAAGCATCATGTACTGGATTGCCCAATTGCTTGGATCTGTTATTGCATGCTTGCTTCTTAAGTTTGCCACT
GGTGGACTCGAAACATCAGCCTTTGCACTTTCATCGGGAGTGACAGTTTGGAATGCAGTCATTTTCGAGATAGTG
ATGACCTTTGGACTTGTTTACACAGTTTATGCCACTGCAGTGGATCCAAAGAAGGGTAATTTAGGGATCATTGCT
CCCATTGCCATTGGCTTCATTGTTGGTGCCAACATCTTAGCCGGTGGCGCATTCGATGGTGCATCCATGAACCCA
GCCGTGTCTTTTGGCCCTGCTGTCGTCAGCTGGACATGGGACAGCCACTGGGTCTACTGGCTCGGCCCATTTGTC
GGTGCTGGAATTGCTGCTCTGGTCTATGAAATCTTGTTCATTAACCAGAGCCATCAACAGCTCCCTACCAGTGCA
GAATAC**TAA**
>*Oeu*TIP1;1_*Oeu*058531.1
MPISRVAIGSPAEFSQLDAIKAAVAEFISMLIFVFAGQGSGMAFAKLTDNESTTPAGLIAASIAHAFALFVAVSV
GANISGGHVNPAVTLGAFVGGHITLFRSIMYWIAQLLGSVIACLLLKFATGGLETSAFALSSGVTVWNAVIFEIV
MTFGLVYTVYATAVDPKKGNLGIIAPIAIGFIVGANILAGGAFDGASMNPAVSFGPAVVSWTWDSHWVYWLGPFV
GAGIAALVYEILFINQSHQQLPTSAEY*

*Oeu*TIP1;2 *Oeu*041014.1

>*Oeu*TIP1;2_*Oeu*041014.1 | chr20:11814751..11816218 reverse
ATGCCTTTCTCAAGAATTTCGGTCGGAAGTCCGGCGGAGGCCAGCCATCCAGATACCCTGAAAGCCGCCTTAGCC
GAATTCATTTCAATGGTAATTTTTGTATTTGCCGGTGAAGGCTCTGGCATGGCTTTTGGTAAGTACTTGTGCCCT
CATATTAGTTTTACAATTGTTCTTCCTTGACAAAATTATTTGCTTACTTCCTTGACAAAATGACACTTGGTATTC
AATCATTAGACTGTTTAAAAAACAGTTAGTTTTTCACCCAATCATCTGCTGCCAACGTTGTATCTATTAAGTAGG
TGGGCGGAAAATTATGTACAAATGGCTCAATCCTAAGTTCTAAACACTTGTCCTTTATTCTATTCAATCTCATAT
ATAATCATTTTTTCTACAGCCAAGCTTACAGACGGTGGATCGAGCACGCCGGCTGGCCTTATTGCTGCGGCGATA
GCCCATGCTTTCGCACTTTTTGTGGCAGTCTCGGTTGCTGCCAACATTTCCGGTGGTCATGTAAACCCTGCAGTC
ACCTTTGGTGCCTTTGTTGGTGGTCACCTATCATTGTTTAAAACTATTTTGTATTGGATTGCCCAATTGCTTGGA
GCTATTGTTGCATGCTTGCTCCTAAAGTTCTCCACCGGTGGCTTGGTAAGTAATTAATTCCTCAAAGCTATATTA
TTACACATAAAAGTTCTTCTTCATCATTTTTCTGAAATGTCGAAGAAGAACGAATGATGCGATTTCATTAGAATT
CTTGAAAAAAAATAAGGAAAATTCAAATTAATCTCTTCTACAGGAAACAAAAATAAAAGCAAAGACTATACCAAA
CGGACCCTTAGAATCCCTGGCCTAGAGCATGTTTGGCTGGCTAAGCTTCAAAAGAAGTAAAAGTTAATTTTCTTT
TCAAACATGTTAATAGAACTTATTTTCCTTAATTTTAAGTAAAAGAATTATTTATTTAAAAGAAAATTTTACGCA
TAAGAACCTTCCATGTTTTCAATCTCCAAGAATAAAAAATTGTCCATTACAGAACTTACTTATTTCCATGCTTTT
GTGTTTGACCTGAACTCAAATTTTGTCTTGTTACATCGTGCAGGAAACTTCCGCATTTGCACTTTCAACCGGGGT
ATCGTCGTGGAATGCCCTTGTTTTCGAAATAGTGATGACGTTTGGCCTGGTTTACACAGTTTATGCCACAGCAGT
AGATCCAAAGAGGGGTGATATAGGTATCATAGCCCCTATTGCCATTGGCTTCATTGTGGGTGCCAACATTTTGGC
TGGCGGTGCCTTCGACGGTGCATCCATGAACCCAGCCGTGTCGTTCGGCCCTGCAGTCGTCAGCTGGACATGGGA
CAGCCACTGGGTGTACTGGCTTGGCCCATTTGTTGGTGCTGGAATTGCTGCTATTGTCTATGAAATCGTCTTCAT
TAACCAGACCACTCATGAACCACTCCCCTCCAGTGAGTAC**TAA**

>*Oeu*TIP1;2_*Oeu*041014.1
ATGCCTTTCTCAAGAATTTCGGTCGGAAGTCCGGCGGAGGCCAGCCATCCAGATACCCTGAAAGCCGCCTTAGCC
GAATTCATTTCAATGGTAATTTTTGTATTTGCCGGTGAAGGCTCTGGCATGGCTTTTGCCAAGCTTACAGACGGT
GGATCGAGCACGCCGGCTGGCCTTATTGCTGCGGCGATAGCCCATGCTTTCGCACTTTTTGTGGCAGTCTCGGTT
GCTGCCAACATTTCCGGTGGTCATGTAAACCCTGCAGTCACCTTTGGTGCCTTTGTTGGTGGTCACCTATCATTG
TTTAAAACTATTTTGTATTGGATTGCCCAATTGCTTGGAGCTATTGTTGCATGCTTGCTCCTAAAGTTCTCCACC
GGTGGCTTGGAAACTTCCGCATTTGCACTTTCAACCGGGGTATCGTCGTGGAATGCCCTTGTTTTCGAAATAGTG
ATGACGTTTGGCCTGGTTTACACAGTTTATGCCACAGCAGTAGATCCAAAGAGGGGTGATATAGGTATCATAGCC
CCTATTGCCATTGGCTTCATTGTGGGTGCCAACATTTTGGCTGGCGGTGCCTTCGACGGTGCATCCATGAACCCA
GCCGTGTCGTTCGGCCCTGCAGTCGTCAGCTGGACATGGGACAGCCACTGGGTGTACTGGCTTGGCCCATTTGTT
GGTGCTGGAATTGCTGCTATTGTCTATGAAATCGTCTTCATTAACCAGACCACTCATGAACCACTCCCCTCCAGT
GAGTAC**TAA**
>*Oeu*TIP1;2_*Oeu*041014.1
MPFSRISVGSPAEASHPDTLKAALAEFISMVIFVFAGEGSGMAFAKLTDGGSSTPAGLIAAAIAHAFALFVAVSV
AANISGGHVNPAVTFGAFVGGHLSLFKTILYWIAQLLGAIVACLLLKFSTGGLETSAFALSTGVSSWNALVFEIV
MTFGLVYTVYATAVDPKRGDIGIIAPIAIGFIVGANILAGGAFDGASMNPAVSFGPAVVSWTWDSHWVYWLGPFV
GAGIAAIVYEIVFINQTTHEPLPSSEY*

***Oeu*TIP1;3 *Oeu*035479.1**

>*Oeu*TIP1;3_*Oeu*035479.1 | Scaffold3011:24603..25462 forward
ATGTCTAGCGAGTTCTTTAACGTGCCACTCCGACGGGTTGCCTTTGGACATCGAGATGAGTTCCGCCAGCCCGGT
GCTATCAAGGCGGCGCTAGCCGAGTTCTTCAGTACCCTGATTTTTGTGTTTGCTGGTGAGGGTTCCGGCATGGCT
TACAACAAATTGACTGGGAATGCTCCTACCTCCCCATCCGGCCTTGTGGCGGCAGCGGTGGCTCATGGTTTTGCT
TTGTTTGTAGCTGTTGCCGTCAGTGCTAACATTTCCGGCGGTCATGTAAACCCTGCCGTCACTTTTGGTTTGTTT
CTTGGTGGAAACGTCTCTCTCTTCCGTGGTATCCTTTATATAATTGCTCAGCTGCTGGGCTCTGTCGTTGCTTGC
CTTCTCCTCTTGTTCTCAACTGGTGGACTGGTAAGTTTTAGAAATAAACAAATTCTTGGTCCTCCCAAACAATAA
CAGCGGCTCCTAATTTATTGAATGAAACTTCGATGCAGGAAACATCTGCATTTGCTTTAACTGCCGGCGTGTCGG
TGTGGAACGCCTTTGTTTTCGAGATTGTAATGACCTTTGGCCTGGTTTACACTGTGTACGCCACCGCTGTTGACC
CAAAGAAGGGAGAAATTGGCATTATTGCACCAATTGCAATTGGTTTCATCGTGGGTGCCAATATTCTGGCGGGTG
GTGCATTTACCGGTGCTTCAATGAACCCTGCAGTGTCATTTGGTCCAGCTCTGGTCAGCTTCACCTGGACCCATC
ACTGGGTATACTGGGCTGGACCTCTTATCGGTGGTGGCCTTGCTGGTATTGTTTATGAAGTCTTCTTCGTCAACC
ATTCTCACGAACCCTTGCCAGGTTCAGAGTAC**TGA**
>*Oeu*TIP1;3_*Oeu*035479.1
ATGTCTAGCGAGTTCTTTAACGTGCCACTCCGACGGGTTGCCTTTGGACATCGAGATGAGTTCCGCCAGCCCGGT
GCTATCAAGGCGGCGCTAGCCGAGTTCTTCAGTACCCTGATTTTTGTGTTTGCTGGTGAGGGTTCCGGCATGGCT
TACAACAAATTGACTGGGAATGCTCCTACCTCCCCATCCGGCCTTGTGGCGGCAGCGGTGGCTCATGGTTTTGCT
TTGTTTGTAGCTGTTGCCGTCAGTGCTAACATTTCCGGCGGTCATGTAAACCCTGCCGTCACTTTTGGTTTGTTT
CTTGGTGGAAACGTCTCTCTCTTCCGTGGTATCCTTTATATAATTGCTCAGCTGCTGGGCTCTGTCGTTGCTTGC
CTTCTCCTCTTGTTCTCAACTGGTGGACTGGAAACATCTGCATTTGCTTTAACTGCCGGCGTGTCGGTGTGGAAC
GCCTTTGTTTTCGAGATTGTAATGACCTTTGGCCTGGTTTACACTGTGTACGCCACCGCTGTTGACCCAAAGAAG
GGAGAAATTGGCATTATTGCACCAATTGCAATTGGTTTCATCGTGGGTGCCAATATTCTGGCGGGTGGTGCATTT
ACCGGTGCTTCAATGAACCCTGCAGTGTCATTTGGTCCAGCTCTGGTCAGCTTCACCTGGACCCATCACTGGGTA
TACTGGGCTGGACCTCTTATCGGTGGTGGCCTTGCTGGTATTGTTTATGAAGTCTTCTTCGTCAACCATTCTCAC
GAACCCTTGCCAGGTTCAGAGTAC**TGA**
>*Oeu*TIP1;3_*Oeu*035479.1
MSSEFFNVPLRRVAFGHRDEFRQPGAIKAALAEFFSTLIFVFAGEGSGMAYNKLTGNAPTSPSGLVAAAVAHGFA
LFVAVAVSANISGGHVNPAVTFGLFLGGNVSLFRGILYIIAQLLGSVVACLLLLFSTGGLETSAFALTAGVSVWN
AFVFEIVMTFGLVYTVYATAVDPKKGEIGIIAPIAIGFIVGANILAGGAFTGASMNPAVSFGPALVSFTWTHHWV
YWAGPLIGGGLAGIVYEVFFVNHSHEPLPGSEY*

***Oeu*TIP1;4 *Oeu*040290.1**

>*Oeu*TIP1;4_*Oeu*040290.1 | chr4:16254381..16255311 reverse
ATGCCGATTTCGAGAATAGCTGTCGGATCTCTGGCCGAGGCTAGTCAGCCGGAAGCCCTCAAGGCCACCCTGGCC
GAGTTCATTTCGATGTTGATTTTTGTTTTTGCTGGTGAAGGTTCTGGCATGGCTTTCGGTAAGTAGTTATCCTGT
ACAAATTCATTTAAAAGTTTAAGTTGTTAGAATTCGAGAATTAAATCATTGGGATTTTGACTTTTTCCACAGCAA
AGCTTACAAACAATGGATCGACCACGCCAACTGGCCTAATTGCTGAGGCGATAGCGCACGCTTTTGCACTTTTCG
TGGCGGTTTCGGTCGGTGCTAACATCTCTGGCGGCCACGTAAACCCTGCTGTCACCTTTGGTGCCTTTGTTGGTG
GTCACATCACATTGATTAGGAGCATCCTGTACTGGATAGCCCAATTACTTGGATCTGTTGTTGCATGCTTGCTTC
TTAGGTTAGCCACAGGCGGACTCGTAAGTTGAATCTTACTGTACTATCCAGTCAAGTCGAGTCCATAACTATAAC

AAATAGGCTAAAGTTCAATTCTTGTTTACAGGAAACATCAGCTTTTGCACTTTCATCTGGAGTGACAGTTTGGAA
TGCAGTTGTTTTTGAGATAGTGATGACCTTTGGACTCGTTTACACAGTTTATGCTACTGCAGTGGATCCAAAGAG
GGGTAACTTAGGGATCATTGCCCCCATTGCCATTGGCTTCATTGTAGGTGCTAACATCTTAGCCGGTGGTGCCTT
CGATGGTGCATCCATGAACCCCGCCGTGTCTTTTGGCCCTGCTGTGGTCAGCTGGACATGGGACAACCACTGGGT
ATTCTGGCTCGGCCCATTTGTCGGTGCTGGAATTGCTGCTCTGATCTATGACATCTTGTTCATTAACCAAAGCCA
TGAACAACTCCGTACTAGTGCAGAATAC**TAA**

>*Oeu*TIP1;4_*Oeu*040290.1

ATGCCGATTTCGAGAATAGCTGTCGGATCTCTGGCCGAGGCTAGTCAGCCGGAAGCCCTCAAGGCCACCCTGGCC
GAGTTCATTTCGATGTTGATTTTTGTTTTTGCTGGTGAAGGTTCTGGCATGGCTTTCGCAAAGCTTACAAACAAT
GGATCGACCACGCCAACTGGCCTAATTGCTGAGGCGATAGCGCACGCTTTTGCACTTTTCGTGGCGGTTTCGGTC
GGTGCTAACATCTCTGGCGGCCACGTAAACCCTGCTGTCACCTTTGGTGCCTTTGTTGGTGGTCACATCACATTG
ATTAGGAGCATCCTGTACTGGATAGCCCAATTACTTGGATCTGTTGTTGCATGCTTGCTTCTTAGGTTAGCCACA
GGCGGACTCGAAACATCAGCTTTTGCACTTTCATCTGGAGTGACAGTTTGGAATGCAGTTGTTTTTGAGATAGTG
ATGACCTTTGGACTCGTTTACACAGTTTATGCTACTGCAGTGGATCCAAAGAGGGGTAACTTAGGGATCATTGCC
CCCATTGCCATTGGCTTCATTGTAGGTGCTAACATCTTAGCCGGTGGTGCCTTCGATGGTGCATCCATGAACCCC
GCCGTGTCTTTTGGCCCTGCTGTGGTCAGCTGGACATGGGACAACCACTGGGTATTCTGGCTCGGCCCATTTGTC
GGTGCTGGAATTGCTGCTCTGATCTATGACATCTTGTTCATTAACCAAAGCCATGAACAACTCCGTACTAGTGCA
GAATAC**TAA**

>*Oeu*TIP1;4_*Oeu*040290.1

MPISRIAVGSLAEASQPEALKATLAEFISMLIFVFAGEGSGMAFAKLTNNGSTTPTGLIAEAIAHAFALFVAVSV
GANISGGHVNPAVTFGAFVGGHITLIRSILYWIAQLLGSVVACLLLRLATGGLETSAFALSSGVTVWNAVVFEIV
MTFGLVYTVYATAVDPKRGNLGIIAPIAIGFIVGANILAGGAFDGASMNPAVSFGPAVVSWTWDNHWVFWLGPFV
GAGIAALIYDILFINQSHEQLRTSAEY*

***Oeu*TIP1;5 *Oeu*040289.1**

>*Oeu*TIP1;5_*Oeu*040289.1 | chr4:16257013..16257943 reverse

ATGCCGATTTCGAGAATAGCTGTCGGATCTCTGGCCGAGGCTAGTCAGCCGGAAGCCCTCAAGGCCACCCTGGCC
GAGTTCATTTCGATGTTGATTTTTGTTTTTGCTGGTGAAGGTTCTGGCATGGCTTTCGGTAAGTAGTTATCCTGT
ACAAATTCATTTAAAAGTTTAAGTTGTTAGAATTCGAGAATTAAATCATTGGGATTTTGACTTTTTCCACAGCAA
AGCTTACAAACAATGGATCGACCACGCCAACTGGCCTAATTGCTGAGGCGATAGCGCACGCTTTTGCACTTTTCG
TGGCGGTTTCGGTCGGTGCTAACATCTCTGGCGGCCACGTAAACCCTGCTGTCACCTTTGGTGCCTTTGTTGGTG
GTCACATCACATTGATTAGGAGCATCCTGTACTGGATAGCCCAATTACTTGGATCTGTTGTTGCATGCTTGCTTC
TTAGGTTAGCCACAGGCGGACTCGTAAGTTGAATCTTACTGTACTATCCAGTCAAGTCGAGTCCATAACTATAAC
AAATAGGCTAAAGTTCAATTCTTGTTTACAGGAAACATCAGCTTTTGCACTTTCATCTGGAGTGACAGTTTGGAA
TGCAGTTGTTTTTGAGATAGTGATGACCTTTGGACTCGTTTACACAGTTTATGCTACTGCAGTGGATCCAAAGAG
GGGTAACTTAGGGATCATTGCCCCCATTGCCATTGGCTTCATTGTAGGCGCTAACATCTTAGCCGGTGGTGCCTT
CGATGGTGCGTCCATGAACCCCGCCGTGTCTTTTGGCCCTGCTGTGGTCAGCTGGACATGGGACAGCCACTGGGT
CTTCTGGCTCGGCCCGTTTGTCGGTGCTGGAATTGCTGCTCTGATCTATGAAATCTTGTTCATTAACCAAAGCCA
TGAACAACTCCGTACTAGTGCAGAATAC**TAA**

>*Oeu*TIP1;5_*Oeu*040289.1

ATGCCGATTTCGAGAATAGCTGTCGGATCTCTGGCCGAGGCTAGTCAGCCGGAAGCCCTCAAGGCCACCCTGGCC
GAGTTCATTTCGATGTTGATTTTTGTTTTTGCTGGTGAAGGTTCTGGCATGGCTTTCGCAAAGCTTACAAACAAT
GGATCGACCACGCCAACTGGCCTAATTGCTGAGGCGATAGCGCACGCTTTTGCACTTTTCGTGGCGGTTTCGGTC
GGTGCTAACATCTCTGGCGGCCACGTAAACCCTGCTGTCACCTTTGGTGCCTTTGTTGGTGGTCACATCACATTG
ATTAGGAGCATCCTGTACTGGATAGCCCAATTACTTGGATCTGTTGTTGCATGCTTGCTTCTTAGGTTAGCCACA
GGCGGACTCGAAACATCAGCTTTTGCACTTTCATCTGGAGTGACAGTTTGGAATGCAGTTGTTTTTGAGATAGTG
ATGACCTTTGGACTCGTTTACACAGTTTATGCTACTGCAGTGGATCCAAAGAGGGGTAACTTAGGGATCATTGCC
CCCATTGCCATTGGCTTCATTGTAGGCGCTAACATCTTAGCCGGTGGTGCCTTCGATGGTGCGTCCATGAACCCC
GCCGTGTCTTTTGGCCCTGCTGTGGTCAGCTGGACATGGGACAGCCACTGGGTCTTCTGGCTCGGCCCGTTTGTC
GGTGCTGGAATTGCTGCTCTGATCTATGAAATCTTGTTCATTAACCAAAGCCATGAACAACTCCGTACTAGTGCA
GAATAC**TAA**

>*Oeu*TIP1;5_*Oeu*040289.1

MPISRIAVGSLAEASQPEALKATLAEFISMLIFVFAGEGSGMAFAKLTNNGSTTPTGLIAEAIAHAFALFVAVSV
GANISGGHVNPAVTFGAFVGGHITLIRSILYWIAQLLGSVVACLLLRLATGGLETSAFALSSGVTVWNAVVFEIV
MTFGLVYTVYATAVDPKRGNLGIIAPIAIGFIVGANILAGGAFDGASMNPAVSFGPAVVSWTWDSHWVFWLGPFV
GAGIAALIYEILFINQSHEQLRTSAEY*

*Oeu*TIP1;6 *Oeu*033244.1

>*Oeu*TIP1;6_*Oeu*033244.1 | Scaffold2789:55535..57322 forward
ATGCCGATTTATCGGATTGCGATTGGGACGCCTGGAGAAGCGAGCCACCCTGATGCACTCAAGGCCGCGCTTGCT
GAGTTCTTCTCTATGCTTATTTTTGTTTTTGCTGGTCAAGGCTCCGGGATGGCTTTTAGTACGTTCGCTCTTTCT
CCTCTACATCTATTTATATTCCGTTGATACGAAACAATGATCGAATTAACGGAGACTCTAGGAATCCCACTTTCT
TCATTTGATATTTGATGGGCTAGTGTGAGCTTATCGAATAATATTTTTTCTTAAAGATCCTGGCTTAATCTTCCC
TGGAAAGACACGTATGAGATCTTCAATTTTTGGGAGTTTTTCTATGCTATCATTTTTCAATCTTCTTGTTGCTGG
TATCTCATTCGTGTACATCATTTCTTTATTTTTCGATCTTCTAACGTAAAACGTTTGATGATTGTATCATATTAT
GGCAGATAAGTTGAGCAATGATGGGTCACCAAATCCATCAGGACTGCTAGCTGCAGCATTAGCCCATGCATTTGC
ACTTTTTGTGGCAGTCTCAGTAGGTGCAAACATATCAGGGGGTCATGTGAATCCTGCTGTCACGTTTGGTGCTTT
CATTGGTGGGAACATAACACTTTTGAGGAGCATTTTGTACTGGATTGCTCAATTACTTGGCTCTGTTGTTGCCTG
CATACTTCTTAAGTTTGCTACTGGAGGATTGGTAAGAAAGTTGATCGTTTTACGACAAATTTTGCTTCCAAACTA
CGAAAATTTTTGGTGTAAAAAATCATTGATTGTTTCGTAAAAATAGGGTCGTTGAGAGAACTTCTTTTCGTTGTT
AAATGTGAAATTTCTTGTAGTAGCTTAATTCAGTGCATTAATCAACAATGATTTGCTTCCATTACTGGAATTTTA
CAAATTTTAGTCTTTTCATTGTCTGAACTTAGAATATATGTAAGAATTTATACATGTTTGGAGAAAAAGGAGAAC
AAGATAGATGGCGATGCCCTCCCAAGTAAATCTAGACATACCTGAGTATTAGAGAATATACAATTCATGTCTAGA
TGGTTTGAAATTCAAACAAAAATAGAAATGGAAAATTTGGTAAAAATTTGAAGAGCCCATCTCATTTCCTCGTCT
TTTACTCGTGTAAGTAAATAATAAGGCTTTAGGGCATTTATACACTTTCTTAAACATAACATTACCTCCCCAATC
CAAGAGACAAGTTATCTTGAGGAGTATGCAACAAACCCTCAATACTTTCTATCATAAACTTGTAATAGATAATTA
AATATGTATTTATTGTAATTATTTATGCAAGTGAAAATAAATGTGGCAAAATATATTGAAGTACCAAGTAAAAAG
TTATACATTCTAATCTTTCCTTCCTCTCTTTTGACGTGGGACCTTGTTTATTTCAATTTTATGACAATCTACTTC
AACATGTACGTTCACGATTTTCGTGGGATGAAATCGACATATGAGCCAACTTATATATTGCAGGAAACATCAGCA
TTTGCCTTGTCGTCCGGTGTTTCTGTGTGGAACGCCCTAGTTTTTGAGATTGTGATGACCTTCGGGCTGGTGTAT
ACGGTGTATGCTACTGCAGTGGATCCAAAAAAGGGTAATTTGGGAAACATTGCACCCATTGCAATTGGGTTCATT
GTGGGTGCCAATATCTTAGCAGGTGGTCCATTTGATGGTGCATCAATGAACCCAGCAGTCTCCTTTGGACCAGCT
GTGGTCAGTTTGTCGGTTTTTGATCAGAAAGATCGAATTAGATTATCGATTAAAGAAAAATAA
>*Oeu*TIP1;6_*Oeu*033244.1
ATGCCGATTTATCGGATTGCGATTGGGACGCCTGGAGAAGCGAGCCACCCTGATGCACTCAAGGCCGCGCTTGCT
GAGTTCTTCTCTATGCTTATTTTTGTTTTTGCTGGTCAAGGCTCCGGGATGGCTTTTAATAAGTTGAGCAATGAT
GGGTCACCAAATCCATCAGGACTGCTAGCTGCAGCATTAGCCCATGCATTTGCACTTTTTGTGGCAGTCTCAGTA
GGTGCAAACATATCAGGGGGTCATGTGAATCCTGCTGTCACGTTTGGTGCTTTCATTGGTGGGAACATAACACTT
TTGAGGAGCATTTTGTACTGGATTGCTCAATTACTTGGCTCTGTTGTTGCCTGCATACTTCTTAAGTTTGCTACT
GGAGGATTGGAAACATCAGCATTTGCCTTGTCGTCCGGTGTTTCTGTGTGGAACGCCCTAGTTTTTGAGATTGTG
ATGACCTTCGGGCTGGTGTATACGGTGTATGCTACTGCAGTGGATCCAAAAAAGGGTAATTTGGGAAACATTGCA
CCCATTGCAATTGGGTTCATTGTGGGTGCCAATATCTTAGCAGGTGGTCCATTTGATGGTGCATCAATGAACCCA
GCAGTCTCCTTTGGACCAGCTGTGGTCAGTTTGTCGGTTTTTGATCAGAAAGATCGAATTAGATTATCGATTAAA
GAAAAATAA
>*Oeu*TIP1;6_*Oeu*033244.1
MPIYRIAIGTPGEASHPDALKAALAEFFSMLIFVFAGQGSGMAFNKLSNDGSPNPSGLLAAALAHAFALFVAVSV
GANISGGHVNPAVTFGAFIGGNITLLRSILYWIAQLLGSVVACILLKFATGGLETSAFALSSGVSVWNALVFEIV
MTFGLVYTVYATAVDPKKGNLGNIAPIAIGFIVGANILAGGPFDGASMNPAVSFGPAVVSLSVFDQKDRIRLSIK
EK*

*Oeu*TIP1;7 *Oeu*045237.1

***Observation*: 2059n were removed at the 5′ extremity**

>*Oeu*TIP1;7_*Oeu*045237.1 | chr11:5859698..5862731 reverse original version
ATGCCGGTCAGCAGGATAGCGGTGGGGAGGCTGGAAGAGGCGACTCAGCAGGACGCCTTGAAGGCGGCATTGGCT
GAGTTCATATCAACCCTAATATTTGTGTTTGCTGGCTCAGGTTCAGGCATGGCCTTCAACAAGCTAACAGACGAC
GGAGCCACCACTCCGGCAGGCCTCATTGCAGCTGCAGTGGCCCATGCATTTGCACTTTTCGTGGCTGTTTCCGTT
GGTGCCAACATTTCCGGTGGCCATGTTAACCCTGCTGTCACTTTTGGAGCCTCTAAAAAAGAAAATATTTTTCAT
CTTTTGTGGAAAATAAAAATCACCTCTACCCAAGGAAAATGTTTTCCTTGAAAACAAGAAAAATTAAGCATATTT
TCAAAAGTTGGAAAATATTTTTCATGAAAAAAAATAGCAATCAAATACTCTAAATAGGAATTTGGGTGGAAATTT
GATATTTTTCATGAAAAATAAATGTAATCAAACACACTCTTAAAGAGGATATACTGAGAATAATCAACTTCAATT
TTCGTTATCGCCGGCCTAGATTACATCGGCAATTATTTACAATAATAAATAAGACTCGATGATAAACTATATAAA
ATTTGAATTACTAGTGAAAATTTTACAATATACTGAATGAAACTTATCGAACATGCGTAGACAGCCCCAAGGAAA
AGGTTGCCTTAGACGTGGAATCACCCTAGCATTAGATCGGTTCAATCTAGCTCAATTAACCAAACACACCACGAC
TTTTTCCATTGTTAGTAGTCAATTTTGATCGATTCTTCAATTGTTTTTGGACATATTGCTCATTCTTAAAATCAA
TTATGATATAGTATATTAAGAGCCTGTTTGGGAGTAATTTTGAGAATTGATTTTTTGACCGTTAAAAAAAAAGTG
GGGTCCACCTGTAAGAGAAGAAGTCAAATATGACGACTAAAATAGTGAAATAACGGTCAAAAAATCAAATTTTTG

GATGAAAAAATCGCTCCCAAACTGACCCTAAGTAAAGAAAATACTTAACAAATTTATCACGCCACATATGTCATA
TTTATCTCATAACACGAGTGTGTAAAGTTCCATCCAGTCTTATCATATCTTAGAGTTATTCTTTCATCATCATTA
AATAATCGCAAATTATTATTCAATATAAAAACATTCACATTAAAAGTGTAAAAATTAAATTAATGTTGTTAGTTT
TTCATTTAGTTGCCCTCATGACACAGCCACTACACCACAAAATATTCTTTTGAAAATAAAATATCAATTAAGTTA
TAATATAATTCAAATACTTGAACTTATCTTATTGTTTGAGTTACATGTCTTTGTCTTATCAAAGATTCTGTTTAT
CTAAAGAATAATTCTATTTGTAGGTAGGATTAGCAAGTTCATAAGGTGCTCCCCATGATTTAAAAAGTTAAATTA
ATTTTTTTTATGTTAAAAATTCAAAATGTTAAATAGGCATTCAACGAGTCACGATGACTATTTCAAATTATACAA
ATTAATATTAAAATTAAAATTGTTGATCACAAATAATATTAGAATTTGTTAATGAATTTTTGAATCTAGCGTATT
CTACGTAGCGATTTTCCTCTCTATTTATTGGTGTTAGGACAAGCGATTTCATTAATTTTCTATTATATGTTTGAC
AATCATTAAAATAATTTTTTTTCTCAAACTCATTTTTATTCAGACATTCTTACAATGAAATACTTTGCATATAAA
TCTATAATTTTTTCATACTTTTTAAGTACATATTTTTCAAAAATTTATTATTTCAAAATTTATTTATTTTTTCAA
AAAATTTCATTTTAATAGTTGTGGCTAAAAGTAAAAGAAAAAAGAAATACACTTATATACGCAGTCGTGTCACTA
ATCTAATCCCTTTATCCATCCATCAATTGCTTAACTGTATGATTCTTGATTTCTTTTTGAATGATTGGACACAAA
ATATTTATTTAAATATACAAAAATAAACAAACACGTACTGTATTAGTATAATCCATTTTTAAACGGAAACTGGCT
ATATCAGGTAGCACATTCCCTTCAGCATCAGTATATGTACTCGAACAGCATGAGAAGTGGAAACCAGTAATATAC
TCACCAGAAAGATTCAAAGGAAGTGTGTTTCTGGTACCCCAGTTAATTTTTCAATTATTCGAAGATGCCGGTCAG
CAGGATAGCGGTGGGGAGGCTGGAAGAGGCGACTCAGCAGGACGCCTTGAAGGCGGCATTGGCTGAGTTCATATC
AACCCTAATATTTGTGTTTGCTGGCTCAGGTTCAGGCATGGCCTTCAACAAGCTAACAGACGACGGAGCCACCAC
TCCGGCAGGCCTCATTGCAGCTGCAGTGGCCCATGCATTTGCACTTTTCGTGGCTGTTTCCGTTGGTGCCAACAT
TTCCGGTGGCCATGTTAACCCTGCTGTCACTTTTGGAGCCTTTGTCGGTGGAAATATTAGCCTTATCCGTGGTAT
CCTCTACTGGATTGCTCAGTTGCTTGGTTCTGTCGTTGCTTGCTTGCTTCTCAAGTTCGCCACCGGTGGTTTGGT
GAGTTTTTCGCCCTCTTGCGTGAAAATTCTGTCTCTGTTTTACAATAATCACTTCGTCTAAAAGCTCAAGTTAAC
GAAAAACATTCACCCCATTTGTAATTTTATTTTTCAGACTACTGCAGCCTTTTCATTGTCAGCTGGAGTCAACGT
ATGGAATGCATTTGTATTAGAAATTGTGATGACCTTTGGGCTAGTCTACACCGTGTACGCCACCGCAGTGGATCC
AAAGAGGGGCAGTTTGGGAACAATTGCACCAATTGCTATTGGTTTCATTGTGGGTGCCAACATCTTAGCTGGTGG
GGCCTTTGATGGGGCCTCAATGAACCCAGCAGTGTCATTCGGCCCAGCTGTGGTGAGCTGGACCTGGGACAACCA
CTGGGTCTATTGGGCCGGGCCTCTAATTGGTGGTGGGCTTGCTGGGCTTATCTATGAGTTCTTCTTCATTAACCA
AACCCATGAACAGTTGCCTACAACTGATCAT**TAA**

>*Oeu*TIP1;7_*Oeu*045237.1 chr11:5861757..5862731 reverse Corrected version

ATGTACTCGAACAGCATGAGAAGTGGAAACCAGTAATATACTCACCAGAAAGATTCAAAGGAAGTGTGTTTCTGG
TACCCCAGTTAATTTTTCAATTATTCGAAGATGCCGGTCAGCAGGATAGCGGTGGGGAGGCTGGAAGAGGCGACT
CAGCAGGACGCCTTGAAGGCGGCATTGGCTGAGTTCATATCAACCCTAATATTTGTGTTTGCTGGCTCAGGTTCA
GGCATGGCCTTCAACAAGCTAACAGACGACGGAGCCACCACTCCGGCAGGCCTCATTGCAGCTGCAGTGGCCCAT
GCATTTGCACTTTTCGTGGCTGTTTCCGTTGGTGCCAACATTTCCGGTGGCCATGTTAACCCTGCTGTCACTTTT
GGAGCCTTTGTCGGTGGAAATATTAGCCTTATCCGTGGTATCCTCTACTGGATTGCTCAGTTGCTTGGTTCTGTC
GTTGCTTGCTTGCTTCTCAAGTTCGCCACCGGTGGTTTGGTGAGTTTTTCGCCCTCTTGCGTGAAAATTCTGTCT
CTGTTTTACAATAATCACTTCGTCTAAAAGCTCAAGTTAACGAAAAACATTCACCCCATTTGTAATTTTATTTTT
CAGACTACTGCAGCCTTTTCATTGTCAGCTGGAGTCAACGTATGGAATGCATTTGTATTAGAAATTGTGATGACC
TTTGGGCTAGTCTACACCGTGTACGCCACCGCAGTGGATCCAAAGAGGGGCAGTTTGGGAACAATTGCACCAATT
GCTATTGGTTTCATTGTGGGTGCCAACATCTTAGCTGGTGGGGCCTTTGATGGGGCCTCAATGAACCCAGCAGTG
TCATTCGGCCCAGCTGTGGTGAGCTGGACCTGGGACAACCACTGGGTCTATTGGGCCGGGCCTCTAATTGGTGGT
GGGCTTGCTGGGCTTATCTATGAGTTCTTCTTCATTAACCAAACCCATGAACAGTTGCCTACAACTGATCAT**TAA**

>*Oeu*TIP1;7_*Oeu*045237.1

ATGTACTCGAACAGCATGAGAAGTGGAAACCAGATAGCGGTGGGGAGGCTGGAAGAGGCGACTCAGCAGGACGCC
TTGAAGGCGGCATTGGCTGAGTTCATATCAACCCTAATATTTGTGTTTGCTGGCTCAGGTTCAGGCATGGCCTTC
AACAAGCTAACAGACGACGGAGCCACCACTCCGGCAGGCCTCATTGCAGCTGCAGTGGCCCATGCATTTGCACTT
TTCGTGGCTGTTTCCGTTGGTGCCAACATTTCCGGTGGCCATGTTAACCCTGCTGTCACTTTTGGAGCCTTTGTC
GGTGGAAATATTAGCCTTATCCGTGGTATCCTCTACTGGATTGCTCAGTTGCTTGGTTCTGTCGTTGCTTGCTTG
CTTCTCAAGTTCGCCACCGGTGGTTTGACTACTGCAGCCTTTTCATTGTCAGCTGGAGTCAACGTATGGAATGCA
TTTGTATTAGAAATTGTGATGACCTTTGGGCTAGTCTACACCGTGTACGCCACCGCAGTGGATCCAAAGAGGGGC
AGTTTGGGAACAATTGCACCAATTGCTATTGGTTTCATTGTGGGTGCCAACATCTTAGCTGGTGGGGCCTTTGAT
GGGGCCTCAATGAACCCAGCAGTGTCATTCGGCCCAGCTGTGGTGAGCTGGACCTGGGACAACCACTGGGTCTAT
TGGGCCGGGCCTCTAATTGGTGGTGGGCTTGCTGGGCTTATCTATGAGTTCTTCTTCATTAACCAAACCCATGAA
CAGTTGCCTACAACTGATCAT**TAA**

>*Oeu*TIP1;6_*Oeu*045237.1

MYSNSMRSGNQIAVGRLEEATQQDALKAALAEFISTLIFVFAGSGSGMAFNKLTDDGATTPAGLIAAAVAHAFAL
FVAVSVGANISGGHVNPAVTFGAFVGGNISLIRGILYWIAQLLGSVVACLLLKFATGGLTTAAFSLSAGVNVWNA
FVLEIVMTFGLVYTVYATAVDPKRGSLGTIAPIAIGFIVGANILAGGAFDGASMNPAVSFGPAVVSWTWDNHWVY
WAGPLIGGGLAGLIYEFFFINQTHEQLPTTDH*

***Oeu*TIP2;1 *Oeu*024074.1**

>*Oeu*TIP2;1_Oeu024074.1 | chr2:21805437..21806390 reverse
ATGCCTGCAATTGCTGTCGGAAGATTAGATGATTCATTTAGCATTGGTTCGTTGAAGGCCTATGTAGCTGAATTC
ATCTCCACCTTGCTCTTTGTTTTCGCCGGAGTCGGCTCCGCCATTGCCTACAGTTAGTTCTAAAACCGTTTTCTT
ATTTTTTTAAGTTAACAAGCACTTGGTAACATTTTGATCTTTGACAGACAAGTTGACGGCGGACGCAGCTCTTG
ATCCGGCGGGGCTGGTGGCTGTGGCCATCTGCCATGGTTTCGCTCTGTTTGTAGCCGTCTCGATCGGAGCCAACA
TCTCCGGTGGCCATGTAAACCCTGCAGTCACCTTTGGTTTGGCTGTTGGCGGCCAAATTACGCTACTTACTGGCC
TCTTTTACTGGATTGCTCAACTTTTGGGCTCCATTGTGGCATGCTATTTACTCGTATTCGTAACCGGAGGATTGG
TATGTCCACCCAAAATAATATTTTAAAATTCGAGTCGGTTTTTATTTACAGAGTTTTTATAGTCCGATTCTTGAT
TTCTCTGGCTAAACTACCCCGATTAGTTCGATTCCTAACAAAATATATAATTTCATGCAGGCTATTCCAATTCAT
GGAGTTGGTGCTGGTGTTGGAGCCTTTGAAGGAGTTGTTATGGAAATTGTTATCACATTTGCTTTAGTCTATACT
GTGTATGCGACAGCAGCTGATCCAAAGAAGGGCTCATTGGGCACAATTGCACCCATAGCCATTGGTTTCATTGTT
GGTGCCAATATTTTGGCTGCCGGTCCATTTTCAGGTGGGTCGATGAACCCTGCCCGCTCCTTCGGACCGGCTGTG
GCCAGCGGCAACTTCTCCGGCATCTGGATTTACTGGGTTGGACCCCTCATTGGTGGTGGCCTCGCCGGTCTCGTC
TACGCCTATGTGTTCATGTCCCACGAGCATGCTCCCCTACCTACCGACTTT**TAA**
>*Oeu*TIP2;1_Oeu024074.1
ATGCCTGCAATTGCTGTCGGAAGATTAGATGATTCATTTAGCATTGGTTCGTTGAAGGCCTATGTAGCTGAATTC
ATCTCCACCTTGCTCTTTGTTTTCGCCGGAGTCGGCTCCGCCATTGCCTACAACAAGTTGACGGCGGACGCAGCT
CTTGATCCGGCGGGGCTGGTGGCTGTGGCCATCTGCCATGGTTTCGCTCTGTTTGTAGCCGTCTCGATCGGAGCC
AACATCTCCGGTGGCCATGTAAACCCTGCAGTCACCTTTGGTTTGGCTGTTGGCGGCCAAATTACGCTACTTACT
GGCCTCTTTTACTGGATTGCTCAACTTTTGGGCTCCATTGTGGCATGCTATTTACTCGTATTCGTAACCGGAGGA
TTGGCTATTCCAATTCATGGAGTTGGTGCTGGTGTTGGAGCCTTTGAAGGAGTTGTTATGGAAATTGTTATCACA
TTTGCTTTAGTCTATACTGTGTATGCGACAGCAGCTGATCCAAAGAAGGGCTCATTGGGCACAATTGCACCCATA
GCCATTGGTTTCATTGTTGGTGCCAATATTTTGGCTGCCGGTCCATTTTCAGGTGGGTCGATGAACCCTGCCCGC
TCCTTCGGACCGGCTGTGGCCAGCGGCAACTTCTCCGGCATCTGGATTTACTGGGTTGGACCCCTCATTGGTGGT
GGCCTCGCCGGTCTCGTCTACGCCTATGTGTTCATGTCCCACGAGCATGCTCCCCTACCTACCGACTTT**TAA**
>*Oeu*TIP2;1_Oeu024074.1
MPAIAVGRLDDSFSIGSLKAYVAEFISTLLFVFAGVGSAIAYNKLTADAALDPAGLVAVAICHGFALFVAVSIGA
NISGGHVNPAVTFGLAVGGQITLLTGLFYWIAQLLGSIVACYLLVFVTGGLAIPIHGVGAGVGAFEGVVMEIVIT
FALVYTVYATAADPKKGSLGTIAPIAIGFIVGANILAAGPFSGGSMNPARSFGPAVASGNFSGIWIYWVGPLIGG
GLAGLVYAYVFMSHEHAPLPTDF*

***Oeu*TIP2;2 *Oeu*024076.1**

>*Oeu*TIP2;2_*Oeu*024076.1 | chr2:21795643..21796596 reverse
ATGCCTGCAATTGCTGTCGGAAGATTAGATGATTCATTTAGCATTGGTTCGTTGAAGGCCTATGTAGCTGAATTC
ATCTCCACCTTGCTCTTTGTTTTCGCCGGAGTCGGCTCCGCCATTGCCTACAGTTAGTTCTAAAACCGTTTTCTT
ATTTTTTTAAGTTAACAAGCACTTGGTAACATTTTGATCTTTGACAGACAAGTTGACGGCGGACGCAGCTCTTG
ATCCGGCGGGGCTGGTGGCTGTGGCCATCTGCCATGGTTTCGCTCTGTTTGTAGCCGTCTCGATCGGAGCCAACA
TCTCCGGTGGCCATGTAAACCCTGCAGTCACCTTTGGTTTGGCTGTTGGCGGCCAAATTACGCTACTTACTGGCC
TCTTTTACTGGATTGCTCAACTTTTGGGCTCCATTGTGGCATGCTATTTACTCGTATTCGTAACCGGAGGATTGG
TATGTCCACCCAAAATAATATTTTAAAATTCGAGTCGGTTTTTATTTACAGAGTTTTTATAGTCCGATTCTTGAT
TTCTCTGGCTAAACTACCCCGATTAGTTCGATTCCTAACAAAATATATAATTTCATGCAGGCTATTCCAATTCAT
GGAGTTGGTGCTGGTGTTGGAGCCTTTGAAGGAGTTGTTATGGAAATTGTTATCACATTTGCTTTAGTCTATACT
GTGTATGCGACAGCAGCTGATCCAAAGAAGGGCTCATTGGGCACAATTGCACCCATAGCCATTGGTTTCATTGTT
GGTGCCAATATTTTGGCTGCCGGTCCATTTTCAGGTGGGTCGATGAACCCTGCCCGCTCCTTCGGACCGGCTGTG
GCCAGCGGCAACTTCTCCGGCATCTGGATTTACTGGGTTGGACCCCTCATTGGTGGTGGCCTCGCCGGTCTCGTC
TACGCCTATGTGTTCATGTCCCACGAGCATGCTCCCCTACCTACCGACTTT**TAA**
>*Oeu*TIP2;2_*Oeu*024076.1
ATGCCTGCAATTGCTGTCGGAAGATTAGATGATTCATTTAGCATTGGTTCGTTGAAGGCCTATGTAGCTGAATTC
ATCTCCACCTTGCTCTTTGTTTTCGCCGGAGTCGGCTCCGCCATTGCCTACAACAAGTTGACGGCGGACGCAGCT
CTTGATCCGGCGGGGCTGGTGGCTGTGGCCATCTGCCATGGTTTCGCTCTGTTTGTAGCCGTCTCGATCGGAGCC
AACATCTCCGGTGGCCATGTAAACCCTGCAGTCACCTTTGGTTTGGCTGTTGGCGGCCAAATTACGCTACTTACT
GGCCTCTTTTACTGGATTGCTCAACTTTTGGGCTCCATTGTGGCATGCTATTTACTCGTATTCGTAACCGGAGGA
TTGGCTATTCCAATTCATGGAGTTGGTGCTGGTGTTGGAGCCTTTGAAGGAGTTGTTATGGAAATTGTTATCACA
TTTGCTTTAGTCTATACTGTGTATGCGACAGCAGCTGATCCAAAGAAGGGCTCATTGGGCACAATTGCACCCATA
GCCATTGGTTTCATTGTTGGTGCCAATATTTTGGCTGCCGGTCCATTTTCAGGTGGGTCGATGAACCCTGCCCGC
TCCTTCGGACCGGCTGTGGCCAGCGGCAACTTCTCCGGCATCTGGATTTACTGGGTTGGACCCCTCATTGGTGGT
GGCCTCGCCGGTCTCGTCTACGCCTATGTGTTCATGTCCCACGAGCATGCTCCCCTACCTACCGACTTT**TAA**

>*Oeu*TIP2;2_*Oeu*024076.1
MPAIAVGRLDDSFSIGSLKAYVAEFISTLLFVFAGVGSAIAYNKLTADAALDPAGLVAVAICHGFALFVAVSIGA
NISGGHVNPAVTFGLAVGGQITLLTGLFYWIAQLLGSIVACYLLVFVTGGLAIPIHGVGAGVGAFEGVVMEIVIT
FALVYTVYATAADPKKGSLGTIAPIAIGFIVGANILAAGPFSGGSMNPARSFGPAVASGNFSGIWIYWVGPLIGG
GLAGLVYAYVFMSHEHAPLPTDF*

*Oeu*TIP2;3 *Oeu*053988.1

>*Oeu*TIP2;3_*Oeu*053988.1 | chr22:5185229..5186864 forward
ATGCCAACCATTGCCTTCGGACGAATCGATGATTCATTAAGCGTTGGCTCTTTGAAGGCCTATTTAGCCGAATTC
ATCTCGACTTTGCTCTTCGTCTTCGCTGGAGTCGGCTCAGCCATTGCTTACAGTTAGTCATCACTTCTTTTTCCT
TTTAATAAAGTAAGATTTGATGCAGCACAATTCACACGAATCCCTCTCCCTGTACAAATCATTCAGAGGGAGGAA
AATACGCAGAATTCTTCGTTAGATCGGATCTATTAGACTCATTCTTTGGTGTTTCTCACTCTTTCACACACGCTA
GGTGTGAGAGATTGATGCATAAAATAAAATTAACCCTAGCCATGCAATGCAGCTTGTGATTATAAATGTTGGTTT
CGTATACAGTTCTAATTTGTGGGTATGTTTGATATTCTATTTCGACAGACAAGTTGACATATGATGCGGCTCTCG
ACCCAGCGGGGCTGGTAGCGGTGGCGGTCTGCCACGGATTTGCTCTGTTTGTGGCGGTTTCGATCGGAGCCAACA
TCTCCGGTGGCCATGTAAACCCTGCAGTCACCTTTGGTTTGGCTGTTGGTGGCCAAATTACAATTCTCACTGGCC
TCTTCTATTGGATTGCTCAACTTTTGGGCTCCATTGTAGCATGCTTCTTGCTCAAATTTGTCACAGGTGGCTTGG
TAAGTTGTTGTTCTATAATTATGTTATTCTTGAATTCTTTTCTAACTTCCTTCAAATGTGAGAGAGGGGATAAGA
TTATGTCTTGATACGTCCCCATCACGTACATTCCTTTTTTTTTTTTTTTTAATCAAAAGTTGACGCGTACACAAT
TTCCGTAAATAAATAATAAAGATTTTAAATCTCTGACTTTAAAGTTCTAATGCCATTTTGACTAAATCTAAAAGT
TCATATCTAATCTAAACATACATTCTCAAAAACTAAAACTGATTAATTGTACTCCTTTTCGACTTTCAACACAAA
GAATAGTAAATCTTCACCATGTGTAGTTCATATTCGTTTTGAATAAAAATGCTAACACGTGCACAATTTTCATAA
ATGAATGACGACAAAATTTGAACTTGGTTCCTCTATGTGTTCTCTACAATTTTGACTCGATCTAAAAGTTTACAA
CTGATCTAAGCTTACAGTACAAATGAGAATTGTATTCTTTTTCAACTTTCAACACGTTATATTAAAGAATAATCG
ATCTTGAATGATTTCATAAACAAGTGTCTAATTATGTTTGTAAATTTGATGATTAATTTCTATGTAGGCCATTCC
AACTCACAGTATGGCAGCTGGTGTTGGAGCCATTGAAGGAGTTGTGATGGAAATTGTTATCACTTTTGCTTTGGT
GTACACAGTTTATGCCACAGCAGTTGACCCAAAGAAGGGCTCATTAGGCACAATTGCCCCCATTGCCATTGGCTT
CATTGTTGGGGCCAACATTTTGGCCGCCGGCCCATTCTCCGGTGGCTCAATGAACCCGGCTAGGTCCTTCGGTCC
GGCTGTGGCCAGCGGTGACTTTGCCGACAACTGGATTTACTGGGTCGGTCCACTCATCGGTGGTGGTCTGGCTGG
ACTCGTCTATGCCTATGTGTTCATGGTCCACGAACACGTTCCTCTTGTTAGCGAGTTC**TAA**
>*Oeu*TIP2;3_*Oeu*053988.1
ATGCCAACCATTGCCTTCGGACGAATCGATGATTCATTAAGCGTTGGCTCTTTGAAGGCCTATTTAGCCGAATTC
ATCTCGACTTTGCTCTTCGTCTTCGCTGGAGTCGGCTCAGCCATTGCTTACAACAAGTTGACATATGATGCGGCT
CTCGACCCAGCGGGGCTGGTAGCGGTGGCGGTCTGCCACGGATTTGCTCTGTTTGTGGCGGTTTCGATCGGAGCC
AACATCTCCGGTGGCCATGTAAACCCTGCAGTCACCTTTGGTTTGGCTGTTGGTGGCCAAATTACAATTCTCACT
GGCCTCTTCTATTGGATTGCTCAACTTTTGGGCTCCATTGTAGCATGCTTCTTGCTCAAATTTGTCACAGGTGGC
TTGGCCATTCCAACTCACAGTATGGCAGCTGGTGTTGGAGCCATTGAAGGAGTTGTGATGGAAATTGTTATCACT
TTTGCTTTGGTGTACACAGTTTATGCCACAGCAGTTGACCCAAAGAAGGGCTCATTAGGCACAATTGCCCCCATT
GCCATTGGCTTCATTGTTGGGGCCAACATTTTGGCCGCCGGCCCATTCTCCGGTGGCTCAATGAACCCGGCTAGG
TCCTTCGGTCCGGCTGTGGCCAGCGGTGACTTTGCCGACAACTGGATTTACTGGGTCGGTCCACTCATCGGTGGT
GGTCTGGCTGGACTCGTCTATGCCTATGTGTTCATGGTCCACGAACACGTTCCTCTTGTTAGCGAGTTC**TAA**
>*Oeu*TIP2;3_*Oeu*053988.1
MPTIAFGRIDDSLSVGSLKAYLAEFISTLLFVFAGVGSAIAYNKLTYDAALDPAGLVAVAVCHGFALFVAVSIGA
NISGGHVNPAVTFGLAVGGQITILTGLFYWIAQLLGSIVACFLLKFVTGGLAIPTHSMAAGVGAIEGVVMEIVIT
FALVYTVYATAVDPKKGSLGTIAPIAIGFIVGANILAAGPFSGGSMNPARSFGPAVASGDFADNWIYWVGPLIGG
GLAGLVYAYVFMVHEHVPLVSEF*

*Oeu*TIP2;4 *Oeu*002329.1

>*Oeu*TIP2;4_*Oeu*002329.1 | chr13:8623502..8625525 reverse
ATGGTGAAGATGACATTGGGTAGCATTGGTGACTCTTTTTCAGTTCCGTCGCTAAAGGCTTATCTTGCTGAGTTC
ATTGCCACCCTCCTCTTTGTCTTTGCTGGTGTTGGTTCCGCTATAGCTTACAGTAAGTACATATTTTCTGTTTTC
ATAAAGTGTATATATTTTACGGTCGGCCCCCAAGCCCAGATATAGAATGAGGGTCGGGTTAGGTTAAGAGCAAGT
GTAAAACTTTATCAAAGCTTATGATATTAGTCGATTTCAAATTTATTGGAATTTGTGCAGACAAGCTCACGTCCG
ATGCAGCTCTCGACCCACCTGGATTGGTGGCAGTAGCGGTGGCTCACGCATTTGCATTGTTTGTGGGCGTTTCCA
TGGCGGCCAATGTATCCGGCGGTCATCTGAATCCGGCCGTCACATTTGGACTGGCCATCGGAGGCAACATCACAA
TCATAACTGGCATTTTCTATTGGATTGCCCAGTTGCTTGGTTCCACTGTTGCATGCCTTCTCCTCAAATTCGTCA
CTAATGGCTTGGTAAGTCATTCCCAATACCAAAATTTAATCTTATTTTGACAGTAAAGCAATAAAATTACCACCG
CAGGAGCTTTCTTAATGGCCTCCGGAGGTCATCTCGTGACCTTGGGGATTTCTGTGATTAAAAAAAAAAAAAAAG
CAAAAGAAAAAGAAAAGTTATTCATCCTTTGGATTAATTGAATATGCGAGCTTGTTCCATCGTTTGTGTTATACA

AAGTATCGAACCAAACATATCTCGAACTTAGAAGATTTACAGAAAACTTAAAGAAATTCACAACTTCCTTACAAG
CTTTCTCAAACCGCTACCATGGCATCAAGAATGAATATGTTTCAATGGACCCAGAAAACGAATTGTACCCGTCT
GTGTGACTAAAATCCTAACCCAGATCATCAAGCACACGTTTATAGATTAAAGGAAGCCATAAGGGTGGATGAGTT
CTCACCATCTCTTATCAGTACACAAAGTATCAAGATTACACAAAGAATCAGAAAGAAAATCAACGTCCAATAAAA
AACTGGAAGATGAATACAAAAGAGACAGGAAGAGTCACAAAGCATACACACGACGCAAACCCTAAGAATGAAAGA
ATAGGGCAAAAAATTATGTATAGGCTTTGAACCATTGATCAGATTCCAATCTAACGGTCTAGTTGGCTCCTCAAA
CAAAGAAGAAATCGTGTGAGTCTGTAACAATAAAAGTACACGCCTTAAAACACGACTTTAGTGCAATTGGACTCG
GGTAACTGGGTCACACCACATTATTGGGCAATTGTAGGTTTTAATAGAAAATGATTTTGGGCCTTAACCTTATCA
TCATTGCCCCAAGAGATAATTTGCCATCACAACCCAACAGTATATTTGTATAGGAATCTAATTTTTGTAAAAGAG
CATTTTGACAAAATTACTCTATTGTGCCAACTTTTCTAACTAGATATAGTACTTTCAGAAAATTATTTAAACATG
TTCAAAAACAGCTTGAAACAGTTTTTGATACTACATGCTCTTTAAAATTAACTTGAATTAGACTCCATCTAGGGT
GAATCAAGATGGGTTTTTTCGAGTTTTTCTAAATGTGCTAGGCCAAAACCATACTAATAGTTGGTGTTTGGCAGG
CCATTCCCACTCATGCAGTTGCTTCTGGAATGAATGCTGTGGAGGGTGTAGTAATGGAGATTGTCATCACTTTTG
CTCTAGTCTACACAGTCTATGCCACTGCAGCAGACCCCAAAAAGGGCTCATTGGGCATAATTGCACCCATTGCAA
TTGGGTTCATTGTTGGTGCAAACATTTTAGCTGCTGGCCCATTTAGTGGTGGATCAATGAACCCGGCCAGATCAT
TTGGGCCGGCCGTTGCTGCTGGAGACTTCTCTCAGAACTGGATCTATTGGGTTGGCCCACTTATTGGGGGAGGAT
TGGCAGGCCTGGTCTATGGTTATGTTTTTATTGCATCATATGCCCCATTCCCAGCCCAAGAGGGATATGCT**TAA**

>*Oeu*TIP2;4_*Oeu*002329.1

ATGGTGAAGATGACATTGGGTAGCATTGGTGACTCTTTTTCAGTTCCGTCGCTAAAGGCTTATCTTGCTGAGTTC
ATTGCCACCCTCCTCTTTGTCTTTGCTGGTGTTGGTTCCGCTATAGCTTACAACAAGCTCACGTCCGATGCAGCT
CTCGACCCACCTGGATTGGTGGCAGTAGCGGTGGCTCACGCATTTGCATTGTTTGTGGGCGTTTCCATGGCGGCC
AATGTATCCGGCGGTCATCTGAATCCGGCCGTCACATTTGGACTGGCCATCGGAGGCAACATCACAATCATAACT
GGCATTTTCTATTGGATTGCCCAGTTGCTTGGTTCCACTGTTGCATGCCTTCTCCTCAAATTCGTCACTAATGGC
TTGGCCATTCCCACTCATGCAGTTGCTTCTGGAATGAATGCTGTGGAGGGTGTAGTAATGGAGATTGTCATCACT
TTTGCTCTAGTCTACACAGTCTATGCCACTGCAGCAGACCCCAAAAAGGGCTCATTGGGCATAATTGCACCCATT
GCAATTGGGTTCATTGTTGGTGCAAACATTTTAGCTGCTGGCCCATTTAGTGGTGGATCAATGAACCCGGCCAGA
TCATTTGGGCCGGCCGTTGCTGCTGGAGACTTCTCTCAGAACTGGATCTATTGGGTTGGCCCACTTATTGGGGGA
GGATTGGCAGGCCTGGTCTATGGTTATGTTTTTATTGCATCATATGCCCCATTCCCAGCCCAAGAGGGATATGCT
TAA

>*Oeu*TIP2;4_*Oeu*002329.1

MVKMTLGSIGDSFSVPSLKAYLAEFIATLLFVFAGVGSAIAYNKLTSDAALDPPGLVAVAVAHAFALFVGVSMAA
NVSGGHLNPAVTFGLAIGGNITIITGIFYWIAQLLGSTVACLLLKFVTNGLAIPTHAVASGMNAVEGVVMEIVIT
FALVYTVYATAADPKKGSLGIIAPIAIGFIVGANILAAGPFSGGSMNPARSFGPAVAAGDFSQNWIYWVGPLIGG
GLAGLVYGYVFIASYAPFPAQEGYA*

***Oeu*TIP2;5 *Oeu*040207.1**

Observation: Intron 3 converted in exon

>*Oeu*TIP2;5_*Oeu*040207.1 | chr19:9881246..9883023 forward original version

ATGGTGAAGATTAATTGGGGTAGCATTGGCGACTCTTTTTCGGTTACGTCGCTAAAGGCTTACATTGCTGAGTTC
ATTGCTACCCTTCTCTTTGTTTTTGCTGGTGTTGGTTCTGCTATAGCTTATAGTAAGTCATCTTTTATGTTTTGC
TAGAGTATATACACAAGTAATTCCGTCTATATTTAGTCTCTATCTGGTGGGGATTTTTAGTAGATTTTGATAAAT
GTTGATAAAATATGTGGTTGAAAAGTGATGAGTGTTATAAAATGTGTTGTTAAAATTAAATGTTGATTTTAGTTT
AGATTAAAATTTGAATTTCAATTTTCATCGTACAGGCGCTCAGATAGTTGACCATAAGCCCGAATAAAGGAGGAT
CGTTGCAATAGATTAACCATAAGAATAAAAAGGTTTAGGCTTAGGTGTACTAATATATGGAGTATGATTGATGTT
TGCAGACAAGCTCACGTCTGATGCAGCTCTCGACCCACCCGGTTTGGTGGCAGTCGCGGTGGCTCACGCATTTGC
ATTGTTCGTAGGCGTTTCCATGGCGGCCAATGTGTCCGGTGGTCATTTGAATCCGGCTGTCACATTCGGATTGGC
CATCGGAGGCAACATCACCATCATAACTGGCCTTTTCTATTGGATTGCACAATTGCTTGGTTCTATTGTTGCATG
CCTTCTCCTCAAATTTGTCACCGGTGGCTTGGTAAGTTATACCAAGATTAAAATTAAACTTATTTTGAGGGTAAA
GCACTAAAATTGCTCATTTTGAGATCTTTAGAGCCTTTCGCGATAAGAAAAATACTCATCTTTTGGATTAACTGC
ATGTTGAAGCTTATTTTTAATTATTTTCTGATTGATATAAGATAGAAAACAGCTTGTCTACTCTGATGTTGGACC
AAAACAAGTCATAATTTATGTAAAATCGACTTCTAAATTGAAACGTCAACTCAATTCATTATAGGTTCGTTAAGC
TTTAATAACTGAGGTCACAAGAAATTACTCAAATTGTTTGAGTAGTTCGTGTTCGACTAGACAAAAACCTTACAA
AACATCACCTGATACATTAAAGACTGGAGTTCAAGTATTTCATTGAATTAGCATTTAAATTCTACTCGAATTTGA
GTAGTTTTACCCTTTGTTTGAAACCCCATTAAATTAAAATTCATTCTCTCTCTCTTTTTATCAAGCATACATTTT
TCAATAAAACAATATTACACGTAAACTTAAATACTTTTCCTTTCTATTTAACCACACATTCTTCAAAAAATTACT
ATTCTTTTCATTATAATTCGAAATAATGGATCCCAAACAAGGGCCTAGACTATCGCTAGATTGAATCAAGATGGG
CTTTTGCCTGAATGTGGTAAGCCCAAACCATACTAATATTTGATGTTTGGCAGGCCATTCCCACTCATGCAGTTG
CTTCTGGTATGAATGCTGTGGAGGGTGTTGTAATGGAGATTGTCATCACTTTTGCTTTGGTCTACACAGTCTATG
CCACTGCTGCGGACCCCAAAAAAGGCTCGTTGGGCACAATTGCACCCATTGCAATTGGGTTCATTGTGGGTGCAA
ACATTTTAGCTGCTGGCCCATTTAGTGGTGGATCGATGAACCCGGCCCGATCATTTGGGCCAGCCGTTGCCGCAG

TCGACTTCTCTCAGAACTGGATCTATTGGGTCGGCCCACTTATTGGTGGAGGATTGGCTGGCCTGGTATATGGTG
ATGTTTTTATTGGATCATATGCCCCACTTCCAGTCCAAGAAGAATATCCT**TAA**

>*Oeu*TIP2;5_*Oeu*040207.1 Corrected version

ATGGTGAAGATTAATTGGGGTAGCATTGGCGACTCTTTTTCGGTTACGTCGCTAAAGGCTTACATTGCTGAGTTC
ATTGCTACCCTTCTCTTTGTTTTTGCTGGTGTTGGTTCTGCTATAGCTTATAGTAAGTCATCTTTTATGTTTTGC
TAGAGTATATACACAAGTAATTCCGTCTATATTTAGTCTCTATCTGGTGGGGATTTTTAGTAGATTTTGATAAAT
GTTGATAAAATATGTGGTTGAAAAGTGATGAGTGTTATAAAATGTGTTGTTAAAATTAAATGTTGATTTTAGTTT
AGATTAAAATTTGAATTTCAATTTTCATCGTACAGGCGCTCAGATAGTTGACCATAAGCCCGAATAAAGGAGGAT
CGTTGCAATAGATTAACCATAAGAATAAAAAGGTTTAGGCTTAGGTGTACTAATATATGGAGTATGATTGATGTT
TGCAGACAAGCTCACGTCTGATGCAGCTCTCGACCCACCCGGTTTGGTGGCAGTCGCGGTGGCTCACGCATTTGC
ATTGTTCGTAGGCGTTTCCATGGCGGCCAATGTGTCCGGTGGTCATTTGAATCCGGCTGTCACATTCGGATTGGC
CATCGGAGGCAACATCACCATCATAACTGGCCTTTTCTATTGGATTGCACAATTGCTTGGTTCTATTGTTGCATG
CCTTCTCCTCAAATTTGTCACCGGTGGCTTGGTAAGTTATACCAAGATTAAAATTAAACTTATTTTGAGGGTAAA
GCACTAAAATTGCTCATTTTGAGATCTTTAGAGCCTTTCGCGATAAGAAAAATACTCATCTTTTGGATTAACTGC
ATGTTGAAGCTTATTTTTAATTATTTTCTGATTGATATAAGATAGAAAACAGCTTGTCTACTCTGATGTTGGACC
AAAACAAGTCATAATTTATGTAAAATCGACTTCTAAATTGAAACGTCAACTCAATTCATTATAGGTTCGTTAAGC
TTTAATAACTGAGGTCACAAGAAATTACTCAAATTGTTTGAGTAGTTCGTGTTCGACTAGACAAAAACCTTACAA
AACATCACCTGATACATTAAAGACTGGAGTTCAAGTATTTCATTGAATTAGCATTTAAATTCTACTCGAATTTGA
GTAGTTTTACCCTTTGTTTGAAACCCCATTAAATTAAAATTCATTCTCTCTCTCTTTTTATCAAGCATACATTTT
TCAATAAAACAATATTACACGTAAACTTAAATACTTTTCCTTTCTATTTAACCACACATTCTTCAAAAAATTACT
ATTCTTTTCATTATAATTCGAAATAATGGATCCCAAACAAGGGCCTAGACTATCGCTAGATTGAATCAAGATGGG
CTTTTGCCTGAATGTGGTAAGCCCAAACCATACTAATATTTGATGTTTGGCAGGCCATTCCCACTCATGCAGTTG
CTTCTGGTATGAATGCTGTGGAGGGTGTTGTAATGGAGATTGTCATCACTTTTGCTTTGGTCTACACAGTCTATG
CCACTGCTGCGGACCCCAAAAAAGGCTCGTTGGGCACAATTGCACCCATTGCAATTGGGTTCATTGTGGGTGCAA
ACATTTTAGCTGCTGGCCCATTTAGTGGTGGATCGATGAACCCGGCCCGATCATTTGGGCCAGCCGTTGCCGCAG
TCGACTTCTCTCAGAACTGGATCTATTGGGTCGGCCCACTTATTGGTGGAGGATTGGCTGGCCTGGTATATGGTG
ATGTTTTTATTGGATCATATGCCCCACTTCCAGTCCAAGAAGAATATCCT**TAA**

>*Oeu*TIP2;5_*Oeu*040207.1

ATGGTGAAGATTAATTGGGGTAGCATTGGCGACTCTTTTTCGGTTACGTCGCTAAAGGCTTACATTGCTGAGTTC
ATTGCTACCCTTCTCTTTGTTTTTGCTGGTGTTGGTTCTGCTATAGCTTATAACAAGCTCACGTCTGATGCAGCT
CTCGACCCACCCGGTTTGGTGGCAGTCGCGGTGGCTCACGCATTTGCATTGTTCGTAGGCGTTTCCATGGCGGCC
AATGTGTCCGGTGGTCATTTGAATCCGGCTGTCACATTCGGATTGGCCATCGGAGGCAACATCACCATCATAACT
GGCCTTTTCTATTGGATTGCACAATTGCTTGGTTCTATTGTTGCATGCCTTCTCCTCAAATTTGTCACCGGTGGC
TTGGCCATTCCCACTCATGCAGTTGCTTCTGGTATGAATGCTGTGGAGGGTGTTGTAATGGAGATTGTCATCACT
TTTGCTTTGGTCTACACAGTCTATGCCACTGCTGCGGACCCCAAAAAAGGCTCGTTGGGCACAATTGCACCCATT
GCAATTGGGTTCATTGTGGGTGCAAACATTTTAGCTGCTGGCCCATTTAGTGGTGGATCGATGAACCCGGCCCGA
TCATTTGGGCCAGCCGTTGCCGCAGTCGACTTCTCTCAGAACTGGATCTATTGGGTCGGCCCACTTATTGGTGGA
GGATTGGCTGGCCTGGTATATGGTGATGTTTTTATTGGATCATATGCCCCACTTCCAGTCCAAGAAGAATATCCT
TAA

>*Oeu*TIP2;5_*Oeu*040207.1

MVKINWGSIGDSFSVTSLKAYIAEFIATLLFVFAGVGSAIAYNKLTSDAALDPPGLVAVAVAHAFALFVGVSMAA
NVSGGHLNPAVTFGLAIGGNITIITGLFYWIAQLLGSIVACLLLKFVTGGLAIPTHAVASGMNAVEGVVMEIVIT
FALVYTVYATAADPKKGSLGTIAPIAIGFIVGANILAAGPFSGGSMNPARSFGPAVAAVDFSQNWIYWVGPLIGG
GLAGLVYGDVFIGSYAPLPVQEEYP*

*Oeu*TIP3;1 *Oeu*038387.1

>*Oeu*TIP3;1_*Oeu*038387.1 | chr10:29024107..29025120 forward

ATGCCTCGTGGGTATACTTTTGGTAGGGCTGAAGAAGCCACCCACCCCGACTCTGCCAGGGCCACCTTGGCTGAG
TTTCTCTCCATGCTCGTCTTTGTCTTCGCCGGCGAAGGCTCCGTCCTTGCTCTTGGTATCTTAATTCTTCAGTAA
CGTTCTTGAAAATAAGAAGAAGTAGGTGCACATGGACGTACAGCTCATATTATTCTCAATTTGTATCGATTTGCA
GATAAGATGTATAAGGATACAGCCTTAGGAGCATCAAGCCTGGTGGTCATCGCTCTCGCCCACGCCCTGTCACTG
TTTGCAGCTGTGGCTTCGAGTATGAATGTTTCCGGCGGGCACGTCAATCCTGCCGTCACTTTCGGCGCACTTGTA
GGGGGAAGAGTCTCTGTGCTGCGTGCTATTTACTACTGGATTGCTCAACTTTTGGGTGCAGTTTTAGCTGCTATC
TTGTTGAGGCTGGCAACTGATGGCTTGGTAAAAAGAAATAGAAAAACCCACCCGCCTTCCACCTTAATTAATTAC
TTGCATGTCTCAGCACATGTTTTGGTGTGTTTATAAGTGCTTATAAGTACCGGGACTTTCTACTCGTTTTTAATA
AGCATCCTGTGTTTGCAGAGGCCGCGAGGGTTTTCCGTGGCGGATGGAGTAGGAGAATGGAACGCTTTGGTGATG
GAGATTGTGCTGACATTTGGACTGGTATATACAGTGTACGCGACTGCAATCGATCCAAAAAGAGGAAGCTTGGGC
ACCATTGCTCCTCTTGCCATTGCCTTCATAGTTGGGGCTAATGTGCTGGTTGGAGGGCCTTTCTCCGGAGCATCG
ATGAACCCTGCTCGTGCGTTCGGGCCAGCACTTGTGGGATGGAGGTGGAAAGACCACTGGGTCTACTGGTTGGGA

CCATTCGTTGGTGCGGCTCTGGCTGGAGTTATATACGAGTTCGGGGTGATACAAACCGAGACCCCGCACCCCACG
CATCACCTTCACCAGCCTTTGGCCCCAGAAGATTAC**TAG**
>*Oeu*TIP3;1_*Oeu*038387.1
ATGCCTCGTGGGTATACTTTTGGTAGGGCTGAAGAAGCCACCCACCCCGACTCTGCCAGGGCCACCTTGGCTGAG
TTTCTCTCCATGCTCGTCTTTGTCTTCGCCGGCGAAGGCTCCGTCCTTGCTCTTGATAAGATGTATAAGGATACA
GCCTTAGGAGCATCAAGCCTGGTGGTCATCGCTCTCGCCCACGCCCTGTCACTGTTTGCAGCTGTGGCTTCGAGT
ATGAATGTTTCCGGCGGGCACGTCAATCCTGCCGTCACTTTCGGCGCACTTGTAGGGGGAAGAGTCTCTGTGCTG
CGTGCTATTTACTACTGGATTGCTCAACTTTTGGGTGCAGTTTTAGCTGCTATCTTGTTGAGGCTGGCAACTGAT
GGCTTGAGGCCGCGAGGGTTTTCCGTGGCGGATGGAGTAGGAGAATGGAACGCTTTGGTGATGGAGATTGTGCTG
ACATTTGGACTGGTATATACAGTGTACGCGACTGCAATCGATCCAAAAAGAGGAAGCTTGGGCACCATTGCTCCT
CTTGCCATTGCCTTCATAGTTGGGGCTAATGTGCTGGTTGGAGGGCCTTTCTCCGGAGCATCGATGAACCCTGCT
CGTGCGTTCGGGCCAGCACTTGTGGGATGGAGGTGGAAAGACCACTGGGTCTACTGGTTGGGACCATTCGTTGGT
GCGGCTCTGGCTGGAGTTATATACGAGTTCGGGGTGATACAAACCGAGACCCCGCACCCCACGCATCACCTTCAC
CAGCCTTTGGCCCCAGAAGATTAC**TAG**
>*Oeu*TIP3;1_*Oeu*038387.1
MPRGYTFGRAEEATHPDSARATLAEFLSMLVFVFAGEGSVLALDKMYKDTALGASSLVVIALAHALSLFAAVASS
MNVSGGHVNPAVTFGALVGGRVSVLRAIYYWIAQLLGAVLAAILLRLATDGLRPRGFSVADGVGEWNALVMEIVL
TFGLVYTVYATAIDPKRGSLGTIAPLAIAFIVGANVLVGGPFSGASMNPARAFGPALVGWRWKDHWVYWLGPFVG
AALAGVIYEFGVIQTETPHPTHHLHQPLAPEDY*

*Oeu*TIP4;1 *Oeu*030229.1/*Oeu*036364.1

Observation: *Oeu*tip4;1 was generated after combination of 2 scaffolds :
Oeu030229.1 | scaffold25131:282..785 reverse
Oeu036364.1 | scaffold311:586778..587098 forward

>OeuTIP4;1 *Oeu*030229.1/*Oeu*036364.1
ATGGCTAAGATTGCCTTGGGAAATGCCCGAGAGGCCTTTCAATCGGACTGCATCCAGGCCCTTATTGTGGAATTC
ATTTGCACGTTTCTTTTCGTGTTCGCCGGGGTCGGCGCTGCCATGGCTACCGGTAATGTCTTGAGTTTCCTCATT
TAAATTCTATTTTCAACTACATGTCTTCGATATGGTAACCTTTTAACAGATAAGCTGAATGGGGATCCGTTGGTG
GGACTATTTTTTGTGGCTATGGCACATGCGTTGGTGGTGGCGGTGATGATTTCAGCCGGATTCCGCGTTTCCGGT
GGCCACATAAACCCTGCTGTCACCCTCGGCCTATTAGCAGGCGGCCATATCACTGTCGTCAGATCAATCCTCTAC
TGGATTGATCAGTTATTAGCTTCTACAGCAGCTTGTGCTCTGCTCAAGTACCTAACTGGAGGATTGGTTAGTATT
AGTATATCTTTGCCTTATACGAATGCACTCAGTTTTGTCTTTAAAGACGCATAATCCTAAAAAAAATTGAAATCT
CGTTTCATTCACTTCGGCTTCCCATCTTAATGATCTCGGTTAGTCAAGTAAAGTGACGTAGAGAGCGACTTCTAA
ATGGTTGGCCCAAGTGGTGGAATGTGACCCGGTTTGGCCCCTTGTGCTCTATCTCGCATTTACTTAATACACAAC
TTATTGAGCGACTGAGATGATGACACATGTTGCTAGTTGAACTGTTGGATGCATTATGACCTTTCATATTCTAGT
CTCTCTCCTTTTTTTGGGTTCAAGATTATTGTAGGTTTCCCGACTCTATTTGTCTTTATGATTTCTCTCTCTAAA
AACCCTTGATTTTCATTTGTAATCTTGAATCTTATTGTAATCTCGGTGGTGTCCAATATAAATCATGTTATGGTT
AGAATTTTATTTTTCTTGGGTGAACTGCAACTCTTGGTTACGTGGTGTAATCTGTAAACGAGAGAAAAAGTGAGA
GTTGAGTCTGAGGGCTTTAAGTCGAGTGATTTTTGAGTATCTGATTCTTGTATTTTGACACAATTTGTTGGATTC
TAGAGGATTAGAACGTTAACCACTTAGGTGGATGCTCAACGTGGTGAGTAGCCGCCCATTCTTGGGTGAACATCT
GGGCCTACGAGCGAAAAATCTTTATGTTCTCTGTGTTTGATTTACTTGAATAAATTTTGGGATTGTTAGATACCA
ACAGTGGTATCAGAGCTGCTCAATCTGTGTTGGAAGCCTTCGGTCTGAATATTTCTGGTGCTCTTGGTGACCAAA
ACTCTAGATCTTCACAGATTTGTATTTTTAAGGGCTTGATTTTCATTAATTTCTAAGAAAGTAAAATAGTTTTGA
TATTCACTATTTAGTGTCCTAAGACGTGTTGGTAACGAGTTTCTAAACCCTATTTCCTTCTTTAAAACTTGCATA
TCTTGGTTGAAACTCACCCTGCTCTTTCTTTGTCTAGGGTTTCATGCGACCTAGAATCTGAGGGAACGATGTTTA
ACGGTTTCTTTCAAGATTATTGTTTATCCGTGATTCTGATATTTGTACTTTATGATTGGGTTATTTGTTGTGATC
TGCCTTCTCTGATTTGCATATTGGTGTATTTGCGTGGACACTATTGCACATTATCACTTTGTGCGTATATCTCTG
TGAATAAATTGTGTCTTAGTTTTGTGTACTTTAGACTTTATACCGAAATTTTTTAAAAATCATGGGAAGTACCAA
AATTGATATTGAGATATGGTATGGCAATGAAGACTTTAGTCTCTGGAGTAAAAAAATGAAGGCCCTTTTAGTACA
ACAAAGATGTGTGAAAGCTATAGATGAATCATGGGGTACAAAAGTTACTGAAAATAGGAGGACTGATCAAGATAA
AATTGCATGGAGTACAATTTTCTTGCATTTATTTGAAAGTGTGATTAGAGAGGTAGAAAAAACTAAGACTGCAAT
AGAAATCTAGACAAAGCATGATTTTCTTTATCAAACTAAGACCCTTCCCAACAAATGTTACTTGTGGAAACAATT
TTTCTGTTTCAAATTTGACCCTAATTGTGATCTTGAAGTTAATCCTGATAGATTTAATAAACTCTTTCAAGATAT
CACTAATGGTGGTAAGACTTTATCTGATGATAAAAAATCTGTGGCCCTACTGTCATTTTTACCTGAGAAATTTAA
AGAAGTGAAACATCCCCTTGAATATGAAAGAACTACTGTCACAGTTAATGATATTATGACTTCTTTAAGGAATAA
GGAACATGAAGTAAAGAGTGATTCAAAAACTCCATAACTGGAGTAGGTTACAATATAAAAGGTACTAATTCCTTC
AAGAAGAAGAATAATCAATATATGAATAAAAAAATTTGGGAAACCAAAAGAATATTATAGTACATCTCAAAAGGGT
AAGTCAAAATTTAATTAGAACAACAGTAAGGCTAAAGATAGGAAGTGTTACTTTTGTGGAAAAATGGGCCACTAC

ATCCAAGATTGTATGAAAAGAATTGAGGAACTGCAAAATGCAAATAAGGTTGAAGGTGAAGCTGCTATAGCTTAT
GAAGAAACAACAACCTCAAATGGACAAGTTTATCTGACTTCTCAACCCATAGTCAAAGATGACTGGATACTAGAC
TCTGGATATACTTTCCATATGCCACCCTAATAGAGCTCTTCACTGAATTTTATGAAACAAGTGGAGGACAAGTAA
CTATGAGAAATGATTTTTCGTGCAAGATTAAAGGAATTGGAAAGATTGTTTTAAAACTAGACAATGGGAATGTAC
TAACCTCAAACAAAACTAGATACACTCCTAACTTAAAGAGAAACTTAATCTCTTTGGGCACACTTGATGATGAAG
GTTTTAATACAATCATAAATAAAAGTGTTTTGACACTCCAAAATAATCTTGCTAATATCATTTAAGTAGAAAAAT
CAAATTGGATATATGTCATGAAAGCTATATCACATAAAAATCAATTTATTGCTGCTACTGTCTCCCTGCTTGACA
AATCTAAAATATGGCATAAAAGGTTAAGATCCATAAGTGATAAAAATCTGCATGTCCTTAAAAAACAAGGTCTCT
TGGAAAATGATAAAATTGAAAGAGTGTCATATTGTAAACATTGCATCTATGGAAAGCAGAAAAAATTACCTTTTA
CTACTGGTACCCATAAATCTAAAGTTGTTCTTGATTACTTACATGCTGACCTGTGGGGGCCAACTTCTGTAAGTA
CTTATTCTGGTTTTAAATACTATTTCCTGATTGTTGATGATTTTCAAGGAAAAATTGGGTTATGTTCTTGAAAAA
TAAATCTGATACCTTGCAAAACTTTGTTTCTTGGAAAGCTCTTGTTGAAAATCAAGTTAACAAGAAGGTTAAAGC
TCTTAAAACTGATAATGGCCTTGAATTCCGCAATGAAGAATTTGATAAGTACTTCAGAGATAATGAGATCCTTAG
GCATGAGACTGTCACTTACACACCACAATAGAATGGACTCGCAGAAAGAATGAATAGGACCCTTCTAAAAAGAAT
CAGGTGCATGTTGATAAGTTCAGGTCTTTCAAAGTTATTCTAGGGGAGGCAGCTAAAAACTGCCTCATGTATGAT
AAATAAATCTCCCACTAAATCCCTGAATTATGATACCCCTGATCATACTTTGAAATGGACATTTTATTGGCTATT
CCAAAATTAAAATTTTTGGTTGTATTACTTATGTACGCTAAAGTGAAGGGAAGCTTCAGCCAAGATCTATAAAAG
GTGAATTATTAGGATATGGGGAAGGCATAAAAGGATATAGAATCTGGTTGAGAGGTCAGGGAGGATATGGAGTTG
TAATAAGCAGAAATGTAGTCTTTAACGAATCAGATATGCCCTGCATTAAAGAAAAATCAACTGTTGACACAGATT
ATGATCAAAACAGGTACTTTATGCAAACTGAACTTTTTCCGACTAACATGACCCTTGAAATGAGGATCTGAGGTG
GAGCCTGGATTAGAGCAAGGCATAGAACTTGAACTAGACCAGTCTACAGGTTCCTTAGAAGACTATCATTTGGTT
AGGGACAGAATAAGAAGAGAAATAAGGCCTCCCCTGAGATATAAAGAACCTGACTTTATTACTGCACTAAATGTT
CAAAACTTATTGGAAAATGAACTTGTAACCTATGAAGAAGCAGTTAAGAGTAAAGACTCAAACTCTTGGCAGACA
ACCATGGAAGAAAGATGCAGTCCTTAATGAAAAATAAAACTTGGGTCCTAGTAAATAAACCAAAAGGGGGAAATT
ATTGAATTCAAACGGATTTATAAGATAAAGGAAGGTGATCCTTTAACTGAAAATGTTAGGTTTAAGGCCAGATTA
GTTGCTAAAGGTTTTAACCAAAGAGATGGAATTAACTACAATGAAATTTTTCCCCAGTAGTCAAGTAAACCTCTA
TAAGAATTTTACTTTCACTTGTTTCGTAATTTGATTTTTAATTGGAATAATTGGACGTGAAAACTGCCTTTTTAC
ATAGAAATTTACATGACTCAACCAAAAAGTTTTGAACAAAAGGGGAAAGAAAACTGTGTGTGTTTATTGCAAGAA
TCTCTTTATAGTTTAAAACAACCACCAAGACAATAGTACATCAGATTTGATACTTTTATCAACAGTCTTGATTTT
AATAGAAGTGAATATGATCCATATTTTTATTATAAAGGAAGTAGAGGTCCAAATTCTTGTTATTTACTGATTTAT
GTGGATGATATGTTTCTTGCTGAATGTGATGTAACTAAAATAAATAACATTAAGCAAAAGTTAAAGCAAGAATTT
GAGATGAAGGACTTAGGATCAACCAAAAGGATTTTGGGTATGGAGATTAAAAGGGATAGATCTAATTATACCTTG
TTTCTCACACAGTAGTCATATATTTTAAAAATATTGAATATATTTTCTATGAAGAAGCTAAGGATGTCACCTTAC
CTCTAGGTTGTCACTTTAAATTCACTGCCGAACAATGTCTTAAAACTGAAATGGAAAAGAGTAGAATGGATAATG
TTCCTTGTGCAAATGTTATTGGTTCTGTTATGTATGTTTTAGTTTGTATAAGAAGTGATCTTGCTTTTGCTGTAA
GTGTCTTGAGTAGATACATGAGTAACCCGGGTGAGTATCATTGAGAAACAATGAAATGACTACTTAGATACTTAA
AAAGGACTACAAATGTAGGGCTGATGTTCAAAAATAGTAAAATTGGAATACAACTTTAAGGTTTTGTTGATGCAG
ATTTTGTTGGAAATTGTGACAATAGAAAATCTACTCCTGCATACGTGTTTACATTAAATAATACCTTTATAAATT
GGAAATATCAGCTGCAAAAAAATAGTTCCTCTATCTCCAACAGAGGCTGAGTATATTGTTGCTACAGATGCAGTTA
AGGAAGGTATTTGGTTGAAAAATTTATTAGATAATATTGGCTTTACTAGTAATAATGCTACAGTGTATTCTGATG
GTCAATCTGCCATACATCTAACTAGAAACCTTGTTTTTTATAATAGGACTAAACATATAGATGTTAAATTCCATT
TTATAAGATATATTATTGAATAAGAGAATAAAGATTGAAAAGGTTTCAACCAAGTATAACCCTATGGATGGGAAC
TAAAGTTCTTCCTTTATACAAATTTAGGGCTTGTTTAAATATCCTAAATGTTGTTACTAAGGATATTTTTGTAGG
TACATAGAGCATATGAAACCATAAGGAGGAGTTAACCCCTAATTCAGATGAAACCTAAAGATGTTAAGCAAGGTG
GAGATTGTTAAAATATGCTTAACATCTTATTATTTGAGGTCCAGTGATGTAGCCCAATAAATTGTATTTCCTATG
GTTGGCCCATGTGGTAGAATGTGACTCGGTTTGGCCCCTAGTGCTCTATCTCGCATTTACTTAATACACGACTTA
TTGAATGGCTGAGATGATGACACCTGTTATTGGCTGAACCGTTGGATGCATTATGACCTTTTATATTCTAGTCTC
TCTCATTTTTCTGGGTTTGAGGGTTTCAGTCTCTACTTGTCTTTATGATTTCTCTCTCTGAAAACCTTTGGTTTT
CATTTGTAATCTTGAGTCTTCTAGTATTCTTGGTGATGTCCAGTGTAAATCATGTTATGGTTAGAGTTTTATTGT
TCTTGGGTGAATTGTAATTCTTGGTTACTTGGGGGTAATCTGTAAACGAGAGAAAAAGTGAGAGTTGAGTTTGAG
GGCTTTAAGTCGAGTGATTCTTGGGTTTTTGATTCCTGTATTTTGACGCACTTTGCTGGGTTCTAGTGTATTGGA
GTGTTAATCACTTAGGTGGATACTCAACGTGGTGATTAGTCCATTTTTGAGTGAACATCCGGACTTACAGACGAA
ACATCTTTGTGTTCTCTGTGTTTGATTTACTCAATTTAATTTTGGGATTGTTAGATACCAACGTATTATATTCTA
ACAAGGAACCATGTGATTTCTTGATTCTTGCAGACTATACCAGTACATACACTTGCAACTGGGGTGGACTTCGCC
CAAGGGGTTATAATGGAAATTGTGTTGACATTTTCATTGTTATTCACTGTTTATGCAACAATTGTGGACCCCAAG
AAGGGGTTCCTTGAAGGGATGGGCCCACTTCTTACTGGTCTTGTTGTTGGGGCCAACGTCATGGCTGGTGGGCCA
TTCTCAGGGGCTTCAATGAACCCTGCAAGGTCATTCGGGCCAGCTTTGGTCAGCGGGGATTGGACCGATCACTGG
GTATACTGGGTTGGGCCACTCATTGGAGGTGGTCTTGCTGGATATATCTATGAGAACTTCTTCATTGTCAGATCT
CATGTTCAACTGTCAGGAGAGGAAGTTTTC**TAA**
>OeuTIP4;1 *Oeu*030229.1/*Oeu*036364.1
ATGGCTAAGATTGCCTTGGGAAATGCCCGAGAGGCCTTTCAATCGGACTGCATCCAGGCCCCTTATTGTGGAATTC
ATTTGCACGTTTCTTTTCGTGTTCGCCGGGGTCGGCGCTGCCATGGCTACCGATAAGCTGAATGGGGATCCGTTG
GTGGGACTATTTTTTGTGGCTATGGCACATGCGTTGGTGGTGGCGGTGATGATTTCAGCCGGATTCCGCGTTTCC

GGTGGCCACATAAACCCTGCTGTCACCCTCGGCCTATTAGCAGGCGGCCATATCACTGTCGTCAGATCAATCCTC
TACTGGATTGATCAGTTATTAGCTTCTACAGCAGCTTGTGCTCTGCTCAAGTACCTAACTGGAGGATTGACTATA
CCAGTACATACACTTGCAACTGGGGTGGACTTCGCCCAAGGGGTTATAATGGAAATTGTGTTGACATTTTCATTG
TTATTCACTGTTTATGCAACAATTGTGGACCCCAAGAAGGGGTTCCTTGAAGGGATGGGCCCACTTCTTACTGGT
CTTGTTGTTGGGGCCAACGTCATGGCTGGTGGGCCATTCTCAGGGGCTTCAATGAACCCTGCAAGGTCATTCGGG
CCAGCTTTGGTCAGCGGGGATTGGACCGATCACTGGGTATACTGGGTTGGGCCACTCATTGGAGGTGGTCTTGCT
GGATATATCTATGAGAACTTCTTCATTGTCAGATCTCATGTTCAACTGTCAGGAGAGGAAGTTTTC**TAA**
>OeuTIP4;1 *Oeu*030229.1/*Oeu*036364.1
MAKIALGNAREAFQSDCIQALIVEFICTFLFVFAGVGAAMATDKLNGDPLVGLFFVAMAHALVVAVMISAGFRVS
GGHINPAVTLGLLAGGHITVVRSILYWIDQLLASTAACALLKYLTGGLTIPVHTLATGVDFAQGVIMEIVLTFSL
LFTVYATIVDPKKGFLEGMGPLLTGLVVGANVMAGGPFSGASMNPARSFGPALVSGDWTDHWVYWVGPLIGGGLA
GYIYENFFIVRSHVQLSGEEVF*

***Oeu*TIP5;1 *Oeu*021708.1**

>*Oeu*TIP5;1_*Oeu*021708.1 | chr12:21524153..21525389 reverse
ATGGCGTCCTTAAAATCCCGTTTGGAGCACACTGTTACACCCACTGCCCTCAGATCGTATCTTGCAGAGTTCATT
TCCACATTCTTCTTCGTCTTCGCTTCTGTTGGAGCCACAATGTCCACGCGTAAGTTTTCATTTTCTTGTTTCATC
AAAGGGTTGGAACCACAATACCAGTGACGAAAATGGGTTAAAATCGACCCCATATTTGTTAAAAATTTGATTTCT
AATTGATTGTTTTAATAGGGCTTAGTTTGTTATTGTTTTGCTTCAATATGATTCATTGTTCGTACCTGTGTCGTG
ATTACAGTGAAAATGATGCCGGATGCAGCATCAAATTCTTCCGGTCTGGTAGCTATTGCAGTTGCAAATGCCTTT
GCATTATCAGTAGCAGTGTACATTGCTTATAATATCTCCGGTGGCCATGTGAATCCGGCTGTTACATTTGGAATG
GCTATCGGAGGCCACATAAGCATTCCGATGGCCATTTTCTACTGGATTTCTCAGACCCTCGGCTCTGTCATAGCT
TGCATTCTCTTAAAAGTGACCACTGTTGGACAGGTACTAAGAGTTGTAAATGCTTTAGAATGTACAGTCAAATGG
GTAAAGCCACGTTGCCTGAAAGATATTAACAGGTTTCTTGACATAATGGGTGTTGACAAGATATGATTGAATGAA
AAAGTTTACTGTTTTAAACAATCTATTAATAGAATGTTATGTGACATTATGTCAATGATTATATCAACATCTATA
GCACTGTAACATGATTGTCACTGGACACGACACTATCCGTTTGTGCATCTCTTTCTAGCAATGATCTCAAGAGTC
TTTTCTTCTTATTTCATTTTTGCAGCACGTTCCAACGCACTCAATTCCACAAGAAATGACCGGTTTTGGTGCAGC
CATACTGGAAGGCGTGATGACATTTACATTGGTGTACACTGTATATGCTGCTGCTGATCCGAGGCGAGGTTCCAT
GTCAATAATTGGCCCGTTGGCCATAGGGCTGGTGTCAGGGGCTAACGTACTGGCATCGGGACCTTTTACCGGTGG
CTCAATGAACCCCGCCTACTCCTTCGGTTCTGCACTTGTTGGAGGAAGTTTCAAGAATCAAGCAGTCTATTGGAT
TGGACCATTGATTGGAGCAGCCACTGCAGGAATTTTATATGAGCATGTAGTTTTCCCTGCTCAACAAGAGCCTGA
TTCATTGAGGGGAATTCCTCATGGAGTTGAATTT**TAA**
>*Oeu*TIP5;1_*Oeu*021708.1
ATGGCGTCCTTAAAATCCCGTTTGGAGCACACTGTTACACCCACTGCCCTCAGATCGTATCTTGCAGAGTTCATT
TCCACATTCTTCTTCGTCTTCGCTTCTGTTGGAGCCACAATGTCCACGCTGAAAATGATGCCGGATGCAGCATCA
AATTCTTCCGGTCTGGTAGCTATTGCAGTTGCAAATGCCTTTGCATTATCAGTAGCAGTGTACATTGCTTATAAT
ATCTCCGGTGGCCATGTGAATCCGGCTGTTACATTTGGAATGGCTATCGGAGGCCACATAAGCATTCCGATGGCC
ATTTTCTACTGGATTTCTCAGACCCTCGGCTCTGTCATAGCTTGCATTCTCTTAAAAGTGACCACTGTTGGACAG
CACGTTCCAACGCACTCAATTCCACAAGAAATGACCGGTTTTGGTGCAGCCATACTGGAAGGCGTGATGACATTT
ACATTGGTGTACACTGTATATGCTGCTGCTGATCCGAGGCGAGGTTCCATGTCAATAATTGGCCCGTTGGCCATA
GGGCTGGTGTCAGGGGCTAACGTACTGGCATCGGGACCTTTTACCGGTGGCTCAATGAACCCCGCCTACTCCTTC
GGTTCTGCACTTGTTGGAGGAAGTTTCAAGAATCAAGCAGTCTATTGGATTGGACCATTGATTGGAGCAGCCACT
GCAGGAATTTTATATGAGCATGTAGTTTTCCCTGCTCAACAAGAGCCTGATTCATTGAGGGGAATTCCTCATGGA
GTTGAATTT**TAA**
>*Oeu*TIP5;1_*Oeu*021708.1
MASLKSRLEHTVTPTALRSYLAEFISTFFFVFASVGATMSTLKMMPDAASNSSGLVAIAVANAFALSVAVYIAYN
ISGGHVNPAVTFGMAIGGHISIPMAIFYWISQTLGSVIACILLKVTTVGQHVPTHSIPQEMTGFGAAILEGVMTF
TLVYTVYAAADPRRGSMSIIGPLAIGLVSGANVLASGPFTGGSMNPAYSFGSALVGGSFKNQAVYWIGPLIGAAT
AGILYEHVVFPAQQEPDSLRGIPHGVEF*

***Oeu*TIP5;2 *Oeu*021709.1**

>*Oeu*TIP5;2_*Oeu*021709.1 | chr12:21526411..21527647 reverse
ATGGCGTCCTTAAAATCCCGTTTGGAGCACACTGTTACACCCACTGCCCTCAGATCGTATCTTGCAGAGTTCATT
TCCACATTCTTCTTCGTCTTCGCTTCTGTTGGAGCCACAATGTCCACGCGTAAGTTTTCATTTTCTTGTTTCATC
AAAGGGTTGGAACCACAATACCAGTGACGAAAATGGGTTAAAATCGACCCCATATTTGTTAAAAATTTGATTTCT
AATTGATTGTTTTAATAGGGCTTAGTTTGTTATTGTTTTGCTTCAATATGATTCATTGTTCGTACCTGTGTCGTG
ATTACAGTGAAAATGATGCCGGATGCAGCATCAAATTCTTCCGGTCTGGTAGCTATTGCAGTTGCAAATGCCTTT
GCATTATCAGTAGCAGTGTACATTGCTTATAATATCTCCGGTGGCCATGTGAATCCGGCTGTTACATTTGGAATG

GCTATCGGAGGCCACATAAGCATTCCGATGGCCATTTTCTACTGGATTTCTCAGACCCTCGGCTCTGTCATAGCT
TGCATTCTCTTAAAAGTGACCACTGTTGGACAGGTACTAAGAGTTGTAAATGCTTTAGAATGTACAGTCAAATGG
GTAAAGCCACGTTGCCTGAAAGATATTAACAGGTTTCTTGACATAATGGGTGTTGACAAGATATGATTGAATGAA
AAAGTTTACTATTTTAAACAATCTATTAATAGAATGTTATGTGACATTATGTCAATGATTATATCAACATCTATA
GCACTGTAACATGATTGTCACTGGACACGACACTATCCGTTTGTGCATCTCTTTCTAGCAATGATCTCAAGAGTC
TTTTCTTCTTATTTCATTTTTGCAGCACGTTCCAACGCACTCAATTCCACAAGAAATGACCGGTTTTGGTGCAGC
CATACTGGAAGGCGTGATGACATTTACATTGGTGTACACAGTATATGCTGCTGCTGATCCGAGGCGAGGTTCCAT
GTCAATAATTGGCCCGTTGGCCATAGGGCTGGTGTCAGGGGCTAACGTACTGGCATCGGGACCTTTTACCGGTGG
CTCAATGAACCCCGCCTACTCCTTCGGTTCTGCACTTGTTGGAGGAAGTTTCAAGAATCAAGCAGTCTATTGGAT
TGGACCATTGATTGGAGCAGCCACTGCAGGAATTTTATATGAGCATGTAGTTTTCCCTGCTCAACAAGAGCCTGA
TTCATTGAGGGGAATTCCTCATGGAGTTGAATTT**TAA**
>*Oeu*TIP5;2_*Oeu*021709.1
ATGGCGTCCTTAAAATCCCGTTTGGAGCACACTGTTACACCCACTGCCCTCAGATCGTATCTTGCAGAGTTCATT
TCCACATTCTTCTTCGTCTTCGCTTCTGTTGGAGCCACAATGTCCACGCTGAAAATGATGCCGGATGCAGCATCA
AATTCTTCCGGTCTGGTAGCTATTGCAGTTGCAAATGCCTTTGCATTATCAGTAGCAGTGTACATTGCTTATAAT
ATCTCCGGTGGCCATGTGAATCCGGCTGTTACATTTGGAATGGCTATCGGAGGCCACATAAGCATTCCGATGGCC
ATTTTCTACTGGATTTCTCAGACCCTCGGCTCTGTCATAGCTTGCATTCTCTTAAAAGTGACCACTGTTGGACAG
CACGTTCCAACGCACTCAATTCCACAAGAAATGACCGGTTTTGGTGCAGCCATACTGGAAGGCGTGATGACATTT
ACATTGGTGTACACAGTATATGCTGCTGCTGATCCGAGGCGAGGTTCCATGTCAATAATTGGCCCGTTGGCCATA
GGGCTGGTGTCAGGGGCTAACGTACTGGCATCGGGACCTTTTACCGGTGGCTCAATGAACCCCGCCTACTCCTTC
GGTTCTGCACTTGTTGGAGGAAGTTTCAAGAATCAAGCAGTCTATTGGATTGGACCATTGATTGGAGCAGCCACT
GCAGGAATTTTATATGAGCATGTAGTTTTCCCTGCTCAACAAGAGCCTGATTCATTGAGGGGAATTCCTCATGGA
GTTGAATTT**TAA**
>*Oeu*TIP5;2_*Oeu*021709.1
MASLKSRLEHTVTPTALRSYLAEFISTFFFVFASVGATMSTLKMMPDAASNSSGLVAIAVANAFALSVAVYIAYN
ISGGHVNPAVTFGMAIGGHISIPMAIFYWISQTLGSVIACILLKVTTVGQHVPTHSIPQEMTGFGAAILEGVMTF
TLVYTVYAAADPRRGSMSIIGPLAIGLVSGANVLASGPFTGGSMNPAYSFGSALVGGSFKNQAVYWIGPLIGAAT
AGILYEHVVFPAQQEPDSLRGIPHGVEF*

***Oeu*TIP5;3 *Oeu*022479.1**

>*Oeu*TIP5;3_*Oeu*022479.1 | chr1:6772133..6773251 reverse
ATGGCTTCTCTGAAATCCCGTTTGGAGCATTCAGTCACTGCCATTGCTCTTAGATCGTATCTTGCTGAGTTTATC
TCTACATTCTTCTTCGTCTTTGCTGCTGCTGGGGCAACAATGTCTTCGCGTAAGCCAATTTTCTTCATTTTTAAT
TTGGAAATTAATTGTTAATCCATTTCGTTTGATCATGGATCGTACCTTTTCCCTTTACATGATTTTTTAATGATG
TTTATTGATGTCATAAAAACAGGGAAAATGATGCCGGATGCAGCATCGGATTCCTCGAGTCTTGTTGCAATTGCA
GTTGCGAATGCGTTTGCACTGTCAGTTACAGTGTATATTTCTGCCAATATTTCCGGGGGCCACGTGAATCCGGCT
GTCACATTTGGAATGGTTGTGGGAGGCCATATTAGTATTCCTATGGCTATTTTCTATTCGATTTCGCAAACGTTT
GGTTCTGTCATGGCATGCCTGCTATTAAAAGCAACCACCGCAGGACAGGTACGTCACTCAAAGTCCCAATGAGTA
CCGCGTACTAAATGCATCAAAAATTGATTAAATGTGTGTCTGAGTGTAAGATAAACAGACAAAAAGTTTGATTAC
CCGACTAATTCTACTGATTACAACATACTAAACATGCCACTATCGACTACCCATCAGATGTACAGATGGAAAGTT
GCATCTTGGTGACTCAAATATCACATTATCTTTAACAGATACTTCATAATTGTTTTGCAGCATGTTCCAACTCAT
GCAATAGAACATGAGATGACTGGTTTTGGAGGAGCAGTGCTAGAGGGTGTTATGACTTTTGCATTGGTGTACACG
GTTTATGTAGCCTCGAACCTGAGGCCGGTTTCTGCCGGTGCCATCGGCCCCTTGGCAATTGGGCTTATTGCGGGG
GCTAACGTTCTTGCATCAGGACCGTTTACCGGTGGATCCATGAACCCGGCATACGCATTCGGTTCAGCCATTGTT
GGAGGGAGTTTTAAGAATCAAGCAGTATATTGGGTTGGACCATTGTTTGGCGCAGCCATTGCTGGGATTTTACAT
GATAATGTAGTCTGTCCTGCTCCAGTGCCTGATTCCTTTAGGGGGAATGCTGATGGAGTGGGAGTT**TAA**
>*Oeu*TIP5;3_*Oeu*022479.1
ATGGCTTCTCTGAAATCCCGTTTGGAGCATTCAGTCACTGCCATTGCTCTTAGATCGTATCTTGCTGAGTTTATC
TCTACATTCTTCTTCGTCTTTGCTGCTGCTGGGGCAACAATGTCTTCGCGGAAAATGATGCCGGATGCAGCATCG
GATTCCTCGAGTCTTGTTGCAATTGCAGTTGCGAATGCGTTTGCACTGTCAGTTACAGTGTATATTTCTGCCAAT
ATTTCCGGGGGCCACGTGAATCCGGCTGTCACATTTGGAATGGTTGTGGGAGGCCATATTAGTATTCCTATGGCT
ATTTTCTATTCGATTTCGCAAACGTTTGGTTCTGTCATGGCATGCCTGCTATTAAAAGCAACCACCGCAGGACAG
CATGTTCCAACTCATGCAATAGAACATGAGATGACTGGTTTTGGAGGAGCAGTGCTAGAGGGTGTTATGACTTTT
GCATTGGTGTACACGGTTTATGTAGCCTCGAACCTGAGGCCGGTTTCTGCCGGTGCCATCGGCCCCTTGGCAATT
GGGCTTATTGCGGGGGCTAACGTTCTTGCATCAGGACCGTTTACCGGTGGATCCATGAACCCGGCATACGCATTC
GGTTCAGCCATTGTTGGAGGGAGTTTTAAGAATCAAGCAGTATATTGGGTTGGACCATTGTTTGGCGCAGCCATT
GCTGGGATTTTACATGATAATGTAGTCTGTCCTGCTCCAGTGCCTGATTCCTTTAGGGGGAATGCTGATGGAGTG
GGAGTT**TAA**
>*Oeu*TIP5;3_*Oeu*022479.1
MASLKSRLEHSVTAIALRSYLAEFISTFFFVFAAAGATMSSRKMMPDAASDSSSLVAIAVANAFALSVTVYISAN
ISGGHVNPAVTFGMVVGGHISIPMAIFYSISQTFGSVMACLLLKATTAGQHVPTHAIEHEMTGFGGAVLEGVMTF

ALVYTVYVASNLRPVSAGAIGPLAIGLIAGANVLASGPFTGGSMNPAYAFGSAIVGGSFKNQAVYWVGPLFGAAI
AGILHDNVVCPAPVPDSFRGNADGVGV*

OeuTIP-related, considered as Pseudogenes

*Oeu*050612.1 *Oeu*TIP2;1-related

>*Oeu*050612.1 | chr18:23502061..23503102 forward
Incomplete sequence

>*Oeu*050612.1
ATGGCTGTCCGCCATGGATTTGCTCTGTTTGTGGCAATTTTCGTCGGAGCCAAAATCTCCGGTGGCCATGTAAAC
CCTGTTGTCACCTTTGGTTGGCTGTTGGTGGCAAATCACTGTTCTCACTGGCCTCTTCTATTGGATTGCTCAACT
TTTGGGCTCCATTGTAGCATGCTTCTTGCTCAAAGTTGTCACAGGTGGCTTGGTAAGTTAGTTTATTTTTCCACA
ATAATGTTACTCTTGAAATAATTTTTTTCTCGCTCACTCCAAATGTGAGAGAGCGGAAAATATTATGCCCCTATT
TGGTTGTATTAAAAAATTGATTAAAATTTAAATCAAAGTCAACTATTAATTCATGGTACACAAAATTTTTTAATT
ATACATTCATTATTTTTTATCAAAATCTACTAAATTCTGATTATTAACTAAATCATACAGCAGTTATTTCTTTCA
TGCATCTAAGTGTAGGCCATGTTCATTTTTCATTAAATGTTTGACACGTGTACAATTTTCATAAATAAGTGATTA
TGAGAATATTGAAAATAATGATTCACGAGATGAGAATCCTATTCTTTTTTGACTGTCAACACTCTTTATGAAGAA
TAGTAAAATGGGAAATTGTTTTCTAAACAAATTAGTAATTATTTTGATGATTAATTTAATAATTTCTATGCAGGC
TATTCTAATCCACGGTGTGGCTGCTGGTGTTGGAGCCATTGAAGGAGTTGTGATGGAAATTATAATCACTTTTGC
TTTGGTGTACACAGTTTATGCCACAGCAGCTGACCCAAAGAAAGGCTCATTAGGCACAATTGCCCCCATTGCTAT
TGGCTTCATTGCTGGTGCCAACATTTTGGCTGCTGGCCCATTCTCTGGTGGCTCCATGAACCTGGCTAGGTCGTT
CGGTCCAGCCGTGGCTAGCGGTGACTTTGCGGGCAACTGGATTTATTGGGTCGGTCCACTTATCGGTGGTGGCCT
GGCTGGACTTGTCTATGCCAATGTGTTCATGGTCCACGAACACACACCACTTGTTAGCGAGTTC**TAA**
>*Oeu*050612.1
ATGGCTGTCCGCCATGGATTTGCTCTGTTTGTGGCAATTTTCGTCGGAGCCAAAATCTCCGGTGGCCATGTAAAC
CCTGTTGTCACCTTTGCATGCTTCTTGCTCAAAGTTGTCACAGGTGGCTTGGCTATTCTAATCCACGGTGTGGCT
GCTGGTGTTGGAGCCATTGAAGGAGTTGTGATGGAAATTATAATCACTTTTGCTTTGGTGTACACAGTTTATGCC
ACAGCAGCTGACCCAAAGAAAGGCTCATTAGGCACAATTGCCCCCATTGCTATTGGCTTCATTGCTGGTGCCAAC
ATTTTGGCTGCTGGCCCATTCTCTGGTGGCTCCATGAACCTGGCTAGGTCGTTCGGTCCAGCCGTGGCTAGCGGT
GACTTTGCGGGCAACTGGATTTATTGGGTCGGTCCACTTATCGGTGGTGGCCTGGCTGGACTTGTCTATGCCAAT
GTGTTCATGGTCCACGAACACACACCACTTGTTAGCGAGTTC**TAA**
>*Oeu*050612.1
MAVRHGFALFVAIFVGAKISGGHVNPVVTFACFLLKVVTGGLAILIHGVAAGVGAIEGVVMEIIITFALVYTVYA
TAADPKKGSLGTIAPIAIGFIAGANILAAGPFSGGSMNLARSFGPAVASGDFAGNWIYWVGPLIGGGLAGLVYAN
VFMVHEHTPLVSEF*

*Oeu*007734.1 *Oeu*TIP1;3-related

>*Oeu*007734.1 | chr4:20127443..20127820 forward
Incomplete sequence

>*Oeu*007734.1
ATGCAGGAAACATCTGCATTTGCTTTGGGTGCCAACGTGTCTGTGTGGAACGCCTTTGTATTTGAGATTGTAATG
ACTTTTGGCCTGGTTTACACTGTGTACGCCACCGCGATTGACCCGAAGAAGGGAGAAGTTGGCATTATTGCACCA
ATTGCAATTGGTTTCATTGTGGGTGCCAACATTCTTGCTGGTGGGGCATTTACTGGTGCTTCAATGAACCCTGCA
GTGTCATTTGGCCCTGCTCTGGTTAGCTTCACCTGGACCCATCACTGGGTATACTGGGCTGGACCTCTAATTGGT
GGTGGACTTGCTGGTATTGTTTATGAAGTCTTCTTCATCAGTCATTCTCACGAGCCACTGCCCGGATCGGAGTAC
TGA
>*Oeu*007734.1
ATGCAGGAAACATCTGCATTTGCTTTGGGTGCCAACGTGTCTGTGTGGAACGCCTTTGTATTTGAGATTGTAATG
ACTTTTGGCCTGGTTTACACTGTGTACGCCACCGCGATTGACCCGAAGAAGGGAGAAGTTGGCATTATTGCACCA
ATTGCAATTGGTTTCATTGTGGGTGCCAACATTCTTGCTGGTGGGGCATTTACTGGTGCTTCAATGAACCCTGCA
GTGTCATTTGGCCCTGCTCTGGTTAGCTTCACCTGGACCCATCACTGGGTATACTGGGCTGGACCTCTAATTGGT
GGTGGACTTGCTGGTATTGTTTATGAAGTCTTCTTCATCAGTCATTCTCACGAGCCACTGCCCGGATCGGAGTAC
TGA
>*Oeu*007734.1
MQETSAFALGANVSVWNAFVFEIVMTFGLVYTVYATAIDPKKGEVGIIAPIAIGFIVGANILAGGAFTGASMNPA
VSFGPALVSFTWTHHWVYWAGPLIGGGLAGIVYEVFFISHSHEPLPGSEY*

*Oeu*041020.1 *Oeu*TIP-related

>Oeu041020.1 | chr20:11792223..11792531 forward
Incomplete sequence

>*Oeu*041020.1
ATGACGTTTGGCCTGGTTTACACAGTTTATGCCACGGCAGTGGATCCAAAGAGGGGTGACATTGGTATCATAGCC
CCTATTGCCATTGGCTTCATTGTGGGTGCCAACATTTTGGCTGGCGGTGCCTTCGACGGTGCATCCATGAACCCA
GCCGTGTCGTTCGGCCCTGCAGTCGTCAGCTGGACATGGGACAGCCACTGGGTGTACTGGCTTGGCCCATTTGTT
GGTGCTGGAATTGCTGCTATTGTCTATGAAATCGTCTTCATTAACCAGACCACTCATGAACCACTCCCCTCCAGT
GAGTAC**TAA**
>*Oeu*041020.1
ATGACGTTTGGCCTGGTTTACACAGTTTATGCCACGGCAGTGGATCCAAAGAGGGGTGACATTGGTATCATAGCC
CCTATTGCCATTGGCTTCATTGTGGGTGCCAACATTTTGGCTGGCGGTGCCTTCGACGGTGCATCCATGAACCCA
GCCGTGTCGTTCGGCCCTGCAGTCGTCAGCTGGACATGGGACAGCCACTGGGTGTACTGGCTTGGCCCATTTGTT
GGTGCTGGAATTGCTGCTATTGTCTATGAAATCGTCTTCATTAACCAGACCACTCATGAACCACTCCCCTCCAGT
GAGTAC**TAA**
>*Oeu*041020.1
MTFGLVYTVYATAVDPKRGDIGIIAPIAIGFIVGANILAGGAFDGASMNPAVSFGPAVVSWTWDSHWVYWLGPFV
GAGIAAIVYEIVFINQTTHEPLPSSEY*

*Oeu*000463.1 *Oeu*TIP2;2-related

>*Oeu*000463.1 | chr17:6142042..6142582 forward
Incomplete sequence

>*Oeu*000463.1
ATGCCTGGAATCGCTTTCGGAAGATTTGACGATTCATTTACCATTGGTTCATGAAGGCCTATGTGGCTGAATTCA
TATCCACCTTGCTCTTCGTCTTCACCTAATTGGGCTCCACCATTGCTTATAGTTAGTACTAAAACCACTCTCTTA
TTTTTCACAGCCTATATTTGCGTGCATGAAAACTATAGTAAGTGCGATATATAATATGCTGATATTTAGTAACAT
TTTGATAGTTGATAGATAAGTTGGCGGCGGACGCGGCCCTTGATCCAGCAGGGTTGGTGGCGTTGCTGTTTGCCA
TGGTTTCGCCCTGTTTGTAGCTGTCTCGGTCGGAACCAACATCTCCGACGGCCATGTAAACCCTGCAGTCACCTT
TGGTTTGGCTCTTGACAGCCAAATTACACTTCTTAATGGCATTTTTTTATTGGATTGCTCAACTTTTGGCTCCAT
TGTGGCATGCTTTTTGCTTGTAGTTGTCACCGGAGGATTGGTAAGTTCAATTGTAATTGAAAATGCAAAATTTAC
ATGTTTAGTAAGT**TAG**
>*Oeu*000463.1
ATGCCTGGAATCGCTTTCGGAAGATTTGACGATTCATTTACCATTGGTTCAGTTGCTGTTTGCCATGGTTTCGCC
CTGTTTGTAGCTGTCTCGGTCGGAACCAACATCTCCGACGGCCATGTAAACCCTGCAGTCACCTTTGGTTTGGCT
CTTGACAGCCAAATTACACTTCTTAATGGCATTTTTTTATTGGATTGCTCAACTTTTGGCTCCATTGTGGCATGC
TTTTTGCTTGTAGTTGTCACCGGAGGATTGGTAAGTTCAATTGTAATTGAAAATGCAAAATTTACATGTTTAGTA
AGT**TAG**
>*Oeu*000463.1
MPGIAFGRFDDSFTIGSVAVCHGFALFVAVSVGTNISDGHVNPAVTFGLALDSQITLLNGIFLLDCSTFGSIVAC
FLLVVVTGGLVSSIVIENAKFTCLVS*

XIP – Uncharacterized *X* intrinsic proteins (XIPs)

*Oeu*XIP1;1 *Oeu*010056.1

Observation: exon 1 downstream extended with 21n

>*Oeu*XIP1;1_*Oeu*010056.1 | chr3:335356..336578 forward **Original version**
ATGGCTGCTAATATTTGGAATGTACATGGAGTAGATGATGAAGAAAGTCAAACCAGTTTGAGCAAAATTCAGCCT
GTCCCCTCTACACCAGTGCAAGTTCTTTTTTTCTTAAATCTCAAGTTCAATATCTTCAGTTTCTTGTCCTTTTAT
CCTGAAAAAATTTGAGGGTATTTCTTAGTTTCTTACATTTTGTTTGGCAGGACACAACAATTGACAGCTGATCTC
GATAAGAAGAAGCAAACTCCTCTTACCTCGAGTCAAATGCTTGGTTTGAACGAGCTTTTCTCCCTAGACGTTAGT
ACATATCCCGATATGTACTTGCAAATTTTAGTTTCTTCTTCCTTTTAATTACTGTATAGTCTTATAAATAAGACT
GTCCTATATAATCCAAGTAACTTAAGTTTTCTTGATAACTGAAGGTTTGGAGGGCATCGGTGGGAGAGCTTATTG
GATCAGCAGTTCTAGTATTCATGATTGACACTATTGTGATCTCAACCTTTGAGACTGATATCAAAATGCCAAACC
TCATAATCTCAATTCTCATTGCCATTGTCGTTGCAATTCTCCTCCTCGCTGTACATCCTGTTTCCGGTGGCCACA
TCAACCCCGTTATCTCCTTTTCGGCTGGGCTAGTGGGCCTTATCTCCATGTCACGCGCTATAGTTTACATCCTAG
CCCAATGCATTGGTGCAATACTTGGTGCACTGGCCCTCAAAGCCGTAGTGAGCAGCACCATTGAACAAAACTTTT
CACTTGGAGGTTGCACTCTAACAGTGATTGCACCTGGACCCAATGGACCCATAACCCTTGGCCTTGAGACGGCAC
AAGCCTTTTGGCTCGAGATTTTCTGCACCTTTGTTTTCCTTTTTGCTTCAATTTGGATGGCATATGATCATCGCC
AAGCAAAGAATTTAGGCCTTGTTCTTGTTTTTTCAATTATAGGACTTGTGCTAGGCCTCTTAGTGTTTATTTCCA
CCACTGTCACTGCCAAAAAGGGCTATGCCGGGGCAGGAATGAACCCTGCTAGGTGCCTTGGGGCTGCAATTGTTA
GAGGTGGCCACCTCTGGGATGGCCACTGGGTGTTTTGGGCCGGGCCTGCTATTGCTTGTGTTGCCTTCTATTTAT
ACACAAAGATAATTCCACGGCAGCACCATCATGCAAAGGGCTATGGACATGATTTCTTCGATACTGTGAAGGCTA
TGCTTGGATTTGTTGATCAC**TGA**

>*Oeu*XIP1;1_*Oeu*010056.1 **Corrected version**
ATGGCTGCTAATATTTGGAATGTACATGGAGTAGATGATGAAGAAAGTCAAACCAGTTTGAGCAAAATTCAGCCT
GTCCCCTCTACACCAGTGCAAGTTCTTTTTTTCTTAAATCTCAAGTTCAATATCTTCAGTTTCTTGTCCTTTTAT
CCTGAAAAAATTTGAGGGTATTTCTTAGTTTCTTACATTTTGTTTGGCAGGACACAACAATTGACAGCTGATCTC
GATAAGAAGAAGCAAACTCCTCTTACCTCGAGTCAAATGCTTGGTTTGAACGAGCTTTTCTCCCTAGACGTTAGT
ACATATCCCGATATGTACTTGCAAATTTTAGTTTCTTCTTCCTTTTAATTACTGTATAGTCTTATAAATAAGACT
GTCCTATATAATCCAAGTAACTTAAGTTTTCTTGATAACTGAAGGTTTGGAGGGCATCGGTGGGAGAGCTTATTG
GATCAGCAGTTCTAGTATTCATGATTGACACTATTGTGATCTCAACCTTTGAGACTGATATCAAAATGCCAAACC
TCATAATCTCAATTCTCATTGCCATTGTCGTTGCAATTCTCCTCCTCGCTGTACATCCTGTTTCCGGTGGCCACA
TCAACCCCGTTATCTCCTTTTCGGCTGGGCTAGTGGGCCTTATCTCCATGTCACGCGCTATAGTTTACATCCTAG
CCCAATGCATTGGTGCAATACTTGGTGCACTGGCCCTCAAAGCCGTAGTGAGCAGCACCATTGAACAAAACTTTT
CACTTGGAGGTTGCACTCTAACAGTGATTGCACCTGGACCCAATGGACCCATAACCCTTGGCCTTGAGACGGCAC
AAGCCTTTTGGCTCGAGATTTTCTGCACCTTTGTTTTCCTTTTTGCTTCAATTTGGATGGCATATGATCATCGCC
AAGCAAAGAATTTAGGCCTTGTTCTTGTTTTTTCAATTATAGGACTTGTGCTAGGCCTCTTAGTGTTTATTTCCA
CCACTGTCACTGCCAAAAAGGGCTATGCCGGGGCAGGAATGAACCCTGCTAGGTGCCTTGGGGCTGCAATTGTTA
GAGGTGGCCACCTCTGGGATGGCCACTGGGTGTTTTGGGCCGGGCCTGCTATTGCTTGTGTTGCCTTCTATTTAT
ACACAAAGATAATTCCACGGCAGCACCATCATGCAAAGGGCTATGGACATGATTTCTTCGATACTGTGAAGGCTA
TGCTTGGATTTGTTGATCAC**TGA**

>*Oeu*XIP1;1_*Oeu*010056.1
ATGGCTGCTAATATTTGGAATGTACATGGAGTAGATGATGAAGAAAGTCAAACCAGTTTGAGCAAAATTCAGCCT
GTCCCCTCTACACCAGTGCAAGTTCTTTTTTTCTTAAATCTCAAGTTCAATATCTTCAGTTTCTTGACACAACAA
TTGACAGCTGATCTCGATAAGAAGAAGCAAACTCCTCTTACCTCGAGTCAAATGCTTGGTTTGAACGAGCTTTTC
TCCCTAGACGTTTGGAGGGCATCGGTGGGAGAGCTTATTGGATCAGCAGTTCTAGTATTCATGATTGACACTATT
GTGATCTCAACCTTTGAGACTGATATCAAAATGCCAAACCTCATAATCTCAATTCTCATTGCCATTGTCGTTGCA
ATTCTCCTCCTCGCTGTACATCCTGTTTCCGGTGGCCACATCAACCCCGTTATCTCCTTTTCGGCTGGGCTAGTG
GGCCTTATCTCCATGTCACGCGCTATAGTTTACATCCTAGCCCAATGCATTGGTGCAATACTTGGTGCACTGGCC
CTCAAAGCCGTAGTGAGCAGCACCATTGAACAAAACTTTTCACTTGGAGGTTGCACTCTAACAGTGATTGCACCT
GGACCCAATGGACCCATAACCCTTGGCCTTGAGACGGCACAAGCCTTTTGGCTCGAGATTTTCTGCACCTTTGTT
TTCCTTTTTGCTTCAATTTGGATGGCATATGATCATCGCCAAGCAAAGAATTTAGGCCTTGTTCTTGTTTTTTCA
ATTATAGGACTTGTGCTAGGCCTCTTAGTGTTTATTTCCACCACTGTCACTGCCAAAAAGGGCTATGCCGGGGCA
GGAATGAACCCTGCTAGGTGCCTTGGGGCTGCAATTGTTAGAGGTGGCCACCTCTGGGATGGCCACTGGGTGTTT
TGGGCCGGGCCTGCTATTGCTTGTGTTGCCTTCTATTTATACACAAAGATAATTCCACGGCAGCACCATCATGCA
AAGGGCTATGGACATGATTTCTTCGATACTGTGAAGGCTATGCTTGGATTTGTTGATCAC**TGA**

>*Oeu*XIP1;1_*Oeu*010056.1
MAANIWNVHGVDDEESQTSLSKIQPVPSTPVQVLFFLNLKFNIFSFLTQQLTADLDKKKQTPLTSSQMLGLNELF
SLDVWRASVGELIGSAVLVFMIDTIVISTFETDIKMPNLIISILIAIVVAILLLAVHPVSGGHINPVISFSAGLV
GLISMSRAIVYILAQCIGAILGALALKAVVSSTIEQNFSLGGCTLTVIAPGPNGPITLGLETAQAFWLEIFCTFV

FLFASIWMAYDHRQAKNLGLVLVFSIIGLVLGLLVFISTTVTAKKGYAGAGMNPARCLGAAIVRGGHLWDGHWVF
WAGPAIACVAFYLYTKIIPRQHHHAKGYGHDFFDTVKAMLGFVDH*

***Oeu*XIP1;2 *Oeu*020684.1**

Observation: 5′ extended with 1455n, generating 2 exons and 2 introns

>*Oeu*XIP1;2_*Oeu*020684.1 chr5:9245927..9247542 reverse Original version
ATGCTTGACACTATTGTGATATCAACCATTGAGTCTGATATCAAAATTCCAAACCTTGTACTATCAATCCTTGCA
GCCATTATTATTGCTATACTACTTCTTGCTGTACATCCAGTTTCCGGTGGCCACATCAACCCGGTTATCTCCTTC
TCTGCCGCCCTGGTTGGGTTAATCTCTATGTCGAAAGCCTTCATATACATTGTTGCACAATGTCTTGGAGCCGTG
CTAGGTGCGGGTTGAAAACCCAATGAACAAACTTTATTTTGTTTCAAAGTAGTCCCCTCACGTTTATGTTTTCTT
TCAGCTTGATGACTATGGCAACATGTTCGACTTAAATTGGATGGTCAAAGTGAACAAAAAGCAAACTACTTAGGG
CCTGTTTGAAAGGGCCAATTTTCCATGGAAAATGGAAACATTTTTCCCTTTTGGCCTGTTTGATTGCAATAATTT
TCTAACCCCCACATGACCATTTTTCCTTGATTTTGTGGAAAACGGGAATTGGTTTTCCAGCTGATTTTTTTCTTTT
TTTTTTTTTAATATTTTTTGGCAATTGGAACAAATTCTATTTATAATTGTCAAAAATTCTATCATTTTAATTGAC
AATTTTCATGAAACACAAAGTCAAATGAACACTAAAAGATCCAAAATTTCAACATCTTTTAAGTGAAAAATTATA
CCAATCAAACACATTTAATTGACAATTTCCAATAAAATAAGTTCTAGGAAATTTAATTCTCTGAAATTCTTTTTC
TTTTCACCCAAATTTCATTCTTACAATCAAACGGATCCTTAGGGTTATTCCCCCCTGTATAGAGAGTTTTAGCGC
AGCACCCATGTGGTTGGAATTTTTTTTCCCCAAAGCTCAATAGAATAATGCCAAGTGTCTCCCTTTAAACAAATC
TAGAAAGTTTGTCTATACACATGGCACGGTTCAACCACTTAAAAGTATGTTTCGTTTAGATTAAAATCATCACAT
ATTTCAAAATCTTCTATTACAATATACTAAAAATCTAATTTTAATCCTTACCAAACACCATCTAAATAAAGTTCG
ATAATGGGTTGAAATTTTGCTATGAGTTGAGCGACTAACAATATTTGCTTACAGGTGCACTAGCTCTTAAAGCTG
TGGTCAGTAGCACCACTGAAGAAACCTTCTCACTTGGCGGCTGCACCCTGACTGTGATTGCACCCGGCCTGAATG
GACCTGCTACGGTAGGCCTCGAGACGGTCCAAGCCTTTTGGCTAGAAATATTTTGCACCTTTGTGTTCCTTTTTG
CTTCAATTTGGATGGCATATGATCCACGGCAAGCAAAGGAATTAGGTCATGTCCTCATCTTTTCAATTGTGGGAG
TTGTGTTAGGTCTCCTAGTATTTATCTCCACCACTGTCACAGCCAAAAAGGGATATGCTGGGGCCGGGATGAACC
CGGCTAGGTGTTTTGGCCCGGCGATTGTTAGAGGCGGCCACCTATGGAATGTCTACTGGGTTTTCTGGGCTGGCC
CGACCATTGCATGCGTGGCATTCTATTTGTACACACAGATTATTCCATGTCAGCACCACCGGTCGAAGTATTCTT
ATGATCATGATTTCTATAATGTTGTCAAGGCTATATTT**TGA**
>*Oeu*XIP1;2_*Oeu*020684.1 chr5: 9245927..9248997 Corrected version
ATGGCTGGTAATAATGGGCATGTAAATGTAGAATATGATGAAGAAGGCTTGAGTGGTGCAAGCAAAATTCAACCT
GTCTTCTCTACTCCAATGCAAGTTCATTTCTTTCTTTGAACTTTTATATTATTATATTAAATTTTTCAAAATCTT
GTTATAGTTGATAAAAATAGGGGGTGGACATGGTTCCGTTTAATCCTAGTTCCAAGATAATCGAATCGAACTCAA
TCATGAATCAATTATTTTTTTGGTAATGGAACTAATGGTTTTTATTTTATTTTATTTTCTAAAGATATGGAACCG
AGTTAAGTTCGATTTCATTTAATTTTGGTCTGGTTTGGTTTGATTCCATTTTTAAGTAACGGAACCTGAACCGAA
TCTTTAAAGAAGAAGAAGAAGAAATCATTGGTTTCATGATTAAAAAAGCCATTGGTTCGTGGTTCGCTTCGATTC
TCTTGGAATCAAAACCAAATGGACCATGTCTACCCTAGGTAAAAATATAGTCAATTTAGATTTTTAATAGTTATA
GTGCTTGTTTGGTAGAGATTTTGTTAATTGATTTTTTGACCGATAGGATTCTACTTGTGAGAAAAGAATTCAAAA
ACGACGGCTGGAATAGTGAAATAATGGTAAAAAAACTAATGTTAATTGAATTAAAATCTCTACCAAACATGCTCA
TAGCAGTGAACTGAAAGAATAGATAGGAAAGAACAATCACTCTTTTCTTTTTCTTTTTCCTTTTTTTCCTTTTCT
TTTTTTCCTTTTCTTTCTTTCTTTCTTTCTTTTTTTTTTTGAAGATCATATGTTTTCCAATCTTGTTCATGTCAT
CGTAGCTGACCTTAAATTTATTAAGAATATTATGTTTCTTCAAATCTCGTAGTTGATACGTCTCCGTGAACTAAA
TAGAAAGATGAAAATGATTCAAGTAGTCCATCCAAAATTTATAAAGAATAACCAATAGTTTATCTTTTGTTGAGG
TTTTCTTTAAAAATCTTGTTGTTCTGGGAAGTTAAAACTGTGTATGCTTAGCAGGCCAGAGAACAGGACAGATGA
GGAGAAAATGAAGAAGACAACTTCTCTTACTCTGAGTGAATTGCTGGGTTTGGACGACTTCCTTTCCTCAGATGT
TAGTACTTCTTTCCTCTTCAAGAAAATGTCTTTAGATTAGTGTTCAAAGATAAATTTTTCATAAATAAACATGTC
ATGTCTTCAATAATTTAAACTTTTAACAAGATAGTTCAACTTAGTATTGGAATGGCAAAGATCCACCGTTCAAGT
TTCACCAATTACTTACATGCGGAAAACTGGATTTAAATAAATCAATATGCCGTATATCTAACAATTTAAACTTTT
AAATGAGATAATATGAGTAAGAAAATATCATGTTATTTTTGTTTATTCTTGAAGGTTTGGAGAGCCTCAGTAGGC
GAGCTGGTTGGCACGGCCGTTCTTGTATTCATGCTTGACACTATTGTGATATCAACCATTGAGTCTGATATCAAA
ATTCCAAACCTTGTACTATCAATCCTTGCAGCCATTATTATTGCTATACTACTTCTTGCTGTACATCCAGTTTCC
GGTGGCCACATCAACCCGGTTATCTCCTTCTCTGCCGCCCTGGTTGGGTTAATCTCTATGTCGAAAGCCTTCATA
TACATTGTTGCACAATGTCTTGGAGCCGTGCTAGGTGCGGGTTGAAAACCCAATGAACAAACTTTATTTTGTTTC
AAAGTAGTCCCCTCACGTTTATGTTTTCTTTCAGCTTGATGACTATGGCAACATGTTCGACTTAAATTGGATGGT
CAAAGTGAACAAAAAGCAAACTACTTAGGGCCTGTTTGAAAGGGCCAATTTTCCATGGAAAATGGAAACATTTTT
CCCTTTTGGCCTGTTTGATTGCAATAATTTTCTAACCCCCACATGACCATTTTTCCTTGATTTTGTGGAAAACGG
GAATTGGTTTTCCAGCTGATTTTTTTCTTTTTTTTTTTTTAATATTTTTTGGCAATTGGAACAAATTCTATTTATA
ATTGTCAAAAATTCTATCATTTTAATTGACAATTTTCATGAAACACAAAGTCAAATGAACACTAAAAGATCCAAA
ATTTCAACATCTTTTAAGTGAAAAATTATACCAATCAAACACATTTAATTGACAATTTCCAATAAAATAAGTTCT
AGGAAATTTAATTCTCTGAAATTCTTTTTCTTTTCACCCAAATTTCATTCTTACAATCAAACGGATCCTTAGGGT

TATTCCCCCCTGTATAGAGAGTTTTAGCGCAGCACCCATGTGGTTGGAATTTTTTTCCCCAAAGCTCAATAGAA
TAATGCCAAGTGTCTCCCTTTAAACAAATCTAGAAAGTTTGTCTATACACATGGCACGGTTCAACCACTTAAAAG
TATGTTTCGTTTAGATTAAAATCATCACATATTTCAAAATCTTCTATTACAATATACTAAAAATCTAATTTTAAT
CCTTACCAAACACCATCTAAATAAAGTTCGATAATGGGTTGAAATTTTGCTATGAGTTGAGCGACTAACAATATT
TGCTTACAGGTGCACTAGCTCTTAAAGCTGTGGTCAGTAGCACCACTGAAGAAACCTTCTCACTTGGCGGCTGCA
CCCTGACTGTGATTGCACCCGGCCTGAATGGACCTGCTACGGTAGGCCTCGAGACGGTCCAAGCCTTTTGGCTAG
AAATATTTTGCACCTTTGTGTTCCTTTTTGCTTCAATTTGGATGGCATATGATCCACGGCAAGCAAAGGAATTAG
GTCATGTCCTCATCTTTTCAATTGTGGGAGTTGTGTTAGGTCTCCTAGTATTTATCTCCACCACTGTCACAGCCA
AAAAGGGATATGCTGGGGCCGGGATGAACCCGGCTAGGTGTTTTGGCCCGGCGATTGTTAGAGGCGGCCACCTAT
GGAATGTCTACTGGGTTTTCTGGGCTGGCCCGACCATTGCATGCGTGGCATTCTATTTGTACACACAGATTATTC
CATGTCAGCACCACCGGTCGAAGTATTCTTATGATCATGATTTCTATAATGTTGTCAAGGCTATATTT**TGA**

>*Oeu*XIP1;2_*Oeu*020684.1

ATGGCTGGTAATAATGGGCATGTAAATGTAGAATATGATGAAGAAGGCTTGAGTGGTGCAAGCAAAATTCAACCT
GTCTTCTCTACTCCAATGCAAAACAGGACAGATGAGGAGAAAATGAAGAAGACAACTTCTCTTACTCTGAGTGAA
TTGCTGGGTTTGGACGACTTCCTTTCCTCAGATGTTTGGAGAGCCTCAGTAGGCGAGCTGGTTGGCACGGCCGTT
CTTGTATTCATGCTTGACACTATTGTGATATCAACCATTGAGTCTGATATCAAAATTCCAAACCTTGTACTATCA
ATCCTTGCAGCCATTATTATTGCTATACTACTTCTTGCTGTACATCCAGTTTCCGGTGGCCACATCAACCCGGTT
ATCTCCTTCTCTGCCGCCCTGGTTGGGTTAATCTCTATGTCGAAAGCCTTCATATACATTGTTGCACAATGTCTT
GGAGCCGTGCTAGGTGCACTAGCTCTTAAAGCTGTGGTCAGTAGCACCACTGAAGAAACCTTCTCACTTGGCGGC
TGCACCCTGACTGTGATTGCACCCGGCCTGAATGGACCTGCTACGGTAGGCCTCGAGACGGTCCAAGCCTTTTGG
CTAGAAATATTTTGCACCTTTGTGTTCCTTTTTGCTTCAATTTGGATGGCATATGATCCACGGCAAGCAAAGGAA
TTAGGTCATGTCCTCATCTTTTCAATTGTGGGAGTTGTGTTAGGTCTCCTAGTATTTATCTCCACCACTGTCACA
GCCAAAAAGGGATATGCTGGGGCCGGGATGAACCCGGCTAGGTGTTTTGGCCCGGCGATTGTTAGAGGCGGCCAC
CTATGGAATGTCTACTGGGTTTTCTGGGCTGGCCCGACCATTGCATGCGTGGCATTCTATTTGTACACACAGATT
ATTCCATGTCAGCACCACCGGTCGAAGTATTCTTATGATCATGATTTCTATAATGTTGTCAAGGCTATATTT**TGA**

>*Oeu*XIP1;2_*Oeu*020684.1

MAGNNGHVNVEYDEEGLSGASKIQPVFSTPMQNRTDEEKMKKTTSLTLSELLGLDDFLSSDVWRASVGELVGTAV
LVFMLDTIVISTIESDIKIPNLVLSILAAIIIAILLLAVHPVSGGHINPVISFSAALVGLISMSKAFIYIVAQCL
GAVLGALALKAVVSSTTEETFSLGGCTLTVIAPGLNGPATVGLETVQAFWLEIFCTFVFLFASIWMAYDPRQAKE
LGHVLIFSIVGVVLGLLVFISTTVTAKKGYAGAGMNPARCFGPAIVRGGHLWNVYWVFWAGPTIACVAFYLYTQI
IPCQHHRSKYSYDHDFYNVVKAIF*

*Oeu*XIP1;3 *Oeu*046122.1

Observation: 5′ extended with 855n, generating 2 exons and 2 introns

>*Oeu*XIP1;3_*Oeu*046122.1 chr16:11367503..11368665 reverse Original version

ATGCTTGACACTATTGTGATATCAACCATTGAGACTGATGTCAAAATGCCAAACCTTGTATTGTCAATCCTTGCA
GCCATTATTATTGCAATAGTACTTCTTGCAGTGCATCCTATTTCTGGAGGCCACATCAACCCAGTTATCTCCTTC
TCGGCTGCCCTAGTTGGGTTGATCTCTGTGTCGAAAGCCTTAATCTACATTAGTGCACAATGTCTTGGAGCCGCG
CTAGGTGAGGGTTAAACTCCCTATCGACGAACTTTATTTTGTTCCAAAGTAGTCCATGAAGTTCACGTTGTCTTC
CAGTTTGATGACTAAAGTTAAAATTCTTGAAAAATGCTACCTGTACAGATTTTTTAACAGACAGGGTTACAGGT
GCTGAGTTGATAAGATTTTATTGGTTGGTTTAACTTTGTTTTGTTATCCCAAATGGTGTGACATCTCGTCAACGT
AGCATTCTGTTAAAACAGTTTGTACAAGTAGCATTTCTCCAAATTCTTTAGTTTTTCTAATAGAATGTTTGGCTT
AGATCAGATGATCAGGTGAAACAAAAAGCAAACTCTAGTTATCACTCTAAAGCAAAGAAATGTTCGATAAATAAT
ATGAAACCTCACTATAAATTGAGTGACTAACAATGTTTTTTTACGCGACTACAGGTGCACTAGCTCTTAAAGCTG
TGGTTAGTAGCACCATTGAACAAACCTTCTCACTTGGAGGCTGCACCCTGACAGTGATTGCCCCTGGCCCGAATG
GGCTCGTTACAATCGGCCTCGAGACTGCCCAAGCCTTTTGGCTAGAAATAATTTGTACCTTCGTACTCCTTTTTG
CTTCAGTTTGGATGGCATATGATCATCGCCAAGCAAAGGCATTAGGTCGTGTTCTTGTCTTTTCAATTGTGGGAG
TTGTGCTAGGTCTCTTAGTGTTCATCTCCACCACAGTCACGGCCAAAAAGGGCTACGCCGGGGCGGGGATGAACC
CGGCTAGGTGTTTTGGTGCAGCGGTTGTTAGAGGTGGCCACCTATGGAATGGACACTGGATTTTCTGGGTGGGCC
CCGCCATTGCTTGCGTGGCCTTCTATTTGTACACGAAGATTATTCCACGTCAGCATTATCAGGCGAAGTTTTATG
ATCACGATTTCTTTAATGTTGTCAAGGCTATGTTT**TGA**

>*Oeu*XIP1;3_*Oeu*046122.1 chr5: 11367503..11369530; Corrected version

ATGGCCGGTGATAATGGGCATGTACATGTAGAATATGATGAAGAAAGTTTGAGTAGTGCAAACAAAATTCAACCT
GTCTTCTCTACACCTATGCAAGTTCATTTTCTTTCCTTCAACTTTTACTTATTTATATATGTTTTTCAAATCTTC
TTGTAGTTGGAAAAAAAATAGTCGATTTAGATTTTCCAGTAGGTGTAACAGTGAAGCGAACGAAAAGATGGGAAA
GATGTTCACAGTAGCTAGATATGTATCCGTAGTAGATTCCAAATTTATTAAGAATAAAGCATAATTTATCATTTG
TTGTGGTTTCTTCAAAACTCTTGTCGTTTTGATAAGTTAAAACTTCGAACGTTTAGCAGGCCAGAGCACTGGACA
GATGAGCAGAAAAGGACGAAGAATACTTCTCTTACACTGAGTGAATCGCTGGGCTTGGAAGACTTCCTTTCTTTA
GATGTTTGTACCTTTTTTTCCTCTTCAACCACCGCGCCCTGCTCCTCCCCACCCCACTCAAAGATTAAATATACA

TGATTAAACATGTCATTATTAAAGTCTTCCGAATGATGCGATTCTATATAGTGTTAGAATGACAAAATCTTGCTT
TCAAGACTCGCCACCGGACCGGTATGAAAATTATGCATATGATTGAGCCTTTTGATGAGAATATTTCTACCTACA
TCTGAAAGGACGTGCTAAAAATTGAATTTAAACAAATAAATTTTTTTCTATCTATAAATTTAAACTTTTAAACAA
GATGATTCTTGTTTACTCTTGAAGGTTTGGAGAGCCTCACTGGGAGAGCTGATTGGCACAGCAGTTCTTGTGTTC
ATGCTTGACACTATTGTGATATCAACCATTGAGACTGATGTCAAAATGCCAAACCTTGTATTGTCAATCCTTGCA
GCCATTATTATTGCAATAGTACTTCTTGCAGTGCATCCTATTTCTGGAGGCCACATCAACCCAGTTATCTCCTTC
TCGGCTGCCCTAGTTGGGTTGATCTCTGTGTCGAAAGCCTTAATCTACATTAGTGCACAATGTCTTGGAGCCGCG
CTAGGTGAGGGTTAAACTCCCTATCGACGAACTTTATTTTGTTCCAAAGTAGTCCATGAAGTTCACGTTGTCTTC
CAGTTTGATGACTAAAGTTAAAATTCTTGAAAAATGCTACCTGTACAGATTTTTTTAACAGACAGGGTTACAGGT
GCTGAGTTGATAAGATTTTATTGGTTGGTTTAACTTTGTTTTGTTATCCCAAATGGTGTGACATCTCGTCAACGT
AGCATTCTGTTAAAACAGTTTGTACAAGTAGCATTTCTCCAAATTCTTTAGTTTTTCTAATAGAATGTTTGGCTT
AGATCAGATGATCAGGTGAAACAAAAAGCAAACTCTAGTTATCACTCTAAAGCAAAGAAATGTTCGATAAATAAT
ATGAAACCTCACTATAAATTGAGTGACTAACAATGTTTTTTTACGCGACTACAGGTGCACTAGCTCTTAAAGCTG
TGGTTAGTAGCACCATTGAACAAACCTTCTCACTTGGAGGCTGCACCCTGACAGTGATTGCCCCTGGCCCGAATG
GGCTCGTTACAATCGGCCTCGAGACTGCCCAAGCCTTTTGGCTAGAAATAATTTGTACCTTCGTACTCCTTTTTG
CTTCAGTTTGGATGGCATATGATCATCGCCAAGCAAAGGCATTAGGTCGTGTTCTTGTCTTTTCAATTGTGGGAG
TTGTGCTAGGTCTCTTAGTGTTCATCTCCACCACAGTCACGGCCAAAAAGGGCTACGCCGGGGCGGGGATGAACC
CGGCTAGGTGTTTTGGTGCAGCGGTTGTTAGAGGTGGCCACCTATGGAATGGACACTGGATTTTCTGGGTGGGCC
CCGCCATTGCTTGCGTGGCCTTCTATTTGTACACGAAGATTATTCCACGTCAGCATTATCAGGCGAAGTTTTATG
ATCACGATTTCTTTAATGTTGTCAAGGCTATGTTT**TGA**

>*Oeu*XIP1;3_*Oeu*046122.1

ATGGCCGGTGATAATGGGCATGTACATGTAGAATATGATGAAGAAAGTTTGAGTAGTGCAAACAAAATTCAACCT
GTCTTCTCTACACCTATGCAACACTGGACAGATGAGCAGAAAAGGACGAAGAATACTTCTCTTACACTGAGTGAA
TCGCTGGGCTTGGAAGACTTCCTTTCTTTAGATGTTTGGAGAGCCTCACTGGGAGAGCTGATTGGCACAGCAGTT
CTTGTGTTCATGCTTGACACTATTGTGATATCAACCATTGAGACTGATGTCAAAATGCCAAACCTTGTATTGTCA
ATCCTTGCAGCCATTATTATTGCAATAGTACTTCTTGCAGTGCATCCTATTTCTGGAGGCCACATCAACCCAGTT
ATCTCCTTCTCGGCTGCCCTAGTTGGGTTGATCTCTGTGTCGAAAGCCTTAATCTACATTAGTGCACAATGTCTT
GGAGCCGCGCTAGGTGCACTAGCTCTTAAAGCTGTGGTTAGTAGCACCATTGAACAAACCTTCTCACTTGGAGGC
TGCACCCTGACAGTGATTGCCCCTGGCCCGAATGGGCTCGTTACAATCGGCCTCGAGACTGCCCAAGCCTTTTGG
CTAGAAATAATTTGTACCTTCGTACTCCTTTTTGCTTCAGTTTGGATGGCATATGATCATCGCCAAGCAAAGGCA
TTAGGTCGTGTTCTTGTCTTTTCAATTGTGGGAGTTGTGCTAGGTCTCTTAGTGTTCATCTCCACCACAGTCACG
GCCAAAAAGGGCTACGCCGGGGCGGGGATGAACCCGGCTAGGTGTTTTGGTGCAGCGGTTGTTAGAGGTGGCCAC
CTATGGAATGGACACTGGATTTTCTGGGTGGGCCCCGCCATTGCTTGCGTGGCCTTCTATTTGTACACGAAGATT
ATTCCACGTCAGCATTATCAGGCGAAGTTTTATGATCACGATTTCTTTAATGTTGTCAAGGCTATGTTT**TGA**

>*Oeu*XIP1;3_*Oeu*046122.1

MAGDNGHVHVEYDEESLSSANKIQPVFSTPMQHWTDEQKRTKNTSLTLSESLGLEDFLSLDVWRASLGELIGTAV
LVFMLDTIVISTIETDVKMPNLVLSILAAIIIAIVLLAVHPISGGHINPVISFSAALVGLISVSKALIYISAQCL
GAALGALALKAVVSSTIEQTFSLGGCTLTVIAPGPNGLVTIGLETAQAFWLEIICTFVLLFASVWMAYDHRQAKA
LGRVLVFSIVGVVLGLLVFISTTVTAKKGYAGAGMNPARCFGAAVVRGGHLWNGHWIFWVGPAIACVAFYLYTKI
IPRQHYQAKFYDHDFFNVVKAMF*

NIP – Nodulin-26 like intrinsic proteins

*Oeu*NIP1;1 *Oeu*060187.1

>*Oeu*NIP1;1_*Oeu*060187.1 | chr21:7140088..7142220 forward
ATGGCTGAAATGTCCGGAGCAAATGGAGTGTCCCTGAGTATAAAGGATGATGTGAACGAGAAGAGAATTCAAGAT
TCTCCAGACTGGTTGCCTTTTATACAGAAGGTACATACGAGGTTTCCTTTTATTTTCGACAACCTTAATCCTCCC
AACAGAACACAAACTGATATCTTCTATGTCCATAATTATGTGTTCAATCTTTTCTTGGAGAGACGATGAAATCGA
TGCCCTCTTAATAAATGCTACAGTTAGTGGTTTATAAAGTACATAAGATTGTCCAACCAGGCAACAGTCTTTAGT
AATCAATATTACGAAAATGATGTATAGCTGCTTAATTTGACACTGGTAATTAATGTTGAAAATCTGATTGGCCAA
CTTATTTTGGTACTGTAGATAATTGCAGAGATTTTGGGTACATACTTCTTGATTTTCGCCGGTTGTGCCGCGGTG
GTGGTGAACGCCGACAAGGACAAGATTATAACCATGCCCGGAATCGCGATTGTGTGGGGACTTGTGGTCATGGTT
ATGATTTACTCGGTTGGTCATATATCTGGTGCACATTTTAATCCGGCTGTGACCATTGCTTTTGCCTCCTGCAAG
AGGTTCCCATTGAGACAGGTTAGGTCAGCTCAAGTTACATCGATCCTTATGTACCGAATATGCTCTTAATTTCTT
TGTGTTCGGTTGTGACTCCAAACAGGTACCGGCATATATTTCGGCTCAAATGATTGGAGCCACACTAGCAGGCGG
AACCCTACGGTTATTGTTCCAAGGAAAACATGACCACTTCGGTGGAACATTTCCGACCGGCAGCGACATGCAGTC
TTTTGTTGTGGAATTCATAATCACATTCTACCTCATGTTCGTCATATCTGGAGTTGCTACCGACAACCGAGCTGT
AATTAGTCTAATTCCTTCACCAATTATTATTAAATAGTTATATTCAGTACTACTGGTATCAAGCACCAGTTTAAG
TAGCAATTGTATGACATTACATGATAGATTCATTTAAATGTATCGTCACATGATTCATTTAATTGAACTCATCAA
GTGTGGTTATTTAAACTGATGTTATAGCACCACTGATACTAGATAACATTATTCATTATTATTAGTCCAAAAATT
TATTTATAATATATATCGTGTTCTTAGGATAATTCTTCCAAAAGACAACATAGAGTTCTGTACTTTTGTTAATAA
ATTCAAGAAACGGGATTAATAAGAAATCATGGTGATTTTTTTATAGGTAAAAATGGAGGAAGATAATGACATTTT
TATTAAAGCTTATCTATGTTGACAATGATATGGCCCTTGTCGATAATTTCCTTTTATGATTTTCTTTTATTCGGG
TTGCCATGCAGATTGGAGAACTTGCGGGTTTAGCCATTGGAGCGACTATTTTACTTAATGTGATGATTGCAGGGT
AAGTTCTATGCTCGATTATCTTGCTAATTTTTAATAAAGATTGTATTACAAAATCATATCTTGGGAACATTTTCA
ATTTGTGATCTATTTCGAATTCTTAAAATTCTTGCTTAATCGGTATCATGATTCTACTTAAATTTCTAATAAGAC
AAATATATTGGATTCAAAACTAAATGATGCGATAGGAATTTCTGAAGAGTAAGATAAGCCGAAGCTCTTCTAAAA
TAATAGTACGAATAAGTTAATGACTTGAGTACAGTGTGTTTTGAGTTTGGATAATGTTATTTTGGTGACGACACT
ATTGATATTAAATTATATGTTTAATTAAATACAATAAATTATGACTCATTTAATAGGATATGCTGACATATAAAC
TAGTGTTGAACACATAAATTGGTACTAGATACTATTATTCTTTAAGTTTATATTGAAATTTTATTTCAAAAATAT
CGATTATGGACTTGATGCTTGGTAGCTTTAATGGACAGGCCAGTATCTGGGGCTTCAATGAACCCAGCAAGGAGT
TTGGGCCCAGCAATAGTGTCCAATAAATACAAGGGAATATGGATATACATAGTGGGCCCAACTCTTGGGGCTGTA
GCAGGTGCTTGGGTCTACAATATCATCAGATTCACAAACAAGCCTTTAAGAGAAATCACCAAGAGTGCTTCTTTC
CTCAAGAGCTCGGCCCGCAATGGGTCCAAC**TAA**
>*Oeu*NIP1;1_*Oeu*060187.1
ATGGCTGAAATGTCCGGAGCAAATGGAGTGTCCCTGAGTATAAAGGATGATGTGAACGAGAAGAGAATTCAAGAT
TCTCCAGACTGGTTGCCTTTTATACAGAAGATAATTGCAGAGATTTTGGGTACATACTTCTTGATTTTCGCCGGT
TGTGCCGCGGTGGTGGTGAACGCCGACAAGGACAAGATTATAACCATGCCCGGAATCGCGATTGTGTGGGGACTT
GTGGTCATGGTTATGATTTACTCGGTTGGTCATATATCTGGTGCACATTTTAATCCGGCTGTGACCATTGCTTTT
GCCTCCTGCAAGAGGTTCCCATTGAGACAGGTACCGGCATATATTTCGGCTCAAATGATTGGAGCCACACTAGCA
GGCGGAACCCTACGGTTATTGTTCCAAGGAAAACATGACCACTTCGGTGGAACATTTCCGACCGGCAGCGACATG
CAGTCTTTTGTTGTGGAATTCATAATCACATTCTACCTCATGTTCGTCATATCTGGAGTTGCTACCGACAACCGA
GCTATTGGAGAACTTGCGGGTTTAGCCATTGGAGCGACTATTTTACTTAATGTGATGATTGCAGGGCCAGTATCT
GGGGCTTCAATGAACCCAGCAAGGAGTTTGGGCCCAGCAATAGTGTCCAATAAATACAAGGGAATATGGATATAC
ATAGTGGGCCCAACTCTTGGGGCTGTAGCAGGTGCTTGGGTCTACAATATCATCAGATTCACAAACAAGCCTTTA
AGAGAAATCACCAAGAGTGCTTCTTTCCTCAAGAGCTCGGCCCGCAATGGGTCCAAC**TAA**
>*Oeu*NIP1;1_*Oeu*060187.1
MAEMSGANGVSLSIKDDVNEKRIQDSPDWLPFIQKIIAEILGTYFLIFAGCAAVVVNADKDKIITMPGIAIVWGL
VVMVMIYSVGHISGAHFNPAVTIAFASCKRFPLRQVPAYISAQMIGATLAGGTLRLLFQGKHDHFGGTFPTGSDM
QSFVVEFIITFYLMFVISGVATDNRAIGELAGLAIGATILLNVMIAGPVSGASMNPARSLGPAIVSNKYKGIWIY
IVGPTLGAVAGAWVYNIIRFTNKPLREITKSASFLKSSARNGSN*

OeuNIP1;2 Oeu040934.1

Observation: 5′ extended with 2042n, generating 3 exons and 3 introns

>*Oeu*NIP1;2_*Oeu*040934.1 | chr13:1733166..1733943 forward original version
ATGTTTGTCATTTCTGGTGTAGCCACAGATAATCGAGCTGTTAGTTCATCTTCTTCATCCATTATATTTTTTATT
CATCCATGTTTAGATTTCTGATTGATTTAAAATTCTATTGTTAATTACTAATTGTCTCTATCTAATAATAACAAC
ATTCTTTGTCAACGATGCAGATTGGAGAACTCAAAGGCATTGCCATTGGAGCTACTCTTTCACTTAACATACTTT
TCGCCGGGTACGGCTGATTAATTTAGATAATTGTCCCGTAGCTTAATTTCACACCAGATTGATTCAAGTTCCGAT

GAATCAACTACTATGAATATTGAGTACGATGAGTTTTGATTAAATGAAGATGAATTTGAAAAAAATAAATGTGTC
ATTTTTTTAATATTGCATGTTTTTAGATGAGATAGTTTAACAAACAATTTCTTGAATACTTTCATCACACATTCG
AATTTGATACACGATACTCGACATGATTGAAAAATATTAACTCTAAGGCTTGTTCTGAAATGACAGGCCAATTTC
AGGGGCATCACTGAATCCAGCAAGGAGTTTGGGACCAGCAATTGTTTACAATCGCTACGAGGGTTTGTGGAACTA
CATTCTGGCTCCTATTTCTGGAGCCATAGCAGGTGCTTGGGTCTATAATTTTATCAGATTTACGGACAAGCCCTT
GCGTGAAATTATCAAAAGGGGATCTTCCATTAGAAACGCAGCACGAACCAACTCTACTCAAGTATCAAGAAACAA
AAAGAGGATAAAATTTTGTTTATTA**TAA**

>*Oeu*NIP1;2_*Oeu*040934.1 | chr13:1731124..1733943 forward Corrected version

ATGGACGATATGTCTCGAGTCAGTTCCAACCGAGTAGTGTCCATTAACATAGAAGATGATGATTTCAATCAAAAC
CCTCCTCCTCCTACATCCACTAACACCAGGAATTCTTCAGGCTGTTCTATTTCTGTATCTTTCGTGCAAAAGGTG
ATTCTGATTCTCCTACAAAAGTTTTTTTATCTGTTATCTACTATTATTTGTGCTAATATATATGTTATATGTA
ATTTATTCCATGCTTACAGTTGATCGCAGAGTTTATTGGAACCTACTTCCTAATATTTGGTGGTTGCGCAGCCAT
CACGGTGAACAAAAACATGGAAAATGTAGTGACACTTCCAGGAATAGCCGCCGTGTGCGGACTGGTGGTGGTGGT
CATGATTTACTCCGTTGGACATATTTCTGGAGCACATTTGAATCCCGCTGTAACCATTGCTTTTGCCACCTTCAA
GAGGTTCCCGTGGAAACAGGAAATATACATATATACGTTATACTTGAATCTCCCCTAATAGGTTTTAACAGTTGA
AAACGCCGTTTCTCATCGCTTAAGCTTTAAGATGAGTAGGCTTGCGTTTGATGAAAAATCCAAGCTTCACATGCG
GAGGCTTTGTCCTCTCTATAAAAGCTTAAGCTTCTATATGAGGTGGTTCAACACATCTATATATTGTTTTTGACA
TGATCAAACGTGGGTTTTCTCATCGTGCAGGTACCAGCCTATGTCGTATCTCAAGCTCTTGCTTCAATTGTAGCC
AGGCGCGGAGCCAGCACCCGGCGAGGGGGCGCGGCCGCGCGTGCTGGCTCTGCCGGAGTCTGCAGAGCCGCCGGG
CAGTGTCACCAGTGGTCGCCGACGGTCGCTGGAGAAGAAGAAGATGGAACCCGTTTCAATTTTTTTTTTTTGGTT
TGGGCTTTTTTTACAAAATGTTGGGCCTTTTATTATTTAGCCCAAATTCCAAACCCATTAGCCTGACCCATATTA
ATACCCATTTCAATGATTTCATTGCCCTTTCTTTCAATTTCAGAATCGTGAAGGTGAATTCGTGACTTCGTTCAA
TTCGTTGGCTGCCGGCTGGGACTGGGAGGGATCCTCTTCTAATCCTCTTCACTTTCATTTTGTGGACCGGTTCAG
AAAAATTGCTGCAGTCTGCAGACTGCAGACCGAAACTCGAACTCGATCGAAGACTCGCCTGCTCCAGCCTCCAGG
CCTCCAGTGCTCCTGCAGTCCTACTGCCGCCTGCTGCGGTGCTGCCGAGAGGCCCTAGTCACCCGACTCCGACTG
CCGACTGGTAAGTGTAAAGTGTGGTAACTCTTTTTTTTTTCCGGCCCTCTTTATTTTTTATTTTTTTTGTTCT
TTTATTGGTTTTGTTCTATTTTGATTTGGCTATTTTGATTTTGTTGAATTTTGTTCTACTTGTAAATGTCTCAAT
TTATTGGAGTTTTGAGCTTTTCAATCATGTGTTCCCTCATACTGTACCTTTAATCAATTGTCAAAACCAATGAGA
TTTAGCACTTAGCAGCAATGATTTTTAATAAGTTAAATTCTCTTTCTTCTCTCATGCGTTTTCACAAATTATACG
GAAATTTATACTTGCTAGTTGCTAAATGTAGTCAAGGATTTTGTTCTATAATTTTGACATCTACAAACCACTGGT
CGTTTATAGAGTTGTAAAGGTAGTGATGTAAGGTTAGAATGACCCAATTTTGTAGTGTAGTCAAGCGTGCATGAA
ATAGTTAAATTTTTCTTCGATTCTTGATCAAAATTTACATATTTTTGCCATTTCTTTCCTCATAAAAATTTTCTG
CAACTATAATATTAAAAAATAACAATTACGAAGGTCAACCAGTCCTAGTCCGATCGTCAACGGTCAACAGTCAAC
GGTCAACCCGCCCTAGCTGGACTTCGATCCTGGATTCGCCGCTGATTGTAGCTAGTGGAACCCTTCGACTATTAT
TTATGGGGAAACAAGATCACTTTATTGGAACCCTTTCGTCGGGATCGGATGTTCAATCCTTTATGATTGAGTTCA
TTACCACATTTTACCTCATGTTTGTCATTTCTGGTGTAGCCACAGATAATCGAGCTGTTAGTTCATCTTCTTCAT
CCATTATATTTTTTATTCATCCATGTTTAGATTTCTGATTGATTTAAAATTCTATTGTTAATTACTAATTGTCTC
TATCTAATAATAACAACATTCTTTGTCAACGATGCAGATTGGAGAACTCAAAGGCATTGCCATTGGAGCTACTCT
TTCACTTAACATACTTTTCGCCGGGTACGGCTGATTAATTTAGATAATTGTCCCGTAGCTTAATTTCACACCAGA
TTGATTCAAGTTCCGATGAATCAACTACTATGAATATTGAGTACGATGAGTTTTGATTAAATGAAGATGAATTTG
AAAAAAATAAATGTGTCATTTTTTTAATATTGCATGTTTTTAGATGAGATAGTTTAACAAACAATTTCTTGAATA
CTTTCATCACACATTCGAATTTGATACACGATACTCGACATGATTGAAAAATATTAACTCTAAGGCTTGTTCTGA
AATGACAGGCCAATTTCAGGGGCATCACTGAATCCAGCAAGGAGTTTGGGACCAGCAATTGTTTACAATCGCTAC
GAGGGTTTGTGGAACTACATTCTGGCTCCTATTTCTGGAGCCATAGCAGGTGCTTGGGTCTATAATTTTATCAGA
TTTACGGACAAGCCCTTGCGTGAAATTATCAAAAGGGGATCTTCCATTAGAAACGCAGCACGAACCAACTCTACT
CAAGTATCAAGAAACAAAAAGAGGATAAAATTTTGTTTATTA**TAA**

>*Oeu*NIP1;2_*Oeu*040934.1

ATGGACGATATGTCTCGAGTCAGTTCCAACCGAGTAGTGTCCATTAACATAGAAGATGATGATTTCAATCAAAAC
CCTCCTCCTCCTACATCCACTAACACCAGGAATTCTTCAGGCTGTTCTATTTCTGTATCTTTCGTGCAAAAGTTG
ATCGCAGAGTTTATTGGAACCTACTTCCTAATATTTGGTGGTTGCGCAGCCATCACGGTGAACAAAAACATGGAA
AATGTAGTGACACTTCCAGGAATAGCCGCCGTGTGCGGACTGGTGGTGGTGGTCATGATTTACTCCGTTGGACAT
ATTTCTGGAGCACATTTGAATCCCGCTGTAACCATTGCTTTTGCCACCTTCAAGAGGTTCCCGTGGAAACAGGTA
CCAGCCTATGTCGTATCTCAAGCTCTTGCTTCAATTGTAGCCAGTGGAACCCTTCGACTATTATTTATGGGGAAA
CAAGATCACTTTATTGGAACCCTTTCGTCGGGATCGGATGTTCAATCCTTTATGATTGAGTTCATTACCACATTT
TACCTCATGTTTGTCATTTCTGGTGTAGCCACAGATAATCGAGCTATTGGAGAACTCAAAGGCATTGCCATTGGA
GCTACTCTTTCACTTAACATACTTTTCGCCGGGCCAATTTCAGGGGCATCACTGAATCCAGCAAGGAGTTTGGGA
CCAGCAATTGTTTACAATCGCTACGAGGGTTTGTGGAACTACATTCTGGCTCCTATTTCTGGAGCCATAGCAGGT
GCTTGGGTCTATAATTTTATCAGATTTACGGACAAGCCCTTGCGTGAAATTATCAAAAGGGGATCTTCCATTAGA
AACGCAGCACGAACCAACTCTACTCAAGTATCAAGAAACAAAAAGAGGATAAAATTTTGTTTATTA**TAA**

>*Oeu*NIP1;2_*Oeu*040934.1

MDDMSRVSSNRVVSINIEDDDFNQNPPPPTSTNTRNSSGCSISVSFVQKLIAEFIGTYFLIFGGCAAITVNKNME
NVVTLPGIAAVCGLVVVVMIYSVGHISGAHLNPAVTIAFATFKRFPWKQVPAYVVSQALASIVASGTLRLLFMGK

QDHFIGTLSSGSDVQSFMIEFITTFYLMFVISGVATDNRAIGELKGIAIGATLSLNILFAGPISGASLNPARSLG
PAIVYNRYEGLWNYILAPISGAIAGAWVYNFIRFTDKPLREIIKRGSSIRNAARTNSTQVSRNKKRIKFCLL*

***Oeu*NIP1;3 *Oeu*039868.1**

Observation: 5′ extended with 792n, Intron/exon readjusted, and 3′ truncated of 246n

>*Oeu*NIP1;3_*Oeu*039868.1 | chr16:12429791..12432026 reverse original sequence
ATGGCTATGGTTTACTCAGTTGGCCATATTTCCGGTGCTCATTTTAATCCGGCTGTGACCATTGCTTTTGCCACC
TGCAAGAGGTTCCCACTTAGACAGGTGAAGATGGTATATTCAACAGAATTATATGCATTGAACGAACGTGCTCTT
GATTTCTTCGTGCTCATCCTGATAGGTACCGGCATATATTGCGGCACAAGTGATCGGAGCCACGCTAGCAAGCGG
TACCCTGAGATTACTGTTCATGGGAAAACACGATCACTTTGCCGGAACACTTCCGACCGGCTCCGACTTGCAGTC
TTTTGTTATGGAATTTATAATCACGTTCTACCTAATGTTTGTCATATCTGGAGTGGCTACAGACAACCGTGCTGT
AATTCCTTCACCTTATTCTTGGTCTTTCGAAAGACAACATAGAGTTATCTACTTCATGTTTATAAATTCAAGAAA
ACCGGATTAGCAAGAGATCATGATGGTGATTTCATAATTTGAAAGGGGTTAAGACAAATGACATCATTTTGACAA
TGATATGTTTTTTTTTATCTTGAAGACGATGAAGAAATCGATGCCTTCTTAATAAATGCTAGCTAGTAGGTTGT
ATAACTAACTGCTACTCGTAATTAATTATAACAATCTGATAGCTGATTATCTCAAAATATATGTAGGTAATTGCA
GAGGGTTTTGGTACCTACTTCTTGATTTTTGCCGGTTGTGCCGCAGTGGTGGTGAACGCCGATAAGGAGGAGGTG
ATAACCCAGCCGGGAATCGCAATAGTGTGGGGACTTGTCGTCATGGCTATGGTTTACTCAGTTGGCCATATTTCC
GGTGCTCATTTTAATCCGGCTGTGACCATTGCTTTTGCCACCTGCAAGAGGTTCCCACTTAGACAGGTGAAGATG
GTATATTCAACAGAATTATATGCATTGAACGAACGTGCTCTTGATTTCTTCGTGCTCATCCTGATAGGTACCGGC
ATATATTGCGGCACAAGTGATCGGAGCCACGCTAGCAAGCGGTACCCTGAGATTACTGTTCATGGGAAAACACGA
TCACTTTGCCGGAACACTTCCGACCGGCTCCGACTTGCAGTCTTTTGTTATGGAATTTATAATCACGTTCTACCT
AATGTTTGTCATATCTGGAGTGGCTACAGACAACCGTGCTGTAATTCCTTCACCTTATTCTTGGTCTTTCGAAAG
ACAACATAGAGTTATCTACTTCATGTTTATAAATTCAAGAAAACCGGATTAGCAAGAGATCATGATGGTGATTTC
ATAATTTGAAAGGGGTTAAGACAAATGACATCATTTTGACAATGATATGGATCTTTTTTATCAATCATTTTATGA
CCTTTCTTTTTTTTTAATTATTGTTCGTGCTGCCATGCAGATTGGAGAACTTTCGGGTTTAGCCATTGGAGCAAC
TATTTTACTTAACGTGATGTTTGCAGGGTAAATACTGTGCTCAATTGTCTTGCTATTCTTATATATATAGATACA
AAACTGATCTCCTGCTCATGCCGATGGTTGAACATCTATCTCATGTCCACTATGGGTAGGAGATCAGTTCTACAT
AAAGATATTGTATTTGACGAAAGCGATTTCAAAAGTGTCAGACAAGCCATCAAGAAAAATAATAGACTTGTTCGG
ATAGTGATAATCATAATCTTGGGACGTTTTTTGAATTTGCGATCTGTTTCGAATTTTCAAAAATTGTTTTTTATG
TTATAATTCTTCAATGTGGGCCTAATGGGTAGTTTTGATGGACAGGCCAGTATCTGGGGCTTCCATGAACCCAGT
AAGGAGTTTAGGCCCAGCAATAGTGTCAAATCGTTACGAGGGAATATGGATATACATAGTAGGCCCGACTCTTGG
GGCTATAGCGGGTGCTTGGGCCTATAATATCATCAGATTCACAGACAAACCTTTACGAGAAATCACCCAGACTGC
TTCTTTCCTCAAGATCTTGGCCCGTAACAGGCCCAATTGATAACTTAGGTCCTATTTTCTGGATTAATTAGTATA
GCTTGCTGTATTAGGTAAGAGAAAATTTCTTTCCCATTTTCTGGCTATGTGGCATTGTTATTTTACAAATTCAGT
TGTGTTCTGAATGAAACCATTCATTCATCTAATAGGGGGTGAAGCAGGGAGTGAACAGAGTAAATCTGACCTCCA
TGAGCATTTTATCGAGCAAACTGCGACGGCTAGTTTTCCAATACTGCAATGCTTCACC**TAG**
>*Oeu*NIP1;3_*Oeu*039868.1 | chr16:12429545..12432818 reverse Corrected version
ATGGCTATGGTTTACTCAGTTGGCCATATTTCCGGTGCTCATTTTAATCCGGCTGTGACCATTGCTTTTGCCACC
TGCAAGAGGTTCCCACTTAGACAGGTGAAGATGGTATATTCAACAGAATTATATGCATTGAACGAACGTGCTCTT
GATTTCTTCGTGCTCATCCTGATAGGTACCGGCATATATTGCGGCACAAGTGATCGGAGCCACGCTAGCAAGCGG
TACCCTGAGATTACTGTTCATGGGAAAACACGATCACTTTGCCGGAACACTTCCGACCGGCTCCGACTTGCAGTC
TTTTGTTATGGAATTTATAATCACGTTCTACCTAATGTTTGTCATATCTGGAGTGGCTACAGACAACCGTGCTGT
AATTCCTTCACCTTATTCTTGGTCTTTCGAAAGACAACATAGAGTTATCTACTTCATGTTTATAAATTCAAGAAA
ACCGGATTAGCAAGAGATCATGATGGTGATTTCATAATTTGAAAGGGGTTAAGACAAATGACATCATTTTGACAA
TGATATGTTTTTTTTTATCTTGAAGACGATGAAGAAATCGATGCCTTCTTAATAAATGCTAGCTAGTAGGTTGT
ATAACTAACTGCTACTCGTAATTAATTATAACAATCTGATAGCTGATTATCTCAAAATATATGTAGGTAATTGCA
GAGGGTTTTGGTACCTACTTCTTGATTTTTGCCGGTTGTGCCGCAGTGGTGGTGAACGCCGATAAGGAGGAGGTG
ATAACCCAGCCGGGAATCGCAATAGTGTGGGGACTTGTCGTCATGGCTATGGTTTACTCAGTTGGCCATATTTCC
GGTGCTCATTTTAATCCGGCTGTGACCATTGCTTTTGCCACCTGCAAGAGGTTCCCACTTAGACAGGTGAAGATG
GTATATTCAACAGAATTATATGCATTGAACGAACGTGCTCTTGATTTCTTCGTGCTCATCCTGATAGGTACCGGC
ATATATTGCGGCACAAGTGATCGGAGCCACGCTAGCAAGCGGTACCCTGAGATTACTGTTCATGGGAAAACACGA
TCACTTTGCCGGAACACTTCCGACCGGCTCCGACTTGCAGTCTTTTGTTATGGAATTTATAATCACGTTCTACCT
AATGTTTGTCATATCTGGAGTGGCTACAGACAACCGTGCTGTAATTCCTTCACCTTATTCTTGGTCTTTCGAAAG
ACAACATAGAGTTATCTACTTCATGTTTATAAATTCAAGAAAACCGGATTAGCAAGAGATCATGATGGTGATTTC
ATAATTTGAAAGGGGTTAAGACAAATGACATCATTTTGACAATGATATGGATCTTTTTTATCAATCATTTTATGA
CCTTTCTTTTTTTTTAATTATTGTTCGTGCTGCCATGCAGATTGGAGAACTTTCGGGTTTAGCCATTGGAGCAAC
TATTTTACTTAACGTGATGTTTGCAGGGTAAATACTGTGCTCAATTGTCTTGCTATTCTTATATATATAGATACA
AAACTGATCTCCTGCTCATGCCGATGGTTGAACATCTATCTCATGTCCACTATGGGTAGGAGATCAGTTCTACAT
AAAGATATTGTATTTGACGAAAGCGATTTCAAAAGTGTCAGACAAGCCATCAAGAAAAATAATAGACTTGTTCGG

ATAGTGATAATCATAATCTTGGGACGTTTTTTGAATTTGCGATCTGTTTCGAATTTTCAAAAATTGTTTTTTATG
TTATAATTCTTCAATGTGGGCCTAATGGGTAGTTTTGATGGACAGGCCAGTATCTGGGGCTTCCATGAACCCAGT
AAGGAGTTTAGGCCCAGCAATAGTGTCAAATCGTTACGAGGGAATATGGATATACATAGTAGGCCCGACTCTTGG
GGCTATAGCGGGTGCTTGGGCCTATAATATCATCAGATTCACAGACAAACCTTTACGAGAAATCACCCAGACTGC
TTCTTTCCTCAAGATCTTGGCCCGTAACAGGCCCAATTGA

>*Oeu*NIP1;3_*Oeu*039868.1

ATGGCTATGGTTTACTCAGTTGGCCATATTTCCGGTGCTCATTTTAATCCGGCTGTGACCATTGCTTTTGCCACC
TGCAAGAGGTTCCCACTTAGACAGGTAATTGCAGAGGGTTTTGGTACCTACTTCTTGATTTTTGCCGGTTGTGCC
GCAGTGGTGGTGAACGCCGATAAGGAGGAGGTGATAACCCAGCCGGGAATCGCAATAGTGTGGGGACTTGTCGTC
ATGGCTATGGTTTACTCAGTTGGCCATATTTCCGGTGCTCATTTTAATCCGGCTGTGACCATTGCTTTTGCCACC
TGCAAGAGGTTCCCACTTAGACAGGTACCGGCATATATTGCGGCACAAGTGATCGGAGCCACGCTAGCAAGCGGT
ACCCTGAGATTACTGTTCATGGGAAAACACGATCACTTTGCCGGAACACTTCCGACCGGCTCCGACTTGCAGTCT
TTTGTTATGGAATTTATAATCACGTTCTACCTAATGTTTGTCATATCTGGAGTGGCTACAGACAACCGTGCTATT
GGAGAACTTTCGGGTTTAGCCATTGGAGCAACTATTTTACTTAACGTGATGTTTGCAGGGCCAGTATCTGGGGCT
TCCATGAACCCAGTAAGGAGTTTAGGCCCAGCAATAGTGTCAAATCGTTACGAGGGAATATGGATATACATAGTA
GGCCCGACTCTTGGGGCTATAGCGGGTGCTTGGGCCTATAATATCATCAGATTCACAGACAAACCTTTACGAGAA
ATCACCCAGACTGCTTCTTTCCTCAAGATCTTGGCCCGTAACAGGCCCAATTGA

>*Oeu*NIP1;3_*Oeu*039868.1

MAMVYSVGHISGAHFNPAVTIAFATCKRFPLRQVIAEGFGTYFLIFAGCAAVVVNADKEEVITQPGIAIVWGLVV
MAMVYSVGHISGAHFNPAVTIAFATCKRFPLRQVPAYIAAQVIGATLASGTLRLLFMGKHDHFAGTLPTGSDLQS
FVMEFIITFYLMFVISGVATDNRAIGELSGLAIGATILLNVMFAGPVSGASMNPVRSLGPAIVSNRYEGIWIYIV
GPTLGAIAGAWAYNIIRFTDKPLREITQTASFLKILARNRPN*

*Oeu*NIP3;1 *Oeu*053221.1

***Observation*:**

Two sequences in tandem at the end of the scaffold 635.
The first, ***Oeu*053221.1a_*Oeu*NIP3;1** | scaffold635:392813..394288. Full length sequence.
The second, ***Oeu*053221.1b_*Oeu*NIP3;1-related** | scaffold635:402535..403011. Incomplete sequence.

>*Oeu*NIP3;1_*Oeu*053221.1a | scaffold635:392813..394288

ATGGCAAATTCACCTTCTATTATTAGCTTTTCCTCTAAACAAAATTTACCAACAGACTTGTCCATTACAGAGGAA
GCAAAGGGTTACTCCGCCGACGAATTCCCTCCAAGAGATTCTGTTTCCCCGTCGTACTACCAGAAAGTAAGTTTT
TAAACGATTGCATTTGACAATACAGTATACAAGATTTTAATGGATCATTTGTACTTGAGTTAACAGATTATAGCA
GAGATTTTGGGCACTTATGTTCTTGTTTTTGCGGGTTGCGGTGCTGCTCTAGTTGATCGAGAGAAGACATTAACA
GTTGTGGGTTTAGCAATGGTGTGGGGGTTGGCTTTGATGGCAATGATATATGCACTTGGCCATGTTTCTGGTGGG
CATTTCAACCCTGCAGTTACCATTGGCTTTGCAGCAGCTCGAAAAATTCCACTCGTTCACGTAAGAATATCATTT
GTACACGAAATTTGGATTGTTCAGTAGTATCCAATCATCTGTTTTGTAACATTGGAATCGTCTTGCAGGTCCCGT
TGTACGTTTTATCGCAGTTTTTGGGTTCAACGCTAGCATGCCTCACCCTGAAAGTACTATTCAATGACCAAGCTG
ATATACTACCCACTCTCACTCATTATTCCAGTTCAACTACTGATCTTGAAGCGATTACATGGGAATTCGTCATCA
CTTTCATTTTTATGCTCACGATACGTGGAGTAGCTTCTGATGACAGAGCAGTATGTATTTCTCGTTGTGATACCT
CTATATTTAGTTCTTGCATGGTTTTATCTAAGCGTTATTGATACTTTTCAACCATCTTCTTTCATAATCTATAAC
AGATCATTCAATTACTGATTTTGCAATTTTTGTTGATGAAATCAGAATAATGAACTGGCTGGAGTTGCAATTGGA
GCTACAGTGATATTTAATGCCCTTGTTGCAGGGTAAGACTTCCCATCCCACAAGCTTCATGCATGTTTTGCTAAG
CTTAAAAGGTTAATAAATATATAAATTTTAAAATAAATAATTTTTTTGTTGTTCATAAGGGAGATGAAAGTAAGA
AAAAAGTAACATATTTTATCTTATTTGCATAAGCAGTAAACACAAAATGATCCCACCATTCTGCACTATTCTTCT
CTTAATATGAACTTCTAGTAACGCTTTCCATCATGTAATCGAAATTGCAACACAAGAAAATAGAAAAGATAAGGG
AACCAGAATAAAGGGAGTTCTTAAATATACTTATATAGGTGAATTGAAAAAAAATGCAGGCCTATTACTGGAGCT
TCTATGAATCCTGCAAGAAGCATGGGTCCAGCAATAGTTTCTGGAGTCTATAAAAATCAATGGGTGTATGTTGTT
GCTCCCATTCTTGGAGCCATAGCTGCAAGCCTGATATACAGTCTTCTCCAGCCATGGAAGCCGGAAAAGCATAGA
GAGAGTGCTAGAAGTACATACAATGATCTATATCTACGTCCCGAGGTATGA

>*Oeu*NIP3;1_*Oeu*053221.1a

ATGGCAAATTCACCTTCTATTATTAGCTTTTCCTCTAAACAAAATTTACCAACAGACTTGTCCATTACAGAGGAA
GCAAAGGGTTACTCCGCCGACGAATTCCCTCCAAGAGATTCTGTTTCCCCGTCGTACTACCAGAAAATTATAGCA
GAGATTTTGGGCACTTATGTTCTTGTTTTTGCGGGTTGCGGTGCTGCTCTAGTTGATCGAGAGAAGACATTAACA
GTTGTGGGTTTAGCAATGGTGTGGGGGTTGGCTTTGATGGCAATGATATATGCACTTGGCCATGTTTCTGGTGGG
CATTTCAACCCTGCAGTTACCATTGGCTTTGCAGCAGCTCGAAAAATTCCACTCGTTCACGTCCCGTTGTACGTT
TTATCGCAGTTTTTGGGTTCAACGCTAGCATGCCTCACCCTGAAAGTACTATTCAATGACCAAGCTGATATACTA
CCCACTCTCACTCATTATTCCAGTTCAACTACTGATCTTGAAGCGATTACATGGGAATTCGTCATCACTTTCATT
TTTATGCTCACGATACGTGGAGTAGCTTCTGATGACAGAGCATTGCAATTGGAGCTACAGCCTATTACTGGAGCT

TCTATGAATCCTGCAAGAAGCATGGGTCCAGCAATAGTTTCTGGAGTCTATAAAAATCAATGGGTGTATGTTGTT
GCTCCCATTCTTGGAGCCATAGCTGCAAGCCTGATATACAGTCTTCTCCAGCCATGGAAGCCGGAAAAGCATAGA
GAGAGTGCTAGAAGTACATACAATGATCTATATCTACGTCCCGAGGTATGA
>*Oeu*NIP3;1_*Oeu*053221.1a
MANSPSIISFSSKQNLPTDLSITEEAKGYSADEFPPRDSVSPSYYQKIIAEILGTYVLVFAGCGAALVDREKTLT
VVGLAMVWGLALMAMIYALGHVSGGHFNPAVTIGFAAARKIPLVHVPLYVLSQFLGSTLACLTLKVLFNDQADIL
PTLTHYSSSTTDLEAITWEFVITFIFMLTIRGVASDDRALQLELQPITGASMNPARSMGPAIVSGVYKNQWVYVV
APILGAIAASLIYSLLQPWKPEKHRESARSTYNDLYLRPEV*

*Oeu*NIP3;1-related *Oeu*053221.1b_

>*Oeu*NIP3;1-related_*Oeu*053221.1b | scaffold635:402535..403011 forward
ATGGCAAATTCACCTTCTATTGTTAGCTTTTCCTCTAAACAAAATTTACCAACAGACTTGTCCATTACAGAGGAA
GCAAAGGGTTACTCCGCCGACGAATTCCCTCCAAGAGATTCTGTTTCCCCGTCGTACTACCAGAAAGTAAGTTTT
TAAACGATTGCATTTGACAATACAGTATACAAGATTTTAATGGATAATTTGTGCTTGAGTTAACAGATTATAGCA
GAGATTTTGGGCACTTATGTTCTTGTTTTTGCGGGTTGCGGGGCTGCTCTAGTTGATCGAGAGAAGACATTAACA
GTTGTGGGTTTAGCAATGGTGTGGGGGCTGGCTTTGATGGCAATGATATATGCACTTGGCCATGTTTCTGGTGGG
CATTTCAACCCTGCAGTTACCATTGGCTTTGCAGCAGCGCGAAAAATTCCACTCGTTCACGTAAGAATATCATTT
GTACACGAAATTTGGATTGTTCAGTAG
>*Oeu*NIP3;1-related_*Oeu*053221.1b
ATGGCAAATTCACCTTCTATTGTTAGCTTTTCCTCTAAACAAAATTTACCAACAGACTTGTCCATTACAGAGGAA
GCAAAGGGTTACTCCGCCGACGAATTCCCTCCAAGAGATTCTGTTTCCCCGTCGTACTACCAGAAAATTATAGCA
GAGATTTTGGGCACTTATGTTCTTGTTTTTGCGGGTTGCGGGGCTGCTCTAGTTGATCGAGAGAAGACATTAACA
GTTGTGGGTTTAGCAATGGTGTGGGGGCTGGCTTTGATGGCAATGATATATGCACTTGGCCATGTTTCTGGTGGG
CATTTCAACCCTGCAGTTACCATTGGCTTTGCAGCAGCGCGAAAAATTCCACTCGTTCACGTAAGAATATCATTT
GTACACGAAATTTGGATTGTTCAGTAG
>*Oeu*NIP3;1-related_*Oeu*053221.1b
MANSPSIVSFSSKQNLPTDLSITEEAKGYSADEFPPRDSVSPSYYQKIIAEILGTYVLVFAGCGAALVDREKTLT
VVGLAMVWGLALMAMIYALGHVSGGHFNPAVTIGFAAARKIPLVHVRISFVHEIWIVQ*

*Oeu*NIP4;1 *Oeu*047418.1

>*Oeu*NIP4;1_*Oeu*047418.1 | chr18:8000311..8001846 forward
ATGGCAATCGAAGAAATGGAAGAAGGAGTAGACACCTCAACGTTCAATGCTAAAGAAGGCTTCTGTATGTCGGCT
TCAATCGTAACTATCGTACAAAAGGTTAGATCTCGTAACATTTCACTTGAAAACTTTTGGGACGTTCTCTACAAT
CTTCGATTTTTCATATTTATGCATAACATGTCACTTTCAATCTTGTTCGGTCGAGCATGCAAAAAAATGTACTAA
ACGTAATGTGTTAGATGTTTCCCAGGTGATTGCAGAGGTGATTGGCACGTATTTTGTTATATTTTCGGGGTGTGG
CTCTGTTGCGGTGAATAAATTGTATGGTGGGACGGTCACATTCCCAGGCATTTGTGCGACATGGGGACTAATTGT
GATGGTCATGGTCTACTCCGTAGGTCACATATCCGGGGCTCATTTTAACCCTGCGGTCACCATTACCTTCGCCAT
CTTTCGCCGTTTTCCATGGAAAGAGGTACCGATTAATACAACAGACATGTTTAATATGTTAGATCGATTTCATGT
ACATACATACACGTATGGGCAATTATGATGGTTTAAGTAAAACGGGATGAGAATTGATAAGTTTGAAATGTGATA
ATATCGTGCAAGAAATTCGTGGGTTTTTGTTATCATTTATTATGTTCCATATTGCATATTAAACAAAAAGATGTA
TTGTTTTGTATTGATAATTTAAATCATAGTTATTAAACACGGATTGAACTGATTTTCTTTTAAACTGTGCAACTC
GAATCATCGTAGATACAGTAATGTTCCAAGATTTGCAGGTTCCATTGTATATAATAGCGCAATTGATTGGATCGA
TCCTTGCGAGTTGTACTTTGGCTATCTTGTTTGACATCACCTCTGAAGCTTACTTTGGCACCTTGCCCGTTGGAT
CAAATGGGCAATCTTTCGCCATAGAAATCATCATCTCCTTCCTCTTGATGTTCACCATTTCTGGCGTAGCAACAG
ATAGTAGAGCGGTATGTAACATCAAAAAACATACATGACGTATTGGTAAAATGACTTAATTTTATTATTTAGATG
CTCTAAATTTCATACTTCTGAACGAGCTGCCCACGTAATTTTCTGCTTGCTTAACCTAATTTGTTTATTTATTTT
CCTTTTGAAACATAGATTGGAGATCTTGCCGGTTTAGTCGTTGGGATGACTATAATGTTAAATGTGTTCGTTGCC
GGGTACGTTAATGTCTCAATACTGTGTTTTGTTACGGGATTGGACAATATAAAAAATAATAATTATAATAATAAA
ATCAACAATATTTTATTTTTAATTTTCTATTCGATCTGCAGGCCGATTTCTGGAGCCTCAATGAACCCAGCAAGG
AGCATAGGCCCAGCCATTGTGAAGCATACTTATAAAGGACTCTGGGTTTATATATTTGGCCCACTTATTGGGACC
ATACTTGGAGGATTTGCTTATAATTTGATTAGATATACGAATAAACCCCTTCGTGAATTAACTAAAAGTAGTTCA
TTAATCAGAAGCATATCCAAAAATAATACAACTTAG
>*Oeu*NIP4;1_*Oeu*047418.1
ATGGCAATCGAAGAAATGGAAGAAGGAGTAGACACCTCAACGTTCAATGCTAAAGAAGGCTTCTGTATGTCGGCT
TCAATCGTAACTATCGTACAAAAGGTGATTGCAGAGGTGATTGGCACGTATTTTGTTATATTTTCGGGGTGTGGC
TCTGTTGCGGTGAATAAATTGTATGGTGGGACGGTCACATTCCCAGGCATTTGTGCGACATGGGGACTAATTGTG
ATGGTCATGGTCTACTCCGTAGGTCACATATCCGGGGCTCATTTTAACCCTGCGGTCACCATTACCTTCGCCATC
TTTCGCCGTTTTCCATGGAAAGAGGTTCCATTGTATATAATAGCGCAATTGATTGGATCGATCCTTGCGAGTTGT
ACTTTGGCTATCTTGTTTGACATCACCTCTGAAGCTTACTTTGGCACCTTGCCCGTTGGATCAAATGGGCAATCT

TTCGCCATAGAAATCATCATCTCCTTCCTCTTGATGTTCACCATTTCTGGCGTAGCAACAGATAGTAGAGCGATT
GGAGATCTTGCCGGTTTAGTCGTTGGGATGACTATAATGTTAAATGTGTTCGTTGCCGGGCCGATTTCTGGAGCC
TCAATGAACCCAGCAAGGAGCATAGGCCCAGCCATTGTGAAGCATACTTATAAAGGACTCTGGGTTTATATATTT
GGCCCACTTATTGGGACCATACTTGGAGGATTTGCTTATAATTTGATTAGATATACGAATAAACCCCTTCGTGAA
TTAACTAAAAGTAGTTCATTAATCAGAAGCATATCCAAAAATAATACAACTTAG
>*Oeu*NIP4;1_*Oeu*047418.1
MAIEEMEEGVDTSTFNAKEGFCMSASIVTIVQKVIAEVIGTYFVIFSGCGSVAVNKLYGGTVTFPGICATWGLIV
MVMVYSVGHISGAHFNPAVTITFAIFRRFPWKEVPLYIIAQLIGSILASCTLAILFDITSEAYFGTLPVGSNGQS
FAIEIIISFLLMFTISGVATDSRAIGDLAGLVVGMTIMLNVFVAGPISGASMNPARSIGPAIVKHTYKGLWVYIF
GPLIGTILGGFAYNLIRYTNKPLRELTKSSSLIRSISKNNTT*

*Oeu*NIP5;1 *Oeu*020545.1

>*Oeu*NIP5;1_Oeu020545.1 | chr18:6958376..6972407 forward
ATGCCGGAATTCGAAGTCGAAACTCTGACAATGTCGGCGCCAGCGACGCCTGGAACGCCGGCGCCACTGATTTCT
TCTCTGCGAATAGACTCAGTTTCTTACGATTGGAAGTCAATGCCACGATGCAAGTGCCTGCCAGTTGATGCACCC
ACTTGGGGTGCACCACATACGTGCAGCCTCACCGATTTCTCTGTGCCTGACATCCCTCTTACTCGCAAGGTTAGA
CTTTACACTCTCTCTATCTCTCTCTCTCTCTCTCACACACAATATAATATGAATATATATTTTGTTTTTTTAAAT
TTATTATGTGGAGTGAGTGGGGTCCTTAACCTTTCAGTTGCCTTTCAAATTTTCTGTATTGAACAATGATGAGTC
GGTTGAATGAATTTATAATTGAGTTTTGATACTCAAACAGCTGCTGTCACGGCCCTGTCACAGTAGCGTAACCCT
TTATAATCACGGAAATTATTGTCATTTCTATATCATCGTGACATTCTATCATTTTTTTTACAAATTTTAACACTC
TTGTGACGTGGTGTAGTGATAAGCTACATAGCATTTATGTGAAACATATGTTGTAGTATGACTATCAATTAATTA
ATTAATTTTTTTTTTTTTTTTTTTTCAAAATGTTGTGAAACCTGAAACCAAATTGAATTCAATGATTCAATTGAA
ACAAAATTGAAGTTAATTAACCCAATTGAACAATAATTGAAATTTGATTGCCCAATTGAGAATCTGAGATGATGT
GAAAGTTNNNNNNNNNNNNNNNNNNNNNNNNNNNNNNNNNNNNNNNNNNNNNNNNNNNNNNNNNNNNNNNNNNNN
NNNNNNNNNNNNNNNNNNNNNNNNNNNNNNNNNNNNNNNNNNNNNNNNNNNNNNNNNNNNNNNNNNNNNNNNNNN
NNNNNNNNNNNATATATATATATATATATATATATATATAGATGATAATTTGGACCAGTCTGGGCCCGCATATTGGCAG
GTCCGTGGGCTGGGCCTCACCTTAATCGGACCTAATTGTACCCTACACATCCATTTTGAAACTATTGCTTCCCGT
TGTCTCTTCTATTATCTTTATTCATTATTGGATAGTGATAAGTTAATGAGGGGAAAAATTCATAATTACAAATGT
TTTGAAATATAATTTCAAAAATAACAAACTCGTTTAAAAAAAATTCAAAAATCAAGTGATCACATTTTATGTTAT
TACGTATAATTTAATTATACGGGTCCCAATAATTTATGGTCATGTTCTCACACTAATCACAAATAATACGAAGTC
ATGGTCATACAAGACAAAAAAATGTGTGCTACAATTTCATATGTAGTCGCATTGCCCACATTTTATAGGTCATAA
ACTAATTATTAGTCACAAATCATCCAATAACTGGCTACAAAACATTCAATTAATTGTTTACAAATACTATTCACA
TCATCTCACTCCACATCATCCTATCCCATCGCTTCTCTCTAATTTAGGGATATCAACAAGTCGGGTCGGATTAGG
ACAACTATTCTCTTCTCTCGTCTTGTTGAGTAGTTTTATTACCTTACCCCGTCTTCATACACAGTTAAGGCGAGG
TTGGACAAGCTGGCTCCTTGTCCTATTTATATTTTATTTTTTTCATATCTTTAATTGGTAAATATTATTATCATT
ATTTATTTTTTTTCAATTTGTTAATAACACCCATGTTTCCTCTAAAAACGATGATGATATTTTTCTTTTTACATG
ATAAAAGTTTAAATTTGAAATGAATTTTAGACAAGCAGGATTTATGTTCCCCAAACCAACTCGGGGCAATGACCC
TCACTTGTCCCCCACCTCATGTACTAAGCCCCGTTTGATTATCGTTGTTTGATCACAAAAATAAAAAATATAATC
GCTATTTATTGTATTAAGTTACTATATTTGACTTTTTATCTTTTCGATAGTTTTCATTATCATTCTTACAAAAAT
AATACCAAATGGGACCTAAGTAGGGAACCTCCATGCATTTGGGGCGAGGTCCAAGCGTATAAGCTTTTTTGCCAT
CCTTAATCTCATTCTTCACCCGCTTTCTCCTCCGAACTACCACCGGCAGCCACTGAGTTTTTTTCTCACCACGGT
CGACAATCACAGAAAAAATAAGTCTACAATTACCGGCAACAACCATAGAAAAACAGATATCCAGTCAATCACAAC
ATCCACCTCATAGTGACGACGCTACCACAACTATAGAAAGACAGATCTACAGCCACATCACAACACCCACCTCAT
CGCGGTGGCGCCACCAACATCATCCCGCCATTTCACCCTTTCCACAACGTCTCTTCCATTTTTTATTTTCGCATC
TGCATTCTACACAACTGACGGCACAGACACAAAATCACCACCAACCACGTTGATAGAGTAGGAACCTGCACGGGC
GCTGTCGATCTAAAAGTCAACACCACGGAAGACGGAGCGGCAGAAATGAGTAAATATGTGAAATTTTAGGATTAG
AGTTTAGTTATTTGGGATAAGCGGAGTAAATCTGTGACGAATTTAGGGGGCAAAATGGGAAACTAAAGAGTTTAG
AGAAGATAGTGGTGGCACTTGAGGATGGCCATAGCTTTTTCCACAATTAGCCCTTGTTTGAGCCATATTATTTTT
TTTAGAATGCATTTTTGAATAATGTATTGATGAAAAATGTGTGATTAAAAAATAAAATAAAATTTTGAATTTGCG
TGTAATATTATTTTATTGAAAAATGTGCGATTGAAATGATATTAAATCACTATTTAAGAATATATATATTTTTTTTT
AAATGTGGCTCAAACATACCTTGACACCGTCTGACATAGAAATCCGTTATAAATGTGAGTAGGTGAGGGTAATGT
GTATTTGTTAATGGAGGAGAATATTGGAGTTAAAAAATCAAATTGTGCAGTTTTGAAGTTCTATAATGATTTATG
CAAGGATTAAGATGTTTGCGCCGAGGGGGTAATCGAACTGTGTCCTCCCTTCACAAACATGCAGGCCCCGCCCCT
TATTAAATAATTCAAGCCGTGGTTTCCACCTCATGTTGTGTGTAGTAGATAAAAGGTTATATAGATGATTATGTA
CGTGTAGTATGACCCTTTATTTAGGATTAGAATTTAGTTATTTGGGCTAAGAGGAGTAAATCGTGAGCTCACGAG
CCACGAGCTGCTCGCGAACTCGTCACTCCACATCATCCTATCCCATCGCTTCTCTCTAATTTAGGGATATCAACA
AGTCGGGTCGGATTAGGACAACTATTCTCTTCTCTCGTCTTGTTGAGTAGTTTTATTACCTTACCCCATCTCCAT
ACACAGTTAAGGCGAGGTTGGACAAGCTGGCTCCTTGTCCTATTTATATTTTATTTTTTTCATATCTTTAATTGG
TAAATATTATTATCATTATTTATTTTTTTTCAATTTGTTAATAACACCCATGTTTCCTCTAAAAACGATGATGAT
ATTTTTCTTTTTACATGATAAAAGTTTAAATTTGAAATGAATTTTAGACAAGCAGGATTTATGTTCCCCAAACCA
ACTCGGGGCAATGACCCTCACTTGTCCCCCACCTCATGTACTAAGCCCCGTTTGATTATCGTTGTTTGATCACAA

AAATAAAAAATATAATCGCTATTTATTGTATTAAGTTACTATATTTGACTTTTTATCTTTTCGATAGTTTTCATT
ATCATTCTTACAAAAATAATACCAAATGGGACCTAAGTAGGGAACCTCCATGCATTTGGGGCGAGGTCCAAGCGT
ATAAGCTTTTTTGCCATCCTTAATCTCATTCTTCACCCGCTTTCTCCTCCGAACTACCACCGGCAGCCACTGAGT
TTTTTTCTCACCACGGTCGACAATCACAGAAAAAATAAGTCTACAATTACCGGCAACAACCATAGAAAAACAGAT
ATCCAGTCAATCACAACATCCACCTCATAGTGACGACGCTACCACAACTATAGAAAGACAGATCTACAGCCACAT
CACAACACCCACCTCATCGCGGTGGCGCCACCAACATCATCCCGCCATTTCACCCTTTCCACAACGTCTCTTCCA
TTTTTTATTTTCGCATCTGCATTCTACACAACTGACGGCACAGACACAAAATCACCACCAACCACGTTGATAGAG
TAGGAACCTGCACGGGCGCTGTCGATCTAAAAGTCAACACCACGGAAGACGGAGCGGCAGAAATGAGTAAATATG
TGAAATTTTAGGATTAGAGTTTAGTTATTTGGGATAAGAGGAGTAAATCTGTGACGAATTTAGGGGGCAAAATGG
GAAACTAAAGAGTTTAGAGAAGATAGTGGTGGCACTTGAGGATGGCCATAGCTTTTTCCACAATTAGCCCTTGTT
TGAGCCATATTATTTTTTTTAGAATGCATTTTTGAATAATGTATTGATGAAAAATGTGTGATTAAAAAATAAAAT
AAAATTTTGAATTTGCGTGTAATATTATTTTATTGAAAAATGTGCGATTGAAATGATATTAAATCACTATTTAAG
AATATATATTTTTTTTTAAATGTGGCTCAAACATACCTTGACACCGTCTGACATAGAAATCCGTTATAAATGTGA
GTAGGTGAGGGTAATGTGTATTTGTTAATGGAGGAGAATATTGGAGTTAAAAAATCAAATTGTGCAGTTTTGAAG
TTCTATAATGATTTATGCAAGGATTAAGATGTTTGCGCCGAGGGGGTAATCGAACTGTGTCCTCCCTTCACAAAC
ATGCAGGCCCCGCCCCTTATTAAATAATTCAAGCCGTGGTTTCCACCTCATGTTGTGTGTAGTAGGTAAAAGGTT
ATATAGATGATTATGTACGTGTAGTATGACCCTTTATTTAGGATTAGAATTTAGTTATTTGGGCTAAGAGGAGTA
AATCATGAGCTCACGAGCCACGAGCTGCTCGCGAACTCGTGATTTTATTTTTTTATTTTTTTTTCACATTTGTGG
ACCTTTTTTTCATAATTTTTTCATATTTTTTTATGAGCCCACGAGCTCAGCTCAAGCTTGTGGGATTGAGCCCCC
TACTCAACTCGTGCTCAGGCTCGGGCTCGTTTTATCCCACGAGCTGGGTTTTGACTAGCCGAGCGTTCGAGCCGC
TCGGCATGTTTGACATCCCTACCTTTATTCAATAAGTGGCATAATGGTTGGGGTAGGAAGTGGGGTCTAATGGAG
AAAATCTTCGGTGAGGTGATCGTATTAGCGCATGTGGAATGTCCACCCTAACATAGAACGGCAGGTGTACAATTT
TAAGTGGGTTGGTAAATTATTTTGCTGTCTTACTTTTTCTGATTTTTCCTTTTTTATTTTATATTTTTGAAAAGG
GCTAGAAACCACCCCTCGTTAAACTGGAGTAACAGAGAAGTTGTCTTGAACAAAATAGTAATAGCACTCTTAATA
TACAGTAATGTCAAAGCAAAAACACCTTCATTAAAGAAAACGATATTTAATATTGTCTAAAAATCGGAATAGTGT
AATTCAATTTATCCATTGAAATTTAGCAAAATTACACATAAGCTTCTTTTTATCTTCTACCATAACTTCAATCAA
TTGAAAAATGTATACAAACTAATAAATGAAAGGAAAATTGCGACAGTGGTCCCCTAACCTTTCACTTTTTTGCAA
ACTTTACTCAAACATTCAATTTGTGATAACGAAGATCTCCAACCTTTCAAAAATGTAGAGCAAAGGTCCAAATGG
ACGGTTTTCGATTTTTCATCCATTTGACTATTAGTTGACCAAGAACAATAAATTTTGACCAATTTTGACTAAATT
AAAAAGGGTGAAATAGTAACTTGATAATTACCATATTAATAAATAAACAAAAAAAATTAGGGAAAATAAAAAAACC
AAAAAAATTAAAAAAAAAAAAGAAAAGAAATAAAAAAATTGTTGTCTCCTCCAGCGCCCTGCCCCACTCCACTTTA
CTCCGTCTCACCATCTCTACGTCATCCCACCCTCCTTCCCTTCGAAATCAGCCACCAGCACTGCCACCTATCGTC
GATAACATCGCCAAAAAATCAATAACACGCCCTGCTGGCAACATCTCCTTCTCACACCCATCACCAAAAATCCAA
TATATATACCCATACTCCTTATACTTAATCAATTTTTATAATGCCTGTTATAAAATTATATTAGAAAAATGAACA
AATTTCTCCCAAACCTCAAGATCTTTCAACAATCGTTTATTGCCTAGATCTGCAATAAGCAATAATAAAAGGCGT
CAGCTGAGATTAAAAACTTGCCATTTTCTTCCATTTCCTTCGTGAATAAAATCAGCCTCAAAACTAGAACGGTGT
TTTCCTGTGATTTCCAACCTTAAAATCTTAACAATCAACAGATCAATTTAAAAAAATGTTGAGGTGGGGTTTTTT
GTTTTGGGTGTAGGTTTGATGGTAATGGTGGTTGTTCACCATGCAACTCTTACTCAATGGTTGGGTGGGGTTTTT
GGTTTAGTTTTGATAGTAGTGGTGGCAGTTTGTTTTTTGGTAACTGTAGCTGATCGACAGGATGTGGTAAGTGGG
GTGGCTCTAGCTATTGACATTGCTAGTTACTGTGGGGATGGGGCTGGGGCTGGGGCTGGGGTGTTTGGAGGAGAT
AACATTTTTTCTATTTTTTGGTTCTTTTTCGTGATTTTTTTGTTCATTTATTAATATGGTAATTATCAAGTTACC
ATTTTTAAATTAGTCAAATTTATTTTTCTTGGTCAGTTAACGGTCTAAGGATGGAAATCGTCCATTTCGACCTCT
GCTATAATTTTTGAAAGATTAGGGACCGTCCCTGCTACAAATTGAAGGTTAGGGTAAAGCCTGTAAAAAAGTGAA
AGGTTAGAGACTGCTACTGCAATTTTTCCTAAATAAAATTTACAATCCAAATTCATTTGTACAAAACTTAGAACT
TCATCAAACACATTTTATTTAATAAAGCTCATACCAGTGTTATATCAAAATTTTCGCTATGATGTCATTAATCTT
TTCTTTGCTGGTCATCAAAACTATAATAATAATAATGAGAAAAAAATATATCAGTCAAGTAATCATATCGTTGTG
GTCTTGAAGCATGTTTGGAAGGTGAAAATTTGAACGCTCAAGAGCTAAGTGATGAATAAACAGATTAAAGATAGT
TCATGTAGGCAAAAATATTGCGTAAGGTGTGCCACCATTATTTTAATGACGTTCTTCATTTAACGTAACCTGATC
ATCATAAATGACAATAACTGTTATATTATTTAAGAATTGAGTAAGGGTAATTATTGAGGAAAAAAAATTATCATA
TTTTAACTTTGCTAAAACATTTTAAGTTAAAGCAAAGTTAAAATATGATAATTTTATTTCACCCTTTTTTAGCAA
GGTTAAAATGTTTTAGCAAAGTTTGTCCATCTCTTACTTCAATCATTTCCCCCTTTTCTCTTTGCAAATAATTTC
TCATGTTTTTTTTTGGTAAATCCAACATTTTAATCTTCACCAACCGCATTTGTGAAATAAAATCATGATTTTTTT
TTGTCATATAATGGCATTAACCATGCAATTAACGACATCTGCTTAAGCATTAGACACTTTACGTCTTGATCTGAG
AAATTGTCTTTCTTCAGGTCTAGCAAGTTCATGGTTATGATGAGTATTGATATGAGAAATTATCCATATTCCACC
GCTAACTGCAAAACGCACCTTAAATTTGCAACCTATTCTTGTCTCTAATCTATTATATATCCTCACATCAAGTTA
CTCTCCATCCCTTCGAGACCTTTCATTTGAACATAAAAATTCTCGTCGTTTAATATTTTTTATGCAACTCTTCGA
ATATGCCCTTTGCGAATACTAAAACTTTTTCTTATAGCATATGAATTACATATACTATAAGCTTCTTCATCAAAA
CATACTTACACGTTGCGTTTGTACCCAAATTAGGGTGAATTTTTTTTAGAAATAAATTATTTCCTTATTTCCATG
TGGAAAAATTTGTTTCCTTATATTTCACGTGATTTTTATGAAAATATATTATTTCCTTATTCCACATTATTTACA
TCTTTATGCGATGACATGTACCTATAAATACTGATCTTCTCTTTCAGTTTTATTCAAACAAGAATTGTTAAGTTC
TTGTATTTCTTCCCATATTGTCGATTAATAAAAAACGTCGTGCTTTTGTGATTAACCCTCTTTGTTAATTCCTCT
ACTTTTAAATCTTGTTTCTCGTTGCAATCTTCATTACTTCATATCGCGTCATTAATCTCATTCACTACAATCCTT
TCGGGTCCAACAAATGGTATCAGAGCTTGGATCTTGTTGTGGATTGGTTAAAGTTTTTCAGAGATGGCTGCTTTG

AAGTATGATATTCCATAATTGAATCGCAATACAAGGTTTTCTTTGTGGCAAGTAAAGATGAAAGTTATTTTGGCA
CAGTTAGATTTGGATGATGTGTTGCTGGGTTTTAAGAAGATACCAGCTAATTAGACAACGGAGGAGAAACAACAA
AGGGATCAGAAGGCTTTGTCGTTGATTCATTTTCATCTATCCAACAATATTCTGCAAGATGTCTTGAAGGGAAAA
ACAGCAGCTGCTTTATGGTTGAAACTGAAGCAGTTTTGTATGATCAAGAGTCTTGCTAGGAAACTGGCTCTAAAG
CAGAAGCAGTGTTCCTATCGCGTGGCAGTAGGAGAATTGTTGGAAGACCATATTGCTGTGTTAAAGGAAATTATC
GCAGATTTGGAAACATTGGAGGTTAAGTGCGATAAAGATGATCTTGCCTTAATCCTATTATGTTCGTTGTCTGGC
TCTTATGCGTCGTTTCGGGACTCCATTTTGTATAGTCGAGATACTTTAACTATAGACGACGTCTATGATGATTTG
CACTTAAAGAAGAAGTTGAAGTATTTGGTTGGAGCTACATCGAAGGATGAGGAAGCTATTGTATCTCATGGTGGT
CGACAGAGGGGCGGATCGAAGAATAACAGCAATTTTTTTTTGCTTCTACTACATACATAAGGGGGACATATTCG
GAAGGATTGTAATAAATTGCAAGCTAAAGGTGATTTAACTACAAATCGTAGAGCGCAACCTTTGACTTCATATGA
AACAAATACAGTTGAGGATTTTTCAGACGACAGAGATGTGATGTTAATTGTAATATTTGACATGGAATCTATGCT
CGATGCTTCAACTTTAAGGTTGCAAACCCAAGTGCATGTTGTCTCAAGTAGTAAACTCGAAATTTCGAGGTCAAA
CCATAAAGAATTGGAAGGAAATAAACTATTCTATGAGTGGTGAACAGTTGGTGGAAATACGATGGAGAAGAACAC
TAGAGTGTTGAGGATTCACTTCGGATAAATGAGTAGAATTACTCTTGATCTTCTATTTAACTCATATGAAATATG
GAAGATAAAACATTGATTGCAAATATACTAAAAGATAGATCTTGATAATTATTTGAGGGAAAATATGTCAAAATA
AGTTGTTGGAATGTAGAGATTATCTAAGCATTTATTGTGTCAAAATTTGTCAACAATAAATCAAAGCCTTTAACA
AAGCAAAACTGTTTATAGAACATATACTTCAACCAAGCAAGAAGATGAATATTGAAGAACAAAGTGAAAAATCGA
AGACAATACTACGCATTCACCAAAGTTTCACCCCTAGCTCTAGCTAATGGTTTAGAAATACATAATGAAATGTTA
CAAAAAAAATGAAGGAAACTAAAATATGATGACGAAGAATTGATGCTCCAAAATCTTGGCCAAGGTCCCTTTTAC
AGGCAAAGAAATATCCAAAATGCAAGTGGACAATGCAACTTGTTTCTGTCCAGTTTTAAGACAGTTAGAACATGC
AACTTGATGCCAATTTTCAGATACTTTGTGCAATGAATTTAGACTTATGATCGATAGAAAAATTGTTTCCACTTG
AGTTAGCTTTCCATTGGTGTAAATATCAGGTCCATAGGATCTATATAACTCTAGATATGGCCAAAACAATGAGTG
CTGCTTCACAGGTAGAAAAGTTGTATCCGGTAGGTAACAAACTGGACTTTACACAGTAATTTTGGCTACCTTATG
ATACAAATTTGACTTCTTGGTAAGCATAAAAGTTGTAGATACATATCATATCTTTCTAATCCAACTTGAATCACC
TTGTTTCGATAGCTACAACTCGAGTTATGACTATTTTACCGAGACAGCTGTATCTTGCTCAAGACAGCTAATCAT
TTTTTTACTTTTCAACATATTTTCTCCACTTGCTCCACTTGTTCTATCTTGGAAATACATGTGAAATATATCGAA
TAAATACTCTTTCAAAAGTATAATACTCATGCCAATATGTATTTAAATACGTATTTCAATTAATTGCATTTTTAA
CCATGTATTTATGGAAATTGAATTTACAACAAGAGAATTGAAATTAAAGACTAACATTAGTGAATTTAAACAAAT
TGAATGTAAAATGTCTTGTAAATTGTATATCTTTGAAGCATTTTTCGACAATAATCACCTGACGAGGAGTGAATC
CTTGATTCGACATGTTCGCATCATATATTTTCCAATCAAAATTTCTTTCAGACATATGAGGCTGTCAAGTAAGGT
CGAGTTGTGATAGGAAACAACCAGTCTTGTCGTGTTGATGAAATCGGTCGAGTCAGATTAAGACTGCGAGATGGG
ACAGTCAGAACAATTGATCAGGTTCTGCATGTTCCAGACCTGGAAAAGAATATCATCTCACTTAGTACTTTGGAT
GCAAAATGGTATAAATTCTCTAGTGGGAGTGGGGGTGGGGTGGGGTTCTGAAGGTTCAAATGGGAGTCCGAATTA
TATTGGAGAAACAGAAGAAGTCTCAGTTGTGTATCTTGCTGGAAGCGCAGAACTAGTTAAAAGATATCGAGACAA
GTTCAGTACTGTGGTAGATATTCAGTTTGGTACAGTACCTCTTATGACGAGCTCTGGGTCAGTTTAAAACATTGA
TCGGGGGAGCATTGGCGATTCTACTTTTCCACCAATTTCAGCAGATACACTGTGTTCATTTGCTAGGGTTGGACT
CATGGAGCGTAGATACCGTGGTGTCAATAATGTTTGGGATGCTTTTGGGGACGTTGAAGGCGACAGTTCTAGCAA
TGGGCTTTCTGATCCAATGTGTTGTATGGTTTCTGCTACAGGATGTGCTTCGACTCTATTTGGTCAATAGTAATC
CCTCAGTGAAATTTTGGAAACCTGAGATTTGGGTTCAAATGTCGTTTATGGACGCCTTGCAGAAGACGACGATTT
GGTTTGGAGAGTTTTGGTTACTGTGTTGAAGAGATTGATTATTTCGATGCAAACGGATTTGAAACCTTGGAATTT
CCTTTGTTATACTAGAATTTGGGTCAAGGCGGAGTTTGTTGGGTTTGTTACCCAAATTAGTGTTTTTTTTTTTTT
TAAATAAATTGTTTCCTTATTTTCATGTGATTTTTATGAAAATATATTATTTCCTTATTCCACATTATTTTCATC
TTTATACTATGTCATGTACCTATAAATAGTGTTCTTCTCTTTCAATTTTGTTCAAACAAGAATTGTTGAATTCTT
AAATTTCTCTCTATATTGTCGATTAATAAAATTTCATGCTTCTGTGATTAACTCTCTTTGTTAATTTCTATACTT
TTAAGTCATGTTTCTTATTGCAATTTTCATTACTTCATATCGTGTCATTAATTTCATTCATTACAATCCTTCCGA
GTCCAACATTACATTCCTATATTAAGTACTTCATAATTTTTTTCTCTACTACTTAAATGCTCCATTGGATAGTAA
AATATTAAAAATCCATAGTAATATCTAGGAGTGTCAACGAGTAGAGCTGAAATTCTTGGCTCGGGCTCAGCTTGA
ACTTTTTAAGACGGGCTTTTTTTTTTGCTCAAGCCGAGCCAGACGAAAGCTCCGCCAAAAAAATAAGGCTCAACC
CGAGCTTTCGACTCGGGCTAGCCGAGCCAAGCTCAGTTTTTTGGAATTTTTTTAATTTTTTTTCCCTAAAAGTGG
CATTTGCTGCCTGTTTGGCAGCTAAATATAGGCTGCCTTTTCTAATCCCTTTTTTTACGAGCCGACCCGACTTGA
GCCGACTCCCCAAAATGGGGCTCCGTCCGAGCCAAGCCGACTCGGGCTTTCCCCAGCCCAAATTTCAGGCAATCT
GAGCCGGCAAGCCCGATTGTCATCTCTAGTAATACCCATTCAACCCACTTTAATATCAACAAAATAAATACAATA
AAAGATTATTTCACTTTCCCATTTACATTACAAAATTCTAGAAAAATGAAATTAATAAATTAAAATGCATATATG
ATTTTGCTGGGTTGCAAGTGTCCAGAAAGCATGGCAACAAGACTCTAAAGAGAGAGAGAGAGACAGAGACAGAGA
GAGAGAGGGGCTTCTCTAGAAGACGAGTCTAATTTTAAAGGGGAAAAAAAAAGTGGGACCATAAATTTTGGATGG
AGCCCAATGTATTTTATTTTTTTTTAATTTAAAATATAAACATTTGTCAAAGGTTGCTTAAAATTGTACACGTGT
CATTCTGTGATAGGGTGGACATTCTGCATGGGTGGTACGACAACCTCACCCAAGATTTTCTCGATGTAATGAGAG
AGATTAATTATTCAATTATGCTACAAATACACCTATTTTTACATCATTTTTGACAGCCCTCACACGATACAAGTG
GGCCCACGATTAAAATAATCGTGAGACTCACTCGTGTGAGTGGGCTGTCAAAGTGAGTGTACAGCAGATGCTGTA
CACTCAGCTTAATTCTTAATTATTACAGGACAAATTGTACAACTTTTACAGGATTATCTCCATTGCTGCAATAGT
TAGGATAGAGGTGGGATAATCGATATATGTTTTTTTGGGGGAAAAAAATAATAGCACCTAGTTTATGAAATTGAA
TATTGAAATGTAGGGGGTTTTGAGGTAATTTTCCCTTGAATGAATTTATAATTATACTTGCCTTGGAAGTTCCAA
GAGTACTTCATTAATTTCTACTAAAATTTTAATGAAAAACTAGTATATTAATTGATAATCTGTACCTTTCGGTTG

TAAAAATTAGGAACTATACTACCACTTAATCATTATAAAACCTCACAGAAATCCAATAATATTTATCAAAAATAA
AATAATTAACTATAATCTGGTGTTAACTGTAGCTGGGAGCAGAGTTTGTTGGCACATTTATCCTGATATTTGCTG
CAACGGCCGGACCAATCGTAAACCAAAAGTACAATCAAGCCGAAACACTTATAGGCAACGCAGCATGTGCTGGTC
TCGCAGCTATGATCGTGATTTTATCAACAGGTCACATCTCGGGTGCTCATCTTAATCCGTCACTCACTATTGCTT
TTGCTGCAGTTCGCCACTTCCCATGGGCTCATGTACCAGGCTACATTGTAGCACAAATTTCAGCCTCCATTTGTG
CTTCCTTTGCGCTCAAAGGTGTTTTCCATCCTTTTATGTCCGGTGGTGTTACTGTCCCTTCTGTCAGCATAGGCC
AGGCTTTTGCCCTTGAATTTCTTATAACCTTCAATCTGCTATTCGTCGTTACTGCTGTTGCCACTGATACCCGCG
CTGTGAGAAATCAATTGAACATGAACATTCTTTTCTTCATATATTACAAGTAACTAATCGCCATCTGAATTAATC
CTTGTTGATAGGTTGGAGAATTAGCAGGCATAGCAGTTGGAGCCACTGTCATGCTTAATATTCTTGTTGCGGGGT
GAGTAAATGATTCTTTTGCTACGTCGTCTTCTTCTTGAAAGTAATAGTAAATGAAAAGGGTGATGGAAAAAGGAC
GCTAGCTAATTGTTTTTACTCTCTTTGAATTGGCAGACCATCGAGTGGTGGCTCGATGAATCCTGTCCGAACTCT
TGGACCAGCAGTGGCAGCAGGGAATTACAAGGCAGTCTGGATATACCTTTTGGCACCTACACTTGGAGCTCTTGC
AGGGGCTGGCATTTATACTCTAGTTAAGCTTAACGGAGAAGAGGAAGAAGCCCCAAGCCAGGCGAGGAGTTTCCG
TCGC**TAG**

>*Oeu*NIP5;1_*Oeu*020545.1

ATGCCGGAATTCGAAGTCGAAACTCTGACAATGTCGGCGCCAGCGACGCCTGGAACGCCGGCGCCACTGATTTCT
TCTCTGCGAATAGACTCAGTTTCTTACGATTGGAAGTCAATGCCACGATGCAAGTGCCTGCCAGTTGATGCACCC
ACTTGGGGTGCACCACATACGTGCAGCCTCACCGATTTCTCTGTGCCTGACATCCCTCTTACTCGCAAGCTGGGA
GCAGAGTTTGTTGGCACATTTATCCTGATATTTGCTGCAACGGCCGGACCAATCGTAAACCAAAAGTACAATCAA
GCCGAAACACTTATAGGCAACGCAGCATGTGCTGGTCTCGCAGCTATGATCGTGATTTTATCAACAGGTCACATC
TCGGGTGCTCATCTTAATCCGTCACTCACTATTGCTTTTGCTGCAGTTCGCCACTTCCCATGGGCTCATGTACCA
GGCTACATTGTAGCACAAATTTCAGCCTCCATTTGTGCTTCCTTTGCGCTCAAAGGTGTTTTCCATCCTTTTATG
TCCGGTGGTGTTACTGTCCCTTCTGTCAGCATAGGCCAGGCTTTTGCCCTTGAATTTCTTATAACCTTCAATCTG
CTATTCGTCGTTACTGCTGTTGCCACTGATACCCGCGCTGTTGGAGAATTAGCAGGCATAGCAGTTGGAGCCACT
GTCATGCTTAATATTCTTGTTGCGGGACCATCGAGTGGTGGCTCGATGAATCCTGTCCGAACTCTTGGACCAGCA
GTGGCAGCAGGGAATTACAAGGCAGTCTGGATATACCTTTTGGCACCTACACTTGGAGCTCTTGCAGGGGCTGGC
ATTTATACTCTAGTTAAGCTTAACGGAGAAGAGGAAGAAGCCCCAAGCCAGGCGAGGAGTTTCCGTCGC**TAG**

>*Oeu*NIP5;1_*Oeu*020545.1

MPEFEVETLTMSAPATPGTPAPLISSLRIDSVSYDWKSMPRCKCLPVDAPTWGAPHTCSLTDFSVPDIPLTRKLG
AEFVGTFILIFAATAGPIVNQKYNQAETLIGNAACAGLAAMIVILSTGHISGAHLNPSLTIAFAAVRHFPWAHVP
GYIVAQISASICASFALKGVFHPFMSGGVTVPSVSIGQAFALEFLITFNLLFVVTAVATDTRAVGELAGIAVGAT
VMLNILVAGPSSGGSMNPVRTLGPAVAAGNYKAVWIYLLAPTLGALAGAGIYTLVKLNGEEEEAPSQARSFRR*

***Oeu*NIP5;2 *Oeu*046374.1**

>*Oeu*NIP5;2_*Oeu*046374.1 | Scaffold476:396474..398858 forward

ATGCCGGAATTCGAAGCCGGAACACCGACATTGTCGGCGCCAGCGACGCCAGGAACGCCGGCGCCACTGATGTCT
TCTCTGCGCATAGATTCGCTGTCTTATGATCGAAAATCAATGGCGCGATGCAAGTGCCTTCCAGTTGATGCATCC
ACGTGGGGTACACCACATACCTGCAGCCTCACCGACTTCTCTGTGCCTGACATCTCTCTTACTCGCAAGGTTAGA
CCACTTTACGTTACAGTTAGATCTCTCTCTCCCTCCCTTGCCTCGAGCATACTTAAGTGGTTCAAAGAATAAGAG
TGAGGTATAGTTAGCCTTGATGTGAGGGTTCGATTCCTGAAACATGAATTGAAACTTATACAGACAACTTTTGTT
CCTTGACCTGTTGAGACATCCCACGATTAACTTCTCTCGTGATGCTCCAAGAACCTTGGGTTCAACTTTTTTAGA
TCTCTCTCTCTCTCTCTTGAATATAATATGAACATATATGATTATTGACGTGGAAATTGGAATGGGGTCCTTACC
TTTCAGTTGCCTCTTTTAATTTTCTTTGTTGAATACAATATGAATAATGATCAGTCAGTCATTTCTTTGTCACGC
ATCCTATAAATTAAAATGGTAGGAGTATGATTATGACATGATTTTAGGTTAGAGGTTTAAATTCACATTTTTTTT
TAACGCTTCTATGATGTGACGTAACGATAAGTTAGCATTGATGCGGTGATTATTTGAAACATATGTTGTTATATT
GTTATCGAATTTTCATCAATTTAGAAGATGATAGTCCAAAATGTTGTGAAATTTGAAACAAAGCCCTTGTTCGAT
AATCATACTTTTCAAACTTTTTTTTTCTACAGACTTATTTTCAACCACACTTCCACACAATAAAACATTTTGCAT
GTAAACTCGAATTTTTTTTTTTACTTTTTAACAACACATTCTTTTAAAAATCACTATTCAAAACTCTATTTTTCT
GAAAGTATGATTTTCAAACTTGGGCTAAATTGAGTCCAATGATCTAATTGAAACAAAATTGTAGTTCATCAATCC
TATTGAAAAACAATTGAAATTCAATCACCTAATTGAGATTTTGTTGAGACTTCTGAGTTCAGTGACTGAAAATGA
AATTTAACGAAGTGAATTGTTGATGTGGAGTGGGTCCTTCGCTTAGTTGTGTCAAATGGGACCGGTCGCTCAAGG
CCCCACCTCGTTATAAACTCGTATTGAAACCAAAATGAATGAGTCATGTCGTGTTTGATTGACTAGCCCAACAGT
CCATGAATTTCGAATTAGTTTGCTCGAGTATACCTGCTGTGAAAAATGCCAAGAATCCTCCAATAATTTCTGTAA
TGAGTATCGATAGAGAATCTGTACCTTTTTGTTGTATAAACTAGGATGTACACTGAAACTTATTCATCCCAATAA
CAGAGGCAAAAACCACTCAGAAATTCGTTAAAATTTGTCAAAAATGAAGGAATTAACTATGTCAACATAATTTGG
TGTTAAATGTAGCTGGGAGCAGAGTTTGTTGGGACATTTATCCTGATATTTGCTGCAACGGCCGGACCAATCGTA
AACCAAAAGTACAATCAAGCTGAAACACTTATAGGCAATGCAGCATGTGCTGGTCTTGCCGTTATGATAGTGATT
TTATCTACAGGTCACATCTCGGGAGCTCATCTTAACCCGTCTCTCACTATTGCCTTTGCTGCACTTCGCCATTTC
CCGTGGGCTCATGTACCGGCCTACATAGCAGCGCAGGTTTCGGCCTCCATTTGTGCTTCTTTTGCACTCAAAGGT
GTTTTCCATCCTTTCATGTCCGGTGGTGTGACTGTCCCTTCTGTCAGCACCGGCCAGGCTTTCGCTCTTGAATTT
CTTATAACCTTCAATCTGCTATTCGTGGTTACTGCTGTTGCCACTGATACCCGCGCTGTGAGTAATCAATTGAGC

ACCAACATTCTTTTCTTTCTCCTTTTCTTCAGATATTACAAAGTAATCAAAATCCTTGTTGACAGGTGGGAGAAT
TAGCAGGCATAGCAGTTGGAGCCACCGTTATGCTTAATATTCTTGTTGCGGGGTGAGTTAACGATCCCTTTTTGC
TTCATCTTCTTCCTGAAAACAATGGTAATTGAAAATCGTGAAGAAAAAGGGCACCAGCTAACTGTTTAGACTCT
CTTTGAATTGGCAGACCATCGAGTGGTGGCTCAATGAATCCTGTCCGAACTTTGGGACCAGCAGTTGCAGCAGGA
AATTACAAGGTGATATGGGTATACCTTCTGGCACCTACACTTGGAGCTCTTGCAGGGGCCGGTATTTATACTCTG
GTTAAGCTTAATGGACAAGAGGAAGAAGTCCCACGCCAAGTGAGGAGTTTCCGTCGC**TAG**

>*Oeu*NIP5;2_*Oeu*046374.1

ATGCCGGAATTCGAAGCCGGAACACCGACATTGTCGGCGCCAGCGACGCCAGGAACGCCGGCGCCACTGATGTCT
TCTCTGCGCATAGATTCGCTGTCTTATGATCGAAAATCAATGGCGCGATGCAAGTGCCTTCCAGTTGATGCATCC
ACGTGGGGTACACCACATACCTGCAGCCTCACCGACTTCTCTGTGCCTGACATCTCTCTTACTCGCAAGCTGGGA
GCAGAGTTTGTTGGGACATTTATCCTGATATTTGCTGCAACGGCCGGACCAATCGTAAACCAAAAGTACAATCAA
GCTGAAACACTTATAGGCAATGCAGCATGTGCTGGTCTTGCCGTTATGATAGTGATTTTATCTACAGGTCACATC
TCGGGAGCTCATCTTAACCCGTCTCTCACTATTGCCTTTGCTGCACTTCGCCATTTCCCGTGGGCTCATGTACCG
GCCTACATAGCAGCGCAGGTTTCGGCCTCCATTTGTGCTTCTTTTGCACTCAAAGGTGTTTTCCATCCTTTCATG
TCCGGTGGTGTGACTGTCCCTTCTGTCAGCACCGGCCAGGCTTTCGCTCTTGAATTTCTTATAACCTTCAATCTG
CTATTCGTGGTTACTGCTGTTGCCACTGATACCCGCGCTGTGGGAGAATTAGCAGGCATAGCAGTTGGAGCCACC
GTTATGCTTAATATTCTTGTTGCGGGACCATCGAGTGGTGGCTCAATGAATCCTGTCCGAACTTTGGGACCAGCA
GTTGCAGCAGGAAATTACAAGGTGATATGGGTATACCTTCTGGCACCTACACTTGGAGCTCTTGCAGGGGCCGGT
ATTTATACTCTGGTTAAGCTTAATGGACAAGAGGAAGAAGTCCCACGCCAAGTGAGGAGTTTCCGTCGC**TAG**

>*Oeu*NIP5;2_*Oeu*046374.1

MPEFEAGTPTLSAPATPGTPAPLMSSLRIDSLSYDRKSMARCKCLPVDASTWGTPHTCSLTDFSVPDISLTRKLG
AEFVGTFILIFAATAGPIVNQKYNQAETLIGNAACAGLAVMIVILSTGHISGAHLNPSLTIAFAALRHFPWAHVP
AYIAAQVSASICASFALKGVFHPFMSGGVTVPSVSTGQAFALEFLITFNLLFVVTAVATDTRAVGELAGIAVGAT
VMLNILVAGPSSGGSMNPVRTLGPAVAAGNYKVIWVYLLAPTLGALAGAGIYTLVKLNGQEEEVPRQVRSFRR*

*Oeu*NIP6;1 *Oeu*060126.1

>*Oeu*NIP6;1_*Oeu*060126.1 | Scaffold830:250629..252602 forward

ATGGGCACTAAAGATGTTGCATCAGTTCCTACAATGTCTGTAACGCCCGGGATTCCGTGCTGCACTCCTCTTGTC
GATGGCCAGTTCAAGCCGGAAAGGTGTGGCGATAGCCGGAAATCCCTTTTTAAGAGCTGCAAGTGCTTTAGTGTT
GAAGCACTTTCTTTGGAAGAAGGGACCTTACCCTCTGTCTCTTGTGCATTGATTCCACATCCAATCTCCCTTGCA
AGAAAGGTGACCTAGTTCTTCCCTCAACCTAGTCAATTTTGTTAAACCTAAGTTCTTGGTTCAAGTCTTGCCGCC
ATTAATGTATAATGCATTTATATCTTGAACAACGTCTAAACCAGGTGGGAGCTGAGTTTATAGGTACCTTCATAC
TCATTTTCACAGGCACAGCCACAGCGATTGTGAACCAAAAGACACAAGGCTCGGAGACCCTTCTCGGCTTGGCAG
TCTCCACCGGCCTGGCAGTCATGATAGTTATACTCTCCACGGGTCACATCTCCGGGGCACATGTCAATCCATCTG
TCACCATTGCCTTTGCTGCTCTGAAGCATTTTCCATGGAAACATGTAAAATGACTCTTAAAACAACTCTACAACC
AGTAGTGAATAACAAAACAATTTTTTCTTTTCTTGCTTAATATTGTCCAGTCTATCCCGTCAATGTTTATTGATT
TTGGCAGGTTCCAGTATATATTGGAGCACAAATATTGGCATCAACATGTGCTGCATTCTTACTGAAGGCTATTTT
TCACCCTATAATGGGTGGTGGAGTAACTGTTCCTTCAGGGGGATTTGCTCAAGCTTTTGCTTTGGAGTTCGTCAT
TAGCTTCAACCTCATGTTTGTAGTTACTGCAGTGGCCACAGATACAAGAGCTGTAAGCATTTGCTTTACAGCTTT
CAGCTAGTTCTACACATTGACTAGAAATCACAATTGACAAATTTTCGCGAACTTTATTTCTTTTATATTATCCAA
TCTTACATACAAAAAGCATGCCCTATTTCGCACACCCTTTTATCCATTTGTTTGCAAGATAAGTGCTTCCACGAG
AATAATAGAGACCTCTGGCATAACTAGCACAAAACTTCACGATAAGTCGAGTGACTAGCTGTATTTTTAAACCAT
TAATTTTTGTCCATTGCAGGAAGTAAGAAACTAGATTTTAGCCCTGTTTGTCACCCATTATTTGGAAAATAAAGG
AGTTTTGAATAGTATTTTTCGAAGAATGTGTGTTAAAAAATAAAGAAACTTTATTAGTTTGTGTGTAGAATATTT
TAATGTATGAATATGTAGTGGAGAATGATACTGTAGCAAACAATATAAATTTTAAAGGGTGCCAAACAGGCTCTT
ATGGTAGAAGTTGGCAACTAGAAGTCCAGATTTCAACAAACATTTTATGAATATTGCAGGCAGTAGTAAACTGAC
TAGTTGTGGGAATAATCAACAACAGTTGAATATATTGAACAATTTTTTGATAGTATATATGGAATCTTCAGCATG
TTTAGCCAAGCTTAAAATGTTTGCTTATAAGTAAATAAGTATATCTTAAAATAAATAACTATTTTTTCCTCTCTG
AACAGGTAGGGAAGCTTGCAGGAATTGGGGTTGGAGCTACTGTAATGCTCAACATACTGATAGCTGGGTGATGCT
CTTAACTCTCCTTTAATTTCAGCACAATTCCTCCTATAAACTTGCTATATTCGTTCGAACAAAACTATGCTCTGA
AAAGAGCTTGATAGAAACATAAACCTTGTCTATTCATCCAGGGAAACAACTGGAGCATCAATGAATCCCGTGAGA
ACTCTGGGGCCAGCAATAGCTGCAAACAACTATAAAGCCATATGGATTTACCTCACTGCGCCTTTCCTCGGTGCT
CTGGCCGGGGCAGCTGCTTATTCTTTGGTTAAACTGCCAAACCGAGATGTCAATGGACACGAGAAACTGCCTAAT
GCAGAACATAGCTTCAGGAGA**TGA**

>*Oeu*NIP6;1_*Oeu*060126.1

ATGGGCACTAAAGATGTTGCATCAGTTCCTACAATGTCTGTAACGCCCGGGATTCCGTGCTGCACTCCTCTTGTC
GATGGCCAGTTCAAGCCGGAAAGGTGTGGCGATAGCCGGAAATCCCTTTTTAAGAGCTGCAAGTGCTTTAGTGTT
GAAGCACTTTCTTTGGAAGAAGGGACCTTACCCTCTGTCTCTTGTGCATTGATTCCACATCCAATCTCCCTTGCA
AGAAAGGTGGGAGCTGAGTTTATAGGTACCTTCATACTCATTTTCACAGGCACAGCCACAGCGATTGTGAACCAA
AAGACACAAGGCTCGGAGACCCTTCTCGGCTTGGCAGTCTCCACCGGCCTGGCAGTCATGATAGTTATACTCTCC

ACGGGTCACATCTCCGGGGCACATGTCAATCCATCTGTCACCATTGCCTTTGCTGCTCTGAAGCATTTTCCATGG
AAACATGTTCCAGTATATATTGGAGCACAAATATTGGCATCAACATGTGCTGCATTCTTACTGAAGGCTATTTTT
CACCCTATAATGGGTGGTGGAGTAACTGTTCCTTCAGGGGGATTTGCTCAAGCTTTTGCTTTGGAGTTCGTCATT
AGCTTCAACCTCATGTTTGTAGTTACTGCAGTGGCCACAGATACAAGAGCTGTAGGGAAGCTTGCAGGAATTGGG
GTTGGAGCTACTGTAATGCTCAACATACTGATAGCTGGGGAAACAACTGGAGCATCAATGAATCCCGTGAGAACT
CTGGGGCCAGCAATAGCTGCAAACAACTATAAAGCCATATGGATTTACCTCACTGCGCCTTTCCTCGGTGCTCTG
GCCGGGGCAGCTGCTTATTCTTTGGTTAAACTGCCAAACCGAGATGTCAATGGACACGAGAAACTGCCTAATGCA
GAACATAGCTTCAGGAGA**TGA**

>*Oeu*NIP6;1_*Oeu*060126.1

MGTKDVASVPTMSVTPGIPCCTPLVDGQFKPERCGDSRKSLFKSCKCFSVEALSLEEGTLPSVSCALIPHPISLA
RKVGAEFIGTFILIFTGTATAIVNQKTQGSETLLGLAVSTGLAVMIVILSTGHISGAHVNPSVTIAFAALKHFPW
KHVPVYIGAQILASTCAAFLLKAIFHPIMGGGVTVPSGGFAQAFALEFVISFNLMFVVTAVATDTRAVGKLAGIG
VGATVMLNILIAGETTGASMNPVRTLGPAIAANNYKAIWIYLTAPFLGALAGAAAYSLVKLPNRDVNGHEKLPNA
EHSFRR*

*Oeu*NIP6-related *Oeu*011481.1

Observation: Incomplete at the 3' end

>*Oeu*NIP6-related_ Oeu011481.1 | scaffold14354:165..1696 reverse

ATGGATGGTGAGGATATTGCATCAGCTCCTTCAACACCTGTAACACCAGGGACTCCAGGTGCTCCTCTCTTTCGA
GATTTCAAAAGGGACAATAATTGCTTTAATGGTAGAAAATCATCTCTTCTTAAGAGCAGCAAATGCTTTAGTGCT
GAAGCTTGGGAATTGGAAGAAGGGAGTTTCCCCCCTGTCTCTTGTGCATTGCCCCCACATTCTGTCTCTCTTGCA
AGAAAGGTGTGCATGAACAACTCTACTAAATCATCTTGTCTAATTTTCGTTTTATTTTATTTTTTTATTATTTTT
TTTTCTTGAAGGCCTTTTAATAGGTGACAATGATATTTGTGTTAAGTTGTGGAACCTAATTATTGTTGAGCCATA
ATTATTGCTGCTTCGCAGAGATGGTCGCGGATCAAATTAAGGGCTATTTACGAATTTATATTTTTGTAAAATTAA
ATGAAGGAATAGGAGTCTATATAAAATTTGATTTTAACCTAATTTATCTTATAAATAGATAGCAGACCTTAGACT
TTATTTATTATCATCAAATGGAGAGAGAGAGATTGGAGAAAATATGATTGATATTTTTTCTTGTAAACCTTTTTT
CATCATAGTGAATTATTGTAGTGACAGGACAGGACGTAGTCTCATACTGAGACAAACCTCTATAAATCTTGCGTG
CTTTGTTTGTTTATTTCTTTCTATAACTTGATTGTTCTTGATGGGATAGAATTAGGGTTTATAATTCAGAACAAT
TTGAAGTTGGGATTCTCGACTGATATATATGTTTCTTACCAGATGGGAGCTGAGTTTATAGGTACCCTCATACTT
ATCTTCACTGGAACAGCCACTGCCATTGTGAACCAAAAGACAAACGGATCGGAAACCCTTATTGGCCTGGCAGCC
TCCACCGGGCTGGCGGTGATGGTAGTCATACTCTCCACTGGTCACATTTCTGGAGCTCATCTCAATCCTGCTATC
ACCATTGCCTTTGCTGCTCTCAAGCATTTTCCATGGAAACATGTAAGCCCCTCTTTGACAGCCATTATCCAAAAA
TTATTTTTTTCTACGTACTCATTTTCAAAACATTTTTCTACTTTTAACCACACATTCTTCCAAAATTCACCATTC
AAAACTTTGTTTTTACCAAATAATAGCCATTAAATAGGGCCTAAATTTACATTCATGGCTGACTGATAGTATTAG
AAACTGTTAACAAAAAAGCTAAACTCTTGTCTGTTTCATGTCAACTATGGTGCTCTATCACATTGGATTGATTCA
ACTTTAATTTAATTTGGGTGCAGGTTCCTTCTTACATTGGAGCCCAAGTAATGGCATCATTAACTGCTTCATTTC
TGGTAAAAGGGATTTTCAATTCTATGATGGGTGGGGGAGTAACAGCTCCTTCAGGGGGATATGCTCAAGCTTTTG
CTTTGGAATTTATTATCACTTTCAACCTCATGTTTGTTCTTACAGCAGTAGCCACAGATACAAGAGCTGTAAGTC
TCCCTCTCTCTAGTTACTTTCGAGATATA**TAA**

>*Oeu*NIP6-related_ Oeu011481.1

ATGGATGGTGAGGATATTGCATCAGCTCCTTCAACACCTGTAACACCAGGGACTCCAGGTGCTCCTCTCTTTCGA
GATTTCAAAAGGGACAATAATTGCTTTAATGGTAGAAAATCATCTCTTCTTAAGAGCAGCAAATGCTTTAGTGCT
GAAGCTTGGGAATTGGAAGAAGGGAGTTTCCCCCCTGTCTCTTGTGCATTGCCCCCACATTCTGTCTCTCTTGCA
AGAAAGATGGGAGCTGAGTTTATAGGTACCCTCATACTTATCTTCACTGGAACAGCCACTGCCATTGTGAACCAA
AAGACAAACGGATCGGAAACCCTTATTGGCCTGGCAGCCTCCACCGGGCTGGCGGTGATGGTAGTCATACTCTCC
ACTGGTCACATTTCTGGAGCTCATCTCAATCCTGCTATCACCATTGCCTTTGCTGCTCTCAAGCATTTTCCATGG
AAACATGTTCCTTCTTACATTGGAGCCCAAGTAATGGCATCATTAACTGCTTCATTTCTGGTAAAAGGGATTTTC
AATTCTATGATGGGTGGGGGAGTAACAGCTCCTTCAGGGGGATATGCTCAAGCTTTTGCTTTGGAATTTATTATC
ACTTTCAACCTCATGTTTGTTCTTACAGCAGTAGCCACAGATACAAGAGCTGTAAGTCTCCCTCTCTCTAGTTAC
TTTCGAGATATA**TAA**

>*Oeu*NIP6-related_ Oeu011481.1

MDGEDIASAPSTPVTPGTPGAPLFRDFKRDNNCFNGRKSSLLKSSKCFSAEAWELEEGSFPPVSCALPPHSVSLA
RKMGAEFIGTLILIFTGTATAIVNQKTNGSETLIGLAASTGLAVMVVILSTGHISGAHLNPAITIAFAALKHFPW
KHVPSYIGAQVMASLTASFLVKGIFNSMMGGGVTAPSGGYAQAFALEFIITFNLMFVLTAVATDTRAVSLPLSSY
FRDI*

***Oeu*NIP7;1   *Oeu*037688.1**

Observation: 1841n removed at the 5' end
Intron/exon redesigned by homology with Oleur061Scf4622g04016.1

>*Oeu*NIP7;1_*Oeu*037688.1 | chr4:6837162..6838876 forward original version
ATGGCTTTCACGTACTTGTTTTCTTTATCCTGCATTCTGTTCCATTTCATATGCCCTTCCGTAGCCAAAAATTCT
TTCAGACCAAAGGCACTTTTCTTACCGGTAACCAAAGATCCATCAACCCTTCAATATGTTACTCAAATCGAGCAA
AGGACACCTCTAGTGCCAATTAAACTGACTATTCACCTTGGTGGACAGACCTTATGGGTGGATTGTGAAAATGGA
TACAACAGTTCATCGTATAGGTCTGCTCGGTGTCGCTCAGCCCAATGCTCGATTGCCAAGTCCCATGCCTGTGGA
GACTGCTTCACCCGTATGCCTAAAATCGGATGCAGCAATAATGCCTGCTATAACACCCCGGATAATCCTGCTATC
AACACTTTCTATGATGGTGGGGAGATTTCTGAGGATGTTTTGTCAATCCAATCCACCAACGGATTCAACCCTGGC
CAACCTGTTAACATTTCACGATTTATTTTCTCGTGTGTTCCCTCATTTCTGACAGCAGGGCTTGCTAACGGGTTA
AGGGTACAGCAGGGCTCGGGGGAAGCCTGATCGCCCTTCCTTCTCAACTTGCTCGTGTTTTCAGCTTTCAAAGAA
AATTTGCTATTTGCTTGAGCTCCTCCACTAGATCCAATGGCATCGTATTTTTTGGAAATGGCCCATATGTTTTGC
TTCCTGGTATTGATGTGTCTATGTCTCTTATTTATACACCACTTATCAAGAATCCATTTGGCATTGGATCCGTCC
CATTTCTGAACGAGCTCTCTTCGGAATACTTCATTGGATTAAAATTGATCAGAATCAACGGAAAATCAGTGCAAC
TAAATAGAACTTTGCTATCAATTGACAGGGAAGGTAATGGTGGAACCAAAATCAGCACCTTTAGACCTTACACTG
TAATGCAGACTTCGATTTACAATGCTTTGATTACTGCTTTCAGAAAAGAACTAGCCAATGTCACTAGAGTGCTTC
CGGTAGCACCATTTGGGACATGCTTCGACTCTAAGAACATTGGTAGCACGCTCGTGGGGCCTGCTGTTCCACCCA
TTGATCTTGTCTTTAAAGGCAACAATGTGTACTGGAGTATTTTTGGTGCAAACTCAATGGTGCAGGTTAATGAAG
ATGTAATGTGTCTCGGATTCGTGGATGGAGGGGAGAATCCCATGACATCAATAGTCATTGGTGGACATCAAATTG
AGGACAATCTTCTGCAGTTTGATCTAGCTAGATCTAGGCTCGGGTTTAGCTCTTCGCTCTTGTTCAGGCAAACAA
CCTGTGCCAACTTCAACTTTACATCTCTCGCTTAAGGGTTCTGTTTCATTTTTCTCATTCAGAATAACATCAAAC
TATAAAATAAATTCTTGAACTCTTTCTGTTTTTGTTCTGTTCTCATACAACTGCACGTTTTTTTCTTCGTGTTCC
TTTTTTTGCTTTTCATATCGCCTAAAAGTTGGCAAAGTAGTGGATATTAGTAACTAAAAGTATGTTCTTGATTTT
GCTTGGAAATGATATTAACAACTGCACCCCCTCCAACCTCCACCCCATCCCAAAACAAAAACATTGTAAGTGTTA
TATAATTGAGAAACAGTTGGACAGTAAAGGTCAACCTATTTTCCTTAAACATTAAAGAAGCATGAGTTACTTATA
ATCCCTTTGAAAGAACACAAGGATTGCGTTGAAACAAACAGTCCCCAACTTCTGCAACGTTATAAACTCCTCAAT
CGAAATGTGTATTCCTAACATAGAAATAAAAATGCAAAAGTGCGAAAGGATTCAACATTAAACTACAATTCATTA
GGCCTGTGGAACTCAAAATCCAATTCCACGAGCACGAAAAAATGCAGCAAAGTATGAAAGCACCATTTCGGGAAC
TGTCATCGACTCCTGACATTTTAGGTAGTACAGCCACAAGAAGCCAGCCCAAAAATGACCTAGAAAATGGAGATT
CAATGCTAAATGAAACTGTCTTCAACTCTCTTCATAAAATCGACAAAAGTCTTGTGCGTAAGGTACATGTCTACA
AAAGAATCTCCCATATTTGCTCGTAAAATCCATCTTTTCATCGTCTAAATCTGCAGGTATTGGCAGAGGCATTAG
GGACATTCGTGCTAATGTTCTGCATTTGCGCGATCATAGCAATAATGCATTTAATCGGATTGGAAGCAGGCCTAA
TGGAATATGCAGCCACAGCAGCACTAACAGTAACTGTTATAGTTTTCTCCATAGGAGCAATTTCTGGAGCCCATG
TCAATCCTGCTGTCACAATTGCCTTCGCAACTGTCGGCCCATTCCCATGGTCCGAGGTAGTTCCAAGTCCAAAAC
TTGTTAAATTTGAAGATACGGATCAAGAAATTGAATTTCAAATTTATTTCCCTTTATTCTTTGAAGGTTCCACTC
TATATATTGGCACAAATTGGGGGATCTACTTTGGCAACTTATATTGCAAGATTAGTATATGGCATTAGATCAGAG
CTTGTGATGACCAAACCACTCCAAGGATGCACTGCAGCTTTCTGGGTGGAGCTTATTGCAACTTTCATAATTCTT
TTCCTCACTGCATCCTTGTCGGATCGAGAAATTGAATTTCAAATTTATTTCCCTTTTTCTTTGAAGGTTCCACTC
TATATATTGGCACAAATTGGGGGATCTACTTTGGCAACTTATATTGCAAGATTAGTATATGGCATTAGATCAGAG
CTTGTGATGACCAAACCACTCCAAGGATGCACTGCAGCTTTCTGGGTGGAGCTTATTGCAACTTTCATAATTCTT
TTCCTCACTGCATCCTTGTCAAATGACCCTCAATCTGTAAGATTCTGTCAATTCTACTCGAGTCATTTTACGTTC
GACTACACTTATCCTGTGATATGTTCGGTTTAGAGTTATATCAGACATGTTGCACATATTAGGATATTCGATTTA
CAGTGTTCCTAGTAATATTTCTTCTGGTATGATATAGTGCCATTTTTGCAGATAGGACAGGTGTCTGGCTTTGTT
GTCGGGGTGGCCATTGGACTAGGAGTGCTAATTTCTGGGTAAAGCATAAAGATTCTACTCGAGTCATTTTACGTT
CGACTACACTTATCCTGTGACCGCAGATATGTTCGGTTTAGAGTTATATCAGACATGTTGCACATATTAGGATAT
TCGATTTACGGTGTTCCTAGTAATATTTCTTCTGGTATGATATAGTGCAATTTTTGCAGATAGGACAGGTGTCTG
GCTTTGTTGTCGGGGTGGCCATTGGACTAGGAGTGCTAATTTCTGGGTAAAGCATAAATAATTACTTATTACAAA
CTAACTAGAACTCACCAGATTCACTTAAGTCGATTAAATTATCAAATGCAGGCCTGTTTCAGGTGGGTCGATGAA
TCCAGCGAGATCGTTAGGACCTGCACTTGTTTCATGGAAATTTGATGATCACTTATGGATATATTTAGTAGCACC
AACTATAGGAGCCATTGCTGGTGTTTTTGTGTACCGGATTTTACGACTACAAGGTTGGTCTTGTGAGTCGAGTCA
AGCCCGAAAAGGAAGTGATTCTTTACATTAG

>*Oeu*NIP7;1_*Oeu*037688.1 | chr4:6837162..6838876 forward corrected version
ATGCAGCAAAGTATGAAAGCACCATTTCGGGAACTGTCATCGACTCCTGACATTTTAGGTAGTACAGCCACAAGA
AGCCAGCCCAAAAATGACCTAGAAAATGGAGATTCAATGCTAAATGAAACTGTCTTCAACTCTCTTCATAAAATC
GACAAAAGTCTTGTGCGTAAGGTACATGTCTACAAAAGAATCTCCCATATTTGCTCGTAAAATCCATCTTTTCAT
CGTCTAAATCTGCAGGTATTGGCAGAGGCATTAGGGACATTCGTGCTAATGTTCTGCATTTGCGCGATCATAGCA
ATAATGCATTTAATCGGATTGGAAGCAGGCCTAATGGAATATGCAGCCACAGCAGCACTAACAGTAACTGTTATA
GTTTTCTCCATAGGAGCAATTTCTGGAGCCCATGTCAATCCTGCTGTCACAATTGCCTTCGCAACTGTCGGCCCA
TTCCCATGGTCCGAGGTAGTTCCAAGTCCAAAACTTGTTAAATTTGAAGATACGGATCAAGAAATTGAATTTCAA
ATTTATTTCCCTTTATTCTTTGAAGGTTCCACTCTATATATTGGCACAAATTGGGGGATCTACTTTGGCAACTTA

TATTGCAAGATTAGTATATGGCATTAGATCAGAGCTTGTGATGACCAAACCACTCCAAGGATGCACTGCAGCTTT
CTGGGTGGAGCTTATTGCAACTTTCATAATTCTTTTCCTCACTGCATCCTTGTCGGATCGAGAAATTGAATTTCA
AATTTATTTCCCTTTTTCTTTGAAGGTTCCACTCTATATATTGGCACAAATTGGGGGATCTACTTTGGCAACTTA
TATTGCAAGATTAGTATATGGCATTAGATCAGAGCTTGTGATGACCAAACCACTCCAAGGATGCACTGCAGCTTT
CTGGGTGGAGCTTATTGCAACTTTCATAATTCTTTTCCTCACTGCATCCTTGTCAAATGACCCTCAATCTGTAAG
ATTCTGTCAATTCTACTCGAGTCATTTTACGTTCGACTACACTTATCCTGTGATATGTTCGGTTTAGAGTTATAT
CAGACATGTTGCACATATTAGGATATTCGATTTACAGTGTTCCTAGTAATATTTCTTCTGGTATGATATAGTGCC
ATTTTTGCAGATAGGACAGGTGTCTGGCTTTGTTGTCGGGGTGGCCATTGGACTAGGAGTGCTAATTTCTGGGTA
AAGCATAAAGATTCTACTCGAGTCATTTTACGTTCGACTACACTTATCCTGTGACCGCAGATATGTTCGGTTTAG
AGTTATATCAGACATGTTGCACATATTAGGATATTCGATTTACGGTGTTCCTAGTAATATTTCTTCTGGTATGAT
ATAGTGCAATTTTTGCAGATAGGACAGGTGTCTGGCTTTGTTGTCGGGGTGGCCATTGGACTAGGAGTGCTAATT
TCTGGGTAAAGCATAAATAATTACTTATTACAAACTAACTAGAACTCACCAGATTCACTTAAGTCGATTAAATTA
TCAAATGCAGGCCTGTTTCAGGTGGGTCGATGAATCCAGCGAGATCGTTAGGACCTGCACTTGTTTCATGGAAAT
TTGATGATCACTTATGGATATATTTAGTAGCACCAACTATAGGAGCCATTGCTGGTGTTTTTGTGTACCGGATTT
TACGACTACAAGGTTGGTCTTGTGAGTCGAGTCAAGCCCGAAAAGGAAGTGATTCTTTACAT**TAG**

>*Oeu*NIP7;1_*Oeu*037688.1

ATGCAGCAAAGTATGAAAGCACCATTTCGGGAACTGTCATCGACTCCTGACATTTTAGGTAGTACAGCCACAAGA
AGCCAGCCCAAAAATGACCTAGAAAATGGAGATTCAATGCTAAATGAAACTGTCTTCAACTCTCTTCATAAAATC
GACAAAAGTCTTGTGCGTAAGGTATTGGCAGAGGCATTAGGGACATTCGTGCTAATGTTCTGCATTTGCGCGATC
ATAGCAATAATGCATTTAATCGGATTGGAAGCAGGCCTAATGGAATATGCAGCCACAGCAGCACTAACAGTAACT
GTTATAGTTTTCTCCATAGGAGCAATTTCTGGAGCCCATGTCAATCCTGCTGTCACAATTGCCTTCGCAACTGTC
GGCCCATTCCCATGGTCCGAGGTTCCACTCTATATATTGGCACAAATTGGGGGATCTACTTTGGCAACTTATATT
GCAAGATTAGTATATGGCATTAGATCAGAGCTTGTGATGACCAAACCACTCCAAGGATGCACTGCAGCTTTCTGG
GTGGAGCTTATTGCAACTTTCATAATTCTTTTCCTCACTGCATCCTTGTCAAATGACCCTCAATCTATAGGACAG
GTGTCTGGCTTTGTTGTCGGGGTGGCCATTGGACTAGGAGTGCTAATTTCTGGGCCTGTTTCAGGTGGGTCGATG
AATCCAGCGAGATCGTTAGGACCTGCACTTGTTTCATGGAAATTTGATGATCACTTATGGATATATTTAGTAGCA
CCAACTATAGGAGCCATTGCTGGTGTTTTTGTGTACCGGATTTTACGACTACAAGGTTGGTCTTGTGAGTCGAGT
CAAGCCCGAAAAGGAAGTGATTCTTTACAT**TAG**

>*Oeu*NIP7;1_*Oeu*037688.1

MQQSMKAPFRELSSTPDILGSTATRSQPKNDLENGDSMLNETVFNSLHKIDKSLVRKVLAEALGTFVLMFCICAI
IAIMHLIGLEAGLMEYAATAALTVTVIVFSIGAISGAHVNPAVTIAFATVGPFPWSEVPLYILAQIGGSTLATYI
ARLVYGIRSELVMTKPLQGCTAAFWVELIATFIILFLTASLSNDPQSIGQVSGFVVGVAIGLGVLISGPVSGGSM
NPARSLGPALVSWKFDDHLWIYLVAPTIGAIAGVFVYRILRLQGWSCESSQARKGSDSLH*

*Oeu*NIP-related, considered as Pseudogenes

*Oeu*NIP4.2

>*Oeu*NIP4;2-related_OeuChr8 17852182..17858508 chromosome 8

ATGGAGGTTATTCCTTATTATTGCTAAATTTATGCAATCTGCATAGTAAATATAGCCTTATTTTGGATACATTAA
CAAAGAAATGAGTGTAATAACTTGATATGATGTTTTAAAACTAGTATTTTATACAAAATTAGGATAGGAAAAATG
GTGTTACATTAGAGTCGCCATGCTAATGTGGATTGCATAAAAAATTGATGCATAAGCAAAAGTATATAATGTCTT
CAAAAACTCATCTCCAGTGCAACCTTAGTAGGGCTTCGTCTAGCTGTCGTGACAAGAAAAAATAGACATGGTGAA
GATGTTCATCAGTTATAAATGCTCCGTTAGATGGAGTAATTCAGCAAATTAAAACTCTCTCCTTTTCTCTCAGAT
TTGTTATTTTTTTCTCTTCCATTAAATATATTTGAGTAATAGAAAGAATTTATTCTAAATGAGATTTGAACTTCA
GCATCTATTCATTAGACATAATGAAACGTGTCTGGGAAGCCTTTTTATTTGTCTACCATTTGGATTGCAATCCAT
AGAATGTTTTAGAGATGCTCTTAGTTGAGCTCCACTGGTATAGGTGCTTCAGCAGATAATTGTGTCTATACTTTG
TTATTTTAATAATATTCTTGAAATAATCTTGAGTTGTTTGTATAATCTTGGCACTAACAATCCCCATTCCAATAT
TTCAAGATTAAGATTTCACAAATATTAAATAAATTTAAAGATTAGGATACCATGGAATATGTGACCGTATTGTGA
AGAATCGATTAGTGATGATGTATATAGCTGATCATTAAATGGAAGGGTTGAATTAAGGGCTGTGATTTCTTCTAA
TCATAGTGTGACATATTAATTCAAATTTTGATATTACATAATTAACAACCATAAAAGGCAAATTCTCTTGATTTA
CTATCTAAAGAATATAAGTGTATCCAAATGGGCCAATTACGATGTCTAATAAAGGAGGAGAGTGAAAGTTTAATT
ATATCCATCGACATGTCTCTAAATTAGCTTAACCATTAATCTTCTGATTATCTCAAACCATGCATATACTTTAGG
ACATTTTAAGCTCACCTAAACTCTTTTTCCTTTTAATGCAGACAGAGAAGTAATTCTTTAATTTTTGCTAATTAT
TCATCAACAATATCAGAATGTCTGCAAGAGATATTGCAGAGATTGAAGAAGGTGTACCTGATCCATCGGATAATC
GAACTGTAAATGATGGTACAACTTTTTGCACCTCTGCTAATGTTGTTGTGGTCACTCAAAAGGTATTCAAATCCT
CCCCCTCATGAAAGAAAGAAAGAAAAATTTAGGGTGTGATTGATGATCATGAAAATTTTCATTTTCTAATAACTT
CTACACTCTTTTCTCTTCACTTTCGTGATATTTAGATTGTCGCAGAGGCCATTGGTACATATTTCTTGATATTCG
TAGGCTGCGGTGCTGTTGCTGTTAATAAAATTTAAGGTACAATAACATTTCCAGGAGTATGTGTGGCATGGGGTC
TGATATTTATGGTCATGGTTTACTCCGTTGGCCACATCTCTGGCGCGCATTTTAATCCAGCAGTCACCATTGCTT
TTGCCATCTTTCGCCAATTTTCTTACAAACAGGCATGCCCTTAGTTTTCTTCTTCTTCATTAAAAAACGTGAATG

ATTCAATTCACTCATCGGTGACAGCTTCTATGAGCATTTTTTTTTTTTTTAAAAACAATTATGGGGTGCTAAGG
ACGGTGGACTGGACAGGATCAGATAGGACAACCGCGCATTTGTTTCCTTAATCTTGTCCTGTCCAAGTACGAAA
CACGTGAACAATTTATTTTTCTTTCTTTTGTAATATGTGTGGATTTGTCATGATTTATTAGTAATGTTACTGCAG
GTACCACTTTACATTTTGGCCCAGTTGTTAGGATCAATTCTTGCTAGTGGTACATTATAACTTCTGTTCGATGTG
AAAAGTGAAGATTTCTTTGGAACCACACCAGTGGGATCCAATATTCAGTCCCTTGTGACAGAATTTATTACCTCC
TTCCTCTTGATGTTTGTCATTTCTGGGGTTGCCACAGATAACAGATCGGTAAGCAAATTTTCTGTAAACACCCTA
ATGTATGCATTGCTTATTCTTTAGCGGATTATGAAGTTATTACACATTCACACAACTATGACTTGCACGTAGACG
TGTAAAGAAGGACGCGCCATGCAAAATATCTACTTTTTGATGTTCTGTGCTCTTGGGTTGCTTGGGCCGGCACAG
TAAAAATTCCATCACCCAAAATAATCAACGATCCCATTGTTGGGTAAATGGTCAACAAAAGCACGTGCATGCAAA
TATGAATAGCATATGTGCATAAAAAGTTAAGCAAAATAATGACTTACTGACGTATAGAAATAATAAGGAGATGAA
ATGAAGTAAGATACAGAAATGCAAATTTCCTTAAAATAGATTTTGTCCTTGCACTGGTGCTAATGGACATATTTC
TCTCAAGATACAACATCTCTTCCAAAATTAATATAGTACAATATTAATTTTGTGTGGTGAACCACTGTTAGATGT
TACTCTTCTAGAATGAAATATATAAAACTAAATATCTAACACGAAACTCAAAAGAAATTTGGAAATTATTTTGGC
ATAATTTAGAGAAGGAATGAACTAGAAATCAATTATATTGAATTGATCTGGAGTGAAGAAAATGGAATTAGAAAT
CAACACATATTGAATTGGCTCAAGAGGTCGGCGACAGTTTGTTAAAGAAATTTGATGCTATCCTTGTGTATTGCA
GTCTGGCTCAACTCACTTTATGCTTCTATCAATAAAATATTTGCACATTACAATATGAACATGCATGAAACAATT
TATACCCCTTCTGAAGATACTCTTTTTGAATTACCAATTATGTAACAAGTTTTAGCAATTTGTAATTATCCTCTT
TTATATTAGAAATGACCCTAAAGGTTTTTACTTGTGCAGATTGGAGAACTGGCTGGAATTGCCATAGGAATGACA
ATACTCATAGATGTTCTTATTGCAGGGTAATTACAATTTCACCTTTCACTTTATAAATCTTCATTCTTTCGTGCT
TATAAATGATGTTGGTAGTGTCAAAAAAATCCCTTAATACTTGGTTATTTTTGGCTAGGATTGGAAGTAGAAGTT
AATTTCAAATATTTTTTTTAACTGTTTTGAATAAGCCCCTTTTTCTTATTTTTACGTAAAAATTTTTTTTACTTA
GTCTGAAAATAATTCTTACTTTAGTGTCAAAATAATTAGTTTACTTGCTTTTAAGTTTGACTCATAACTAATTAG
CCTTTATATGTTAACATAAAAATTAGAGATAATTAAATGACTAAAATTTTAGCTCACGGCTGGTTATTAATCTTC
GTAGAAATATTAATTTGGTGTAGTGAAAAATATTATTAATCCTCGAATAACGGATTCTTATGTTGGACCCGGAAG
GATTGTAGTAAATGAGATTAATGACGCGATATGAAGTAATGAAGATCACACTGAGAAACAAGATTTAAAAGTAAA
GAAATTAGTAAAGAGAGTTAATCATAGAGGCACGACGTTTTATTAATCGACAATATGGAGAGAAATACAAGAACT
CAGCAATTCTTGCTTAAACAAAACTGAAAGAGAAGACCACTATTTATAGACACATGGCATAGCATAAAATGGAAA
TAATGTGAAATAAGAAAATAATATATTTCCATAAAAATCATGTGAAATATAAAGAAACAAATTTTCCTGTATGAA
AATAAGGAAACAATTTATTTCAAAAAAAAAAAAATCCATCCTAATTTGGGTTACAAACCCAACAATCTCTACCTTG
ACTTAAATTCTGGTGCAACAAACGAAACTCGAAAATTTCAAATCCGCCTGCATCAAAATAGTCAATCTCCTTAAT
ACAGTAACTAAAACTTTCCAAACCAAATCTTCGTCATCTAAAAGGCGTCCATAAACGACATTTGAACCCAAATCC
TACGTTCCCAGAATTCCATCGAGGAATTATTGTTGACAAAATAAAACAGGATCGAAGCACACACTGTAGCAGAAA
TCATACCACATGCTGGATCAGTAGGCCCATTGCTAGAACCGTCACCTTCAACGTTCCCAAAAGCATCCCAGTTAT
TATTAACACTATTGTACCTACGCCTCATGAGTTCAACCCAAGAAAATGAATACAGTGTATCCGTTGAAATTGGTG
GCAAAGTGAAATCGTCAATGCTCCCCTGAGCAATATTCTGAATTGATCCAAAGATCGTCACAAGAGGTACTATAC
CAAATGGAATATCCACCACGGTATTGAACTTAGCCCCATGTCTCTTAAGTGATTCTGCATTTCCCTGCAAGATGT
ACAACTGAGATTTCTTCGTCCCTCCAATACAATCCTGATACTCATTCGGACATTCAAAACTCCACCTTCACCGAA
AAATTTATACTCTTTTGCATCCAGAGTACTAAGTGAGATGGGATTCCTTTTCAGGTCTGGAATATGTTGAACCCG
ATCAATTGTTTTAACTGTCCTATCCCGTAGTCTAAATCTGACTCGATCGATCCCAACAATACGGTAAGACTGGTT
GTTTCCCATTACAACTCCACCTTGTTGAATGGCCTCATATGTTTGAAAAAAAAATCGATTTTGGAACACATATGA
TGCGAACATGCCGAATCGAGAATTCACTCCTCGTCAGGCCTAGATTTAACGTCAGATACCACGGTCAACATCACA
TCTCCACTGTCTGAAAATCTTCAACTACATTTGTTTCATCTGAAGTCAAAGGTTGCGCTCCACGATTTGTAGGTA
AATCACATTTAGCTTGCAACTTATCACAATCCTTCCGAATATGTTCATTACACCTGCAGTAGAAGCAAAACAAAT
TGTTATTATTCTTCGATCTTTCCCTCCATCGACCACCATGAGATATAAGAGCTTTCTCATCCCTAAATGTAGCTC
CAACAAAATGCTTCAATTTCTCCATTGAGTGCAAAGCATCATAGACGTCTTCTATAGTCAAAGTATCCTGACTAT
ACAAAATAGTGTCTCGAAACGAAGCATAAGAGCTAGCAGCGAATATAACAGGATCAAGGTAAGATCCTTTTCATC
GTACTTAACCTCTAACGTTTCCAAATCTATGACAATTTCCTTGGACACGGCAATATGGTCTTCTAACGACCCTTC
TTCCACCATGGGGTGGGAATATAGTTTCTACTTTAGAGCTAGTTTACCGCTAAGACTCTTGGTCATGCATAATTG
CTCCAATTTCAACCACAAAATAGCTGTAATTTCACTTTTAAGACATCTTGTAGAGTATTTTTGAATAGATGAAGA
TGAATCAATGACAAAACCTTCTGATCCCTCCGTTGTCCAATTAGTTAACATCTTCTCAAAACCCTGTAACACATC
ATCCAAATCCAACTGCGCGAAAATAGCCTCATCTTTACTTGCTACAAAGAAAACCTTGTATTGTGATCTAGCAGC
GAAATGTCATACTTTGTGGTAGTCATCTCTAAAAAAACCTCAACCAATCCTCAAAAGAAAAATCAAGCTCTGGTA
CCACTTGTTGGACCCGGAAGGATTGTAGTAAATGAGATTAATAACGCGATATGAAGTAATGAAGATCGCAACGAG
AAACAAGATTTAAAAGTAAAGAAATTAACAAAGAGTTAATCATACGACATTTTATTAATCAACAATATGAAGAGA
AATACAAAAATTCAGTAATTCTTGCTTAAGCAAAACTGAAAGAGAAGACTATTATTTATAGGTACATAGCATAGT
ATAAAGATGAAAATAATATATTTCCATAAGAACCACGTGAAATATAAGAAAACAAATTTTCCATCATGAAAATAA
GGAAACAATTTATTTCCAAAAAAAATCTACTCTAATTTGAGTCACAAACCCAACATCTTATCTTATATGTATACAA
GTGTGAAAAATAAACCTCAATAACATCCAAGTCTAACTGTTGAATGTGTACCTTCTCAAAACTAATACTTTTTAG
AGTATCCTCACAAAATGAAATAATATTTATATTATTACTTCTTTTTCTTTTTCGTGGGAATTAATTTTCTCCTTT
CATTTTTCTTTTATGCTAACAACATAACAATGATTTACAGGCCAATTTCAGGAGGTTCCATGAATCCTGCAAGGA
GTATCGGGCCAGCACTTATAATGCACCAATTCAAGTCACTTTGAGTGTACGTAGTCGGTCCACTAGCGGGCACCA
TAGCAGGCGGTTTCACATACAATTTGATTCACAGAAAAACCACTTCGAGAAATTACAAAAACGAGTACTTTCCTC
AAAAGTATGTCAAGAAGCCACGCT**TAA**

>*Oeu*NIP4;2-related_OeuChr8
ATGGAGAATGTCTGCAAGAGATATTGCAGAGATTGAAGAAGGTGTACCTGATCCATCGGATAATCGAACTGTAAA
TGATGGTACAACTTTTTGCACCTCTGCTAATGTTGTTGTGATTGTCGCAGAGGCCATTGGTACATATTTCTTGAT
ATTCGTAGGCTGCGGTGCTGTTGCTGTTAATAAAATTTAAGGTACAATAACATTTCCAGGAGTATGTGTGGCATG
GGGTCTGATATTTATGGTCATGGTTTACTCCGTTGGCCACATCTCTGGCGCGCATTTTAATCCAGCAGTCACCAT
TGCTTTTGCCATCTTTCGCCAATTTTCTTACAAACAGGCATGCCCTTACTTCTATGAGCATTTTTTTTTTTTTTT
AAAAACAATTATGGGGTGCTAAGGACGGTGGACTGGACAGGATCAGATAGGACAACCGCGCATTTGTTTTCCTTA
ATCTTGTCCTGTCCAAGTACGAAACACGTACCACTTTACATTTTGGCCCAGTTGTTAGGATCAATTCTTGCTAGT
GGTACATTATAACTTCTGTTCGATGTGAAAATGGGATCCAATATTCAGTCCCTTGTGACAGAATTTATTACCTCC
TTCCTCTTGATGTTTGTCATTTCTGGGGTTGCCACAGATAACAGATCGATTGGAGAACTGGCTGGAATTGCCATA
GGAATGACAATACTCATAGATGTTCTTATTGCAGGGCCAATTTCAGGAGGTTCCATGAATCCTGCAAGGAGTATC
GGGCCAGCACTTATAATGCACCAATTCAAGTCACTTTGAGTGTACGTAGTCGGTCCACTAGCGGGCACCATAGCA
GGCGGTTTCACATACAATTTGATTCACAGAAAAACCACTTCGAGAAATTACAAAAACGAGTACTTTCCTCAAAAG
TATGTCAAGAAGCCACGCT**TAA**
>*Oeu*NIP4;2-related_OeuChr8
MGSNIQSLVTEFITSFLLMFVISGVATDNRSIGELAGIAIGMTILIDVLIAGPISGGSMNPARSIGPALIMHQFK
SL

*Oeu*005264.1 *Oeu*NIP7-related

>*Oeu*005264.1 | Scaffold11917:939..1312 reverse
Incomplete sequence

>*Oeu*005264.1
ATGCTAAACAACACTTTTATCAACTGGCTTCAGAACATCGATAAAAGTCTTGTCCATAAGGTACTTGTGTACAAA
ACAAACTCCCATATTTGCTCGTAAACTCCCATCTTTTAAACGTCTAAATCTGCAGGTATTGGCAGATGTTGGGGA
CATTCATGCTAATGTTCTGCATTTGCGCTATCATAGCAATAGTGCAGTTAATAGTAGTGGAAGTAGGCCTAATGG
AATATGCAGCCACAGCAACCTTGACAGTAACTGTCATAGTTTTCTCTATAGGAGCCATTTCTGGAGCCCATGTCA
ATCCTGCAGTCACAATTGCTTTTGCAACTGTTGGTCCATTTCCATGGTCCAAGATATTTATAATTACTTGC**TAA**
>*Oeu*005264.1
ATGCTAAACAACACTTTTATCAACTGGCTTCAGAACATCGATAAAAGTCTTGTCCATAAGGTACTTGTGTATTGG
CAGATGTTGGGGACATTCATGCTAATGTTCTGCATTTGCGCTATCATAGCAATAGTGCAGTTAATAGTAGTGGAA
GTAGGCCTAATGGAATATGCAGCCACAGCAACCTTGACAGTAACTGTCATAGTTTTCTCTATAGGAGCCATTTCT
GGAGCCCATGTCAATCCTGCAGTCACAATTGCTTTTGCAACTGTTGGTCCATTTCCATGGTCCAAGATATTTATA
ATTACTTGC**TAA**
>*Oeu*005264.1
MLNNTFINWLQNIDKSLVHKVLVYWQMLGTFMLMFCICAIIAIVQLIVVEVGLMEYAATATLTVTVIVFSIGAIS
GAHVNPAVTIAFATVGPFPWSKIFIITC*

*Oeu*053221.1 *Oeu*NIP-related

>*Oeu*053221.1 | Scaffold635:402535..403011 forward
Incomplete sequence
3' end stopped by an other gene

>*Oeu*053221.1
ATGGCAAATTCACCTTCTATTGTTAGCTTTTCCTCTAAACAAAATTTACCAACAGACTTGTCCATTACAGAGGAA
GCAAAGGGTTACTCCGCCGACGAATTCCCTCCAAGAGATTCTGTTTCCCCGTCGTACTACCAGAAAGTAAGTTTT
TAAACGATTGCATTTGACAATACAGTATACAAGATTTTAATGGATAATTTGTGCTTGAGTTAACAGATTATAGCA
GAGATTTTGGGCACTTATGTTCTTGTTTTTGCGGGTTGCGGGGCTGCTCTAGTTGATCGAGAGAAGACATTAACA
GTTGTGGGTTTAGCAATGGTGTGGGGGCTGGCTTTGATGGCAATGATATATGCACTTGGCCATGTTTCTGGTGGG
CATTTCAACCCTGCAGTTACCATTGGCTTTGCAGCAGCGCGAAAAATTCCACTCGTTCACGTAAGAATATCATTT
GTACACGAAATTTGGATTGTTCAG**TAG**
>*Oeu*053221.1
ATGGCAAATTCACCTTCTATTGTTAGCTTTTCCTCTAAACAAAATTTACCAACAGACTTGTCCATTACAGAGGAA
GCAAAGGGTTACTCCGCCGACGAATTCCCTCCAAGAGATTCTGTTTCCCCGTCGTACTACCAGAAAATTATAGCA
GAGATTTTGGGCACTTATGTTCTTGTTTTTGCGGGTTGCGGGGCTGCTCTAGTTGATCGAGAGAAGACATTAACA
GTTGTGGGTTTAGCAATGGTGTGGGGGCTGGCTTTGATGGCAATGATATATGCACTTGGCCATGTTTCTGGTGGG
CATTTCAACCCTGCAGTTACCATTGGCTTTGCAGCAGCGCGAAAAATTCCACTCGTTCACGTAAGAATATCATTT
GTACACGAAATTTGGATTGTTCAG**TAG**
>*Oeu*053221.1

MANSPSIVSFSSKQNLPTDLSITEEAKGYSADEFPPRDSVSPSYYQKIIAEILGTYVLVFAGCGAALVDREKTLT
VVGLAMVWGLALMAMIYALGHVSGGHFNPAVTIGFAAARKIPLVHVRISFVHEIWIVQ*

*Oeu*017634.1

>*Oeu*017634.1 | Scaffold1737:102936..103291 forward
Incomplete sequence

>*Oeu*017634.1
ATGGCGGATTTCGAACCGGGGACACCAGGGACGCCGACAACGCCGACGCCGCTTATGTCGTCTCTGAGAGTGGAT
TCACTGTCGTACGATCGGAAGTCAATGCCGAGATGCAAGTGCTTGCCTTTGGATTCGTCTACTTGGGCGGCACCT
CAAACTTGCTTCACCCATTTCTCTGCACCTGAAATTTCTCTCACTCGCAAGGTTTCGCAATCTCTCTCTCTCTCT
AACACACGCGCGCGCACTGAGTTACTGAGATCCTTACACGCACGCACTATTTTCCTGGAGATCCTTGCACGCACG
AATTCCTGGAAAATATACTCGTTATTATGTATGGAAAACAGGAAAAGAAAAGA**TGA**
>*Oeu*017634.1
ATGGCGGATTTCGAACCGGGGACACCAGGGACGCCGACAACGCCGACGCCGCTTATGTCGTCTCTGAGAGTGGAT
TCACTGTCGTACGATCGGAAGTCAATGCCGAGATGCAAGTGCTTGCCTTTGGATTCGTCTACTTGGGCGGCACCT
CAAACTTGCTTCACCCATTTCTCTGCACCTGAAATTTCTCTCACTCGCAAGGAAAAGAAAAGA**TGA**
>*Oeu*017634.1
MADFEPGTPGTPTTPTPLMSSLRVDSLSYDRKSMPRCKCLPLDSSTWAAPQTCFTHFSAPEISLTRKEKKR*

SIP – Small Basic Intrinsic Protein

*Oeu*SIP1;1 *Oeu*050292;1

>*Oeu*SIP1;1_*Oeu*050292.1 | chr4:9340524..9350151 reverse
ATGGGTGCTATAAAGGCAGCGGTAGCCGATGGGGTGTTGACTTTTATGTGGGTTTTTTGCGCATCGACTTTGGGC
GCACTCACTTTTGTGGTGGCTTCTGCTCTCGGAGTATCCCAAGGACTACCCACTCTTCTCGTCACTACTGTCCTT
GTTTTTGTACTGCTGTTCGTGTTCGGCATCATCGGCGATTTCTTGGGCGGCGCCACCTTCAATCCCACTGCAATC
GCTGCTTTCTATGCCGCTGGTATTGGAGGCGCCGATTCTCTCTTCTCTGCTGCCCTCCGCTTTCCTGCCCAGGTA
TGAATCATGTGATTAGTCAGATATTGATCATGTTTCTTGAGAGTATGTAATCCTACTTGCTTGATTCAAATGTAG
ATTAGTATGTGAATATTGTCAGTCGATGATATGTTTGTGAAAATCTAGATTGCTTAGGGCTAGATCTATATAATG
GAATTTAAGATTTTAAAAAAAAGGAGGTCTCCCTTGACAGATGATATAGTTGAGAAAACTAACAGATTTGAACTG
AACGACCAAATTTAAATAAAAGGCACACATGAATAATGAGTTTGCAAATAATAAACTTTGGTGACTTACACGAAA
ATTTTACCACAATTTAAGTGTATACTATGCAGGGAATGAAAATGCTAAAGTTATTACTTTTATCCTTGACAGGCG
GCTGGTGCAGTAGGTGGTGCTCTTGCAATTATGGAGGTTATGCCAATGCAGTACAAACACATGCTTGGGGGTCCT
TCATTGAAGGTTGACCTGCACACTGGAGCTATTGCTGAGGGCGTTTTGACTTTCACAATTACCTTTGCTGTCCTT
CTCATAGTCCTTAGGGGTCCAAGCAACCCACTAGTGAAGAATTGGTTGCTTGCCATGGCAACTGTTTCGGCAGTC
GTTGCCGGTTCAAGTTATACTGGACCTTCGATGAATCCTGCCAATGTAAGTGACATATCTCTTTTCCATTCTATC
GCTAGATTCTGAAATTATATGATGATCTATGTCTTATTGTGGAATACCCTAATCCTTAAAAATTGCTGAGTAATC
AAAAAATTAAAGAAACAAAATAACATGAGGTGAGAGTTTCTCTTTCCCATAGCACATAAGGACAACGTATCTGCT
GAACCTTTCTTCTAATTTTTTTTTCACATCCCAAGCCAACCTGGAATAGAGTAGTTACTTTTCTCAATATGGTA
CCCGACCGTGTTTCTTTAAAATAAAAAAATGTTTATATGTAATGTGATTGAGCTTAGACATCTGAAGCACAATTT
AGAGTACTGCTCGCATTTGGCTCCCATATTTCCATTTAAGAGGTCCACGAGTCGCATCATTATTGTCCTGTTGTG
GGATGTCAAATTATCCTCAACTACCAAGTTTCTCTTTCCGTCTATGTGCCACCTTGTGATTGATCGATTAAGTGC
TGTTACCCTAATTAGGACCCACAGCAAAACAGTAGCCCTTTCCTTTACATTGACCTTAAGTGGGTTTGGAAGTTC
CATCTATCGTAAACTTCAGATCACTTTCCACATGATTGGATTTTTCTCCAACCTTGGAATAACCAATCATTTTCG
TATTGAGTCCTGAAAACTCCAGCTTGATCATTAACTTTCAGATTCAAAGAATATAGTTTTAGCTTTTGCTCATTT
TGACCATTATGGTCTGGTAGTGGCATTGATTTCCAGACTCAGCCATTACTTGGGAGCATTTGTTGAAATAACATC
TGCTGTAGGAATAAGGACAATGTCTTGGGAAAGTCAATTTTGGAGAGATGAAAAGCTTGCTGGTACATCAGTTAT
ATAACCGGTTGTAGAAAGTTGCATTGGAATTGAATAGCGCATGCTGAGCACGCAATAGAATGCATGCTTCAATTA
ATTAGTAGAGAAATGATAAAATTACATATGAAGAAGAATGATGAAGACTTTAAAATTTTCAGCTCAAGCTTCTAA
TACTTAGGTTTCTTCCAACCGAAGCTACTATTTATTCACTTTGGTGCGTGGGACTTGGGCACCTATATCCAACTC
AAGTTCAATGTGTATCTTTTTCAAGTTTCACGCTATTAAACTAATGAAGTTTTCTGGGGAAAGTGTGTTAAAATT
CTGTCTGGAAGTAAACTACTGCAGAGTTTTCTTGAAAAAGTAATCTGTCTTTCTCAACTATTTTTGTGGCCATAA
ATTAGGTTCATTAATGGATTAATTATGTCATGACCATTTCGCTAATAATCTACTACACAATTGCTGAACAATTGA
AATATGCCCGACGCAATGAAGGAATTGTGAGAATAAAAAGTTTGAGACTTGAGATGAACCAAAAAGGAAGTTTCT
CACTTCACCACAGTGCTAGCCTGAATAAGGATGGAGATTAGAGAATAATCACAAGCAAATTAAAGGGCTTAGGAT
TAAATTAAATCCAAGCTATCCAATACTTAGTCATATTAGTCAGACAAAATGTCTGATTTTTATACATGCATTTGG
AATAAAATATGAATTTTTAGTTTCATTTTTTTTTGAGTTCCTATTTTTTTTCCCACATGTCAAAACTTTATTAT
ATTGAAAAACATAAATAGGTATCTAAAACTAATAAGTAATAATTTATAATGAGTTAGTATTTAATCCAATTAGGA
AACCTTCTATTTTTTTGTATGTGCTTAGTTAAAAATAAAATATCCAAATATAAATACCCTCTAAAACTCAATTAA
ATCATCACTTGGCAATTCTGAGTAATTAACCACCAATGGTTTGGCCAATGACCTTAGGACGAACAAAAGTATCTA
AGCTCTAATGAGATCCCCTGAATACTGGGCAAATTATGTTGAAGTAACATTATTGTATCTTGTCCATAACCTACC
AATAACTTTGAATGCCCTTATGGAATGAATATCCTCGACCCATAGAAATGGAAAATTATCAACCTTTTAGGTTAC
ATTTGACATTCCAATCTTCCTTGATTCACTAATTCGAACCATGTGTGTAACGCTTCGAGATCTTTTTCATCTTC
ATTATTTTATTCTTTTATAAAAATAGGCTCAAGGTGTTACTAACATCTTCTTTGTCTAATTTAAAAAGAAATATC
AGTTTCATTAAGTGTTGAAAATGCATAAAAATAACTATTAAATAATGTGCACAAAATTCAATATGAAGATGTTCT
TTAAATAAAAGTTTGACAATTATCTTTTTCAGAAAATTTACTTGGCGATCCCATAAGCTACACTCGGTCAATCTT
ATATGCCTCCAAACGACCCGTTCTCATCTGAAAGGTATTGTAAAAGTGTATTTTGAAAACATTCAGTAAGAATAC
GAGCACATTTTTATTAACACGATATAAATATAAAACTGACATAATATATCGACATTTCTTAGATTTAGAGTTCAA
GGAATTTATTAAGAATTCCCATATCCCCTAAGATTTAACATTTTTGACTAAACAGTCCATCCCGTAGGATTTAAC
ATTTTTGACCTAACGGTCCATCTAGAAGGATTTAACATAGAGTTCGACTGAAGGCCTCATAGCATTTTATGCCAA
AAATTATACAAACATCAAGGAATAAGAATTTACCATTTTGCACGATGTATTCTTACCTCAATGATACCAAAACAA
AATATGAAAATATGGTTTGTAGAATAGAAAAATAAATTATTTCTAATAGATTGTGTGTATGTGAATTTATAGGGA
GTGCACCCTTTTTTTCTGGGAATTATAATCTAGTTAGATCACCTATATATATATATATATATATATATATATATA
TATATATATATATATATATATATATATATATATATATATATTAGATTTAATAGGCTCGTGATATTCTTTTCAATTTC
CAACGTACCAATATGAATTAAATGAAAGTTTTGATTTATTATTTTATTACTATTTATATTTGCTTATAAACAAA
ATATAAAATCTAGAATGCTTTCTTTATAACAAAATATCTCGGGTATAACTAGTTTAAAAATTACTTTTTCTTGAT
TCCAAATTATATTATTTAATACAAGCGATAAAACGAGTATAATATTTATATTTTATCAACTTCTAAAATACGTAA
ATAAATATTCGACCCAAATAAGATTTACGATAAATTCTAAGTACACATGAGCAAAATAAATCATCAATTTTTGGA
CTTTTAGTGTAACGACAAATCTAAACATGAATTGTTTCATTTGCTAAGTGACCCATAAAAAGTTCGTGGTGTTAC
AATGTGACCACCAATATTGATGCAAGATAAAATAAGGAGAAGGGGAAAGGGAAAGGGAAAGGGAAAGGGAAAGGG

AGAGAAGAGGAGAAAGGAGGCGGAAAGATAAGAAGGGGGGAGAAAATAGAGAGAAATAGGAGAGAACATCAAGTC
ATTTCATAATCAAAGATTATTTCCAATTACATTGCATAGACCCTTTATATAGGGTTTGAACTAATTATATTTGAA
GTAAGAAAATACTCAAAATAAATAACTAATTATGTTTCTTAAAATAAACTTTAGAATGAAAACTAAATATACTAA
AATCCATCCTAATTCTCCATCATTTCTCCTAAGCTGAGATAACTCGACTCCAGCAAAATAAATTGCAACTTCTTA
CCCAAGGAGGGCAATGTTTCTTCTTCACCAAATAATGGACCCATTCACATATCAATAATTGAACCTGAAAAACAT
TCACAAACTTCCAATTTGACCAAAGGGCTCATAACCAAATAGCAATAAGGAACTTTAGGAAGCCACAACTTGTGC
AACTCATTTACAAAAACTGTTACAAGATCCACTTGAAGGGCACAAGGTACTAGCACAATCGACTAGCTTGCAGAA
ATGAATAAATGCAACAATGGTGGCTCGAATGTACCCTGACAAGGCAACAATTCTGTTATATTGAAAATAAAACCC
TCATTTAAGTGTGATGGCCAATCTAATAGATTAAATTCAAGTGAGAAATTTAATCGTTTCTCTACATGTGTAAGA
AAAGTGTTTGAACGAACAATGACATTAGATAATGTGGTAACCCGAGAAGCATCACAAAGTAAAGGTAAAACAATA
TCAAAGTCAACGATAGAGTCATACAACAGATTAGGCAAGATCAATTTTGGCACGTAGTCGTGGATAGGTGGATTG
TTTGACTCAGGTGACAATAATATAGATAATTCCCCATCCTCGATATTAGAATATTCAATTGACTCAACGATAGAA
ACCTCAATTTCTTCTTCAAGAGCAATACAACTAGACTCAAGAGATGATTGTTGGAAATGGGTGGTAGACCTAGCT
TATGACACGCTTCGCGAAGATGGAAGGATAAGTTCCATAGGTTGTCCTGTTGGTAAGCTTTGTCTAAGGATTGGA
ATGTAGGAATATCATTAGGCAAAGTTTCAGGATTCAAACCATTGATAAACCTAGATGTTGTGATGTAGAGAGCCT
TATAAATATAATAATTGTTCAATAACTCTTCGAAGTGGGCAAAATATGCACTAGCAGTGGACGTACCTTGCCTAA
AGTGCAAAAAGTATTGATTTAACAATTGAGTTCGATAATTTACATATGCATATTTATTACTTAGGACTTGTTTCA
TCTCATCCCACAAGGTGACACTGCGTTGGCCTAGACGATTATGCAAATATGATCTCCAATAAATTTTAGTAGAAT
CGACCGATGATATATACTAGCATGACAAATTTTACATGCATCACTCAAATTGTGATAGAAAAAGAATTTTTCAAG
ATTATCAAGCCAATCAAGAACTGAATCAGATCCAGTGAGACATTAAAGTCCTATAGGTGGATTGCAACTTGCTGA
GGAGAAGAATGTACCTTTTTTGACAAAGGTATTAGACGGGGAAAATTGCGCGGATAAGTGCAAAGTGAAGGTATA
TGGTTGTGGTAGTGACCTAAGGATGTGTTGTGGGCGTAACTGTTGTTACATGAAGTGTATGTAGTGTACGATGAC
ATTTTTTTTTTTTTTTTTTTTTTTTTTTTTTATTATTATAAAAATGAGGTTTGATGGGCTACGAACACCGAAATG
AAAAGCAAAGCGTTCAAATAACAAGATGATATACCTTACAAAAGTCCAATCAAGCCGCTGGTTGAGCACCAATTT
TAAGTTCCAATCTGACAGATTATACACTTTATTGTAAAATTAACTGCTAGCTCACGAGAGACACAAGTTGTGGCG
GTGCAATTTAGTTCTGCTGCCCAATAATCTGAAAAATAAACTATAAGCCAGTATGTGGAATTAACCGCAAGTTGC
TATAAACACTCTTGTTGGTTCGTCTATGTAAAAGGATGAAGCTTCTGCAGCTCAAATGTGCTCTGAAATTGAAAG
AAAACTTAGTAAAACATGTGAGCTTTTGATATAGATTGAGTGATTTTGCAGTGACCAAGCGAATTGTTGAGATGG
AAACTATAGTGTAGATTAGGAATAATTTGGATGGGAGAAAAATCCAAAACTAGTTGGGGATCATTGTTAATTGAA
ATATTAGGGAGGATTGTGAGAGTGTGAAGGAAAAGTGTTGAAGAATAGAGAGATACAGATTTGTGCAGTGACAGC
GATGCGTGTTGGTGATGGAAGTGGGCCGGCACCGAGGAATCAGGCAGTCATGGCTACAGATGTTCTGGTATTTGG
TGTGTATAGGATGGACTTTTATGTGATGGGTACTATACGTGTATTTTAGAATTTGATGTGGGCTGGGTATGTGTT
GTGTTGCTTGTGTTTTGGATAGGTGGATGATAATATTTGGTTAGTGGGTTGGGTTAGGGTAATTGGTTATGGGTT
TCATTGGGCCATTGGATGTTGGCGAATGTGTGTATATGTATATGGGTTAGTGGGTTGGTGTAACAAATTAGGGTT
TGGGGAAATTATCTAGTGGGCCAGGTTGGTATGGGCTTGGTCTTGCTTGATGTATAGGCTATTGGGTTCGTATAG
GTGGGTAATATAGGGTTTCTTCGGTTGAAAGCAACCAAATTGGCCTGATCAAAAATAAATTGTACTATTTTTTTC
AGAAACTGATGAAAATACTAAAATTAAAAATTTCACGGTGTAAACAGTATCCACTAATAGTACAATGAGCAATAA
CTTCTTCTTTCTTTCTTTTTTTTTTTTTTTTTTTTTTTTTTTTTTTTGGTTGATGCCTGCTGCCACCTTTTTTGTG
GTTTAAAGTAGGTTTTGGCCGGATATGTTGGCGGCTATGGTTTGATTGTTGAATGATGAAACGGGTGGTTTAAAT
GTGGAAGTGAAATTTGTCGGTCAAGGGAAAGAAATTGAGCTCTACTACCAAATTGATGTAGGATACAATAAGGAG
AAGGGGAAAGGGGAGAGAAAAAAAAGAAATGAGGGGGGAAAAGAGAAGGGGGAGAGAATAGAGAGAAATAGGGAG
AGTATCAATTTATTTCATAATCAAAGGTTATTTCCAATTACATTGCATAGACCCTTTATGTAGGGTTTGAATTAA
TTATAATTGAAGTAAGAAAATACTCTAAATAAATCACTAATTGAATCCATCCTAATTCTCCATCAGATATGTATC
CATATTTGAAATCCAATATCTAATATCTGTTAACTAAAATACAATAATCATATATTGAATGATTATACGCATGAT
GTCTGTTGTGAGCCTATGTTTGACTCATGTTTATAAACACTGAAATAATTTTTTTCCTCACATTTCATGGGACCT
ACCAAACTACTTATTTATCCACCACTCATTTGTATCTATTTATGGTTTAAACCATTTATAAACAATTTTCATTCA
CGCTTTAAGAGATATCTGGTGCCTATTGACAATATTTAATTCCATAACCATAAAGATGTTTCACTCAACTTATTG
AGGGTATTGGCCGTAAGTAAGATCTCTTCTGTTGATATTTCAAAGTAATAAATCTTATGATTTGAATCTATTTTT
CTTTTTAAGATTAAAGACATACATGCTTTAGTAGTTTGAAGAAAACCAATCTAACTGATGCAATTGTATATGAAT
TTTCTGTCTGGGTCTTGTTCAACGCATCGAACCCCATGTACACACTGCATGTCAAACCCAAAATCTCATTTTGGC
AACCAAGATAAATCGCCCCATCCCGTAGAGGTTGACGTGCTACAATTTATGATGAGTTGTCATAACTCAACCTAA
CTGTGATCTGGTAATAAAGTTACATGGAACTCTTGATTCTATCTTCTTAGCCAGTGTACATATCCCAAGTCATGT
ACAGTTTAAATTCAATGGCATTGTAAATTTCATCTAACAAATTGCCTGCTGTAAATGTATATTGGTAATGAATTC
AAAATCTACTAAAGGTAAAGAACGATTGCAGTACTTAACTAGTTGTATACACAAGAAGTTAACCTGATAATGCAT
GTAGGATCTACTGTTTGTTGTATTGGTAAGAGATTCTTGGAGTTTCCCACGTAGGTCAAGAGGAGGTAGTTGTGT
ATGCCATGCAATACTGTGTCGAGAGCTGCTCAGTTTCAGTGTCTATCTTAACTTTTAAGGAAGGAAGAAAAACCA
CTTCACAGATTCATGACACAGATGTTCTTTCCTCTTTGGAAAACTCTGTCTGGATAGAACTTCCACGTGCTGGTG
CCTCTGCTGTTTTTACCTTTTCTCCTCCTCTTTTTTCCCTGTATTCTAAAGTAGGTCCTTTCAAAAAGTATTTGA
TGCTTTTGTTTGTATCTTTTTTCTTTGGAACTATTTGGTCCCCTTTCTTCATCTGTTACTATGGTATGCCTTTAA
GAAGATGGAATCCAATAACATTCCATCAATTGTGCCGGTTCTTTTAACGAATCCCAATTCCACTTTGGGAGAAAT
TAAAATTAATTTGTATAACTGCTGAATTAATTGGATTGTTGCAAGTGGAATGAATGGGATGCGGTCTGTTACATA
GCAAATTAACTAATTTCGAATTTATTCAATTTGAGCTTACTAATTAGCTGATCTAAACTTCTATTTAGCTACATA
TTTGAATCACTAGGCTCAAATCTTGAACTCAAATAAGTGCCGAGAACTTTTGAAGTATAACTAGCAGTTTAAAAA

TTGAATAAATTTATCAAAACTGTTCTTTAGATATTGGCAGTTTTGCCGAGCAAAGAATTGTTTAATATAATAAAA
ATTGAGGTACCGTTTCAGGACATTATAAGAGTTCTGATCTGTAACTGCTGTCAAATTTTTCTTTATGTGTTTTCT
TGAATATGGAAAGTCAAAGTTGTTCTTTGAAGTCTTATATTTGCATTTAGTTTCTTGATTCTCTTTCCTCCTAAT
CTGACTTCTCTATCCTCAAAATTTTCAGAAGATAATTTTGCTTAGTGATAGTTTCGATTCCTGTGTGTAATTCCA
TAGCTACTACGGCAAGGATTGCTGTGGTTTAGTTTAGTTTCTTGCAAGTGTGCTAACATCCAAACATCTTGTGTA
ATTCCATCATTCTTAACCTGTTCTTATTATTTTTATTAGCTATTTTCCGCAAAGCTTGTGTTAAAATTGGATGCT
AATCGTGTCTAATATATTGCAGGCATTCGGGTGGGCATATGTATATAATAAGCACAACACATGGGAGCAATTCTA
TGTTTACTGGATCTGTCCATTCACTGGAGCAATATTGGCTGCGTGGACCTTCCGCTTTCTATTCCCGCCCCCAAC
AAAGCAGGACAAGGCGAAGAAAGCC**TGA**

>*Oeu*SIP1;1_*Oeu*050292.1

ATGGGTGCTATAAAGGCAGCGGTAGCCGATGGGGTGTTGACTTTTATGTGGGTTTTTTGCGCATCGACTTTGGGC
GCACTCACTTTTGTGGTGGCTTCTGCTCTCGGAGTATCCCAAGGACTACCCACTCTTCTCGTCACTACTGTCCTT
GTTTTTGTACTGCTGTTCGTGTTCGGCATCATCGGCGATTTCTTGGGCGGCGCCACCTTCAATCCCACTGCAATC
GCTGCTTTCTATGCCGCTGGTATTGGAGGCGCCGATTCTCTCTTCTCTGCTGCCCTCCGCTTTCCTGCCCAGGCG
GCTGGTGCAGTAGGTGGTGCTCTTGCAATTATGGAGGTTATGCCAATGCAGTACAAACACATGCTTGGGGGTCCT
TCATTGAAGGTTGACCTGCACACTGGAGCTATTGCTGAGGGCGTTTTGACTTTCACAATTACCTTTGCTGTCCTT
CTCATAGTCCTTAGGGGTCCAAGCAACCCACTAGTGAAGAATTGGTTGCTTGCCATGGCAACTGTTTCGGCAGTC
GTTGCCGGTTCAAGTTATACTGGACCTTCGATGAATCCTGCCAATGCATTCGGGTGGGCATATGTATATAATAAG
CACAACACATGGGAGCAATTCTATGTTTACTGGATCTGTCCATTCACTGGAGCAATATTGGCTGCGTGGACCTTC
CGCTTTCTATTCCCGCCCCCAACAAAGCAGGACAAGGCGAAGAAAGCC**TGA**

>*Oeu*SIP1;1_*Oeu*050292.1

MGAIKAAVADGVLTFMWVFCASTLGALTFVVASALGVSQGLPTLLVTTVLVFVLLFVFGIIGDFLGGATFNPTAI
AAFYAAGIGGADSLFSAALRFPAQAAGAVGGALAIMEVMPMQYKHMLGGPSLKVDLHTGAIAEGVLTFTITFAVL
LIVLRGPSNPLVKNWLLAMATVSAVVAGSSYTGPSMNPANAFGWAYVYNKHNTWEQFYVYWICPFTGAILAAWTF
RFLFPPPTKQDKAKKA*

*Oeu*SIP1;2 *Oeu*014723.2

>*Oeu*SIP1;2_*Oeu*014723.2 | Scaffold1584:16236..19503 reverse

ATGAGGGCAATTAAGGTAGCAATCGCCGATGCGATAGTGACGTGCTTGTGGGTTTTTTGTGCATCGTCGTTGGGT
GCACTCACTTTCGTTGTGGCTTCTGCTTTTGGGGTTAGCCAAGGCCTGCCTACGCTTTTGATCACCACTTTTCTC
CTTTTTGTTCTTTTGTTTGTATTCGGATTTATTGGAGATGCGCTGGGAGGCGCCACCTTCAATCCCACAGGTCCC
GCCGCCTTTTATGCCGCCGGCGTTGGAGGCACTGAGTCTCTTGTCAGCGCCGCCGTCCGCTTTCCTGCTCAGGTG
ACCTCTTCTTGTACGATTCTCATGTTTCGTTTAATGGTCTATGTTATTGCTTTGCAAGATTATAAGTATTAGAAA
GCAAAAAAATTCAGTCGTTTTGTCATAGCCGTAGGCCATACGTCAATAATTCAATGCTGAATCATTTGGATTCCAT
TGGTGACGGGACATAGAATGTGGATTTTGAAGAGAGATTCAACCACTACAAAAAGATCGATTTTTCGGGATCCTT
TCTTAAAAGGGGAGGAAAAGGTGTAAAATCAGACCAATTCGTACAATTCCTTTCTGGATATTCCTTAGTGTTCCG
GCTATTCATGAAATGTGAGATGATGATTGTAACTGAGTCCAAATCCTTGACATCAAGCCGAGTGACTTTTGATCC
TTGCAAGATGATTCTTGGAAATTGTATGACTCGTTTTTCTTCTGTACATGACGAAGATTAGTAGATCATCTTGGA
TTGTATGCAGGGCCTGGCTCTGAGATCTTGGATGTCCAGTGTGAAATTTCAAAATGGGCCCCTTTTTTCATAAAT
ATTGTGCCCTTTAACTAACATAATAATAAAAATTTTAAATTTTAAAATTACTATATATAACAAAAAATATTTTGG
TGCCCCTACAACGTTGAGGCCTTGTGCGGTTGCCCCACCTGCGCACCCTTAGGGCCACCTCTGATTGTATGACTC
GCTTGTACTTTGTAAATGACAAAGATTAGAGGATTGCTGGCATTGAAATATAGTTTAGATTTGCAATATTTAAAA
TTTGCATTGCTTAAGTTCAGGCAGTTGAACACTTGATTCATGCTAATCTGATCATGAAGAACAATGAGGTTCCAG
AAGAGCTAAACGTGCTGTGAAAGAGTGGAATGAATGGATGGAAAAAACCAATAGACTCTTTTTGTAAAAATCGTA
AATTATTTTGTGGTGTCCTTGACAGGCAGCTGGTGCAGTAGGTGGTGCACTTGGGATTCTAGAGGTTATGCCAGT
GCAATACAAGCACATGCTAGATTGTCCTTCTTTGAAAGTCGATTTGAATACAGGAGCAATTGCTGAGGGAGTATT
GACCTTCATAATTACCTTTGCTGTCCTTTACATTGTCCTGAGGGGTCCTACCAACCCATTTGTGAAGAATTGGTT
GCTAGCTATGTCAACTGTTGCCTTAGTGGTTGCAGGTTCGAGTTATACTGGACCTTCCATGAATCCTGCAAATGT
AAGTCCCATCTCTCTTTACGTTCCCTTTTTAGATAAATCATGGGCAATTTATGGTAAATTCTTGTCTTACATGCA
ATGAATTTTCCTTCAATCAAGTTATAAGAAACTTTAGAGTGTTTCTTTGCTTAGCATAATAGCAGAAATATCCAC
ACCATTATTTTTGTTTATTTATATCTTTCAAATTCCTTGTTAGATTTTATTATATTCTTTCCTAATCATGCTTCT
CTCTGCATCAAAATTCTGATTAAGATGCCAGGTATAGATTGATCTTTGACATCTATAAGCACAACGAGTGCATGC
TTGGTGTAAACATAGATGGAAGCTAAATAACAAAATTTAAGTTTCTTTTTATTCCTAAATAGTGGTCAATGTTAA
CAATTCTTTCTAGCAAACATGCCATTAGATTATTGTCCATCAATGCTGACTGAACATATGGGATTCCACGTCTTT
CTTGCTACTTAGGTCGGAATGTAAGCTAGTAATGTTTTCGGCTCTTGTACTTCACCATCTATGGTGTTGCTTCGT
TTAGCAGCACGACTATTGTATTGCCCGCCCACAACACTGTTTCACAGTTGATCTATGATGGATCTGTTAGCCGCG
CGCATAATTTTAAGGATCTCAATTCATTGCACTGTCATGTCTTAATATTGCAATCACTCTTAATAAGCCCCCGTT
TGGCCCCCATCCCTTCAAATTTTAATGGTTGGAATAATTTTTTTTGAAAAATGTGTTGTTGAAAAAGAATGAAAA
GTTTTTGATTTGCATGTAATATTATTTTATGGAAGATTATGTAGTTGAAAATAAGAAAAAAAAGAAGAAGGAGAA
GGAGAATGGGAGCCAAACGGGGCCATAGTAGCAAGCTTCTCAACCACTCAATCTTTGCCATTTTCCCTAATTGCA
AGCTTGCAAATGACCTATTAATTCCTGCAATCCAAAACTTGAACTAGAGTTCTAAAATATGATTTTCTTTAAATT

AACTTTACCTTTATGGTGCGTTTGATTGCAAGTATGAAATTTGCATAGAAAGAAAATAAATTATAGGTAATTTAA
TTGCCCTAGAACTTATTTGTATGAAAATTATCAATTAAATGTGTTTGATTGATTTAATTTTTAGCCTTAAAAATA
TTATACTTGTCCAATTTTTATTTGTGGTTAGATGAGAGAGTGGATGAATAAGAAAGTGAAAGGAGAAAATTCCGT
TGGAAAATAAAATCCTACTACACCTAAAGAAAACATTTTCCTTGATTTTGAGGAAAATATGGTCACATGACCAAA
AGTAGGAAAATGTTTTTCATGGAAAAAAGTCAATCAAACAGTATAAAAGTAGGAATTTGGGTGAAAAAATACATT
TTTCATGGAAAATATTGACAATCAAACGGACCCTTAGTTTTCGATACATTCATAAGTTTGAATTTAATATCACTA
TAACAAACGAGCTAATCAGTCATTCAACTTTGCAACATATGTGCTGATTTTCAGAAAGTTTCCATACATTGTTGT
TTTTATCACTTGTTATTGTTCTCCTATCCCTTCTGCTTATTTTCGTGCTCTTTATCTATAAATGGATCATATGCT
CCTTATCATACATCTTAATCTATATCTAATATCTTTGCAGGCCTTTGGCTGGGCATATGTAAACAATACGCACAA
AACATGGGAGCAATTCTACGTCTACTGGATCTGCCCCTTCATTGGAGCAATATTAGCTGCGTGGGTTTTCCGTTT
TCTATTTCCGCCTCCTCCAGTCAAGAAGCAGAAGAAAGCC**TGA**

>*Oeu*SIP1;2_*Oeu*014723.2

ATGAGGGCAATTAAGGTAGCAATCGCCGATGCGATAGTGACGTGCTTGTGGGTTTTTTGTGCATCGTCGTTGGGT
GCACTCACTTTCGTTGTGGCTTCTGCTTTTGGGGTTAGCCAAGGCCTGCCTACGCTTTTGATCACCACTTTTCTC
CTTTTTGTTCTTTTGTTTGTATTCGGATTTATTGGAGATGCGCTGGGAGGCGCCACCTTCAATCCCACAGGTCCC
GCCGCCTTTTATGCCGCCGGCGTTGGAGGCACTGAGTCTCTTGTCAGCGCCGCCGTCCGCTTTCCTGCTCAGGCA
GCTGGTGCAGTAGGTGGTGCACTTGGGATTCTAGAGGTTATGCCAGTGCAATACAAGCACATGCTAGATTGTCCT
TCTTTGAAAGTCGATTTGAATACAGGAGCAATTGCTGAGGGAGTATTGACCTTCATAATTACCTTTGCTGTCCTT
TACATTGTCCTGAGGGGTCCTACCAACCCATTTGTGAAGAATTGGTTGCTAGCTATGTCAACTGTTGCCTTAGTG
GTTGCAGGTTCGAGTTATACTGGACCTTCCATGAATCCTGCAAATGCCTTTGGCTGGGCATATGTAAACAATACG
CACAAAACATGGGAGCAATTCTACGTCTACTGGATCTGCCCCTTCATTGGAGCAATATTAGCTGCGTGGGTTTTC
CGTTTTCTATTTCCGCCTCCTCCAGTCAAGAAGCAGAAGAAAGCC**TGA**

>*Oeu*SIP1;2_*Oeu*014723.2

MRAIKVAIADAIVTCLWVFCASSLGALTFVVASAFGVSQGLPTLLITTFLLFVLLFVFGFIGDALGGATFNPTGP
AAFYAAGVGGTESLVSAAVRFPAQAAGAVGGALGILEVMPVQYKHMLDCPSLKVDLNTGAIAEGVLTFIITFAVL
YIVLRGPTNPFVKNWLLAMSTVALVVAGSSYTGPSMNPANAFGWAYVNNTHKTWEQFYVYWICPFIGAILAAWVF
RFLFPPPPVKKQKKA*

***Oeu*SIP2;1 *Oeu*060749.1**

Observation: 3′ extended by 261n, generating 1 intron and 1 exon

>*Oeu*SIP2;1_*Oeu*060749.1 chr9:9170322..9172233 forward Original version

ATGGCTGCCGTAGGAGGAAGGCTGCTGCTAGTGTCGGATTTCTTTATGTCGTTCATGTGGGTTTGGTCTAGCGTG
CTTATCAAGATTTTTGTGTATAAAATATTGGGCTATGGCACCCATGATTTGAAGGGTGAAATTCTGAGGTATGGA
ATCTCCCTTGTCAACATGTTCTTCTTTGCATACTTGGCCAAAGCCACCAAGGGTGGGGCCTACAACCCCCTCACC
ATCTTGTCCTCCGCCATCTCTGGGGATTTCAGCAATTTCCTCTTCACTGTCGGGGCCAGAATTCCTATCCAGGTT
CGCTGAGTGCAAATGTTTTAACTCTTGTTTCTCTAATTATTATGCTATTTTTCGTTTTAGGAAACATTGTGGCTC
GATAAATTTGGCGATTGATGCGTGTAATTATGTGCATAAATTGACGTTTTGTTTGGTGTGACATTGAATTAGAGA
AATTCGATGATGATTCTTCTCTTTATTTTCAGGAATGCTTGCTTTTGGGTTGCTCGTAAATTCCGATTAGGGCAT
GCAATAAATCTCTGAGTTTAAGCCAAAAATTTCTATTTCTTTTTTGGAAAGTGAATTGATCTAATATCTAAAACC
TTATACAGTGTAGGTAGGTAAAAGTAATTTTATTACTGAAAGAGCTTAAGAAATTTAGGGATTTGACCTTGAAAG
AAGATTATTCTCAGTTAATGTTAGTTAAGCTAAATCTTGTGGTAAAAAAAAAGTCTCATTGCCATATATTCAAGA
CTCATTCTCAGTTAATAATTTATGAAAACTGATGTGTGTTTTGTTGATTGATTTGTACGAACTAAAGATTGCAAT
ATGATTAAGAGGTCTAACTTACAATACTGTACTTGTGGAAGAAGTGGGACAGTGGAGATATCTTGTTAGTTTGGC
AAATTCTTTGCTGATAATTCTGTAGAACCGAGCTCCACGTACAGGACTTTCTAATTCTGTATCTACATGTAGGAC
TTTCTTATTCGAGGTTTCTAATTTGGAATTGTTCTCAACTAGTCGAATTCTTTTCACCACATAGTTTTTGTACCA
TTATCTTCTTATTTAGCCCCGTTTGATGGGCTTTTCTTCAAAAGTCTTTTATCTATATACTCATTTTCATCCACA
CATTCACACAATAAAATATTATGTACGTAAACTCAAAACTTTTCCTTACTTTTTAACTATACATTCTCCAAAAAT
TCGCTATTCAAAGCATTATTTTTCCCAAGTAATGGCCATCAATCAGGGGCTTAGAGAACATCCCTTTTCCCGTGT
TGTCACTGCTGCAGTGCTTCTAGTTTCTTTCTCTCTCAGAGAGGTTATGAAGAGTAGTAATTTTCAAAACTCATA
GTTTATTCATAATCGTCTGATAGTCATCTGGAAATAAAAGACTCGTCATGCTTTTTAAATGACTTCTGGAAGTTG
AGTTAAAGATGATTGCCCCGTTTGTTTTCCATTAAAATGAAAAATATGATCGTTATCCATCACATCAAGGCACCA
AATTTGAATTTCTATCTCTGTGGGGCCCATAACGACTATATTTTTCATTTTCATTTTTGCAAAAATAAGAAACAA
ATGCCCCCTTAGTTTTTTTACCGGCGCATACCCTTATTTTTGTTCTTCCATCTCTCTTTTAAAGGTCTTGGGGTC
TATTTATGGAGTTAGGTTCATTCTCGCGGCATTTCCTGAAATAGGACGTGGGCCACGTCTGAATGTTGACATAGC
TCGAGGTGCATTGACTGAAGGTCTTTTGACATTTGGGATTGTGATTATATCCCAAGGGCTCTCGAGAAAAATTCC
CGGAAGTTTCTTCATGAAGACTTGGATCTCCAGCGTCTCAAAGCTCACTCTTCACATTCTCGGCTCTGATCTGAC
TGGAGGATGCATGAATCCAGCCTCTTTAAGTACT**TAA**

>*Oeu*SIP2;1_*Oeu*060749.1 | chr9:9170322..9172494 forward Corrected version
ATGGCTGCCGTAGGAGGAAGGCTGCTGCTAGTGTCGGATTTCTTTATGTCGTTCATGTGGGTTTGGTCTAGCGTG
CTTATCAAGATTTTTGTGTATAAAATATTGGGCTATGGCACCCATGATTTGAAGGGTGAAATTCTGAGGTATGGA
ATCTCCCTTGTCAACATGTTCTTCTTTGCATACTTGGCCAAAGCCACCAAGGGTGGGGCCTACAACCCCCTCACC
ATCTTGTCCTCCGCCATCTCTGGGGATTTCAGCAATTTCCTCTTCACTGTCGGGGCCAGAATTCCTATCCAGGTT
CGCTGAGTGCAAATGTTTTAACTCTTGTTTCTCTAATTATTATGCTATTTTTCGTTTTAGGAAACATTGTGGCTC
GATAAATTTGGCGATTGATGCGTGTAATTATGTGCATAAATTGACGTTTTGTTTGGTGTGACATTGAATTAGAGA
AATTCGATGATGATTCTTCTCTTTATTTTCAGGAATGCTTGCTTTTGGGTTGCTCGTAAATTCCGATTAGGGCAT
GCAATAAATCTCTGAGTTTAAGCCAAAAATTTCTATTTCTTTTTTGGAAAGTGAATTGATCTAATATCTAAAACC
TTATACAGTGTAGGTAGGTAAAAGTAATTTTATTACTGAAAGAGCTTAAGAAATTTAGGGATTTGACCTTGAAAG
AAGATTATTCTCAGTTAATGTTAGTTAAGCTAAATCTTGTGGTAAAAAAAAAGTCTCATTGCCATATATTCAAGA
CTCATTCTCAGTTAATAATTTATGAAAACTGATGTGTGTTTTGTTGATTGATTTGTACGAACTAAAGATTGCAAT
ATGATTAAGAGGTCTAACTTACAATACTGTACTTGTGGAAGAAGTGGGACAGTGGAGATATCTTGTTAGTTTGGC
AAATTCTTTGCTGATAATTCTGTAGAACCGAGCTCCACGTACAGGACTTTCTAATTCTGTATCTACATGTAGGAC
TTTCTTATTCGAGGTTTCTAATTTGGAATTGTTCTCAACTAGTCGAATTCTTTTCACCACATAGTTTTTGTACCA
TTATCTTCTTATTTAGCCCCGTTTGATGGGCTTTTCTTCAAAAGTCTTTTATCTATATACTCATTTTCATCCACA
CATTCACACAATAAAATATTATGTACGTAAACTCAAAACTTTTCCTTACTTTTTAACTATACATTCTCCAAAAAT
TCGCTATTCAAAGCATTATTTTTCCCAAGTAATGGCCATCAATCAGGGGCTTAGAGAACATCCCTTTTCCCGTGT
TGTCACTGCTGCAGTGCTTCTAGTTTCTTTCTCTCTCAGAGAGGTTATGAAGAGTAGTAATTTTCAAAACTCATA
GTTTATTCATAATCGTCTGATAGTCATCTGGAAATAAAAGACTCGTCATGCTTTTTAAATGACTTCTGGAAGTTG
AGTTAAAGATGATTGCCCCGTTTGTTTTCCATTAAAATGAAAAATATGATCGTTATCCATCACATCAAGGCACCA
AATTTGAATTTCTATCTCTGTGGGGCCCATAACGACTATATTTTTCATTTTCATTTTTGCAAAAATAAGAAACAA
ATGCCCCCTTAGTTTTTTTACCGGCGCATACCCTTATTTTTGTTCTTCCATCTCTCTTTTAAAGGTCTTGGGGTC
TATTTATGGAGTTAGGTTCATTCTCGCGGCATTTCCTGAAATAGGACGTGGGCCACGTCTGAATGTTGACATAGC
TCGAGGTGCATTGACTGAAGGTCTTTTGACATTTGGGATTGTGATTATATCCCAAGGGCTCTCGAGAAAAATTCC
CGGAAGTTTCTTCATGAAGACTTGGATCTCCAGCGTCTCAAAGCTCACTCTTCACATTCTCGGCTCTGATCTGAC
TGGAGGATGCATGAATCCAGCCTCTTTAAGTACTTAATAATCATTCTATGCATAATCTATGTTTACTGCCGAATT
TTGTTGGATGCACACTGAGTAAAATTTTAATTGCATCTCCCAGGTAATGGGGTGGGCTTTTGCACACGGAGAACA
TATAAGCAAGGAGCATTTAATTGTGTACTGGCTTGCTCCTATGGAGGCAACTTTGGCAGCAGTGTGGGCATTTAG
ACTGATAGTTCGGCCCAAGAAAGACGAGACACTAAAGGCGAAGTCCGTAAAATCAGATTGTAAGAACAGTTGA
>*Oeu*SIP2;1_*Oeu*060749.1
ATGGCTGCCGTAGGAGGAAGGCTGCTGCTAGTGTCGGATTTCTTTATGTCGTTCATGTGGGTTTGGTCTAGCGTG
CTTATCAAGATTTTTGTGTATAAAATATTGGGCTATGGCACCCATGATTTGAAGGGTGAAATTCTGAGGTATGGA
ATCTCCCTTGTCAACATGTTCTTCTTTGCATACTTGGCCAAAGCCACCAAGGGTGGGGCCTACAACCCCCTCACC
ATCTTGTCCTCCGCCATCTCTGGGGATTTCAGCAATTTCCTCTTCACTGTCGGGGCCAGAATTCCTATCCAGGTC
TTGGGGTCTATTTATGGAGTTAGGTTCATTCTCGCGGCATTTCCTGAAATAGGACGTGGGCCACGTCTGAATGTT
GACATAGCTCGAGGTGCATTGACTGAAGGTCTTTTGACATTTGGGATTGTGATTATATCCCAAGGGCTCTCGAGA
AAAATTCCCGGAAGTTTCTTCATGAAGACTTGGATCTCCAGCGTCTCAAAGCTCACTCTTCACATTCTCGGCTCT
GATCTGACTGGAGGATGCATGAATCCAGCCTCTGTAATGGGGTGGGCTTTTGCACACGGAGAACATATAAGCAAG
GAGCATTTAATTGTGTACTGGCTTGCTCCTATGGAGGCAACTTTGGCAGCAGTGTGGGCATTTAGACTGATAGTT
CGGCCCAAGAAAGACGAGACACTAAAGGCGAAGTCCGTAAAATCAGATTGTAAGAACAGTTGA
>*Oeu*SIP2;1_*Oeu*060749.1
MAAVGGRLLLVSDFFMSFMWVWSSVLIKIFVYKILGYGTHDLKGEILRYGISLVNMFFFAYLAKATKGGAYNPLT
ILSSAISGDFSNFLFTVGARIPIQVLGSIYGVRFILAAFPEIGRGPRLNVDIARGALTEGLLTFGIVIISQGLSR
KIPGSFFMKTWISSVSKLTLHILGSDLTGGCMNPASVMGWAFAHGEHISKEHLIVYWLAPMEATLAAVWAFRLIV
RPKKDETLKAKSVKSDCKNS*

***Oeu*SIP2;2 *Oeu*Chr11**

>*Oeu*SIP2;2_chr11 chr11:18362597..18364222 Reverse
ATGGCGGCCGTAAGAGGTAGGCGGCTGCTGGTGTCGGATTTCTTGATGTCTTTCATGTGGGTTTGGTCCAGTGTG
CTCATTAAGATTTTTGTTTATAAAATATTGGATTATGGCTCCCATGATATGAAGGGTGAAATTTTGAGGTATGGA
ATCTCCATTGTCAACATGTTCTTCTTTGCATTCTTGGCCAAAGCTACCAAGGGTGGGGCCTACAACCCCCTCACC
GTCTTGTCCTCGGCCATTTCTGGGAATTTCAGCAATTTCCTCTTCACTGTCGGGGCCAGAATTCCTGTCCAGGTT
CGCTAGATACAATTGTTTTAAAATCTTGTTCTTTAATTATCATGTCACCGTTTGTTTTAGGAAATATTGACAGTT
TCTTTTGTGAGGGACATTGAATTAGAGAAGTTAGATTGATGGTTCTTCTCTTTATTTTAGGACTTCATGTTTTTG
GGTTAATCGTAAATTTGATTAGGGCCTGCATCTTAAATCAATCTGGGAGTTTCAGTAAATACGTTCTATTGATTA
TTTGGCAAGTGAATTGATAGATAATCTGAAACCATATACATGGTAGGCTTAGTGAAAGCAGCAATTTCAATGATT
GTCACATGTTATTGGAATATCTTAAGAATCATTCAAAAATCGAAGAAATGATCAAAACAAAATGATTTTTGAATT
GTATTCCAGAAAAATTTTATTCACAAATAAAACTATATCTCGTTATCTTGGCAAATTATTTGAGGCTAATTATAT
ATCTGCATGTAGGACTTTCTTTCTTATCCAAGATTCCCCCAATTCGACATCTTTCTCAAATATCAACTATAAAAA
ATTCTTTCCCCACTTACTTTGTGTTCCAATATCTATACTTATTTAGAGAGTTTCCCTTTTTCCCATTTGTTAAT

GCTGACACGATGAATGGATCGTTGTTCTCCAGAAAAACCCTCAGACAGGTTGTGAAGAGGAATGACATGGTGTTC
TTAAAATAAGTGTTCCAAAACTCATAGTTTGTTCATAATCATCATCTGGAAGTTGAGTTAAGATGGTTTAGTTGT
GTACTGGAGCTTATCCTTGTTCTTCCATTTCTTTTTCAAAGGTCTTGGGGTCTATTTATGGGGTCAGGCTCATTC
TTGCGGCATTTCCAGAAATAGGACGTGGGCCACGTCTGAATGTTGACATAACTCGAGGTGCATTGACTGAAGGGT
TTTTGACGTTTGGAATTGTAATTATATCCCAAGGGCTCTCGAGAAAACTTCCTGGAAGTTTCTTCATGAAGACTT
GGATCTCAAGCGTCTCGAAGCTCACTCTTCACATGCTTGGCTCTGATCTGACTGGAGGATGCATGAATCCGGCAT
CTGTAAGTACTTATATTCAGTCTAAATGTAATCTATGCTTACTGCGGAATTGTGTTGGATACATTACACTTGAAT
GAACATCGAGTAAATTCTAATTGCGTCCCTCAGGTAATGGGGTGGGCTTTTACACGCGGGAACATATAAGCAAG
GAACATTTAATTGTATACTGGCTTGCTCCTATGGAGGCGACTTTGGCAGCAGTGTGGGCATTTAGAGTTGTAGTT
CGACCCGAGAATGATAAGACACCTAAGGTGAAGTCCGGAAAATCAGAA**TGA**

>*Oeu*SIP2;2_chr11

ATGGCGGCCGTAAGAGGTAGGCGGCTGCTGGTGTCGGATTTCTTGATGTCTTTCATGTGGGTTTGGTCCAGTGTG
CTCATTAAGATTTTTGTTTATAAAATATTGGATTATGGCTCCCATGATATGAAGGGTGAAATTTTGAGGTATGGA
ATCTCCATTGTCAACATGTTCTTCTTTGCATTCTTGGCCAAAGCTACCAAGGGTGGGGCCTACAACCCCCTCACC
GTCTTGTCCTCGGCCATTTCTGGGAATTTCAGCAATTTCCTCTTCACTGTCGGGGCCAGAATTCCTGTCCAGGTC
TTGGGGTCTATTTATGGGGTCAGGCTCATTCTTGCGGCATTTCCAGAAATAGGACGTGGGCCACGTCTGAATGTT
GACATAACTCGAGGTGCATTGACTGAAGGGTTTTTGACGTTTGGAATTGTAATTATATCCCAAGGGCTCTCGAGA
AAACTTCCTGGAAGTTTCTTCATGAAGACTTGGATCTCAAGCGTCTCGAAGCTCACTCTTCACATGCTTGGCTCT
GATCTGACTGGAGGATGCATGAATCCGGCATCTGTAATGGGGTGGGCTTTTACACGCGGGAACATATAAGCAAG
GAACATTTAATTGTATACTGGCTTGCTCCTATGGAGGCGACTTTGGCAGCAGTGTGGGCATTTAGAGTTGTAGTT
CGACCCGAGAATGATAAGACACCTAAGGTGAAGTCCGGAAAATCAGAA**TGA**

>*Oeu*SIP2;2_chr11

MAAVRGRRLLVSDFLMSFMWVWSSVLIKIFVYKILDYGSHDMKGEILRYGISIVNMFFFAFLAKATKGGAYNPLT
VLSSAISGNFSNFLFTVGARIPVQVLGSIYGVRLILAAFPEIGRGPRLNVDITRGALTEGFLTFGIVIISQGLSR
KLPGSFFMKTWISSVSKLTLHMLGSDLTGGCMNPASVMGWAFTRGEHISKEHLIVYWLAPMEATLAAVWAFRVVV
RPENDKTPKVKSGKSE*

OeuSIP-related, considered as Pseudogenes

*Oeu*061896.1 *Oeu*SIP1-related

>*Oeu*061896.1 | chr22:4366217..4368873 forward

Incomplete sequence

>*Oeu*061896.1

ATGGGTGCTATTAAAGCAGCGGTAGCCGATGGGGTGCTGACTTTTATGTGGGTTTTTTAAACATCGACGTTGGGC
CGCACTCACTTTTGTGGTAGCTTCTGCTCTCGGAGTATCCCAAGGCCTACCCACTCTTTTCATCACTACTGTTCT
CGTTTTTCTCCTGTTGATCTTGTTCGGCATCATCGGCGATGTATTGGGAGGCGCTACCTTCAATCCCACTGCCCT
CGCCGCCTTCTATGCCGCTGGTATTGGGGGCGCTGATTCGCTAGTCTCTGCCGCCCTCCGATTTCCTGCCCAGGC
ATGACTTGCCTACTCTTCATTTTTAGATTAGTCAGATATTAAACACAGTATGCAATCATCCTACTGACTTGATTC
AAACGTAGATTAATAAGTGGAAATTCTCAATTGATGATATGTTTGTGAAAATCTAGATTGCTCAGGGCTAGGCTA
GATATAATGAAACTCGAGATAATAAGAATAAAAAATGAACTTCCAATTTTTTAAAAAATTCACAGATTTTAACTG
AACGACCAAATTACAACAAAAGGCAAACTTGAATAATGAGTTTGCACAAAATAAAGTTCGGTGACTAATGCGAAG
CTTTGCCATAAGTTAAGTAAAAGTTATGCAAGGAATGAGAATGGTAAAGCCGTTACTTGTATCCTTGACAGGCAG
CTGGTGCAGTAGGTGGGGCTCTTGCAATTATGGAGGTTATGCCGATGCAGTACAAACACATGCTTGGGGGTCCTT
CTCTGAAGGTTGACTTGCACACTGGAGCCATTGCTGAGGGAGTTTTGACGTTCACGATCTCTTTTGCTGTCCTTC
TCATCGTCCTTAGGGGTCCAAGCAGCCCACTTGTGAAGAATTGGTTGCTTGCCATGTCAACTGTTTCGCTAGTCG
TTGCCGGTTCAAGTTATACTGGACCTTCGATGAATCCTGCAAATGTAAGTGGTGCATCTCTTTTCCGTTCTTTTG
CTAGATTTTGAAATAGTATAAGATGATATATCTATGTCTTATTGTAGAATATTCTAAATCCTTTAAAATTGCAGA
CTAAAGAAACAAAATAACATGAGGTGAGAGTTTCTCCTTCCTATAGCACAAAATGAAAAGATATCTGCCAAACAT
TTTGTATTTAACTTTTTTATTTCATATCCCAAATGCCAACTTAGAATATAGAAGTTACTTTTCCCATTGATGGTA
CATGATTGTGTATCTTTTGCAATCAAAGTGAGATTGGTAATGTGATCGAGCTTTGACATTCGGAGCACAATCAGA
GTACTGTTCTCTTGACTTAATTTTTGGATTAGGAATGTGATTTGGTCCCCATATTTACATTTATGGGGTTCACTC
ATCCCATCATTATTGTCCCGTTATGGGGTGTCAAATTATCGAGATCCTCAACTACCAGGTTTCTATATGAGAACT
TTAGGAAAGAATGCTACTAGATTCTATTTTCCTCTATGCACCAAACTGTAACTGATCCTCAAATGCCGTCACCCT
AATTAGGACTTTGTAGCACACAGTAATACTTTCCTTTACACTCACGTTAAAATTTGTTTTGGAAGATCCATCTTT
CTTTAACTTCTGACGTCTTTCGACGTGATTGGATTTTTCTCCTTCCTTTGGAAGAACCAATCATTTTCTTATTGA
GACCCGAAAACACCAGCTTGATTAATTAACTTTCCAATTCAAAGAGCTTTAACTTTGCTCCTTTTTCTTCAATCA
TGTATGTCTTCAAAATAATGAATGGGACACAGTCTGTTACAAAGCCAAATAATAATTTAGACTTTGATTCATGTT

TAGCTTATCTTAAAGTCTCTTTAGTTACATTTGCAAATTGCTAATCTCAAATCGTGAACTAAAAATGTGCGGAGA
ACTTTTGAAGCATAACTTTAACATTTTTAAGCAGTAATTAATTAGTCAAAACTGCTCTCTAGATATTGGCTATAT
GCCAAATTTTTAGGTAGAGTTTTAGGACATTATAAGAGTTTTGATCTGTAACTGCGGTCAAATTGTTCTTTCTGT
GTATTTTCTTGAATCTGGAAAGTCAAAATTGTTCTTTGAAGTCTTATATTTGCATCTTATTTCTTGATTCTCTTC
TTCCCTAATCTGACTTATCTATTCTCAAAATTGTTAGAAGATATTTTGCAAAGTGATAGTTTTGAGCATGTGCAG
TCAGAAGTTTCTTAGTGTAGAATACTTCTGATTTTCATGCCCTTCAATTAAGTTGCCAAACATAGTTTTCGGATT
CTAGTTTTGACATTTGTAATGTGGTTTACATGGGACGGCTTAGCCTCACAGAGAAAAGCTACAACCCTGGAGTTC
AAATCACTTTTAGTTCCATAACTACTGCAAAATGGATTGGCTGTGCTTTAGTCTGGTTTCTTAGAACAAGTTTGC
TAATATCAGAACATCTTGTGCAATTCTTTCTTTCTTAATTTGATATTTCTCGCAAAACTTGTGTTATAACTGGCT
TTCAATTGTGCCTAATATCATTGCAGGCGTTTGGGTGGGCTTATGTACATAATAAGCACAACACATGGGAGCAAT
TCTATGTTTATTGGATCTGTCCCTTCGTAGGAGCAATATTGGCTGCGTGGACATTTCGCTTTCTATTCCCGCCCC
CATCAAATCAGCACAAGGCGAAGAAAGCC**TGA**

>*Oeu*061896.1

ATGGGTGCTATTAAAGCAGCGGTAGCCGATGGGGTGCTGACTTTTATGTGGGTTTTTGCAGCTGGTGCAGTAGGT
GGGGCTCTTGCAATTATGGAGGTTATGCCGATGCAGTACAAACACATGCTTGGGGGTCCTTCTCTGAAGGTTGAC
TTGCACACTGGAGCCATTGCTGAGGGAGTTTTGACGTTCACGATCTCTTTTGCTGTCCTTCTCATCGTCCTTAGG
GGTCCAAGCAGCCCACTTGTGAAGAATTGGTTGCTTGCCATGTCAACTGTTTCGCTAGTCGTTGCCGGTTCAAGT
TATACTGGACCTTCGATGAATCCTGCAAATGCGTTTGGGTGGGCTTATGTACATAATAAGCACAACACATGGGAG
CAATTCTATGTTTATTGGATCTGTCCCTTCGTAGGAGCAATATTGGCTGCGTGGACATTTCGCTTTCTATTCCCG
CCCCCATCAAATCAGCACAAGGCGAAGAAAGCC**TGA**

>*Oeu*061896.1

MGAIKAAVADGVLTFMWVFAAGAVGGALAIMEVMPMQYKHMLGGPSLKVDLHTGAIAEGVLTFTISFAVLLIVLR
GPSSPLVKNWLLAMSTVSLVVAGSSYTGPSMNPANAFGWAYVHNKHNTWEQFYVYWICPFVGAILAAWTFRFLFP
PPSNQHKAKKA*

*Oeu*050293.1 *Oeu*SIP-related

>*Oeu*050293.1 | chr4:9356974..9357960 reverse

Incomplete sequence

Intron 2 and Exon 3 truncated

>*Oeu*050293.1

ATGGGTGCTATAAAGGCAGCGGTAGCCGATGGGGTGTTGACTTTTATGTGGGTTTTTTGCGCATCGACTTTGGGC
GCACTCACTTTTGTGGTGGCTTCTGCTCTCGGAGTATCCCAAGGACTACCCACTCTTCTCGTCACTACTGTCCTT
GTTTTTGTACTGCTGTTCGTGTTCGGCATCATCGGCGATTTCTTGGGCGGCGCCACCTTCAATCCCACTGCAATC
GCTGCTTTCTATGCCGCTGGTATTGGAGGCGCCGATTCTCTCTTCTCTGCTGCCCTCCGCTTTCCTGCCCAGGTA
TGAATCATGTGATTAGTCAGATATTGATCATGTTTCTTGAGAGTATGTAATCCTACTTGCTTGATTCAAATGTAG
ATTAGTATGTGAATATTGTCAGTCGATGATATGTTTGTGAAAATCTAGATTGCTTAGGGCTAGATCTATATAATG
GAATTTAAGATTTTAAAAAAAAGGAGGTCTCCCTTGACAGATGATATAGTTGAGAAAACTAACAGATTTGAACTG
AACGACCAAATTTAAATAAAAGGCACACATGAATAATGAGTTTGCAAATAATAAACTTTGGTGACTAACACGAAA
ATTTTACCACAATTTAAGTGTATACTATGCAGGGAATGAAAATGCTAAAGTTATTACTTTTATCCTTGACAGGCG
GCTGGTGCAGTAGGTGGTGCTCTTGCAATTATGGAGGTTATGCCAATGCAGTACAAACACATGCTTGGGGGTCCT
TCATTGAAGGTTGACCTGCACACTGGAGCTATTGCTGAGGGCGTTTTGACTTTCACAATTACCTTTGCTGTCCTT
CTCATAGTCCTTAGGGGTCCAAGCAACCCACTAGTGAAGAATTGGTTGCTTGCCATGGCAACTGTTTCGGCAGTC
GTTGCCGGTTCAAGTTATACTGGACCTTCGATGAATCCTGCCAATGTAAGTGACATATCTCTTTTCCATTCTATC
GCTAGATTC**TGA**

>*Oeu*050293.1

ATGGGTGCTATAAAGGCAGCGGTAGCCGATGGGGTGTTGACTTTTATGTGGGTTTTTTGCGCATCGACTTTGGGC
GCACTCACTTTTGTGGTGGCTTCTGCTCTCGGAGTATCCCAAGGACTACCCACTCTTCTCGTCACTACTGTCCTT
GTTTTTGTACTGCTGTTCGTGTTCGGCATCATCGGCGATTTCTTGGGCGGCGCCACCTTCAATCCCACTGCAATC
GCTGCTTTCTATGCCGCTGGTATTGGAGGCGCCGATTCTCTCTTCTCTGCTGCCCTCCGCTTTCCTGCCCAGGCG
GCTGGTGCAGTAGGTGGTGCTCTTGCAATTATGGAGGTTATGCCAATGCAGTACAAACACATGCTTGGGGGTCCT
TCATTGAAGGTTGACCTGCACACTGGAGCTATTGCTGAGGGCGTTTTGACTTTCACAATTACCTTTGCTGTCCTT
CTCATAGTCCTTAGGGGTCCAAGCAACCCACTAGTGAAGAATTGGTTGCTTGCCATGGCAACTGTTTCGGCAGTC
GTTGCCGGTTCAAGTTATACTGGACCTTCGATGAATCCTGCCAATGTAAGTGACATATCTCTTTTCCATTCTATC
GCTAGATTC**TGA**

>*Oeu*050293.1

MGAIKAAVADGVLTFMWVFCASTLGALTFVVASALGVSQGLPTLLVTTVLVFVLLFVFGIIGDFLGGATFNPTAI
AAFYAAGIGGADSLFSAALRFPAQAAGAVGGALAIMEVMPMQYKHMLGGPSLKVDLHTGAIAEGVLTFTITFAVL
LIVLRGPSNPLVKNWLLAMATVSAVVAGSSYTGPSMNPANVSDISLFHSIARF*
