# Supplementary material for: Genome Wild Analysis and Molecular Understanding of the Aquaporin Diversity in Olive Trees (Olea Europaea L.)
Source: Int J Mol Sci. 2020 Jun 11;21(11):4183. doi: 10.3390/ijms21114183 (PMC7312470; doi:10.3390/ijms21114183)
Supplement: Supplementary file 1 [file ijms-21-04183-s001.zip › ijms-821530_Supplementary_files/Figure S2_Faize_OleurAQP_sequences.pdf]

Figure S2 Detailed *Oleur*AQP sequences (genomic, CDS and protein sequences) from the domesticated olive species *Olea europaea* cv. Picual.

Sequences are presented in their original version, and if need be, in their corrected versions.
Only the "corrected" sequences were used for analysis.
For the genomic sequences, the introns are in blue.
Codons "Stop" and "Start" are in red.
All sequences are classified according to their subfamily.

***Oleur*PIP1;1 Oleur061Scf2846g07022.1**

>Oleur061Scf2846:701124..702805 (- strand) class=gene length=1682

>*Oleur*PIP1;1_Oleur061Scf2846 Original version
ATGGAGAGCAAGGAGGAGGATGTACGAGTGGGAGCCAACAAATTCGCCGAGAGGCAGCCGATTGGCACGGCTGCT
CAAAGCCAGGACAAGGACTACAAGGAACCACCACCGGCGCCACTTTTCGAGCCGGGTGAGCTGTCCTCGTGGTCG
TTCTACAGAGCTGGCATAGCAGAGTTCATAGCCACTTTCTTGTTCCTTTATATTACAGTTTTGACAGTGATGGGA
GTTAGTAAGTCTGATTCCAAGTGCAAAACTGTTGGAATCCAAGGTATTGCTTGGGCATTTGGTGGCATGATCTTT
GCCCTTGTTTACTGCACTGCAGGAATTTCAGGCAAGTTCTTTTCTATATATATAATTTGCAAATTTTGCTTGTAT
TTGCTATGATCTTCAACAGTAACGATCCGTCTTAAAGCTCAAAGAGGGAGTCGTCAGATTTGAAGATTGGTTTGT
GGGGTAGATATGAAAATACTAACCAACATATATATTGGATTATGGTCTCTGTCTCTGCAGTTTAGTCTTTGATAG
TATATGGAATTTAGGTTTTGCCATTAAAATTAGAAATTACCTAATTCAAAATTGAGTTGATTTTTCATGAATTTC
AGTTATAAGGTTTTAATTCCATTTGATATTAACATGGCAATTCAGAAAACCAAATCGTTTCAGCAATCAAATTTA
TGATGAACAATTTTAACAGTACTACTAACCTATTTTCTTCAAAGAAAAGGGCAAAATTTTAATGAACTGCTTGTT
TTGCAGGGGGGCATATAAATCCGGCAGTGACTTTCGGGCTGTTTTTGGGGAGGAAATTGTCGTTGACCCGGGCTG
TGTTCTACATGGTGATGCAGTGTCTTGGAGCCATATGTGGTGCTGGTGTGGTTAAGGGATTCGGAAAGACCCTGT
ACATGACCAAGGGTGGTGGTGCTAATGTAGTAGCCCACGGTTACACCAAGGGCGATGGCCTAGGTGCTGAAATAA
TCGGAACTTTTGTGCTCGTTTACACTGTCTTCTCTGCTACTGATGCCAAACGTAGCGCCAGAGACTCGCACGTTC
CTGTAAGCACTCCTCTTGTCTTTGTTGATTGAGCTTGAACAAGAATTTGCTTGTGTATCTGTAATCAAATTGTCT
TTTCGTTTTGTAAGAACTGATGAATCAAAAAATGTCATTGTTTATCAAGAAAACGTTTGATTAATTGGATTCCAT
ATGCTGTAATTCAATGGTTTTCAGATATTGGCCCCCTTGCCTATTGGGTTTGCTGTGTTCTTGGTGCACTTGGCA
ACCATCCCTATTACCGGTACAGGTATTAACCCCGCCAGGAGTCTTGGTGCAGCAATCATCTACAACAAAGACAAG
GCATGGGATGATCACGTAAGCTAAAAATTCAACTGTCTGAATTCAACTATTGAAATATTTGAGTTAAGGGCATAC
CTAGACAATTATATCCTAACAAATTTTTACGTTGTTCACTTATCACAATCCTCCTTTTATCTATTTTGGGACTAC
GACGAAATTTTTTAATAATCTGGCAGATGAGATGATATGATTGTCTAAAGTTTGTTCTCACTCTCATCTTGCTCT
GATATGATAATATGCAGTGGATATTCTGGGTTGGACCGTTCATTGGGGCAGCACTTGCAGCTCTATACCATGTGG
TGGTGATCCGAGCCATCCCATTCAAAAAA**TAA**
>*Oleur*PIP1;1 corrected
ATGGAGAGCAAGGAGGAGGATGTACGAGTGGGAGCCAACAAATTCGCCGAGAGGCAGCCGATTGGCACGGCTGCT
CAAAGCCAGGACAAGGACTACAAGGAACCACCACCGGCGCCACTTTTCGAGCCGGGTGAGCTGTCCTCGTGGTCG
TTCTACAGAGCTGGCATAGCAGAGTTCATAGCCACTTTCTTGTTCCTTTATATTACAGTTTTGACAGTGATGGGA
GTTAGTAAGTCTGATTCCAAGTGCAAAACTGTTGGAATCCAAGGTATTGCTTGGGCATTTGGTGGCATGATCTTT
GCCCTTGTTTACTGCACTGCAGGAATTTCAGGCAAGTTCTTTTCTATATATATAATTTGCAAATTTTGCTTGTAT
TTGCTATGATCTTCAACAGTAACGATCCGTCTTAAAGCTCAAAGAGGGAGTCGTCAGATTTGAAGATTGGTTTGT
GGGGTAGATATGAAAATACTAACCAACATATATATTGGATTATGGTCTCTGTCTCTGCAGTTTAGTCTTTGATAG
TATATGGAATTTAGGTTTTGCCATTAAAATTAGAAATTACCTAATTCAAAATTGAGTTGATTTTTCATGAATTTC
AGTTATAAGGTTTTAATTCCATTTGATATTAACATGGCAATTCAGAAAACCAAATCGTTTCAGCAATCAAATTTA
TGATGAACAATTTTAACAGTACTACTAACCTATTTTCTTCAAAGAAAAGGGCAAAATTTTAATGAACTGCTTGTT
TTGCAGGGGGGCATATAAATCCGGCAGTGACTTTCGGGCTGTTTTTGGGGAGGAAATTGTCGTTGACCCGGGCTG
TGTTCTACATGGTGATGCAGTGTCTTGGAGCCATATGTGGTGCTGGTGTGGTTAAGGGATTCGGAAAGACCCTGT
ACATGACCAAGGGTGGTGGTGCTAATGTAGTAGCCCACGGTTACACCAAGGGCGATGGCCTAGGTGCTGAAATAA
TCGGAACTTTTGTGCTCGTTTACACTGTCTTCTCTGCTACTGATGCCAAACGTAGCGCCAGAGACTCGCACGTTC
CTGTAAGCACTCCTCTTGTCTTTGTTGATTGAGCTTGAACAAGAATTTGCTTGTGTATCTGTAATCAAATTGTCT
TTTCGTTTTGTAAGAACTGATGAATCAAAAAATGTCATTGTTTATCAAGAAAACGTTTGATTAATTGGATTCCAT
ATGCTGTAATTCAATGGTTTTCAGATATTGGCCCCCTTGCCTATTGGGTTTGCTGTGTTCTTGGTGCACTTGGCA
ACCATCCCTATTACCGGTACAGGTATTAACCCCGCCAGGAGTCTTGGTGCAGCAATCATCTACAACAAAGACAAG
GCATGGGATGATCACGTAAGCTAAAAATTCAACTGTCTGAATTCAACTATTGAAATATTTGAGTTAAGGGCATAC
CTAGACAATTATATCCTAACAAATTTTTACGTTGTTCACTTATCACAATCCTCCTTTTATCTATTTTGGGACTAC
GACGAAATTTTTTAATAATCTGGCAGATGAGATGATATGATTGTCTAAAGTTTGTTCTCACTCTCATCTTGCTCT

GATATGATAATATGCAGTGGATATTCTGGGTTGGACCGTTCATTGGGGCAGCACTTGCAGCTCTATACCATGTGG
TGGTGATCCGAGCCATCCCATTCAAAAAA**TAA**

>*Oleur*PIP1;1

ATGGAGAGCAAGGAGGAGGATGTACGAGTGGGAGCCAACAAATTCGCCGAGAGGCAGCCGATTGGCACGGCTGCT
CAAAGCCAGGACAAGGACTACAAGGAACCACCACCGGCGCCACTTTTCGAGCCGGGTGAGCTGTCCTCGTGGTCG
TTCTACAGAGCTGGCATAGCAGAGTTCATAGCCACTTTCTTGTTCCTTTATATTACAGTTTTGACAGTGATGGGA
GTTAGTAAGTCTGATTCCAAGTGCAAAACTGTTGGAATCCAAGGTATTGCTTGGGCATTTGGTGGCATGATCTTT
GCCCTTGTTTACTGCACTGCAGGAATTTCAGGGGGGCATATAAATCCGGCAGTGACTTTCGGGCTGTTTTTGGGG
AGGAAATTGTCGTTGACCCGGGCTGTGTTCTACATGGTGATGCAGTGTCTTGGAGCCATATGTGGTGCTGGTGTG
GTTAAGGGATTCGGAAAGACCCTGTACATGACCAAGGGTGGTGGTGCTAATGTAGTAGCCCACGGTTACACCAAG
GGCGATGGCCTAGGTGCTGAAATAATCGGAACTTTTGTGCTCGTTTACACTGTCTTCTCTGCTACTGATGCCAAA
CGTAGCGCCAGAGACTCGCACGTTCCTATATTGGCCCCCTTGCCTATTGGGTTTGCTGTGTTCTTGGTGCACTTG
GCAACCATCCCTATTACCGGTACAGGTATTAACCCCGCCAGGAGTCTTGGTGCAGCAATCATCTACAACAAAGAC
AAGGCATGGGATGATCACTGGATATTCTGGGTTGGACCGTTCATTGGGGCAGCACTTGCAGCTCTATACCATGTG
GTGGTGATCCGAGCCATCCCATTCAAAAAA**TAA**

>*Oleur*PIP1;1

MESKEEDVRVGANKFAERQPIGTAAQSQDKDYKEPPPAPLFEPGELSSWSFYRAGIAEFIATFLFLYITVLTVMG
VSKSDSKCKTVGIQGIAWAFGGMIFALVYCTAGISGGHINPAVTFGLFLGRKLSLTRAVFYMVMQCLGAICGAGV
VKGFGKTLYMTKGGGANVVAHGYTKGDGLGAEIIGTFVLVYTVFSATDAKRSARDSHVPILAPLPIGFAVFLVHL
ATIPITGTGINPARSLGAAIIYNKDKAWDDHWIFWVGPFIGAALAALYHVVVIRAIPFKK*

*Oleur*PIP1;2a Oleur061Scf0176g00018.1

>Oleur061Scf0176:63114..64769 (+ strand) class=gene length=1656

>*Oleur*PIP1;2a

ATGGAGAGCAAAGAGGAAGATGTACGAGTGGGAGCCAACAAATTCAACGAGAGGCAGCCGATTGGCACGGCGGCG
CAATCCCAAGACAAGGACTACAAGGAGCCACCACCGGCGCCGCTTTTCGAGCCCGGCGAGCTGTCCTCATGGTCG
TTCTACAGAGCTGGCATTGCCGAGTTCATAGCCACATTCCTGTTCCTGTATATTACAATTTTGACAGTGATGGGA
GTTAGCAAGTCTGATTCTAAGTGCTCTACTGTTGGAATCCAAGGTATTGCTTGGGCATTCGGTGGCATGATCTTT
GCCCTTGTTTACTGCACCGCTGGAATTTCAGGCAAGTTCTTTACCTATAAATGGTTCGCATATTTGGCTTCTATA
TACCTTGATCTTTAACCTTAATATCTCATACGAGTAATCGATCTGTCTAAAACTCTAAAATTGAGTTAGATTTCA
GGATTGGCGTTCTCAGTTGGGTTGCTCCGTCTTGATGATGAGGTTGAAGTTTAACATGGAATCGTCTGTGTCTCT
GTAGTTAGTCTTTGATAGTAGCTTTGGGAATCAAAATAAGAAATTACCTAATTCAAAATTGAGTTGATTTTATAT
TAATTTCTGTTCTGAATAACAATTATAACAATATTAACTTATTTCTTCAAAGAAAATGGCATAATTATTACTGG
AAAAGAAAGCAAGTTGTATGACCAGTAACTTATTGACGAGTTGTTTGCTGTGCAGGGGGGCATATAAACCCGGCA
GTGACATTTGGGCTGTTTTTGGCCAGGAAATTGTCATTGACTCGGGCTATATTCTACATGGTGATGCAGTGCCTA
GGAGCCATATGTGGTGCTGGTGTGGTTAAGGGGTTCGGAAAGACCCTGTACATGTCCAAGGGTGGTGGTGCTAAT
GTTGTAGCTCACGGTTACACCAAGGGTAGTGGCCTAGGTGCTGAAATTATAGGTACCTTCGTTCTCGTTTACACC
GTTTTCTCTGCTACTGATGCCAAACGTAGCGCCAGAGACTCTCACGTTCCAGTAAGCACTCCTCTTTTCTTTTTT
GGCTAGTTTCTCTTGAAATTTTTCATTTGTACAGGTTGTAGATATTGATCAATCAAAGCACAACATTATTGACTC
AAAAAAGTTTGATTAATTGGACTCCCTAATTCTGTAATTCTATGGTTTTCAGATATTAGCCCCCTTGCCTATTGG
GTTTGCCGTGTTCTTGGTGCACTTGGCGACCATCCCTATTACAGGTACTGGTATTAACCCCGCCAGGAGTCTTGG
TGCAGCAATCATCTACAACAAAGACAAGGCATGGGATGATCACGTAAGCTAAAATTCAACTGTATCTGAATTCAG
TATAATCAGGATTCTACTATTGAAATATTCGAGTTAAGGACTCACTCAGACAATTATATCCTAACTTACACTACA
AGTCGTGTTAAATTTGGCGTGTATAAGGTGAATGGTAAAATGTGACATATTTTTACGTTGTTAACTTACCACAAC
CCTCCTATTTTAGGATGATAAGGTTGGATAAAGTTAGTTATCTTTTTTCCTGATAATGTGCAGTGGATATTCTGG
GTTGGACCATTCATTGGGGCAGCACTTGCAGCTCTCTACCATGTGGTTGTGATAAGAGCCATCCCATTCAAGAAT
AAG**TGA**

>*Oleur*PIP1;2a

ATGGAGAGCAAAGAGGAAGATGTACGAGTGGGAGCCAACAAATTCAACGAGAGGCAGCCGATTGGCACGGCGGCG
CAATCCCAAGACAAGGACTACAAGGAGCCACCACCGGCGCCGCTTTTCGAGCCCGGCGAGCTGTCCTCATGGTCG
TTCTACAGAGCTGGCATTGCCGAGTTCATAGCCACATTCCTGTTCCTGTATATTACAATTTTGACAGTGATGGGA
GTTAGCAAGTCTGATTCTAAGTGCTCTACTGTTGGAATCCAAGGTATTGCTTGGGCATTCGGTGGCATGATCTTT
GCCCTTGTTTACTGCACCGCTGGAATTTCAGGGGGGCATATAAACCCGGCAGTGACATTTGGGCTGTTTTTGGCC
AGGAAATTGTCATTGACTCGGGCTATATTCTACATGGTGATGCAGTGCCTAGGAGCCATATGTGGTGCTGGTGTG
GTTAAGGGGTTCGGAAAGACCCTGTACATGTCCAAGGGTGGTGGTGCTAATGTTGTAGCTCACGGTTACACCAAG
GGTAGTGGCCTAGGTGCTGAAATTATAGGTACCTTCGTTCTCGTTTACACCGTTTTCTCTGCTACTGATGCCAAA
CGTAGCGCCAGAGACTCTCACGTTCCAATATTAGCCCCCTTGCCTATTGGGTTTGCCGTGTTCTTGGTGCACTTG
GCGACCATCCCTATTACAGGTACTGGTATTAACCCCGCCAGGAGTCTTGGTGCAGCAATCATCTACAACAAAGAC
AAGGCATGGGATGATCACTGGATATTCTGGGTTGGACCATTCATTGGGGCAGCACTTGCAGCTCTCTACCATGTG
GTTGTGATAAGAGCCATCCCATTCAAGAATAAG**TGA**

>*Oleur*PIP1;2a
MESKEEDVRVGANKFNERQPIGTAAQSQDKDYKEPPPAPLFEPGELSSWSFYRAGIAEFIATFLFLYITILTVMG
VSKSDSKCSTVGIQGIAWAFGGMIFALVYCTAGISGGHINPAVTFGLFLARKLSLTRAIFYMVMQCLGAICGAGV
VKGFGKTLYMSKGGGANVVAHGYTKGSGLGAEIIGTFVLVYTVFSATDAKRSARDSHVPILAPLPIGFAVFLVHL
ATIPITGTGINPARSLGAAIIYNKDKAWDDHWIFWVGPFIGAALAALYHVVVIRAIPFKNK*

*Oleur*PIP1;2b Oleur061Scf0994g03003.1

>Oleur061Scf0994 :369686..371341 (+ strand) class=gene length=1656

>*Oleur*PIP1;2b
ATGGAGAGCAAAGAGGAAGATGTACGAGTGGGAGCCAACAAATTCAACGAGAGGCAGCCGATTGGCACGGCGGCG
CAATCCCAAGACAAGGACTACAAGGAGCCACCACCGGCACCGCTTTTCGAGCCCGGTGAGCTGTCCTCATGGTCG
TTCTACAGAGCTGGCATTGCCGAGTTCATAGCCACATTCCTGTTCCTGTATATTACAATTTTGACAGTGATGGGA
GTTAGCAAGTCTGATTCTAAGTGCTCTACTGTTGGAATCCAAGGTATTGCTTGGGCATTCGGTGGCATGATCTTT
GCCCTTGTTTACTGCACCGCTGGAATTTCAGGCAAGTTCTTTACCTATAAATGGTTCGCATATTTGGCTTCTATA
TACCTTGATCTTTAACCTTAATATCTCATACGAGTAATCGATCTGTCTAAAACTCTAAAATTGAGTTAGATTTCA
GGATTGGCGTTCTCAGTTGGGTTGCTCCGTCTTGATGATGAGGTTGAAGTTTAACATGGAATCGTCTGTGTCTCT
GTAGTTAGTCTTTGATAGTAGCTTTGGGAATCAAAATAAGAAATTACCTAATTCAAAATTGAGTTGATTTTATAT
TAATTTCTGTTCTGATGAACAATTATAACAATATTAACTTAGTTTCTTCAAAGAAAATGGTATAATTATTACTGG
AAAAGAAAGCAAGTTGTATGACCAGTAACTTATTGACGAGCTGTTTGCTGTGCAGGGGGGCATATAAACCCGGCA
GTGACATTTGGGCTGTTTTTGGCCAGGAAATTGTCATTGACTCGGGCTATATTCTACATGGTGATGCAGTGTCTA
GGAGCCATATGTGGTGCTGGTGTGGTTAAGGGGTTCGGAAAGACCCTGTACATGTCCAAGGGTGGTGGTGCTAAT
GTTGTAGCTCACGGTTACACCAAGGGTAGTGGCCTAGGTGCTGAAATTATAGGTACCTTCGTTCTCGTTTACACC
GTTTTCTCTGCTACTGATGCCAAACGTAGCGCCAGAGACTCTCACGTTCCTGTAAGCACTCCTCTTTTATTTTTC
GGCGCGTTTCTCTTGAAATTTTTCATTTGTACATGTTGTAGATATTGATCAATCAAAACACAACATTATTGACAC
AAAAAAGTTTGATTAATTGGAATCCCTAATTCTGTAATTCTATGGTTTTCAGATATTAGCCCCCTTGCCTATTGG
GTTTGCCGTGTTCTTGGTGCACTTGGCGACCATCCCTATTACAGGTACTGGTATTAACCCCGCCAGGAGTCTTGG
TGCAGCAATCATCTACAACAAAGACAAGGCATGGGATGATCACGTAAGCTAAAATTCAACTGTATCTGAATTCAG
TATAATCAGGATTCTACTATTGAATTATTCGAGTTAAGGACTCACTCAGACAATTATATCCTAACTTACACTACA
AGTCGTGTTAAATTTGGCGTGTATAAGGTGAATGGTAAAATGTGACATATTTTTACGTTGTTAACTTACCACCAC
CCTCCTATTTTGGGATGATAAGGTTGGATAAAGTTAGTTATCTTTTTTCCTGATAATATGCAGTGGATATTCTGG
GTTGGACCATTCATTGGGGCAGCACTTGCAGCTCTCTACCATGTGGTCGTGATAAGAGCCATCCCATTCAAGAAT
AAG**TGA**
>*Oleur*PIP1;2b
ATGGAGAGCAAAGAGGAAGATGTACGAGTGGGAGCCAACAAATTCAACGAGAGGCAGCCGATTGGCACGGCGGCG
CAATCCCAAGACAAGGACTACAAGGAGCCACCACCGGCACCGCTTTTCGAGCCCGGTGAGCTGTCCTCATGGTCG
TTCTACAGAGCTGGCATTGCCGAGTTCATAGCCACATTCCTGTTCCTGTATATTACAATTTTGACAGTGATGGGA
GTTAGCAAGTCTGATTCTAAGTGCTCTACTGTTGGAATCCAAGGTATTGCTTGGGCATTCGGTGGCATGATCTTT
GCCCTTGTTTACTGCACCGCTGGAATTTCAGGGGGGCATATAAACCCGGCAGTGACATTTGGGCTGTTTTTGGCC
AGGAAATTGTCATTGACTCGGGCTATATTCTACATGGTGATGCAGTGTCTAGGAGCCATATGTGGTGCTGGTGTG
GTTAAGGGGTTCGGAAAGACCCTGTACATGTCCAAGGGTGGTGGTGCTAATGTTGTAGCTCACGGTTACACCAAG
GGTAGTGGCCTAGGTGCTGAAATTATAGGTACCTTCGTTCTCGTTTACACCGTTTTCTCTGCTACTGATGCCAAA
CGTAGCGCCAGAGACTCTCACGTTCCTATATTAGCCCCCTTGCCTATTGGGTTTGCCGTGTTCTTGGTGCACTTG
GCGACCATCCCTATTACAGGTACTGGTATTAACCCCGCCAGGAGTCTTGGTGCAGCAATCATCTACAACAAAGAC
AAGGCATGGGATGATCACTGGATATTCTGGGTTGGACCATTCATTGGGGCAGCACTTGCAGCTCTCTACCATGTG
GTCGTGATAAGAGCCATCCCATTCAAGAATAAG**TGA**
>*Oleur*PIP1;2b
MESKEEDVRVGANKFNERQPIGTAAQSQDKDYKEPPPAPLFEPGELSSWSFYRAGIAEFIATFLFLYITILTVMG
VSKSDSKCSTVGIQGIAWAFGGMIFALVYCTAGISGGHINPAVTFGLFLARKLSLTRAIFYMVMQCLGAICGAGV
VKGFGKTLYMSKGGGANVVAHGYTKGSGLGAEIIGTFVLVYTVFSATDAKRSARDSHVPILAPLPIGFAVFLVHL
ATIPITGTGINPARSLGAAIIYNKDKAWDDHWIFWVGPFIGAALAALYHVVVIRAIPFKNK*

*Oleur*PIP1;3a Oleur061Scf1119g10041.1

>Oleur061Scf1119:1019235..1022315 (- strand) class=gene length=3081

>*Oleur*PIP1;3a

ATGGCAGAAAGCAAGGAAGAAGATGTTAAACTTGGAGCCAACAAGTTCACAGAGAAGCAACCATTAGGCACAGCG
GCTCAAACAGACAAGGACTATAAGGAGCCACCACCAGCACCTTTATTTGAGCCTGGAGAATTGACATCATGGTCC
TTTTACAGGGCTGGAATTGCAGAATTCATAGCCACTTTCTTGTTCTTGTACATATCTATTTTGACTGTTATGGGT
GTTTTCAGTGCAAAGAACAAGTGTGCCTCTGTGGGCGTCCAGGGAATTGCTTGGGCCTTTGGGGGTATGATCTTT
GCACTTGTCTACTGTACTGCTGGTATCTCAGGTCAGTCCCTAAAACCCCTTTCCTATCTTTACTTTTAATTTACC
CTTTTTTTTTTTCAAATTGCTTAATTTTTCTTTTTCTTTTTTATTTTTGGACTTCTCTCTGTATATCTTTTTTT
TTAGTCCTTCTTTTTACGATCTTTGACTCAAAAAAGTTCTTCAATCGGTTTGTGGGTCTTTAGAGATTCTTGAGG
AAATTTATTCAGTTGAGTAGTAGTGATTTGTGTGGTTCCTTCTTTGTATTGGAAAATTTTGTGGCTTTTTATTGT
TCTGTTTATCTGTATAAGTGCTTAGTTTAACCACGGGCTAGCTACATAGATGATATTTGGAACTAAAGATTGTAT
CTTTGTGATTTTTTGAAATTTTTTTGACTTTTTTCAGTGTAAGCTGTTTGGCTAAGCTTAAAATGTTTGCTTATA
AGCAAATAAGTATTAAAATAATTACTAGCTTAGTAAGGTAAAGTGTATTAGGTATATACACTAACGTTTTTCTTC
TACTTTGATGGTAAGGTAAAAATTGTTATGACCATGTTAACAAGGTTGATCTTATTAAGAGGAATGACTCGATAT
CTATCTAAGTTGGTCAATATTTCGATATTTAATCAATACGTGATTTATTCTCTTAACATGCTCCCGCAAAATAAT
GGCTCTTTTTTAGCAGACCAATTTTGAAATTCGTGTTCTTATTTTTGAGACTTAATCGGATCGAATAGCTCGTTG
GAGTTACTGGGCTCTGATATCGTGTTAACAACCTTACTAGGAGTGGCCCAAAATCTATTTAAGTTGGTCAATATT
TCGGTATTTAACCGACGTGTGATTTATTCCCTTAACAAAAATTGTCAAAATATGTAAAAAATTTTATTTTTATAG
CTTAGCTAGACATAAGTGAGGGAAAAGTAAGATATTGAAACATTTTTGAAACGGAAATTTACTTATGAGCAACTT
TTAAACTACTTTGATGAAGCATATTTAAATTTTTCTTGTTTGCATGTTAAAACAGGAGGGCACATCAACCCAGCT
GTGACATTTGGCTTGTTCTTGGCAAGGAAACTTTCTTTGATCAGGGCTTTATTTTACATGGTGATGCAGTGCCTG
GGTGCCATATGTGGTGCTGGTGTTGTTAAGGGGTTCATGAAGGGTCAATACGAGAGACTTCAAGGCGGTGCTAAC
ATTGTGCAGCATGGCTACACCAAAGGCGATGCCCTTGGTGCGGAGATAGTTGGCACCTTTATTCTCGTCTACACT
GTCTTCTCGGCTACTGATGCCAAGAGAAATGCTAGAGACTCACACGTCCCGGTATGATTACCCATTCTTCGCTAA
TTACTTCGACTAACATCACTATTATTGACTAACATCACTATTATTGCTCGCTTAAAATTAATTAATTAATTAATT
AATTTATTATTATTATTTTTTTTTTTTTTTTTTTTTTTTTTTAATTATTATTATTATTTTTTTATGAAATTGCTGCA
AAAGCTTAAGTTGAGAGGGCATATTGACTGATTTAAATTTAGAAGGCTGGTATATGCATGATTTTTGCGAAGGCT
TGGCGATGAGACTTGAACTTATACCCCTGCAATTTAACATTGAGTGTTTTGACAAATTTCATTATTTAGTGCGTT
GTGGTTGCTAGGGGATCTATTATTGGTATGATGAATGTGCCAAAAACTTTTCGAAAATATAAGATAATACCGAAG
CTGATCCTGTGAAATTTCTTGATTTTAAAACTTGGGATTTCATTATAAAATGTCAATCTTGTTAAGAAGAATAGT
CAAGCCCTTAACGTGCTCATTTTTGGAGTCTTAATTGGATCAAATGGCTCGTTGGAGCTTAATAGGTTTATACCA
TATTAAAACTTGGGATTTCATTTTAAAAGGTTTATCTTGTTAAGAGATGCAGTTCTAACCATTATGACGTCCATG
TTTTATGTGGAATTTTATCCCTTGACATACTGGTTCTTAAATATGTCTTCGTGATTGTTGCAGCTTTTGGCTCCA
CTCCCCATTGGATTTGCAGTATTTTTGGTTCATTTGGCCACCATCCCCATTACTGGAACAGGTATCAACCCTGCT
AGGAGTCTCGGAGCTGCCATAATTTACAATAGAGACCACGCATGGGACGACCAGGTAAGATTCTTGCTATACATT
ACATATAACACCTGTATCTATTCCAATGTTTTTTCTTGACTAAATTACGCGATTTCTTTATTTACAGTGGATCTT
CTGGGTTGGTCCCTTCATTGGAGCTGCTCTTGCTGCAATTTACCACCAGATTGTCATCAGAGCCATTCCTTTCAA
GAGCAGGGCT**TAA**

>*Oleur*PIP1;3a

ATGGCAGAAAGCAAGGAAGAAGATGTTAAACTTGGAGCCAACAAGTTCACAGAGAAGCAACCATTAGGCACAGCG
GCTCAAACAGACAAGGACTATAAGGAGCCACCACCAGCACCTTTATTTGAGCCTGGAGAATTGACATCATGGTCC
TTTTACAGGGCTGGAATTGCAGAATTCATAGCCACTTTCTTGTTCTTGTACATATCTATTTTGACTGTTATGGGT
GTTTTCAGTGCAAAGAACAAGTGTGCCTCTGTGGGCGTCCAGGGAATTGCTTGGGCCTTTGGGGGTATGATCTTT
GCACTTGTCTACTGTACTGCTGGTATCTCAGGAGGGCACATCAACCCAGCTGTGACATTTGGCTTGTTCTTGGCA
AGGAAACTTTCTTTGATCAGGGCTTTATTTTACATGGTGATGCAGTGCCTGGGTGCCATATGTGGTGCTGGTGTT
GTTAAGGGGTTCATGAAGGGTCAATACGAGAGACTTCAAGGCGGTGCTAACATTGTGCAGCATGGCTACACCAAA
GGCGATGCCCTTGGTGCGGAGATAGTTGGCACCTTTATTCTCGTCTACACTGTCTTCTCGGCTACTGATGCCAAG
AGAAATGCTAGAGACTCACACGTCCCGTGCGTTGTGGTTGCTAGGGGATCTATTATTGTATTTTTGGTTCATTTG
GCCACCATCCCCATTACTGGAACAGGTATCAACCCTGCTAGGAGTCTCGGAGCTGCCATAATTTACAATAGAGAC
CACGCATGGGACGACCAGTGGATCTTCTGGGTTGGTCCCTTCATTGGAGCTGCTCTTGCTGCAATTTACCACCAG
ATTGTCATCAGAGCCATTCCTTTCAAGAGCAGGGCT**TAA**

>*Oleur*PIP1;3a

MAESKEEDVKLGANKFTEKQPLGTAAQTDKDYKEPPPAPLFEPGELTSWSFYRAGIAEFIATFLFLYISILTVMG
VFSAKNKCASVGVQGIAWAFGGMIFALVYCTAGISGGHINPAVTFGLFLARKLSLIRALFYMVMQCLGAICGAGV
VKGFMKGQYERLQGGANIVQHGYTKGDALGAEIVGTFILVYTVFSATDAKRNARDSHVPCVVVARGSIIVFLVHL
ATIPITGTGINPARSLGAAIIYNRDHAWDDQWIFWVGPFIGAALAAIYHQIVIRAIPFKSRA*

***Oleur*PIP1;3b Oleur061Scf3031g04003.1**

>Oleur061Scf3031:499618..502336 (+ strand) class=gene length=2719

>*Oleur*PIP1;3b

ATGGCAGAAAGCAAGGAAGAAGATGTTAAACTCGGAGCCAACAAGTTCACAGAGAGGCAGCCATTAGGCACAGCG
GCTCAAACAGACAAGGACTATAAGGAGCCACCACCAGCACCTTTATTTGAGCCTGGAGAATTGACATCATGGTCC

TTTTACAGGGCTGGAATTGCAGAATTCATAGCCACTTTCTTGTTCTTGTACATATCTATTTTGACTGTTATGGGT
GTTTTAAGTGCAAAGAATAAGTGTGCCTCTGTGGGCGTCCAGGGAATTGCTTGGGCCTTTGGGGGTATGATCTTT
GCACTTGTCTATTGTACTGCTGGTATCTCAGGTCAGTCTCTAAACCCCCTTTCCTCTCTTTACTTTTAAGTAACC
TTTTTTTTTTTTTAAAATTTCAAATTACTTAATTTTTCTTTTTCTTTTTTTATTTTTGGACTTCTCTCTGTATAT
CTATCTTTTTAGTCCTGTTTTTTTACGATCTTTGACTCAAAAAAGTTCTTCAATCGGTTTGTGGGGTTTTAGAGA
TTTTTGAGGAAATTTATTCAGTTGAGTAGTGATTTGTGTGGTTCCTTCTTTGTATTGGAAATTTTCGTCGCTTTT
TATTGTTCTGTTTATCTGTATAAGTGCTTAGTTTAACCACGGGCTAGTTACATAGATGATATTTGGAACCAAAGA
TTGTATCTTTGTGATTTCTTGAAATTTTTTTGACTTATTTCCGTGTAAGCTGTTTGGCTAAGCTTAAACTGTTTG
CTTATAAGCAAATAAGTATTAAAATAACTACTATTTTGATGGTTAACGTTGTATTAGTAGCTTAATAAGCTAACG
TGTATATATATATGTATATATAAAATAACATTTTTCTTCTACTTTGATGGTAAGGTAAAAATTGTCAAAATATGT
AATTTTTTTTTTTTTAATAGCTTAGCTAGACATAAGTGAGGGAAAAGTAAGATACTGAAACATTTTTGAAACAAAA
ATTTACTTATGAGCAACTTTTAAACTACTTTGATGAAGCATATTTAAATTTTTCTTGTTTGCATGTTAAAACAGG
AGGGCACATCAACCCAGCTGTGACCTTTGGCTTGTTCTTGGCAAGGAAACTTTCTTTGATCAGGGCTTTATTTTA
CATGGTGATGCAGTGCCTGGGTGCCATATGTGGTGCTGGTGTTGTTAAGGGGTTCATGGAGGGTCAATACGAGAG
ACTTCAAGGCGGTGCTAACATTGTGCAGCATGGCTACACCAAAGGCGATGCCCTTGGTGCGGAGATAGTTGGCAC
CTTTATTCTCGTCTACACTGTCTTCTCGGCTACTGATGCCAAGAGAAATGCCAGAGACTCACACGTCCCAGTATG
ATTGATTACCCATTCTTCGCTAAATTACTTCCGACTAACATCACCATTATTGCTCGCTTAATATTATTATTATTA
ATTTTTTTTTTTGGAAATTTCGGCAAAAGCTTAAGTTGAGAGGGCGTATTGATTGATTTAAATTTAGAAGACTG
GTATATGCACAATTTTTGCAAAGGTGTTGCGATGAGACTTGAACTTATATCCTCCAATTTAACATTGAGTGTTTT
GACAAATTTGATTATTTAGCGCGTTGTGGTTGCTAGGATCTATTATTAGTATATTTGGAATGTAGGAATGAATGT
GCCAAAATCTTTTTGAAAATATTTTTCAAAAATCCTTGTACAGTTGTTGGTACCCCTTTTGGAGTAAGGATAATA
CATACAGGAGCTGATCCTGTGAAATTTCTCGATTTCAAAATTTGGGATTTCATTTTAAAATGTCAATCTTGTTAA
GAGAAATAGTCAAGCCCTTTTATGTTAGTTTATGTTTTCATGTGGGATTTTATCCCTTGATGTGCTCATTTTTCG
AGTCTTAATCGGATCAAATTGCTCGTCGGAGGTTAACGGGTTCTGATACCGTTTTATGTGGAATTTTATCCAAGA
CATAGTGGTTCTTAAATATATCTTCGTGATTGTTGCAGCTTTTGGCTCCACTCCCCATTGGATTTGCAGTATTTT
TGGTTCATTTGGCCACCATCCCCATCACTGGAACAGGTATCAACCCTGCTAGGAGTCTCGGAGCTGCTATAATTT
ACAATAGAGACCACGCATGGGACGACCAGGCAAGATTCTTGCTA**TAA**

>*Oleur*PIP1;3b

ATGGCAGAAAGCAAGGAAGAAGATGTTAAACTCGGAGCCAACAAGTTCACAGAGAGGCAGCCATTAGGCACAGCG
GCTCAAACAGACAAGGACTATAAGGAGCCACCACCAGCACCTTTATTTGAGCCTGGAGAATTGACATCATGGTCC
TTTTACAGGGCTGGAATTGCAGAATTCATAGCCACTTTCTTGTTCTTGTACATATCTATTTTGACTGTTATGGGT
GTTTTAAGTGCAAAGAATAAGTGTGCCTCTGTGGGCGTCCAGGGAATTGCTTGGGCCTTTGGGGGTATGATCTTT
GCACTTGTCTATTGTACTGCTGGTATCTCAGGAGGGCACATCAACCCAGCTGTGACCTTTGGCTTGTTCTTGGCA
AGGAAACTTTCTTTGATCAGGGCTTTATTTTACATGGTGATGCAGTGCCTGGGTGCCATATGTGGTGCTGGTGTT
GTTAAGGGGTTCATGGAGGGTCAATACGAGAGACTTCAAGGCGGTGCTAACATTGTGCAGCATGGCTACACCAAA
GGCGATGCCCTTGGTGCGGAGATAGTTGGCACCTTTATTCTCGTCTACACTGTCTTCTCGGCTACTGATGCCAAG
AGAAATGCCAGAGACTCACACGTCCCACTTTTGGCTCCACTCCCCATTGGATTTGCAGTATTTTTGGTTCATTTG
GCCACCATCCCCATCACTGGAACAGGTATCAACCCTGCTAGGAGTCTCGGAGCTGCTATAATTTACAATAGAGAC
CACGCATGGGACGACCAGGCAAGATTCTTGCTA**TAA**

>*Oleur*PIP1;3b

MAESKEEDVKLGANKFTERQPLGTAAQTDKDYKEPPPAPLFEPGELTSWSFYRAGIAEFIATFLFLYISILTVMG
VLSAKNKCASVGVQGIAWAFGGMIFALVYCTAGISGGHINPAVTFGLFLARKLSLIRALFYMVMQCLGAICGAGV
VKGFMEGQYERLQGGANIVQHGYTKGDALGAEIVGTFILVYTVFSATDAKRNARDSHVPLLAPLPIGFAVFLVHL
ATIPITGTGINPARSLGAAIIYNRDHAWDDQARFLL*

*Oleur*PIP1;4a Oleur061Scf0931g02045.1

>Oleur061Scf0931:329811..332344 (- strand) class=gene length=2534

>*Oleur*PIP1;4a

ATGGAGGGTAAAGAGGAGGATGTGAGGCTAGGAGCCAACAAGTTCACAGAGAGGCAACCATTGGGTACCTCAGCA
CAGAGCAAGGACTACAAGGAGCCACCTCCAGCTCCACTTTTTGAGCCTGGTGAACTCCAGTCATGGTCCTTTTGG
AGAGCTGGGATTGCTGAGTTTATGGCTACTTTCTTGTTTCTTTATATCACAATCTTGACTGTAATGGGTGTCAGC
AGGTCTGGCAGCAAGTGTGCTTCTGTGGGTATTCAGGGCATTGCTTGGGCCTTTGGTGGCATGATTTTTGCCCTT
GTCTACTGCACTGCTGGCATCTCAGGTCCTTCTCTCTCTCTCTCTCTCTCAATATCTCTGTGTTAGTGTGTTCTA
TATTCTGTTGCTTAAGTAAATTTCGAGTCTGTTGTGTTTGGTGTGCAAGATTTTAACTGCTAAACTAATTTTGAG
TCCTCATTTTCATGGGATCTTGGAAAATATGAAGTATGAAGCTTCTGTTAGTGTTCTATATTCTGTTCTCAATGA
AATATAGAGTCTAGTCTGTGTTTAGTATGGAGTATTGGATGGAAGTGAACTATATAATGAACAAGATTTTATCTT
CGAACATACCAAGCAATACCACTAGATCTGGGTCCTCATTTTCATGGGATCTTGGAAATATGTAAATTATGCAGC
CTCTGTTTGTGTTCGGTAATCTGTTGCTTAAGTAAATCTAGAGTTTTGACTAGTATAATGAGCAAGATTTTATTT
TCTATATCTAACTAGCCACAAATGTACTAGTTAGGATTTTCTTTCTTTACCCCTTTTTTATTTTTTGGTGGAATT
CATTGTTGTTTTTCAGGTTTTAAATCTTGAATGTGGTCTTGTAATGTGGTTCAAACCCTGCTCTTCTCTTCGAAA

TCTGCACCTTGTGTTAGCAGCTTATTAATTTAATGGGATGTAGGGTTTTGTGCTGACATTTCTTTATTGTGATAC
TGATTAATGCCCTCGTTTGAAAATTATTGAACTTTTTTTATGTACTGATATTGGAATTTTTTATGGGTGATTTCA
GGTGGACACATAAATCCTGCAGTAACATTTGGTCTGTTCCTAGCAAGAAAATTGTCTCTAACCAGAGCAATTTTT
TACATCGTGATGCAATGTCTCGGAGCAATCTGTGGTGCTGGTGTCGTAAAGGGCTTCCAGCCTTCTTTTTACAAG
GCTAATGGTGGTGGTGCTAATGTTGTTTCTCATGGTTACACCAAGGGCGATGGACTTGGTGCTGAGATTATCGGC
ACTTTTGTTCTTGTTTACACTGTCTTTTCTGCTACTGATGCCAAGAGAAATGCCAGAGACTCACATGTCCCTGTA
AGTATTTCTTTGCAATCTTATTTTTTCTTGCCTGTTTTGTACTTATAGCATTTTGTTTATTGGTATTATTTCCGC
TTTCTGAATGATTGCCCTTTCTTACGCACAAGGCATGTGCCTTATCTTTAATGTGTCTGAATGTCTCATGAATTT
GGTTATACTTTTCTACTTGAAAAAAGGATTGCAATGCTGGAAGTCCATATAGTCTTGGCATGGTTAGGACTTAGG
AGCAATATCAATTTCTGCGCACGTCTTTTTTGTGATTCAGGAAGATGGAAATGAATTTTGAGGTTTTTTCCGTAG
TGTATGATGCTTTACATGATATATTATGGAGCCTTACGATATGCGTAGCCTTTGATTTTTCTTCTAGTACATAGT
TGGGCTAGAAATCAGTAATTTATGCCAAAACTTGAATCTTAACGTGGGATGCCAAATTGATGACTCCTTTGATCT
TCCAAATTTCACAGATTTTGGCTCCACTCCCAATTGGGTTTGCCGTCTTTTTGGTTCACTTGGCAACCATCCCCA
TCACCGGAACTGGCATCAACCCAGCCAGAAGCCTTGGCGCTGCCATCATTTACAACAAAGATCATGCTTGGGATG
ATCACGTAAGTTTACTTCATTAACTTCCACAGACAATCTTCTTACCTGTTCATGACATTTTTGGCGTCCATACTT
AAACATTTTTGTCACGTGCCTTTCTTCAGTGGATCTTCTGGGTCGGACCTTTCATTGGAGCTGCCCTTGCTGCAC
TTTACCATCAGGTTATCATTCGAGCCATCCCATTTAAGAGTAGCAGC**TGA**

***>Oleur*PIP1;4a**

ATGGAGGGTAAAGAGGAGGATGTGAGGCTAGGAGCCAACAAGTTCACAGAGAGGCAACCATTGGGTACCTCAGCA
CAGAGCAAGGACTACAAGGAGCCACCTCCAGCTCCACTTTTTGAGCCTGGTGAACTCCAGTCATGGTCCTTTTGG
AGAGCTGGGATTGCTGAGTTTATGGCTACTTTCTTGTTTCTTTATATCACAATCTTGACTGTAATGGGTGTCAGC
AGGTCTGGCAGCAAGTGTGCTTCTGTGGGTATTCAGGGCATTGCTTGGGCCTTTGGTGGCATGATTTTTGCCCTT
GTCTACTGCACTGCTGGCATCTCAGGTGGACACATAAATCCTGCAGTAACATTTGGTCTGTTCCTAGCAAGAAAA
TTGTCTCTAACCAGAGCAATTTTTTACATCGTGATGCAATGTCTCGGAGCAATCTGTGGTGCTGGTGTCGTAAAG
GGCTTCCAGCCTTCTTTTTACAAGGCTAATGGTGGTGGTGCTAATGTTGTTTCTCATGGTTACACCAAGGGCGAT
GGACTTGGTGCTGAGATTATCGGCACTTTTGTTCTTGTTTACACTGTCTTTTCTGCTACTGATGCCAAGAGAAAT
GCCAGAGACTCACATGTCCCTATTTTGGCTCCACTCCCAATTGGGTTTGCCGTCTTTTTGGTTCACTTGGCAACC
ATCCCCATCACCGGAACTGGCATCAACCCAGCCAGAAGCCTTGGCGCTGCCATCATTTACAACAAAGATCATGCT
TGGGATGATCACTGGATCTTCTGGGTCGGACCTTTCATTGGAGCTGCCCTTGCTGCACTTTACCATCAGGTTATC
ATTCGAGCCATCCCATTTAAGAGTAGCAGC**TGA**

***>Oleur*PIP1;4a**

MEGKEEDVRLGANKFTERQPLGTSAQSKDYKEPPPAPLFEPGELQSWSFWRAGIAEFMATFLFLYITILTVMGVS
RSGSKCASVGIQGIAWAFGGMIFALVYCTAGISGGHINPAVTFGLFLARKLSLTRAIFYIVMQCLGAICGAGVVK
GFQPSFYKANGGGANVVSHGYTKGDGLGAEIIGTFVLVYTVFSATDAKRNARDSHVPILAPLPIGFAVFLVHLAT
IPITGTGINPARSLGAAIIYNKDHAWDDHWIFWVGPFIGAALAALYHQVIIRAIPFKSSS*

*Oleur*PIP1;4b Oleur061Scf0180g12067.1

>Oleur061Scf0180:1186896..1189701 (+ strand) class=gene length=2806

***>Oleur*PIP1;4b**

ATGGAGGGTAAAGAGGAGGATGTGAGACTAGGAGCCAACAAATTCACAGAGAGGCAACCATTGGGTACCTCAGCA
CAGAGCAAGGACTACAAGGAGCCACCTCCAGCTCCACTTTTTGAGCCTGGTGAACTCCAATCATGGTCCTTTTGG
AGAGCTGGGATTGCTGAGTTTATGGCTACTTTCTTGTTTCTTTATATCACAATCTTGACTGTAATGGGTGTCAGC
AGGTCTGGCAGCAAGTGTGCTTCTGTGGGTATTCAGGGCATTGCTTGGGCCTTTGGTGGCATGATTTTTGCCCTT
GTCTACTGCACTGCTGGCATCTCAGGTTTCTCTCTCTCTCAATATCTCTGTGTTAGTGTGTTCTATATTCTGTTG
CTTCAGTGAATTTCGAGTCTGTTGTGTTTGGTGAGCAAGATTTTAACTCCTAGACATGCCAAGAATGTACTACTA
AACTATCTTTAAGTCCTCATTTTCATGGGATCTTGGAAAATATGAAGCATGAAGCTTCTGTTAGTGTTCTATATT
CTGTTCTCAAGTAAATATAGAGTTTAGTCTGTGTTTAGTATGGAGTATTGGATGGAAGTGAACTGTATAATGAAC
AAGATTTTATCTTCGAACATAACAAGCAATACTACTAGATCTGGGTCCTCATTTTCATGGGATCTTGGAAAGATG
TAAATTATGCAGCTTCTGTTTGTGTTCGGTAATCTGTTGCTCAAGTAAATCTAGAGTTTTGACTAGTATAATGAG
CAAGATTTTATTTTCTATATCTAACTAGCCACAAATGTACTAGTTAAGGATTTTCTTTCTTTCTTTCCTTTCTTT
TTTTTTTCTTGGTGGAATTCATTGTTGTTTTTCAGGTTTTAAATCTTGAATGTGGTCTTGTAATGTAGTTCAAAC
CATGCTCTTCTCTTCAAAATCTGCACCGTGTGTTACCAGCTTATTAATTTAATGGGATGTAGGGTTTTGTGCTGA
CATTTCTTTATTGTGATAATGACTAATGCCCTTGTTTGAAAATTATTGAACTTTTTTATGTACTGATATTGGAAT
TTTTTATGGATGATTTCAGGTGGACACATAAATCCTGCAGTAACATTTGGTCTGTTTCTAGCAAGAAAATTGTCT
CTAACCAGAGCAATTTTTTACATCGTGATGCAATGTCTCGGAGCAATCTGTGGTGCTGGTGTCGTAAAGGGCTTC
CAGCCTTCTTTTTACAAGGCTAATGGTGGTGGTGCTAATGTTGTTTCCCATGGTTACACCAAGGGCGATGGGCTT
GGTGCTGAGATTATCGGCACTTTTGTTCTTGTTTACACTGTCTTTTCTGCTACTGATGCGAAGAGAAATGCCAGA
GACTCACATGTCCCTGTAAGTATTTCTTTGCAATCTTTTTTTTTCGTCTGTTTTGTACTCATAGCATTTTGTTTA
TTGGTATTATTTTCGCTTTCTGTATGATTGCCCTTTCTTACACACAAGGCATGTGCCTTATCTGTAATGTGTCTC
ATGAATTTGATTATACTTTTCTACTTGAAAAAAGGATTGCAATGCTGGAAGTCCATATAGTCTTGGCATGGTTAG

GACTTAGGAGCAATATCAATTTCTGCGCACGTCTTTTTTGTGATTCAGGAAGATGGAAATGAATTTCGAGGTTTT
TTCCGTAGTGTATGATGCTTTACATGATATATTATGGAGCCTTACGATATGCGTAGCCTTTGATTTTTCTTCTAG
TACATAGTTGGGCTAGAAATCAGTAATTTATGCTAAAACTTGAATCTTAACGTGGGATGCCAAATTGATGACTCC
TTTGATCTTCCAAATTTCACAGATTTTGGCTCCACTCCCAATTGGGTTTGCCGTCTTTTTGGTTCACTTGGCAAC
CATCCCCATCACCGGAACTGGCATCAACCCAGCCAGAAGCCTTGGCGCCGCCATCATTTACAACAAAGATCATGC
TTGGGATGATCACGTAAGTTTACTTCATTAACTTCCACAGACAATCTTCTTACCTGTTCATGACATTTTTGGCGT
CCATACTTAAACATTTTTGTCACATGTGCCTTTCTTCAGTGGATCTTCTGGGTCGGACCTTTCATTGGAGCTGCC
CTTGCTGCACTTTACCATCAGGTTATCATTCGAGCCGTCCCATTTAAGAGTAGCAGC**TGA**

>*Oleur*PIP1;4b
ATGGAGGGTAAAGAGGAGGATGTGAGACTAGGAGCCAACAAATTCACAGAGAGGCAACCATTGGGTACCTCAGCA
CAGAGCAAGGACTACAAGGAGCCACCTCCAGCTCCACTTTTTGAGCCTGGTGAACTCCAATCATGGTCCTTTTGG
AGAGCTGGGATTGCTGAGTTTATGGCTACTTTCTTGTTTCTTTATATCACAATCTTGACTGTAATGGGTGTCAGC
AGGTCTGGCAGCAAGTGTGCTTCTGTGGGTATTCAGGGCATTGCTTGGGCCTTTGGTGGCATGATTTTTGCCCTT
GTCTACTGCACTGCTGGCATCTCAGGTGGACACATAAATCCTGCAGTAACATTTGGTCTGTTTCTAGCAAGAAAA
TTGTCTCTAACCAGAGCAATTTTTTACATCGTGATGCAATGTCTCGGAGCAATCTGTGGTGCTGGTGTCGTAAAG
GGCTTCCAGCCTTCTTTTTACAAGGCTAATGGTGGTGGTGCTAATGTTGTTTCCCATGGTTACACCAAGGGCGAT
GGGCTTGGTGCTGAGATTATCGGCACTTTTGTTCTTGTTTACACTGTCTTTTCTGCTACTGATGCGAAGAGAAAT
GCCAGAGACTCACATGTCCCTATTTTGGCTCCACTCCCAATTGGGTTTGCCGTCTTTTTGGTTCACTTGGCAACC
ATCCCCATCACCGGAACTGGCATCAACCCAGCCAGAAGCCTTGGCGCCGCCATCATTTACAACAAAGATCATGCT
TGGGATGATCACTGGATCTTCTGGGTCGGACCTTTCATTGGAGCTGCCCTTGCTGCACTTTACCATCAGGTTATC
ATTCGAGCCGTCCCATTTAAGAGTAGCAGC**TGA**
>*Oleur*PIP1;4b
MEGKEEDVRLGANKFTERQPLGTSAQSKDYKEPPPAPLFEPGELQSWSFWRAGIAEFMATFLFLYITILTVMGVS
RSGSKCASVGIQGIAWAFGGMIFALVYCTAGISGGHINPAVTFGLFLARKLSLTRAIFYIVMQCLGAICGAGVVK
GFQPSFYKANGGGANVVSHGYTKGDGLGAEIIGTFVLVYTVFSATDAKRNARDSHVPILAPLPIGFAVFLVHLAT
IPITGTGINPARSLGAAIIYNKDHAWDDHWIFWVGPFIGAALAALYHQVIIRAVPFKSSS*

***Oleur*PIP1;5a Oleur061Scf2999g01010.1**

>Oleur061Scf2999:181146..184008 (- strand) class=gene length=2863

>*Oleur*PIP1;5a
ATGGCAGAAAACAGGGAAGAGGATGTTAGGCTTGGAGCCAACAAGTTTACTGAGAAGCAGCCTTTGGGTACAGCT
GCTCAAACAGACAAGGACTACAAGGAGCCACCTCCAGCCCCTTTGTTTGAGCCTGGGGAGATTAAATCATGGTCT
TTTTACAGGGCTGGTATTGCAGAGTTTGTGGCTACCTTCTTGTTCTTGTACATTACCATTTTGACTGTCATGGGG
GTTGGTAGATCAAATAGTAAGTGTGCCTCTGTGGGCATCCAGGGGATTGCTTGGTCCTTTGGTGGCATGATTTTT
GCACTTGTATACTGCACTGCTGGGATTTCAGGTTAGTTTATTTTTGTTTTTTTAATCTCACTTAATCTTGTTTTT
TCTTTGCATGTTTCTGTTTGTTTCTTTTACTGTGAGTCTTGGCAAAATTGTGTATTTCCTTTGTTTTTATGGGTT
TCTGGGGTTTCGTTTTGCCAGATTGATTATGAATTATTGTTACTGGATCTTGTTAAATAAGTTTTTAATTGATCC
ATTTAGATATTATTTGCGATAAAAGCTAGGATCTTGGTGATTTTTTCAGGAAACTCCAGTTTTAGATTATATGCT
ATGGCTTAGGGTACCCGCACCCTAACCCCTCTTAGACCATTGTCTATTATATGTAGCTTCAAAACTGATTTTCTG
TATTGGCCGTTGAACAGGAGGGCACATCAACCCTGCTGTGACCTTCGGTTTGTTCTTGGCAAGGAAGCTTTCCTT
GACCAGAGCTATCTTCTACATAGTGATGCAGTGCCTTGGTGCAATCTGTGGTGCTGGTGTTGTCAAGGGATTCAT
GGAGGGTCCATACCAGAGGCTACAAGGTGGGGCCAACGTTGTGCAGCATGGCTACACCAAGGGTGATGGCCTTGG
TGCTGAGATTGTTGGCACTTTTATTCTTGTTTACACTTCTTCTCTGCCACTGATGCCAAGAGAAACGCCAGAGAC
TCGCATGTTCCAGTAGGTCTTCCTTTGTCGGTTATGCACTGATATTAACTTTTGGTTGCTTAAACTTGTTTGGAA
AGGCTGGATTTGGTGCCTTGATTTGTTTTTTGTTACTTCTAATTAAGCGTTCATACCCAAGAAAGGAAGAATCGA
TAATTTTATTATGTTTTGTGTAGCTGGAATGCTACATTTCTCTGATTTCATTTCGCTAATATTTGGTTTATATTT
ATAAACTGTTTAGAAACGCTCAATTCACTTTATGTCCACACATTTCTAAGGACCCACAACCACAAGTACTGCCCA
CTCCAACGCGACTCATTTCTACCTATTTATAATTTAAACCATCTTATAAACAACTTTCCAAACATGCTTTTAGAT
GTTGAGCAATTTTGCTTATTTAGCCTCAGTTCATTCAATGCAATCTTGACATGATATGGGCCAATATCTTTAGTT
ACTGAAATTCCGTTTAGACCAAAACTGACCCTATACTGAATGTCATGTATCATAGCACAAGGTTTGTCTTTCCAA
AGTATCGCCCCACGCAAAATTTTCTGTTTTGCTTTGATGATGCATTCAGCTTATTTGTTGGTTTATAAATCTATT
GCCTGTCGTTAAGCAGGGGTGGAAGATGGGGTTAGGATGATGTGCAGTTTTGATAATTTTGATGTGCTTTTGGGT
AATGTGGCTTTTTAGCTGTCACATGGAACCCACAACTTAAAATATTTATTCTCATTCTATCTTTTGAGAAGAAAG
AGTTGTGAAGCAGTTGCATTAAGTGCTTTTATTCTCATCATAGGGAATGTTGGCAGCTCCAATTGGTCTTTTAGC
ACCATAGGCACATAACACCATCAATTAATTGAGCTATTGATTTGGTCGAAGTCTCAAATTTTATTGTTATCACAT
GGACATTAATGTAGACTACGATTCTTGGTAATATAATTGTGTGATTGTACATAATGATTCGGAGAAACAACAAAA
GCTCATGGCGATTAGCTTCTTGATTTTGATAAGAGAATTCATGTAGTAGTTCTAAAATTCTTTTATGTTTCAGCT
TTTGGCTCCTCTCCCCATTGGGTTTGCAGTATTTTTGGTTCATCTGGCCACCATCCCCATCACTGGAACCAGCAT

CAACCCTGCCAGGAGTCTTGGAGCTGCCATTATTTACAACAGAGATAACGCATGGGACGACCATGTAAGAATCTC
TCTATGCAGATCCTTTATAACTCTCGTAATTATTAATCAGTAGTACATTTTCAACTAAAACCATTGTTTCTTTAC
GTCGTCCAGTGGATCTTCTGGGTCGGACCCTTCATTGGAGCTGCTCTTGCTGCTATTTACCACCAGATTGTGATC
AGAGCCATTCCTTTCAAGAGCAGCCAGGCCTGT**TAA**

>*Oleur*PIP1;5a

ATGGCAGAAAACAGGGAAGAGGATGTTAGGCTTGGAGCCAACAAGTTTACTGAGAAGCAGCCTTTGGGTACAGCT
GCTCAAACAGACAAGGACTACAAGGAGCCACCTCCAGCCCCTTTGTTTGAGCCTGGGGAGATTAAATCATGGTCT
TTTTACAGGGCTGGTATTGCAGAGTTTGTGGCTACCTTCTTGTTCTTGTACATTACCATTTTGACTGTCATGGGG
GTTGGTAGATCAAATAGTAAGTGTGCCTCTGTGGGCATCCAGGGGATTGCTTGGTCCTTTGGTGGCATGATTTTT
GCACTTGTATACTGCACTGCTGGGATTTCAGGAGGGCACATCAACCCTGCTGTGACCTTCGGTTTGTTCTTGGCA
AGGAAGCTTTCCTTGACCAGAGCTATCTTCTACATAGTGATGCAGTGCCTTGGTGCAATCTGTGGTGCTGGTGTT
GTCAAGGGATTCATGGAGGGTCCATACCAGAGGCTACAAGGTGGGGCCAACGTTGTGCAGCATGGCTACACCAAG
GGTGATGGCCTTGGTGCTGAGATTGTTGGCACTTTTATTCTTCTTTTGGCTCCTCTCCCCATTGGGTTTGCAGTA
TTTTTGGTTCATCTGGCCACCATCCCCATCACTGGAACCAGCATCAACCCTGCCAGGAGTCTTGGAGCTGCCATT
ATTTACAACAGAGATAACGCATGGGACGACCATTGGATCTTCTGGGTCGGACCCTTCATTGGAGCTGCTCTTGCT
GCTATTTACCACCAGATTGTGATCAGAGCCATTCCTTTCAAGAGCAGCCAGGCCTGTTAA

>*Oleur*PIP1;5a

MAENREEDVRLGANKFTEKQPLGTAAQTDKDYKEPPPAPLFEPGEIKSWSFYRAGIAEFVATFLFLYITILTVMG
VGRSNSKCASVGIQGIAWSFGGMIFALVYCTAGISGGHINPAVTFGLFLARKLSLTRAIFYIVMQCLGAICGAGV
VKGFMEGPYQRLQGGANVVQHGYTKGDGLGAEIVGTFILLLAPLPIGFAVFLVHLATIPITGTSINPARSLGAAI
IYNRDNAWDDHWIFWVGPFIGAALAAIYHQIVIRAIPFKSSQAC*

*Oleur*PIP1;5b Oleur061Scf0451g01009.1

>Oleur061Scf0451:160899..163458 (- strand) class=gene length=2560

>*Oleur*PIP1;5b

ATGGCGGAGAACAGGGAAGAGGATGTGAGGCTTGGAGCCAACAAGTTCACTGAGAAGCAGCCTTTGGGTACAGCT
GCTCAAACAGACAAGGACTACAAGGAGCCACCTCCAGCCCCCTTGTTTGAGCCTGGGGAGCTGAAATCATGGTCT
TTTTACAGGGCTGGCATTGCAGAGTTTGTGGCCACTTTCTTGTTCTTGTACATAACGATCTTGACTGTTATGGGA
GTTGGTAGATCACATAATAAGTGTGCCTCTGTGGGCATCCAGGGGATTGCTTGGTCCTTTGGTGGCATGATTTTT
GCACTTGTATACTGCACTGCCGGGATTTCAGGTTAGTTTTTATCCAGATTACTGTGCTTTCTCTTTGCATGCCTA
CTGTTTGTTTCGTTTACTCTGAATTTGGGAAAAAATTGTGTCTTTCCTTTTGTTTTATGGGGTTTTCTGGGTTTT
GTTTTAGATAGGAAAGTTGTGGTAAATTATGAATGATTGTCACTGGCTCTGGTTGTATAAGTTCTTTATTTATCC
AGTGATATTTCATTTTGGAGAAAAGCTAGGATCTTGGTGATTTTTCCTAGCTACCCTGGTTTCAAATTGTCACTA
CTCTATAGAAGCTTTCAAATTTGAAGCTCAAAAACTGAATCTTTGTGCTGTCTGTTGAACAGGGGGGCACATCAA
CCCTGCTGTGACTTTTGGTTTGTTCTTGGCAAGGAAGCTTTCCTTTACCAGGGCTATCTTTTACATAGTGATGCA
GTGCCTTGGTGCAATCTGTGGTGCTGGTGTGGTCAAGGGATTCATGGAGGGTCCATACCAGAGACTGCAAGGTGG
GGCCAACATGGTCCAGCATGGCTACACCAAGGGTGATGGCCTTGGTGCTGAGATTGTTGGCACTTTTATTCTTGT
TTACACCGTCTTCTCTGCCACTGATGCCAAGAGAAGCGCCCGAGACTCACATGTTCCGGTATACCCTTCTTTGTT
ATTTATAGCCATAGGCCAATGTTTACTTTTCCTTATTGATACTTGATTAGACTTGCTGGTAAAGACAGGATTTGG
TGCTTTGATCTGTTAGTTACTACTTCAACTTAAGCTTTCCTAATTCCTATCATGCCTTGTTTACTAGTGTCCAAA
AAGTGGTATTGATGATTTAATTATAATTGTGTAGCTAGAATGCTCGGTTTTTTTCTAATTTCATTCGGACATTGA
GCACAAATGCTTATTTAGATCCACTTTCTTCAAGTCATTCAATGAAATCTTGACATGATATGAGGCTTATATGTT
TAGTTATTAAGGTGCCGTTCAGGCCAAAACTGACCTATACCGAATGTCACCAAACATGGATGTATCATCTCACCT
AGTTGTTGTCTGTTTGGCGCTTTTTATAATTGGTCAAACATGCTTTTTATTTGCATATTTTTGTGGGACCCATTA
GTACTATCCGAGAACCTGTAAAACTCGTGAGTCATTAAAGGTTATCATTTGCTATGTGAAAAGATAATCCATGC
AGTAGAGTCCTAAAATTCTTTAATCTTGCAGATTTTGGCTCCTCTCCCCATTGGGTTTGCGGTATTTTTGGTTCA
TTTGGCCACCATCCCCGTCACTGGAACCGGCATCAACCCAGCCAGGAGTCTTGGAGCTGCCATCATTTACAACAA
AGAGCACGTATGGAGCGACCACGTAAGAATCTCCCTATGCTTAGATTCATTATAATTTATCGTAATTATGAATCA
GTTGTAAATTTCAATTGAAACTCATGTTTGTTTACGTCGTTCAGTGGATCTTCTGGGTCGGACCCTTCATCGGAG
CTGCTCTTGCTGCAATCTACCACCAGATTGTCATCAGAGCCATTCCTTTCAAGAGCAGCAGGGCTTGT**TAA**

>*Oleur*PIP1;5b

ATGGCGGAGAACAGGGAAGAGGATGTGAGGCTTGGAGCCAACAAGTTCACTGAGAAGCAGCCTTTGGGTACAGCT
GCTCAAACAGACAAGGACTACAAGGAGCCACCTCCAGCCCCCTTGTTTGAGCCTGGGGAGCTGAAATCATGGTCT
TTTTACAGGGCTGGCATTGCAGAGTTTGTGGCCACTTTCTTGTTCTTGTACATAACGATCTTGACTGTTATGGGA
GTTGGTAGATCACATAATAAGTGTGCCTCTGTGGGCATCCAGGGGATTGCTTGGTCCTTTGGTGGCATGATTTTT
GCACTTGTATACTGCACTGCCGGGATTTCAGGGGGGCACATCAACCCTGCTGTGACTTTTGGTTTGTTCTTGGCA
AGGAAGCTTTCCTTTACCAGGGCTATCTTTTACATAGTGATGCAGTGCCTTGGTGCAATCTGTGGTGCTGGTGTG
GTCAAGGGATTCATGGAGGGTCCATACCAGAGACTGCAAGGTGGGGCCAACATGGTCCAGCATGGCTACACCAAG
GGTGATGGCCTTGGTGCTGAGATTGTTGGCACTTTTATTCTTGTTTACACCGTCTTCTCTGCCACTGATGCCAAG
AGAAGCGCCCGAGACTCACATGTTCCGATTTTGGCTCCTCTCCCCATTGGGTTTGCGGTATTTTTGGTTCATTTG

GCCACCATCCCCGTCACTGGAACCGGCATCAACCCAGCCAGGAGTCTTGGAGCTGCCATCATTTACAACAAAGAG
CACGTATGGAGCGACCACTGGATCTTCTGGGTCGGACCCTTCATCGGAGCTGCTCTTGCTGCAATCTACCACCAG
ATTGTCATCAGAGCCATTCCTTTCAAGAGCAGCAGGGCTTGT**TAA**

>*Oleur*PIP1;5b

MAENREEDVRLGANKFTEKQPLGTAAQTDKDYKEPPPAPLFEPGELKSWSFYRAGIAEFVATFLFLYITILTVMG
VGRSHNKCASVGIQGIAWSFGGMIFALVYCTAGISGGHINPAVTFGLFLARKLSFTRAIFYIVMQCLGAICGAGV
VKGFMEGPYQRLQGGANMVQHGYTKGDGLGAEIVGTFILVYTVFSATDAKRSARDSHVPILAPLPIGFAVFLVHL
ATIPVTGTGINPARSLGAAIIYNKEHVWSDHWIFWVGPFIGAALAAIYHQIVIRAIPFKSSRAC*

*Oleur*PIP1;6a Oleur061Scf6143g00002.1

>Oleur061Scf6143:10938..14002 (- strand) class=gene length=3065

>*Oleur*PIP1;6a

ATGGAGGGTAAAGAGGAGGATGTAAGGCTAGGAGCCAACAAATTCACAGAGAGGCAGCCATTGGGTACCTCAGCA
CAGAGCAAGGACTACAAGGAGCCACCTCCAGCTCCACTTTTTGAGCCTGGTGAACTCCAATCATGGTCCTTTTGG
AGGGCTGGGATTGCTGAGTTTATGGCCACTTTCTTGTTCCTTTATATCACGATCTTGACTGTAATGGGTGTGAGC
AGGTCTAACAGTAAGTGTACTTCTGTGGGTATTCAGGGCATTGCTTGGGCCTTTGGTGGCATGATTTTTGCCCTT
GTCTACTGCACTGCCGGCATCTCAGGTCTCTGTCTCTCAATTTGTGTGTGTTCTGTTCTCTGTTACTTAAATAAA
TTTAGGGTCTGCTGTTTGGTAGTGGACTGGATTTATAATGAACAAGATTTAACTTCGAACTTGCCAAGAATGTAT
TAGTACTACTAATATTGAGTTCTCATTTTCATGGGATCTTGGAAAATGTAAAGCATGCAGCTTCTGTCTAAGTTC
TACTACCTGTTGCTTAAGTGAATCGAGAGTCTTGCTAGTTTGGTATGCAGTATTAGATGGAACTAGTAGTATAAA
GAACAAGATTATATCTTCGAACATGCCAAGAATGTTAATGCACCACTAATTATGAGTCCATATTTTCATGGGATC
TTGAAAAATGTAAGTATGCGGCTTCTTTTTGTTTTTTATAATCTGTTGCTTAAGTAAATATCGAGTCTTGTGTGT
TTCGTGTGTAGAAGTGGATGGAATTATATCACATAATGTACAAGTATGATCTTCAAACATGCCAAGAATGTACTA
CTAGTTTAAAAAAATCTCGAGTCTTGCATGCTGGATGGAACTATATAGTATAATGAACAAAATTTGACAACATGC
CAAGAATGTACCACTGGTTTAAGTAAATCTAGATTCTTGGTAAGCAATACCAGACAACATTGAATAGTGAACAAG
ATTTGATCTTTAAACATGACAAGAACGTCCTATTACTAGAGTCTTGCCTGTTTAATACGTAGTATACGATGAGAC
TAAATAATATAATGAACAAGATTGATCTTCTAACATGCCAAGAATATACTACTAAGTAAATCCAGAGTCTCGACT
GTTTGGTAGCAATATTGGACAAGACGATTTTATCCTCCAAAAAAGACAAGAATGTACTAATACTTTTGAACCCTC
ATTTTCATGGGATTTTGGCAAATGGAAAGTAGAGTAATTTGTTATTGTTTGTGATAATTTGCTGTTGTTTTTTGA
GTTTTAAGTCTTGAATGTCACAGAATTTAAACCCTGCTTCTTCTTTTTTCAAATCCTGCTAAAATTTAATGGTTT
TTGTTCTTTTAGTTCTACTCTCCTTGATGCTAAGCAAAAAATATTTCTTCAAATATGAGTTCAACTTATAATTCA
TACAAAAAAAGGCCAAATCTTTGATATAATTTCTGGAAAAGAACTTATGATAAGTGATTATAATGCCCTTGTTTA
AAGATTATTGGAGCTTTTCATGTGCTGATATTTGAAATTTTTTATGGGTAATTTCAGGTGGACACATAAATCCTG
CAGTAACATTTGGTCTATTCGTAGCAAGGAAATTGTCTCTGACCAGGGCAATTTTCTACATTGTGATGCAATGCC
TTGGCGCAATCTGCGGCGCGGGTGTCGTAAAGGGCTTCGGGCCTTCTTTTTACAAGGCTAACGGTGGTGGTGCTA
ATGTTGTTGCCCATGGTTACACTAAGGGTGATGGACTTGGTGCTGAGATTATTGGCACCTTTGTTCTTGTTTACA
CTGTCTTCTCTGCTACTGATGCCAAGAGAAATGCCAGAGACTCGCATGTCCCTGTGAGTATTTCTTTTTAATCGT
GATTTTGAAAAGTGTTTTACTTTCAATGCAGTTTAGTATTAGTATTATTTTTTATGCTGAGGAAGGTATCATAGA
TATTTTAACCATGTATTCTTGCCTCGTCGGCTATAGATTATGTTTGTGTTGGGAAAATTGGACTTTCTTCATTGA
GAGTGGGCAAGACCCGGTCTGCATTCTGTTTGATTGTGCTCGATCTGAAATTCGAGTTCTTGAAAAGATATGTTG
GGCTACGATGGAATGATTTGGTCAATCAATAGTGTGCTCGAGCACTTTGATTCGAAATTCTGAAGTGCTGATTCG
ACTTTCTTTTGCACTTTGATTCTTCTTCTATTTCATGGTCAAACTAGAAACGAGTAATTTTGCAGATTTAACTTG
GCCTCAACTGTATGATTTGTGCCATAACTAGAATCTCATTGTGGGATGCCATCTTGATGACTACTAATTTTTCGA
ATTTCATAGATTTTAGCTCCACTCCCAATTGGGTTTGCCGTCTTTTTGGTTCACTTGGCAACAATCCCCATCACT
GGAACCGGTATCAACCCAGCCCGAAGCCTTGGTGCTGCCATCATTTACAACAAAGATCACGCTTGGGACGACCAT
GTAAGTTTTACGTCTGTAACTTCCATAGACTATGTTCTTACCTGTTCATGACATATGATTAGGCGCCCGGCCGTA
CTTATATTTGTGTCACTTCTGTCTTTCTTCAGTGGATCTTTTGGGTCGGACCTTTCATCGGAGCTGCACTCGCTG
CCCTTTACCACCAGGTTATCATTAGAGCTATCCCATTCAAGAGTGGCAAC**TGA**

>*Oleur*PIP1;6a

ATGGAGGGTAAAGAGGAGGATGTAAGGCTAGGAGCCAACAAATTCACAGAGAGGCAGCCATTGGGTACCTCAGCA
CAGAGCAAGGACTACAAGGAGCCACCTCCAGCTCCACTTTTTGAGCCTGGTGAACTCCAATCATGGTCCTTTTGG
AGGGCTGGGATTGCTGAGTTTATGGCCACTTTCTTGTTCCTTTATATCACGATCTTGACTGTAATGGGTGTGAGC
AGGTCTAACAGTAAGTGTACTTCTGTGGGTATTCAGGGCATTGCTTGGGCCTTTGGTGGCATGATTTTTGCCCTT
GTCTACTGCACTGCCGGCATCTCAGGTGGACACATAAATCCTGCAGTAACATTTGGTCTATTCGTAGCAAGGAAA
TTGTCTCTGACCAGGGCAATTTTCTACATTGTGATGCAATGCCTTGGCGCAATCTGCGGCGCGGGTGTCGTAAAG
GGCTTCGGGCCTTCTTTTTACAAGGCTAACGGTGGTGGTGCTAATGTTGTTGCCCATGGTTACACTAAGGGTGAT
GGACTTGGTGCTGAGATTATTGGCACCTTTGTTCTTGTTTACACTGTCTTCTCTGCTACTGATGCCAAGAGAAAT
GCCAGAGACTCGCATGTCCCTATTTTAGCTCCACTCCCAATTGGGTTTGCCGTCTTTTTGGTTCACTTGGCAACA
ATCCCCATCACTGGAACCGGTATCAACCCAGCCCGAAGCCTTGGTGCTGCCATCATTTACAACAAAGATCACGCT
TGGGACGACCATTGGATCTTTTGGGTCGGACCTTTCATCGGAGCTGCACTCGCTGCCCTTTACCACCAGGTTATC
ATTAGAGCTATCCCATTCAAGAGTGGCAAC**TGA**

>*Oleur*PIP1;6a
MEGKEEDVRLGANKFTERQPLGTSAQSKDYKEPPPAPLFEPGELQSWSFWRAGIAEFMATFLFLYITILTVMGVS
RSNSKCTSVGIQGIAWAFGGMIFALVYCTAGISGGHINPAVTFGLFVARKLSLTRAIFYIVMQCLGAICGAGVVK
GFGPSFYKANGGGANVVAHGYTKGDGLGAEIIGTFVLVYTVFSATDAKRNARDSHVPILAPLPIGFAVFLVHLAT
IPITGTGINPARSLGAAIIYNKDHAWDDHWIFWVGPFIGAALAALYHQVIIRAIPFKSGN*

*Oleur*PIP1;6b Oleur061Scf8779g00011.1

>Oleur061Scf8779:132959..136100 (- strand) class=gene length=3142

>*Oleur*PIP1;6b
ATGGAGGGTAAAGAGGAGGATGTAAGGCTAGGAGCCAACAAATTCACAGAGAGGCAGCCATTGGGTACCTCAGCA
CAGAGCAAGGACTACAAGGAGCCACCTCCAGCTCCACTTTTTGAGCCTGGTGAACTCCAATCATGGTCCTTTTGG
AGGGCTGGGATTGCTGAGTTTATGGCCACTTTCTTGTTCCTTTATATCACGATCTTGACTGTAATGGGTGTGAGC
AGGTCTAACAGTAAGTGTACTTCTGTGGGTATTCAGGGCATTGCTTGGGCCTTTGGTGGCATGATTTTTGCCCTT
GTCTACTGCACTGCCGGCATCTCAGGTCTCTGTCTCTCAATTTGTGTGTGTTCTGTTCTCTGTTACTTAAATAAA
TTTAGGGTCTGCTGTTTGGTAGTGGACTGGATTTATAATGAACAAGATTTAACTTCGAACTTGCCAAGAATGTAT
TAGTACTACTAATATTGAGTTCTCATTTTTATGGATCTTGGAAAATGTAAAGCATGCAGCTTCTGTCTAAGTTC
TACTACCTGTTGCTTAAGTGAATCGAGAGTCTTGCTAGTTTGGTATGCAGTATTAGATAGAACTAGTAGTATAAA
GAACAAGATTATATCTTCGAACATGCCAAGAATGTTAATGCACCACTAATTTTGAGTCCATATTTTCATGGGATC
TTGAAAAATGTAAGTATGCGGCTTCTTTTTGTTTTTTATAATCTGTTGCTTAAGTAAATATCGAGTCTTGTGTGT
TTCGTGTGTAGAACTGGATGGAATTATATCACATAATGTACAAGTATGATCTTCAAACATGCCAAGAATGTACTA
CTAGTTTAAAAAAATCTCGAGTCTTGCATGCTGGATGGAACTATATAGTATAATGAACAAAATTTGACAACATGC
CAAGAATGTACCACTGGTTTAAGTAAATCTAGATTCTTGGTAAGCAATACCAGACAACATTGAATAGTGAACAAG
ATTTGATCTTTAAACATGACAAGAACGTCCTATTACTAGAGTCTTGCCTGTTTAATACGTAGTATACGATGAGAC
TAAATAATATAATGAACAAGATTGATCTTCTAACATGCCAAGAATATACTACTAGTTTAAGTAAATCCAGAGTCT
TGACTGTTTGGTAGCAATATTGGACAAGACGATTTTATCCTCCAAAAAAGACAAGAATGTACTAATACTTTTGAA
CCCTCATTTTCATGGGATTTTGGCAAATGGAAAGTAGAGTAATTTCTTATTGTTTGTGATAATTTGCTGTTGTTT
TTTGAGTTTTAAGTCTTGAATGTCACAGAATTTAAACCCTGCTTTTTTTTATCAAATCCTGCTAAAATTTAATG
GTTTTTGTTCTTTTAGTTCTACTCTCCTTGATGCTAAGCAAAAAATATTTCTTCAAATATGAGTTCAACTTATAA
TTCATACAAAAAAAGGTCAAATCTTTGATATAATTTCTGGAAAAGAACTTATGATTAGTGATTATAATGCCCTTG
TTTAAAGATTATTGGAGCTTTTCATGTGCTGATATTTGAAATTTTTTATGGGTAATTTCAGGTGGACACATAAAT
CCTGCAGTAACATTTGGTCTATTCCTAGCAAGGAAATTGTCTCTTACCAGGGCAATTTTCTACATTGTGATGCAA
TGCCTTGGCGCAATCTGCGGCGCGGGTGTCGTAAAGGGCTTCGGGCCTTCTTTTTACAAGGCTAACGGTGGTGGT
GCAAATGTTGTTGCCCATGGTTACACTAAGGGTGATGGACTTGGTGCTGAGATTATTGGCACCTTTGTTCTTGTT
TACACTGTCTTCTCTGCTACTGATGCCAAGAGAAATGCCAGAGACTCGCATGTCCCCGTGAGTATTTCTTTGTAA
TCGTGATTTTGAAAAGTGTTTTACTTTCAATGCAGTTTAGTATTAGTATTATTTTTTATGCTGAGGAAGGTATCA
TAGATATTTTAACCATGTATTCTTGCCTCGTCGGCTATAGATTATGTTTGTGTTGGGAAAATTGGACTTTCTTCA
TTGAGAGTGGGCAAGACCGGGTCTGCATTCTGTTTGATTGTGCTCGATCTGAAATTCGAGTTCTTGAAAATATAT
GTTGGGCTACGATGGAATGATTTGGTCAATCAATAGTGTGCTCGAGCACTTTGATTTGAAATTCTGAAGTGCTGA
TTCGACTTTCTTTTGCACTTCGATTCTTCTTCTATTTCATGGTCAAACTAGAAACGAGTAATTTGCAGATTTAA
CTTGGCCTCAACTGTATGATTTGTGCCATAACTAGAATCTCATTGTGGGATGCCATCTTGATGACTACTAATTTT
TCGAATTTCATAGATTTTAGCTCCACTCCCAATTGGGTTTGCCGTCTTTTTGGTTCACTTGGCAACAATCCCCAT
CACTGGAACCGGTATCAACCCAGCCCGAAGCCTTGGTGCTGCCATCATTTACAACAAAGATCACGCTTGGGACGA
CCATGTAAGTTTTACGTCTGTAACTTCCATAGACTATGTTCTTACCTGTTCATGACATATGATTAGGCGCCCGGC
CGTACTTATATTTGTGTCACTTCTGTCTTTCTTCAGTGGATCTTTTGGGTCGGACCTTTCATCGGAGCTGCACTC
GCTGCCCTTTACCACCAGGTTATCATTAGAGCTATCCCATTCAAGAGTGGCAAC**TGA**

>*Oleur*PIP1;6b
ATGGAGGGTAAAGAGGAGGATGTAAGGCTAGGAGCCAACAAATTCACAGAGAGGCAGCCATTGGGTACCTCAGCA
CAGAGCAAGGACTACAAGGAGCCACCTCCAGCTCCACTTTTTGAGCCTGGTGAACTCCAATCATGGTCCTTTTGG
AGGGCTGGGATTGCTGAGTTTATGGCCACTTTCTTGTTCCTTTATATCACGATCTTGACTGTAATGGGTGTGAGC
AGGTCTAACAGTAAGTGTACTTCTGTGGGTATTCAGGGCATTGCTTGGGCCTTTGGTGGCATGATTTTTGCCCTT
GTCTACTGCACTGCCGGCATCTCAGGTGGACACATAAATCCTGCAGTAACATTTGGTCTATTCCTAGCAAGGAAA
TTGTCTCTTACCAGGGCAATTTTCTACATTGTGATGCAATGCCTTGGCGCAATCTGCGGCGCGGGTGTCGTAAAG
GGCTTCGGGCCTTCTTTTTACAAGGCTAACGGTGGTGGTGCAAATGTTGTTGCCCATGGTTACACTAAGGGTGAT
GGACTTGGTGCTGAGATTATTGGCACCTTTGTTCTTGTTTACACTGTCTTCTCTGCTACTGATGCCAAGAGAAAT
GCCAGAGACTCGCATGTCCCCATTTTAGCTCCACTCCCAATTGGGTTTGCCGTCTTTTTGGTTCACTTGGCAACA
ATCCCCATCACTGGAACCGGTATCAACCCAGCCCGAAGCCTTGGTGCTGCCATCATTTACAACAAAGATCACGCT
TGGGACGACCATTGGATCTTTTGGGTCGGACCTTTCATCGGAGCTGCACTCGCTGCCCTTTACCACCAGGTTATC
ATTAGAGCTATCCCATTCAAGAGTGGCAAC**TGA**

>*Oleur*PIP1;6b

MEGKEEDVRLGANKFTERQPLGTSAQSKDYKEPPPAPLFEPGELQSWSFWRAGIAEFMATFLFLYITILTVMGVS
RSNSKCTSVGIQGIAWAFGGMIFALVYCTAGISGGHINPAVTFGLFLARKLSLTRAIFYIVMQCLGAICGAGVVK
GFGPSFYKANGGGANVVAHGYTKGDGLGAEIIGTFVLVYTVFSATDAKRNARDSHVPILAPLPIGFAVFLVHLAT
IPITGTGINPARSLGAAIIYNKDHAWDDHWIFWVGPFIGAALAALYHQVIIRAIPFKSGN*

*Oleur*PIP2;1a Oleur061Scf0523g01011.1

>Oleur061Scf0523:148832..151185 (+ strand) class=gene length=2354

>*Oleur*PIP2;1a
ATGACGAAAGACGTCGAATCTCATGTGGAGCCAGCGGAGGAATACTCCGCCAAGGACTACCATGATCCGCCACCG
GCGCCTCTCATTGATTTTGATGAGCTGAGCAGCTGGTCGTTTTACAGAGCACTTATTGCTGAGTTCGTTGCCACT
TTGCTGTTCCTTTACGTCACAGTTTTGACCGTTATAGGCTACAAAAGCCAGATTGACCCAGCTCACGGCGGCGAC
GACTGCGGCGGCGTTGGGATTCTTGGCATTGCATGGGCTTTCGGCGGCATGATTTTCATTCTTGTCTACTGCACC
GCCGGTATCTCTGGTCAGTTTTACAGGTTTCAGCCAGCTTTTCCTTGATTGGTACCTGCACCATAATAAGTAAAT
TACTTGTGAAAATAAGTAACTTTTTCATATTGTGACAGGAGGACATATTAACCCGGCAGTGACATTAGGGCTGTT
CTTGGGAAGAAAGGTGTCACTGATCAGAGCAGTGCTTTACATGGTAGCACAATGTTTGGGTGCAATCTGTGGTGT
GGGATTGGTGAAAGCATTCCAAAAATCGTACTATCATAAGTATGGTGGTGGAGCAAATGAACTTGCTCCAGGGTA
CAACAAAGGGGTTGGTTTGGGTGCTGAAATTATTGGCACCTTTGTTTTGGTCTACACCGTTTTCTCAGCCACTGA
TCCTAAGAGGAATGCCAGAGACTCCCATGTCCCTGTAAGCACTTTTTTTCTCTGCTAACGCACCCACACCCCCAA
GTCTTATTATCCCAAGGGAGGAGTTCTCCTCGGAATAACAACCCCTTGATTGGATGTTTTAAATTTGTTTTTTGG
GCAAAATCCAATGAAGTTTTGCCATTTGACAACCTGTAACTCCTCTGGCAAATTTGTTGTACATATAACATGGTT
CGTTCTCCACAGAATCATTTTTTTGCCACTAATCTGTCGTACTAAAGGTGTCATAAAATCGCGTGGGGTTCATCA
GGGTCCTAAATTAATTTGTACACGGGTCAACGGGCAATAATAAGTTCTTGACCCAAAGTCCATCAATGAACTTTA
ATTTATAGAAAATTTTGGATTATATCCTTGCAAATATTGATTTAATCTGATACATATGAAGGTCTTGGCTCCACT
TCCCATCGGATTTGCTGTGTTCATGGTTCACCTTGCCACCATCCCCATCACCGGAACTGGCATCAACCCTGCTAG
GAGCTTTGGAGCTGCTGTCATCTACAACGAAGACAAGCCCTGGGATGACCATGTATGCCTCCCTTTTACCTCTTT
TTTACTGATTTTACACGCACAATAAGTTGATCAATCCTTTTTTTTAAATTGTAATTATTTTTATAGAATTTAATG
ACATAATTCATAAAAGCTAGTCAAGTCCCCAATTTATCTTTTTAGTTTTTGGACAAGGGTATCTCAATTTGATAC
AAAAACAAGTAGGAGGTCCCGAGTTCAAGTTGCACTTCCACTCATTTATAAAAATATGCACGTCTCATGTCTGCG
CATGAGGACATGTGTTCAAGACGTAATTTAACACATTCCTCTCCCGTTAACACATTAAGCCCCTGTTTGATAGGG
ATTAAAATCAGATTTATAATAGATTTTGATAAAAGTTGATGAAATGTGTACTTGAAAAGTTGTGGGTCCCCTAAA
ATATGTTGTTAGAATTAATTATTAAATTTGACTTAGATTAAAATCTGAATTTTAATTTTTACCAAACATGGACTA
AGCTTTTAAACGAGTTATTAAAAACGAAGATAGTACTAATATATTTGTTAATATTCAGTGGATTTTTTGGGTTGG
ACCATTCGTTGGAGCTCTAATTGCTGCAATCTACCACCAATACATCCTAAGGGCAGGAGCCATCAAGGCACTGGG
TTCTTTCAGGAGCAACGCTTAA
>*Oleur*PIP2;1a
ATGACGAAAGACGTCGAATCTCATGTGGAGCCAGCGGAGGAATACTCCGCCAAGGACTACCATGATCCGCCACCG
GCGCCTCTCATTGATTTTGATGAGCTGAGCAGCTGGTCGTTTTACAGAGCACTTATTGCTGAGTTCGTTGCCACT
TTGCTGTTCCTTTACGTCACAGTTTTGACCGTTATAGGCTACAAAAGCCAGATTGACCCAGCTCACGGCGGCGAC
GACTGCGGCGGCGTTGGGATTCTTGGCATTGCATGGGCTTTCGGCGGCATGATTTTCATTCTTGTCTACTGCACC
GCCGGTATCTCTGGAGGACATATTAACCCGGCAGTGACATTAGGGCTGTTCTTGGGAAGAAAGGTGTCACTGATC
AGAGCAGTGCTTTACATGGTAGCACAATGTTTGGGTGCAATCTGTGGTGTGGGATTGGTGAAAGCATTCCAAAAA
TCGTACTATCATAAGTATGGTGGTGGAGCAAATGAACTTGCTCCAGGGTACAACAAAGGGGTTGGTTTGGGTGCT
GAAATTATTGGCACCTTTGTTTTGGTCTACACCGTTTTCTCAGCCACTGATCCTAAGAGGAATGCCAGAGACTCC
CATGTCCCTGTCTTGGCTCCACTTCCCATCGGATTTGCTGTGTTCATGGTTCACCTTGCCACCATCCCCATCACC
GGAACTGGCATCAACCCTGCTAGGAGCTTTGGAGCTGCTGTCATCTACAACGAAGACAAGCCCTGGGATGACCAT
TGGATTTTTTGGGTTGGACCATTCGTTGGAGCTCTAATTGCTGCAATCTACCACCAATACATCCTAAGGGCAGGA
GCCATCAAGGCACTGGGTTCTTTCAGGAGCAACGCTTAA
>*Oleur*PIP2;1a
MTKDVESHVEPAEEYSAKDYHDPPPAPLIDFDELSSWSFYRALIAEFVATLLFLYVTVLTVIGYKSQIDPAHGGD
DCGGVGILGIAWAFGGMIFILVYCTAGISGGHINPAVTLGLFLGRKVSLIRAVLYMVAQCLGAICGVGLVKAFQK
SYYHKYGGGANELAPGYNKGVGLGAEIIGTFVLVYTVFSATDPKRNARDSHVPVLAPLPIGFAVFMVHLATIPIT
GTGINPARSFGAAVIYNEDKPWDDHWIFWVGPFVGALIAAIYHQYILRAGAIKALGSFRSNA*

*Oleur*PIP2;1b Oleur061Scf0872g05024.1

>Oleur061Scf0872:512608..514991 (+ strand) class=gene length=2384

>*Oleur*PIP2;1b

ATGACGAAAGACGTCGAATCTCATGTGGAGCCAGCGGAGGAATACTCCGCCAAGGACTACCATGATCCACCACCG
GCGCCTCTCATTGATTTTGATGAGCTGAGCAGCTGGTCGTTTTACAGAGCACTTATTGCTGAGTTCGTTGCCACT
TTGCTGTTCCTTTACGTCACAGTTTTGACCGTTATAGGCTACAAAAGCCAGATTGACCCAGCTCACGGCGGCGAC
GACTGCGGCGGCGTTGGGATTCTTGGCATTGCATGGGCTTTCGGTGGAATGATTTTCATTCTTGTTTACTGCACC
GCCGGTATCTCTGGTCAGTTTTACAAGTTTCAGCCAGCTTTTCCTTGATTGGTACCTGCACCATAATAAGTAAAT
TACTCGTGAAAATAAGTAACTTTTTCATATTGTGACAGGAGGACATATTAACCCGGCAGTGACATTAGGGCTGTT
CTTGGGAAGAAAGGTGTCACTGATCAGAGCAGTGCTTTACATGGTAGCACAATGTTTGGGTGCAATCTGTGGTGT
GGGATTGGTGAAAGCATTCCAAAAATCGTACTATCATAAGTATGGTGGTGGAGCAAATGAACTTGCTCCAGGGTA
CAACAAAGGGGTTGGTTTGGGTGCTGAAATTATTGGCACCTTTGTTTTGGTCTACACCGTCTTCTCAGCCACTGA
TCCTAAGAGGAATGCCAGAGACTCCCATGTCCCTGTAAGCACTTTTTTCTCTGCTAACCCACCCACACCCCCAAG
TCTTATTATCCCAAGAAAGGAGTTCTCCTCGGAATAAGCCAGGGAAGTGTACAGGGGTTGAGATGGCAGCCCTTG
ATTGGATGTTATAAATTTGTTTTTTTGGGCAAACTCCAATGAAGTTTTGCCATTTGACAACCTGTAACTCCTTTG
GCAAATTTGTTGTACATATAACATGGTTCGTTCTCCTCAGAATCAATTTTTTTGCCACTAATCTATCGTACTAAAG
GTGTCATAAAATCGCGTGGGGTTCATCAGGGTCCTTAATTGATTTGTACACAGTCAATGGGCAATAATAAGTTCC
TGACCCAAAGTCCATCAATGAACTTTAATTTATAGAAAATTTTGGATTATATCCTTGCAAATATTGATTTAATCT
GATACATATGAAGGTCTTGGCTCCACTTCCCATCGGATTTGCTGTGTTCATGGTTCACCTTGCCACCATCCCCAT
CACCGGAACTGGCATCAACCCTGCTAGGAGCTTTGGAGCTGCTGTCATCTACAACGAAGACAAGCCCTGGGATGA
CCATGTATGCCTCCCCTTTACCTCTTTTTTACTGATTTTACACGCACAATAAGTTGATCAATCCTTTTTTTTTTT
TTTTTTTTTAAATTTTAATTATTTTTGTAGACTTTAATGACATAATTCATAAAAGCTAGTCAAGTCCCCAATTTA
TCTTTTTAGTTTTTGGACAAGGGTATCTCAATTTGATACAAAAACAAGTAGGAGTTCCCGAGTTCAAGTTGCACT
TCCACTCATTTATAAAAATATGCACGTCTCATGTCTGCGCATGAGGACATGTGTTCAAGACATAATTTAATACAT
TCCTCTCCCGTTAACACATTAAGCCCCTGTTTGATAGGGATTAAATCAGATTTATAATAGATTTTGATAAAAGTT
AATGAAATGTGTGCTTGAAAAGTTGTGGTCCCCTAAAATATGTTGTTAGAATTAATTGTTAAATTTGACTTAAAT
TAAAATCTGAATTTTAATTTTTACCAAACATGGACTAAGCTTTTAAACGAGTTATTAAAAATGAAGATAGTACTA
ATATATTTGTTAATATACAGTGGATTTTTTGGGTTGGACCATTCGTTGGAGCTCTAATTGCTGCAATCTACCACC
AATACATCCTAAGGGCAGGAGCCATCAAGGCACTGGGATCTTTCAGGAGCAACGCT**TAA**

>*Oleur*PIP2;1b

ATGACGAAAGACGTCGAATCTCATGTGGAGCCAGCGGAGGAATACTCCGCCAAGGACTACCATGATCCACCACCG
GCGCCTCTCATTGATTTTGATGAGCTGAGCAGCTGGTCGTTTTACAGAGCACTTATTGCTGAGTTCGTTGCCACT
TTGCTGTTCCTTTACGTCACAGTTTTGACCGTTATAGGCTACAAAAGCCAGATTGACCCAGCTCACGGCGGCGAC
GACTGCGGCGGCGTTGGGATTCTTGGCATTGCATGGGCTTTCGGTGGAATGATTTTCATTCTTGTTTACTGCACC
GCCGGTATCTCTGGAGGACATATTAACCCGGCAGTGACATTAGGGCTGTTCTTGGGAAGAAAGGTGTCACTGATC
AGAGCAGTGCTTTACATGGTAGCACAATGTTTGGGTGCAATCTGTGGTGTGGGATTGGTGAAAGCATTCCAAAAA
TCGTACTATCATAAGTATGGTGGTGGAGCAAATGAACTTGCTCCAGGGTACAACAAAGGGGTTGGTTTGGGTGCT
GAAATTATTGGCACCTTTGTTTTGGTCTACACCGTCTTCTCAGCCACTGATCCTAAGAGGAATGCCAGAGACTCC
CATGTCCCTGTCTTGGCTCCACTTCCCATCGGATTTGCTGTGTTCATGGTTCACCTTGCCACCATCCCCATCACC
GGAACTGGCATCAACCCTGCTAGGAGCTTTGGAGCTGCTGTCATCTACAACGAAGACAAGCCCTGGGATGACCAT
TGGATTTTTTGGGTTGGACCATTCGTTGGAGCTCTAATTGCTGCAATCTACCACCAATACATCCTAAGGGCAGGA
GCCATCAAGGCACTGGGATCTTTCAGGAGCAACGCT**TAA**

>*Oleur*PIP2;1b

MTKDVESHVEPAEEYSAKDYHDPPPAPLIDFDELSSWSFYRALIAEFVATLLFLYVTVLTVIGYKSQIDPAHGGD
DCGGVGILGIAWAFGGMIFILVYCTAGISGGHINPAVTLGLFLGRKVSLIRAVLYMVAQCLGAICGVGLVKAFQK
SYYHKYGGGANELAPGYNKGVGLGAEIIGTFVLVYTVFSATDPKRNARDSHVPVLAPLPIGFAVFMVHLATIPIT
GTGINPARSFGAAVIYNEDKPWDDHWIFWVGPFVGALIAAIYHQYILRAGAIKALGSFRSNA*

*Oleur*PIP2;2a Oleur061Scf6622g04008.1

>Oleur061Scf6622:431311..433104 (- strand) class=gene length=1794

>*Oleur*PIP2;2a

ATGACGAAAGACGTTGAATCTCACGGGGAGCCGGCGGAATTCTCGGCCAAGGACTACCATGATCCACCACCGGCG
CCGCTCATTGATTTTGAAGAGCTGAGCAGCTGGTCATTTTACAGAGCACTTATTGCTGAGTTCATTGCCACTTTA
CTGTTCCTCTACGTCACAGTTTTGACTGTTATTGGATACAAAAGCCAGATTGACCCCGCTCACGGCGGCGATCAG
TGCGGCGGCGTCGGGATTCTAGGCATTGCATGGGCTTTCGGTGGCATGATTTTCATTCTTGTTTACTGCACCGCC
GGTATCTCTGGTCAGTTTTAAAAGTCTTTACAAGCTTTTTTCCTTATAGATTTTGATGAGTGACTTACTGGTGAA
ATAAGTAACTTTTTGATAATGTGACAGGAGGACACATTAACCCGGCTGTGACATTCGGGCTGTTCTTGGGACGAA
AGGTGTCACTGATCAGAGCAGTGATGTACATGGTAGCACAGTGTTTGGGTGCAATCTGTGGTGTGGGTTTGGTGA
AAGCATTCCAAAAATCTTATTACAACCTGTACGGTGGTGGAGCAAATGAGCTTGCTCCAGGTTACAACAAAGGTG
TTGGATTGGGTGCTGAAATTATTGGCACATTTGTTTTGGTTTACACTGTCTTCTCAGCCACTGATCCTAAGAGGA
ATGCCAGAGATTCCCATGTTCCCGTAAGCAATTTTTTTTCTCTTCTAACCCAAACTCTGGTAAAATTTTTGGTTA
TAGTCTTGAAAATATTGATTTAAATCTTACATATATGAAGGTGTTGGCTCCACTTCCCATTGGATTTGCTGTTTT
CATGGTTCACCTGGCCACCATCCCCATCACTGGAACTGGCATCAACCCTGCTAGGAGCTTTGGAGCTGCTGTCAT

CTACAACAAAGACAAGCCCTGGGATGACCATGTATGCTCTTTTTTACTTCTTTAGGCCCTGTTTTGACGGTCTTT
TTTTTTTTTTTTGGAAAGAATGTATTTTTGAATAGTAACTTTTTGGAGAATGCGTGATTAAAAAGTAAAAAATGT
CTTGGGTTTATATGAATAATATTTTATTGTGAGAATGTATGCTCGAAAATGAATATGGGAAAGTAGAGTAATGA
AAGAATTACCGAAGAGGGGCTTATATAAATAATTATTTCATGTCTTGTTTTCACTTGTTTATGAAGGGTAGATGT
GTGGAATATTTGATAAAAAAGATAAACGAGGTTGTGTTGAATACATAATTTAAAGAAGTTTATCTGTTGTTTAAC
AACTTGAACTTTTGGATAAGGTTTTTGAGACTAACATATGTTATTACTTTTGTTTAAATAAATATGCAGTGGATT
TTCTGGGTTGGGCCATTTATTGGAGCTGCCATTGCTGCATTCTACCACCAATACATTCTAAGGGCAGCAGCCATT
AAGGCACTGGGATCTTTCAGGAGCAATAAT**TAA**

>*Oleur*PIP2;2a

ATGACGAAAGACGTTGAATCTCACGGGGAGCCGGCGGAATTCTCGGCCAAGGACTACCATGATCCACCACCGGCG
CCGCTCATTGATTTTGAAGAGCTGAGCAGCTGGTCATTTTACAGAGCACTTATTGCTGAGTTCATTGCCACTTTA
CTGTTCCTCTACGTCACAGTTTTGACTGTTATTGGATACAAAAGCCAGATTGACCCCGCTCACGGCGGCGATCAG
TGCGGCGGCGTCGGGATTCTAGGCATTGCATGGGCTTTCGGTGGCATGATTTTCATTCTTGTTTACTGCACCGCC
GGTATCTCTGGAGGACACATTAACCCGGCTGTGACATTCGGGCTGTTCTTGGGACGAAAGGTGTCACTGATCAGA
GCAGTGATGTACATGGTAGCACAGTGTTTGGGTGCAATCTGTGGTGTGGGTTTGGTGAAAGCATTCCAAAAATCT
TATTACAACCTGTACGGTGGTGGAGCAAATGAGCTTGCTCCAGGTTACAACAAAGGTGTTGGATTGGGTGCTGAA
ATTATTGGCACATTTGTTTTGGTTTACACTGTCTTCTCAGCCACTGATCCTAAGAGGAATGCCAGAGATTCCCAT
GTTCCCGTGTTGGCTCCACTTCCCATTGGATTTGCTGTTTTCATGGTTCACCTGGCCACCATCCCCATCACTGGA
ACTGGCATCAACCCTGCTAGGAGCTTTGGAGCTGCTTGGATTTTCTGGGTTGGGCCATTTATTGGAGCTGCCATT
GCTGCATTCTACCACCAATACATTCTAAGGGCAGCAGCCATTAAGGCACTGGGATCTTTCAGGAGCAATAAT**TAA**

>*Oleur*PIP2;2a

MTKDVESHGEPAEFSAKDYHDPPPAPLIDFEELSSWSFYRALIAEFIATLLFLYVTVLTVIGYKSQIDPAHGGDQ
CGGVGILGIAWAFGGMIFILVYCTAGISGGHINPAVTFGLFLGRKVSLIRAVMYMVAQCLGAICGVGLVKAFQKS
YYNLYGGGANELAPGYNKGVGLGAEIIGTFVLVYTVFSATDPKRNARDSHVPVLAPLPIGFAVFMVHLATIPITG
TGINPARSFGAAWIFWVGPFIGAAIAAFYHQYILRAAAIKALGSFRSNN*

*Oleur*PIP2;2b Oleur061Scf6622g04008.1

>Oleur061Scf8869:18552..20344 (- strand) class=gene length=1793

>*Oleur*PIP2;2b

ATGACGAAAGACGTTGAATCTCACGGGGAGCCGGCGGAATTCTCGGCCAAGGACTACCATGATCCACCACCGGCG
CCGCTCATTGATTTTGAAGAGCTGAGCAGCTGGTCATTTTACAGAGCACTTATTGCTGAGTTCATTGCCACTTTA
CTGTTCCTCTACGTCACAGTTTTGACTGTTATTGGATACAAAAGCCAGATTGACCCCGCTCACGGCGGCGATCAG
TGCGGCGGCGTCGGGATTCTAGGCATTGCATGGGCTTTCGGTGGCATGATTTTCATTCTTGTTTACTGCACCGCC
GGTATCTCTGGTCAGTTTTAAAAGTCTTTACAAGCTTTTTTCCTTATAGATTTTGATGAGTGACTTACTGGTGAA
ATAAGTAACTTTTTGATAATGTGACAGGAGGACACATTAACCCGGCTGTGACATTCGGGCTGTTCTTGGGACGAA
AGGTGTCACTGATCAGAGCAGTGATGTACATGGTAGCACAGTGTTTGGGTGCAATCTGTGGTGTGGGTTTGGTGA
AAGCATTCCAAAAATCTTATTACAACCTGTACGGTGGTGGAGCAAATGAGCTTGCTCCAGGTTACAACAAAGGTG
TTGGATTGGGTGCTGAAATTATTGGCACTTTTGTTTTGGTTTACACTGTCTTCTCAGCCACTGATCCTAAGAGGA
ATGCCAGAGATTCCCATGTTCCCGTAAGCAATTTTTTCTCTTCTAACCCAAACTCTGGTAAAATTTTTGGTTATA
GTCTTGAAAATATTGATTTAAATCTTACATATATGAAGGTGTTGGCTCCACTTCCCATTGGATTTGCTGTTTTCA
TGGTTCACCTGGCCACCATCCCCATCACTGGAACTGGCATCAACCCTGCTAGGAGCTTTGGAGCTGCTGTCATCT
ACAACAAAGACAAGCCCTGGGATGACCATGTATGCTCTTTTTTACTTCTTTAGGCCCTGTTTTGACGGTCTTTTT
TTTTTTTTTTTGGAAAGAATGTATTTTTGAATAGTAACTTTTTGGAGAATGCGTGATTAAAAAGTAAAAAATGTC
TTGGGTTTATATGAATAATATTTTATTGTGAGAATGTATGCTCGAAAATGAATATGGGAAAGTAGAGTAATGAA
AGAATTACCGAAGAGGGGCTTATATAAATAATTATTTCATGTCTTGTTTTCACTTGTTTATGAAGGGTAGATGTG
TGGAATATTTGATAAAAAAGATAAACGAGGTTGTGTTGAATACATAATTTAAAGAAGTTTATCTGTCGTTTAACA
ACTTGAACTTTTGGATAAGGTTTTTGAGACTAACATATGTTATTACTTTTGTTTAAATAAATATGCAGTGGATTT
TCTGGGTTGGGCCATTTATTGGAGCTGCCATTGCTGCATTCTACCACCAATACATTCTAAGGGCAGCAGCCATTA
AGGCACTGGGATCTTTCAGGAGCAATAAT**TAA**

>*Oleur*PIP2;2b

ATGACGAAAGACGTTGAATCTCACGGGGAGCCGGCGGAATTCTCGGCCAAGGACTACCATGATCCACCACCGGCG
CCGCTCATTGATTTTGAAGAGCTGAGCAGCTGGTCATTTTACAGAGCACTTATTGCTGAGTTCATTGCCACTTTA
CTGTTCCTCTACGTCACAGTTTTGACTGTTATTGGATACAAAAGCCAGATTGACCCCGCTCACGGCGGCGATCAG
TGCGGCGGCGTCGGGATTCTAGGCATTGCATGGGCTTTCGGTGGCATGATTTTCATTCTTGTTTACTGCACCGCC
GGTATCTCTGGAGGACACATTAACCCGGCTGTGACATTCGGGCTGTTCTTGGGACGAAAGGTGTCACTGATCAGA
GCAGTGATGTACATGGTAGCACAGTGTTTGGGTGCAATCTGTGGTGTGGGTTTGGTGAAAGCATTCCAAAAATCT
TATTACAACCTGTACGGTGGTGGAGCAAATGAGCTTGCTCCAGGTTACAACAAAGGTGTTGGATTGGGTGCTGAA
ATTATTGGCACTTTTGTTTTGGTTTACACTGTCTTCTCAGCCACTGATCCTAAGAGGAATGCCAGAGATTCCCAT
GTTCCCGTGTTGGCTCCACTTCCCATTGGATTTGCTGTTTTCATGGTTCACCTGGCCACCATCCCCATCACTGGA

ACTGGCATCAACCCTGCTAGGAGCTTTGGAGCTGCTTGGATTTTCTGGGTTGGGCCATTTATTGGAGCTGCCATT
GCTGCATTCTACCACCAATACATTCTAAGGGCAGCAGCCATTAAGGCACTGGGATCTTTCAGGAGCAATAAT**TAA**

>*Oleur*PIP2;2b
MTKDVESHGEPAEFSAKDYHDPPPAPLIDFEELSSWSFYRALIAEFIATLLFLYVTVLTVIGYKSQIDPAHGGDQ
CGGVGILGIAWAFGGMIFILVYCTAGISGGHINPAVTFGLFLGRKVSLIRAVMYMVAQCLGAICGVGLVKAFQKS
YYNLYGGGANELAPGYNKGVGLGAEIIGTFVLVYTVFSATDPKRNARDSHVPVLAPLPIGFAVFMVHLATIPITG
TGINPARSFGAAWIFWVGPFIGAAIAAFYHQYILRAAAIKALGSFRSNN*

*Oleur*PIP2;3 Oleur061Scf0690g01039.1

>Oleur061Scf0690:166917..168873 (- strand) class=gene length=1957

>*Oleur*PIP2;3a
ATGGTAAAAGATGGTGGAGTGGCTGAGCATGGATCATTCACAGTCAAAGACTACCACGATCCACCACCGGCGCCG
TTGATTGATGCGGCGGAGCTTACAAAGTGGTCATTTTACAGAGCTATTATAGCTGAGTTTATAGCCACCTTGCTT
TTCCTTTACATTACGGTTTTGACTGTGATAGGGTACAAGAGCCAGACTGATACCAACGCCGGCCACGGCGGTGAT
CAGTGCGGTGGCGTTGGAGTTCTTGGTATTGCATGGGCCTTTGGTGGCATGATCTTCGTTCTTGTCTACTGCACT
GCTGGAATTTCCGGTGAGCTTTGTTTTATATAAAATTATGGATCAGAAAACAAGTTTATATATATATTGATTAAA
TGGTTTATCATCATGTGCAGGGGGGCACATTAATCCAGCAGTGACATTCGGGCTTTTCTTGGCCCGGAAGGTGTC
GATGATCAGAGCCATTATGTATATGGTGGCTCAGTGTTTGGGGGCCATTTGTGGCGTCGGACTGGTGAAGGCATT
CCAGAAAGCATATTACAAGAAGTACGGCGGCGGCGCCAATGAACTCGCAGACGGCTACAGCACCGGCGTGGGATT
GTCGGCTGAGATCATCGGAACATTCGTTCTTGTTTACACAGTTTTCTCAGCCACCGATCCCAAGAGAAGTGCCAG
GGATTCCCATGTTCCTGTGAGTTGCACATATACAATTCCCCATCTTTTTTTCTTCTGAGAAGTGCATGTTTGGTT
AAGTTATAAGGCTTACTTATAAGGAATAAGTATTTAGAAAAATTGGATCCATGAAATAACTTAAATATTTCGAAA
ACAAAGAAAGCACCTACTAGTGATAAGTACAAGTTAAAATTACTTTAAACTTGACTTTGTTCGACTTCAAAATTG
ATCCTAAACTCTTTACTTGAGTTTGAGCCCGGCTAGAATTCAGTGGTTTTATCTTTATGAAACGATTCGATATTG
ACACTACTTGAATCTGGACTTGAATCTCAGGTTTTGGCACCTCTCCCAATTGGATTTGCAGTTTTCATGGTTCAC
TTGGCAACCATCCCAATCACTGGTACTGGCATCAACCCTGCTAGAAGTTTTGGAGCTGCAGTAATCTACAACCAG
GAAAAGGCTTGGGATGACCAGGTTAGCATTTTTGGAAAGTAATTATGAATTAGAGTTGACTATATGAATCGTGTT
TTGCACCATTTAATTCACTTTGCTGCTAATTATACTCGAAGAGCCCTCATGCAATGATTAGTAGGATTTGTGTCA
TAAGATGTGATTAAATTTCAAGTTCAATGTCAATATTCTACCTCAACCCTTCGGGTTTTTATCTAAACATGTGAC
AAGATCCCTTTTTTTTTTCTTTTTTCTTTTTTCTTTTTTTTTTTCTTTTTTTAAGTCTAAACATGAGACGAGCTA
CGAAAAAACATGAGTTACATCCCTGCATTTACATTGAAAAAACACCTACAATCACAAGTAATAATTTATGTATAC
TTTTGTCTCCTTCAGTGGATCTTCTGGGTAGGACCCTTCATTGGTGCAGCCATTGCAGCCTTCTACCACCAGTTT
ATTTGAGAGCGGGAGCAGCTAAAGCTCTTGGTTCATTCAGAAGCAGCTACCATGTT**TGA**
>*Oleur*PIP2;3a
ATGGTAAAAGATGGTGGAGTGGCTGAGCATGGATCATTCACAGTCAAAGACTACCACGATCCACCACCGGCGCCG
TTGATTGATGCGGCGGAGCTTACAAAGTGGTCATTTTACAGAGCTATTATAGCTGAGTTTATAGCCACCTTGCTT
TTCCTTTACATTACGGTTTTGACTGTGATAGGGTACAAGAGCCAGACTGATACCAACGCCGGCCACGGCGGTGAT
CAGTGCGGTGGCGTTGGAGTTCTTGGTATTGCATGGGCCTTTGGTGGCATGATCTTCGTTCTTGTCTACTGCACT
GCTGGAATTTCCGGGGGGCACATTAATCCAGCAGTGACATTCGGGCTTTTCTTGGCCCGGAAGGTGTCGATGATC
AGAGCCATTATGTATATGGTGGCTCAGTGTTTGGGGGCCATTTGTGGCGTCGGACTGGTGAAGGCATTCCAGAAA
GCATATTACAAGAAGTACGGCGGCGGCGCCAATGAACTCGCAGACGGCTACAGCACCGGCGTGGGATTGTCGGCT
GAGATCATCGGAACATTCGTTCTTGTTTACACAGTTTTCTCAGCCACCGATCCCAAGAGAAGTGCCAGGGATTCC
CATGTTCCTGTTTTGGCACCTCTCCCAATTGGATTTGCAGTTTTCATGGTTCACTTGGCAACCATCCCAATCACT
GGTACTGGCATCAACCCTGCTAGAAGTTTTGGAGCTGCAGTAATCTACAACCAGGAAAAGGCTTGGGATGACCAG
TGGATCTTCTGGGTAGGACCCTTCATTGGTGCAGCCATTGCAGCCTTCTACCACCAGTTTATTTGAGAGCGGGA
GCAGCTAAAGCTCTTGGTTCATTCAGAAGCAGCTACCATGTT**TGA**
>*Oleur*PIP2;3a
MVKDGGVAEHGSFTVKDYHDPPPAPLIDAAELTKWSFYRAIIAEFIATLLFLYITVLTVIGYKSQTDTNAGHGGD
QCGGVGVLGIAWAFGGMIFVLVYCTAGISGGHINPAVTFGLFLARKVSMIRAIMYMVAQCLGAICGVGLVKAFQK
AYYKKYGGGANELADGYSTGVGLSAEIIGTFVLVYTVFSATDPKRSARDSHVPVLAPLPIGFAVFMVHLATIPIT
GTGINPARSFGAAVIYNQEKAWDDQWIFWVGPFIGAAIAAFYHQFILRAGAAKALGSFRSSYHV*

*Oleur*PIP2;3b Oleur061Scf8232g00023.1

>Oleur061Scf8232:53550..55667 (+ strand) class=gene length=2118

>*Oleur*PIP2;3b

ATGGTAAAAGATGGTGGAGTGGCTGAGCATGGATCATTCACGGTCAAAGACTACCACGATCCACCACCGGCGCCG
TTGATTGATGCGGCAGAGCTTACAAAGTGGTCATTTTACAGAGCTGTTATAGCTGAGTTTATAGCCACCTTGCTT
TTCCTTTACATTACGGTTTTGACTGTGATAGGGTACAAGAGCCAGACTGATACCAACGCCGGCCACGGCGGTGAT
CAGTGCGGTGGTGTTGGAATTCTTGGTATTGCATGGGCCTTTGGTGGCATGATCTTCGTTCTTGTCTACTGCACT
GCTGGAATTTCCGGTGAGTTTTGTTTTATATAAAATTATGGATCAGAAAACAAGTTTATATATATATATATATAT
TGATTAAATGGTATATCATCATGTGCAGGGGGACATATTAATCCAGCAGTGACATTCGGGCTTTTCTTGGCCCGG
AAGGTGTCGATGATCAGAGCCATTATGTATATGGTGGCTCAGTGTTTGGGGGCCATTTGTGGCGTCGGACTGGTG
AAGGCATTCCAGAAAGCATATTACAAGAAGTACGGCGGCGGCGCCAATGAACTCGCAGACGGCTACAGCACTGGC
GTGGGATTGTCGGCGGAGATCATCGGAACATTCGTTCTTGTTTACACAGTTTTCTCAGCCACCGATCCCAAGAGA
AGTGCCAGGGATTCCCATGTTCCTGTGAGTTGCACATATACAATTCCCCATCTTTTTTTCTTCTGAGAAGTAAAT
GTTCGGTTAAGTTATTTAAGTATTAGAAAAATTGGATCCATGAAATAACTTAAATTTTTCGAAAACAAAGAAATC
ACTTACTAGTGATAAGCATAAGTTAAAATTACTTTAAAGTTGACTTTGTTCGACTTCAAAATTGATCCTAATACA
CTTAAGTGAGTTTGAGCCCGGCTAGAATTCAGTGGTTTTATCTTTATGAAACGATTCGATATTGACACTACTTGA
ATCTCAGGTTTTGGCACCTCTCCCAATTGGATTTGCAGTTTTCATGGTTCACTTGGCAACCATCCCAATCACTGG
TACTGGCATCAACCCTGCTAGAAGTTTTGGAGCTGCAGTAATCTACAACCAGGAAAAGGCTTGGGATGACCAGGT
TAGCATTTTTGGAAAGTAATTATGAATTAGAGTTGACTATATGAGTCGTGTTTTGCACCATTTAATTCACTTTAC
TGCTAATTATACTCGAAGAGTTCTCATGCAATGATTAGTAGGATTTGTGTCATAAGATGTGATTAAATTTCAAGT
TCAATGTCACTATTCTACCTCAACCCTTCGGGTTTTTATCTAAACATGAGTCGAGCCATGAAAAAAACACGAGTT
ACAATCCTTTTTTTTAGGGCTTCCTTTTTTTTTTTTGGATTTTGCTACTGTACATAAAAAATGTACATAATGAAT
TACAAATTATGATTTGAATGGAAATGATTGGATGAAAAAGTTGATTATATTTTAAACTAACAAATGACAGAATGC
CATGTTACATTATGTAAATCTTAATATACATAAAGTCTAAACATGAGACGAGCTACGAAAAAACATGAGTTACAT
CCCCGCATTTACATTGAAAAAACACCCACAATCACAAGCAATAATTTATGTATACTTTTGTCTCCTTCAGTGGAT
CTTCTGGGTAGGACCCTTCATTGGTGCAGCCATTGCAGCCTTCTACCACCAGTTTATTTTGAGGGCGGGAGCAGT
TAAAGCTCTTGGTTCATTCAGAAGCAGCTACCATGTTTGA

>*Oleur*PIP2;3b

ATGGTAAAAGATGGTGGAGTGGCTGAGCATGGATCATTCACGGTCAAAGACTACCACGATCCACCACCGGCGCCG
TTGATTGATGCGGCAGAGCTTACAAAGTGGTCATTTTACAGAGCTGTTATAGCTGAGTTTATAGCCACCTTGCTT
TTCCTTTACATTACGGTTTTGACTGTGATAGGGTACAAGAGCCAGACTGATACCAACGCCGGCCACGGCGGTGAT
CAGTGCGGTGGTGTTGGAATTCTTGGTATTGCATGGGCCTTTGGTGGCATGATCTTCGTTCTTGTCTACTGCACT
GCTGGAATTTCCGGGGGACATATTAATCCAGCAGTGACATTCGGGCTTTTCTTGGCCCGGAAGGTGTCGATGATC
AGAGCCATTATGTATATGGTGGCTCAGTGTTTGGGGGCCATTTGTGGCGTCGGACTGGTGAAGGCATTCCAGAAA
GCATATTACAAGAAGTACGGCGGCGGCGCCAATGAACTCGCAGACGGCTACAGCACTGGCGTGGGATTGTCGGCG
GAGATCATCGGAACATTCGTTCTTGTTTACACAGTTTTCTCAGCCACCGATCCCAAGAGAAGTGCCAGGGATTCC
CATGTTCCTGTTTTGGCACCTCTCCCAATTGGATTTGCAGTTTTCATGGTTCACTTGGCAACCATCCCAATCACT
GGTACTGGCATCAACCCTGCTAGAAGTTTTGGAGCTGCAGTAATCTACAACCAGGAAAAGGCTTGGGATGACCAG
TGGATCTTCTGGGTAGGACCCTTCATTGGTGCAGCCATTGCAGCCTTCTACCACCAGTTTATTTTGAGGGCGGGA
GCAGTTAAAGCTCTTGGTTCATTCAGAAGCAGCTACCATGTTTGA

>*Oleur*PIP2;3b

MVKDGGVAEHGSFTVKDYHDPPPAPLIDAAELTKWSFYRAVIAEFIATLLFLYITVLTVIGYKSQTDTNAGHGGD
QCGGVGILGIAWAFGGMIFVLVYCTAGISGGHINPAVTFGLFLARKVSMIRAIMYMVAQCLGAICGVGLVKAFQK
AYYKKYGGGANELADGYSTGVGLSAEIIGTFVLVYTVFSATDPKRSARDSHVPVLAPLPIGFAVFMVHLATIPIT
GTGINPARSFGAAVIYNQEKAWDDQWIFWVGPFIGAAIAAFYHQFILRAGAVKALGSFRSSYHV*

***Oleur*PIP2;4 Oleur061Scf0101g01002.1**

>Oleur061Scf0101:84671..86479 (- strand) class=gene length=1809

>*Oleur*PIP2;4

ATGGCAAAGGACGCTGGAGTGGCGGAGCATGGATCGTTCGCGGCAAAGGACTACCAAGATCCACCACCAGCTCCG
CTGATTGACGCGGAGGAGCTTACAAAGTGGTCATTTTACAGAGCTGTTATAGCCGAGTTTATAGCCACCTTGCTC
TTCCTTTACATTACGGTTTTGACTGTGATTGGGTACAAGAGCCAGATTGATACCACGGTTGGCCACGGCGGTGAT
CAGTGCGGTGGCGTTGGAATTCTTGGTATTGCATGGGCCTTTGGTGGAATGATCTTCGTTCTTGTCTACTGCACC
GCTGGAATTTCCGGTGAGTTTTGTTTATTCGCACACAGATACATGCATACATGTATGGTTTGATTTTAATGGCAT
TTCTCATGTCATGTCATGTGCATCTCAGGGGGACACATTAATCCAGCAGTGACATTCGGGCTTTTCTTGGCCCGA
AAGGTGTCAATGATCAGAGCCATTATGTATATGGTGGCTCAGTGCTTGGGGGCCATTTGTGGCGTCGCGCTGGTG
AAGGCGTTACAGAAGTCACACTATAAGAAATATGGCGGCGGCGTAAATGAGCTCGCCGATGGCTACAGCACCGGC
GTCGGATTATCCGCGGAGATAATCGGAACATTCGTCCTTGTTTACACAGTTTCTCTGCCACTGATCCCAAGAGA
AGTGCCAGGGATTCCCATGTCCCTGTAAGTTGACATGTACAATTCTCTATTTTTTTTCTTTTGAGAAGTGCATGT
TTGTTAACCTTAAAAAATTTACTTATAATTGAATAAGTATTAAAATAATTTTTTTGGGGGTCCAAGTAGAAAATA
ACTTATTTAGTAGCTTTACTGACAATATTACTTAAAAGTAATTTAATATTGACAATTTTTGCATTTGGACTTGAA
TCTTAGGTATTGGCACCTCTTCCAATTGGATTTGCAGTTTTCATGGTTCATTTGGCCACCATCCCCATCACTGGT

ACTGGTATCAACCCTGCTAGGAGTTTAGGGGCTGCAGTGATCTACAACCAGGAAAAAGCTTGGGATGATCAGGTT
AGCATTTTTCCCAATGTATTATAAATTAGATTTAACTATAGGAATAATGTTTTGCGCCATTTCATTCACTTTATT
TATTGCTAATTCTTCTCGAAGAGCCCCGATAAATTTCAAGTTCATTGTCAACATACCAGCTCTCATGTTTTTATG
AATTTTTTGAATTATGAGAGCCTAGTCAAAAAGTATCGTTCAACTCATGTTATAAGAAGTGACTTCGTGTCAACC
TCCTTTCTTTATTTGAACTTGAGACTGACTATAAACAGAAATAGCAAAAAGTTATATTTCAAAAGCTCTAAATAT
TATAGTTTTCAATTATAGCCCCACCCAAAGTCACTAGTAGTAATTTATCTGTTTACTTTCGTGTCCTTCAGTGGA
TCTTCTGGGTTGGACCCTTCTTTGGTGCAGCCATTGCAGCCTTCTACCACCAGTTTATTTTGAGAGCAGGAGCAG
CTAAAGCCCTTGGTTCATTCAGAAGCAGCTCCCATGTT**TAA**

>*Oleur*PIP2;4
ATGGCAAAGGACGCTGGAGTGGCGGAGCATGGATCGTTCGCGGCAAAGGACTACCAAGATCCACCACCAGCTCCG
CTGATTGACGCGGAGGAGCTTACAAAGTGGTCATTTTACAGAGCTGTTATAGCCGAGTTTATAGCCACCTTGCTC
TTCCTTTACATTACGGTTTTGACTGTGATTGGGTACAAGAGCCAGATTGATACCACGGTTGGCCACGGCGGTGAT
CAGTGCGGTGGCGTTGGAATTCTTGGTATTGCATGGGCCTTTGGTGGAATGATCTTCGTTCTTGTCTACTGCACC
GCTGGAATTTCCGGGGGACACATTAATCCAGCAGTGACATTCGGGCTTTTCTTGGCCCGAAAGGTGTCAATGATC
AGAGCCATTATGTATATGGTGGCTCAGTGCTTGGGGGCCATTTGTGGCGTCGCGCTGGTGAAGGCGTTACAGAAG
TCACACTATAAGAAATATGGCGGCGGCGTAAATGAGCTCGCCGATGGCTACAGCACCGGCGTCGGATTATCCGCG
GAGATAATCGGAACATTCGTCCTTGTTTACACAGTTTTCTCTGCCACTGATCCCAAGAGAATTTTCATGGTTCAT
TTGGCCACCATCCCCATCACTGGTACTGGTATCAACCCTGCTAGGAGTTTAGGGGCTGCAGTGATCTACAACCAG
GAAAAAGCTTGGGATGATCAGTGGATCTTCTGGGTTGGACCCTTCTTTGGTGCAGCCATTGCAGCCTTCTACCAC
CAGTTTATTTTGAGAGCAGGAGCAGCTAAAGCCCTTGGTTCATTCAGAAGCAGCTCCCATGTT**TAA**

>*Oleur*PIP2;4
MAKDAGVAEHGSFAAKDYQDPPPAPLIDAEELTKWSFYRAVIAEFIATLLFLYITVLTVIGYKSQIDTTVGHGGD
QCGGVGILGIAWAFGGMIFVLVYCTAGISGGHINPAVTFGLFLARKVSMIRAIMYMVAQCLGAICGVALVKALQK
SHYKKYGGGVNELADGYSTGVGLSAEIIGTFVLVYTVFSATDPKRIFMVHLATIPITGTGINPARSLGAAVIYNQ
EKAWDDQWIFWVGPFFGAAIAAFYHQFILRAGAAKALGSFRSSSHV*

***Oleur*PIP2;5 Oleur061Scf3195g08011.1 Corrected**

>Oleur061Scf3195:828028..829785 (+ strand) class=gene length=1758

>*Oleur*PIP2;5
ATGGGCAAGGACATTGAGGTTGGCGCCGAATACACCACCAAGGACTACCAAGATCCGCCACCGGCTCCGCTGATT
GACCCGGAGGAGCTAACCAAGTGGTCGTTCTACAGGGCCATTATAGCTGAATTCATCGCCACGCTCTTGTTCCTG
TACATAACTGTTCTGACTGTGATTGGGTACAAGAGCGAAAGCGCCGCCGATAAATGCGGCGGCGTCGGGATTCTT
GGCATAGCTTGGGCTTTTGGTGGCATGATTTTTGTCCTCGTTTACTGCACTGCTGGCATTTCCGGTAAGTACCTT
AATGTCTAGACTTCGACCGACTAAATTCCATTTAGAGGGAGCTAGAAATTTTGTTACATGTACGAATACTCAATA
TAATTCCAATGGGTGCAGGAGGGCACATCAACCCGGCAGTGACATTAGGCCTATTCTTGGCCCGTAAGGTGTCAC
TGGTTCGGGCTATCATGTACATGGTGGCGCAATGTTTAGGTGCCATTTGCGGTTGCGGCTTGGTAAAGGCATTCC
AGAAGTCTTACTATGTAAAATTCGGCGGCGGTGCCAATGAACTGGCGGATGGGTACAGTAACGGCACCGGATTGG
CGGCGGAGATCATTGGCACATTCGTCCTTGTTTACACCGTATTCTCTGCCACTGACCCCAAGAGAAATGCTAGAG
ACTCCCACGTCCCTGTAAGTGCATTTGACTCCCCTAGTTTCTTCCATTTTTTATACGGACTTGTTAAAATCCCGG
TTCGACTCGGCCGGGTATGAATGAAAGGCTGACTCTGACGGACCCTGTTGACACCCCTATCTAGGTCAAGAATTT
GTTTCCTCTGTTCGTGTTGATTAATCCTAATTGAAATTTTTGACAGGTTTTGGCACCTCTTCCAATTGGATTTGC
AGTGTTCATGGTTCATTTAGCCACAATCCCAATTACCGGCACGGGTATCAACCCGGCCCGAAGTTTAGGAGCTGC
TGTTATCTATGGCAAAGACAAGGCCTGGGATGATCAAGTACGCATTATTTCAATGTTTTACAATTTGTACCTTAT
TTCCAGGAAAAAAAAATTAACGTTAATTCTCATATATTTTGCAGTGGATTTTCTGGGTGGGACCATTCATTGGGG
CAGCCATTGCTGCATTTTACTACCAGTATATTCTGAGGGCAGGAGCTGTAAAAGCTCTGGGATCATTCAGGAGCA
CTGCTCAT**TGA**

>*Oleur*PIP2;5
ATGGGCAAGGACATTGAGGTTGGCGCCGAATACACCACCAAGGACTACCAAGATCCGCCACCGGCTCCGCTGATT
GACCCGGAGGAGCTAACCAAGTGGTCGTTCTACAGGGCCATTATAGCTGAATTCATCGCCACGCTCTTGTTCCTG
TACATAACTGTTCTGACTGTGATTGGGTACAAGAGCGAAAGCGCCGCCGATAAATGCGGCGGCGTCGGGATTCTT
GGCATAGCTTGGGCTTTTGGTGGCATGATTTTTGTCCTCGTTTACTGCACTGCTGGCATTTCCGGAGGGCACATC
AACCCGGCAGTGACATTAGGCCTATTCTTGGCCCGTAAGGTGTCACTGGTTCGGGCTATCATGTACATGGTGGCG
CAATGTTTAGGTGCCATTTGCGGTTGCGGCTTGGTAAAGGCATTCCAGAAGTCTTACTATGTAAAATTCGGCGGC
GGTGCCAATGAACTGGCGGATGGGTACAGTAACGGCACCGGATTGGCGGCGGAGATCATTGGCACATTCGTCCTT
GTTTACACCGTATTCTCTGCCACTGACCCCAAGAGAAATGCTAGAGACTCCCACGTCCCTGTTTTGGCACCTCTT
CCAATTGGATTTGCAGTGTTCATGGTTCATTTAGCCACAATCCCAATTACCGGCACGGGTATCAACCCGGCCCGA
AGTTTAGGAGCTGCTGTTATCTATGGCAAAGACAAGGCCTGGGATGATCAATGGATTTTCTGGGTGGGACCATTC
ATTGGGGCAGCCATTGCTGCATTTTACTACCAGTATATTCTGAGGGCAGGAGCTGTAAAAGCTCTGGGATCATTC
AGGAGCACTGCTCAT**TGA**

>*Oleur*PIP2;5

MGKDIEVGAEYTTKDYQDPPPAPLIDPEELTKWSFYRAIIAEFIATLLFLYITVLTVIGYKSESAADKCGGVGIL
GIAWAFGGMIFVLVYCTAGISGGHINPAVTLGLFLARKVSLVRAIMYMVAQCLGAICGCGLVKAFQKSYYVKFGG
GANELADGYSNGTGLAAEIIGTFVLVYTVFSATDPKRNARDSHVPVLAPLPIGFAVFMVHLATIPITGTGINPAR
SLGAAVIYGKDKAWDDQWIFWVGPFIGAAIAAFYYQYILRAGAVKALGSFRSTAH*

***Oleur*PIP2;6a Oleur061Scf3195g08011.1**

>Oleur061Scf0106:611096..613917 (- strand) class=gene length=2822

>*Oleur*PIP2;6a
ATGACAAAGGACGCAGGTGTGGCGGAGCACGGGTCGTTCACGACCAAGGACTACCATGATCCGCCTCCGGCGCCA
CTCATCGACGCGGAGGAGCTGACAAAATGGTCATTTTACAGGGCTATAATAGCTGAGTTCATAGCCACTTTGCTG
TTTCTTTACATTACAGTTTTGACTGTAATTGGGTACAAGAGCCAGACTGATCCCGACTTGAAGGGTGACCAGTGT
GATGGGGTTGGAATTCTTGGTATTGCATGGGCCTTTGGTGGCATGATCTTCATTCTGGTTTACTGCACTGCTGGA
ATTTCTGGTCAGTTTTATTTTGTTGAGACTCGAGATTCCATCTTAAGAGTTAATTTTTTTGTTACTATTTTCATA
TGTAACCATTTTGATTTACCCTTTAACATGATCAGCAAGATGGTGGCTTATGTGCCACAATTAATCTTGATATCT
GACATGATAAAGTTACATGCTCAATTAATTTGTCCAATAAAAATGGTGTAATTTGAGTGATCTGTGTGCATGCAG
GGGGGCACATAAACCCAGCAGTGACCTTTGGCCTGTTCTTGGCCCGGAAAGTGTCTTTGATCCGAGCAGTGATGT
ACATGGTGGCACAGTGTTTGGGAGCCATCTGCGGCGTTGGTTTGGTTAAGGCATTTCAGAAATCATACTACAAGA
AATACGGTGGCGGTGCCAACGTGCTGGCCGATGGGATCAGCACCGGCGTCGGGTTAGCTGCCGAAATCATCGGAA
CATTCGTCCTTGTTTACACTGTTTTCTCCGCCACTGATCCAAAGCGTAGTGCCAGAGATTCTCACGTCCCTGTAA
GTTTCTATGAGCTCAATTTTTCCTCAAGTGATTAAGCCTTGGAATATGTGATTAAAAATTAATTGTTGACTTAAC
TTAAAATCTAACTTAATATAGATTTAAATTCCCTTTGAGTTTATTATAATTAGATGAGTAAAGATGTATCCTTTT
TTGCTGTCTCGATTCAAGTTCGGTAAAGATTAGTAGTAGGGGTGAGCAGGGGCAGACCTACTTGAGACCCAGCGG
GTCGGCAGCCCCCACTCGATTTTCAGATTTTTAGTTTTTCTCCAAGTTTTTGTAGGTTTTCAATTTTTTTCCCAC
GGTGTTTTGCTTTTGTCCCCCACTGCACTTCCGTCCTGAGTCCACCATTGAATGTGAGCCATACCAAATTGCAAT
ATGCTGTACAACTGGGGCACTTGCCAGTATTATGTGGTTGTAAGTGAAATGAAGAAATTAATGTACCAAAGGGGA
CTTTACCTTGGTGACAGATGCAATGAATCAAGAAAAGTACAAGAGATATTATAATATTGGTTTAACTAATTCGTT
ATGGACCATGAAACTTTGGATTATACGACTTTGTGAGAACGATTTTGACCGATGCAGTGAAAGAAGTATTGGTTA
CGGCAGATGATTTGATGAGAAAGTTGATCGTATTTTAAAAAATTTAATGATAGAAAATTATATGGTATTATGTCA
ACCTGTCAACATATCTAGCACTATAACACCATTGTAAAATCTTACCTTGGGGACATGGATTCGTAGATTAGATAG
CAATATTAGTAGAAAACTTTTGTGGGCCCTATTAGAACTAAAAAAAATTCCACGCTTGTGGGGGATATCGTGTGA
TATACAACAGTAGACAATGATACATGAATTTTGCTAAACGACCTGCACCGTTCACTACACCGATGATTGCACCCT
CACATGATGCATTGGTCCCACCTGAATAAAAAAATGTCGACGCCATGTGTATGTGTGTGTGTGGTCACTTGCGCG
GGTCGTGTAGCGTGGTCCATGATACATTAGCGGATAAATTTTTTTACCAAAAAAAAAAAGAAAAAAAAAAATTTCT
TCTATAGTGATGTCCATTAATGTATTTAAATTCAACATACTAATTATGCCGAAGCCATTATTATTTCGAAAATAC
TAGGTATTGGCACCTCTTCCAATTGGATTTGCAGTGTTCATGGTTCATTTGGCCACCATTCCAATCACCGGAACG
GGCATCAACCCAGCTCGGAGTCTCGGAGCCGCAGTAATCTACGACCAGCACGAGGCTTGGGACGATCACGTTAGT
ACATATATTCCAAATTGAATTTAATTACTTTTCATAAAACGTATACTATAACGAAAATTTCTGTCTTGAAAAGGT
CTTTTGATGACCCATAATTGGTGCTATTTAATCTAATAATTACATGTATATGTTTTTTCAGTGGATTTTCTGGGT
CGGACCGTTTATCGGTGCAGCCGTTGCAGCATTCTACCACCAGTTTATATTGAGAGCTGGAGCAGCAAAGGCCCT
TGGTTCATTCAGGAGTAGTGCTTCTCATCATTGA

>*Oleur*PIP2;6a
ATGACAAAGGACGCAGGTGTGGCGGAGCACGGGTCGTTCACGACCAAGGACTACCATGATCCGCCTCCGGCGCCA
CTCATCGACGCGGAGGAGCTGACAAAATGGTCATTTTACAGGGCTATAATAGCTGAGTTCATAGCCACTTTGCTG
TTTCTTTACATTACAGTTTTGACTGTAATTGGGTACAAGAGCCAGACTGATCCCGACTTGAAGGGTGACCAGTGT
GATGGGGTTGGAATTCTTGGTATTGCATGGGCCTTTGGTGGCATGATCTTCATTCTGGTTTACTGCACTGCTGGA
ATTTCTGGGGGGCACATAAACCCAGCAGTGACCTTTGGCCTGTTCTTGGCCCGGAAAGTGTCTTTGATCCGAGCA
GTGATGTACATGGTGGCACAGTGTTTGGGAGCCATCTGCGGCGTTGGTTTGGTTAAGGCATTTCAGAAATCATAC
TACAAGAAATACGGTGGCGGTGCCAACGTGCTGGCCGATGGGATCAGCACCGGCGTCGGGTTAGCTGCCGAAATC
ATCGGAACATTCGTCCTTGTTTACACTGTTTTCTCCGCCACTGATCCAAAGCGTAGTGCCAGAGATTCTCACGTC
CCTGTATTGGCACCTCTTCCAATTGGATTTGCAGTGTTCATGGTTCATTTGGCCACCATTCCAATCACCGGAACG
GGCATCAACCCAGCTCGGAGTCTCGGAGCCGCAGTAATCTACGACCAGCACGAGGCTTGGGACGATCACTGGATT
TTCTGGGTCGGACCGTTTATCGGTGCAGCCGTTGCAGCATTCTACCACCAGTTTATATTGAGAGCTGGAGCAGCA
AAGGCCCTTGGTTCATTCAGGAGTAGTGCTTCTCATCATTGA

>*Oleur*PIP2;6a
MTKDAGVAEHGSFTTKDYHDPPPAPLIDAEELTKWSFYRAIIAEFIATLLFLYITVLTVIGYKSQTDPDLKGDQC
DGVGILGIAWAFGGMIFILVYCTAGISGGHINPAVTFGLFLARKVSLIRAVMYMVAQCLGAICGVGLVKAFQKSY
YKKYGGGANVLADGISTGVGLAAEIIGTFVLVYTVFSATDPKRSARDSHVPVLAPLPIGFAVFMVHLATIPITGT
GINPARSLGAAVIYDQHEAWDDHWIFWVGPFIGAAVAAFYHQFILRAGAAKALGSFRSSASHH*

*Oleur*PIP2;6b Oleur061Scf0638g02012.1

>Oleur061Scf0638:213705..216526 (- strand) class=gene length=2822

>*Oleur*PIP2;6b
ATGACAAAGGACGCAGGTGTGGCGGAGCACGGGTCGTTCACGACCAAGGACTACCATGATCCGCCTCCGGCGCCG
CTCATCGACGCGGAGGAGCTGACAAAATGGTCATTTTACAGGGCTATAATAGCTGAGTTCATAGCCACTTTGCTG
TTTCTTTACATTACAGTTTTGACTGTAATTGGGTACAAGAGCCAGACTGATCCCGACTTGAAGGGTGACCAGTGT
GATGGGGTTGGAATTCTTGGTATTGCATGGGCCTTTGGTGGCATGATCTTCATTCTGGTTTACTGCACTGCTGGA
ATTTCTGGTCAGTTTTATTTTGTTGAGACTCGAGATTCCATCTTAAGAGTTAATTTTTTTGTTACTATTTTCATA
TGTAACCATTTTGATTTACCCTTTAACATGATCAGCAAGATGGTGGCTTATGTGCCACAATTAATCTTGATATCT
GACATGATAAAGTTACATGCTCAATTAATTTTTCCAATAAAAATGGTGTAATTTGAGTGATCTGTGTGCATGCAG
GGGGGCACATAAACCCAGCAGTGACCTTTGGCCTGTTCTTGGCCCGGAAAGTGTCTTTGATCCGGGCAGTGATGT
ACATGGTGGCACAGTGTTTGGGAGCCATCTGCGGCGTTGGTTTGGTCAAGGCATTTCAGAAATCATACTACAAGA
AATACGGTGGCGGCGCCAACGTGCTGGCCGATGGGATCAGCACCGGCGTCGGGTTAGCTGCCGAAATCATCGGAA
CATTCGTCCTTGTTTACACTGTTTTCTCCGCCACTGATCCAAAGCGTAGTGCCAGAGATTCTCACGTCCCTGTAA
GTTTCTATGAGCTCAATTTTTGCTCAAGTGATTAAGCCTGATTAAGTTTGGAATATTGCGAATTTCATGAAATAT
GTGATTAAAAATTAATTGTTGACTTAACTTAAAATCTAACTTAATATAGATTTAAATGCCCTTTTGAGTTTATTA
TAATTAGATAAGTAAAGATGTATCTTTTTTTGCTGTCTCGATTCAAGTTCGGTAAAGATTAGTAGTAAGGGTGAG
CAGGGGCAGACCTACTTGAGGACCAGCGGGTCGGCTGCGCCCCCTCGATTTTCAGATTTTTAGTTTTTCTCCAAA
TTTTTTTAGGTTTTCAGATTTTTTTTCCACGGTGTTTTGGTTTTGTCCCCCACTGCACTTCCGTCCTGAGTCCAC
CATTGAATGTGACCCATACCAAATTGCAATATGCTGTACAACTGGGGCACTTGCCAGCATTATGTGGTTGTAAGT
GAAATGAAGAAATTAATGTACCAAAGGGGACTTTACCTTGGTGACAGATGCAATGAATCAAGAAAAGTACAAGAG
ATATTATAATATTGGTTTAACTAATTCGTTATGGACCATGAAACTTTGGATTATACCACTTTGTGAGAATGATTT
TGACCGATGCAGTGAAAGAAGTATTGGTGACGGCAGATGATTTGATGAGAAAATTGATCGTATTTTAAACAATTT
AATGATAGAAAATTATATGGTATTATGTCAACCTGTCAACATATCTAGCACTATAACACCATTGTAAAATCTTAC
CTTGGGGACATGGATTCGTAGATTAGATAGCAATATTAGTAGAAAACTTTTGTGGGCCCTATTAGAACTAAAAAA
AATTCCACGCTTGTGGGGGATATCGTGTGATATACAACAGTAGACAATGATACGTGAATTTTGCTAAACGACCCG
CACCGTTCACATGATGCAGGGGTCTCACTTGAATAAAAAAATGTCGACGCCATGTGTATGTGTGTGTGCGGTCAC
CTGCGCGGGTCGTGCAGTGTGATCCATGATACATTGGCGGATAAATTTTTTTACCAAAAAAAAAAATTAAAATTC
TTCTGTAGTGATGTCCATTAATGTATTTAAATTCAACATACTAATTATGCCGAAGCCATTATTATTTCGAAAATA
CTAGGTATTGGCACCTCTTCCAATTGGATTTGCAGTGTTCATGGTTCATTTGGCTACCATTCCAATCACCGGAAC
GGGCATCAACCCAGCTCGGAGTCTTGGAGCCGCAGTAATCTACGACCAGCACAAGGCTTGGGACGATCACGTTAG
TACATATATTTCAAATTAAATTTAGTTATTCCCATTAAACGTATACTATAACGAAAATTTCTGTCTTGAAAAGGT
CTTTGATGACCCATAATTGGTGCTATTTAATCTAATAATTACATGTATATGTTTTTTCAGTGGATTTTCTGGGT
CGGACCGTTTATCGGTGCAGCCGTTGCAGCATTCTACCACCAGTTTATATTGAGAGCTGGAGCAGCAAAGGCCCT
TGGTTCATTCAGGAGTAGTGCTTCTCATCAT**TGA**

>*Oleur*PIP2;6b
ATGACAAAGGACGCAGGTGTGGCGGAGCACGGGTCGTTCACGACCAAGGACTACCATGATCCGCCTCCGGCGCCG
CTCATCGACGCGGAGGAGCTGACAAAATGGTCATTTTACAGGGCTATAATAGCTGAGTTCATAGCCACTTTGCTG
TTTCTTTACATTACAGTTTTGACTGTAATTGGGTACAAGAGCCAGACTGATCCCGACTTGAAGGGTGACCAGTGT
GATGGGGTTGGAATTCTTGGTATTGCATGGGCCTTTGGTGGCATGATCTTCATTCTGGTTTACTGCACTGCTGGA
ATTTCTGGGGGGCACATAAACCCAGCAGTGACCTTTGGCCTGTTCTTGGCCCGGAAAGTGTCTTTGATCCGGGCA
GTGATGTACATGGTGGCACAGTGTTTGGGAGCCATCTGCGGCGTTGGTTTGGTCAAGGCATTTCAGAAATCATAC
TACAAGAAATACGGTGGCGGCGCCAACGTGCTGGCCGATGGGATCAGCACCGGCGTCGGGTTAGCTGCCGAAATC
ATCGGAACATTCGTCCTTGTTTACACTGTTTTCTCCGCCACTGATCCAAAGCGTAGTGCCAGAGATTCTCACGTA
TTGGCACCTCTTCCAATTGGATTTGCAGTGTTCATGGTTCATTTGGCTACCATTCCAATCACCGGAACGGGCATC
AACCCAGCTCGGAGTCTTGGAGCCGCAGTAATCTACGACCAGCACAAGGCTTGGGACGATCACTGGATTTTCTGG
GTCGGACCGTTTATCGGTGCAGCCGTTGCAGCATTCTACCACCAGTTTATATTGAGAGCTGGAGCAGCAAAGGCC
CTTGGTTCATTCAGGAGTAGTGCTTCTCATCAT**TGA**

>*Oleur*PIP2;6b
MTKDAGVAEHGSFTTKDYHDPPPAPLIDAEELTKWSFYRAIIAEFIATLLFLYITVLTVIGYKSQTDPDLKGDQC
DGVGILGIAWAFGGMIFILVYCTAGISGGHINPAVTFGLFLARKVSLIRAVMYMVAQCLGAICGVGLVKAFQKSY
YKKYGGGANVLADGISTGVGLAAEIIGTFVLVYTVFSATDPKRSARDSHVLAPLPIGFAVFMVHLATIPITGTGI
NPARSLGAAVIYDQHKAWDDHWIFWVGPFIGAAVAAFYHQFILRAGAAKALGSFRSSASHH*

***Oleur*PIP2;7 Oleur061Scf6451g01042.1**

>Oleur061Scf6451:160614..162280 (+ strand) class=gene length=1667

>*Oleur*PIP2;7
ATGGTGAAGGATGTTGAGGTCGGAGGCCCTGAATTTACTACAAAAGACTATCAAGACCCTCCTCCAGCACCGTTG
ATTGATCCGGAAGAACTGACTAAATGGTCATTTTATAGAGCTATCATTGCTGAATTCGTAGCTACATTACTGTTC
TTATACATTACTGTTTTGACTGTGATTGGGTACAAGAGCCAGAGCGCCACCGATGAATGCGGCGGCGTTGGCATT
TTGGGCATTGCTTGGGCCTTTGGTGGCATGATTTTCGTACTTGTTTACTGCACTGCTGGTATTTCCGGTGAGTTG
CACATCTTAAATATGATTCAAATCATAAAATTGACAAAGTTTTAACACAGATTCCTCTTTCTTTGTGATGATATT
TACGACGTTAATGGTGCAGGAGGGCACATTAACCCGGCAGTGACATTCGGGTTGTTTTTAGCCCGAAAAGTTTCA
TTGATTCGGGCCATTATGTACATGGTGGCTCAGTGTGTAGGGGCCATTTGTGGGTGTGGATTGGTGAAGGCATTC
CAGAGGGCTTACTACGTTAGATACGGTGGTGGCGCCAATGAACTTGGAGATGGATATAGCACAGGCACCGGATTG
GCGGCCGAGATTATCGGCACTTTTGTGCTTGTTTACACTGTCTTCTCCGCCACGGACCCTAAAAGAAGTGCCAGA
GACTCCCATGTTCCGGTAATATCCGTGCTTAAGTTTTTAGCCAAACAAACTCCATAATTTTAGCAAGTCAAGATT
TTCTTCAATCAATTTTGGCTGTTGATTAATCCTACTTGAAAATTAATCTAAAGGTATTGGCACCACTGCCTATTG
GATTTGCTGTGTTCATGGTTCATTTGGCTACAATTCCGATCACCGGCACTGGTATCAACCCTGCCCGAAGTTTTG
GAGCTGCAGTGATCTATGGCAAGGATAAGGCCTGGGACGATCAAGTATTGAACATTATTTAAAAATTTGTTTATT
TTATTGTTAAGTGTTGTCGAGTACGGCTGCTGACTGACCCTTTTGATCGATTTTTGACAGTGGATCTTCTGGGCT
GGACCTTTCATTGGGGCAGCCATTGCTGCATTGTACCATCAGTACATTCTTAGAGCAGGTGCAGTTAAAGCTCTA
GGATCATTCAGGAGTAGTGCTTCTGCCTATTGA
>*Oleur*PIP2;7
ATGGTGAAGGATGTTGAGGTCGGAGGCCCTGAATTTACTACAAAAGACTATCAAGACCCTCCTCCAGCACCGTTG
ATTGATCCGGAAGAACTGACTAAATGGTCATTTTATAGAGCTATCATTGCTGAATTCGTAGCTACATTACTGTTC
TTATACATTACTGTTTTGACTGTGATTGGGTACAAGAGCCAGAGCGCCACCGATGAATGCGGCGGCGTTGGCATT
TTGGGCATTGCTTGGGCCTTTGGTGGCATGATTTTCGTACTTGTTTACTGCACTGCTGGTATTTCCGGAGGGCAC
ATTAACCCGGCAGTGACATTCGGGTTGTTTTTAGCCCGAAAAGTTTCATTGATTCGGGCCATTATGTACATGGTG
GCTCAGTGTGTAGGGGCCATTTGTGGGTGTGGATTGGTGAAGGCATTCCAGAGGGCTTACTACGTTAGATACGGT
GGTGGCGCCAATGAACTTGGAGATGGATATAGCACAGGCACCGGATTGGCGGCCGAGATTATCGGCACTTTTGTG
CTTGTTTACACTGTCTTCTCCGCCACGGACCCTAAAAGAAGTGCCAGAGACTCCCATGTTCCGGTATTGGCACCA
CTGCCTATTGGATTTGCTGTGTTCATGGTTCATTTGGCTACAATTCCGATCACCGGCACTGGTATCAACCCTGCC
CGAAGTTTTGGAGCTGCAGTGATCTATGGCAAGGATAAGGCCTGGGACGATCAATGGATCTTCTGGGCTGGACCT
TTCATTGGGGCAGCCATTGCTGCATTGTACCATCAGTACATTCTTAGAGCAGGTGCAGTTAAAGCTCTAGGATCA
TTCAGGAGTAGTGCTTCTGCCTATTGA
>*Oleur*PIP2;7
MVKDVEVGGPEFTTKDYQDPPPAPLIDPEELTKWSFYRAIIAEFVATLLFLYITVLTVIGYKSQSATDECGGVGI
LGIAWAFGGMIFVLVYCTAGISGGHINPAVTFGLFLARKVSLIRAIMYMVAQCVGAICGCGLVKAFQRAYYVRYG
GGANELGDGYSTGTGLAAEIIGTFVLVYTVFSATDPKRSARDSHVPVLAPLPIGFAVFMVHLATIPITGTGINPA
RSFGAAVIYGKDKAWDDQWIFWAGPFIGAAIAALYHQYILRAGAVKALGSFRSSASAY*

***Oleur*PIP2;8a Oleur061Scf3978g00008.1**

>Oleur061Scf3978:91118..92620 (- strand) class=gene length=1503

>*Oleur*PIP2;8a
ATGTCGAAAGAAGTGACTGAAGAAGGACAAGTACACCCCCATGGAAAGGACTATGAGGACCCACCGCCAGCACCA
TTACTGGACATGGGTGAGCTCATGCTCTGGTCCTTTTACAGAGCCATAATTGCCGAGTTCATTGCCACCCTGCTC
TTCCTTTACGTCACCGTCGCCACCGTCATCGGCCACAAGAAGCTCAACGCCGCCGACCAATGCGATGGCGTTGGC
ATCCTCGGCATCGCTTGGGCCTTTGGTGGCATGATCTTCATCCTTGTTTACTGCACCGCCGGCATCTCAGGTACA
TGATTCACTGCCTCAACTTCTTAAAGACAAATCACCATTTATTTCTTGAAATTTTACCTGAATTTTTTCTCTGCT
GTTCTACAGGTGGTCACATAAACCCAGCTGTGACTTTCGGGTTGTTCTTGGCCAGGAAGGTGTCGTTGATCCGTG
CAATTATGTACATGATTGCACAGTGTTTGGGTGCCGTCTGTGGAGTTGGCCTGGTGAAGGCCTTCATGAAGAGCT
ACTACAACCGTCTAGGCGGCGGCGCTAACGTGGTGTCTCATGGGTACAATATCGGCACAGCTTTGGGTGCTGAAA
TTATCGGTACCTTTGTTCTTGTATACACCGTCTTCTCGGCCACTGACCCCAAGAGAAGTGCACGTGACTCTCACG
TCCCTGTAAGTAGAAAAAAAATGGCTAGCTTATTTGTGGGTTTTTTCCTTTTCTTTTCTAAGTTAACTTATCATT
TGGATTATTTTAGGTTTTGGCTCCTCTTCCAATTGGGTTTGCAGTTTTCATGGTGCACTTAGCGACCATCCCCAT
CACTGGAACTGGTATCAACCCTGCTAGGAGTTTTGGAGCTGCTGTGATCTACAACAATGACAAAGTCTGGGATGA
CCATGTAAGTTGGTTTGGATAAATTACTTTCTTTTTTTTTTTTTCGTTTCTTACTTTTGTTGAACTTACAAGTA

AAAAAATTTGGATGGTTCATACAGTGGATTTTCTGGGTTGGACCATTTGTGGGAGCTTTGGCGGCAGCAGCATAT
CATCAGTACATCTTGAGAGCTGCAGCTATTAAGGCAATTGGATCATTCCGCAGCAATCCCACAAAC**TAA**

>*Oleur*PIP2;8a
ATGTCGAAAGAAGTGACTGAAGAAGGACAAGTACACCCCCATGGAAAGGACTATGAGGACCCACCGCCAGCACCA
TTACTGGACATGGGTGAGCTCATGCTCTGGTCCTTTTACAGAGCCATAATTGCCGAGTTCATTGCCACCCTGCTC
TTCCTTTACGTCACCGTCGCCACCGTCATCGGCCACAAGAAGCTCAACGCCGCCGACCAATGCGATGGCGTTGGC
ATCCTCGGCATCGCTTGGGCCTTTGGTGGCATGATCTTCATCCTTGTTTACTGCACCGCCGGCATCTCAGGTGGT
CACATAAACCCAGCTGTGACTTTCGGGTTGTTCTTGGCCAGGAAGGTGTCGTTGATCCGTGCAATTATGTACATG
ATTGCACAGTGTTTGGGTGCCGTCTGTGGAGTTGGCCTGGTGAAGGCCTTCATGAAGAGCTACTACAACCGTCTA
GGCGGCGGCGCTAACGTGGTGTCTCATGGGTACAATATCGGCACAGCTTTGGGTGCTGAAATTATCGGTACCTTT
GTTCTTGTATACACCGTCTTCTCGGCCACTGACCCCAAGAGAAGTGCACGTGACTCTCACGTCCCTGTTTTGGCT
CCTCTTCCAATTGGGTTTGCAGTTTTCATGGTGCACTTAGCGACCATCCCCATCACTGGAACTGGTATCAACCCT
GCTAGGAGTTTTGGAGCTGCTGTGATCTACAACAATGACAAAGTCTGGGATGACCATTGGATTTTCTGGGTTGGA
CCATTTGTGGGAGCTTTGGCGGCAGCAGCATATCATCAGTACATCTTGAGAGCTGCAGCTATTAAGGCAATTGGA
TCATTCCGCAGCAATCCCACAAAC**TAA**
>*Oleur*PIP2;8a
MSKEVTEEGQVHPHGKDYEDPPPAPLLDMGELMLWSFYRAIIAEFIATLLFLYVTVATVIGHKKLNAADQCDGVG
ILGIAWAFGGMIFILVYCTAGISGGHINPAVTFGLFLARKVSLIRAIMYMIAQCLGAVCGVGLVKAFMKSYYNRL
GGGANVVSHGYNIGTALGAEIIGTFVLVYTVFSATDPKRSARDSHVPVLAPLPIGFAVFMVHLATIPITGTGINP
ARSFGAAVIYNNDKVWDDHWIFWVGPFVGALAAAAYHQYILRAAAIKAIGSFRSNPTN*

*Oleur*PIP2;8b Oleur061Scf0410g01025.1

>Oleur061Scf0410:167787..169203 (- strand) class=gene length=1417

>*Oleur*PIP2;8b
ATGTCGAAAGAAGTGATTGAAGAAGGACAAGTACACCCCCATGGAAAGGACTATGAGGACCCACCGCCAGCACCA
TTACTGGACATGGGTGAGCTCATGCTCTGGTCCTTTTATAGAGCCATAATTGCCGAGTTCATTGCCACCCTGCTC
TTCCTTTACGTCACCGTCGCCACCGTCATCGGCCACAAGAAGCTCAACGCCGCCGACCAATGCGATGGCGTTGGC
ATCCTCGGCATCGCTTGGGCCTTTGGTGGCATGATCTTCATCCTTGTTTACTGCACCGCCGGCATCTCAGGTACA
TGATTCACTGCCTCAACTTCTTCAAGACAAATCACCATTTATTTCTTGAAATTTTACCTGAATTTTTTCTCTGCT
GTTCTACAGGTGGTCACATAAACCCAGCTGTTACTTTCGGGTTGTTCTTGGCCAGGAAGGTGTCGTTGATCCGTG
CAATTATGTACATGATTGCACAGTGTTTGGGTGCCGTCTGTGGAGTTGGCCTGGTGAAGGCCTTCATGAAGAGTT
ACTACAACCGTCTAGGCGGCGGCGCTAACTTGGTGTCTCATGGGTACAATATCGGCACAGCTTTGGGTGCTGAAA
TTATCGGTACCTTTGTTCTTGTATACACCGTCTTCTCGGCCACTGACCCCAAGAGAAGTGCACGTGACTCTCACG
TCCCTGTAAGTAGAAAAAAAATAGCTAGCTTATTTGTGGGTTTTTCCTTTTCTTTTCTAAGTTAACTTATTTTT
TGGATTATTTTAGGTTTTGGCTCCTCTTCCAATTGGGTTTGCAGTTTTCATGGTGCACTTAGCGACCATCCCCAT
CACTGGAACTGGTATCAACCCTGCTAGGAGTTTTGGAGCTGCTGTGATCTACAACAATGACAAAGTCTGGGATGA
CCATGTAAGTTGGTTTGGATAAATTACTTTCTTTTTATTTTTTCGTTTCTTACTTTTGTTGAACTTACAAGTAAA
AAAATTTGGATGGTTCATACAGTGGATTTTCTGGGTAGGACCATTTGTGGGAGCTTTGGCGGCAGCAGCATATCA
TCAGTACATCTTGAGAGCTGCAGCTATTAAGGCAATTGGATCATTCCGCAGCAATCCCACAAAC**TAA**
>*Oleur*PIP2;8b
ATGTCGAAAGAAGTGATTGAAGAAGGACAAGTACACCCCCATGGAAAGGACTATGAGGACCCACCGCCAGCACCA
TTACTGGACATGGGTGAGCTCATGCTCTGGTCCTTTTATAGAGCCATAATTGCCGAGTTCATTGCCACCCTGCTC
TTCCTTTACGTCACCGTCGCCACCGTCATCGGCCACAAGAAGCTCAACGCCGCCGACCAATGCGATGGCGTTGGC
ATCCTCGGCATCGCTTGGGCCTTTGGTGGCATGATCTTCATCCTTGTTTACTGCACCGCCGGCATCTCAGGTGGT
CACATAAACCCAGCTGTTACTTTCGGGTTGTTCTTGGCCAGGAAGGTGTCGTTGATCCGTGCAATTATGTACATG
ATTGCACAGTGTTTGGGTGCCGTCTGTGGAGTTGGCCTGGTGAAGGCCTTCATGAAGAGTTACTACAACCGTCTA
GGCGGCGGCGCTAACTTGGTGTCTCATGGGTACAATATCGGCACAGCTTTGGGTGCTGAAATTATCGGTACCTTT
GTTCTTGTATACACCGTCTTCTCGGCCACTGACCCCAAGAGAAGTGCACGTGACTCTCACGTCCCTGTTTTGGCT
CCTCTTCCAATTGGGTTTGCAGTTTTCATGGTGCACTTAGCGACCATCCCCATCACTGGAACTGGTATCAACCCT
GCTAGGAGTTTTGGAGCTGCTGTGATCTACAACAATGACAAAGTCTGGGATGACCATTGGATTTTCTGGGTAGGA
CCATTTGTGGGAGCTTTGGCGGCAGCAGCATATCATCAGTACATCTTGAGAGCTGCAGCTATTAAGGCAATTGGA
TCATTCCGCAGCAATCCCACAAAC**TAA**
>*Oleur*PIP2;8b
MSKEVIEEGQVHPHGKDYEDPPPAPLLDMGELMLWSFYRAIIAEFIATLLFLYVTVATVIGHKKLNAADQCDGVG
ILGIAWAFGGMIFILVYCTAGISGGHINPAVTFGLFLARKVSLIRAIMYMIAQCLGAVCGVGLVKAFMKSYYNRL
GGGANLVSHGYNIGTALGAEIIGTFVLVYTVFSATDPKRSARDSHVPVLAPLPIGFAVFMVHLATIPITGTGINP
ARSFGAAVIYNNDKVWDDHWIFWVGPFVGALAAAAYHQYILRAAAIKAIGSFRSNPTN*

*Oleur*PIP2;9a Oleur061Scf0410g01025.1

>Oleur061Scf7760:51176..52742 (- strand) class=gene length=1567

>*Oleur*PIP2;9a
ATGACGAAGGAAGTGAGTGAAGAAGGACAGGTACATCACCATGGAAAGGACTACGTGGACCCACCACCAGCACCA
TTGCTAGACATGGGTGAGCTCAAGCTCTGGTCCTTTTACAGAGCTCTTATCGCCGAGTTCATCGCCACCCTTCTC
TTCCTCTACGTCACCGTCGCTACCGTCATCGGCCACAAGAAGCTCAACGCGGCCGACCAATGTGATGGCGTTGGC
ATTCTCGGTATCGCCTGGGCCTTTGGTGGCATGATCTTCATCCTCGTTTACTGTACCGCCGGCATCTCAGGTACT
CAATTCTTTAGGACAAATTCTTCTCCAGAAGCACTACCTATTCCTTGAAATTTCCCTGAATTTCTCTCTATTTGC
GCTGCAGGTGGTCACATAAACCCGGCTGTGACTTTTGGTTTGTTCTTGGCAAGGAAGGTATCGTTGATCCGTGCT
GTTATGTACATGATTGCACAGTGTTTGGGTGCCATTTGTGGAGTTGGGCTGGTGAAGGCCTTCATGAAGGGGTAC
TACAACCGTCTAGGCGGCGGCGCTAACTCGGTGTCTCATGGTTACAACACAGGCACAGCTTTGGGTGCTGAAATC
ATCGGCACCTTTGTGCTTGTTTACACCGTCTTCTCCGCCACTGACCCCAAGAGAAGTGCACGTGACTCTCATGTC
CCTGTAAGTAGAAGCAATTTCTAGCTTATTTTGTGTTTGCTTTTTCTAAAGTGAACTTGAACTTATTTTGGATTA
TTTTCAGGTTTTGGCTCCTTTACCAATTGGATTTGCAGTGTTCGTGGTGCACTTGGCCACCATCCCTATTACTGG
AACTGGTATCAACCCTGCTAGGAGTTTTGGAGCTGCCGTCATCTTCAACAATGATAAAGTCTGGGATGACCATGT
AAGTTCGTCTAGTAAGTTACTTTTTTTCAAGTATATATAATTTATTTTCTTGAACTAATAAGTATAATTTTGAT
GATGCATGTAGTGGATTTTCTGGGTTGGACCATTTGTGGGAGCATTGGCAGCAGCAGCATATCATCAGTACATCT
TAAGGGCTGCAGCTATTAAGGCATTGGGATCATTCCGCAGCAACCCCACAAACTAA

>*Oleur*PIP2;9a
ATGACGAAGGAAGTGAGTGAAGAAGGACAGGTACATCACCATGGAAAGGACTACGTGGACCCACCACCAGCACCA
TTGCTAGACATGGGTGAGCTCAAGCTCTGGTCCTTTTACAGAGCTCTTATCGCCGAGTTCATCGCCACCCTTCTC
TTCCTCTACGTCACCGTCGCTACCGTCATCGGCCACAAGAAGCTCAACGCGGCCGACCAATGTGATGGCGTTGGC
ATTCTCGGTATCGCCTGGGCCTTTGGTGGCATGATCTTCATCCTCGTTTACTGTACCGCCGGCATCTCAGGTGGT
CACATAAACCCGGCTGTGACTTTTGGTTTGTTCTTGGCAAGGAAGGTATCGTTGATCCGTGCTGTTATGTACATG
ATTGCACAGTGTTTGGGTGCCATTTGTGGAGTTGGGCTGGTGAAGGCCTTCATGAAGGGGTACTACAACCGTCTA
GGCGGCGGCGCTAACTCGGTGTCTCATGGTTACAACACAGGCACAGCTTTGGGTGCTGAAATCATCGGCACCTTT
GTGCTTGTTTACACCGTCTTCTCCGCCACTGACCCCAAGAGAAGTGCACGTGACTCTCATGTCCCTGTTTTGGCT
CCTTTACCAATTGGATTTGCAGTGTTCGTGGTGCACTTGGCCACCATCCCTATTACTGGAACTGGTATCAACCCT
GCTAGGAGTTTTGGAGCTGCCGTCATCTTCAACAATGATAAAGTCTGGGATGACCATTGGATTTTCTGGGTTGGA
CCATTTGTGGGAGCATTGGCAGCAGCAGCATATCATCAGTACATCTTAAGGGCTGCAGCTATTAAGGCATTGGGA
TCATTCCGCAGCAACCCCACAAACTAA

>*Oleur*PIP2;9a
MTKEVSEEGQVHHHGKDYVDPPPAPLLDMGELKLWSFYRALIAEFIATLLFLYVTVATVIGHKKLNAADQCDGVG
ILGIAWAFGGMIFILVYCTAGISGGHINPAVTFGLFLARKVSLIRAVMYMIAQCLGAICGVGLVKAFMKGYYNRL
GGGANSVSHGYNTGTALGAEIIGTFVLVYTVFSATDPKRSARDSHVPVLAPLPIGFAVFVVHLATIPITGTGINP
ARSFGAAVIFNNDKVWDDHWIFWVGPFVGALAAAAYHQYILRAAAIKALGSFRSNPTN*

*Oleur*PIP2;9b Oleur061Scf1616g00016.1

>Oleur061Scf1616:35879..37453 (+ strand) class=gene length=1575

>*Oleur*PIP2;9b
ATGACGAAGGAAATGAGTGAAGAAGGACAGGTACATCACCATGGAAAGGACTACGTGGATCCACCACCAGCACCA
TTGCTAGACATGGGTGAGCTCAAGCTCTGGTCCTTTTACAGAGCTCTTATCGCCGAGTTCATCGCCACCCTTCTC
TTCCTCTACGTCACCGTCGCTACCGTCATCGGCCACAAGAAGCTCAACGCCGCCGATCAATGTGATGGCGTTGGC
ATTCTCGGTATCGCCTGGGCCTTTGGTGGCATGATCTTCATCCTCGTTTACTGTACCGCCGGCATCTCAGGTACT
CAATTCTTTAGGACAAATTCTTCTCCAGAAGCACCACCTATTCCTTGAAATTTCCCTAAATTTCTCTCTATTTGC
GCTGCAGGTGGTCACATAAACCCGGCTGTGACTTTTGGTTTGTTCTTGGCAAGGAAGGTATCGTTGATCCGTGCT
GTTATGTACATGATTGCACAGTGTTTGGGTGCCATTTGTGGAGTTGGGCTGGTGAAGGCCTTCATGAAGGGGTAC
TACAACCGTCTAGGCGGCGGCGCTAACTCGGTGTCTCATGGTTACAACACAGGCACAGCTTTGGGTGCTGAAATC
ATCGGCACCTTTGTGCTTGTTTACACCGTCTTCTCCGCCACTGACCCCAAGAGAAGTGCACGTGACTCTCATGTC
CCTGTAAGTAGAAGCAATTTCTAGCTTATTTTGTGTTTGCTTTTTCTAAAGTGAACTTGAACTTATTTTGGATTA
TTTTCAGGTTTTGGCTCCTTTACCAATTGGATTTGCAGTGTTCGTGGTGCACTTGGCCACCATCCCTATTACTGG
AACTGGTATCAACCCTGCTAGGAGTTTTGGAGCTGCCGTCATCTTCAACAATGATAAAGTCTGGGATGACCATGT
AAGTTCGTCTAGTAAGTTACTTTTTTTCAAGTATATATAATTAATTTTCTTGAACTAATAAGTATAATTTTGAT

GATGCATGTAGTGGATTTTCTGGGTTGGACCATTTGTGGGAGCATTGGCAGCAGCAGCATATCATCAGTACATCT
TAAGGGCTGCAGCTATTAAGGCATTGGGATCATTCCGCAGCAACCCCACAAACTAA

>*Oleur*PIP2;9b

ATGACGAAGGAAATGAGTGAAGAAGGACAGGTACATCACCATGGAAAGGACTACGTGGATCCACCACCAGCACCA
TTGCTAGACATGGGTGAGCTCAAGCTCTGGTCCTTTTACAGAGCTCTTATCGCCGAGTTCATCGCCACCCTTCTC
TTCCTCTACGTCACCGTCGCTACCGTCATCGGCCACAAGAAGCTCAACGCCGCCGATCAATGTGATGGCGTTGGC
ATTCTCGGTATCGCCTGGGCCTTTGGTGGCATGATCTTCATCCTCGTTTACTGTACCGCCGGCATCTCAGGTGGT
CACATAAACCCGGCTGTGACTTTTGGTTTGTTCTTGGCAAGGAAGGTATCGTTGATCCGTGCTGTTATGTACATG
ATTGCACAGTGTTTGGGTGCCATTTGTGGAGTTGGGCTGGTGAAGGCCTTCATGAAGGGGTACTACAACCGTCTA
GGCGGCGGCGCTAACTCGGTGTCTCATGGTTACAACACAGGCACAGCTTTGGGTGCTGAAATCATCGGCACCTTT
GTGCTTGTTTACACCGTCTTCTCCGCCACTGACCCCAAGAGAAGTGCACGTGACTCTCATGTCCCTGTTTTGGCT
CCTTTACCAATTGGATTTGCAGTGTTCGTGGTGCACTTGGCCACCATCCCTATTACTGGAACTGGTATCAACCCT
GCTAGGAGTTTTGGAGCTGCCGTCATCTTCAACAATGATAAAGTCTGGGATGACCATTGGATTTTCTGGGTTGGA
CCATTTGTGGGAGCATTGGCAGCAGCAGCATATCATCAGTACATCTTAAGGGCTGCAGCTATTAAGGCATTGGGA
TCATTCCGCAGCAACCCCACAAACTAA

>*Oleur*PIP2;9b

MTKEMSEEGQVHHHGKDYVDPPPAPLLDMGELKLWSFYRALIAEFIATLLFLYVTVATVIGHKKLNAADQCDGVG
ILGIAWAFGGMIFILVYCTAGISGGHINPAVTFGLFLARKVSLIRAVMYMIAQCLGAICGVGLVKAFMKGYYNRL
GGGANSVSHGYNTGTALGAEIIGTFVLVYTVFSATDPKRSARDSHVPVLAPLPIGFAVFVVHLATIPITGTGINP
ARSFGAAVIFNNDKVWDDHWIFWVGPFVGALAAAAYHQYILRAAAIKALGSFRSNPTN*

*Oleur*PIP2;10 Oleur061Scf0101g01021.1

>Oleur061Scf0101:86880..93288 (- strand) class=gene length=6409

>*Oleur*PIP2;10

ATGGAAAACCACGTTGGAGTGGCGAAGGACTACCGCGATCCACTGCCGGCCCCGCTGATTGGCTTGATGGAGCTT
ACAAAGTGGTCATTTTATAGAGCTGTTATAGCTGAGTTTATAGCCACCTTGCTCTTCCTTTACATTACGGTTTTG
ACTGTGATTGGGTACAAGAGCCAGAATGATACTCATTGCGGCGGCGGCGGCGTCGGAATTCTTGGTATTGCATGG
GCATTTGGTGGCATGATCTTCGTTCTTGTCTACTGCACTGCTGGAATCTCCGGTGAGTCTTGTTTATACATATAC
ATACATACATCAGATGCATAAATGTAATGGCATATTTCATGTCATGTCCATCTCAGGGGGACACATTAATCCAGC
AGTGACATTCGGGCTTTTCTTGGCCCGACAGGTGTCAATGATCAAAGCCATTATGTATATGGTGGCTCAGTGTTT
GGGGGCCATTTGTGGCGTCGCGCTGGTGAAGGCGGTACAGAAGTCACACTACAAGAAATATGGCGGCGGCGTAAA
TGAGCTCTCCGATGGCTACAGCACCGGCGGCGGATTGGCAGCAGAGATCATCGGAACATTCGTTCTTGTCTACAC
AGTTTTCTCTGCCATTGATGCCAAGAGAAAAGCCAGGGATTCCCATGTCCCTGTAAGTGCATATATACAATTCTC
CATTTTTGACCTGCTCGATAATCTACTTATAAGTACATTTAGAGCATGTGTTTTTCAATTAAACACTTGGTAAGT
GAATGCCATGCATTATGATACCATACACGTGAAACTGCAAAACTTCGTGGTTCAATAATGATACCATGCACGTTG
TACTGTAAAACCATATTGTCCATGCATCATTATTACATCAATATGGAGTTCCAATTTTTTTTACGATAAGATTT
GTATTGCTTGAGTTTGAAATCGAACTTGTCACAGTTAATCTTATTTTGAGCTCGACTTAATTCAGTTCTTTTATC
TATACGAAAGGAGCGAATATTGATATTTTTTGTATTTGAACTTGAATCATAGGTTTTGGCACCTCTCTCAATTGG
ATTTGCAGTTTTTATGGTTCACTTGGCCACTATCCCGATCACTGGTACTAGTATTAATCCAGCTAGAAGCTTTGG
ACCTGCAGTGATCTACAACCAGAAAAAGGCTTGGGATGATCAGGTTAACATAGCCTAAAAGATTTATAACGAACT
AAGGGTGCTTTTGGTTTGTCATTTTCAGTTTTGCAAAATACTGAAAACGTGTTTGATCTGTTATTTTAGAAAATG
CAGTTTTGAAAATAAGCCGTTTGGTATCCATTTTTTTCAAAATGAATTTTTCTATTTTTTCTTTTCAACCACACA
TTTTTTAATTAAAATAATATTACACATAAATCAAAAACTTCTCCTTATTTTTCAACTACACATTTTTCATTAATT
TACTATTCAAAAATGTATTTTTACATAAAAATGTCTTTCAAACACCCTCTAAATTTATAATAAAAACAACGTTAT
ATATATAGAATAATTTTATGGAATAAAATGTCATACAATAAATTTTCAAAATTTTAAACCAAACATGTTTTCTAA
TTTTTTGTTTTGAGAAATAATTTTTTAAAATGAAAAATCAAACGAGTTTTTACCAATATTGAATAGATCGTCAAA
ACAGAGACCTAAAATGAACTCAAATTGAAAATTGAAACATAAACCAAACAGACCTAAATCTTACGATGAAATTTG
GATCTACAACTTGAATATTTTTTCTCTCCTCTATTCAATTCATTGTTGATGCTATTGGGGTGTCCAAATGAAAGA
TGTTAGTAAGACTAAAGTCAGGGAGAATATTTTATTTCAATTACTCCTAATTATTTATCCGACAATAAGAATGAT
TTTTAAAACGCCACACTAGTTAACTAAAAGTTACATGGATCCCTTTCTATAAGTACTTTGTTCCTTTATTGCCGA
AATAGTCCTAATGAAATGCCTAGTAGGATTCATATTATAAAATATGAGAAAATTAATATGCGTTTAATAATAACC
TAGCTCAATCTTTATGATCCTTAATATATATGAACTCGAGATTGAGTATGGAAAAAATATTTATATCCCAACACT
AGTTGGAACGACTATGAACAAAATACAATATGAGTACCATTCCTATTAGTCATAAAATTTATAAATCCCTGTTTG
GTTCCTATTCTTTTCTCATTAAAATGAAAAATATGATAGTTATTCATCATATCAAGTCACCAAATTTGAATTTAT
ATTTCTTGTGTAGGGTCCGTAACGACTATATTTTTCATTCTCATTCTCCCAAAAATGAGAAACAAACGGGGGCTA
ATTTTAACATTATCTACCCCACCAGCCCACAAGTGATAATTTATTTGTTTATTTTTGTTCTCTTAATTCCAGTGG
ATATTCTGGGTAGGACCCTTCATTGGTGCAGCCATGGCAGCCTTCTACCACCAGTGTATTTTGAGAGCTAAAGCC
TTTCGTTCATATAGAAGCAGCTCCCGTATTTAA

>*Oleur*PIP2;10

ATGGAAAACCACGTTGGAGTGGCGAAGGACTACCGCGATCCACTGCCGGCCCCGCTGATTGGCTTGATGGAGCTT
ACAAAGTGGTCATTTTATAGAGCTGTTATAGCTGAGTTTATAGCCACCTTGCTCTTCCTTTACATTACGGTTTTG
ACTGTGATTGGGTACAAGAGCCAGAATGATACTCATTGCGGCGGCGGCGGCGTCGGAATTCTTGGTATTGCATGG
GCATTTGGTGGCATGATCTTCGTTCTTGTCTACTGCACTGCTGGAATCTCCGGGGGACACATTAATCCAGCAGTG
ACATTCGGGCTTTTCTTGGCCCGACAGGTGTCAATGATCAAAGCCATTATGTATATGGTGGCTCAGTGTTTGGGG
GCCATTTGTGGCGTCGCGCTGGTGAAGGCGGTACAGAAGTCACACTACAAGAAATATGGCGGCGGCGTAAATGAG
CTCTCCGATGGCTACAGCACCGGCGGCGGATTGGCAGCAGAGATCATCGGAACATTCGTTCTTGTCTACACAGTT
TTCTCTGCCATTGATGCCAAGAGAAAAGCCAGGGATTCCCATGTCCCTGTTTTGGCACCTCTCTCAATTGGATTT
GCAGTTTTTATGGTTCACTTGGCCACTATCCCGATCACTGGTACTAGTATTAATCCAGCTAGAAGCTTTGGACCT
GCATGGATATTCTGGGTAGGACCCTTCATTGGTGCAGCCATGGCAGCCTTCTACCACCAGTGTATTTTGAGAGCT
AAAGCCTTTCGTTCATATAGAAGCAGCTCCCGTATTTAA
>*Oleur*PIP2;10
MENHVGVAKDYRDPLPAPLIGLMELTKWSFYRAVIAEFIATLLFLYITVLTVIGYKSQNDTHCGGGGVGILGIAW
AFGGMIFVLVYCTAGISGGHINPAVTFGLFLARQVSMIKAIMYMVAQCLGAICGVALVKAVQKSHYKKYGGGVNE
LSDGYSTGGGLAAEIIGTFVLVYTVFSAIDAKRKARDSHVPVLAPLSIGFAVFMVHLATIPITGTSINPARSFGP
AWIFWVGPFIGAAMAAFYHQCILRAKAFRSYRSSSRI*

*Oleur*PIP2;12 Oleur061Scf0101g01021.1

>Oleur061Scf0156:141041..142902 (- strand) class=gene length=1862

>*Oleur*PIP2;12
ATGACAAAGGAAGTTAGCGAAGAAGGACAAGTACATCATCATGGCAAGGACTACGTGGATCCACCACCAGCTCCA
CTGCTGGACATGGGTGAGCTCAAGCTCTGGTCTTTCTATAGGGCCCTAATCGCTGAGTTCATCGCCACACTTCTC
TTTCTCTACGTCACTGTCGCCACCGTCATCGGCCACAAGAAGCTCAACAACGCCGACCAGTGCGACGGAGTTGGT
CTCCTCGGTATTGCCTGGGCCTTTGGTGGCATGATCTTCATTCTCGTCTACTGCACCGCTGGAATCTCAGGTTCC
ACCACCATTTTCAACACCCTAATAGAATCTCTTACGAAATTTTTGTGCTTCCTTGTCTGAAATATGAACTTTTTG
TTATTTTTGTGGTGTAGGTGGTCACATAAATCCGGCCGTGACTTTCGGGTTGTTCTTGGCGAGGAAGGTGTCTCT
GTTTAGGGCGCTTGGGTACATGGTGGCGCAGTGTTTGGGTGCTATTTGCGGAGTTGGGTTGGTAAAGGCCTTCAT
GAAGAGTTTCTACAACCACTTGGGTGGTGGTGCTAACTCTGTGGCTTCTGGGTACAACAAGGGCACTGCCCTCGG
TGCTGAGATTATCGGCACTTTTGTACTTGTTTACACCGTCTTCTCAGCCACTGACCCAAAGAGAAGCGCACGTGA
TTCTCACGTCCCGGTACGTATACATACAACTCATCTATTCAAGATATTTTTAGTGATTCTTGAGAATTAACTTGA
AGTTTTGGTGCTTATAGGTTTTGGCTCCGCTTCCAATTGGATTTGCAGTATTCATGGTTCATTTGGCCACCATCC
CAATCACTGGAACTGGTATAAACCCTGCTAGGAGCTTCGGAGCTGCTGTTATCTTCAACAACGAGAAAGTCTGGG
ATGATCATGTAAGTTTCAATTGCATGCAGGCATGCATATAGAGATTTGTTCTGGTATCTTTTGTTTTATGTTTAA
ACATGAACTTACTTTGATTGTGCAGTGGATTTTCTGGGTTGGACCATTTGTGGGAGCATTGGCAGCAGCGATATA
CCACCAGTATATCCTTAGAGCTGCAGCCCTTAAGGCTTTGGGATCCTTCAGGAGCAACCCCACCAACTAA
>*Oleur*PIP2;12
ATGACAAAGGAAGTTAGCGAAGAAGGACAAGTACATCATCATGGCAAGGACTACGTGGATCCACCACCAGCTCCA
CTGCTGGACATGGGTGAGCTCAAGCTCTGGTCTTTCTATAGGGCCCTAATCGCTGAGTTCATCGCCACACTTCTC
TTTCTCTACGTCACTGTCGCCACCGTCATCGGCCACAAGAAGCTCAACAACGCCGACCAGTGCGACGGAGTTGGT
CTCCTCGGTATTGCCTGGGCCTTTGGTGGCATGATCTTCATTCTCGTCTACTGCACCGCTGGAATCTCAGGTGGT
CACATAAATCCGGCCGTGACTTTCGGGTTGTTCTTGGCGAGGAAGGTGTCTCTGTTTAGGGCGCTTGGGTACATG
GTGGCGCAGTGTTTGGGTGCTATTTGCGGAGTTGGGTTGGTAAAGGCCTTCATGAAGAGTTTCTACAACCACTTG
GGTGGTGGTGCTAACTCTGTGGCTTCTGGGTACAACAAGGGCACTGCCCTCGGTGCTGAGATTATCGGCACTTTT
GTACTTGTTTACACCGTCTTCTCAGCCACTGACCCAAAGAGAAGCGCACGTGATTCTCACGTCCCGGTTTTGGCT
CCGCTTCCAATTGGATTTGCAGTATTCATGGTTCATTTGGCCACCATCCCAATCACTGGAACTGGTATAAACCCT
GCTAGGAGCTTCGGAGCTGCTGTTATCTTCAACAACGAGAAAGTCTGGGATGATCATTGGATTTTCTGGGTTGGA
CCATTTGTGGGAGCATTGGCAGCAGCGATATACCACCAGTATATCCTTAGAGCTGCAGCCCTTAAGGCTTTGGGA
TCCTTCAGGAGCAACCCCACCAACTAA
>*Oleur*PIP2;12
MTKEVSEEGQVHHHGKDYVDPPPAPLLDMGELKLWSFYRALIAEFIATLLFLYVTVATVIGHKKLNNADQCDGVG
LLGIAWAFGGMIFILVYCTAGISGGHINPAVTFGLFLARKVSLFRALGYMVAQCLGAICGVGLVKAFMKSFYNHL
GGGANSVASGYNKGTALGAEIIGTFVLVYTVFSATDPKRSARDSHVPVLAPLPIGFAVFMVHLATIPITGTGINP
ARSFGAAVIFNNEKVWDDHWIFWVGPFVGALAAAIYHQYILRAAALKALGSFRSNPTN*

*Oleur*TIP1;1 Oleur061Scf3090g01006.1

>Oleur061Scf3090:93487..94956 (- strand) class=gene length=1470

>*Oleur*TIP1;1
ATGCCTATTTCAAGAGTCGCTATTGGATCTCCGGCGGAGTTCAGCCAACTGGATGCCATCAAGGCTGCCGTGGCT
GAGTTCATTTCCATGTTGATTTTTGTTTTTGCTGGTCAAGGCTCTGGCATGGCTTTCGGTAAGTAGTTCTGCTGA
ACAAACTCATTTAAAAGTTTTAAGTTGTTAGAAATCGATAATTACATCATTGGATTTTTATTTTTTCACTTTTTC
AACAGCTAAGCTTACGGACAATGAATCAACAACGCCGGCTGGCCTCATTGCTGCATCGATAGCACATGCTTTTGC
GCTTTTTGTGGCGGTTTCGGTCGGTGCCAACATCTCCGGCGGCCACGTAAACCCTGCTGTCACCTTAGGTGCATT
TGTTGGTGGTCACATCACATTGTTTAGAAGCATCATGTACTGGATTGCCCAATTGCTTGGATCTGTTATTGCATG
CTTGCTTCTTAAGTTTGCCACTGGTGGACTCGTAAGTTGAATCTTATATACTCTGTCCAGTTGAGTCCATTACTG
TAACATATTGGCTAATGTTCAATCCTTGATTGTAGGAAACATCAGCCTTTGCACTTTCATCGGGAGTGACAGTTT
GGAATGCAGTCATTTTCGAGATAGTGATGACCTTTGGACTTGTTTACACAGTTTATGCCACTGCAGTGGATCCAA
AGAAGGGTAATTTAGGGATCATTGCTCCCATTGCCATTGGCTTCATTGTTGGTGCCAACATCTTAGCCGGTGGCG
CATTCGATGGTGCATCCATGAACCCAGCCGTGTCTTTTGGCCCTGCTGTCGTCAGCTGGACATGGGACAGCCACT
GGGTCTACTGGCTCGGCCCATTTGTCGGTGCTGGAATTGCTGCTCTGGTCTATGAAATCTTGTTCATTAACCAGA
GCCATCAACAGCTCCCTACCAGTGCAGAATAC**TAA**

>*Oleur*TIP1;1
ATGCCTATTTCAAGAGTCGCTATTGGATCTCCGGCGGAGTTCAGCCAACTGGATGCCATCAAGGCTGCCGTGGCT
GAGTTCATTTCCATGTTGATTTTTGTTTTTGCTGGTCAAGGCTCTGGCATGGCTTTCGCTAAGCTTACGGACAAT
GAATCAACAACGCCGGCTGGCCTCATTGCTGCATCGATAGCACATGCTTTTGCGCTTTTTGTGGCGGTTTCGGTC
GGTGCCAACATCTCCGGCGGCCACGTAAACCCTGCTGTCACCTTAGGTGCATTTGTTGGTGGTCACATCACATTG
TTTAGAAGCATCATGTACTGGATTGCCCAATTGCTTGGATCTGTTATTGCATGCTTGCTTCTTAAGTTTGCCACT
GGTGGACTCGAAACATCAGCCTTTGCACTTTCATCGGGAGTGACAGTTTGGAATGCAGTCATTTTCGAGATAGTG
ATGACCTTTGGACTTGTTTACACAGTTTATGCCACTGCAGTGGATCCAAAGAAGGGTAATTTAGGGATCATTGCT
CCCATTGCCATTGGCTTCATTGTTGGTGCCAACATCTTAGCCGGTGGCGCATTCGATGGTGCATCCATGAACCCA
GCCGTGTCTTTTGGCCCTGCTGTCGTCAGCTGGACATGGGACAGCCACTGGGTCTACTGGCTCGGCCCATTTGTC
GGTGCTGGAATTGCTGCTCTGGTCTATGAAATCTTGTTCATTAACCAGAGCCATCAACAGCTCCCTACCAGTGCA
GAATAC**TAA**

>*Oleur*TIP1;1
MPISRVAIGSPAEFSQLDAIKAAVAEFISMLIFVFAGQGSGMAFAKLTDNESTTPAGLIAASIAHAFALFVAVSV
GANISGGHVNPAVTLGAFVGGHITLFRSIMYWIAQLLGSVIACLLLKFATGGLETSAFALSSGVTVWNAVIFEIV
MTFGLVYTVYATAVDPKKGNLGIIAPIAIGFIVGANILAGGAFDGASMNPAVSFGPAVVSWTWDSHWVYWLGPFV
GAGIAALVYEILFINQSHQQLPTSAEY*

***Oleur*TIP1;2a Oleur061Scf3090g01006.1**

>Oleur061Scf8429:141483..143267 (+ strand) class=gene length=1785

>*Oleur*TIP1;2a
ATGCCTTTCTCAAGAATTTCGGTCGGAAGTCCGGCGGAGGCCAGCCATCCAGATACCCTGAAAGCCGCCTTAGCC
GAATTCATTTCAATGGTAATTTTTGTATTTGCTGGTGAAGGCTCTGGCATGGCTTTTGGTAAGTACTTGTGCCCT
CATATTAGTTTTACAATTGTTCTTCCTTGACAAAATTATTTGCTTACTTCCTTGACAAAATGACACTTGGTATTC
AATCATTAGACTGTTTAAAAAACAGTTAGTTTTTCACCCAATCATCTGCTGCCAACGTTGTATCTATTAAGTAGG
TGGGCGGAAAATTATGTACAAATGACTCAATCCTAAGTTCTAAACACTTGTCCTTTATTCTATTCAATCTCATAT
ATAATCATTTTTTCTACAGCCAAGCTTACAGACGGTGGATCGAGCACGCCGGCTGGCCTTATTGCTGCGGCGATA
GCCCATGCTTTCGCACTTTTTGTGGCAGTCTCGGTTGCTGCCAACATTTCCGGTGGTCATGTAAACCCTGCAGTC
ACCTTTGGTGCCTTTGTTGGTGGTCACCTATCATTGTTTAAAACTATTTTGTATTGGATTGCCCAATTGCTTGGA
GCTATTGTTGCATGCTTGCTCCTAAAGTTCTCCACCGGTGGCTTGGTAAGTAATTAATTCCTCAAAGCTATATTA
TTACACATAAAAGTTCTTCTTCATCATTTTTCTGAAATGTCGAAGAAGAACGAATGATGCGATTTCATTAGAATT
CTTGAAAAAAAATAAGGAAAATTCAAATTAATCTCTTCTACAGGAAACAAAAATAAAAGCAAAGACTATACCAAA
CGTCCCTTAGAATCGCTGGCCTACAGCATGTTTGGCTGGCTAAGCTTCAAAAGAAGTAAAAGTTAATTTTCTTTT
CAAACATGTTAATAGAACTTATTTTCCTTAATTTTAAGTAAAAGAATTATTTATTTAAAAGAAAATTTTACGTAT
AAGAACCTTCCATGTTTTCAATCTCCACGAATAAAAAATTGTCCATTACAGAACTTACTTATTTCCATGCTTTTG
TGTGTGACCTGAACTCAAATTTTGTCTTGTTACATCGTGCAGGAAACTTCCGCATTTGCACTTTCAACCGGGGTA
TCGTCGTGGAATGCCCTTGTTTTCGAAATAGTGATGACATTTGGCCTGGTTTACACAGTTTATGCCACAGCAGTG
GATCCAAAGAGGGGTGATATAGGTATCATAGCCCCTATTGCCATTGGCTTCATTGTGGGTGCCAACATTTTGGCT
GGCGGTGCCTTCGACGGTGCATCCATGAACCCAGCCGTGTCGTTCGGCCCTGCAGTCGTCAGCTGGACATGGGAT
AGCCACTGGGTGTACTGGCTTGGCCCATTTGTTGGTGCTGGAATTGCTGCTATTGTCTATGAAATCGTCTTCATT
AACCAGACCACTCACGAGCCACTCCCCTCCAGTGAGTAC**TAA**

>*Oleur*TIP1;2a
ATGCCTTTCTCAAGAATTTCGGTCGGAAGTCCGGCGGAGGCCAGCCATCCAGATACCCTGAAAGCCGCCTTAGCC
GAATTCATTTCAATGGTAATTTTTGTATTTGCTGGTGAAGGCTCTGGCATGGCTTTTGCCAAGCTTACAGACGGT
GGATCGAGCACGCCGGCTGGCCTTATTGCTGCGGCGATAGCCCATGCTTTCGCACTTTTTGTGGCAGTCTCGGTT
GCTGCCAACATTTCCGGTGGTCATGTAAACCCTGCAGTCACCTTTGGTGCCTTTGTTGGTGGTCACCTATCATTG

TTTAAAACTATTTTGTATTGGATTGCCCAATTGCTTGGAGCTATTGTTGCATGCTTGCTCCTAAAGTTCTCCACC
GGTGGCTTGGAAACTTCCGCATTTGCACTTTCAACCGGGGTATCGTCGTGGAATGCCCTTGTTTTCGAAATAGTG
ATGACATTTGGCCTGGTTTACACAGTTTATGCCACAGCAGTGGATCCAAAGAGGGGTGATATAGGTATCATAGCC
CCTATTGCCATTGGCTTCATTGTGGGTGCCAACATTTTGGCTGGCGGTGCCTTCGACGGTGCATCCATGAACCCA
GCCGTGTCGTTCGGCCCTGCAGTCGTCAGCTGGACATGGGATAGCCACTGGGTGTACTGGCTTGGCCCATTTGTT
GGTGCTGGAATTGCTGCTATTGTCTATGAAATCGTCTTCATTAACCAGACCACTCACGAGCCACTCCCCTCCAGT
GAGTAC**TAA**

>*Oleur*TIP1;2a
MPFSRISVGSPAEASHPDTLKAALAEFISMVIFVFAGEGSGMAFAKLTDGGSSTPAGLIAAAIAHAFALFVAVSV
AANISGGHVNPAVTFGAFVGGHLSLFKTILYWIAQLLGAIVACLLLKFSTGGLETSAFALSTGVSSWNALVFEIV
MTFGLVYTVYATAVDPKRGDIGIIAPIAIGFIVGANILAGGAFDGASMNPAVSFGPAVVSWTWDSHWVYWLGPFV
GAGIAAIVYEIVFINQTTHEPLPSSEY*

*Oleur*TIP1;2b Oleur061Scf0417g02009.1

>Oleur061Scf0417:223466..225244 (- strand) class=gene length=1779

>*Oleur*TIP1;2b
ATGCCTTTCTCAAGAATTTCGGTCGGAAGTCCGGCGGAGGCCAGCCATCCAGATACCCTGAAAGCCGCCTTAGCC
GAATTCATTTCAATGGTAATTTTTGTATTTGCTGGTGAAGGCTCTGGCATGGCTTTTGGTAAGTACTTGTGCCCT
CATATTAGTTTTACAATTGTTCTTCCTTGACAGAATTATTTGCTTACTTCCTTGACAAAATGACACGTGGTATTC
AATCATTAGACTATTTAAAAAACAGTTAATTTTTCACCCAATCATTTGCTGCCAACGTTGTATCTATTAAGTAGG
TGGGCAGAAAATTCTGTACAAATGGCTAAATCCTAAGTTCTAAACACTTGTCCTTTATTCTATTCAATCTCATTT
ATTATCATTTTTTCTACAGCCAAGCTTACAGACGGTGGATCGAGCACGCCGGCCGGCCTTATTGCTGCGGCGATA
GCCCATGCTTTCGCACTTTTTGTGGCAGTCTCGGTTGCTGCCAACATTTCCGGTGGTCATGTAAACCCTGCAGTC
ACCTTCGGTGCCTTTGTTGGTGGTCACCTATCATTGTTTAAAACTATTTTGTATTGGATTGCCCAATTGCTTGGA
GCTATTGTTGCATGCTTGCTCCTAAAGTTCTCCACCGGTGGCTTGGTAAGTAATTAATTCATCAAAGCTATATTA
TTACACATAAAAGTTCTTCTTCATCATTTTTCTGAAATGTCGAAGAAGAACGAATGATGCGATTTCATTAGAATT
CTTGAAAAAAAATAAGGAAAATTCAAATTAATCTCTTCTACAGGAAACAAAAATAAAAGCAAAGACTATACCTAA
CGGACCCTTAGAATCCCTGGCCTAGAGCATGTTTGGCTGGCTTAGCTTCAAAAGAAGTAAAAGTTAATTTTCTTT
TCAAACATGTTAATAGAACTTATTTTCCTTAATTTTAAGTAAAAGAACTATTTATTTAAAAGACAATTTTACGCA
TAAGAACCTTCCATGTTTTCAATCTCCCAAGAATAAAAACTTGTCCACTACAGAACTTACTTATTTCCATGCTTT
TGTGTGACCAGAACTCAAATTTTGTCTTGTTACATCATGCAGGAAACTTCCGCATTTGCACTTTCAACCGGGGTA
TCGTCGTGGAATGCCCTTGTTTTCGAAATAGTGATGACGTTTGGACTGGTTTACACAGTTTATGCCACAGCAGTA
GATCCAAAGAGGGGTGATATAGGTATCATAGCCCCTATTGCCATTGGCTTCATCGTGGGTGCCAACATTTTGGCC
GGCGGTGCCTTTGACGGTGCATCCATGAACCCAGCCGTGTCGTTCGGCCCTGCAGTCGTTAGCTGGACATGGGAC
AGCCACTGGGTGTACTGGCTTGGCCCATTTGTTGGTGCTGGAATTGCTGCTATTGTCTATGAAATCGTCTTCATT
AACCAGAGCACTCATGAGCCACTCCCCTCCAGTGAGTAC**TAA**

>*Oleur*TIP1;2b
ATGCCTTTCTCAAGAATTTCGGTCGGAAGTCCGGCGGAGGCCAGCCATCCAGATACCCTGAAAGCCGCCTTAGCC
GAATTCATTTCAATGGTAATTTTTGTATTTGCTGGTGAAGGCTCTGGCATGGCTTTTGCCAAGCTTACAGACGGT
GGATCGAGCACGCCGGCCGGCCTTATTGCTGCGGCGATAGCCCATGCTTTCGCACTTTTTGTGGCAGTCTCGGTT
GCTGCCAACATTTCCGGTGGTCATGTAAACCCTGCAGTCACCTTCGGTGCCTTTGTTGGTGGTCACCTATCATTG
TTTAAAACTATTTTGTATTGGATTGCCCAATTGCTTGGAGCTATTGTTGCATGCTTGCTCCTAAAGTTCTCCACC
GGTGGCTTGGAAACTTCCGCATTTGCACTTTCAACCGGGGTATCGTCGTGGAATGCCCTTGTTTTCGAAATAGTG
ATGACGTTTGGACTGGTTTACACAGTTTATGCCACAGCAGTAGATCCAAAGAGGGGTGATATAGGTATCATAGCC
CCTATTGCCATTGGCTTCATCGTGGGTGCCAACATTTTGGCCGGCGGTGCCTTTGACGGTGCATCCATGAACCCA
GCCGTGTCGTTCGGCCCTGCAGTCGTTAGCTGGACATGGGACAGCCACTGGGTGTACTGGCTTGGCCCATTTGTT
GGTGCTGGAATTGCTGCTATTGTCTATGAAATCGTCTTCATTAACCAGAGCACTCATGAGCCACTCCCCTCCAGT
GAGTAC**TAA**

>*Oleur*TIP1;2b
MPFSRISVGSPAEASHPDTLKAALAEFISMVIFVFAGEGSGMAFAKLTDGGSSTPAGLIAAAIAHAFALFVAVSV
AANISGGHVNPAVTFGAFVGGHLSLFKTILYWIAQLLGAIVACLLLKFSTGGLETSAFALSTGVSSWNALVFEIV
MTFGLVYTVYATAVDPKRGDIGIIAPIAIGFIVGANILAGGAFDGASMNPAVSFGPAVVSWTWDSHWVYWLGPFV
GAGIAAIVYEIVFINQSTHEPLPSSEY*

*Oleur*TIP1;3a Oleur061Scf4360g00007.1

>Oleur061Scf1330:642994..644469 (- strand) class=gene length=1476

>*Oleur*TIP1;3a

ATGTCTAGCGAGTTCTTTAACGTGCCACTCCGACGGGTTGCCTTTGGACATCGAGATGAGTTTCGCCAGCCCGGT
GCTATCAAGGCGGCGCTAGCCGAGTTCTTCAGTACCCTGATTTTTGTGTTTGCTGGTGAGGGTTCCGGCATGGCT
TACAACAAATTGACTGGGAATGCTCCTACCTCCCCATCCGGCCTTGTGGCAGCAGCGGTGGCTCATGGTTTTGCT
TTGTTTGTAGCAGTTTCCGTCAGTGCTAACATTTCCGGCGGCCATGTAAACCCTGCCGTCACTTTTGGTTTGTTT
CTTGGTGGAAACGTCTCTCTCTTCCGTGGTATCCTTTACATAATTGCTCAGCTGCTGGGCTCTGTCGTTGCTTGC
CTTCTCCTCTTGTTCTCAACTGGTGGACTGGTAAGTTTTAAAAATAAACAAATTCTTGGTCCTCCCAAACAATAA
CAGCGGCTCCTAATTTATTGAATGAAACTTCGATGCAGGAAACATCTGCATTTGCTTTAACTGCCGGCGTGTCGG
TGTGGAACGCCTTTGTTTTCGAGATTGTAATGACCTTTGGCCTGGTTTACACTGTGTACGCCACCGCTGTTGACC
CAAAGAAGGGAGAAATTGGCATTATTGCACCAATTGCAATTGGTTTCATCGTGGGTGCCAATATTCTGGCGGGTG
GTGCATTTACCGGTGCTTCAATGAACCCTGCAGTGTCATTTGGTCCAGCTCTGGTCAGCTTCACCTGGACCCATC
ACTGGGTGTACTGGGCTGGACCTCTTATTGGTGGTGGCCTTGCTGGTATTGTTTATGAAGTCTTCTTCATCAACC
ATTCTCACGAACCCTTGCCAGGTTCAGAGTAC**TGA**
>*Oleur*TIP1;3a
ATGTCTAGCGAGTTCTTTAACGTGCCACTCCGACGGGTTGCCTTTGGACATCGAGATGAGTTTCGCCAGCCCGGT
GCTATCAAGGCGGCGCTAGCCGAGTTCTTCAGTACCCTGATTTTTGTGTTTGCTGGTGAGGGTTCCGGCATGGCT
TACAACAAATTGACTGGGAATGCTCCTACCTCCCCATCCGGCCTTGTGGCAGCAGCGGTGGCTCATGGTTTTGCT
TTGTTTGTAGCAGTTTCCGTCAGTGCTAACATTTCCGGCGGCCATGTAAACCCTGCCGTCACTTTTGGTTTGTTT
CTTGGTGGAAACGTCTCTCTCTTCCGTGGTATCCTTTACATAATTGCTCAGCTGCTGGGCTCTGTCGTTGCTTGC
CTTCTCCTCTTGTTCTCAACTGGTGGACTGGAAACATCTGCATTTGCTTTAACTGCCGGCGTGTCGGTGTGGAAC
GCCTTTGTTTTCGAGATTGTAATGACCTTTGGCCTGGTTTACACTGTGTACGCCACCGCTGTTGACCCAAAGAAG
GGAGAAATTGGCATTATTGCACCAATTGCAATTGGTTTCATCGTGGGTGCCAATATTCTGGCGGGTGGTGCATTT
ACCGGTGCTTCAATGAACCCTGCAGTGTCATTTGGTCCAGCTCTGGTCAGCTTCACCTGGACCCATCACTGGGTG
TACTGGGCTGGACCTCTTATTGGTGGTGGCCTTGCTGGTATTGTTTATGAAGTCTTCTTCATCAACCATTCTCAC
GAACCCTTGCCAGGTTCAGAGTAC**TGA**
>*Oleur*TIP1;3a
MSSEFFNVPLRRVAFGHRDEFRQPGAIKAALAEFFSTLIFVFAGEGSGMAYNKLTGNAPTSPSGLVAAAVAHGFA
LFVAVSVSANISGGHVNPAVTFGLFLGGNVSLFRGILYIIAQLLGSVVACLLLLFSTGGLETSAFALTAGVSVWN
AFVFEIVMTFGLVYTVYATAVDPKKGEIGIIAPIAIGFIVGANILAGGAFTGASMNPAVSFGPALVSFTWTHHWV
YWAGPLIGGGLAGIVYEVFFINHSHEPLPGSEY*

***Oleur*TIP1;3b Oleur061Scf7549g01032.1**

>Oleur061Scf4360:27330..28805 (+ strand) class=gene length=1476

>*Oleur*TIP1;3b
ATGTCTAGCGAGTTCTTTAACGTGCCACTCCGACGGGTTGCCTTTGGACATCGAGATGAGTTTCGCCAGCCCGGT
GCTATCAAGGCGGCGCTAGCCGAGTTCTTCAGTACCCTGATTTTTGTGTTTGCTGGTGAGGGTTCCGGCATGGCT
TACAACAAATTGACTGGGAATGCTCCTACCTCCCCATCCGGCCTTGTGGCAGCAGCGGTGGCTCATGGTTTTGCT
TTGTTTGTAGCAGTTTCCGTCAGTGCTAACATTTCCGGCGGCCATGTAAACCCTGCCGTCACTTTTGGTTTGTTT
CTTGGTGGAAACGTCTCTCTCTTCCGTGGTATCCTTTACATAATTGCTCAGCTGCTGGGCTCTGTCGTTGCTTGC
CTTCTCCTCTTGTTCTCAACTGGTGGACTGGTAAGTTTTAAAAATAAACAAATTCTTGGTCCTCCCAAACAATAA
CAGCGGCTCCTAATTTATTGAATGAAACTTCGATGCAGGAAACATCTGCATTTGCTTTAACTGCCGGCGTGTCGG
TGTGGAACGCCTTTGTTTTCGAGATTGTAATGACCTTTGGCCTGGTTTACACTGTGTACGCCACCGCTGTTGACC
CAAAGAAGGGAGAAATTGGCATTATTGCACCAATTGCAATTGGTTTCATCGTGGGTGCCAATATTCTGGCGGGTG
GTGCATTTACCGGTGCTTCAATGAACCCTGCAGTATCATTTGGTCCAGCTCTGGTCAGCTTCACCTGGACCCATC
ACTGGGTGTACTGGGCTGGACCTCTTATTGGTGGTGGCCTTGCTGGTATTGTTTATGAAGTCTTCTTCATCAACC
ATTCTCACGAACCCTTGCCAGGTTCAGAGTAC**TGA**
>*Oleur*TIP1;3b
ATGTCTAGCGAGTTCTTTAACGTGCCACTCCGACGGGTTGCCTTTGGACATCGAGATGAGTTTCGCCAGCCCGGT
GCTATCAAGGCGGCGCTAGCCGAGTTCTTCAGTACCCTGATTTTTGTGTTTGCTGGTGAGGGTTCCGGCATGGCT
TACAACAAATTGACTGGGAATGCTCCTACCTCCCCATCCGGCCTTGTGGCAGCAGCGGTGGCTCATGGTTTTGCT
TTGTTTGTAGCAGTTTCCGTCAGTGCTAACATTTCCGGCGGCCATGTAAACCCTGCCGTCACTTTTGGTTTGTTT
CTTGGTGGAAACGTCTCTCTCTTCCGTGGTATCCTTTACATAATTGCTCAGCTGCTGGGCTCTGTCGTTGCTTGC
CTTCTCCTCTTGTTCTCAACTGGTGGACTGGAAACATCTGCATTTGCTTTAACTGCCGGCGTGTCGGTGTGGAAC
GCCTTTGTTTTCGAGATTGTAATGACCTTTGGCCTGGTTTACACTGTGTACGCCACCGCTGTTGACCCAAAGAAG
GGAGAAATTGGCATTATTGCACCAATTGCAATTGGTTTCATCGTGGGTGCCAATATTCTGGCGGGTGGTGCATTT
ACCGGTGCTTCAATGAACCCTGCAGTATCATTTGGTCCAGCTCTGGTCAGCTTCACCTGGACCCATCACTGGGTG
TACTGGGCTGGACCTCTTATTGGTGGTGGCCTTGCTGGTATTGTTTATGAAGTCTTCTTCATCAACCATTCTCAC
GAACCCTTGCCAGGTTCAGAGTAC**TGA**
>*Oleur*TIP1;3b
MSSEFFNVPLRRVAFGHRDEFRQPGAIKAALAEFFSTLIFVFAGEGSGMAYNKLTGNAPTSPSGLVAAAVAHGFA
LFVAVSVSANISGGHVNPAVTFGLFLGGNVSLFRGILYIIAQLLGSVVACLLLLFSTGGLETSAFALTAGVSVWN

AFVFEIVMTFGLVYTVYATAVDPKKGEIGIIAPIAIGFIVGANILAGGAFTGASMNPAVSFGPALVSFTWTHHWV
YWAGPLIGGGLAGIVYEVFFINHSHEPLPGSEY*

***Oleur*TIP1;3c Oleur061Scf0417g02009.1**

>Oleur061Scf7549:241767..243178 (- strand) class=gene length=1412

>*Oleur*TIP1;3c
ATGTCTAGCGAGTTCTTTAACGTGCCACTCCGACGGGTTGCCTTTGGACATCGAGATGAGTTTCGCCAGCCCGGT
GCTATCAAGGCGGCGCTAGCCGAGTTCTTCAGTACCCTGATTTTTGTGTTTGCTGGTGAGGGTTCCGGCATGGCT
TACAACAAATTGACTGGGAATGCTCCTACCTCCCCATCCGGCCTTGTGGCAGCAGCGGTGGCTCATGGTTTTGCT
TTGTTTGTAGCAGTTTCCGTCAGTGCTAACATTTCCGGCGGCCATGTAAACCCTGCCGTCACTTTTGGTTTGTTT
CTTGGTGGAAACGTCTCTCTCTTCCGTGGTATCCTTTACATAATTGCTCAGCTGCTGGGCTCTGTCGTTGCTTGC
CTTCTCCTCTTGTTCTCAACTGGTGGACTGGTAAGTTTTAAAAATAAACAAATTCTTGGTCCTCCCAAACAATAA
CAGCGGCTCCTAATTTATTGAATGAAACTTCGATGCAGGAAACATCTGCATTTGCTTTAACTGCCGGCGTGTCGG
TGTGGAACGCCTTTGTTTTCGAGATTGTAATGACCTTTGGCCTGGTTTACACTGTGTACGCCACCGCTGTTGACC
AAAAGAAGGGAGAAATTGGCATTATTGCACCAATTGCAATTGGTTTCATCGTGGGTGCCAATATTCTGGCGGGTG
GTGCATTTACCGGTGCTTCAATGAACCCTGCAGTATCATTTGGTCCAGCTCTGGTCAGCTTCACCTGGACCCATC
ACTGGGTGTACTGGGCTGGACCTCTTATTGGTGGTGGCCTTGCTGGTATTGTTTATGAAGTCTTCTTCATCAACC
ATTCTCACGAACCCTTGCCAGGTTCAGAGTAC**TGA**
>*Oleur*TIP1;3c
ATGTCTAGCGAGTTCTTTAACGTGCCACTCCGACGGGTTGCCTTTGGACATCGAGATGAGTTTCGCCAGCCCGGT
GCTATCAAGGCGGCGCTAGCCGAGTTCTTCAGTACCCTGATTTTTGTGTTTGCTGGTGAGGGTTCCGGCATGGCT
TACAACAAATTGACTGGGAATGCTCCTACCTCCCCATCCGGCCTTGTGGCAGCAGCGGTGGCTCATGGTTTTGCT
TTGTTTGTAGCAGTTTCCGTCAGTGCTAACATTTCCGGCGGCCATGTAAACCCTGCCGTCACTTTTGGTTTGTTT
CTTGGTGGAAACGTCTCTCTCTTCCGTGGTATCCTTTACATAATTGCTCAGCTGCTGGGCTCTGTCGTTGCTTGC
CTTCTCCTCTTGTTCTCAACTGGTGGACTGGAAACATCTGCATTTGCTTTAACTGCCGGCGTGTCGGTGTGGAAC
GCCTTTGTTTTCGAGATTGTAATGACCTTTGGCCTGGTTTACACTGTGTACGCCACCGCTGTTGACCAAAAGAAG
GGAGAAATTGGCATTATTGCACCAATTGCAATTGGTTTCATCGTGGGTGCCAATATTCTGGCGGGTGGTGCATTT
ACCGGTGCTTCAATGAACCCTGCAGTATCATTTGGTCCAGCTCTGGTCAGCTTCACCTGGACCCATCACTGGGTG
TACTGGGCTGGACCTCTTATTGGTGGTGGCCTTGCTGGTATTGTTTATGAAGTCTTCTTCATCAACCATTCTCAC
GAACCCTTGCCAGGTTCAGAGTAC**TGA**
>*Oleur*TIP1;3c
MSSEFFNVPLRRVAFGHRDEFRQPGAIKAALAEFFSTLIFVFAGEGSGMAYNKLTGNAPTSPSGLVAAAVAHGFA
LFVAVSVSANISGGHVNPAVTFGLFLGGNVSLFRGILYIIAQLLGSVVACLLLLFSTGGLETSAFALTAGVSVWN
AFVFEIVMTFGLVYTVYATAVDQKKGEIGIIAPIAIGFIVGANILAGGAFTGASMNPAVSFGPALVSFTWTHHWV
YWAGPLIGGGLAGIVYEVFFINHSHEPLPGSEY*

***Oleur*TIP1;3d Oleur061Scf0466g03012.1**

>Oleur061Scf0466:372836..374085 (- strand) class=gene length=1250

>*Oleur*TIP1;3d
ATGTCTAGCGAGTACTTTAACGTCCTGTTCCGCCATGTTGCCATCGGAAATCAAGATGATTTCCGCCAGGCCGGC
GCTATCAAGGCGGCGCTGGCTGAGTTCTTCAGTACCCTTATTTTTGTGTTCGCCGGTGAGGGTTCCGGCATGGCA
TACAACAAGTTGACTTGGAATGCTCCTACCTCCCCCTCCGGCCTTGTGGTAGCGGCGGTAGCTCATGCTTTTGGT
TTGTTTGTGGCTGTTTCTATCAGTGCTAACATTTCCGGCGGTCATGTCAACCCTGCCGTCACTTTCGGTTTGTTT
CTTGGTGGAAACATCTCTTTGTTCAGAGGTATCCTTTACATCATCGCTCAATTGCTGGGATCTGTTGTGGCTTGT
CTTCTCCTTTTGTTCTCAACTGGTGGACTGGTAATTTTTAGAAAGAACATTAATTTTTTATCAACTTAGGTACGT
GTTTAATAATTGTGACTCCTATTTATCTACTGAAACTTTGATGCAGGAAACATCTGCATTTGCTTTGGGTGCCAA
CGTGTCTGTGTGGAACGCCTTTGTATTTGAGATTGTGATGACTTTTGGCCTGGTTTACACTGTGTACGCCACCGC
GATTGACCCGAAGAAGGGAGAAGTTGGCATTATTGCACCAATTGCAATTGGTTTCATTGTGGGGGCCAACATTCT
TGCTGGTGGTGCATTTACTGGTGCTTCAATGAACCCTGCAGTGTCATTTGGCCCTGCTCTGGTTAGCTTCACCTG
GACCCATCACTGGGTATACTGGGCTGGACCTCTAATTGGTGGGGGACTTGCTGGTATTGTTTATGAAGTCTTCTT
CATCAGTCATTCTCACGAGCCACTGCCCGGATCGGAGTAC**TGA**
>*Oleur*TIP1;3d
ATGTCTAGCGAGTACTTTAACGTCCTGTTCCGCCATGTTGCCATCGGAAATCAAGATGATTTCCGCCAGGCCGGC
GCTATCAAGGCGGCGCTGGCTGAGTTCTTCAGTACCCTTATTTTTGTGTTCGCCGGTGAGGGTTCCGGCATGGCA
TACAACAAGTTGACTTGGAATGCTCCTACCTCCCCCTCCGGCCTTGTGGTAGCGGCGGTAGCTCATGCTTTTGGT
TTGTTTGTGGCTGTTTCTATCAGTGCTAACATTTCCGGCGGTCATGTCAACCCTGCCGTCACTTTCGGTTTGTTT

CTTGGTGGAAACATCTCTTTGTTCAGAGGTATCCTTTACATCATCGCTCAATTGCTGGGATCTGTTGTGGCTTGT
CTTCTCCTTTTGTTCTCAACTGGTGGACTGGAAACATCTGCATTTGCTTTGGGTGCCAACGTGTCTGTGTGGAAC
GCCTTTGTATTTGAGATTGTGATGACTTTTGGCCTGGTTTACACTGTGTACGCCACCGCGATTGACCCGAAGAAG
GGAGAAGTTGGCATTATTGCACCAATTGCAATTGGTTTCATTGTGGGGGCCAACATTCTTGCTGGTGGTGCATTT
ACTGGTGCTTCAATGAACCCTGCAGTGTCATTTGGCCCTGCTCTGGTTAGCTTCACCTGGACCCATCACTGGGTA
TACTGGGCTGGACCTCTAATTGGTGGGGGACTTGCTGGTATTGTTTATGAAGTCTTCTTCATCAGTCATTCTCAC
GAGCCACTGCCCGGATCGGAGTAC**TGA**

>*Oleur*TIP1;3d

MSSEYFNVLFRHVAIGNQDDFRQAGAIKAALAEFFSTLIFVFAGEGSGMAYNKLTWNAPTSPSGLVVAAVAHAFG
LFVAVSISANISGGHVNPAVTFGLFLGGNISLFRGILYIIAQLLGSVVACLLLLFSTGGLETSAFALGANVSVWN
AFVFEIVMTFGLVYTVYATAIDPKKGEVGIIAPIAIGFIVGANILAGGAFTGASMNPAVSFGPALVSFTWTHHWV
YWAGPLIGGGLAGIVYEVFFISHSHEPLPGSEY*

*Oleur*TIP1;3e Oleur061Scf3806g00010.1

>Oleur061Scf3806:21034..22285 (+ strand) class=gene length=1252

>*Oleur*TIP1;3e

ATGTCTAGCGAGTACTTTAACGTCCCGTTCCGCCATGTTGCCATCGGAAATCAAGATGATTTCCGCCAGCCCGGC
GCTATCAAGGCTGCGCTGGCTGAGTTCTTCAGTACCCTTATTTTTGTGTTTGCCGGTGAGGGTTCCGGCATGGCA
TACAACAAGTTGACTTGGAACGCTCCTACCTCCCCCTCCGGCCTTGTGGTAGCGGCGGTAGCTCATGCTTTTGGT
TTGTTTGTGGCTGTTTCTATCAGTGCTAACATTTCCGGCGGTCATGTCAACCCTGCCGTCACTTTCGGTTTGTTT
CTTGGAGGAAACATCTCTTTGTTCAGAGGTATCCTTTACATCATCGCTCAGTTGCTGGGATCTGTTGTTGCTTGT
CTTCTCCTTTTGTTCTCAACTGGTGGACTGGTAATTTTTAGAAAGAACATTAATTTTTTATCAACTTAGGTACGT
GTTTAATAATTGTGACTCCTATTTATCTACTGAAACTTTGATGCAGGAAACATCTGCATTTGCTTTGGGTGCCAA
CGTGTCTGTGTGGAACGCCTTTGTATTCGAGATTGTGATGACTTTTGGCCTGGTTTACACTGTGTACGCCACCGC
GATTGACCCGAAGAAGGGAGAAGTTGGCATTATTGCACCAATTGCAATTGGTTTCATTGTGGGTGCCAACATTCT
TGCTGGTGGGGCATTTACTGGTGCTTCAATGAACCCTGCAGTGTCATTTGGCCCTGCTCTGGTTAGCTTCACCTG
GACCCATCACTGGGTATACTGGGCTGGACCTCTAATTGGTGGTGGACTTGCTGGTATTGTTTATGAAGTCTTCTT
CATCAGTCATTCTCACGAGCCACTGCCCGGATCGGAGTAC**TGA**

>*Oleur*TIP1;3e

ATGTCTAGCGAGTACTTTAACGTCCCGTTCCGCCATGTTGCCATCGGAAATCAAGATGATTTCCGCCAGCCCGGC
GCTATCAAGGCTGCGCTGGCTGAGTTCTTCAGTACCCTTATTTTTGTGTTTGCCGGTGAGGGTTCCGGCATGGCA
TACAACAAGTTGACTTGGAACGCTCCTACCTCCCCCTCCGGCCTTGTGGTAGCGGCGGTAGCTCATGCTTTTGGT
TTGTTTGTGGCTGTTTCTATCAGTGCTAACATTTCCGGCGGTCATGTCAACCCTGCCGTCACTTTCGGTTTGTTT
CTTGGAGGAAACATCTCTTTGTTCAGAGGTATCCTTTACATCATCGCTCAGTTGCTGGGATCTGTTGTTGCTTGT
CTTCTCCTTTTGTTCTCAACTGGTGGACTGGAAACATCTGCATTTGCTTTGGGTGCCAACGTGTCTGTGTGGAAC
GCCTTTGTATTCGAGATTGTGATGACTTTTGGCCTGGTTTACACTGTGTACGCCACCGCGATTGACCCGAAGAAG
GGAGAAGTTGGCATTATTGCACCAATTGCAATTGGTTTCATTGTGGGTGCCAACATTCTTGCTGGTGGGGCATTT
ACTGGTGCTTCAATGAACCCTGCAGTGTCATTTGGCCCTGCTCTGGTTAGCTTCACCTGGACCCATCACTGGGTA
TACTGGGCTGGACCTCTAATTGGTGGTGGACTTGCTGGTATTGTTTATGAAGTCTTCTTCATCAGTCATTCTCAC
GAGCCACTGCCCGGATCGGAGTAC**TGA**

>*Oleur*TIP1;3e

MSSEYFNVPFRHVAIGNQDDFRQPGAIKAALAEFFSTLIFVFAGEGSGMAYNKLTWNAPTSPSGLVVAAVAHAFG
LFVAVSISANISGGHVNPAVTFGLFLGGNISLFRGILYIIAQLLGSVVACLLLLFSTGGLETSAFALGANVSVWN
AFVFEIVMTFGLVYTVYATAIDPKKGEVGIIAPIAIGFIVGANILAGGAFTGASMNPAVSFGPALVSFTWTHHWV
YWAGPLIGGGLAGIVYEVFFISHSHEPLPGSEY*

*Oleur*TIP1;5 Oleur061Scf7653g04013.1

>Oleur061Scf7653:468560..470420 (- strand) class=gene length=1861

>*Oleur*TIP1;5

ATGCCGATTTCGAGAATAGCTGTCGGATCTCTGGCCGAGGCTAGTCAGCCGGAAGCCCTCAAGGCCACCCTGGCC
GAGTTCATTTCGATGTTGATTTTTGTTTTTGCTGGTGAAGGTTCTGGCATGGCTTTCGGTAAGTAGTTATCCTGT
ACAAATTCATTTAAAAGTTTAAGTTGTTAGAATTCGAGTATTAAATCATCGGGATTTTGACTTTTTCCACAGCAA
AGCTTACAAACAATGGATCGACCACGCCAACTGGCCTAATTGCTGAGGCGATAGCGCACGCTTTTGCACTTTTCG
TGGCGGTTTCGGTCGGTGCTAACATCTCTGGCGGCCACGTAAACCCTGCTGTCACCTTTGGTGCCTTTGTTGGTG
GTCACATCACATTGATTAGGAGCATCCTGTACTGGATAGCCCAATTACTTGGATCTGTTGTTGCATGCTTGCTTC
TTAGGTTAGCCACAGGCGGACTCGTAAGTTGAATCTTACTGTACTATCCAGTCAAGTCGAGTCCATAACTATAAC
AAATAGGCTAAAGTTCAATTCTTGTTTACAGGAAACATCAGCTTTTGCACTTTCATCTGGAGTGACAGTTTGGAA

TGCAGTTGTTTTTGAGATAGTGATGACCTTTGGACTCGTTTACACAGTTTATGCTACTGCAGTGGATCCAAAGAG
GGGTAACTTAGGGATCATTGCCCCCATTGCCATTGGCTTCATTGTAGGCGCTAACATCTTAGCCGGTGGTGCCTT
CGATGGTGCGTCCATGAACCCCGCCGTGTCTTTTGGCCCTGCTGTGGTCAGCTGGACATGGGACAGCCACTGGGT
CTTCTGGCTCGGCCCGTTTGTCGGTGCTGGAATTGCTGCTCTGATCTATGAAATCTTGTTCATTAACCAAAGCCA
TGAACAACTCCGTACTAGTGCAGAATAC**TAA**

>*Oleur*TIP1;5

ATGCCGATTTCGAGAATAGCTGTCGGATCTCTGGCCGAGGCTAGTCAGCCGGAAGCCCTCAAGGCCACCCTGGCC
GAGTTCATTTCGATGTTGATTTTTGTTTTTGCTGGTGAAGGTTCTGGCATGGCTTTCGCAAAGCTTACAAACAAT
GGATCGACCACGCCAACTGGCCTAATTGCTGAGGCGATAGCGCACGCTTTTGCACTTTTCGTGGCGGTTTCGGTC
GGTGCTAACATCTCTGGCGGCCACGTAAACCCTGCTGTCACCTTTGGTGCCTTTGTTGGTGGTCACATCACATTG
ATTAGGAGCATCCTGTACTGGATAGCCCAATTACTTGGATCTGTTGTTGCATGCTTGCTTCTTAGGTTAGCCACA
GGCGGACTCGAAACATCAGCTTTTGCACTTTCATCTGGAGTGACAGTTTGGAATGCAGTTGTTTTTGAGATAGTG
ATGACCTTTGGACTCGTTTACACAGTTTATGCTACTGCAGTGGATCCAAAGAGGGGTAACTTAGGGATCATTGCC
CCCATTGCCATTGGCTTCATTGTAGGCGCTAACATCTTAGCCGGTGGTGCCTTCGATGGTGCGTCCATGAACCCC
GCCGTGTCTTTTGGCCCTGCTGTGGTCAGCTGGACATGGGACAGCCACTGGGTCTTCTGGCTCGGCCCGTTTGTC
GGTGCTGGAATTGCTGCTCTGATCTATGAAATCTTGTTCATTAACCAAAGCCATGAACAACTCCGTACTAGTGCA
GAATAC**TAA**

>*Oleur*TIP1;5

MPISRIAVGSLAEASQPEALKATLAEFISMLIFVFAGEGSGMAFAKLTNNGSTTPTGLIAEAIAHAFALFVAVSV
GANISGGHVNPAVTFGAFVGGHITLIRSILYWIAQLLGSVVACLLLRLATGGLETSAFALSSGVTVWNAVVFEIV
MTFGLVYTVYATAVDPKRGNLGIIAPIAIGFIVGANILAGGAFDGASMNPAVSFGPAVVSWTWDSHWVFWLGPFV
GAGIAALIYEILFINQSHEQLRTSAEY*

*Oleur*TIP1;6 Oleur061Scf5512g00068.1

>Oleur061Scf5512:141526..147261 (- strand) class=gene length=5736

>*Oleur*TIP1;6

ATGCCGATTTATCGGATTGCGATTGGGACGCCTGGAGAAGCGAGCCACCCCGATGCACTCAAGGCCGCGCTTGCT
GAGTTCTTCTCTATGCTTATTTTTGTTTTTGCTGGTCAAGGCTCCGGGATGGCTTTTAGTACGTTCGCTCTTTCT
CCTCTACATCTATTTATATTCCGTTGATACGAAACAATGATTTGAATTAATGGAGACTCTAGGAATCCCACTTTC
TTCATTTGATATTTGATGGGCTAGTGTGAGATTATCGGATAATATTTTTTCTTAAAGATCCTGGCTTAATCTTCC
CTGGAAAGACACGTATGAGATCTTCAATTTTTGGGAGTTTTTCTATGCGATCATTTTTCAATCTTCTTGTTGCTG
GTATCTCATTCGTGTACATTATTTCTTTATTTTTCGATCTTCTAACGTAAAACGTTTGATGATTGTATCATATTA
TGGCAGATAAGTTGAGCAATGATGGGTCACCAAATCCATCAGGACTGCTAGCTGCAGCATTAGCCCATGCATTTG
CACTTTTTGTGGCAGTCTCAGTAGGTGCAAACATATCAGGGGGTCATGTGAATCCTGCTGTCACGTTTGGTGCTT
TCATTGGTGGGAACATAACACTTTTGAGGAGCATTTTGTACTGGATTGCTCAATTACTTGGCTCTGTTGTTGCCT
GCATACTTCTTAAGTTTGCTACTGGAGGATTGGTAAGAAAGTTGATCGTTTTACGACAAATTTTGCTTCCAAACT
ACGAAAATTTTTGGTGTAAAAAATCATTGATTGTTTCGTAAAAATAGGGTCGTTGAGAGAACTTCTTTTCGTTGT
TAAATGTGAAATTTCTTGTAGTAGCTTAATTCAGTGCATTAATCAACAATGATTTGCTTCCGTTACTGGAATTTT
ACAAATTTTAGTCTTTTCATTGTCTGAACTTAGAATATATGTAAGAATTTATACATGTTTGGAGAAAAAGGAGAA
CAAGATAGATGGCGATGCCCTCCCAAGTAAATCTAGACATACCTGAGTATTAGAGAATATACAATTCATGTCTAG
ATGGTTTGAAATTCAAACAAAAATAGAAATGGAAAATTTGGTAAAAATTTGAAGAGCCCATCTCATTTCCTCGTC
TTTTACTCGTGTAAGTAAATAATAAGGCTTTAGGGCATTTATACACTTTCTTAAACATAACATTACCTCCCCAAT
CCAAGAGACAAGTTATCTTGAGGAGTATGCAACAAACCCTCAATACTTTCTATCATAAACTTGTAATAGATAATT
AAATATGTATTTATTGTAATTATTTATGCAAGTGAAAATAAATGTGGCAAAATATATTGAAGTACCAAGTAAAAA
GTTATACATTCTAATCTTTCCTTCCTCTCTTTTGACGTGGGACCTTGTTTATTTCAATTTTATGACAATCTACTT
CAACATGTACGTTCACGATTTTCGTGGGATGAAATCGACATATGAGCCAACTTATATATTGCAGGAAACATCAGC
ATTTGCCTTGTCGTCCGGTGTTTCTGTGTGGAACGCCCTAGTTTTTGAGATTGTGATGACCTTCGGGCTGGTGTA
TACGGTGTATGCTACTGCAGTGGATCCAAAAAAGGGTAATTTGGGAAACATTGCACCCATTGCAATTGGGTTCAT
TGTGGGTGCCAATATCTTAGCAGGTGGTCCATTTGATGGTGCATCAATGAACCCAGCAGTCTCCTTTGGACCAGC
TGTGGTCAGTTGGAATTTTACAAACCATTGGGTGTATTGGATAGGCCCCTTCATTGGTGCCACTATTGCAGCACT
TGTCTATGATAACATCTACATCGATGGTGAGACACATGAGCAGCTTCCAGTTACAGACTAT**TAG**

>*Oleur*TIP1;6

ATGCCGATTTATCGGATTGCGATTGGGACGCCTGGAGAAGCGAGCCACCCCGATGCACTCAAGGCCGCGCTTGCT
GAGTTCTTCTCTATGCTTATTTTTGTTTTTGCTGGTCAAGGCTCCGGGATGGCTTTTAATAAGTTGAGCAATGAT
GGGTCACCAAATCCATCAGGACTGCTAGCTGCAGCATTAGCCCATGCATTTGCACTTTTTGTGGCAGTCTCAGTA
GGTGCAAACATATCAGGGGGTCATGTGAATCCTGCTGTCACGTTTGGTGCTTTCATTGGTGGGAACATAACACTT
TTGAGGAGCATTTTGTACTGGATTGCTCAATTACTTGGCTCTGTTGTTGCCTGCATACTTCTTAAGTTTGCTACT
GGAGGATTGGAAACATCAGCATTTGCCTTGTCGTCCGGTGTTTCTGTGTGGAACGCCCTAGTTTTTGAGATTGTG
ATGACCTTCGGGCTGGTGTATACGGTGTATGCTACTGCAGTGGATCCAAAAAAGGGTAATTTGGGAAACATTGCA
CCCATTGCAATTGGGTTCATTGTGGGTGCCAATATCTTAGCAGGTGGTCCATTTGATGGTGCATCAATGAACCCA

GCAGTCTCCTTTGGACCAGCTGTGGTCAGTTGGAATTTTACAAACCATTGGGTGTATTGGATAGGCCCCTTCATT
GGTGCCACTATTGCAGCACTTGTCTATGATAACATCTACATCGATGGTGAGACACATGAGCAGCTTCCAGTTACA
GACTAT**TAG**

***>Oleur*TIP1;6**
MPIYRIAIGTPGEASHPDALKAALAEFFSMLIFVFAGQGSGMAFNKLSNDGSPNPSGLLAAALAHAFALFVAVSV
GANISGGHVNPAVTFGAFIGGNITLLRSILYWIAQLLGSVVACILLKFATGGLETSAFALSSGVSVWNALVFEIV
MTFGLVYTVYATAVDPKKGNLGNIAPIAIGFIVGANILAGGPFDGASMNPAVSFGPAVVSWNFTNHWVYWIGPFI
GATIAALVYDNIYIDGETHEQLPVTDY*

*Oleur*TIP1;7 Oleur061Scf3360g04010.1

>Oleur061Scf3360:442951..444443 (- strand) class=gene length=1493

***>Oleur*TIP1;7**
ATGCCGGTCAGCAGGATAGCGGTGGGGAGGCTGGAAGAGGCGACTCAGCAGGACGCCTTGAAGGCGGCATTGGCT
GAGTTCATATCAACCCTAATATTTGTGTTTGCTGGCTCAGGTTCAGGCATGGCCTTCAACAAGCTAACAGACGAC
GGAGCCACCACTCCGGCAGGCCTCATTGCAGCTGCAGTGGCCCATGCATTTGCACTTTTCGTGGCTGTTTCCGTT
GGTGCCAACATTTCCGGTGGCCATGTTAACCCTGCTGTCACTTTTGGAGCCTTTGTCGGTGGAAATATTAGCCTT
ATCCGTGGTATCCTCTACTGGATTGCTCAGTTGCTTGGTTCTGTCGTTGCTTGCTTGCTTCTCAAGTTCGCCACC
GGTGGTTTGGTGAGTTTTTCGCCCTCTTGCGTGAAAATTCTGTCTCTGTTTTACAATAATCACTTCGTCTAAAAG
CTCAAGTTAACGAAAAACATTCACCCCATTTGTAATTTTATTTTTCAGACTACTGCAGCCTTTTCATTGTCAGCT
GGAGTCAACGTATGGAATGCATTTGTATTAGAAATTGTGATGACCTTTGGGCTAGTCTACACCGTGTACGCCACC
GCAGTGGATCCAAAGAGGGGCAGTTTGGGAACAATTGCACCAATTGCTATTGGTTTCATTGTGGGTGCCAACATC
TTAGCTGGTGGGGCCTTTGATGGGGCCTCAATGAACCCAGCAGTGTCATTCGGCCCAGCTGTGGTGAGCTGGACC
TGGGACAGCCACTGGGTCTATTGGGCCGGGCCTCTAATTGGTGGTGGGCTTGCTGGGCTTATCTATGAGTTCTTC
TTCATTAACCAAACCCATGAACAGTTGCCTACAACTGATCAT**TAA**
***>Oleur*TIP1;7**
ATGCCGGTCAGCAGGATAGCGGTGGGGAGGCTGGAAGAGGCGACTCAGCAGGACGCCTTGAAGGCGGCATTGGCT
GAGTTCATATCAACCCTAATATTTGTGTTTGCTGGCTCAGGTTCAGGCATGGCCTTCAACAAGCTAACAGACGAC
GGAGCCACCACTCCGGCAGGCCTCATTGCAGCTGCAGTGGCCCATGCATTTGCACTTTTCGTGGCTGTTTCCGTT
GGTGCCAACATTTCCGGTGGCCATGTTAACCCTGCTGTCACTTTTGGAGCCTTTGTCGGTGGAAATATTAGCCTT
ATCCGTGGTATCCTCTACTGGATTGCTCAGTTGCTTGGTTCTGTCGTTGCTTGCTTGCTTCTCAAGTTCGCCACC
GGTGGTTTGACTACTGCAGCCTTTTCATTGTCAGCTGGAGTCAACGTATGGAATGCATTTGTATTAGAAATTGTG
ATGACCTTTGGGCTAGTCTACACCGTGTACGCCACCGCAGTGGATCCAAAGAGGGGCAGTTTGGGAACAATTGCA
CCAATTGCTATTGGTTTCATTGTGGGTGCCAACATCTTAGCTGGTGGGGCCTTTGATGGGGCCTCAATGAACCCA
GCAGTGTCATTCGGCCCAGCTGTGGTGAGCTGGACCTGGGACAGCCACTGGGTCTATTGGGCCGGGCCTCTAATT
GGTGGTGGGCTTGCTGGGCTTATCTATGAGTTCTTCTTCATTAACCAAACCCATGAACAGTTGCCTACAACTGAT
CAT**TAA**
***>Oleur*TIP1;7**
MPVSRIAVGRLEEATQQDALKAALAEFISTLIFVFAGSGSGMAFNKLTDDGATTPAGLIAAAVAHAFALFVAVSV
GANISGGHVNPAVTFGAFVGGNISLIRGILYWIAQLLGSVVACLLLKFATGGLTTAAFSLSAGVNVWNAFVLEIV
MTFGLVYTVYATAVDPKRGSLGTIAPIAIGFIVGANILAGGAFDGASMNPAVSFGPAVVSWTWDSHWVYWAGPLI
GGGLAGLIYEFFFINQTHEQLPTTDH*

*Oleur*TIP1;7b-related Oleur061Scf3142g00008.1

>Oleur061Scf3142:34430..35603 (+ strand) class=gene length=1174
Observation : Pseudognene. Deletion of 13n with OleurTIP1;7

***>Oleur*TIP1;7b**
ATGCCGGTCAGCAGGATAGCGGTGGGGAGGCTGGAAGAGGCGACTCAGCAGGACGCCTTGAAGGCGGCATTGGCT
GAGTTCATATCAACCCTAATATTTGTGTTTGCTGGCTCAGGTTCAGGCATGGCCTTCAACAAGCTAACAGACGAC
GGAGCCACCACTCCGGCAGGCCTCATTGCAGCTGCAGTGGCCCATGCATTTGCACTTTTCGTGGCTGTTTCCGTT
GGTGCCAACATTTCCGGTGGCCATGTTAACCCTGCTGTCTTGTCGGTGGAAATATTAGCCTTATCCGTGGTATCC
TCTACTGGATTGCTCAGTTGCTTGGTTCTGTCGTTGCTTGCTTGCTTCTCAAGTTCGCCACCGGTGGTTTGGTGA
GTTTTTCGCCCTCTTGCGTGAAAATTCTGTCTCTGTTTTACAATAATCACTTCGTCTAAAAGCTCAAGTTAACGA
AAAACATTCACCCCATTTGTAATTTTATTTTTCAGACTACTGCAGCCTTTTCATTGTCAGCTGGAGTCAACGTAT
GGAATGCATTTGTATTAGAAATTGTGATGACCTTTGGGCTAGTCTACACCGTGTACGCCACCGCAGTGGATCCAA
AGAGGGGCAGTTTGGGAACAATTGCACCAATTGCTATTGGTTTCATTGTGGGTGCCAACATCTTAGCTGGTGGGG

CCTTTGATGGGGCCTCAATGAACCCAGCAGTGTCATTCGGCCCAGCTGTGGTGAGCTGGACCTGGGACAACCACT
GGGTCTATTGGGCCGGGCCTCTAATTGGTGGTGGGCTTGCTGGGCTTATCTATGAGTTCTTCTTCATTAACCAAA
CCCATGAACAGTTGCCTACAACTGATCAT**TAA**

>*Oleur*TIP1;7b

ATGCCGGTCAGCAGGATAGCGGTGGGGAGGCTGGAAGAGGCGACTCAGCAGGACGCCTTGAAGGCGGCATTGGCT
GAGTTCATATCAACCCTAATATTTGTGTTTGCTGGCTCAGGTTCAGGCATGGCCTTCAACAAGCTAACAGACGAC
GGAGCCACCACTCCGGCAGGCCTCATTGCAGCTGCAGTGGCCCATGCATTTGCACTTTTCGTGGCTGTTTCCGTT
GGTGCCAACATTTCCGGTGGCCATGTTAACCCTGCTGTCTTGTCGGTGGAAATATTAGCCTTATCCGTGGTATCC
TCTACTGGATTGCTCAGTTGCTTGGTTCTGTCGTTGCTTGCTTGCTTCTCAAGTTCGCCACCGGTGACTACTGCA
GCCTTTTCATTGTCAGCTGGAGTCAACGTATGGAATGCATTTGTATTAGAAATTGTGATGACCTTTGGGCTAGTC
TACACCGTGTACGCCACCGCAGTGGATCCAAAGAGGGGCAGTTTGGGAACAATTGCACCAATTGCTATTGGTTTC
ATTGTGGGTGCCAACATCTTAGCTGGTGGGGCCTTTGATGGGGCCTCAATGAACCCAGCAGTGTCATTCGGCCCA
GCTGTGGTGAGCTGGACCTGGGACAACCACTGGGTCTATTGGGCCGGGCCTCTAATTGGTGGTGGGCTTGCTGGG
CTTATCTATGAGTTCTTCTTCATTAACCAAACCCATGAACAGTTGCCTACAACTGATCAT**TAA**

>OleurTIP1;7b

MPVSRIAVGRLEEATQQDALKAALAEFISTLIFVFAGSGSGMAFNKLTDDGATTPAGLIAAAVAHAFALFVAVSV
GANISGGHVNPAVLSVEILALSVVSSTGLLSCLVLSLLACFSSSPPVTTAAFSLSAGVNVWNAFVLEIVMTFGLV
YTVYATAVDPKRGSLGTIAPIAIGFIVGANILAGGAFDGASMNPAVSFGPAVVSWTWDNHWVYWAGPLIGGGLAG
LIYEFFFINQTHEQLPTTDH*

*Oleur*TIP2;1 Oleur061Scf1395g09059.1

>Oleur061Scf1395:971893..975231 (+ strand) class=gene length=3339

>*Oleur*TIP2;1

ATGCCTGCAATTGCTGTCGGAAGATTAGATGATTCATTTAGCATTGGTTCGTTGAAGGCCTATGTAGCTGAATTC
ATCTCCACCTTGCTCTTTGTTTTCGCCGGAGTCGGCTCCGCCATTGCCTACAGTTAGTTCTAAAACCGTTTTCTT
ATTTTTTTAAGTTAACAAGCACTTGGTAACATTTGATCTTTGACAGACAAGTTGACGGCGGACGCAGCTCTTG
ATCCGGCGGGGCTGGTGGCTGTGGCCATCTGCCATGGTTTCGCTCTGTTTGTAGCCGTCTCGATCGGAGCCAACA
TCTCCGGTGGCCATGTAAACCCTGCAGTCACCTTTGGTTTGGCTGTTGGCGGCCAAATTACGCTACTTACTGGCC
TCTTTTACTGGATTGCTCAACTTTTGGGCTCCATTGTGGCATGCTATTTACTCGTATTCGTAACCGGAGGATTGG
TATGTCCACCCAAAATAATATTTTAAAATTCGAGTCGGTTTTTATTTACAGAGTTTTTATAGTCCGATTCTTGAT
TTCTCTGGCTAAACTACCCCGATTAGTTCGATTCCTAACAAAATATATAATTTCATGCAGGCTATTCCAATTCAT
GGAGTTGGTGCTGGTGTTGGAGCCTTTGAAGGAGTTGTTATGGAAATTGTTATCACATTTGCTTTAGTCTATACT
GTGTATGCGACAGCAGCTGATCCAAAGAAGGGCTCATTGGGCACAATTGCACCCATAGCCATTGGTTTCATTGTT
GGTGCCAATATTTTGGCTGCCGGTCCATTTTCAGGTGGGTCGATGAACCCTGCCCGCTCCTTCGGACCGGCTGTG
GCCAGCGGCAACTTCTCCGGCATCTGGATTTACTGGGTTGGACCCCTCATTGGTGGTGGCCTCGCCGGTCTCGTC
TACGCCTATGTGTTCATGTCCCACGAGCATGCTCCCCTACCTACCGACTTT**TAA**

>*Oleur*TIP2;1

ATGCCTGCAATTGCTGTCGGAAGATTAGATGATTCATTTAGCATTGGTTCGTTGAAGGCCTATGTAGCTGAATTC
ATCTCCACCTTGCTCTTTGTTTTCGCCGGAGTCGGCTCCGCCATTGCCTACAACAAGTTGACGGCGGACGCAGCT
CTTGATCCGGCGGGGCTGGTGGCTGTGGCCATCTGCCATGGTTTCGCTCTGTTTGTAGCCGTCTCGATCGGAGCC
AACATCTCCGGTGGCCATGTAAACCCTGCAGTCACCTTTGGTTTGGCTGTTGGCGGCCAAATTACGCTACTTACT
GGCCTCTTTTACTGGATTGCTCAACTTTTGGGCTCCATTGTGGCATGCTATTTACTCGTATTCGTAACCGGAGGA
TTGGCTATTCCAATTCATGGAGTTGGTGCTGGTGTTGGAGCCTTTGAAGGAGTTGTTATGGAAATTGTTATCACA
TTTGCTTTAGTCTATACTGTGTATGCGACAGCAGCTGATCCAAAGAAGGGCTCATTGGGCACAATTGCACCCATA
GCCATTGGTTTCATTGTTGGTGCCAATATTTTGGCTGCCGGTCCATTTTCAGGTGGGTCGATGAACCCTGCCCGC
TCCTTCGGACCGGCTGTGGCCAGCGGCAACTTCTCCGGCATCTGGATTTACTGGGTTGGACCCCTCATTGGTGGT
GGCCTCGCCGGTCTCGTCTACGCCTATGTGTTCATGTCCCACGAGCATGCTCCCCTACCTACCGACTTT**TAA**

>*Oleur*TIP2;1

MPAIAVGRLDDSFSIGSLKAYVAEFISTLLFVFAGVGSAIAYNKLTADAALDPAGLVAVAICHGFALFVAVSIGA
NISGGHVNPAVTFGLAVGGQITLLTGLFYWIAQLLGSIVACYLLVFVTGGLAIPIHGVGAGVGAFEGVVMEIVIT
FALVYTVYATAADPKKGSLGTIAPIAIGFIVGANILAAGPFSGGSMNPARSFGPAVASGNFSGIWIYWVGPLIGG
GLAGLVYAYVFMSHEHAPLPTDF*

*Oleur*TIP2;3a Oleur061Scf5616g00030.1

>Oleur061Scf5616:47019..49189 (- strand) class=gene length=2171

>*Oleur*TIP2;3a

ATGCCAACCATTGCCTTCGGACGAATCGATGATTCATTAAGCGTTGGCTCTTTGAAGGCCTATTTAGCCGAATTC
ATCTCGACTTTGCTCTTCGTCTTCGCTGGAGTCGGCTCAGCCATTGCTTACAGTTAGTCATCACTTCTTTTTCCT
TTTAATAAAGTAAGATTTGATGCAGCACAATTCACACGAATCCCTCTCCCTGTACAAATCATTCAGAGGGAGGAA
AATACGCAGAATTCTTCGTTAGATCGGATCTATTAGACTCATTCTTTGGTGTTTCTCACTCTTTCACACACGCTA
GGTGTGAGAGATTGATGCATAAAATAAAATTAACCCTAGCCATGCAATGCAGCTTGTGATTATAAATGTTGGTTT
CGTATACAGTTCTAATTTGTGGGTATGTTTGATATTCTATTTCGACAGACAAGTTGACATATGATGCGGCTCTCG
ACCCAGCGGGGCTGGTAGCGGTGGCGGTCTGCCACGGATTTGCTCTGTTTGTGGCGGTTTCGATCGGAGCCAACA
TCTCCGGTGGCCATGTAAACCCTGCAGTCACCTTTGGTTTGGCTGTTGGTGGCCAAATTACAATTCTCACTGGCC
TCTTCTATTGGATTGCTCAACTTTTGGGCTCCATTGTAGCATGCTTCTTGCTCAAATTTGTCACAGGTGGCTTGG
TAAGTTGTTGTTCTATAATTATGTTATTCATGAATTCTTTTCTAACTTCCTTCAAATGTGAGAGAGGGGATAAGA
TTATGTCTTGATACGTCCCCATCACGTACATTCCTTTTTTTTTTTTTTTTTAATCAAAAGTTGACGCGTACACAA
TTTCCGTAAATAAATAATAAAGATTTTAAATCTCTGACTTTAAAGTTCTAATGCCATTTTGACTAAATCTAAAAG
TTCATATCTAATCTAAACATACATTCTCAAAAACTAAAACTGATTAATTGTACTCTTTTTCAACTTTCAACACAA
AGAATAGTAAATCTTCACCATGTGTAGTTCATATTCGTTTTGAATAAAAATGCTAACACGTGCACAATTTTCATA
AATGAATGACAACAAAATTTAAACTTGGTTCCTCTATGTGTTCTCTACAATTTTGACTCGATCTAAAAGTTTACA
ACTGATCTAAGCTTACAGTACAAATGAGAATTGTATTCTTTTTCGACTTTCAACACGTTATATTAAAGAATAATC
GATCTTGAATGATTTCATAAACAAGTGTCTAATTATGTTTGTAAATTTGATGATTAATTTCTATGTAGGCCATTC
CAACTCACAGTATGGCAGCTGGTGTTGGAGCCATTGAAGGAGTTGTGATGGAAATTGTTATCACTTTTGCTTTGG
TGTACACAGTTTATGCCACAGCAGTTGACCCAAAGAAGGGCTCATTAGGCACAATTGCCCCCATTGCCATTGGCT
TCATTGTTGGGGCCAACATTTTGGCCGCCGGCCCATTCTCCGGTGGCTCAATGAACCCGGCTAGGTCCTTCGGTC
CGGCTGTGGCCAGCGGTGACTTTGCCGACAACTGGATTTACTGGGTCGGTCCACTCATCGGTGGTGGTCTGGCTG
GACTCGTCTATGCCTATGTGTTCATGGTCCACGAACACGTTCCTCTTGTTAGCGAGTTC**TAA**

>*Oleur*TIP2;3a
ATGCCAACCATTGCCTTCGGACGAATCGATGATTCATTAAGCGTTGGCTCTTTGAAGGCCTATTTAGCCGAATTC
ATCTCGACTTTGCTCTTCGTCTTCGCTGGAGTCGGCTCAGCCATTGCTTACAACAAGTTGACATATGATGCGGCT
CTCGACCCAGCGGGGCTGGTAGCGGTGGCGGTCTGCCACGGATTTGCTCTGTTTGTGGCGGTTTCGATCGGAGCC
AACATCTCCGGTGGCCATGTAAACCCTGCAGTCACCTTTGGTTTGGCTGTTGGTGGCCAAATTACAATTCTCACT
GGCCTCTTCTATTGGATTGCTCAACTTTTGGGCTCCATTGTAGCATGCTTCTTGCTCAAATTTGTCACAGGTGGC
TTGGCCATTCCAACTCACAGTATGGCAGCTGGTGTTGGAGCCATTGAAGGAGTTGTGATGGAAATTGTTATCACT
TTTGCTTTGGTGTACACAGTTTATGCCACAGCAGTTGACCCAAAGAAGGGCTCATTAGGCACAATTGCCCCCATT
GCCATTGGCTTCATTGTTGGGGCCAACATTTTGGCCGCCGGCCCATTCTCCGGTGGCTCAATGAACCCGGCTAGG
TCCTTCGGTCCGGCTGTGGCCAGCGGTGACTTTGCCGACAACTGGATTTACTGGGTCGGTCCACTCATCGGTGGT
GGTCTGGCTGGACTCGTCTATGCCTATGTGTTCATGGTCCACGAACACGTTCCTCTTGTTAGCGAGTTC**TAA**

>*Oleur*TIP2;3a
MPTIAFGRIDDSLSVGSLKAYLAEFISTLLFVFAGVGSAIAYNKLTYDAALDPAGLVAVAVCHGFALFVAVSIGA
NISGGHVNPAVTFGLAVGGQITILTGLFYWIAQLLGSIVACFLLKFVTGGLAIPTHSMAAGVGAIEGVVMEIVIT
FALVYTVYATAVDPKKGSLGTIAPIAIGFIVGANILAAGPFSGGSMNPARSFGPAVASGDFADNWIYWVGPLIGG
GLAGLVYAYVFMVHEHVPLVSEF*

***Oleur*TIP2;3b Oleur061Scf5616g00030.1**

>Oleur061Scf6190:16438..18599 (+ strand) class=gene length=2162

>*Oleur*TIP2;3b
ATGCCAACCATTGCCTTCGGACGAATCGATGATTCATTAAGCGTTGGCTCTTTGAAGGCCTATTTAGCCGAATTC
ATCTCGACTTTGCTCTTCGTCTTCGCTGGAGTCGGCTCAGCCATTGCTTACAGTTAGTCATCACTTCTTTTTCCT
TTTAATAAAGTAAGATTTGATGCAGCACAATTCACACGAATCCCTCTCCCTGTACAAATCATTCAGAGGGAGGAA
AATACGCAGAATTCTTCGTTAGATCGGATCTATTAGACTCATTCTTTGGTGTTTCTCACTCTTTCACACACGCTA
GGTGTGAGAGATTGATGCATAAAATAAAATTAACCCTAGCCATGCAATGCAGCTTGTGATTATAAATGTTGGTTT
CGTATACAGTTCTAATTTGTGGGTATGTTTGATATTCTATTTCGACAGACAAGTTGACATATGATGCGGCTCTCG
ACCCAGCGGGGCTGGTAGCGGTGGCGGTCTGCCACGGATTTGCTCTGTTTGTGGCGGTTTCGATCGGAGCCAACA
TCTCCGGTGGCCATGTAAACCCAGCAGTCACCTTTGGTTTGGCTGTTGGTGGCCAAATTACAATTCTCACTGGCC
TCTTCTATTGGATTGCTCAACTTTTGGGCTCCATTGTAGCATGCTTCTTGCTCAAATTTGTCACAGGTGGCTTGG
TAAGTTGTTGTTCTATAATTATGTTATTCTTGAATTCTTTTCTAACTTCCTTCAAATGTGAGAGAGGGGATAAGA
TTATGTCTTGATACGTCCCCATCACGTACATTCCTTTTTTTTTTTTTTTTTAATCAAAAGTCTGACGCGTACACA
ATTTCCGTAAATAAATAATAAAGATTTTAAATCTCTGACTTTAAAGTTCTAATGCCATTTTGACTAAATCTAAAA
GTTCATATCTAATCTAAACATACATTCTCAAAAACTAAAACTGATTAATTGTACTCCTTTTCGACTTTCAACACA
AAGAATAGTAAATCTTCACCATGTGTAGTTCATATTCGTTTTGAATAAAAATGCTAACACGTGCACAATTTTCAT
AAATGAATGACGACAAAATTTGAACTTGGTTCCTCTATGTGTTCTCTACAATTTTGACTCGATCTAAAAGTTTAC
AACTGATCTAAGCTTACAGTACAAATGAGAATTGTATTCTTTTTCAACTTTCAACACGTTATATTAAAGAATAAT
CGATCTTGAATGATTTCATAAACAAGTGTCTAATTATGTTTGTAAATTTGATGATTAATTTCTATGTAGGCCATT
CCAACTCACAGTATGGCAGCTGGTGTTGGAGCCATTGAAGGAGTTGTGATGGAAATTGTTATCACTTTTGCTTTG

GTGTACACAGTTTATGCCACAGCAGTTGACCCAAAGAAGGGCTCATTAGGCACAATTGCCCCCATTGCCATTGGC
TTCATTGTTGGGGCCAACATTTTGGCCGCCGGCCCATTCTCCGGTGGCTCAATGAACCCGGCTAGGTCCTTCGGT
CCGGCTGTGGCCAGCGGTGACTTTGCCGACAACTGGATTTACTGGGTCGGTCCACTCATCGGTGGTGGTCTGGCT
GGACTCGTCTATGCCTATGTGTTCATGGTCCACGAACACGTTCCTCTTGTTAGCGAGTTC**TAA**

>*Oleur*TIP2;3b

ATGCCAACCATTGCCTTCGGACGAATCGATGATTCATTAAGCGTTGGCTCTTTGAAGGCCTATTTAGCCGAATTC
ATCTCGACTTTGCTCTTCGTCTTCGCTGGAGTCGGCTCAGCCATTGCTTACAACAAGTTGACATATGATGCGGCT
CTCGACCCAGCGGGGCTGGTAGCGGTGGCGGTCTGCCACGGATTTGCTCTGTTTGTGGCGGTTTCGATCGGAGCC
AACATCTCCGGTGGCCATGTAAACCCAGCAGTCACCTTTGGTTTGGCTGTTGGTGGCCAAATTACAATTCTCACT
GGCCTCTTCTATTGGATTGCTCAACTTTTGGGCTCCATTGTAGCATGCTTCTTGCTCAAATTTGTCACAGGTGGC
TTGGCCATTCCAACTCACAGTATGGCAGCTGGTGTTGGAGCCATTGAAGGAGTTGTGATGGAAATTGTTATCACT
TTTGCTTTGGTGTACACAGTTTATGCCACAGCAGTTGACCCAAAGAAGGGCTCATTAGGCACAATTGCCCCCATT
GCCATTGGCTTCATTGTTGGGGCCAACATTTTGGCCGCCGGCCCATTCTCCGGTGGCTCAATGAACCCGGCTAGG
TCCTTCGGTCCGGCTGTGGCCAGCGGTGACTTTGCCGACAACTGGATTTACTGGGTCGGTCCACTCATCGGTGGT
GGTCTGGCTGGACTCGTCTATGCCTATGTGTTCATGGTCCACGAACACGTTCCTCTTGTTAGCGAGTTC**TAA**

>*Oleur*TIP2;3b

MPTIAFGRIDDSLSVGSLKAYLAEFISTLLFVFAGVGSAIAYNKLTYDAALDPAGLVAVAVCHGFALFVAVSIGA
NISGGHVNPAVTFGLAVGGQITILTGLFYWIAQLLGSIVACFLLKFVTGGLAIPTHSMAAGVGAIEGVVMEIVIT
FALVYTVYATAVDPKKGSLGTIAPIAIGFIVGANILAAGPFSGGSMNPARSFGPAVASGDFADNWIYWVGPLIGG
GLAGLVYAYVFMVHEHVPLVSEF*

*Oleur*TIP2;4 Oleur061Scf2185g00004.1

>Oleur061Scf2185:106959..109328 (- strand) class=gene length=2370

>*Oleur*TIP2;4

ATGGTGAAGATGGCATTGGGTAGCATTGGTGACTCTTTTTCAGTTCCGTCGCTAAAGGCTTATCTTGCTGAGTTC
ATTGCCACCCTCCTCTTTGTCTTTGCTGGTGTAGGTTCCGCTATAGCTTACAGTAAGTACATATTTTCTGTTTTC
ATGAAGTGTGTATATTTTACGGTCGGCCCCCAAGCCCAGATATAGAATGAGGGTCAGGTTAGGTTAAGAGCGAGT
GTAAAACTTTATCAAAGCTTATGATATTAGTCGATTTCAAATTTATTGGAATTTGTGCAGACAAGCTCACGTCCG
ATGCAGCTCTCGACCCACCTGGATTGATGGCAGTAGCGGTGGCTCACGCATTTGCATTGTTTGTGGGCGTTTCCA
TGGCGGCCAATGTATCCGGCGGTCATCTGAATCCGGCCGTCACATTTGGACTGGCCATTGGAGGCAACATCACAA
TCATAACTGGCATTTTCTATTGGATTGCCCAGTTGCTTGGTTCCACTGTTGCATGCCTTCTCCTCAAATTCGTCA
CTAATGGCTTGGTAAGTCATTCCCAATACCAAAATTTAATCTTATTTTGACAGTAAAGCAATAAAATTACCACTG
CAGGAGCTTTCTTAATGGCCTCCGGAGGTCATCTCGTGACCATGGGGGCCTTCTGTGATTAAAAAGAAAAAAGAA
AAAGAAAAAGAAAAGTTACTCATCCTTTGGATTAATTGAATATGCGAGCTTATTCCATCGTTTGTGTTATACGAA
GTATCGAACCAAACATATCTCGAACTTAGAAGATTTACAGAAAAACTTAAAGAAATACACAACTTCCTTTCAAGC
TTTCTCAAACTGCTACCATGGCATCAAGAATGAAGATGTTTCAATGGGGCTCAGGACAAAAAACGAATTGTACCC
GTTTGAGTGACTAAAACCCTAATCCAGATTCTCAAGCGCGCCTTTATTGATTAAAGGAAGCCACAAGGCAGATGA
ATTCACACTACTTCTTATCAGTACACGAAGTATCAAGATTACACGAAGAATCAGAAAGAAATTCAACTTCCAATA
AAAAATTGGAAGATGAATACAAAAGAGATAGGAAGTGTCACAAAGCATATACACAAGGCGCAAACCCTAGGAATG
AAATAATAGGGCAACAAAAGTAGTATTTATGCACTGGCTTCCAACCATTGATCAAATTCCAATCTAACAGTCCAG
TTGGCTCCTCAAACAAAGAAGAAATCCCGTGAGTCTGCAACAATAAAAGTACCATGCCTTAAAACAAGACATTAG
TGCAATTGGACTCGGGTAATTGGGTGACGCCACGTTATTGGGCAATTGCAAATTTTAATAGAAAATGGTTTTGGG
CCTTAGCCTTATCATCATTGCCCTAAGCAGTAATTAGGCATCACATCCCAACAGTTTGTTTGTGTAGGAATTTGC
TTTTTGTAAAAGAGCTTTTTGATAAAACTACTATATTATGCTGAATTTCTCTTAAAATTTTTCTAGCCAGATATA
TCTCCAGAAAATTATTCAAATATGTTCAAAAACAGCTTGAAACAGTTTTTGATACCAAATGCTCTTAAAACTTGA
ATTATAGGGTGAATCAAGACGGGCTTTTTCGAGTTTTTCTTAATGTGCTAGGCCAAATATATACTAATATTTGTT
GTTTGGCAGGCCATTCCCACTCATGCAGTTGCTTCTGGAATGAATGCTGTGGAGGGTGTAGTAATGGAGATTGTC
ATCACTTTTGCTCTAGTCTACACAGTCTACGCCACTGCAGCAGACCCCAAAAAGGGCTCATTGGGCATAATTGCA
CCCATTGCAATTGGGTTCATTGTTGGTGCAAACATTTTAGCTGCTGGCCCATTTAGTGGTGGATCGATGAACCCA
GCCCGATCATTTGGGCCGGCCGTTGCTGCTGGAGACTTCTCTCAGAACTGGATCTATTGGGTTGGCCCACTTATT
GGGGGAGGATTGGCAGGCCTGGTATATGGTTATGTTTTTATTGCATCATATGCCCCATTCCCAGCCCAAGAGGGA
TATGCT**TAA**

>*Oleur*TIP2;4

ATGGTGAAGATGGCATTGGGTAGCATTGGTGACTCTTTTTCAGTTCCGTCGCTAAAGGCTTATCTTGCTGAGTTC
ATTGCCACCCTCCTCTTTGTCTTTGCTGGTGTAGGTTCCGCTATAGCTTACAACAAGCTCACGTCCGATGCAGCT
CTCGACCCACCTGGATTGATGGCAGTAGCGGTGGCTCACGCATTTGCATTGTTTGTGGGCGTTTCCATGGCGGCC
AATGTATCCGGCGGTCATCTGAATCCGGCCGTCACATTTGGACTGGCCATTGGAGGCAACATCACAATCATAACT
GGCATTTTCTATTGGATTGCCCAGTTGCTTGGTTCCACTGTTGCATGCCTTCTCCTCAAATTCGTCACTAATGGC
TTGGCCATTCCCACTCATGCAGTTGCTTCTGGAATGAATGCTGTGGAGGGTGTAGTAATGGAGATTGTCATCACT
TTTGCTCTAGTCTACACAGTCTACGCCACTGCAGCAGACCCCAAAAAGGGCTCATTGGGCATAATTGCACCCATT

GCAATTGGGTTCATTGTTGGTGCAAACATTTTAGCTGCTGGCCCATTTAGTGGTGGATCGATGAACCCAGCCCGA
TCATTTGGGCCGGCCGTTGCTGCTGGAGACTTCTCTCAGAACTGGATCTATTGGGTTGGCCCACTTATTGGGGGA
GGATTGGCAGGCCTGGTATATGGTTATGTTTTTATTGCATCATATGCCCCATTCCCAGCCCAAGAGGGATATGCT
TAA
>*Oleur*TIP2;4
MVKMALGSIGDSFSVPSLKAYLAEFIATLLFVFAGVGSAIAYNKLTSDAALDPPGLMAVAVAHAFALFVGVSMAA
NVSGGHLNPAVTFGLAIGGNITIITGIFYWIAQLLGSTVACLLLKFVTNGLAIPTHAVASGMNAVEGVVMEIVIT
FALVYTVYATAADPKKGSLGIIAPIAIGFIVGANILAAGPFSGGSMNPARSFGPAVAAGDFSQNWIYWVGPLIGG
GLAGLVYGYVFIASYAPFPAQEGYA*

*Oleur*TIP2;5a Oleur061Scf1607g00003.1

>Oleur061Scf1607:86016..87968 (+ strand) class=gene length=1953

>*Oleur*TIP2;5a
ATGGTGAAGATTAATTGGGGTAGCATTGGCGACTCTTTTTCGGTTACGTCGCTAAAGGCTTACATTGCTGAGTTT
ATTGCCACCCTTCTCTTTGTTTTTGCTGGTGTTGGTTCTGCTATAGCTTATAGTAAGTCATCTTTTATGTTTTGC
TACAGTATATACACACGTAATTCCGTCTATATTTAGTCTCTATCTGGTGGGGATTTTTAGTAGATTTTGATAAAT
GTTGATAAAATATGTAGTTGAAAAGTGATGAGTGTTATAAAATGTGTTGTTAAAATTAAATGTTGATTTTAGTTT
AGATTAAAATTTGAATTTGAATTTTCATCGTACAGGCGCTCAGATAGTTGACCATAAGCCCGGATAAAGGAGGAT
CGTTGCAATAGATTAACCATAAGAATAAAAAGGTTTAGGCTTAGGTGTACTAATATATGGAGTATGATTGATGTT
TGCAGACAAGCTCACGTCTGATGCAGCTCTCGACCCACCCGGTTTGGTGGCAGTCGCGGTGGCTCACGCATTTGC
ATTGTTCGTAGGCGTTTCCATGGCGGCCAATGTGTCCGGTGGTCATTTGAATCCGGCTGTCACATTCGGATTGGC
CATCGGAGGCAACATCACCATCATAACTGGCCTTTTCTATTGGATTGCACAATTGCTTGGTTCTATTGTTGCATG
CCTTCTCCTCAAATTTGTCACCGGTGGCTTGGTAAGTTATACCAAGATTAAAATTAAACTTATTTTGAGGGTAAA
GCACTAAAATTGCTCATTTTGAGATCTTTAGAGCCTTTCGCGATAAGAAAAATACTCATCTTTTGGATTAACTGC
ATGTTGAAGCTTATTTTTAATTATTTTCTGATTGATATAAGATAGAAAACAGCTTGTCTACTCTGATGTTGGACC
AAAACAAGTCATAATTTATGTAAAATCGACTTCTAAATTGAAACGTCAACTCAATTCATTATAGGTTCGTTAAGC
TTTAATAACTGAGGTCACAAGAAATTACTCAAATTGTTTGAGTAGTTCGTGTTCGACTAGACAAAAACCTTACAA
AACATCACCTGATACATTAAAGACTGGAGTTCAAGTATTTCATTGAATTAGCATTTAAATTCTACTCGAATTTGA
GTAGTTTTAGCCCTTGTTTGACCCCCCATTAAATTAAAATTCATTTTTTCTCTTTTTATCAAACATATATTTTTC
AATAAAACAATATTACACGTAAACTTAAAAACTTTTCATTTCTATTTAACCACACATTCTTCAAAAAATTACAAT
TCTTTTCATTATAATTCGAAATAATGGATCCCAAACAAGGGCCTAGACTATCGCCAGATTGAATCAAGATGGGCT
TTTACCTGAATGTGGTAAGCCCAAACCATACTAATATTTGATGTTTGGCAGGCCATTCCCACTCATGCAGTTGCT
TCTGGTATGAATGCTGTGGAGGGTGTTGTAATGGAGATTGTCATCACTTTTGCTTTGGTCTACACAGTCTATGCC
ACTGCTGCGGACCCCAAAAAAGGCTCGTTGGGCACAATTGCACCCATTGCAATTGGGTTCATTGTTGGTGCAAAC
ATTTTAGCTGCTGGCCCATTTAGTGGTGGATCGATGAACCCGGCCCGATCATTTGGGCCAGCCGTTGCTGCAGTC
GACTTCTCTCAGAACTGGATCTATTGGGTCGGCCCACTTATTGGTGGAGGTTTGGCTGGCCTGGTATATGGGGAT
GTTTTTATTGGATCATATGCCCCACTTCCAGTCCAAGAAGAATATCCT**TAA**
>*Oleur*TIP2;5a
ATGGTGAAGATTAATTGGGGTAGCATTGGCGACTCTTTTTCGGTTACGTCGCTAAAGGCTTACATTGCTGAGTTT
ATTGCCACCCTTCTCTTTGTTTTTGCTGGTGTTGGTTCTGCTATAGCTTATAACAAGCTCACGTCTGATGCAGCT
CTCGACCCACCCGGTTTGGTGGCAGTCGCGGTGGCTCACGCATTTGCATTGTTCGTAGGCGTTTCCATGGCGGCC
AATGTGTCCGGTGGTCATTTGAATCCGGCTGTCACATTCGGATTGGCCATCGGAGGCAACATCACCATCATAACT
GGCCTTTTCTATTGGATTGCACAATTGCTTGGTTCTATTGTTGCATGCCTTCTCCTCAAATTTGTCACCGGTGGC
TTGGCCATTCCCACTCATGCAGTTGCTTCTGGTATGAATGCTGTGGAGGGTGTTGTAATGGAGATTGTCATCACT
TTTGCTTTGGTCTACACAGTCTATGCCACTGCTGCGGACCCCAAAAAAGGCTCGTTGGGCACAATTGCACCCATT
GCAATTGGGTTCATTGTTGGTGCAAACATTTTAGCTGCTGGCCCATTTAGTGGTGGATCGATGAACCCGGCCCGA
TCATTTGGGCCAGCCGTTGCTGCAGTCGACTTCTCTCAGAACTGGATCTATTGGGTCGGCCCACTTATTGGTGGA
GGTTTGGCTGGCCTGGTATATGGGGATGTTTTTATTGGATCATATGCCCCACTTCCAGTCCAAGAAGAATATCCT
TAA
>*Oleur*TIP2;5a
MVKINWGSIGDSFSVTSLKAYIAEFIATLLFVFAGVGSAIAYNKLTSDAALDPPGLVAVAVAHAFALFVGVSMAA
NVSGGHLNPAVTFGLAIGGNITIITGLFYWIAQLLGSIVACLLLKFVTGGLAIPTHAVASGMNAVEGVVMEIVIT
FALVYTVYATAADPKKGSLGTIAPIAIGFIVGANILAAGPFSGGSMNPARSFGPAVAAVDFSQNWIYWVGPLIGG
GLAGLVYGDVFIGSYAPLPVQEEYP*

*Oleur*TIP2;5b Oleur061Scf8992g00006.1

>Oleur061Scf8992:66181..68174 (+ strand) class=gene length=1994

>*Oleur*TIP2;5b
ATGGTGAAGATTAATTGGGGTAGCATTGGCGACTCTTTTTCGGTTACGTCGCTAAAGGCTTACATTGCTGAGTTT
ATTGCCACCCTTCTCTTTGTTTTTGCTGGTGTTGGTTCTGCTATAGCTTATAGTAAGTCATCTTTTATGTTTTGC
TACAGTATATACACACGTAATTCTGTCTATATTTAGTCTCTATCTGGTGGGAATTTTTAGTAGATTTTGATAAAT
GTTGATGAAATATGTGGTTGAAAAGTGATAAGTGTTATAAAATATGTGGTTAAAATTAAATGTTGATCTTAATTT
AGATTAAAATTTGAATTTGAATTTTCATCATACAGGCGCTCAGATAGTTGACCCTAAGCCCGGATAAAGGAGGAT
GGTTGCAGTAGATTAACCATAAGAATAAAAAGGTTTAGGCTTAGGTGTACTAATATATGGAGTATGATTGATGTT
TGCAGACAAGCTCACGTCTGATGCAGCTCTCGACCCACCTGGTTTGGTGGCAGTCGCGGTGGCTCACGCATTTGC
ATTGTTCGTGGGCGTTTCCATGGCGGCCAATGTGTCCGGTGGTCATTTGAATCCGGCTGTCACATTCGGATTGGC
CATCGGAGGCAACATCACCATCATAACTGGCCTTTTCTATTGGATTGCACAATTGCTTGGTTCTATTGTTGCATG
CCTTCTCCTCAAATTCGTCACCGGTGGCTTGGTAAGTTATACCAAGATTAAATTAAACTTATTTTGAGGGTAAAG
CACTAAAATAGCTCATTTTGAGATCTTTAGAGCCTTTCGCAATAAGAAAAATACTCATCTTTTGGATTAACTGCA
TGTTGAAGCTTATTTTTAATTATTTTCTGATTGATATAAGATAGAAAACAGCTTGTCTACTCTGATGTTGGACCA
AAACAAGTCATAATTTATGTAAAATCGACTTCTAAATTGAAACGTCAACTCAATTCATTATAGGTTCGTTAAGCT
TTAATAACTGAGGTAACAAAAAATTACTCAAATTGTTCGAGTAGTTCGTGTTCGACTAGACAAAAACCTTACAAA
ACATCACCTGATACATTAAAGACTGGAGTTCAAGTATTTCATTGAATTAGCATTTAAATTCTACTCGAATTTGAG
TAGTTTTACCCTTTGTTTGAAACCCCATTAAATTAAAATTCATTCTCTCTCTCTTTTTATCAAGCATACATTTTT
CAATAAAACAATATTACACGTAAACTTAAATACTTTTCCTTTCTATTTAACCACACATTCTTCAAAAAATTACTA
TTCTTTTCATTATAATTCGAAATAATGGATCCCAAACAAGGGCCTAGACTATCGCTAGATCGTATCAAGATGGGC
TTTTACCTGAATGTGGTAAGCCCAAACCATACTAATATTTGATGTTTGGCAGGCCATTCCCACTCATGCAGTTGC
TTCTGGTATGAATGCTGTGGAGGGTGTTGTAATGGAGATTGTCATCACTTTTGCTTTGGTCTACACAGTCTATGC
CACTGCTGCGGACCCCAAAAAAGGCTCGTTGGGCACAATTGCACCCATTGCAATTGGGTTCATTGTGGGTGCAAA
CATTTTAGCTGCTGGCCCATTTAGTGGTGGATCGATGAACCCGGCCCGATCATTTGGGCCAGCCGTTGCCGCAGT
CGACTTCTCTCAGAACTGGATCTATTGGGTCGGCCCACTTATTGGTGGAGGATTGGCTGGCCTGGTATATGGTGA
TGTTTTTATTGGATCATATGCCCCACTTCCAGTCCAAGAAGAATATCCT**TAA**

>*Oleur*TIP2;5b
ATGGTGAAGATTAATTGGGGTAGCATTGGCGACTCTTTTTCGGTTACGTCGCTAAAGGCTTACATTGCTGAGTTT
ATTGCCACCCTTCTCTTTGTTTTTGCTGGTGTTGACAAGCTCACGTCTGATGCAGCTCTCGACCCACCTGGTTTG
GTGGCAGTCGCGGTGGCTCACGCATTTGCATTGTTCGTGGGCGTTTCCATGGCGGCCAATGTGTCCGGTGGTCAT
TTGAATCCGGCTGTCACATTCGGATTGGCCATCGGAGGCAACATCACCATCATAACTGGCCTTTTCTATTGGATT
GCACAATTGCTTGGTTCTATTGTTGCATGCCTTCTCCTCAAATTCGTCACCGGTGGCTTGGCCATTCCCACTCAT
GCAGTTGCTTCTGGTATGAATGCTGTGGAGGGTGTTGTAATGGAGATTGTCATCACTTTTGCTTTGGTCTACACA
GTCTATGCCACTGCTGCGGACCCCAAAAAAGGCTCGTTGGGCACAATTGCACCCATTGCAATTGGGTTCATTGTG
GGTGCAAACATTTTAGCTGCTGGCCCATTTAGTGGTGGATCGATGAACCCGGCCCGATCATTTGGGCCAGCCGTT
GCCGCAGTCGACTTCTCTCAGAACTGGATCTATTGGGTCGGCCCACTTATTGGTGGAGGATTGGCTGGCCTGGTA
TATGGTGATGTTTTTATTGGATCATATGCCCCACTTCCAGTCCAAGAAGAATATCCT**TAA**

>*Oleur*TIP2;5b
MVKINWGSIGDSFSVTSLKAYIAEFIATLLFVFAGVDKLTSDAALDPPGLVAVAVAHAFALFVGVSMAANVSGGH
LNPAVTFGLAIGGNITIITGLFYWIAQLLGSIVACLLLKFVTGGLAIPTHAVASGMNAVEGVVMEIVITFALVYT
VYATAADPKKGSLGTIAPIAIGFIVGANILAAGPFSGGSMNPARSFGPAVAAVDFSQNWIYWVGPLIGGGLAGLV
YGDVFIGSYAPLPVQEEYP*

*Oleur*TIP3;1a Oleur061Scf6254g04008.1

>Oleur061Scf6254:448318..449463 (+ strand) class=gene length=1146

>*Oleur*TIP3;1a
ATGCCTCGTGGGTATACTTTTGGTAGGGCTGAAGAAGCCACCCACCCCGACTCTGCCAGGGCCACCTTGGCTGAG
TTTCTCTCCATGCTCGTCTTTGTCTTCGCCGGCGAAGGCTCCGTCCTTGCTCTTGGTATCTTAATTCTTCAGTAA
CGTTCTTGAAAATAAGAAGAAGTAGGTGCACATGGACGTACAGCTCATATTATTCTCAATTTGTATCGATTTGCA
GATAAGATGTATAAGGATACAGCCTTAGGAGCATCAAGCCTGGTGGTCATCGCTCTCGCCCACGCCCTGTCACTG
TTTGCAGCTGTGGCTTCGAGTATGAATGTTTCCGGCGGGCACGTCAATCCTGCCGTCACTTTCGGCGCACTTGTA
GGGGGAAGAGTCTCTGTGCTGCGTGCTATTTACTACTGGATTGCTCAACTTTTGGGTGCAGTTTTAGCTGCTATC
TTGTTGAGGCTGGCAACTGATGGCTTGGTAAAAAGAAATAGAAAAACCCACCCGCCTTCCACCTTAATTAATTAC
TTGCATGTCTCAGCACATGTTTTGGTGTGTTTATAAGTGCTTATAAGTACCGGGACTTTCTACTCGTTTTTAATA
AGCATCCTGTGTTTGCAGAGGCCGCGAGGGTTTTCCGTGGCGGATGGAGTAGGAGAATGGAACGCTTTGGTGATG
GAGATTGTGCTGACATTTGGACTGGTATATACAGTGTACGCGACTGCAATCGATCCAAAAAGAGGAAGCTTGGGC
ACCATTGCTCCTCTTGCCATTGCCTTCATAGTTGGGGCTAATGTGCTGGTTGGAGGGCCTTTCTCCGGAGCATCG
ATGAACCCTGCTCGTGCGTTCGGGCCAGCACTTGTGGGATGGAGGTGGAAAGACCACTGGGTCTACTGGTTGGGA
CCATTCGTTGGTGCGGCTCTGGCTGGAGTTATATACGAGTTCGGGGTGATACAAACCGAGACCCCGCACCCCACG
CATCACCTTCACCAGCCTTTGGCCCCAGAAGATTAC**TAG**

>*Oleur*TIP3;1a

ATGCCTCGTGGGTATACTTTTGGTAGGGCTGAAGAAGCCACCCACCCCGACTCTGCCAGGGCCACCTTGGCTGAG
TTTCTCTCCATGCTCGTCTTTGTCTTCGCCGGCGAAGGCTCCGTCCTTGCTCTTGATAAGATGTATAAGGATACA
GCCTTAGGAGCATCAAGCCTGGTGGTCATCGCTCTCGCCCACGCCCTGTCACTGTTTGCAGCTGTGGCTTCGAGT
ATGAATGTTTCCGGCGGGCACGTCAATCCTGCCGTCACTTTCGGCGCACTTGTAGGGGGAAGAGTCTCTGTGCTG
CGTGCTATTTACTACTGGATTGCTCAACTTTTGGGTGCAGTTTTAGCTGCTATCTTGTTGAGGCTGGCAACTGAT
GGCTTGAGGCCGCGAGGGTTTTCCGTGGCGGATGGAGTAGGAGAATGGAACGCTTTGGTGATGGAGATTGTGCTG
ACATTTGGACTGGTATATACAGTGTACGCGACTGCAATCGATCCAAAAAGAGGAAGCTTGGGCACCATTGCTCCT
CTTGCCATTGCCTTCATAGTTGGGGCTAATGTGCTGGTTGGAGGGCCTTTCTCCGGAGCATCGATGAACCCTGCT
CGTGCGTTCGGGCCAGCACTTGTGGGATGGAGGTGGAAAGACCACTGGGTCTACTGGTTGGGACCATTCGTTGGT
GCGGCTCTGGCTGGAGTTATATACGAGTTCGGGGTGATACAAACCGAGACCCCGCACCCCACGCATCACCTTCAC
CAGCCTTTGGCCCCAGAAGATTAC**TAG**
>*Oleur*TIP3;1a
MPRGYTFGRAEEATHPDSARATLAEFLSMLVFVFAGEGSVLALDKMYKDTALGASSLVVIALAHALSLFAAVASS
MNVSGGHVNPAVTFGALVGGRVSVLRAIYYWIAQLLGAVLAAILLRLATDGLRPRGFSVADGVGEWNALVMEIVL
TFGLVYTVYATAIDPKRGSLGTIAPLAIAFIVGANVLVGGPFSGASMNPARAFGPALVGWRWKDHWVYWLGPFVG
AALAGVIYEFGVIQTETPHPTHHLHQPLAPEDY*

*Oleur*TIP3;1b Oleur061Scf5220g00047.1

>Oleur061Scf5220:6075..7220 (- strand) class=gene length=1146

>*Oleur*TIP3;1b
ATGCCTCGTGGGTATACTTTTGGTAGGGCTGAAGAAGCCAACCACCCCGACTCTGCCAGGGCCACCTTGGCTGAG
TTTCTCTCCATGCTCGTCTTTGTCTTCGCCGGCGAAGGCTCCGTCCTTGCTCTTGGTATCTTAATTCTTCAGTAA
CGTTCTTGAAAATAAGAAGAAGTAGGTGCACATGGACCTACAGCTCATATTATTCTCAATTTGTATCGATTTGCA
GATAAGATGTATAAGGATACAGCCTTAGGAGCATCAAGCCTGGTGGTCATCGCTCTCGCCCACGCCCTGTCACTG
TTTGCAGCTGTGGCTTCGAGTATGAATGTTTCCGGCGGGCACGTCAATCCTGCCGTCACTTTCGGCGCACTTGTG
GGTGGGAGAGTCTCCGTGCTGCGTGCTATTTACTACTGGATTGCTCAACTTTTGGGTGCAGTTTTAGCTGCTATC
TTGTTGAGGCTGGCAACTGATGGCTTGGTAAAAAGAAATAGAAAAACCCACCCCCCTTCCACCTTAATTAATTAC
TTGCATGTCTTAGCACAAGTTTTGGTGTGTTTATAAGTGCTTATAAGTACCGGGACTTTCTACTCCTTTTTAATA
AGCAGCTTGTGTTTGCAGAGGCCGCGAGGGTTTTCCGTGGCCGATGGAGTAGGAGAATGGAACGCTTTGGTGATG
GAGATAGTGCTGACATTTGGACTGGTATATACAGTGTACGCGACTGCAATCGATCCAAAGAGAGGAAGCTTGGGC
ACCATTGCTCCTCTTGCCATTGCCTTCATAGTTGGGGCTAATGTACTGGTTGGAGGGCCTTTCTCCGGAGCATCG
ATGAACCCTGCTCGTGCATTCGGGCCAGCACTGGTAGGATGGAGGTGGAGAGACCACTGGGTCTACTGGGTGGGA
CCATTCGTTGGTGCGGCTCTAGCTGGAGTTATATATGAGTTCGGAGTGATACAAACCGAGACCCCGCACCCCACG
CATCACCTTCACCAGCCTTTGGCCCCAGAAGATTAC**TAG**
>*Oleur*TIP3;1b
ATGCCTCGTGGGTATACTTTTGGTAGGGCTGAAGAAGCCAACCACCCCGACTCTGCCAGGGCCACCTTGGCTGAG
TTTCTCTCCATGCTCGTCTTTGTCTTCGCCGGCGAAGGCTCCGTCCTTGCTCTTGATAAGATGTATAAGGATACA
GCCTTAGGAGCATCAAGCCTGGTGGTCATCGCTCTCGCCCACGCCCTGTCACTGTTTGCAGCTGTGGCTTCGAGT
ATGAATGTTTCCGGCGGGCACGTCAATCCTGCCGTCACTTTCGGCGCACTTGTGGGTGGGAGAGTCTCCGTGCTG
CGTGCTATTTACTACTGGATTGCTCAACTTTTGGGTGCAGTTTTAGCTGCTATCTTGTTGAGGCTGGCAACTGAT
GGCTTGAGGCCGCGAGGGTTTTCCGTGGCCGATGGAGTAGGAGAATGGAACGCTTTGGTGATGGAGATAGTGCTG
ACATTTGGACTGGTATATACAGTGTACGCGACTGCAATCGATCCAAAGAGAGGAAGCTTGGGCACCATTGCTCCT
CTTGCCATTGCCTTCATAGTTGGGGCTAATGTACTGGTTGGAGGGCCTTTCTCCGGAGCATCGATGAACCCTGCT
CGTGCATTCGGGCCAGCACTGGTAGGATGGAGGTGGAGAGACCACTGGGTCTACTGGGTGGGACCATTCGTTGGT
GCGGCTCTAGCTGGAGTTATATATGAGTTCGGAGTGATACAAACCGAGACCCCGCACCCCACGCATCACCTTCAC
CAGCCTTTGGCCCCAGAAGATTAC**TAG**
>*Oleur*TIP3;1b
MPRGYTFGRAEEANHPDSARATLAEFLSMLVFVFAGEGSVLALDKMYKDTALGASSLVVIALAHALSLFAAVASS
MNVSGGHVNPAVTFGALVGGRVSVLRAIYYWIAQLLGAVLAAILLRLATDGLRPRGFSVADGVGEWNALVMEIVL
TFGLVYTVYATAIDPKRGSLGTIAPLAIAFIVGANVLVGGPFSGASMNPARAFGPALVGWRWRDHWVYWVGPFVG
AALAGVIYEFGVIQTETPHPTHHLHQPLAPEDY*

*Oleur*TIP3;1c Oleur061Scf3963g07030.1

>Oleur061Scf3963:728110..729328 (+ strand) class=gene length=1219

>*Oleur*TIP3;1c
ATGCCTCGTGGGTACACTTTTGGTAGGGCAGAAGAAGCCACTCACCCCGACTCTGCCAGGGCGACCTTGGCTGAG
TTCCTGTCGACCCTCGTCTTCGTATTTGCCGGAGAGGGCTCCATCCTTGCCCTTGGTACGTAATTTTGTACTTCA

GAAACGTCCTAACTAATATGCATAATTTTATGTTCTCAACTTGTATTAATGTTGCAGATAAGATGTATAGGGAGA
CAGCTTTAGGAGCATCGAGCCTGCTGGTTATCGCCCTCGCCCACGCCCTCGCTCTGTTCGCAGCTGTGGCTTCAA
GTATGAACGTCTCTGGCGGGCACGTTAATCCTGCTGTCACTTTTGGCGCACTTGTTGGTGGGAGAGTCTCCGTGC
TACGTGCCATTTTCTATTGGATTGCTCAGATTTTGGGTGCTGTTGCAGCTTCTCTCTTGTTGTGGCTAGCGACTA
ATGGTTTGGTAAACAAATTAAGAAAAAGAAAACCCACTTGTGTTTATAATTAACCGGATTGATTTCAATTTTTTG
ACGTTTGTGGCTTCTATATATGTTATATACGTTTAAGAAAGTAACTTTTAAGCATGTGGTGATTGTGGTCGCAGA
AGCCGCAAGGATATTCCGTGGCGCTTGGAGTAGGAGAGTGGAACGCTTTGGTGATGGAGATAGTGTTGACTTTTG
GACTGGTATATACGGTGTACGCGACTGCGATTGATCCAAAGAGGGGAAGTTTGGGAACTATTGCACCTCTTGCGA
TAGCCTTCATATTTGGGGCTAATGTGCTGGTTGGAGGGCCTTTCTCAGGAGCATCGATGAACCCTGCTCGGGCTT
TTGGGCCTGCACTGGTAGGGGGGAGGTGGAGGGACCACTGGATCTACTGGTTGGGACCGTTGCTTGGTGCGGCCC
TGGCCGGAGTTATATACGAGTTTGGAATGATACAAGCGGAGACCCCTCACCACACACATCACCAGCCTTTGGCTC
CAGAAGATTAC**TAG**

>*Oleur*TIP3;1c

ATGCCTCGTGGGTACACTTTTGGTAGGGCAGAAGAAGCCACTCACCCCGACTCTGCCAGGGCGACCTTGGCTGAG
TTCCTGTCGACCCTCGTCTTCGTATTTGCCGGAGAGGGCTCCATCCTTGCCCTTGATAAGATGTATAGGGAGACA
GCTTTAGGAGCATCGAGCCTGCTGGTTATCGCCCTCGCCCACGCCCTCGCTCTGTTCGCAGCTGTGGCTTCAAGT
ATGAACGTCTCTGGCGGGCACGTTAATCCTGCTGTCACTTTTGGCGCACTTGTTGGTGGGAGAGTCTCCGTGCTA
CGTGCCATTTTCTATTGGATTGCTCAGATTTTGGGTGCTGTTGCAGCTTCTCTCTTGTTGTGGCTAGCGACTAAT
GGTTTGAAGCCGCAAGGATATTCCGTGGCGCTTGGAGTAGGAGAGTGGAACGCTTTGGTGATGGAGATAGTGTTG
ACTTTTGGACTGGTATATACGGTGTACGCGACTGCGATTGATCCAAAGAGGGGAAGTTTGGGAACTATTGCACCT
CTTGCGATAGCCTTCATATTTGGGGCTAATGTGCTGGTTGGAGGGCCTTTCTCAGGAGCATCGATGAACCCTGCT
CGGGCTTTTGGGCCTGCACTGGTAGGGGGGAGGTGGAGGGACCACTGGATCTACTGGTTGGGACCGTTGCTTGGT
GCGGCCCTGGCCGGAGTTATATACGAGTTTGGAATGATACAAGCGGAGACCCCTCACCACACACATCACCAGCCT
TTGGCTCCAGAAGATTAC**TAG**

>*Oleur*TIP3;1c

MPRGYTFGRAEEATHPDSARATLAEFLSTLVFVFAGEGSILALDKMYRETALGASSLLVIALAHALALFAAVASS
MNVSGGHVNPAVTFGALVGGRVSVLRAIFYWIAQILGAVAASLLLWLATNGLKPQGYSVALGVGEWNALVMEIVL
TFGLVYTVYATAIDPKRGSLGTIAPLAIAFIFGANVLVGGPFSGASMNPARAFGPALVGGRWRDHWIYWLGPLLG
AALAGVIYEFGMIQAETPHHTHHQPLAPEDY*

***Oleur*TIP4;1 Oleur061Scf6420g00014.1**

>Oleur061Scf6420:44973..52364 (- strand) class=gene length=7392

>*Oleur*TIP4;1

ATGGCTAAGATTGCCTTGGGAAATGCCCGAGAGGCCTTTCAATCGGACTGCATCCAGGCCCTTATTGTGGAATTC
ATTTGCACGTTTCTTTTCGTGTTCGCCGGGGTCGGCGCTGCCATGGCTACCGGTAATGTCTTGAGTTTCCTCATT
TAAATTCTATTTTCAACTACATGTCTTCGATATGGTAACCTTTTAACAGATAAGCTGAATGGGGATCCGTTGGTG
GGACTATTTTTTGTGGCTATGGCACATGCGTTGGTGGTGGCGGTGATGATTTCAGCCGGATTCCGCGTTTCCGGT
GGCCACATAAACCCTGCTGTCACCCTCGGCCTATTAGCAGGCGGCCATATCACTGTCGTCAGATCAATCCTCTAC
TGGATTGATCAGTTATTAGCTTCTACAGCAGCTTGTGCTCTGCTCAAGTACCTAACTGGAGGATTGGTTAGTATT
AGTATATCTTTGCCTTATACGAATGCACTCAGTTTGTCTTTAAAAACGCATAATCCTAAAAAAAATTGAAATCT
CGTTTCATTCACTTCGGCTTCCCATCTTAATGATCTCGGTTAGTCAAGTAAAGTGACGTAGAGAGCGACTTCTAA
ATGGTTGGCCCAAGTGGTGGAATGTGACCCGGTTTGGCCCCTTGTGCTCTATCTCGCATTTACTTAATACACAAC
TTATTGAGCGACTGAGATGATGACACATGTTGCTAGTTGAACTGTTGGATGCATTATGACCTTTCATATTCTAGT
CTCTCTCCTTTTTTTGGGTTCAAGATTATTGTAGGTTTCCCGACTCTATTTGTCTTTATGATTTCTCTCTCTAAA
AACCCTTGATTTTCATTTGTAATCTTGAATCTTATTGTAATCTCGGTGGTGTCCAATATAAATCATGTTATGGTT
AGAATTTTATTTTTCTTGGGTGAACTGCAACTCTTGGTTACGTGGTGTAATCTGTAAACGAGAGAAAAAGTGAGA
GTTGAGTCTGAGGGCTTTAAGTCGAGTGATTTTTGAGTATCTGATTCTTGTATTTTGACACAATTTGTTGGATTC
TAGAGGATTAGAACGTTAACCACTTAGGTGGATGCTCAACGTGGTGAGTAGCCGCCCATTCTTGGGTGAACATCT
GGGCCTACGAGCGAAAAATCTTTATGTTCTCTGTGTTTGATTTACTTGAATAAATTTTGGGATTGTTAGATACCA
ACAGTGGTATCAGAGCTGCTCAATCTGTGTTGGAAGCCTTCGGTCTGAATATTTCTGGTGCTCTTGGTGACCAAA
ACTCTAGATCTTCACAGATTTGTATTTTTAAGGGCTTGATTTTCATTAATTTCTAAGAAAGTAAAATAGTTTTGA
TATTCACTATTTAGTGTCCTAAGACGTGTTGGTAACGAGTTTCTAAACCCTATTTCCTTCTTTAAAACTTGCATA
TCTTGGTTGAAACTCACCCTGCTCTTTCTTTGTCTAGGGTTTCATGCGACCTAGAATCTGAGGGAACGATGTTTA
ACGGTTTCTTTCAAGATTATTGTTTATCCGTGATTCTGATATTTGTACTTTATGATTGGGTTATTTGTTGTGATC
TGCCTTCTCTGATTTGCATATTGGTGTATTTGCGTGGACACTATTGCACATTATCACTTTGTGCGTATATCTCTG
TGAATAAATTGTGTCTTAGTTTTGTGTACTTTAGACTTTATACCGAAATTTTTTAAAAATCATGGGAAGTACCAA
AATTGATATTGAGATATGGTATGGCAATGAAGACTTTAGTCTCTGGAGTAAAAAATGAAGGCCCTTTTAGTACAA
CAAAGATGTGTGAAAGCTATAGATGAATCATGGGGTACAAAAGTTACTGAAAATAGGAGGACTGATCAAGATAAA
ATTGCATGGAGTACAATTTTCTTGCATTTATTTGAAAGTGTGATTAGAGAGGTAGAAAAAACTAAGACTGCAATA
GAAATCTAGACAAAGCATGATTTTCTTTATCAAACTAAGACCCTTCCCAACAAATGTTACTTGTGGAAACAATTT

TTCTGTTTCAAATTTGACCCTAATTGTGATCTTGAAGTTAATCCTGATAGATTTAATAAACTCTTTCAAGATATC
ACTAATGGTGGTAAGACTTTATCTGATGATAAAAAATCTGTGGCCCTACTGTCATTTTTACCTGAGAAATTTAAA
GAAGTGAAACATCCCCTTGAATATGAAAGAACTACTGTCACAGTTAATGATATTATGACTTCTTTAAGGAATAAG
GAACATGAAGTAAAGAGTGATTCAAAAACTCCATAACTGGAGTAGGTTACAATATAAAAGGTACTAATTCCTTCA
AGAAGAAGAATAATCAATATATGAATAAAAAATTTGGGAAACCAAAAGAATATTATAGTACATCTCAAAAGGGTA
AGTCAAAATTTAATTAGAACAACAGTAAGGCTAAAGATAGGAAGTGTTACTTTTGTGGAAAAATGGCCACTACA
TCCAAGATTGTATGAAAAGAATTGAGGAACTGCAAAATGCAAATAAGGTTGAAGGTGAAGCTGCTATAGCTTATG
AAGAAACAACAACCTCAAATGGACAAGTTTATCTGACTTCTCAACCCATAGTCAAAGATGACTGGATACTAGACT
CTGGATATACTTTCCATATGCCACCCTAATAGAGCTCTTCACTGAATTTTATGAAACAAGTGGAGGACAAGTAAC
TATGAGAAATGATTTTTCGTGCAAGATTAAAGGAATTGGAAAGATTGTTTTAAAACTAGACAATGGGAATGTACT
AACCTCAAACAAAACTAGATACACTCCTAACTTAAAGAGAAACTTAATCTCTTTGGGCACACTTGATGATGAAGG
TTTTAATACAATCATAAATAAAAGTGTTTTGACACTCCAAAATAATCTTGCTAATATCATTTAAGTAGAAAAATC
AAATTGGATATATGTCATGAAAGCTATATCACATAAAAATCAATTTATTGCTGCTACTGTCTCCCTGCTTGACAA
ATCTAAAATATGGCATAAAAGGTTAAGATCCATAAGTGATAAAAATCTGCATGTCCTTAAAAAACAAGGTCTCTT
GGAAAATGATAAAATTGAAAGAGTGTCATATTGTAAACATTGCATCTATGGAAAGCAGAAAAAATTACCTTTTAC
TACTGGTACCCATAAATCTAAAGTTGTTCTTGATTACTTACATGCTGACCTGTGGGGGCCAACTTCTGTAAGTAC
TTATTCTGGTTTTAAATACTATTTCCTGATTGTTGATGATTTTCAAGGAAAAATTGGGTTATGTTCTTGAAAAAT
AAATCTGATACCTTGCAAAACTTTGTTTCTTGGAAAGCTCTTGTTGAAAATCAAGTTAACAAGAAGGTTAAAGCT
CTTAAAACTGATAATGGCCTTGAATTCCGCAATGAAGAATTTGATAAGTACTTCAGAGATAATGAGATCCTTAGG
CATGAGACTGTCACTTACACACCACAATAGAATGGACTCGCAGAAAGAATGAATAGGACCCTTCTAAAAAGAATC
AGGTGCATGTTGATAAGTTCAGGTCTTTCAAAGTTATTCTAGGGGAGGCAGCTAAAAACTGCCTCATGTATGATA
AATAAATCTCCCACTAAATCCCTGAATTATGATACCCCTGATCATACTTTGAAATGGACATTTTATTGGCTATTC
CAAAATTAAAATTTTTGGTTGTATTACTTATGTACGCTAAAGTGAAGGGAAGCTTCAGCCAAGATCTATAAAAGG
TGAATTATTAGGATATGGGGAAGGCATAAAAGGATATAGAATCTGGTTGAGAGGTCAGGGAGGATATGGAGTTGT
AATAAGCAGAAATGTAGTCTTTAACGAATCAGATATGCCCTGCATTAAAGAAAAATCAACTGTTGACACAGATTA
TGATCAAAACAGGTACTTTATGCAAACTGAACTTTTTCCGACTAACATGACCCTTGAAATGAGGATCTGAGGTGG
AGCCTGGATTAGAGCAAGGCATAGAACTTGAACTAGACCAGTCTACAGGTTCCTTAGAAGACTATCATTTGGTTA
GGGACAGAATAAGAAGAGAAATAAGGCCTCCCCTGAGATATAATGAACCTGACTTTATTACTGCACTAAATGTTC
AAAACTTATTGGAAAATGAACTTGTAACCTATGAAGAAGCAGTTAAGAGTAAAGACTCAAACTCTTGGCAGACAA
CCATGGAAGAAAGATGCAGTCCTTAATGAAAAATAAAACTTGGGTCCTAGTAAATAAACCAAAAGGGGGAAATTA
TTGAATTCAAACGGATTTATAAGATAAAGGAAGGTGATCCTTTAACTGAAAATGTTAGGTTTAAGGCCAGATTAG
TTGCTAAAGGTTTTAACCAAAGAGATGGAATTAACTACAATGAAATTTTTCCCCAGTAGTCAAGTAAACCTCTAT
AAGAATTTTACTTTCACTTGTTTCGTAATTTGATTTTTAATTGGAATAATTGGACGTGAAAACTGCCTTTTTACA
TAGAAATTTACATGACTCAACCAAAAAGTTTTGAACAAAAGGGGAAAGAAAACTGTGTGTGTTTATTGCAAGAAT
CTCTTTATAGTTTAAAACAACCACCAAGACAATAGTACATCAGATTTGATACTTTTATCAACAGTCTTGATTTTA
ATAGAAGTGAATATGATCCATATTTTTATTATAAAGGAAGTAGAGGTCCAAATTCTTGTTATTTACTGCTTTATG
TGGATGATATGTTTCTTGCTGAATGTGATGTAACTAAAATAAATAACATTAAGCAAAAGTTAAAGCAAGAATTTG
AGATGAAGGACTTAGGATCAACCAAAAGGATTTTGGGTATGGAGATTAAAAGGGATAGATCTAATTATACCTTGT
TTCTCACACAGTAGTCATATATTTTAAAAATATTGAATATATTTCTATGAAGAAGCTAAGGATGTCACCTTACC
TCTAGGTTGTCACTTTAAATTCACTGCCGAACAATGTCTTAAAACTGAAATGGAAAAGAGTAGAATGGATAATGT
TCCTTGTGCAAATGTTATTGGTTCTGTTATGTATGTTTTAGTTTGTATAAGAACTGATCTTGCTTTTGCTGTAAG
TGTCTTGAGTAGATACATGAGTAACCCGGGTGAGTATCATTGAGAAACAATGAAATGACTACTTAGATATTTAAA
AAGGACTACAAATGTAGGGCTGATGTTCAAAAATAGTAAAATTGGAATACAACTTTAAGGTTTTGTTGATGCAGA
TTTTGTTGGAAATTGTGACAATAGAAAATCTACTCCTGCATACGTGTTTACATTAAATAATACCTTTATAAATTG
GAAATATCAGCTGCAAAAAATAGTTCCTCTATCTCCAACAGAGGCTGAGTATATTGTTGCTACAGATGCAGTTAA
GGAAGGTATTTGGTTGAAAAATTTATTAGATAATATTGGCTTTACTAGTAATAATGCTACAGTGTATTCTGATGG
TCAATCTGCCATACATCTAACTAGAAACCTTGTTTTTTATAATAGGACTAAACATATAGATGTTAAATTCCATTT
TATAAGATATATTATTGAATAAGAGAATAAAGATTGAAAAGGTTTCAACCAAGTATAACCCTATGGATGGGAACT
AAAGTTCTTCCTTTATACAAATTTAGGGCTTGTTTAAATATCCTAAATGTTGTTACTAAGGATATTTTTGTAGGT
ACATAGAGCATATGAAACCATAAGGAGGAGTTAACCCCTAATTCAGATGAAACCTAAAGATGTTAAGCAAGGTGG
AGATTGTTAAAATGTGCTTAACATCTTATTATTTGAGGTCCAGTGATGTAGCCCAATAAATTGTATTTCCTATGG
TTGGCCCATGTGGTAGAATGTGACTCGGTTTGGCCCCTAGTGCTCTATCTCGCATTTACTTAATACACGACTTAT
TGAATGGCTGAGATGATGACACCTGTTATTGGCTGAACCGTTGGATGCATTATGACCTTTTATATTCTAGTCTCT
CTCATTTTTCTGGGTTTGAGGGTTTCAGTCTCTACTTGTCTTTATGATTTCTCTCTCTGAAAACCTTTGGTTTTC
ATTTGTAATCTTGAGTCTTCTAGTATTCTTGGTGATGTCCAGTGTAAATCATGTTATGGTTAGAGTTTTATTGTT
CTTGGGTGAATTGTAATTCTTGGTTACTTGGGGGTAATCTGTAAACGAGAGAAAAAGTGAGAGTTGAGTTTGAGG
GCTTTAAGTCGAGTGATTCTTGGGTCTTTGATTCCTGTATTTTGACGCACTTTGCTGGGTTCTAGTGTATTGGAG
TGTTAATCACTTAGGTGGATACTCAACGTGGTGATTAGTCCATTTTTGAGTGAACATCCGGGCTTACAGACGAAA
CATCTTTGTGTTCTCTGTGTTTGATTTACTCAATTTAATTTTGGGATTGTTAGATACCAACGTATTATATTCTAA
CAAGGAACCTTGTGATTTCTTGATTCTTGCAGACTATACCAGTACATACACTTGCAACTGGGGTGGACTTCGCCC
AAGGGGTTATAATGGAAATTGTGTTGACATTTTCATTGTTATTCACTGTTTATGCAACAATTGTGGACCCCAAGA
AGGGGTTCCTTGAAGGGATGGGCCCACTTCTTACTGGTCTTGTTGTTGGGGCCAACGTCATGGCTGGTGGCCAT
TCTCAGGGGCTTCAATGAACCCTGCAAGGTCATTCGGGCCAGCTTTGGTGAGCGGGGATTGGACCGATCACTGGG

TATACTGGGTTGGGCCACTCATTGGAGGTGGTCTTGCTGGATATATCTATGAGAACTTCTTCATTGTCAGATCTC
ATGTTCAACTGTCAGGAGAGGAAGTTTTC**TAA**

>OleurTIP4;1

ATGGCTAAGATTGCCTTGGGAAATGCCCGAGAGGCCTTTCAATCGGACTGCATCCAGGCCCTTATTGTGGAATTC
ATTTGCACGTTTCTTTTCGTGTTCGCCGGGGTCGGCGCTGCCATGGCTACCGATAAGCTGAATGGGGATCCGTTG
GTGGGACTATTTTTTGTGGCTATGGCACATGCGTTGGTGGTGGCGGTGATGATTTCAGCCGGATTCCGCGTTTCC
GGTGGCCACATAAACCCTGCTGTCACCCTCGGCCTATTAGCAGGCGGCCATATCACTGTCGTCAGATCAATCCTC
TACTGGATTGATCAGTTATTAGCTTCTACAGCAGCTTGTGCTCTGCTCAAGTACCTAACTGGAGGATTGACTATA
CCAGTACATACACTTGCAACTGGGGTGGACTTCGCCCAAGGGGTTATAATGGAAATTGTGTTGACATTTTCATTG
TTATTCACTGTTTATGCAACAATTGTGGACCCCAAGAAGGGGTTCCTTGAAGGGATGGGCCCACTTCTTACTGGT
CTTGTTGTTGGGGCCAACGTCATGGCTGGTGGGCCATTCTCAGGGGCTTCAATGAACCCTGCAAGGTCATTCGGG
CCAGCTTTGGTGAGCGGGGATTGGACCGATCACTGGGTATACTGGGTTGGGCCACTCATTGGAGGTGGTCTTGCT
GGATATATCTATGAGAACTTCTTCATTGTCAGATCTCATGTTCAACTGTCAGGAGAGGAAGTTTTC**TAA**

>*Oleur*TIP4;1

MAKIALGNAREAFQSDCIQALIVEFICTFLFVFAGVGAAMATDKLNGDPLVGLFFVAMAHALVVAVMISAGFRVS
GGHINPAVTLGLLAGGHITVVRSILYWIDQLLASTAACALLKYLTGGLTIPVHTLATGVDFAQGVIMEIVLTFSL
LFTVYATIVDPKKGFLEGMGPLLTGLVVGANVMAGGPFSGASMNPARSFGPALVSGDWTDHWVYWVGPLIGGGLA
GYIYENFFIVRSHVQLSGEEVF*

*Oleur*TIP5;1 Oleur061Scf8528g00015.1

>Oleur061Scf8528:7335..8665 (+ strand) class=gene length=1331

>*Oleur*TIP5;1

ATGGCGTCCTTAAAATCCCGTTTGGAGCACACTGTTACACCCACTGCCCTCAGATCGTATCTTGCAGAGTTCATT
TCCACATTCTTCTTCGTCTTCGCTTCTGTTGGAGCCACAATGTCCACGCGTAAGTTTTCATTTTCTTGTTTCATC
AAAGGGTTGGAACCACAATACCAGTGACGAAAATGGGTTAAAATCGACCCCATATTTGTTAAAAATTTGATTTCT
AATTGATTGTTTTAATAGGGCTTAGTTTGTTATTGTTTTGCTTCAATATGATTCATTGTTCGTACCTGTGTCGTG
ATTACAGTGAAAATGATGCCGGATGCAGCATCAAATTCTTCCGGTCTGGTAGCTATTGCAGTTGCAAATGCCTTT
GCATTATCAGTAGCAGTGTACATTGCTTCTAATATCTCCGGTGGCCATGTGAATCCGGCTGTTACATTTGGAATG
GCTATCGGAGGCCACATAAGCATTCCGATGGCCATTTTCTACTGGATTTCTCAGACCCTCGGCTCTGTCATAGCT
TGCATTCTCTTAAAAGTGACCACTGTTGGACAGGTACTAAGAGTTGTCAATGCTTTAGAATGTACAGTCAAATGG
GTAAAGCCACGTTGCCTGAAAGATATTAACAGGTTTCTTGACATAATGGGTGTTGACAAGATATGATTGAATGAA
AAAGTTTACTGTTTTAAACAATCTATTAATAGAATGTTATGTGACATTATGTCAATGATTATATCAACATCTATA
GCACTGTAACATGATTGTCACTGGACACGACACTATCCGTTTGTGCATCTCTTTCTAGCAATGATCTCAAGAGTC
TTTTCTTCTTATTTCATTTTTGCAGCACGTTCCAACGCACTCAATTCCACAAGAAATGACCGGTTTTGGTGCAGC
CATACTGGAAGGCGTGATGACATTTACATTGGTGTACACAGTATATGCTGCTGCTGATCCGAGGCGAGGTTCCAT
GTCAATAATTGGCCCGTTGGCCATAGGGCTGGTGTCAGGGGCTAACGTACTGGCATCGGGACCTTTTACCGGTGG
CTCAATGAACCCCGCCTACTCCTTCGGTTCTGCACTTGTTGGAGGAAGTTTCAAGAATCAAGCAGTCTATTGGAT
TGGACCATTGATTGGAGCAGCCACTGCAGGAATTTTATATGAGCATGTAGTTTTCCCTGCTCAACAAGAGCCTGA
TTCATTGAGGGGAATTCCTCATGGAGTTGAATTT**TAA**

>*Oleur*TIP5;1

ATGGCGTCCTTAAAATCCCGTTTGGAGCACACTGTTACACCCACTGCCCTCAGATCGTATCTTGCAGAGTTCATT
TCCACATTCTTCTTCGTCTTCGCTTCTGTTGGAGCCACAATGTCCACGCTGAAAATGATGCCGGATGCAGCATCA
AATTCTTCCGGTCTGGTAGCTATTGCAGTTGCAAATGCCTTTGCATTATCAGTAGCAGTGTACATTGCTTCTAAT
ATCTCCGGTGGCCATGTGAATCCGGCTGTTACATTTGGAATGGCTATCGGAGGCCACATAAGCATTCCGATGGCC
ATTTTCTACTGGATTTCTCAGACCCTCGGCTCTGTCATAGCTTGCATTCTCTTAAAACACGTTCCAACGCACTCA
ATTCCACAAGAAATGACCGGTTTTGGTGCAGCCATACTGGAAGGCGTGATGACATTTACATTGGTGTACACAGTA
TATGCTGCTGCTGATCCGAGGCGAGGTTCCATGTCAATAATTGGCCCGTTGGCCATAGGGCTGGTGTCAGGGGCT
AACGTACTGGCATCGGGACCTTTTACCGGTGGCTCAATGAACCCCGCCTACTCCTTCGGTTCTGCACTTGTTGGA
GGAAGTTTCAAGAATCAAGCAGTCTATTGGATTGGACCATTGATTGGAGCAGCCACTGCAGGAATTTTATATGAG
CATGTAGTTTTCCCTGCTCAACAAGAGCCTGATTCATTGAGGGGAATTCCTCATGGAGTTGAATTT**TAA**

>*Oleur*TIP5;1

MASLKSRLEHTVTPTALRSYLAEFISTFFFVFASVGATMSTLKMMPDAASNSSGLVAIAVANAFALSVAVYIASN
ISGGHVNPAVTFGMAIGGHISIPMAIFYWISQTLGSVIACILLKHVPTHSIPQEMTGFGAAILEGVMTFTLVYTV
YAAADPRRGSMSIIGPLAIGLVSGANVLASGPFTGGSMNPAYSFGSALVGGSFKNQAVYWIGPLIGAATAGILYE
HVVFPAQQEPDSLRGIPHGVEF*

*Oleur*TIP5;2 Oleur061Scf0430g00024.1

>Oleur061Scf0430:47031..48350 (- strand) class=gene length=1320

>*Oleur*TIP5;2
ATGGCGTCCTTAAAATCCCGTTTGGGGCACACTGTTACACCCACTGCCCTCAGATCGTATCTTGCAGAGTTCATT
TCCACATTCTTCTTCGTCTTCGCCTCGGTTGGAGCCACTATGTCCACGCGTAAGTTTTCATTTTCTTGTTTCATC
AAAGGGTTGGAACCACAATACCAGTGACGAAAATGGGTTAAAATCGACCCCATATTTGTTAAAAATTTGATTTCT
AATTGATTGTTTTAATAGGGCGTAGTTTGTTATTGTTTTGCTTCAGTATGATTCATTGTTCGTACCTGTATCGTG
ATTACAGTGAAAATGATGCCGGATGCAGCATCAAATTCTTCCGGTCTGGTAGCTATTGCAGTTGCAAACGCCTTT
GCATTATCAGTAGCAGTGTACATTGCTTCTAATATCTCCGGTGGCCATGTGAATCCGGCTGTTACATTTGGAATG
GCTATCGGAGGCCACATAAGCATTCCGATGGCCATTTTCTACTGGATTTCTCAGACCCTCGGCTCTGTCATAGCT
TGCATTCTCTTAAAAGTGACCACTGTTGGAGAGGTACTAAGAGTTGTCAATGCTTTAGAATGTACAGTCAAATGG
GTAAAGCCACGTTGCCTGAAAGATATTAACAGGTTTCTTGACACAATGGGTGTTGACAAGATATGATTGAATGAA
AAAGTTTACTGTTTTAAACAATCTATTAATAGAATGTTATGTGATATTATGTCAATGATTATGTCAACATCTATA
GATCTGTAACATGATTGTCACTGGACACGACGTGCATCTCTTTCTAGCAATGATCTCAAAAGTCTTTTCTTCTTT
TTTCATTTTTGCAGCACGTTCCAACGCACTCAATTCCACAAGAAATGACCGGTTTCGGTGCAGCCATACTGGAAG
GCGTGACGACATTTACATTGGTGTACACAGTATATGCTGCTGCTGATCCGAGGCGAGGTTCCATGTCAATAATTG
GCCCGTTGGCCATAGGGCTGGTGTCAGGGGCTAACGTGCTGGCATTGGGACCTTTCACCGGTGGTTCGATGAATC
CCTCCTACTCCTTCGGTTCTGCACTTGTTGGAGGAAGTTTCAAGAATCAAGCAGTCTATTGGATTGGACCATTGA
CTGGAGCAGCCACTGCAGGAATTTTATATGAGCATGTAGTTTTCCCTGCTCAACAAGAGCAGGATTCATTGAGGG
GAATTGCTCATGGAGTTGAATTT**TAA**
>*Oleur*TIP5;2
ATGGCGTCCTTAAAATCCCGTTTGGGGCACACTGTTACACCCACTGCCCTCAGATCGTATCTTGCAGAGTTCATT
TCCACATTCTTCTTCGTCTTCGCCTCGGTTGGAGCCACTATGTCCACGCTGAAAATGATGCCGGATGCAGCATCA
AATTCTTCCGGTCTGGTAGCTATTGCAGTTGCAAACGCCTTTGCATTATCAGTAGCAGTGTACATTGCTTCTAAT
ATCTCCGGTGGCCATGTGAATCCGGCTGTTACATTTGGAATGGCTATCGGAGGCCACATAAGCATTCCGATGGCC
ATTTTCTACTGGATTTCTCAGACCCTCGGCTCTGTCATAGCTTGCATTCTCTTAAAACACGTTCCAACGCACTCA
ATTCCACAAGAAATGACCGGTTTCGGTGCAGCCATACTGGAAGGCGTGACGACATTTACATTGGTGTACACAGTA
TATGCTGCTGCTGATCCGAGGCGAGGTTCCATGTCAATAATTGGCCCGTTGGCCATAGGGCTGGTGTCAGGGGCT
AACGTGCTGGCATTGGGACCTTTCACCGGTGGTTCGATGAATCCCTCCTACTCCTTCGGTTCTGCACTTGTTGGA
GGAAGTTTCAAGAATCAAGCAGTCTATTGGATTGGACCATTGACTGGAGCAGCCACTGCAGGAATTTTATATGAG
CATGTAGTTTTCCCTGCTCAACAAGAGCAGGATTCATTGAGGGGAATTGCTCATGGAGTTGAATTT**TAA**
>*Oleur*TIP5;2
MASLKSRLGHTVTPTALRSYLAEFISTFFFVFASVGATMSTLKMMPDAASNSSGLVAIAVANAFALSVAVYIASN
ISGGHVNPAVTFGMAIGGHISIPMAIFYWISQTLGSVIACILLKHVPTHSIPQEMTGFGAAILEGVTTFTLVYTV
YAAADPRRGSMSIIGPLAIGLVSGANVLALGPFTGGSMNPSYSFGSALVGGSFKNQAVYWIGPLTGAATAGILYE
HVVFPAQQEQDSLRGIAHGVEF*

*Oleur*TIP5;3a Oleur061Scf7085g01025.1

>Oleur061Scf7085:145441..146632 (- strand) class=gene length=1192

>*Oleur*TIP5;3a
ATGGCTTCTCTGAAATCCCGTTTGGAGCATTCAGTCACTGCCATTGCTCTTAGATCGTATCTTGCTGAGTTTATC
TCTACATTCTTCTTCGTCTTTGCTGCTGCTGGGGCAACAATGTCTTCGCGTAAGCCAATTTTCTTCATTTTTAAT
TTGGAAATTAATTGTTAATCCATTTCGTTTGATCATGGATCGTACCTTTTCCCTTTACATGATTTTTTAATGATG
TTTATTGATGTCATAAAAACAGGGAAAATGATGCCGGATGCAGCATCGGATTCCTCGAGTCTTGTTGCAATTGCA
GTTGCGAATGCGTTTGCACTGTCAGTTACAGTGTATATTTCTGCCAATATTTCCGGGGGCCACGTGAATCCGGCT
GTCACATTTGGAATGGTTGTGGGAGGCCATATTAGTATTCCTATGGCTATTTTCTATTCGATTTCGCAAACGTTT
GGTTCTGTCATGGCATGCCTGCTATTAAAAGCAACCACCGCAGGACAGGTACGTCACTCAAAGTCCCAATGAGTA
CCGCGTACTAAATGCATCAAAAATTGATTAAATGTGTGTCTGAGTGTAAGATAAACAGACAAAAAGTTTGATTAC
CCGACTAATTCTACTGATTACAACATACTAAACATGCCACTATCGACTACCCATCAGATGTACAGATGGAAAGTT
GCATCTTGGTGACTCAAATATCACATTATCTTTAACAGATACTTCATAATTGTTTTGCAGCATGTTCCAACTCAT
GCAATAGAACATGAGATGACTGGTTTTGGAGGAGCAGTGCTAGAGGGTGTTATGACTTTTGCATTGGTGTACACG
GTTTATGTAGCCTCGAACCTGAGGCCGGTTTCTGCCGGTGCCATCGGCCCCTTGGCAATTGGGCTTATTGCGGGG
GCTAACGTTCTTGCATCAGGACCGTTTACCGGTGGATCCATGAACCCGGCATACGCATTCGGTTCAGCCATTGTT
GGAGGGAGTTTTAAGAATCAAGCAGTATATTGGGTTGGACCATTGTTTGGCGCAGCCATTGCTGGGATTTTACAT
GATAATGTAGTCTGTCCTGCTCCAGTGCCTGATTCCTTTAGGGGGAATGCTGATGGAGTGGGAGTT**TAA**
>*Oleur*TIP5;3a
ATGGCTTCTCTGAAATCCCGTTTGGAGCATTCAGTCACTGCCATTGCTCTTAGATCGTATCTTGCTGAGTTTATC
TCTACATTCTTCTTCGTCTTTGCTGCTGCTGGGGCAACAATGTCTTCGCGGAAAATGATGCCGGATGCAGCATCG
GATTCCTCGAGTCTTGTTGCAATTGCAGTTGCGAATGCGTTTGCACTGTCAGTTACAGTGTATATTTCTGCCAAT
ATTTCCGGGGGCCACGTGAATCCGGCTGTCACATTTGGAATGGTTGTGGGAGGCCATATTAGTATTCCTATGGCT
ATTTTCTATTCGATTTCGCAAACGTTTGGTTCTGTCATGGCATGCCTGCTATTAAAAGCAACCACCGCAGGACAG

CATGTTCCAACTCATGCAATAGAACATGAGATGACTGGTTTTGGAGGAGCAGTGCTAGAGGGTGTTATGACTTTT
GCATTGGTGTACACGGTTTATGTAGCCTCGAACCTGAGGCCGGTTTCTGCCGGTGCCATCGGCCCCTTGGCAATT
GGGCTTATTGCGGGGGCTAACGTTCTTGCATCAGGACCGTTTACCGGTGGATCCATGAACCCGGCATACGCATTC
GGTTCAGCCATTGTTGGAGGGAGTTTTAAGAATCAAGCAGTATATTGGGTTGGACCATTGTTTGGCGCAGCCATT
GCTGGGATTTTACATGATAATGTAGTCTGTCCTGCTCCAGTGCCTGATTCCTTTAGGGGGAATGCTGATGGAGTG
GGAGTT**TAA**

>*Oleur*TIP5;3a
MASLKSRLEHSVTAIALRSYLAEFISTFFFVFAAAGATMSSRKMMPDAASDSSSLVAIAVANAFALSVTVYISAN
ISGGHVNPAVTFGMVVGGHISIPMAIFYSISQTFGSVMACLLLKATTAGQHVPTHAIEHEMTGFGGAVLEGVMTF
ALVYTVYVASNLRPVSAGAIGPLAIGLIAGANVLASGPFTGGSMNPAYAFGSAIVGGSFKNQAVYWVGPLFGAAI
AGILHDNVVCPAPVPDSFRGNADGVGV*

*Oleur*TIP5;3b Oleur061Scf2746g00014.1

>Oleur061Scf2746:4573..5762 (- strand) class=gene length=1190

>*Oleur*TIP5;3b
ATGGCTTCTCTGAAATCCCGTTTGGAGCATTCAGTCACTGCCATTGCTCTTAGATCGTATCTTGCTGAGTTTATC
TCTACATTCTTCTTCGTCTTTGCTGCTGCTGGGGCAACAATGTCTTCGCGTAAGCCAATTTTCTTCATTTTTAAT
TTGGAAATTAATTGTTAATCCATTTCGTTTGATCATGGATCGTACCTTTTCCCTTTACATGATTTTTTAATGATG
TTTATTGATGTCATAAAAACAGGGAAAATGATGCCGGATGCAGCATCGGATTCCTCGAGTCTTGTTGCAATTGCA
GTTGCGAATGCGTTTGCACTGTCAGTTACAGTGTATATTTCTGCCAATATTTCCGGGGGCCACGTGAATCCGGCT
GTCACATTTGGAATGGTTGTGGGAGGCCATATTAGTATTCCTATGGCTATTTTCTATTCGATTTCGCAAACGTTT
GGTTCTGTCATGGCATGCCTGCTATTAAAAGCAACCACCGCAGGACAGGTACGTCACTCAAAGTCCCAATGAGTA
CCGCATACTAAATGCATCAAAAATTGATTATATGTGTCTGAGTGTAAGATAAATGGACAAAAAGTTTGATTACCC
GACTAATTCAACTGATTACAACATACTAAACATGCCACTATCGACTACCCATCAGATGTACAGATGGAAAGTTGC
ATCTTGGTGACTCAAATATCACATTATCTTTAACAGATACTTCATAATTGTTTTGCAGCATGTTCCAACTCATGC
AATAGAACATGAGATGACTGGTTTTGGAGGAGCAGTGCTAGAGGGTGTTATGACTTTTGCATTGGTGTACACGGT
TTACGTAGCCTCTAACCTGAGGCCGGTTTCTGCCGGTGCCATCGGCCCCTTGGCAATTGGGCTTATTGCAGGGGC
CAACGTTCTTGCATCAGGACCGTTTACCGGTGGATCCATGAACCCGGCATACGCATTCGGTGCAGCCATTGTTGG
AGGGAGTTTTAAGAATCAAGCAGTATATTGGGTTGGACCATTGTTTGGCGCAGCCATTGCTGGGATTTTACATGA
TAATGTAGTCTGTCCTGCTCCAGTGCCTGATTCCTTTAGGGGGAATGCTGATGGAGTGGGAGTT**TAA**
>*Oleur*TIP5;3b
ATGGCTTCTCTGAAATCCCGTTTGGAGCATTCAGTCACTGCCATTGCTCTTAGATCGTATCTTGCTGAGTTTATC
TCTACATTCTTCTTCGTCTTTGCTGCTGCTGGGGCAACAATGTCTTCGCGGAAAATGATGCCGGATGCAGCATCG
GATTCCTCGAGTCTTGTTGCAATTGCAGTTGCGAATGCGTTTGCACTGTCAGTTACAGTGTATATTTCTGCCAAT
ATTTCCGGGGGCCACGTGAATCCGGCTGTCACATTTGGAATGGTTGTGGGAGGCCATATTAGTATTCCTATGGCT
ATTTTCTATTCGATTTCGCAAACGTTTGGTTCTGTCATGGCATGCCTGCTATTAAAAGCAACCACCGCAGGACAG
CATGTTCCAACTCATGCAATAGAACATGAGATGACTGGTTTTGGAGGAGCAGTGCTAGAGGGTGTTATGACTTTT
GCATTGGTGTACACGGTTTACGTAGCCTCTAACCTGAGGCCGGTTTCTGCCGGTGCCATCGGCCCCTTGGCAATT
GGGCTTATTGCAGGGGCCAACGTTCTTGCATCAGGACCGTTTACCGGTGGATCCATGAACCCGGCATACGCATTC
GGTGCAGCCATTGTTGGAGGGAGTTTTAAGAATCAAGCAGTATATTGGGTTGGACCATTGTTTGGCGCAGCCATT
GCTGGGATTTTACATGATAATGTAGTCTGTCCTGCTCCAGTGCCTGATTCCTTTAGGGGGAATGCTGATGGAGTG
GGAGTT**TAA**
>*Oleur*TIP5;3b
MASLKSRLEHSVTAIALRSYLAEFISTFFFVFAAAGATMSSRKMMPDAASDSSSLVAIAVANAFALSVTVYISAN
ISGGHVNPAVTFGMVVGGHISIPMAIFYSISQTFGSVMACLLLKATTAGQHVPTHAIEHEMTGFGGAVLEGVMTF
ALVYTVYVASNLRPVSAGAIGPLAIGLIAGANVLASGPFTGGSMNPAYAFGAAIVGGSFKNQAVYWVGPLFGAAI
AGILHDNVVCPAPVPDSFRGNADGVGV*

*Oleur*XIP1;1a Oleur061Scf1407g06005.1

>Oleur061Scf1407:601677..603499 (- strand) class=gene length=1823

>OleurXIP1;1a
ATGGCTGCTAATATTTGGAATGTACATGGAGTAGATGATGAAGAAAGCCAAACCAGTTTGAGCAAAATTCAGCCT
GTCCCCTCTACACCAGTGCAAGTTCTTTTTTTCTTAAATCTCAAGTTCAATATCTTCAGTTTCTTGTCCTTTTAT
CCTGAAAAAATTTGAGGGTATTTCTTAGTTTCTTACATTTTGTTTGGCAGGACACAACAATTGACAGCTGATCTC
GATAAGAAGAAGCAAACTCCTCTTACCTCGAGTCAAATGCTTGGTTTGAACGAGCTTTTCTCCCTAGACGTTAGT
ACATATCCCGGACTTGCAAATTTAGTTCTTCTTCCTTTTAATTACTGTATTGTCTTATAAACAAGACTGTCCT

ATATAATCCAAGTAACTTAAGTTCTCTTGATAACTGAAGGTTTGGAGGGCATCGGTGGGAGAGCTTATTGGATCA
GCAGTTCTAGTATTCATGATTGACACTATTGTGATCTCAACCTTTGAGACTGATATCAAAATGCCAAACCTCATA
ATCTCAATTCTCATTGCCATTGTCGTTGCAATTCTCCTCCTCGCTGTACATCCTGTTTCCGGTGGCCACATCAAC
CCCGTTATCTCCTTTTCGGCTGGGCTAGTAGGCCTTATCTCCATGTCACGCGCTATAGTTTACATCCTAGCCCAA
TGCATTGGTGCAATACTTGGTGCACTGGCCCTCAAAGCCGTAGTGAGCAGCACCATTGAACAAAACTTTTCACTT
GGAGGTTGCACTCTAACAGTGATTGCACCTGGACCCAATGGACCCATAACCCTTGGCCTTGAGACGGCACAAGCC
TTTTGGCTCGAGATTTTCTGCACCTTTGTTTTCCTTTTTGCTTCAATTTGGATGGCATATGATCATCGCCAAGCA
AAGAATTTAGGCCTTGTTCTTGTTTTTTCAATTATAGGACTTGTGCTAGGCCTCTTAGTGTTTATTTCCACCACT
GTCACTGCCAAAAAGGGCTATGCCGGGGCAGGAATGAACCCTGCTAGGTGCCTTGGGGCTGCAATTGTTAGAGGT
GGCCACCTCTGGGATGGCCACTGGGTGTTTTGGGCCGGGCCTGCTATTGCTTGTGTTGCCTTCTATTTATACACA
AAGATAATTCCACGGCAGCACCATCATGCAAAGGGCTATGGACATGATTTCTTCGATACTGTGAAGGCTATGCTT
GGATTTGTTGATCAC**TGA**

>*Oleur*XIP1;1a

ATGGCTGCTAATATTTGGAATGTACATGGAGTAGATGATGAAGAAAGCCAAACCAGTTTGAGCAAAATTCAGCCT
GTCCCCTCTACACCAGTGCAAGTTCTTTTTTTCTTAAATCTCAAGTTCAATATCTTCAGTTTCTTGACACAACAA
TTGACAGCTGATCTCGATAAGAAGAAGCAAACTCCTCTTACCTCGAGTCAAATGCTTGGTTTGAACGAGCTTTTC
TCCCTAGACGTTTGGAGGGCATCGGTGGGAGAGCTTATTGGATCAGCAGTTCTAGTATTCATGATTGACACTATT
GTGATCTCAACCTTTGAGACTGATATCAAAATGCCAAACCTCATAATCTCAATTCTCATTGCCATTGTCGTTGCA
ATTCTCCTCCTCGCTGTACATCCTGTTTCCGGTGGCCACATCAACCCCGTTATCTCCTTTTCGGCTGGGCTAGTA
GGCCTTATCTCCATGTCACGCGCTATAGTTTACATCCTAGCCCAATGCATTGGTGCAATACTTGGTGCACTGGCC
CTCAAAGCCGTAGTGAGCAGCACCATTGAACAAAACTTTTCACTTGGAGGTTGCACTCTAACAGTGATTGCACCT
GGACCCAATGGACCCATAACCCTTGGCCTTGAGACGGCACAAGCCTTTTGGCTCGAGATTTTCTGCACCTTTGTT
TTCCTTTTTGCTTCAATTTGGATGGCATATGATCATCGCCAAGCAAAGAATTTAGGCCTTGTTCTTGTTTTTTCA
ATTATAGGACTTGTGCTAGGCCTCTTAGTGTTTATTTCCACCACTGTCACTGCCAAAAAGGGCTATGCCGGGGCA
GGAATGAACCCTGCTAGGTGCCTTGGGGCTGCAATTGTTAGAGGTGGCCACCTCTGGGATGGCCACTGGGTGTTT
TGGGCCGGGCCTGCTATTGCTTGTGTTGCCTTCTATTTATACACAAAGATAATTCCACGGCAGCACCATCATGCA
AAGGGCTATGGACATGATTTCTTCGATACTGTGAAGGCTATGCTTGGATTTGTTGATCAC**TGA**

>*Oleur*XIP1;1a

MAANIWNVHGVDDEESQTSLSKIQPVPSTPVQVLFFLNLKFNIFSFLTQQLTADLDKKKQTPLTSSQMLGLNELF
SLDVWRASVGELIGSAVLVFMIDTIVISTFETDIKMPNLIISILIAIVVAILLLAVHPVSGGHINPVISFSAGLV
GLISMSRAIVYILAQCIGAILGALALKAVVSSTIEQNFSLGGCTLTVIAPGPNGPITLGLETAQAFWLEIFCTFV
FLFASIWMAYDHRQAKNLGLVLVFSIIGLVLGLLVFISTTVTAKKGYAGAGMNPARCLGAAIVRGGHLWDGHWVF
WAGPAIACVAFYLYTKIIPRQHHHAKGYGHDFFDTVKAMLGFVDH*

*Oleur*XIP1;1b Oleur061Scf4628g08012.1

>Oleur061Scf4628:814896..816717 (+ strand) class=gene length=1822

>*Oleur*XIP1;1b

ATGGCTGCTAATATTTGGAATGTACATGGAGTAGATGATGAAGAAAGCCAAACCAGTTTGAGCAAAATTCAGCCT
GTCCCCTCTACACCAATGCAAGTTCTTTTTTCTTAAATTTCAAGTTCAATATCTTCAGTTTCTTGTCCTTTTTTC
CTGAAAAATTTGAGGGTATTTCTTAGTTTCTTACATTTTGCTTGGCAGGACACAACAATTGACAGCTGATCTTG
ATAAGAAGAAGCAAACTCCTCTTACCTCGAGTCAAATGCTTGGTTTGAACGAGCTTTTCTCCCTAGACGTTAGTA
TATATCCCGGACTTGTAAATTTTAGTTTCTTCTTCCTTTTAATTACTGTATAGTCTTATAAACAAGACTGTCCTA
TATAATCCAAGTAACTTAAGTTTTCTTGATAACTGAAGGTTTGGAGGGCATCGGTGGGAGAGCTTATTGGATCAG
CAGTTCTAGTATTCATGATTGACACTATTGTGATCTCAACCTTTGAGACTGATATCAAAATGCCAAACCTCATAA
TCTCAATTCTCATTGCAATTGTCGTTGCAATTCTCCTCCTCGCTGTACATCCTGTTTCCGGTGGCCACATCAACC
CCGTTATCTCCTTTTCGGCTGGGCTAGTAGGCCTTATCTCCATGTCACGCGCTATAGTTTACATCCTAGCCCAAT
GCATTGGTGCAATACTTGGTGCACTGGCCCTCAAAGCCGTAGTGAGCAGCACCATTGAACAAAACTTTTCACTTG
GAGGTTGCACTCTAACAGTGATTGCACCTGGACCCAATGGACCCATAACCCTTGGCCTTGAGACGGCACAAGCCT
TTTGGCTCGAGATTTTCTGCACCTTTGTTTTCCTTTTTGCTTCAATTTGGATGGCATATGATCATCGCCAAGCAA
AGAATTTAGGCCTTGTTCTTGTTTTTTCAATTATAGGACTTGTGCTAGGCCTCTTAGTGTTTATATCCACCACTG
TCACTGCCAAAAAGGGCTATGCCGGGGCAGGAATGAACCCTGCTAGGTGCCTTGGGGCTGCAATTGTTAGAGGTG
GCCACCTCTGGGATGGCCACTGGGTGTTTTGGGCCGGGCCTGCTATTGCTTGTGTTGCCTTCTATTTATACACAA
AGATAATTCCACGGCAGCACCATCATGCAAAGGGCTATGGACATGATTTCTTCGATACTGTGAAGGCTATGCTTG
GATTTGTTGATCAC**TGA**

>*Oleur*XIP1;1b

ATGGCTGCTAATATTTGGAATGTACATGGAGTAGATGATGAAGAAAGCCAAACCAGTTTGAGCAAAATTCAGCCT
GTCCCCTCTACACCAATGCAATTCAATATCTTCAGTTTCTTGACACAACAATTGACAGCTGATCTTGATAAGAAG
AAGCAAACTCCTCTTACCTCGAGTCAAATGCTTGGTTTGAACGAGCTTTTCTCCCTAGACGTTTGGAGGGCATCG
GTGGGAGAGCTTATTGGATCAGCAGTTCTAGTATTCATGATTGACACTATTGTGATCTCAACCTTTGAGACTGAT
ATCAAAATGCCAAACCTCATAATCTCAATTCTCATTGCAATTGTCGTTGCAATTCTCCTCCTCGCTGTACATCCT

GTTTCCGGTGGCCACATCAACCCCGTTATCTCCTTTTCGGCTGGGCTAGTAGGCCTTATCTCCATGTCACGCGCT
ATAGTTTACATCCTAGCCCAATGCATTGGTGCAATACTTGGTGCACTGGCCCTCAAAGCCGTAGTGAGCAGCACC
ATTGAACAAAACTTTTCACTTGGAGGTTGCACTCTAACAGTGATTGCACCTGGACCCAATGGACCCATAACCCTT
GGCCTTGAGACGGCACAAGCCTTTTGGCTCGAGATTTTCTGCACCTTTGTTTTCCTTTTTGCTTCAATTTGGATG
GCATATGATCATCGCCAAGCAAAGAATTTAGGCCTTGTTCTTGTTTTTTCAATTATAGGACTTGTGCTAGGCCTC
TTAGTGTTTATATCCACCACTGTCACTGCCAAAAAGGGCTATGCCGGGGCAGGAATGAACCCTGCTAGGTGCCTT
GGGGCTGCAATTGTTAGAGGTGGCCACCTCTGGGATGGCCACTGGGTGTTTTGGGCCGGGCCTGCTATTGCTTGT
GTTGCCTTCTATTTATACACAAAGATAATTCCACGGCAGCACCATCATGCAAAGGGCTATGGACATGATTTCTTC
GATACTGTGAAGGCTATGCTTGGATTTGTTGATCAC**TGA**

>*Oleur*XIP1;1b
MAANIWNVHGVDDEESQTSLSKIQPVPSTPMQFNIFSFLTQQLTADLDKKKQTPLTSSQMLGLNELFSLDVWRAS
VGELIGSAVLVFMIDTIVISTFETDIKMPNLIISILIAIVVAILLLAVHPVSGGHINPVISFSAGLVGLISMSRA
IVYILAQCIGAILGALALKAVVSSTIEQNFSLGGCTLTVIAPGPNGPITLGLETAQAFWLEIFCTFVFLFASIWM
AYDHRQAKNLGLVLVFSIIGLVLGLLVFISTTVTAKKGYAGAGMNPARCLGAAIVRGGHLWDGHWVFWAGPAIAC
VAFYLYTKIIPRQHHHAKGYGHDFFDTVKAMLGFVDH*

*Oleur*XIP1;2a Oleur061Scf0694g04001.1 corrected

>Oleur061Scf0694:467273..468886 (- strand) class=gene length=1614

>*Oleur*XIP1;2a
ATGGCTGGTAATAATGGGCATGTAAATGTAGAATATGATGAAGAAGGCTTGAGTGGTGCAAGCAAAATTCAACCT
GTCTTCTCTACTCCAATGCAAGTTCATTTCTTTCTTTGAACTTTTATATTATTATATTAAATTTTCAAAATCTTG
TTATAGTTGATAAAAATAGGGGGTGGACATGGTTCCGTTTAATCCTAGTTCCAAGATAATCGAATCGAACTCAAT
CATGAATCAATTATTTTTTTGGTAATGGAACTAATGGTTTTTATTTTATTTTATTTTCTAAAGATATGGAACCGA
GTTAAGTTCGATTTCATTTAATTTTGGTCTGGTTTGGTTTGATTCCATTTTTAAGTAACGGAACCTGAACCGAAT
CCTTAAAGAAGAAGAAGAAGAAATCATTGGTTTCATGATTAAAAAAGCCATTGGTTCGTGGTTCGCTTCGATTCT
CTTGGAATCAAAACCAAATGGACCATGTCTACCCTAGGTAAAAATATAGTCAATTTAGATTTTTAATAGTTATAG
TGCTTGTTTGGTAGAGATTTTGTTAATTGATTTTTTGACCGATAGGATTCTACTTGTGAGAAAAGAATTCAAAAA
CGACGGCTGGAATAGTGAAATAATGGTAAAAAAACTAATGTTAATTGGATTAAAATCTCTACCAAACATGCTCAT
AGCAGTGAACTGAAAGAATAGATAGGAAAGAACAATCACTCTTTTCTTTTTCTTTTTCCTTTTTTCTTTTTCCTT
TTTTTCCTTTTTTTCTTTCTTTCTTTCTTTCTTTCTTTTTTTGAAGATCATATGTTTTCCAATCTTGTTCATGTC
ATCGTAGCTGACCTTAAATTTATTAAGAATATTATGTTTCTTCAAATCTCGTAGTTGATACGTCTCCGTGAACTA
AATAGAAAGATGAAAATGATTCAAGTAGTCCATCCAAAATTTATAAAGAATAACCAATAGTTTATCTTTTGTTGA
GGTTTTCTTTAAAAATCTTGTTGTTCTGGGAAGTTAAAACTGTGTATGCTTAGCAGGCCAGAGAACAGGACAGAT
GAGGAGAAAATGAAGAAGACAACTTCTCTTACTCTGAGTGAATTGCTGGGTTTGGACGACTTCCTTTCCTCAGAT
GTTAGTACTTCTTTCCTCTTCAAGAAAATGTCTTTAGATTAGTGTTCAAAGATAAATTTTCATAAATAAACATGT
CATGTCTTCAATAATTTAAACTTTTAACAAGATAGTTCAACTTAGTATTGGAATGGCAAAGATCCACCGTTCAAG
TTTCACCAATTACTTACATGCGGAAAACTGGATTTAAATAAATCAATATGCCGTATATCTAACAATTTAAACTTT
TAAATGAGATAATATGAGTAAGAAAATATCATGTTATTTTTGTTTATTCTTGAAGGTTTGGAGAGCCTCAGTAGG
CGAGCTGGTTGGCACGGCCGTTCTTGTATTCATGCTTGACACTATTGTGATATCAACCATTGAGTCTGATATCAA
AATTCCAAACCTTGTACTATCAATCCTTGCAGCCATTATTATTGCTATACTACTTCTTGCTGTACATCCAGTTTC
CGGTGGCCACATCAACCCGGTTATCTCCTTCTCTGCCGCCCTGGTTGGGTTAATCTCTATGTCGAAAGCCTTCAT
ATACATTGTTGCACAATGTCTTGGAGCCGTGCTAGGTGCGGGTTGAAAACCCAATGAACAAACTTTATTTTGTTT
CAAAGTAGTCCCCTCACGTTTATGTTTTCTTTCAGCTTGATGACTATGGCAACATGTTCGACTTAAATTGGATGG
TCAAAGTGAACAAAAAGCAAACTACTTAGGGCCTGTTTGAAAGGGCCAATTTTCCATGGAAAATGGAAACATTTT
TCCCTTTTGGCCTGTTTGATTGCAATAATTTTCTAACCCCCACATGACCATTTTTCCTTGATTTTGTGGAAAACG
GGAATTGGTTTTCCAGCTGATTTTTTCTTTTTTTTTTTTAATATTTTTTGGCAATTGGAACAAATTCTATTTAT
AATTGTCAAAAATTCTATCATTTTAATTGACAATTTTCATGAAACACAAAGTCAAATGAACACTAAAAGATCCAA
AATTTCAACATTTTTTAAGTGAAAAATTATACCAATCAAACACATTTAATTGACAATTTCCAATAAAATAAGTTC
TAGACAATTCAATTCTCTGAAATTCTTTTTCTTTTCACCCAAATTTCATTCTTACAATCAAACGGATCCTTAGGG
TTATTCCCCCCTGTATAGAGAGTTTTAGCGCAGCACCCATGTGGTTGGAATTTTTTTTCCCAAAGCTCAATAGAA
TAATGCCAAGTGTCTCCTTTAAACAAATCTAGAAAGTTTGTCTATACACATGGCACGGTTCAACCACTTAAAAGT
ATGTTTCGTTTAGATTAAAATCATCACATATTTCAAAATCTTCTATTACAATATACTAAAAATCTAATTTTAATC
CTTACCAAACACCATCTAAATAAAGTTCGATAATGGGTTGAAATTTTGCTATGAGTTGAGCGACTAACAATATTT
GCTTACAGGTGCACTAGCTCTTAAAGCTGTGGTCAGTAGCACCACTGAAGAAACCTTCTCACTTGGCGGCTGCAC
CCTGACTGTGATTGCACCCGGCCTGAATGGACCTGCTACGGTAGGCCTCGAGACGGTCCAAGCCTTTTGGCTAGA
AATATTTTGCACCTTTGTGTTCCTTTTTGCTTCAATTTGGATGGCATATGATCCACGGCAAGCAAAGGAATTAGG
TCATGTCCTCATCTTTTCAATTGTGGGAGTTGTGTTAGGTCTCCTAGTATTTATCTCCACCACTGTCACAGCCAA
AAAGGGATATGCTGGGGCCGGGATGAACCCGGCTAGGTGTTTTGGCCCGGCGATTGTTAGAGGCGGCCACCTATG
GAATGTCTACTGGGTTTTCTGGGCTGGCCCGACCATTGCATGCGTGGCATTCTATTTGTACACACAGATTATTCC
ATGTCAGCACCACCGGTCGAAGTATTCTTATGATCATGATTTCTATAATGTTGTCAAGGCTATATTT**TGA**

>*Oleur*XIP1;2a
ATGGCTGGTAATAATGGGCATGTAAATGTAGAATATGATGAAGAAGGCTTGAGTGGTGCAAGCAAAATTCAACCT
GTCTTCTCTACTCCAATGCAAAACAGGACAGATGAGGAGAAAATGAAGAAGACAACTTCTCTTACTCTGAGTGAA
TTGCTGGGTTTGGACGACTTCCTTTCCTCAGATGTTTGGAGAGCCTCAGTAGGCGAGCTGGTTGGCACGGCCGTT
CTTGTATTCATGCTTGACACTATTGTGATATCAACCATTGAGTCTGATATCAAAATTCCAAACCTTGTACTATCA
ATCCTTGCAGCCATTATTATTGCTATACTACTTCTTGCTGTACATCCAGTTTCCGGTGGCCACATCAACCCGGTT
ATCTCCTTCTCTGCCGCCCTGGTTGGGTTAATCTCTATGTCGAAAGCCTTCATATACATTGTTGCACAATGTCTT
GGAGCCGTGCTAGGTGCACTAGCTCTTAAAGCTGTGGTCAGTAGCACCACTGAAGAAACCTTCTCACTTGGCGGC
TGCACCCTGACTGTGATTGCACCCGGCCTGAATGGACCTGCTACGGTAGGCCTCGAGACGGTCCAAGCCTTTTGG
CTAGAAATATTTTGCACCTTTGTGTTCCTTTTTGCTTCAATTTGGATGGCATATGATCCACGGCAAGCAAAGGAA
TTAGGTCATGTCCTCATCTTTTCAATTGTGGGAGTTGTGTTAGGTCTCCTAGTATTTATCTCCACCACTGTCACA
GCCAAAAAGGGATATGCTGGGGCCGGGATGAACCCGGCTAGGTGTTTTGGCCCGGCGATTGTTAGAGGCGGCCAC
CTATGGAATGTCTACTGGGTTTTCTGGGCTGGCCCGACCATTGCATGCGTGGCATTCTATTTGTACACACAGATT
ATTCCATGTCAGCACCACCGGTCGAAGTATTCTTATGATCATGATTTCTATAATGTTGTCAAGGCTATATTT**TGA**
>*Oleur*XIP1;2a
MAGNNGHVNVEYDEEGLSGASKIQPVFSTPMQNRTDEEKMKKTTSLTLSELLGLDDFLSSDVWRASVGELVGTAV
LVFMLDTIVISTIESDIKIPNLVLSILAAIIIAILLLAVHPVSGGHINPVISFSAALVGLISMSKAFIYIVAQCL
GAVLGALALKAVVSSTTEETFSLGGCTLTVIAPGLNGPATVGLETVQAFWLEIFCTFVFLFASIWMAYDPRQAKE
LGHVLIFSIVGVVLGLLVFISTTVTAKKGYAGAGMNPARCFGPAIVRGGHLWNVYWVFWAGPTIACVAFYLYTQI
IPCQHHRSKYSYDHDFYNVVKAIF*

*Oleur*XIP1;2b Oleur061Scf0386g00009.1 Corrected

>Oleur061Scf0386:689..2303 (- strand) class=gene length=1615

>*Oleur*XIP1;2b
ATGGCTGGTAATAATGGGCATGTAAATGTAGAATATGATGAAGAAGGCTTGAGTGGTGCAAGCAAAATTCAACCT
GTCTTCTCTACTCCAATGCAAGTTCATTTCTTTCTTTGAACTTTTATATTATTATATTAAATTTTCAAAATCTTG
TTATAGTTGATAAAAATAGGGGGTGGACATGGTTCCGTTTAATCCTAGTTCCAAGATAATCGAATCGAACTCAAT
CATGAATCAATTATTTTTTGGTAATGGAACTAATGGTTTTTATTTTATTTTATTTTCTAAAGATATGGAACCGAG
TTAAGTTCGATTTCATTTAATTTTGGTCTGGTTTGGTTTGATTCCATTTTTAAGTAACGGAACCTGAACCGAATC
TTAAAGAAGAAGAAGAAGAAATCATTGGTTTCATGATTAAAAAAGCCATTGGTTCGTGGTTCGCTTCGATTCTCT
TGGAATCAAAACCAAATGGACCATGTCTACCCTAGGTAAAAATATAGTCAATTTAGATTTTTAATAGTTATAGTG
CTTGTTTGGTAGAGATTTTGTTAATTGATTTTTTGACCGATAAGATTCTACTTGTGAGAAAAGAATTCAAAAACG
ACGGCTGGAATAGTGAAATAATGGTAAAAAAACTAATGTTAATTGGATTAAAATCTCTACCAAACATGCTCATAG
TAGTGAACTGAAAGAATAGATAGGAAAGAACAATCACTCTTTTCTTTTTCTTTTTCCTTTTTTCCTTTTCTTTTT
TTCCTTTTTTTCTTTCTTTCTTTCTTTCTTTTTTTGAAGATCATATGTTTTCCAATCTTGTTCATGTCATCGTAG
CTGACCTTAAATTTATTAAGAATATTATGTTTCTTCAAATCTCGTAGTTGATACGTCTCCGTGAACTAAATAGAA
AGATGAAAATGATTCAAGTAGTCCATCCAAAATTTATAAAGAATAACCAATAGTTTATCTTTTGTTGAGGTTTTC
TTTAAAAATCTTGTTGTTCTGGGAAGTTAAAACTGTGTATGCTTAGCAGGCCAGAGAACAGGACAGATGAGGAGA
AAATGAAGAAGACAACTTCTCTTACTCTGAGTGAATTGCTGGGTTTGGACGACTTCCTTTCCTCAGATGTTAGTA
CTTCTTTCCTCTTCAAGAAAATGTCTTTAGATTAGTGTTCAAAGATAAATTTTTCATAAATAAACATGTCATGTC
TTCAATAATTTAAACTTTTAACAAGATAGTTCAACTTAGTATTGGAATGGCAAAGATCCACCGTTCAAGTTTCAC
CAATTACTTACATGCGGAAAACTGGATTTAAATAAATCAATATGCCGTATATCTAACAATTTAAACTTTTAAATG
AGATAATATGAGTAAGAAAATATCATGTTATTTTTGTTTATTCTTGAAGGTTTGGAGAGCCTCAGTAGGCGAGCT
GGTTGGCACGGCCGTTCTTGTATTCATGCTTGACACTATTGTGATATCAACCATTGAGTCTGATATCAAAATTCC
AAACCTTGTACTATCAATCCTTGCAGCCATTATTATTGCTATACTACTTCTTGCTATACATCCAGTTTTCGGTGG
CCACATCAACCCGGTTATCTCCTTCTCTGCCGCCCTGGTTGGGTTAATCTCTATGTCGAAAGCCTTCATATACAT
TGTTGCACAATGTCTTGGAGCCGTGCTAGGTGCGGGTTGAAAACCCAATGAACAAACTTTATTTTGTTTCAAAGT
AGTCCCCTCACGTTTATGTTTTCTTTCAGCTTGATGACTATGGCAACATGTTCGACTTAAATTGGATGGTCAAAG
TGAACAAAAAGCAAACTACTTAGGGCCTGTTTGAAAGGGCCAATTTTCCATGGAAAATGGAAACATTTTTCCCTT
TTGGCCTGTTTGATTGCAATAATTTTCTAACCCCCACATGACCATTTTTCCTTGATTTTGTGGAAAACGGGAATT
GGTTTTCCAGCTGATTTTTTTTTTTTTTTTTTTAATATTTTTTGGCAATTGGAACAAATTCTATTTATAATTGTC
AAAAATTCTATCATTTTAATTGACAATTTTCATGAAACACAAAGTCAAATGAACACTAAAAGATCCAAAATTTCA
ACATTTTTTAAGTGAAAAATTATACCAATCAAACACATTTAATTGACAATTTCCAATAAAATAAGTTCTAGACAA
TTCAATTCTCTGAAATTCTTTTTCTTTTCACCCAAATTTCATTCTTACAATCAAACGGATCCTTAGGGTTATTCC
CCCCTGTATAGAGAGTTTTAGCGCAGCACCCATGTGGTTGGAATTTTTTTTCCGCAAAGCTCAATAGAATAATGC
CAAGTGTCTCCTTTTAAACAAATCTAGAAAGTTTGTCTATACACATGGCACGGTTCAACCACTTAAAAGTATGTT
TCGTTTAGATTAAAATCATCACATATTTCAAAATCTTCTATTACAATATACTAAAAATCTAATTTTAATCCTTAC
CAAACACCATCTAAATAAAGTTCGATAATGGGTTGAAATTTTGCTATGAGTTGAGCGACTAACAATATTTGCTTA
CAGGTGCACTAGCTCTTAAAGCTGTGGTCAGTAGCACCACTGAAGAAACCTTCTCACTTGGCGGCTGCACCCTGA
CTGTGATTGCACCCGGCCTGAATGGACCTGCTACGGTAGGCCTCGAGACGGTCCAAGCCTTTTGGCTAGAAATAT

TTTGCACCTTTGTGTTCCTTTTTGCTTCAATTTGGATGGCATATGATCCACGGCAAGCAAAGGAATTAGGTCATG
TCCTCATCTTTTCAATTGTGGGAGTTGTGTTAGGTCTCCTAGTATTTATCTCCACCACTGTCACAGCCAAAAAGG
GATATGCTGGGGCCGGGATGAACCCGGCTAGGTGTTTTGGCCCGGCGATTGTTAGAGGCGGCCACCTATGGAATG
TCTACTGGGTTTTCTGGGCTGGCCCGACCATTGCATGCGTGGCATTCTATTTGTACACACAGATTATTCCATGTC
AGCACCACCGGTCGAAGTATTCTTATGATCATGATTTCTATAATGTTGTCAAGGCTATATTT**TGA**

>*Oleur*XIP1;2b
ATGGCTGGTAATAATGGGCATGTAAATGTAGAATATGATGAAGAAGGCTTGAGTGGTGCAAGCAAAATTCAACCT
GTCTTCTCTACTCCAATGCAAAACAGGACAGATGAGGAGAAAATGAAGAAGACAACTTCTCTTACTCTGAGTGAA
TTGCTGGGTTTGGACGACTTCCTTTCCTCAGATGTTTGGAGAGCCTCAGTAGGCGAGCTGGTTGGCACGGCCGTT
CTTGTATTCATGCTTGACACTATTGTGATATCAACCATTGAGTCTGATATCAAAATTCCAAACCTTGTACTATCA
ATCCTTGCAGCCATTATTATTGCTATACTACTTCTTGCTATACATCCAGTTTTCGGTGGCCACATCAACCCGGTT
ATCTCCTTCTCTGCCGCCCTGGTTGGGTTAATCTCTATGTCGAAAGCCTTCATATACATTGTTGCACAATGTCTT
GGAGCCGTGCTAGGTGCACTAGCTCTTAAAGCTGTGGTCAGTAGCACCACTGAAGAAACCTTCTCACTTGGCGGC
TGCACCCTGACTGTGATTGCACCCGGCCTGAATGGACCTGCTACGGTAGGCCTCGAGACGGTCCAAGCCTTTTGG
CTAGAAATATTTTGCACCTTTGTGTTCCTTTTTGCTTCAATTTGGATGGCATATGATCCACGGCAAGCAAAGGAA
TTAGGTCATGTCCTCATCTTTTCAATTGTGGGAGTTGTGTTAGGTCTCCTAGTATTTATCTCCACCACTGTCACA
GCCAAAAAGGGATATGCTGGGGCCGGGATGAACCCGGCTAGGTGTTTTGGCCCGGCGATTGTTAGAGGCGGCCAC
CTATGGAATGTCTACTGGGTTTTCTGGGCTGGCCCGACCATTGCATGCGTGGCATTCTATTTGTACACACAGATT
ATTCCATGTCAGCACCACCGGTCGAAGTATTCTTATGATCATGATTTCTATAATGTTGTCAAGGCTATATTT**TGA**
>*Oleur*XIP1;2b
MAGNNGHVNVEYDEEGLSGASKIQPVFSTPMQNRTDEEKMKKTTSLTLSELLGLDDFLSSDVWRASVGELVGTAV
LVFMLDTIVISTIESDIKIPNLVLSILAAIIIAILLLAIHPVFGGHINPVISFSAALVGLISMSKAFIYIVAQCL
GAVLGALALKAVVSSTTEETFSLGGCTLTVIAPGLNGPATVGLETVQAFWLEIFCTFVFLFASIWMAYDPRQAKE
LGHVLIFSIVGVVLGLLVFISTTVTAKKGYAGAGMNPARCFGPAIVRGGHLWNVYWVFWAGPTIACVAFYLYTQI
IPCQHHRSKYSYDHDFYNVVKAIF*

***Oleur*XIP1;3 Oleur061Scf1977g00011.1**

>Oleur061Scf1977:43695..44812 (- strand) class=gene length=1118

>*Oleur*XIP1;3
ATGGCCGGTTATGATGGGCATGTAAATGTAGAATATGATGAAGAAAGCTTGAATAGTGCAAACAAAATTCAACCT
GTCTTCTCTACACCAATGCAAGTTCATTTTCTTTCCTTCAACTTTTCTTATTTATATATTTTTTCAAATCTTG
TTGTAGTTGGTAAAAAAATAGTCGATTTAGATTTTCCAATAGGTGTAACAGTGAAGCGAACGAAAAGATGAGAAA
GACGTTCACAGTAGATATGTATCCATATATAGTAGATTCCAAATTTATTAAGAATAATGCATAATTTATCATTTG
TTGTGGTTTCTTCAAAATTCTTGTCGTTTTGATAAGTTAAAACTTCGAACGTTTAGCAGGCCAGAGCACTGGACA
GATGAGCAGAAAAGGAAGAAGAATACTTCTCTTACACTGAGTGAATTGCTGGGCTTTGAAGACTTCCTTTCTTTA
GATGTTTGTACCTTTTTTTCCTCTTCAACCACCGCGCCCTGCCCCTCCCCACCCCCACTCAAAGATTAAATATAC
ATGATTAAACATGTCATTAGTAAAGTCTTCCGAATGATGCGATTCTATATAGTGTTAGAATGACAAAATCTTGCT
TTAAAGACTCGCCGCCGGACCGGTATGAAAATTATGCATATGATTGAGCCTTTTGATGAGAATATTTCAACCTAC
ATGTGAAAGGGCGTGCTAAAAATTGAATTTAAATAAATAAATTTTTTTCTATCTAAAAATTTAAACTTTTAAACA
AGATGATTCTTGTTTACTCTTGAAGGTTTGGAGAGCCTCAGTGGGAGAACTGATTGGCACAGCAGTTCTTGTGTT
CATGCTTGACAGTATTGTGATATCAACCAATGAGACTGATATCAAAATGCCAAACCTTGTATTGTCAATCCTTGC
AGCCATTATTATTGCAATACTACTTCTTGCAGTGCATCCTATTTCTGGAGGCCACATCAACCCAGTTATCTCCTT
CTCGGCTGCCCTAGTCGGGTTGATCTCTATGTCGAAAGCCTTAATCTACATTATTGCACAATGTCTTGGAGCCGC
GCTAGGTGAGGGTTAAACTCCCTATCGACGAACTTTATTTGTTCCAAAGTAGTCCATGAAGTTCATGTTGTCTT
CCAGTTTGATGACTAAAGTTAAAATTCTTGAAAAATGCTACTTGTACAGATTTTTTAACAGACGGGTTACAGGT
GCTAAGCTGATAAGATTTTATTGGTTGGTTTAACTTTGTTTTGTTGTCCCAAATGGTGTGAAATCTTGTCAACTT
AGCATTCTGTTAAAACAGTTTGTACAAGTAGCATTTCTCAAAATTCTTTAGTTTTCTAATAGAACGTTTGGCTT
AGATCAGATGATCAAGGTGAAACAAAAAGCAAACTCTAGTTATCACTCTAAAGCAAAGAAATGTTCGATAAATAA
TATGAAACCTCACTATAAATTGAGTGACTAACAATGTTTTTTACGCGACTACAGGTGCACTAGCTCTTAAAGCT
GTGGTTAGTAGCACCATTGAACAAACCTTCTCACTTGGAGGCTGCACCCTGACAGTGATTGCCCCTGGCCAGAAT
GGGCCCGTTACAATCGGCCTCGAGACTGCCCAAGCCTTTTGGCTAGAAATAATTTGTACCTTCGTACTCCTTTTT
GCTTCAGTTTGGATGGCATATGATCATCGCCAAGCAAAGGCATTAGGTCGTGTTCTTGTCTTTTCAATTGTGGGA
GTTGTGCTAGGTCTCTTAGTGTTCATCTCCACCACAGTCACGGCCAAAAAGGGCTACGCCGGGGCGGGGATGAAC
CCGGCTAGGTGTTTTGGTGCAGCAGTTGTTAGAGGTGGCCACCTATGGAATGGACACTGGATTTTCTGGGTGGGC
CCGGCCATTGCTTGCGTGGCCTTCTATTTGTACACGAAGATTATTCCACGTCAGCNATTTTGTGAA**TAA**
>*Oleur*XIP1;3
ATGGCCGGTTATGATGGGCATGTAAATGTAGAATATGATGAAGAAAGCTTGAATAGTGCAAACAAAATTCAACCT
GTCTTCTCTACACCAATGCAACACTGGACAGATGAGCAGAAAAGGAAGAAGAATACTTCTCTTACACTGAGTGAA

TTGCTGGGCTTTGAAGACTTCCTTTCTTTAGATGTTTGGAGAGCCTCAGTGGGAGAACTGATTGGCACAGCAGTT
CTTGTGTTCATGCTTGACAGTATTGTGATATCAACCAATGAGACTGATATCAAAATGCCAAACCTTGTATTGTCA
ATCCTTGCAGCCATTATTATTGCAATACTACTTCTTGCAGTGCATCCTATTTCTGGAGGCCACATCAACCCAGTT
ATCTCCTTCTCGGCTGCCCTAGTCGGGTTGATCTCTATGTCGAAAGCCTTAATCTACATTATTGCACAATGTCTT
GGAGCCGCGCTAGGTGCACTAGCTCTTAAAGCTGTGGTTAGTAGCACCATTGAACAAACCTTCTCACTTGGAGGC
TGCACCCTGACAGTGATTGCCCCTGGCCAGAATGGCCCGTTACAATCGGCCTCGAGACTGCCCAAGCCTTTTGG
CTAGAAATAATTTGTACCTTCGTACTCCTTTTTGCTTCAGTTTGGATGGCATATGATCATCGCCAAGCAAAGGCA
TTAGGTCGTGTTCTTGTCTTTTCAATTGTGGGAGTTGTGCTAGGTCTCTTAGTGTTCATCTCCACCACAGTCACG
GCCAAAAAGGGCTACGCCGGGGCGGGGATGAACCCGGCTAGGTGTTTTGGTGCAGCAGTTGTTAGAGGTGGCCAC
CTATGGAATGGACACTGGATTTTCTGGGTGGGCCCGGCCATTGCTTGCGTGGCCTTCTATTTGTACACGAAGATT
ATTCCACGTCAGCNATTTTGTGAA**TAA**

>*Oleur*XIP1;3

MAGYDGHVNVEYDEESLNSANKIQPVFSTPMQHWTDEQKRKKNTSLTLSELLGFEDFLSLDVWRASVGELIGTAV
LVFMLDSIVISTNETDIKMPNLVLSILAAIIIAILLLAVHPISGGHINPVISFSAALVGLISMSKALIYIIAQCL
GAALGALALKAVVSSTIEQTFSLGGCTLTVIAPGQNGPVTIGLETAQAFWLEIICTFVLLFASVWMAYDHRQAKA
LGRVLVFSIVGVVLGLLVFISTTVTAKKGYAGAGMNPARCFGAAVVRGGHLWNGHWIFWVGPAIACVAFYLYTKI
IPRQXFCE*

*Oleur*NIP1;1 Oleur061Scf1489g00038.1

>Oleur061Scf1489:7790..10379 (+ strand) class=gene length=2590

>*Oleur*NIP1;1

ATGGCTGAAATGTCCGGAGCAAATGGAGTGTCCCTGAGTATAAAGGATGATGTGAACGAGAAGAGCATTCAAGAT
TCTCCAGACTGGTTGCCTTTTATACAGAAGGTACATACGAGGTTTCCTTTTCTTTTCGACAACCTTAATCCTCCC
AACAGAACACAAACTGATATCTTCTATGTCCATAATTATATGTTCAATCTTTTCTTGGAGAGACGATGAAATCGA
TGCCCTCTTAATAAATGCTACAGTTAGTGGTTTATAAAGTACATAAGATTGTCCAACCAGGCAACAGTCTTTAGT
AATCAATATTACGAAAATGATGTATAGCTGCTTAATTTGACACTGGTAATTAATGTTGAAAATCTGATTGGCCAA
CTTATTTTGGTACTGTAGATAATTGCAGAGATTTTGGGTACATACTTCTTGATTTTCGCCGGTTGTGCCGCGGTG
GTGGTGAACGCCGACAAGGACAAGATTATAACCATGCCCGGAATCGCGATTGTGTGGGGACTTGTGGTCATGGTT
ATGATTTACTCGGTTGGTCATATATCTGGTGCACATTTTAATCCGGCTGTGACCATTGCTTTTGCCTCCTGCAAG
AGGTTCCCATTGAGACAGGTTAGGTCAGCTCAAGTTACATCGATCCTTATGTACCGAATATGCTCTTAATTTCTT
TGTGTTCGGTTGTGACTCCAAACAGGTACCGGCATATATTTCGGCTCAAATGATTGGAGCCACACTAGCAGGCGG
AACCCTACGGTTATTGTTCCAAGGAAAACATGACCACTTCGGTGGAACATTTCCGACCGGCAGCGACATGCAGTC
TTTTGTGGTGGAATTCATAATCACATTCTACCTCATGTTCGTCATATCTGGAGTTGCTACTGACAACCGAGCTGT
AATTAGTCTAATTCCTTCACCAATTATTATTAAATAGTTATATTCAGTACTACTGGTATCAAGCACCAGTTTAAG
TAGCAATTGTATGACATTACATGATAGATTCATTTAAATGTATCGTCACATGATTCATTTAATTGAACTCATCAA
GTGTGGTTATTTAAACTGATGTTATAGCACCACTGATACTAGATAACATTATTCATTATTATTAATCCAAAAATT
TATTTATATTATATATCGTGTTCTTACGACAATTCTTCCAAAAGACAACATAGAGTTCTGTACTTTTGTTAATAA
ATTCAAGAAACGGGATTAATAAGAAATCATGGTGATTTTTTTATAGTTAAAAATGGAGGAAGATAATGACATTTT
TATTAAAGCTTATCCATGTTGACAATGATATGGCCCTTGTCGATAATTTCCTTTTATGATTTTCTTTTATTCGGG
TTGCCATGCAGATTGGAGAACTTGCGGGTTTAGCCATTGGAGCGACTATTTTACTTAATGTGATGATTGCAGGGT
AAGTTCTATGCTCGATTATCTTGCTAATTTTCATAAAGATTGTATTTCAAAATCATATCTTGGGAACATTTTCAT
TTTGTGATCTATTTCAAATTCTCTAAATCTTGCTTCGGTGTCATGATTCTACTTAAATTTCTAATAAGACAAATA
TATTGGATTCAAAACTAAATGATGCGATAGGAATTTCTGAAGAGTAAGATAAGCCGAAGCTCTTCTAAAATAACA
GTACGAATAAGTTAATGACTTGAGTACAGTGTGTTTTGAGTTTGGATAATGTTATTTTGGTGGCGACACTATTGA
TATTAAATTATATGTTTAATTAAATACAATAAATTATGACTCATTTAATAGGATATGCTGACATATAAACTGGTG
TTGAACACATAAATTGGTACTAGATACTATTATTCTTTAAGTTTATATTGAAATTTTATTTCAAAAATATCGATT
ATGGACTTGATACTTGGTAGCTTTAATGGACAGGCCAGTATCTGGGGCTTCAATGAACCCAGCAAGGAGTTTGGG
CCCAGCAATAGTGTCCAATAAATACAAGGGAATATGGATATACATAGTGGGCCCAACTCTTGGGGCTGTAGCAGG
TGCTTGGGTCTACAATATCATCAGATTCACAAACAAGCCTTTAAGAGAAATCACCAAGAGTGCTTCTTTCCTCAA
GAGCTCGGCCCGCAATGGGTCCAAC**TAA**

>*Oleur*NIP1;1

ATGGCTGAAATGTCCGGAGCAAATGGAGTGTCCCTGAGTATAAAGGATGATGTGAACGAGAAGAGCATTCAAGAT
TCTCCAGACTGGTTGCCTTTTATACAGAAGATAATTGCAGAGATTTTGGGTACATACTTCTTGATTTTCGCCGGT
TGTGCCGCGGTGGTGGTGAACGCCGACAAGGACAAGATTATAACCATGCCCGGAATCGCGATTGTGTGGGGACTT
GTGGTCATGGTTATGATTTACTCGGTTGGTCATATATCTGGTGCACATTTTAATCCGGCTGTGACCATTGCTTTT
GCCTCCTGCAAGAGGTTCCCATTGAGACAGGTACCGGCATATATTTCGGCTCAAATGATTGGAGCCACACTAGCA
GGCGGAACCCTACGGTTATTGTTCCAAGGAAAACATGACCACTTCGGTGGAACATTTCCGACCGGCAGCGACATG
CAGTCTTTTGTGGTGGAATTCATAATCACATTCTACCTCATGTTCATTGGAGAACTTGCGGGTTTAGCCATTGGA
GCGACTATTTTACTTAATGTGATGATTGCAGGGCCAGTATCTGGGGCTTCAATGAACCCAGCAAGGAGTTTGGGC
CCAGCAATAGTGTCCAATAAATACAAGGGAATATGGATATACATAGTGGGCCCAACTCTTGGGGCTGTAGCAGGT

GCTTGGGTCTACAATATCATCAGATTCACAAACAAGCCTTTAAGAGAAATCACCAAGAGTGCTTCTTTCCTCAAG
AGCTCGGCCCGCAATGGGTCCAACTAA

>*Oleur*NIP1;1
MAEMSGANGVSLSIKDDVNEKSIQDSPDWLPFIQKIIAEILGTYFLIFAGCAAVVVNADKDKIITMPGIAIVWGL
VVMVMIYSVGHISGAHFNPAVTIAFASCKRFPLRQVPAYISAQMIGATLAGGTLRLLFQGKHDHFGGTFPTGSDM
QSFVVEFIITFYLMFIGELAGLAIGATILLNVMIAGPVSGASMNPARSLGPAIVSNKYKGIWIYIVGPTLGAVAG
AWVYNIIRFTNKPLREITKSASFLKSSARNGSN*

*Oleur*NIP1;2 Oleur061Scf2586g05026.1

>Oleur061Scf2586:547390..550593 (- strand) class=gene length=3204

>*Oleur*NIP1;2
ATGGACGATATGTCTCGAGTCAGTTCCAACCGAGTAGTGTCCATTAACATAGAAGATGATGATTTCAATCAAAAC
CCTCCTCCTCCTACATCCACTAACACCAGGAATTCTTCAGGCTGTTCTATTTCTGTATCTTTCGTGCAAAAGGTG
ATTCTGATTCTCCTACAAAAGTTTTTTTATCTGTTATCTACTATTATTTTGTGCTAATATATATGTTATATGTA
ATTTATTCCATGCTTACAGTTGATCGCAGAGTTTATTGGAACCTACTTCCTAATATTTGGTGGTTGCGCAGCCAT
CACGGTGAACAAAAACATGGAAAATGTAGTGACACTTCCAGGAATAGCCGCCGTGTGCGGACTGGTGGTGGTGGT
CATGATTTACTCCGTTGGACATATTTCTGGAGCACATTTGAATCCCGCTGTAACCATTGCTTTTGCCACCTTCAA
GAGGTTCCCGTGGAAACAGGAAATATACATATATACGTTATACTTGAATCTCCCCTAATAGGTTTTAACAGTTGA
AAACGCCGTTTCTCATCGCTTAAGCTTTAAGATGAGTAGGCTTGCGTTTGATGAAAAATCCAAGCTTCACATGCG
GAGGCTTTGTCCTCTCTATAAAAGCTTAAGCTTCTATATGAGGTGGTTCAACACATCTATATATTGTTTTTGACA
TGATCAAACGTGGGTTTTCTCATCGTGCAGGTACCAGCCTATGTCGTATCTCAAGCTCTTGCTTCAATTGTAGCC
AGGCGCGGAGCCAGCACCCGGCGAGGGGGCGCGGCCGCGCGTGCTGGCTCTGCCGGAGTCTGCAGAGCCGCCGGG
CAGTGTCACCAGTGGTCGCCGACGGTCGCTGGAGAAGAAGAAGATGGAACCCGTTTCAATTTTTTTTTTTTGGTT
TGGGCTTTTTTTACAAAATGTTGGGCCTTTTATTATTTAGCCCAAATTCCAAACCCATTAGCCTGACCCATATTA
ATACCCATTTCAATGATTTCATTGCCCTTTCTTTCAATTTCAGAATCGTGAAGGTGAATTCGTGACTTCGTTCAA
TTCGTTGGCTGCCGGCTGGGACTGGGAGGGATCCTCTTCTAATCTTCTTCACTTTCATTTTGTGGACCGGTTCAG
AAAAATTGCTGCAGTCTGCAGACTGCAGACCGAAACTCGAACTCGATCGAAGACTCGCCTGCTCCAGCCTCCAGG
CCTCCAGTGCTCCTGCAGTCCTACTGCCGCCTGCTGCGGTGCTGCCGAGAGGCCCTAGTCACCCGACTCCGACTG
CCGACTGGTAAGTGTAAAGTGTGGTAACTCTTTTTTTTTTTCCGGCCCTCTTTATTTTTTTTTTTTTTTGTTCTT
TTATTGGTTTTGTTCTATTTTGATTTGGCTATTTTGATTTTGTTGAATTTTGTTCTACTTGTAAATGTCTCAATT
TATTGGAGTTTTGAGCTTTTCAATCATGTGTTCCCTCATACTGTACCTTTAATCAATTGTCAAAACCAATGAGAT
TTAGCACTTAGCAGCAATGATTTTTAATAAGTTAAATTCTCTTTCTTCTCTCATGCGTTTTCACAAATTATACGG
AAATTTATACTTGCTAGTTGCTAAATGTAGTCAAGGATTTTGTTCTATAATTTTGACATCTACAAACCACTGGTC
GTTTATAGAGTTGTAAAGGTAGTGATGTAAGGTTAGAATGACCCAATTTTGTAGTGTAGTCAAGCGTGCATGAAA
TAGTTAAATTTTTCTTCGATTCTTGATCAAAATTTACATATTTTTGCCATTTCTTTCCTCATAAAAATTTTCTGC
AACTATAATATTAAAAAATAACAATTACGAAGGTCAACCAGTCCTAGTCCGATCGTCAACGGTCAACAGTCAACG
GTCAACCCGCCCTAGCTGGACTTCGATCCTGGATTCGCCGCTGATTGTAGCTAGTGGAACCCTTCGACTATTATT
TATGGGGAAACAAGATCACTTTATTGGAACCCTTTCGTCGGGATCGGATGTTCAATCCTTTATGATTGAGTTCAT
TACCACATTTTACCTCATGTTTGTCATTTCTGGTGTAGCCACAGATAATCGAGCTGTTAGTTCATCTTCTTCATC
CATTATATTTTTTATTCATCCATGTTTAGATTTCTGATTGATTTAAAATTCTATTGTTAATTACTAATTGTCTCT
ATCTAATAATAACAACATTCTTTGTCAACGATGCAGATTGGAGAACTCAAAGGCATTGCCATTGGAGCTACTCTT
TCACTTAACATACTTTTCGCCGGGTACGGCTGATTAATTTAGATAATTGTCCCGTAGCTTAATTTCACACCAGAT
TGATTCAAGTTCCGATGAATCAACTACTATGAATATTGAGTACGATGAGTTTTGATTAAATGAAGATGAATTTGA
AAAAAATAAATGTGTCATTTTTTTAATATTGCATGTTTTTAGATGAGATAGTTTAACAAACAATTTCTTGAATAC
TTTCATCACACATTCGAATTTGATACACGATACTCGACATGATTGAAAAATATTAACTCTAAGGCTTGTTCTGAA
ATGACAGGCCAATTTCAGGGGCATCACTGAATCCAGCAAGGAGTTTGGGACCAGCAATTGTTTACAATCGCTACG
AGGGTTTGTGGAACTACATTCTGGCTCCTATTTCTGGAGCCATAGCAGGTGCTTGGGTCTATAATTTTATCAGAT
TTACGGACAAGCCCTTGCGTGAAATTATCAAAAGGGGATCTTCCATTAGAAACGCAGCACGAACCAACTCTACTC
AAGTATCAAGAAACAAAAAGAGGATAAAATTTTGTTTATTATAA
>*Oleur*NIP1;2
ATGGACGATATGTCTCGAGTCAGTTCCAACCGAGTAGTGTCCATTAACATAGAAGATGATGATTTCAATCAAAAC
CCTCCTCCTCCTACATCCACTAACACCAGGAATTCTTCAGGCTGTTCTATTTCTGTATCTTTCGTGCAAAAGTTG
ATCGCAGAGTTTATTGGAACCTACTTCCTAATATTTGGTGGTTGCGCAGCCATCACGGTGAACAAAAACATGGAA
AATGTAGTGACACTTCCAGGAATAGCCGCCGTGTGCGGACTGGTGGTGGTGGTCATGATTTACTCCGTTGGACAT
ATTTCTGGAGCACATTTGAATCCCGCTGTAACCATTGCTTTTGCCACCTTCAAGAGGTTCCCGTGGAAACAGGTA
CCAGCCTATGTCGTATCTCAAGCTCTTGCTTCAATTGTAGCCAGTGGAACCCTTCGACTATTATTTATGGGGAAA
CAAGATCACTTTATTGGAACCCTTTCGTCGGGATCGGATGTTCAATCCTTTATGATTGAGTTCATTACCACATTT
TACCTCATGTTTGTCATTTCTGGTGTAGCCACAGATAATCGAGCTATTGGAGAACTCAAAGGCATTGCCATTGGA

GCTACTCTTTCACTTAACATACTTTTCGCCGGGCCAATTTCAGGGGCATCACTGAATCCAGCAAGGAGTTTGGGA
CCAGCAATTGTTTACAATCGCTACGAGGGTTTGTGGAACTACATTCTGGCTCCTATTTCTGGAGCCATAGCAGGT
GCTTGGGTCTATAATTTTATCAGATTTACGGACAAGCCCTTGCGTGAAATTATCAAAAGGGGATCTTCCATTAGA
AACGCAGCACGAACCAACTCTACTCAAGTATCAAGAAACAAAAAGAGGATAAAATTTTGTTTATTA**TAA**

>*Oleur*NIP1;2
MDDMSRVSSNRVVSINIEDDDFNQNPPPPTSTNTRNSSGCSISVSFVQKLIAEFIGTYFLIFGGCAAITVNKNME
NVVTLPGIAAVCGLVVVVMIYSVGHISGAHLNPAVTIAFATFKRFPWKQVPAYVVSQALASIVASGTLRLLFMGK
QDHFIGTLSSGSDVQSFMIEFITTFYLMFVISGVATDNRAIGELKGIAIGATLSLNILFAGPISGASLNPARSLG
PAIVYNRYEGLWNYILAPISGAIAGAWVYNFIRFTDKPLREIIKRGSSIRNAARTNSTQVSRNKKRIKFCLL*

***Oleur*NIP1;3 Oleur061Scf0038g03004.1**

>Oleur061Scf0038:312314..314472 (- strand) class=gene length=2159

>*Oleur*NIP1;3
ATGGCCGAAATGTCAGGAGCAAATGGATTGGTGGATGAGAAGCATATTCAAAATTCTCCAAACTGGTTACCCTTT
ATACAGAAGGTATGTACATACGTACGTTGTGTTTTTTTTCTTTTTTTAAATTTTAAAATTTTTTTTTTATCTTG
AAGACGATGAAGAAATCGATGCCTTCTTAATAAATGCTAGCTAGTAGGTTGTATAACTAACTGCTACTCGTAATT
AATTATAACAATCTGATAGCTGATTATCTCAAAATATATGTAGGTAATTGCAGAGGGTTTTGGTACCTACTTCTT
GATTTTTGCCGGTTGTGCCGCAGTGGTGGTGAACGCCGATAAGGAGGAGGTGATAACCCAGCCGGGAATCGCAAT
AGTGTGGGGACTTGTCGTCATGGCTATGGTTTACTCAGTTGGCCATATTTCCGGTGCTCATTTTAATCCGGCTGT
GACCATTGCTTTTGCCACCTGCAAGAGGTTCCCACTTAGACAGGTGAAGATGGTATATTCAACAGAATTATATGT
ACTGAACGAACGTGCTCTTGATTTCTTCGTGCTCATCCTGATAGGTACCGGCATATATTGCGGCACAAGTGATCG
GAGCCACGCTAGCAAGCGGTACCCTGAGATTACTGTTCATGGGAAAACACGATCACTTTGCCGGAACACTTCCGA
CCGGCTCCGACTTGCAGTCTTTTGTTATGGAATTTATAATCACGTTCTACCTAATGTTTGTCATATCTGGAGTGG
CTACAGACAACCGTGCTGTAATTCCTTCACCTTATTCTTGGTCTTTCGAAAGACAACACAGAGTTATCTACTTCA
TGTTTATAAATTCAAGAAAACCGGATTAGCAAGAGATCATGGTGGTGATTTCATAATTTGAAAGGGTTTAAGATA
AATGACATCTTTTTGACAATGATACGGACCTTTTTTATCAATCATTGTATGACCCTTTTTTTTGTTCGGGCTGCC
ATGCAGATTGGAGAACTTGCCGGTTTAGCCATTGGAGCAACTATTTTACTTAACGTGATGTTTGCAGGGTATGTA
CTATGCTCAATTGTCTTGCTATTCCTCTATATAAAGTTGCAGAACTGATCTTGTGCCCATGTCGCTGGTTGAAGA
TCAGCGGTGGATAATATATCTTATGTCCACTATGGGTAGGAGATCAGTTCTGTATAAAGATATTGTATTTCAAGA
AAGTGATTTCAAAAGTGTCAGACCAAGCCATCAAGAAAAATAATAGACTTGTTCGGATAGTCATAATCATAATCT
TGGGAAGTTTTTTAAATTTGCAATCTGTTTCGAATTTTCAAAAGTTTGTTTTTTTTATGTTATAATTCCTCAATG
TGAGGCTAACGGGTAGTTTTGATGGACAGGCCAATATCTGGGGCTTCAATGAACCCAGTAAGAAGTTTAGGCCCA
GCAATAGTGTCAAATCGGTACGAGGGAATATGGATATACATAGTAGGGCCGACTCTTGGGGCTATAGCGGGTGCT
TGGGCCTATAATATCATCAGATTCACAGACAAACCTTTACGAGAAATCACTAAGACTGCTTCTTTCCTCAAGATC
TAG

>*Oleur*NIP1;3
ATGGCCGAAATGTCAGGAGCAAATGGATTGGTGGATGAGAAGCATATTCAAAATTCTCCAAACTGGTTACCCTTT
ATACAGAAGGTAATTGCAGAGGGTTTTGGTACCTACTTCTTGATTTTTGCCGGTTGTGCCGCAGTGGTGGTGAAC
GCCGATAAGGAGGAGGTGATAACCCAGCCGGGAATCGCAATAGTGTGGGGACTTGTCGTCATGGCTATGGTTTAC
TCAGTTGGCCATATTTCCGGTGCTCATTTTAATCCGGCTGTGACCATTGCTTTTGCCACCTGCAAGAGGTTCCCA
CTTAGACAGGTACCGGCATATATTGCGGCACAAGTGATCGGAGCCACGCTAGCAAGCGGTACCCTGAGATTACTG
TTCATGGGAAAACACGATCACTTTGCCGGAACACTTCCGACCGGCTCCGACTTGCAGTCTTTTGTTATGGAATTT
ATAATCACGTTCTACCTAATGTTTGTCATATCTGGAGTGGCTACAGACAACCGTGCTATTGGAGAACTTGCCGGT
TTAGCCATTGGAGCAACTATTTTACTTAACGTGATGTTTGCAGGGCCAATATCTGGGGCTTCAATGAACCCAGTA
AGAAGTTTAGGCCCAGCAATAGTGTCAAATCGGTACGAGGGAATATGGATATACATAGTAGGGCCGACTCTTGGG
GCTATAGCGGGTGCTTGGGCCTATAATATCATCAGATTCACAGACAAACCTTTACGAGAAATCACTAAGACTGCT
TCTTTCCTCAAGATC**TAG**

>*Oleur*NIP1;3
MAEMSGANGLVDEKHIQNSPNWLPFIQKVIAEGFGTYFLIFAGCAAVVVNADKEEVITQPGIAIVWGLVVMAMVY
SVGHISGAHFNPAVTIAFATCKRFPLRQVPAYIAAQVIGATLASGTLRLLFMGKHDHFAGTLPTGSDLQSFVMEF
IITFYLMFVISGVATDNRAIGELAGLAIGATILLNVMFAGPISGASMNPVRSLGPAIVSNRYEGIWIYIVGPTLG
AIAGAWAYNIIRFTDKPLREITKTASFLKI*

***Oleur*NIP1;4 Oleur061Scf7194g00006.1**

>Oleur061Scf7194:25229..27368 (- strand) class=gene length=2140

>*Oleur*NIP1;4

ATGGCCGAAATGTCAGGAGCAAATGGATTGGTGGATGAGAAGCATATTCAAAATTCTCCAAACTGGTTACCCTTT
ATACAGAAGGTATGTACATACGTACGTTGTGTTTTTTTTTCTTTTTTTAAATTTTTAAATTTTTTTTTTTTTAATC
TTGAAGACGATGAAGAAATCGATGCCTTCTTAATAAATGCTAGCTAGTAAGTTGTATAACTAACTGCTACTCGTA
ATTAATTATAACAAAATATATGTAGGTAATTGCAGAGGGTTTTGGTACCTACTTCTTGATTTTTGCCGGTTGTGC
CGCAGTGGTGGTGAACGCCGATAAGGAGGAGGTGATAACCCAGCCGGGAATCGCAATAGTGTGGGGACTTGTCGT
CATGGCTATGGTTTACTCAGTTGGCCATATTTCCGGTGCTCATTTTAATCCGGCTGTGACCATTGCTTTTGCCAC
CTGCAAGAGGTTCCCACTTAGACAGGTGAAGATGGTATATTCAACAGAATTATATGTACTGAACGAACGTGCTCT
TGATTTCTTCGTGCTCATCCTGATAGGTACCGGCATATATTGCGGCACAAGTGATCGGAGCCACGCTAGCAAGCG
GTACCCTGAGATTACTGTTCATGGGAAAACACGATCACTTTGCCGGAACACTTCCGACCGGCTCCGACTTGCAGT
CTTTTGTTATGGAATTTATAATCACGTTCTACCTAATGTTTGTCATATCTGGAGTAGCTACAGACAACCGTGCTG
TAATTCCTTCACCTTATTCTTGGTCTTTCGAAAGACAACATAGAGTTATCTACTTCATGTTTATAAATTCAAGAA
AACCGGATTAGCAAGAGATCATGGTGGTGATTTCATAATTTGAAAGGGTTTAAGATAAATGACATCTTTTTGACA
ATGATACGGACCTTTTTTATCAATCATTGTATGACCCTTTTTTTTGTTCGGGCTGCCATGCAGATTGGAGAACTT
GCCGGTTTAGCCATTGGAGCAACTATTTTACTTAACGTGATGTTTGCAGGGTATGTACTATGCTCAATTGTCTTG
CTATTCCTCTATATAAAGTTGCAGAACTGATCTTGTGAACATGTCGCTGGTTGAAGATCAGCGGTAGATAATATA
TCTTATGTCCACTATAGGTAGGAGATCAGTTCTGTATAAAGATATTGTATTTCAAGAAAGTGATTTCAAAAATGT
CAGACCAAGCCATCAAGAAAAATAATAGACTTGTTCGGATAGTCATAATCATAATCTTGGGAAGTTTTTTAAATT
TGCAATCTGTTTCGAATTTTCAAAAGTTTGTTTTTTTATGTTATAATTCCTCAATGTGAGGCTAACGGGTAGTTT
TGATGGACAGGCCAATATCTGGGGCTTCAATGAACCCAGTAAGAAGTTTAGGCCCAGCAATAGTGTCAAATCGGT
ACGAGGGAATATGGATATACATAGTAGGCCCGACTCTTGGGGCTATAGCGGGTGCTTGGGCCTATAATATCATCA
GATTCACAGACAAACCTTTACGAGAAATCACTAAGACTGCTTCTTTCCTCAAGATC**TAG**

>*Oleur*NIP1;4

ATGGCCGAAATGTCAGGAGCAAATGGATTGGTGGATGAGAAGCATATTCAAAATTCTCCAAACTGGTTACCCTTT
ATACAGAAGGTAATTGCAGAGGGTTTTGGTACCTACTTCTTGATTTTTGCCGGTTGTGCCGCAGTGGTGGTGAAC
GCCGATAAGGAGGAGGTGATAACCCAGCCGGGAATCGCAATAGTGTGGGGACTTGTCGTCATGGCTATGGTTTAC
TCAGTTGGCCATATTTCCGGTGCTCATTTTAATCCGGCTGTGACCATTGCTTTTGCCACCTGCAAGAGGTTCCCA
CTTAGACAGGTGAAGATGGTACCGGCATATATTGCGGCACAAGTGATCGGAGCCACGCTAGCAAGCGGTACCCTG
AGATTACTGTTCATGGGAAAACACGATCACTTTGCCGGAACACTTCCGACCGGCTCCGACTTGCAGTCTTTTGTT
ATGGAATTTATAATCACGTTCTACCTAATGTTTGTCATATCTGGAGTAGCTACAGACAACCGTGCTATTGGAGAA
CTTGCCGGTTTAGCCATTGGAGCAACTATTTTACTTAACGTGATGTTTGCAGGGCCAATATCTGGGGCTTCAATG
AACCCAGTAAGAAGTTTAGGCCCAGCAATAGTGTCAAATCGGTACGAGGGAATATGGATATACATAGTAGGCCCG
ACTCTTGGGGCTATAGCGGGTGCTTGGGCCTATAATATCATCAGATTCACAGACAAACCTTTACGAGAAATCACT
AAGACTGCTTCTTTCCTCAAGATC**TAG**

>*Oleur*NIP1;4

MAEMSGANGLVDEKHIQNSPNWLPFIQKVIAEGFGTYFLIFAGCAAVVVNADKEEVITQPGIAIVWGLVVMAMVY
SVGHISGAHFNPAVTIAFATCKRFPLRQVKMVPAYIAAQVIGATLASGTLRLLFMGKHDHFAGTLPTGSDLQSFV
MEFIITFYLMFVISGVATDNRAIGELAGLAIGATILLNVMFAGPISGASMNPVRSLGPAIVSNRYEGIWIYIVGP
TLGAIAGAWAYNIIRFTDKPLREITKTASFLKI*

***Oleur*NIP3;1 Oleur061Scf1427g05001.1**

>Oleur061Scf1427:489963..491438 (+ strand) class=gene length=1476

>*Oleur*NIP3;1

ATGGCAAATTCACCTTCTATTATTAGCTTTTCCTCTAAACAAAATTTACCAACAGACTTGTCCATTACAGAGGAA
GCAAAGGGTTACTCCGCCGACGAATTCCCTCCAAGAGATTCTGTTTCCCCGTCGTACTACCAGAAAGTAAGTTTT
TAAACGATTGCATTTGACAATACAGTATACAAGATTTTAATGGATCATTTGTGCTTGAGTTAACAGATTATAGCA
GAGATTTTGGGCACTTATGTTCTTGTTTTTGCGGGTTGCGGTGCTGCTCTAGTTGATCGAGAGAAGACATTAACA
GTTGTGGGTTTAGCAATGGTGTGGGGGCTGGCTTTGATGGCAATGATATATGCACTTGGCCATGTTTCTGGTGGG
CATTTCAACCCTGCAGTTACCATTGGCTTTGCAGCAGCTCGAAAAATTCCACTCGTTCACGTAAGAATATCATTT
GTACACGAAATTTGGATTGTTCAGTAGTATCCAATCATCTGTTTTGTAACATTGGAATCGTCTTGCAGGTCCCGT
TGTACGTTTTATCGCAGTTTTTGGGTTCAACGCTAGCATGCCTCACCCTGAAAGTACTATTCAATGACCAAGCTG
ATATACTACCCACTCTCACTCATTATTCCAGTTCAACTACTGATCTTGAAGCGATTACATGGGAATTCGTCATCA
CTTTCATTTTTATGCTCACCATACGTGGAGTAGCTTCTGATGACAGAGCAGTATGTATTTCTCGTTGTGATACCT
CTATATTTAGTTCTTGCATGGTTTTATCTAAGCGTTATTGATACTTTTCAACCATCTTCTTTCATAATCTATAAC
AGATCATTCAATTACTGATTTTGCAATTTTTGTTGATGAAATCAGAATAATGAACTGGCTGGAGTTGCAATTGGA
GCTACAGTGATATTTAATGCCCTTGTTGCAGGGTAAGACTTCCCATCCCACAAGCTTCATGCATGTTTTGCTAAG
CTTAAAAGGTTAATAAATATATAAATTTTAAAATAAATAATTTTTTTGTTGTTCATAAGGGAGATGAAAGTAAGA
AAAAAGTAACATATTTTATCTTATTTGCATAAGCAGTAAACACAAAATGATCCCACCATTCTGCACTATTCTTCT
CTTAATATGAACTTCTAGTAACGCTTTCCATCATGTAATCGAAATTGCAACACAAGAAAATAGAAAAGATAAGGG
AACCAGAATAAAGGGAGTTCTTAAATATACTTATATAGGTGAATTGAAAAAAAATGCAGGCCTATTACTGGAGCT
TCTATGAATCCTGCAAGAAGCATGGGTCCAGCAATAGTTTCTGGAGTCTATAAAAATCAATGGGTGTATGTTGTT

GCTCCCATTCTTGGAGCCATAGCTGCAAGCCTGATATACAGTCTTCTCCAGCCATGGAAGCCGGAAAAGCATAGA
GAGAGTGCTAGAAGTACATACAATGATCTATATCTACGTCCCGAGGTATGA

>*Oleur*NIP3;1
ATGGCAAATTCACCTTCTATTATTAGCTTTTCCTCTAAACAAAATTTACCAACAGACTTGTCCATTACAGAGGAA
GCAAAGGGTTACTCCGCCGACGAATTCCCTCCAAGAGATTCTGTTTCCCCGTCGTACTACCAGAAAATTATAGCA
GAGATTTTGGGCACTTATGTTCTTGTTTTTGCGGGTTGCGGTGCTGCTCTAGTTGATCGAGAGAAGACATTAACA
GTTGTGGGTTTAGCAATGGTGTGGGGGCTGGCTTTGATGGCAATGATATATGCACTTGGCCATGTTTCTGGTGGG
CATTTCAACCCTGCAGTTACCATTGGCTTTGCAGCAGCTCGAAAAATTCCACTCGTTCACGTCCCGTTGTACGTT
TTATCGCAGTTTTTGGGTTCAACGCTAGCATGCCTCACCCTGAAAGTACTATTCAATGACCAAGCTGATATACTA
CCCACTCTCACTCATTATTCCAGTTCAACTACTGATCTTGAAGCGATTACATGGGAATTCGTCATCACTTTCATT
TTTATGCTCACCATACGTGGAGTAGCTTCTGATGACAGAGCATTGCAATTGGAGCTACAGCCTATTACTGGAGCT
TCTATGAATCCTGCAAGAAGCATGGGTCCAGCAATAGTTTCTGGAGTCTATAAAAATCAATGGGTGTATGTTGTT
GCTCCCATTCTTGGAGCCATAGCTGCAAGCCTGATATACAGTCTTCTCCAGCCATGGAAGCCGGAAAAGCATAGA
GAGAGTGCTAGAAGTACATACAATGATCTATATCTACGTCCCGAGGTATGA
>*Oleur*NIP3;1
MANSPSIISFSSKQNLPTDLSITEEAKGYSADEFPPRDSVSPSYYQKIIAEILGTYVLVFAGCGAALVDREKTLT
VVGLAMVWGLALMAMIYALGHVSGGHFNPAVTIGFAAARKIPLVHVPLYVLSQFLGSTLACLTLKVLFNDQADIL
PTLTHYSSSTTDLEAITWEFVITFIFMLTIRGVASDDRALQLELQPITGASMNPARSMGPAIVSGVYKNQWVYVV
APILGAIAASLIYSLLQPWKPEKHRESARSTYNDLYLRPEV*

***Oleur*NIP4;1a Oleur061Scf7805g00013.1**

>Oleur061Scf7805:30162..32022 (+ strand) class=gene length=1861

>*Oleur*NIP4;1a
ATGGCAATCGAAGAAATGGAAGAAGGAGTAGAAACCTCAACGTTCAATGCTAAAGAAGGCTTCTGTATGTCGGCT
TCAATCGTAACTATCGTACAAAAGGTTAGATCTCGTAACATTTCACTTGAAAACTTTTGGTACGATTCTCTACAA
TCTTCGATTTTTCATATTTATGCATAACATGTCACTTTCAATCTTGTTCGGTCGAGCATGCAAAAAAAATGTACTA
AACGTAATGTGTTAGATGTTTCCCAGGTGATTGCAGAGGTGATTGGCACGTATTTTGTTATATTTTCGGGGTGTG
GCTCTGTTGCGGTGAATAAATTGTATGGTGGGACGGTCACATTCCCAGGCATTTGTGCGACATGGGGACTAATTG
TGATGGTCATGGTCTACTCCGTAGGTCACATATCCGGGGCTCATTTTAACCCTGCGGTCACCATTACCTTCGCCA
TCTTTCGCCGTTTTCCATGGAAAGAGGTACCGATTAATACAACAGACATGTTTAATATGTTAGATCGATTTCATG
TACATACATACACGTATGGGCAATTATGATGGTTTAAGTAAAACGGGATGAGAATTGATAAGTTTGAAATGTGAT
AATATCGTGCAAGAAATTCGTGGGTTTTTGTTATCATTTATTATGTTCCATATTGCATATTAAACAAAAAGATGT
ATTGTTTTGTATTGATAATTTAAATCATAGTTATTAAACACGGATTGAACTGATTTTCTTTTAAACTGTGCAACT
CGAATCATCGTAGATACAGTAATGTTCCAAGATTTGCAGGTTCCATTGTATATAATAGCGCAATTGATTGGATCG
ATCCTTGCGAGTTGTACTTTGGCTATCTTGTTTGACATCACCTCTGAAGCTTACTTTGGCACCTTGCCCGTTGGA
TCAAATGGGCAATCTTTCGCCATAGAAATCATCATCTCCTTCCTCTTGATGTTCACCATTTCTGGCGTAGCAACA
GATAGTAGAGCGGTATGTAACATCAAAAAACATACATGACGTATTGGTAAAATGACTTAATTTTATTATTTAGAT
GCTCTAAATTTCATACTTCTGAACGAGCTGCCCGCGTAATTTTCTGCTTGCTTAACCTAATTTGTTTATTTATTT
TCCTTTTGAAACATAGATTGGAGATCTTGCCGGTTTAGTCGTTGGGATGACTATAATGTTAAATGTGTTCGTTGC
CGGGTACGTTAATGTCTCAATCCTGTGTTTTGTTACGGGATTGGACAATATAAAAAATAATAATTATAATAATAA
AATCAACAATATTTTATTTTTAATTTTCTATTCGATCTGCAGGCCGATTTCTGGAGCCTCAATGAACCCAGCAAG
GAGCATAGGCCCAGCCATTGTGAAGCATACTTATAAAGGACTCTGGGTTTATATATTTGGCCCACTTATTGGGAC
CATACTTGGAGGATTTGCTTATAATTTGATTAGATATACGAATAAACCCCTTCGTGAATTAACTAAAAGTAGTTC
ATTAATCAGAAGCATATCCAAAAATAATACAACTTAG
>*Oleur*NIP4;1a
ATGGCAATCGAAGAAATGGAAGAAGGAGTAGAAACCTCAACGTTCAATGCTAAAGAAGGCTTCTGTATGTCGGCT
TCAATCGTAACTATCGTACAAAAGGTGATTGCAGAGGTGATTGGCACGTATTTTGTTATATTTTCGGGGTGTGGC
TCTGTTGCGGTGAATAAATTGTATGGTGGGACGGTCACATTCCCAGGCATTTGTGCGACATGGGGACTAATTGTG
ATGGTCATGGTCTACTCCGTAGGTCACATATCCGGGGCTCATTTTAACCCTGCGGTCACCATTACCTTCGCCATC
TTTCGCCGTTTTCCATGGAAAGAGGTTCCATTGTATATAATAGCGCAATTGATTGGATCGATCCTTGCGAGTTGT
ACTTTGGCTATCTTGTTTGACATCACCTCTGAAGCTTACTTTGGCACCTTGCCCGTTGGATCAAATGGGCAATCT
TTCGCCATAGAAATCATCATCTCCTTCCTCTTGATGTTCACCATTTCTGGCGTAGCAACAGATAGTAGAGCGATT
GGAGATCTTGCCGGTTTAGTCGTTGGGATGACTATAATGTTAAATGTGTTCGTTGCCGGGCCGATTTCTGGAGCC
TCAATGAACCCAGCAAGGAGCATAGGCCCAGCCATTGTGAAGCATACTTATAAAGGACTCTGGGTTTATATATTT
GGCCCACTTATTGGGACCATACTTGGAGGATTTGCTTATAATTTGATTAGATATACGAATAAACCCCTTCGTGAA
TTAACTAAAAGTAGTTCATTAATCAGAAGCATATCCAAAAATAATACAACTTAG
>*Oleur*NIP4;1a
MAIEEMEEGVETSTFNAKEGFCMSASIVTIVQKVIAEVIGTYFVIFSGCGSVAVNKLYGGTVTFPGICATWGLIV
MVMVYSVGHISGAHFNPAVTITFAIFRRFPWKEVPLYIIAQLIGSILASCTLAILFDITSEAYFGTLPVGSNGQS

FAIEIIISFLLMFTISGVATDSRAIGDLAGLVVGMTIMLNVFVAGPISGASMNPARSIGPAIVKHTYKGLWVYIF
GPLIGTILGGFAYNLIRYTNKPLRELTKSSSLIRSISKNNTT*

*Oleur*NIP4;1b Oleur061Scf3669g01008.1

>Oleur061Scf3669:165510..167403 (+ strand) class=gene length=1894

>*Oleur*NIP4;1b
ATGGCAATCGAAGAAATGGAAGAAGGAGTAGACAACTCAACGTTCAATGCTAAAGAAGGCTTCTGTATGTCGGCT
TCAATCGTAACTATCGTACAAAAGGTTAGATCTCGTAACATTTCACTTGAAAACTTTTGGTACGATTCTCTACAA
TCTTCGATTTTTCATATTAATGCATAACATGTCACTTTCAATCTTGTTCAGTCGAGCATGCAAAAAAATGTACTA
AACGTAATGTGTTAGATGTTTCCCAGGTGATTGCAGAGGTGATTGGCACGTATTTTGTTATATTTTCGGGCTGTG
GCTCTGTTGCGGTGAATAAATTGTATGGTGGGACGGTCACATTCCCAGGCATTTGTGCGACATGGGGACTAATTG
TGATGGTCATGGTCTACTCTGTAGGTCACATATCCGGGGCTCATTTTAACCCTGCGGTCACCATTACCTTCGCCA
TTTTTCGTCGTTTTCCATGGAAAGAGGTACCAATTAATACTACAGACATGTTTAATATGTTAGACCGATTTCATA
TATATATGGACAGGCATGAACAATTATGATAGTATAAATAAAACGGGATGAGAATTGACAAGTTTGAAATGTGAT
CATATCGTGTAAGACATTCGTGGGTTTTCGTCATCATTCATTATGTTCCATATGTTGAATTTGCACTAAATATTT
TATTGGATTCATGTTGCATATTAAAAAAAAAAGAAGCATTGTTGTCTATTGATAATTTAAATCATAGTTATTAAA
CTCGGATTGAATTGATTTTCTTTTTCACTATGCAATTCGAATCATCGTAGATACAGTAATGTTCCAAGATTTGCA
GGTTCCATTGTATATAATAGCGCAATTAATTGGATCGATCCTTGCGAGTTGTACTTTGGCTATCTTGTTTGACAT
CACCTCTGAAGCTTACTTTGGCACCTTGCCCGTTGGATCAAATGGGCAATCTTTCGCCATAGAAATCATCATCTC
CTTCCTCTTGATGTTCACCATTTCTGGCGTAGCAACAGATAGTAGAGCGGTATGTAACATCAAAAAACATACATG
ACATATTGGTAAAATGACTTAATTTTATTATTTAGATGCTCTAAATTTCATACTTCTGAACGAGCTGCCCGCGTA
ATTTTCTGCTTGCTTAACCTAATTTGTTTATTTATTTTCCTTTTGAAACATAGATTGGAGATCTTGCCGGTTTAG
TCGTTGGGATGACTATAATGTTAAATGTGTTCGTTGCCGGGTACGTTAATGTCTCAATACTGTGTTTTGTTACGA
GATTGGACAATATAAAAAATAATAATTATAATAATAAAATCAACAATATTTTATTTTTAATTTTCTATTCAATCT
GCAGGCCGATTTCTGGAGCCTCAATGAACCCAGCAAGGAGCATAGGCCCAGCCATTGTGAAGCATACTTATAAAG
GACTCTGGGTTTATATATTTGGCCCACTTATTGGGACCATACTTGGAGGATTTGCTTATAATTTGATTAGATTTA
CGAATAAACCCCTTCGTGAATTAACTAAAAGTAGTTCATTAATCAGAAGCATATCCAAAAATAATACAACTTAG
>*Oleur*NIP4;1b
ATGGCAATCGAAGAAATGGAAGAAGGAGTAGACAACTCAACGTTCAATGCTAAAGAAGGCTTCTGTATGTCGGCT
TCAATCGTAACTATCGTACAAAAGGTGATTGCAGAGGTGATTGGCACGTATTTTGTTATATTTTCGGGCTGTGGC
TCTGTTGCGGTGAATAAATTGTATGGTGGGACGGTCACATTCCCAGGCATTTGTGCGACATGGGGACTAATTGTG
ATGGTCATGGTCTACTCTGTAGGTCACATATCCGGGGCTCATTTTAACCCTGCGGTCACCATTACCTTCGCCATT
TTTCGTCGTTTTCCATGGAAAGAGGTTCCATTGTATATAATAGCGCAATTAATTGGATCGATCCTTGCGAGTTGT
ACTTTGGCTATCTTGTTTGACATCACCTCTGAAGCTTACTTTGGCACCTTGCCCGTTGGATCAAATGGGCAATCT
TTCGCCATAGAAATCATCATCTCCTTCCTCTTGATGTTCACCATTTCTGGCGTAGCAACAGATAGTAGAGCGATT
GGAGATCTTGCCGGTTTAGTCGTTGGGATGACTATAATGTTAAATGTGTTCGTTGCCGGGCCGATTTCTGGAGCC
TCAATGAACCCAGCAAGGAGCATAGGCCCAGCCATTGTGAAGCATACTTATAAAGGACTCTGGGTTTATATATTT
GGCCCACTTATTGGGACCATACTTGGAGGATTTGCTTATAATTTGATTAGATTTACGAATAAACCCCTTCGTGAA
TTAACTAAAAGTAGTTCATTAATCAGAAGCATATCCAAAAATAATACAACTTAG
>*Oleur*NIP4;1b
MAIEEMEEGVDNSTFNAKEGFCMSASIVTIVQKVIAEVIGTYFVIFSGCGSVAVNKLYGGTVTFPGICATWGLIV
MVMVYSVGHISGAHFNPAVTITFAIFRRFPWKEVPLYIIAQLIGSILASCTLAILFDITSEAYFGTLPVGSNGQS
FAIEIIISFLLMFTISGVATDSRAIGDLAGLVVGMTIMLNVFVAGPISGASMNPARSIGPAIVKHTYKGLWVYIF
GPLIGTILGGFAYNLIRFTNKPLRELTKSSSLIRSISKNNTT*

*Oleur*NIP5;1a Oleur061Scf0098g04035.1

>Oleur061Scf0098:449548..462237 (- strand) class=gene length=1803

>*Oleur*NIP5;1a
ATGCCGGAATTCGAAGTCGAAACTCTGACAATGTCGGCGCCAGCGACGCCTGGAACGCCGGCGCCACTGATTTCT
TCTCTGCGAATAGACTCAGTTTCTTACGATCGGAAGTCAATGCCACGATGCAAGTGCCTGCCAGTTGATGCACCC
ACTTGGGGTGCACCACATACGTGCAGCCTCACCGATTTCTCTGTGCCTGACATCTCTCTTACTCGCAAGGTTAGA
CTTTACACTCTCTCTCTCTCTCTCTCTCTCTCTGTCTCTCACACACAATATAATATGAATATATATTTTGTTTTT
TTAAATTTATTATGTGGAGTGAGTGGGGTCCCTAACCTTTCAGTTGCCTTTCAAATTTTCTGTATTGAACAATGA
TGAGTCGGTTGAATGAATTTATATTGAGTTTTGATACTCAAACAGGAGCTGTCACGGCCCTGTCACACTAGCATA
ACTCTTTATAATCACGGAAATTATTGTCGTTTCTATATCATCGTGACATTCTATCATTTCTTTTACAAATTTTAA
CACTCTTGTGACGTGGTGTAGTGATAAACTACATAGCATTTATGTGAAACATATGTTGTAGTATGACTATCAATT
AATTAATTAATTTTTTTTTTTTTTTTTTTTTGTCAAAATGTTGTGAAACCTGAAACCAAATTGAATTCAATGATT
CAATTGAAACAAAATTGAAGTTAACTAACCCAATTGAACAATAATTGAAATTTGATTGCCCAATTGAGAATCTGA

GATGATGTGAAAGTTTAATCACAAATAATACGAAGTCATGGTCATACAAGACAAAAAAATGTGTGCTACAATTTC
ATATGTAGTCGCATTGCCCACATTTTATAGGTCATAAACTAATTATTAGTCACAAATCATCCAATAACTGGCTAC
AAAACATTCAATTAATTGTTTACAAATACTATTCACATCATCCCACTCCACATCATCCTATCCCATCGCTTCTCT
CTAATTTAGGGATATCAACAAGTCGGGTCGGATTAGGACAACTATTCTCTTCTCTCGTCTTGTTGAGTAGTTTTA
TTACCTTACCCCATCTCCATACACAGTTAAGGCGAGGTTGGACAAGCTGGCTCCTTGTCCTATTTATATTTTATT
TTTTTCATATCTTTAATTGGTAAATATTATTATCATTATTTATTTTTTTCAATTTGTTAATAACACCCATGTTTC
CTCTAAAAACGATGATGATATTTTTCTTTTTACATGATAAAAGTTTAAATTTGAAATGAATTTTAGACAAGCAGG
ATTTATGTTCCCCAAACCAACTCGGGGCAATGACCCTCACTTGTCCCCCACCTCATGTACTAAGCCCCGTTTGAT
TATCGTTCTTTGGTCACAAAAATAAAAAATATAATCGCTATTTATTGTATTAAGTTACTATATTTGACTTTTTAT
CTTTTGGATAGTTTTCATTATCATTCTTACAAAAATAATACCAAATGGGACCTAAGTAGGGAACCTCCATGCATT
TGGGGCGAGGTCCAAGCGTATAAGCTTTTTTGCCATCCTTAATCTCATTCTTCACCCGCTTTCTCCTCGGAACTA
CCACCGGCAGCCACTGAGTTTTTTTCTCACCACGGTCGACAATCACAGAAAAAATAAGTCTACAATTACCGGCAA
CAACCATAGAAAAACAGATATCCAGTCAATCACAACATCCACCTCATAGTGACGACGCTACCACAACTATAGAAA
GACAGATCTACAGCCACATCACAACACCCACCTCATCGCGGTGGCGCCACCAACATCATCCCGCCATTTCACCCT
TTCCACAACGTCTCTTCCATTTTTTTTTTTCGCATCTGCATTCTACACAACTGACGGCACAGACACAAAATCACC
ACCAACCACGTTGATAGAGTAGGAACCTGCACGGGCGCTGTCGATCTAAAAGTCAACACCACGGAAGACGGAGCG
GCAGAAATGAGTAAATATGTGAAATTTTAGGATTAGAGTTTAGTTATTTGGGATAAGAGGAGTAAATCTGTGACG
AATTTAGGGGGCAAAATGGGAAACTAAAGAGTTTAGAGAAGATAGTGGTGGCACTTGAAGATGGCCATAGCTTTT
TCCACAATTAGCCCTTGTTTGAGCCATATTATTTTCTTTAGAATGCATTTTTGAATAATGTATTGATGAAAAATG
TGTGGTTAAAAAATAAAATAAAGTTTTGAATTTGCATGTAATATTATTTTATTGAAAAATGTGCGATTGAAATGA
TATTAAATCACTATTTAAGAATATATATTTTTTTTTTAAATGTGGCTCAAACATACCTTGACACCGTCTGACATAG
AAATCCGTTATAAATGTGAGTAGGTGAGGGCAATATGTATTTGTTAATGGAGGAGAATATTGGAGTTAAAAAATC
AAATTGTGCAGTTTTGAAGTTCTATAATGATTTATGCAAGGATTAAGATGTTTTTATCCGAGGGGGTAATCGAAC
TGTGTCCTCCCTTCACAAACATGCAGGCCCCGCCCCTTATTAAATAATTCAAGCCGTGGTTCCCACCTCATGTTG
TGTGTAGTAGGTAAAAGGTTATATAGATGATTATGTACGTGTAGTATGACCCTTTATTTAGGATTAGAATTTAGT
TATTTGGGCTAAGAGGAGTAAATCGTGAGCTCACGAGCCACGAGCTGCTCGCGAACTCGTCACTCCACATCATCC
TATCCCATCGCTTCTCTCTAATTTAGGGATATCAACAAGTCGGGTCGGATTAGGACAACTATTCTCTTCTCTCGT
CTTGTTGAGTAGTTTTATTACCTTACCCCATCTCCATACACAGTTAAGGCGAGGTTGGACAAGCTGGCTCCTTGT
CCTATTTATATTTTATTTTTTTCATATCTTTAATTGGTAAATATTATTATCATTATTTATTTTTTTCAATTTGTT
AATAACACCCATGTTTCCTCTAAAAACGATGATGATATTTTTCTTTTTACATGATAAAAGTTTAAATTTGAAATG
AATTTTAGACAAGCAGGATTTATGTTCCCCAAACCAACTCGGGGCAATGACCCTCACTTGTCCCCCACCTCATGT
ACTAAGCCCCGTTTGATTATCGTTCTTTGGTCACAAAAATAAAAAATATAATCGCTATTTATTGTATTAAGTTAC
TATATTTGACTTTTTATCTTTTGGATAGTTTTCATTATCATTCTTACAAAAATAATACCAAATGGGACCTAAGTA
GGGAACCTCCATGCATTTGGGGCGAGGTCCAAGCGTATAAGCTTTTTTGCCATCCTTAATCTCATTCTTCACCCG
CTTTCTCCTCGGAACTACCACCGGCAGCCACTGAGTTTTTTTCTCACCACGGTCGACAATCACAGAAAAAATAAG
TCTACAATTACCGGCAACAACCATAGAAAAACAGATATCCAGTCAATCACAACATCCACCTCATAGTGACGACGC
TACCACAACTATAGAAAGACAGATCTACAGCCACATCACAACACCCACCTCATCGCGGTGGCGCCACCAACATCA
TCCCGCCATTTCACCCTTTCCACAACGTCTCTTCCATTTTTTTTTTTCGCATCTGCATTCTACACAACTGACGGC
ACAGACACAAAATCACCACCAACCACGTTGATAGAGTAGGAACCTGCACGGGCGCTGTCGATCTAAAAGTCAACA
CCACGGAAGACGGAGCGGCAGAAATGAGTAAATATGTGAAATTTTAGGATTAGAGTTTAGTTATTTGGGATAAGA
GGAGTAAATCTGTGACGAATTTAGGGGGCAAAATGGGAAACTAAAGAGTTTAGAGAAGATAGTGGTGGCACTTGA
AGATGGCCATAGCTTTTTCCACAATTAGCCCTTGTTTGAGCCATATTATTTTCTTTAGAATGCATTTTTGAATAA
TGTATTGATGAAAAATGTGTGGTTAAAAAATAAAATAAAGTTTTGAATTTGCATGTAATATTATTTTATTGAAAA
ATGTGCGATTGAAATGATATTAAATCACTATTTAAGAATATATATTTTTTTTTTAAATGTGGCTCAAACATACCTT
GACACCGTCTGACATAGAAATCCGTTATAAATGTGAGTAGGTGAGGGCAATATGTATTTGTTAATGGAGGAGAAT
ATTGGAGTTAAAAAATCAAATTGTGCAGTTTTGAAGTTCTATAATGATTTATGCAAGGATTAAGATGTTTTTATC
CGAGGGGGTAATCGAACTGTGTCCTCCCTTCACAAACATGCAGGCCCCGCCCCTTATTAAATAATTCAAGCCGTG
GTTCCCACCTCATGTTGTGTGTAGTAGGTAAAAGGTTATATAGATGATTATGTACGTGTAGTATGACCCTTTATT
TAGGATTAGAATTTAGTTATTTGGGCTAAGAGGAGTAAATCGTGAGCTCACGAGCCACGAGCTGCTCGCGAACTC
GTGATTTTATTTTTTTATTTTTTTTTCACATTTGTGGACCTTTTTTTCATAATTTTTTCATATTTTTTTTATGAGC
CCACGAGCTCAGCTCAAGCTTGTGGGATTGAGCCCCCTGCTCAACTCGTGCTCAGGCTCGGGCTCGTTTTATCCC
ACGAGCTGGGTTTTGACTAGCCGAGCGTTCGAGCCGCTCGGCATGTTTGACATCCCTACCTCTATTCAATAAGTG
GCATAATGGTTGGGCTAGGAAGTGGGGTCTAATGGAGAAAATCTTCGGTGAGGTGATCGTATTAGCGCATGTGGA
ATGTCCACCCTAACATAGAACGGCAGGTGTACAATTTTAAGTGGGTTGGTAAATTATTTTGCTGTCTTACTTTTT
CTGATTTTTCCTTTTTTATTTTATTTTTTTGAAAAGGGCTAGAAACCACCCCTCGTTAAACTGGAGTAACAGAGA
AGTTGTCTTGAACAAAATAGTAATAGCACTCTTAATATACAGTAATGTCAAAGCAAAAACACCTTCATTAAAGAA
AACGATATTTAATATTGTCTAAAAATCGGAATAGTGTAATTCAATTTATCCATTGAAATTTAGCAAAATTACACA
TAAGCGTCTTTTTATCTTCTACCATAACTTCAATCAATTGAAAAATGTAAACAAACTAATAAATGAAAGGAAAAT
TGCGACAGTGGTCCCCTAACCTTTCACTTTTTTGCAAACTTTACTCAAACATTCAATTTGTGATAGCGAAGATCT
CCAACCTTTCAAAAATGTAGAGCAAAGGTCCAAATGGACGGTTTTCGATTTTTCATCCATTTGACTATTAGTTGA
CCAAAAACAATAAATTTTGACCAATTTTGACTAAATAAAAAAGGGTGAAATAGTAACTTGATAATTACCATATTA
ATAAATGAACAAAAAAACTAGGGAAAATAAAAAAACCAAAAAAATTAAAAAAAAAAAAAAAGAAATAAAAAATTG
TTGTCTCCTCCAGCGCCCTGCCCCACTCCACTTTACTCCGTCTCACCATCTCTACGTCATCCCACCCTCCTTCCC

TTCGAAATCAGCCACCAGCACTGCCACCTATCGTCGATAACATCGCCAAAAAATCAATAACACGCCCTGCTGGCA
ACATCTCCTTCTCACACCCATCACCAAAAATCCAATATATATACCCATACTCCTTATACTTAATCAATTTTTATA
ATGCCTGTTATAAAATTATATTAGAAAATTGAACAAATTTCTCCCAAACCTCAAGATCTTTCAACAATCGTTTAT
TGCCTAGATCTGCAATAAGCAATAATAAAAGGCGTCAGCTGAGATTAAAAACTTGCCATTTTCTTCCATTTCCTT
CGTGAATAAAATCAGCCTCAAAACTAGAACGGTGTTTTCCTGTGATTTCCAACCTTAAAATCTTAACAATCAACA
GATCAATTTAAAAAAATGTTGAGGTGGGGTTTTTTGTTTTGGGTGTAGGTTTGATGGTAATGGTGGTTGTTCACC
ATGCAACTCTTACTCAATGGTTGGGTGGGGTTTTTGGTTTAGTTTTGATAGTAGTAGTGGCAGTTTGTTTTTGGG
TAACTGTAGCTGATCGACAGGATGTGGTAAGTGGGGTGGCTCTAGCTATTGACATTGCTAGTTACTGTGGGGATG
GGGCTGGGGCTGGGGTGTTTGGAGGAGATAACATTTTTTCTATTTTTTGGTTTTTTTTCCTGATTTTTTTGTTCA
TTTAATAATATGGTAATTATCAAGTTACCATTTTACCATTTTTAAATTGGTCAAATTTATTTTTCTTTGTCAGTT
AACGGTCTAAGGATGGAAACCGTACATTTGGACCTCTGCTATAATTTTTGAAAGATAAGGACATTTCCTAGTAC
AAATTGAAGTTTAGGGTAAAGCCCGTAAAAAAGTGAAATGTTAGAGACTGCTACTGCAATTTTTCCTAAATAAAA
TTTACAATCCAAATTAATTTGTACAAAACTTAGAACTTCATCAAACGCATTTTATTTAATAAAGCTCATACCAGT
GTTATATCAAAATTTTAGCTATGATGTCATTAATCTTTTCTTTGCTGATCATCAAAACTATAATAATAATAATAA
TAATGAGAAAAAAATCTTAGTCAAGTAATCATATCGTTGTGGTCTTGAAGCATGTTTGGAAGGTGAAAATTTGAA
CACCCGAGAGCTAAGTGATGAATAAACAAATTAAAGATAGTTCATGTAGGCAAAAATATTGGGTAAGGTGTGCCA
CCATTATTTTAATGACATTCTTCGTTTACTGTAAGCTGATCATCGTAAATGATAATAACTGTTATATTATTTAAG
AATTGAGTAAGGGTAATCATTGAGGTAAAAAAAAAAATTATCATATTTTGACTTTGCTAAAACATTGAAGCAAAG
TTAAAATATGATAATTTTATTTCACCCTTTTTTAGCAAGGTTAAAATGTTTTAGCAAAGTTTGTCCATCTCTTAC
TTCAATCATTTCCCCCTTTTCTCTTTGCAAATAATTTCTCATGTTTTTTTTTGGTAAATCCAACATTTTAATCTT
CACCAACCGCATTTGTGAAATAAAATTATGATTTTTTTTTGTCATATAATGGCATTAACCATGCAATTAACGACA
TCTGCTTAAGCATTAGACACTTTACGTCTTGATTTGGGAAATTGTCTTTCTTCAGGTCTAGCAAGTTCATGGTTA
TGATGAGTATTGATATGAGAAATTATCCATATTCCACCGCTAACTGCAAAACGCACCTTAAATTTGCAACCTATT
CTTGTCTCTAATCTATTATATTTCCTCACATCAAGTTACTCTCCATCCCTTCGAGACCTTTCATTTGAACATAAA
AATTCTCGTCGTTTAATATTTTTTATGCAACTCTTCGAATATGCCCTTTGCGAATACTAAAACTTTTTCTTATAG
CATATGAATTACATATACTATAAGCTTCTTCATCAAAACATACTTACACGTTGCGTTTGTACCCAAATTAGGGTG
AATTTTTTTTAGAAATAAACTATTTCCTTATTTCCATGTGGAAATATTTGTTTCCTTATATTTCACGTGATTTTT
ATGAAAATATATTATTTCCTTATTCCACATTATTTACATCTTTATGCGATGACATGTACCTATAAATACTGATCT
TCTCTTTCAGTTTTATTCAAACAAGAATTGTTAAGTTCTTGTATTTCTTCCCATATTGTCGATTAATAAAAAACG
TCGTGCTTTTGTGATTAACTCTCTTTGTTAATTTCTGTACTTTTAAATCTTGTTTCTCGTTGCAATCTTCATTAC
TTCATATCGCGTCATTAATCTCATTCACTACAATCCTTTCGGGTCCAACAAATGGTATCAGAGCTTGGATCTTGT
TGTGGATTGGTTAAAGTTTTTCAGAGATGGCTGCTTTGAAGTATGATATTCCATAATTGAATCACAATACAAGGT
TTTCTTTGTGGCAAGTAAAGATGAAAGTTATTTTGGCACAGTTAGATTTGGATGATGTGTTGCTGGGTTTTGAGA
AGATACCAGCTAATTAGACAACGGAGGAGAAACAACAAAGGGATCAGAAGGCTTTGTCGTTGATTCATTTTCATC
TATCCAACAATATTCTGCAAGATGTCTTGAAGGGAAAAATAGCAGCTGCTTTATGGTTGAAACTGAAGCAGTTTT
GTATGATCAAGAGTCTTGCTAGGAAACTGGCTCTAAAGCAGAAGCAGTGTTCCTATCGCGTGGCAGAAGGAGAAT
CGTTGGAAGACCATATTGCTGTGTTAAAGGAAATTATCGCAGATTTGGAAACATTGGAGGTTAAGTGCGATAAAG
ATGATCTTGCCTTAATCCTATTATGTTCGTTGTCTGGCTCTTATGCGTCGTTTCGGGACTCCATTTTGTATAGTC
GAGATACTTTAACTATAGACGACGTCTATGATGATTTGCACTTAAAGAAGAAGTTGAAGTATTTGGTTGGAGCTA
CATCAAAAAGATGAGGAAGCTATTGTATCTCATGGTGGTCGACAGAGGGGCGGATCGAAGAATAACAGCAATTTT
TTTTTGCTTCTACTACATACATAAGGGGGGACATATTCGGAAGGATTGTAATAAATTGCAAGCTAAAGGTGATTT
AACTACAAATCGTAGAGCGCAACCTTTGACTTCATATGAAACAAATACAGTTGAGGATTTTTCAGACGACAGAGA
TGTGATGTTAATTGTAATATTTGACATGGAATCTATGCTCGATGCTTCAACTTTAAGGTTGCAAACCCAAGTGCA
TGTTGTCTCAAGTAGTAAACTCGAAATTTCGAGGTCAAACCATAAAGAATTGGAAGGAAATAAACTATTCTATGA
GTGGTGAACAGTTGGTGGAAATACGATGGAGAAGAACACTAGAGTGTTGAGGATTCACTTCGGATAAATGAGTAG
AATTACTCTTGATCTTTTATTTAACTCATATGAAATATGGAAAATAAAACATTGATTGCAAATATACTAAAAGAT
AGATCTTGATAATTATTTGAGGGAAAATATGTCAAAATAAGTTGTTGGAATGTAGAGATTATCTAAGCATTTATT
GTGTCAAAATTTGTCAACAATAAATCAAAGCCTTTAACAAAGCAAAACTGTTTATAGAACATATACTTCAACCAA
GCAAGAAGATGAACATTGAAGAACAAAGTGAAAAATCGAAGACAATACTACGCATTCACCAAAGTTTCACCTCTA
GCTCTAGCTAATGGTTTAGAAATACATAATGAAATGTTACAAAAAAATGAAGGAAACTAAAATATGATGACGAAG
AATTGATGCTCCAAAATCTTGGCCAAGGTCCCTTTTACAGGCAAAGAAATATCCAAAATGCAAGTGGACAATGCA
ACTTGTTTCTGTCCAGTTTTAAGACAGTTGGAACATGCAACTTGATGCCAATTTTCAGATACTTTGTGCAATGAA
TTTAGACTTATGATCGATAGAAAAATTGTTTCCACTTGAGTTAGCTTTCCATTGGTGTAAATATCAGGTCCATAG
GATCTATATAACTCTAGATATGGCCAAAACAATGAGTGCTGCTTCACAGGTAGAAAAGTTGTATCCGGTAGGTAA
CAAACTGGACTTTACACAGTAATTTTGGCTACCTTATGATACAAATTTGACTTCTTGGTAAGCATAGAAGTTGTA
GATACATATCATATCTTTCTAATCCAACTTGAATCACCTTGTTTCGATAGCTACAACTCGAGTTATGACTATTTT
ACCGAGACAGCTGTATCTTGCTCAAGACAGCTAATCATTTTTTTACTTTTCAACATCTTTTCTCCACTTGCTCCA
CTTGTTCTATCTTGGAAATACATGTGAAATATATCGAATAAATACTCTTTCAAAAGTATAATACTCATGCCAATA
TGTATTTAAATACGTATTTCGATTAATTGCATTTTTAACCATGTATTTATGGAAATTGAATTTACAACAAGAGAA
TTGATATTAAAGACTAACATTAGTGAATTTAAACAAATTGAATGTAAAATGTCTTGTAAATTGTATATCTTTGAA
GCATTTTTCGACAATAATCACCTGACGAGGAGTGAATCCTTGATTCGACATGTTCGCATCATATATTTTCCAATC
AAAATTTCTTTCAGACATATGAGGCTGTCAAGTAAGGTCGAGTTGTGATAGGAAACAACCAGTCTTGTCGTGTTG
ATGAAATCGATCGAGTCAGATTAAGACTGCGAGATGGGACAGTCAGAACAATTGATCAGGTTCTGCATGTTCCAG

ACCTGGAAAAGAATATCATCTCACTTAGTACTTTGGATGCAAAATGGTATAAATTCTCTAGTGGGAGTGGGGGTG
GGGTGGGGTTCTGAAGGTTCAAATGGGAGTCCGAATTATATTGGAGGAACAGAAGAAGTCTCAGTTGTGTATCTT
GCTGGAAGCGCAGAACTAGTTAAAAGATATCGGGACAAGTTCAGTACTGTGATAGATATTCAGTTTGGTACAGTA
CCTCTTATGACGAGCTCTGGGTCAGTTCAAAACATTGATCGGGGGAGCATTGGCGATTCTACTTTTCCACCAATT
TCAGCAGATACACTGTGTTCATTTGCTAGGGTTGGACTCATGGAGCGTAGATACCGTGGTGTCAATAATGTTTGG
GATGCTTTTGGGGACGTTGAAGGCGACAGTTCTAGCAATGGGCTTTCTGATCCAATGTGTTGTATGGTTTCTGCT
ACAGGATGTGCTTCGACTCTATTTGGTCAATAGTAATCCCTCAGTGAAATTTTGGAAACCTGAGATTTGGGTTCA
AATGTCGTTTATGGACGCCTTGCAGAAGACGACGGTTTGGTTTGGAGAGTTTTGGTTACTGTGTTGAAGAGATTG
ATTATTTCGATGCAAACGGATTTGAAATCTTGGAATTTCCTTTGTTATACTAGAATTTGGGTCAAGGCGGAGTTT
GTTGGGTTTGTTACCCAAATTAGTGTTTTTTTTTTTTTAAATAAATTGTTTCCTTATTTTCATGTGATTTTTATG
AAAATATATTATTTCCTTATTCCACATTATTTTCATCTTTATACTATGTCATGTACCTATAAATAGTGTTCTTCT
CTTTCAATTTTGTTCAAACAAGAATTGTTGAATTCTTAAATTTCTCTCTATATTGTCGATTAATAAAATTTCATG
CTTCTGTGATTAACTCTATTTGTTAATTTCTATACTTTTAAGTCATGTTTCTTATTGCAATTTTCATTACTTCAT
ATCGTGTCATTAATTTCATTCATTATAATCCTTCCGAGTCCAACATTACATTCCTATATTAAGTACTTCATAATT
TTTTTCTCTACTACTTAAATGCTCCATTGGATAGTAAAATATTAAAAATCCATAGTAATATCTAGGAGTGTTAAC
GAGTAGAGCTGAAATTCTTGGCTCGGGCTCAGCTTGAACTTTTTAAGACGGGCTTTTTTTTTTGCTCAAGCCAAG
CCAGATGAAAGCTCCGCCAAAAAAATAAGGCTCAACCGGAGCTTTCGACTCGGGCTAGCCGAGCCAAGCTCAGTT
TTTTGGAATTTTTTTAATTTTTTTCCCTAAAAGTGGCATTTGCTGCCTGTTTGGCAGCTAAATATAGGCTGCCTT
TTCTAATCCCTTTTTTTACGAGCCGACCCGACTTGAACCGACTCCCCAAAATGGGGCTCCGTCCGAGCCAAGCCG
ACTCGGGCTTTCCCCAGCCCAAATTTCAGGCAATCTGAGCCGGCAAGCCCGATTGTCATCTCTAGTAATACCCAT
TCAACCCACTTTAATATCAACAAAATAAATACAATAAAAGATTATTTCACTTTCCCATTTACATTACAAAATTCT
AGAAAAATGAAATTAATAAATTAAAATGCATATATGATTTTGCTGGGTTGCAAGTGTCCAGAAAGCATGGCAACA
AGACTCTAAAGAGAGAGAGAGAGGGGGGCTTCTCTAGAAGACGGGTCTAATTTTAAAAGGGAAAAAAAAAAGTGG
GACCATAAATTTTGGATGGAGCCCAATGTATTTTATTTTTTTTTAATTTAAAATATAAACATTTGTCAAAGGTTG
CTTAAAATTGTACACGTGTCATTCTGTGATAGGGTGGACATTCTGCATGGGTGGTACGACAGCCTCACCCAAGAT
TTTCTCGATGTAATGAGAGAGATTAATTATTCAATTACGCTACAAATATACCTATTTTTACATCATTTTTGACAG
CCCTCACACGATACAAGTGGGCCCACGATTAAAATAATCGTGAGACTCACTCGTGTGAGTGGGCTGTCAAAGTGA
GTGTTCAGCAGATGCTGTACACTCAACTTAATTCTTAATTATTACAGGACAACTTTTACAGGATTATCTCCATTG
TTGCAATAGTTAGGATAGAGGTGGGATAATCGATATATGTTTTTTTGGGGGAAAAAAATAATAGCACCTAGTTTA
TGAAATTGAATATTGAA**ATG**TAGGGGGTTTTGAGGTAATTTTCCCTTGAATGAATTTATAATTATACTTGCCTTG
GAAGTTCCAAGAGTACTTCATTAATTTCTACTAAAATTTTAATGAAAAACTAGTATATTAATTGATAATCTGTAC
CTTTCGGTTGTAAAAATTAGGAACTATACTACCACTTAATCATTATAAAACCTCACAGAAATCCAATAATATTTA
TCAAAAATAAAATAATTAACTATAATCTGGTGTTAACTGTAGCTGGGAGCAGAGTTTGTTGGCACATTTATCCTG
ATATTTGCTGCAACGGCCGGACCAATCGTAAACCAAAAGTACAATCAAGCCGAAACACTTATAGGCAACGCAGCA
TGTGCTGGTCTCGCAGCTATGATCGTGATTTTATCAACAGGTCACATCTCGGGTGCTCATCTTAATCCGTCACTC
ACTATTGCTTTTGCTGCAGTTCGCCACTTCCCATGGGCTCATGTACCAGGCTACATTGTAGCACAAATTTCAGCC
TCCATTTGTGCTTCCTTTGCGCTCAAAGGTGTTTTCCATCCTTTTATGTCCGGTGGTGTTACTGTCCCTTCTGTC
AGCATAGGCCAGGCTTTTGCCCTTGAATTTCTTATAACCTTCAATCTGCTATTCGTCGTCACTGCTGTTGCCACT
GATACCCGCGCTGTGAGAAATCAATTGAACATGAACATTCTTTTCATCAAATATTACAAATAACTAATCGCTATC
TGAATTAATCTTGTTGATAGGTTGGAGAATTAGCAGGCATAGCAGTTGGAGCCACCGTCATGCTTAATATTCTTG
TTGCGGGGTGAGTAAATGATCCTTTTGCTACGTCGTCTTCTTCTTGAAAGTAATAGTAAATGAAAAGGGTGATGG
AAAAAAGGACACTAGCTAATTATTTTGACTCTCTTTGAATTGGCAGACCATCGAGTGGTGGCTCGATGAATCCTG
TCCGAACTCTTGGACCAGCAGTGGCAGCAGGGAATTACAAGGCAGTCTGGATATACCTTTTGGCACCTACACTTG
GAGCTCTTGCAGGGGCTGGCATTTATACTCTAGTTAAGCTTAACGGAGAAGAGGAAGAAGCCCCAAGCCAGGCGA
GGAGTTTCCGTCGC**TAG**
>*Oleur*NIP5;1a
ATGCCGGAATTCGAAGTCGAAACTCTGACAATGTCGGCGCCAGCGACGCCTGGAACGCCGGCGCCACTGATTTCT
TCTCTGCGAATAGACTCAGTTTCTTACGATCGGAAGTCAATGCCACGATGCAAGTGCCTGCCAGTTGATGCACCC
ACTTGGGGTGCACCACATACGTGCAGCCTCACCGATTTCTCTGTGCCTGACATCTCTCTTACTCGCAAGCTGGGA
GCAGAGTTTGTTGGCACATTTATCCTGATATTTGCTGCAACGGCCGGACCAATCGTAAACCAAAAGTACAATCAA
GCCGAAACACTTATAGGCAACGCAGCATGTGCTGGTCTCGCAGCTATGATCGTGATTTTATCAACAGGTCACATC
TCGGGTGCTCATCTTAATCCGTCACTCACTATTGCTTTTGCTGCAGTTCGCCACTTCCCATGGGCTCATGTACCA
GGCTACATTGTAGCACAAATTTCAGCCTCCATTTGTGCTTCCTTTGCGCTCAAAGGTGTTTTCCATCCTTTTATG
TCCGGTGGTGTTACTGTCCCTTCTGTCAGCATAGGCCAGGCTTTTGCCCTTGAATTTCTTATAACCTTCAATCTG
CTATTCGTCGTCACTGCTGTTGCCACTGATACCCGCGCTGTTGGAGAATTAGCAGGCATAGCAGTTGGAGCCACC
GTCATGCTTAATATTCTTGTTGCGGGACCATCGAGTGGTGGCTCGATGAATCCTGTCCGAACTCTTGGACCAGCA
GTGGCAGCAGGGAATTACAAGGCAGTCTGGATATACCTTTTGGCACCTACACTTGGAGCTCTTGCAGGGGCTGGC
ATTTATACTCTAGTTAAGCTTAACGGAGAAGAGGAAGAAGCCCCAAGCCAGGCGAGGAGTTTCCGTCGC**TAG**
>*Oleur*NIP5;1a
MPEFEVETLTMSAPATPGTPAPLISSLRIDSVSYDRKSMPRCKCLPVDAPTWGAPHTCSLTDFSVPDISLTRKLG
AEFVGTFILIFAATAGPIVNQKYNQAETLIGNAACAGLAAMIVILSTGHISGAHLNPSLTIAFAAVRHFPWAHVP
GYIVAQISASICASFALKGVFHPFMSGGVTVPSVSIGQAFALEFLITFNLLFVVTAVATDTRAVGELAGIAVGAT
VMLNILVAGPSSGGSMNPVRTLGPAVAAGNYKAVWIYLLAPTLGALAGAGIYTLVKLNGEEEEAPSQARSFRR*

***Oleur*NIP5;1b Oleur061Scf3450g00011.1**

>Oleur061Scf3450:131943..**143208** (- strand) class=gene length=1126

>*Oleur*NIP5;1b
ATGCCGGAATTCGAAGTCGAAACTCTGACAATGTCGGCGCCAGCGACGCCTGGAACGCCGGCGCCACTGATTTCT
TCTCTGCGAATAGACTCAGTTTCTTACGATCGGAAGTCAATGCCACGATGCAAGTGCCTGCCAGTTGATGCACCC
ACTTGGGGTGCACCACATACGTGCAGCCTCACCGATTTCTCTGTGCCTGACATCTCTCTTACTCGCAAGGTTAGA
CTTTACACTCTCTCTCTCTCTCTCTCTCTCTCTCTCTCTC//**ATG**TCGGGGGTTTTGAGGTAATTTTTCCTTGAA
TGAATTAACAATTATACTTGCCTTGGAAGTGACAAGAATACTTCATTAATTTCTATCAAAATTTTAATGAAAAAC
TAGTATATTAATTGATAATCTGTACCTTTCGGTGGTAAAAATTAGGAACTATACTACCACTTAATCATTATAAAA
CCTCACAGAAATCCAATAATATTTATCAAAAATGAAGTAATTAACTATAATCTGGTGTTAAGTGTAGCTGGGAGC
AGAGTTTGTTGGTACATTTATCCTGATATTTGCTGCAACGGCCGGACCAATCGTAAACCAAAAGTACAATCAAGC
CGAAACACTTATAGGCAACGCAGCATGTGCTGGTCTCGCAGCTATGATCGTGATTTTATCAACAGGTCACATCTC
GGGTGCTCATCTTAATCCGTCACTCACTATTGCTTTTGCTGCAGTTCGCCACTTCCCATGGGCTCATGTACCAGG
CTACATTGTAGCACAAATTTCAGCCTCCATTTGTGCTTCCTTTGCGCTCAAAGGTGTTTTCCATCCTTTTATGTC
CGGTGGTGTTACTGTCCCTTCTGTCAGCATAGGCCAGGCTTTTGCCCTTGAATTTCTTATAACCTTCAATCTGCT
ATTCGTCGTCACTGCTGTTGCCACTGATACCCGCGCTGTGAGAAATCAATTGAACATGAACATTCTTTTCATCAA
ATATTACAAATAACTAATCGCTATCTGAATTAATCTTGTTGATAGGTTGGAGAATTAGCGGGCATAGCAGTTGGA
GCCACCGTCATGCTTAATATTCTTGTTGCGGGGTGAGTAAATGATCCTTTTGCTACGTCGTCTTCTTCTTGAAAG
TAATAGTAAATGAAAAGGGTGATGGAAAAAAGGACACTAGCTAATTATTTTGACTCTCTTTGAATTGGCAGACCA
TCGAGTGAGTGGCTCGATGAATCCTGTCCGAACTCTTGGACCAGCAGTGGCAGCAGGGAATTACAAGGCAGTCTG
GATATACCTTTTGGCACCTACACTTGGAGCTCTTGCAGGGGCTGGCATTTATACTCTAGTTAAGCTTAACGGAGA
AGAGGAAGAAGCCCCAAGCCAGGCGAGGAGTTTCCGTCGC**TAG**
>*Oleur*NIP5;1b
ATGCCGGAATTCGAAGTCGAAACTCTGACAATGTCGGCGCCAGCGACGCCTGGAACGCCGGCGCCACTGATTTCT
TCTCTGCGAATAGACTCAGTTTCTTACGATCGGAAGTCAATGCCACGATGCAAGTGCCTGCCAGTTGATGCACCC
ACTTGGGGTGCACCACATACGTGCAGCCTCACCGATTTCTCTGTGCCTGACATCTCTCTTACTCGCAAGCTGGGA
GCAGAGTTTGTTGGTACATTTATCCTGATATTTGCTGCAACGGCCGGACCAATCGTAAACCAAAAGTACAATCAA
GCCGAAACACTTATAGGCAACGCAGCATGTGCTGGTCTCGCAGCTATGATCGTGATTTTATCAACAGGTCACATC
TCGGGTGCTCATCTTAATCCGTCACTCACTATTGCTTTTGCTGCAGTTCGCCACTTCCCATGGGCTCATGTACCA
GGCTACATTGTAGCACAAATTTCAGCCTCCATTTGTGCTTCCTTTGCGCTCAAAGGTGTTTTCCATCCTTTTATG
TCCGGTGGTGTTACTGTCCCTTCTGTCAGCATAGGCCAGGCTTTTGCCCTTGAATTTCTTATAACCTTCAATCTG
CTATTCGTCGTCACTGCTGTTGCCACTGATACCCGCGCTGTTGGAGAATTAGCGGGCATAGCAGTTGGAGCCACC
GTCATGCTTAATATTCTTGTTGCGGGTGGCTCGATGAATCCTGTCCGAACTCTTGGACCAGCAGTGGCAGCAGGG
AATTACAAGGCAGTCTGGATATACCTTTTGGCACCTACACTTGGAGCTCTTGCAGGGGCTGGCATTTATACTCTA
GTTAAGCTTAACGGAGAAGAGGAAGAAGCCCCAAGCCAGGCGAGGAGTTTCCGTCGC**TAG**
>*Oleur*NIP5;1b
MPEFEVETLTMSAPATPGTPAPLISSLRIDSVSYDRKSMPRCKCLPVDAPTWGAPHTCSLTDFSVPDISLTRKLG
AEFVGTFILIFAATAGPIVNQKYNQAETLIGNAACAGLAAMIVILSTGHISGAHLNPSLTIAFAAVRHFPWAHVP
GYIVAQISASICASFALKGVFHPFMSGGVTVPSVSIGQAFALEFLITFNLLFVVTAVATDTRAVGELAGIAVGAT
VMLNILVAGGSMNPVRTLGPAVAAGNYKAVWIYLLAPTLGALAGAGIYTLVKLNGEEEEAPSQARSFRR

***Oleur*NIP5;2 Oleur061Scf1510g04020.1**

>Oleur061Scf1510:484673..488548 (- strand) class=gene length=3876

>*Oleur*NIP5;2
ATGCCGGAATTCGAAGCCGGAACACCGACATTGTCGGCGCCAGCGACGCCAGGAACGCCGGCGCCACTGATGTCT
TCTCTGCGCATAGATTCGCTGTCTTATGATCGAAAATCAATGGCGCGATGCAAGTGCCTTCCAGTTGATGCATCC
ACGTGGGGTACACCACATACCTGCAGCCTCACCGACTTCTCTGTGCCTGACATCTCTCTTACTCGCAAGGTTAGA
CCACTTTACGTTACAGTTAGATCTCTCTCTCTCTCCCTTGCCGCCTCAAGCATACTTAAGTAGTTCAAAGGATAA
GGGTGAGGTATATTTAGTCTTGTTGACAATCACACATAAAGGGATTCATTCCTGAAACATGAATTGAAACTCTTT
ATGGCATCAGTATACTGACAACTTTTGTTCCTGACTTGTTAAGACACATTCCATGATTAACTTCTCTCGAGATAC
TCCAAGAACCTTGGGTTCAACTTTTTAAGATCTCTCTCTCTCTCGTGAATATAATATGAACATATATGATTATTG
ACGTGGAAATTGGAATGGGGTCCTTAGCTTTCAGTTGCCTCTTTTAATTTTCTTTGTTGAATACAATATGAATAA
TGATCAGTCAGTCATTTCTTTGTCACGCATCCTATAAATTAAAATGGTAGAAGTATGATTATGGCATGATTTTAG
GTTAGAGGTTTAAATTCACATTTTTTTAACGCTTCTATGATGTGACGTAACGATAAGTTAGCATTGATGCGGCG
ATTATTTGAAACATATGTTGATATATTGTTATTGAATTTTCATCAATTTAGGAGATGATAGTCCAAAATGTTGTG

AAATTTGAAACAAAGCCCTTGTTCGACAATCATACTTTTCAAACTTTTTTTTTCATACTTATTTTCAACCACACT
TTTCACATAATAAAATATTTTGTATGTAAACTCATTTTTTTTTAACTACACATTCTCCTGAAAATCAATATTCAA
AACTCTATTTTATCCAAAAGTATGATTGTCAAACTGGGGCTAAATTGAGTTCAATGATCTAACTGAAACAAAATT
GTAGTTCATTCATCCAATTGAAAGACAATTGAAATTCAATCACCTAATTGAGATTTTGTTGAGACTTCTGAGTTC
AGTGACCGAAAATGAAATTTAACGAAGTGAATTGTTGATGTGGAGTGGGTCCTTCGCTTAGTTGTGTCAAATGGG
ACCGGTCGCTAGGTCAAGGCCCCACCTCATTATAAACTCGTAATGAAACCAGTTCGAGTCAGTTCAAGAGTTGGT
CCGCCAAAATGAACGAATCATGTCGTGTTTGATTGACTAGCCCAATAATCCATGAATTTCGAATTAGTTTGCTCG
ATTATACCTACTGTAATCCTTCAATAATTTCTGTAATGAGTATCGATAGAGAATCTGTACCATTTTGTTGTATAA
ACTAGGATGTACACTGAAACTTATTCATCCTAATGACAGAGGCAAAAACCACTCAGAAATTCGTTAAAATTTGTC
AAAAATGAAGGAATTAACTATGTCAACATAATTTGGTGTTAAATGTAGCTGGGACCAGAGTTTGTTGGGACATTT
ATCCTGATATTTGCTGCAACGGCCGGACCAATCGTAAACCAAAAGTACAATCAAGCTGAAACACTTATAGGCAAT
GCAGCATGTGCTGGTCTTGCCGTTATGATAGTGATTTTATCTACAGGTCACATCTCAGGAGCTCATCTTAACCCG
TCTCTCACTATTGCCTTTGCTGCACTTCGCCATTTCCCGTGGGCTCATGTACCGGCCTACATAGCAGCGCAGGTT
TCGGCCTCCATTTGTGCTTCTTTTGCACTCAAAGGTGTTTTCCATCCTTTCATGTCCGGTGGTGTGACTGTCCCT
TCTGTCAGCACCGGCCAGGCTTTCGCTCTTGAATTTCTTATTACCTTCAATCTGCTATTTGTGGTTACTGCTGTT
GCCACTGATACCCGCGCTGTGAGTAATCAATTGAGCACCAACATTCTTTTCTTTCTCCTTTTCTTCAGATATTAC
AAAGTAATCAAATCCTTGTTGACAGGTGGAGAATTAGCAGGCATAGCAGTTGGAGCCACCGTATGCTTAATATTC
TTGTTGCGGGGTGAGTTAACGATCCCTTTTTGCTTCATCTTCTTCCTGAAAACAATGGTAATTGAAAATCGTGAA
GAAAAAAGGGCACCAGCTAATTGTTTAGACTCTCTTTGAATTGGCAGACCATCGAGTGGTGGCTCAATGAATCCT
GTCCGAACTTTGGGACCAGCAGTTGCAGCAGGAAATTACAAGGCGATATGGGTATACCTTCTCGCACCTACACTT
GGAGCTCTTGCAGGGGCTGGTATTTATACTCTGGTTAAGCTTAATGGACAAGAGGAAGAAGCCCCACGCCAGGTG
AGGAGTTTCCGTCGC**TAG**

>*Oleur*NIP5;2

ATGCCGGAATTCGAAGCCGGAACACCGACATTGTCGGCGCCAGCGACGCCAGGAACGCCGGCGCCACTGATGTCT
TCTCTGCGCATAGATTCGCTGTCTTATGATCGAAAATCAATGGCGCGATGCAAGTGCCTTCCAGTTGATGCATCC
ACGTGGGGTACACCACATACCTGCAGCCTCACCGACTTCTCTGTGCCTGACATCTCTCTTACTCGCAAGCTGGGA
CCAGAGTTTGTTGGGACATTTATCCTGATATTTGCTGCAACGGCCGGACCAATCGTAAACCAAAAGTACAATCAA
GCTGAAACACTTATAGGCAATGCAGCATGTGCTGGTCTTGCCGTTATGATAGTGATTTTATCTACAGGTCACATC
TCAGGAGCTCATCTTAACCCGTCTCTCACTATTGCCTTTGCTGCACTTCGCCATTTCCCGTGGGCTCATGTACCG
GCCTACATAGCAGCGCAGGTTTCGGCCTCCATTTGTGCTTCTTTTGCACTCAAAGGTGTTTTCCATCCTTTCATG
TCCGGTGGTGTGACTGTCCCTTCTGTCAGCACCGGCCAGGCTTTCGCTCTTGAATTTCTTATTACCTTCAATCTG
CTATTTGTGGTTACTGCTGTTGCCACTGATACCCGCGCTGTGAGTGGAGAATTAGCAGGCATAGCAGTTGGAGCC
ACCGTATGCTTAATATTCTTGTTGCGGGGTGAACCATCGAGTGGTGGCTCAATGAATCCTGTCCGAACTTTGGGA
CCAGCAGTTGCAGCAGGAAATTACAAGGCGATATGGGTATACCTTCTCGCACCTACACTTGGAGCTCTTGCAGGG
GCTGGTATTTATACTCTGGTTAAGCTTAATGGACAAGAGGAAGAAGCCCCACGCCAGGTGAGGAGTTTCCGTCGC
TAG

>*Oleur*NIP5;2

MPEFEAGTPTLSAPATPGTPAPLMSSLRIDSLSYDRKSMARCKCLPVDASTWGTPHTCSLTDFSVPDISLTRKLG
PEFVGTFILIFAATAGPIVNQKYNQAETLIGNAACAGLAVMIVILSTGHISGAHLNPSLTIAFAALRHFPWAHVP
AYIAAQVSASICASFALKGVFHPFMSGGVTVPSVSTGQAFALEFLITFNLLFVVTAVATDTRAVSGELAGIAVGA
TVCLIFLLRGEPSSGGSMNPVRTLGPAVAAGNYKAIWVYLLAPTLGALAGAGIYTLVKLNGQEEEAPRQVRSFRR
*

*Oleur*NIP5;3 Oleur061Scf4242g00005.1

>Oleur061Scf4242:66824..76868 (- strand) class=gene length=10045

>*Oleur*NIP5;3

ATGGCGGATTTCGAACCGGGGACACCAGGGACGCCGACAACGCCGACGCCGCTTATGTCGTCTCTGAGAGTGGAT
TCACTGTCGTACGATCGGAAGTCAATGCCGAGATGCAAGTGCTTGCCTTTGGTTTCGTCTACTTGGGCGGCACCT
CAAACTTGCCTCACCCATTTCTCTGCACCTGAAATTTCTCTCACTCGCAAGGTTTCACCATCTCTCTCTCACTCT
AACACACGCGCGCGCACTGAGTTACTGAGATTTGAGATCCTTACACGCACGCACTATTTTCCTGGAGATCCTTGC
ACGCACGAATTCCTGGAAAATATACTCGTTTTCATGTATGGAAACAGGAAAGGAAAAGATGAAATCCAGTCCGGT
TTTTATTTGAAACTTCTTTTGCACTTAGATTGTAGTATTACTATTACTATTACTATTACTATTATTGTTCCACTA
TCTAAGGATCCAGGATATATTACTATTATAATACATATGGTCGGATTAATTTCCGTGCACGTCTGGTAAAGTTT
ATAAGTGTATCAAACTCTATTTTGTACTTGCATTTTCGTACCTATGACTTTTACCCATTTATTATCCGTCGCACT
TATAAGAGTTCGATGTAAGTGCAAATTTTACTATTATTGATATATTGTATTTTTAACGTTGTAAACTAGCAAACA
ATTTGCATTGCCACATTACATAACTGACAGGTTCAATTAACTATTAAAATGTTTTTTTGACCATTTTGTTTATTA
CAATCATTAAGTTAGCTAATAATTTGATTGAACATGTTGATATGTTTATGAGCCAAGACCTTACAATTTGTACTT
GAGATAAAGCTCGTTCATTAACAAGTAACAACTTGGATATATACTGACTTTTCAGCTTCAACCAAACTTTTGTTA
GTGATTCCTTATCTATGACGTGATGCATCATGTCAATCGACAGACATAGTCTGATAGTCGACATAGCCTTTGCTT
CAGGTTCCTTCCAATATGTGTCTTCTGGCTTCGTTCCTTATAACATCTCACCATGTCCTACTGCACTAATAGTTC

CTTGACCTCATCTGCCATAATCCGAAATTTCCAATTCCATTGAACTTTGCATAAGAAACCCCAAACATCATAAAT
TTGGCTCTGATATCGGTTGTTGTGCACATATTAACAGTAAAATTGAAGAGAAGAAGAATAATGAGATTGACACAC
AGGAATTTAAGTGATTCGTGAGAATAATCCACTAACTTCGAATAGGGTTACATAAAGTTTGTTTATATAGAACCC
TAATTATTCTTACAATACAAAATTATGAGCCACCTAATCAACAAGTAGGAAGCCAGCTTAGAATTTTTTTCAGC
CTTGCTTACAGCATAATGTGGCTGTTCATTACATAAAAGACAGACGTGCAATTGCATAGTTTGGTTACGATGTAC
ATTTTATATATTGGGATAGTAATTAGGTTGTGGATTGGGAAATTAGAGGATATTATATATGCTTGTGATGTGATT
ATTTGATGTTTGTGGTGAGTAGTGTAGAGCTTGATATAGAATGGAAAAAGAATCAGGTTGGCCTTTTGGTTTTAG
CAGGTTGCTGCAGAGTTTGTGGGAACATTTATTCTGATATTTGGCGCAACAGCAGGACCAATCGTGAACCAAAAA
TATGATCGAGCAGAAGGTCTAGTAGGGAATGCGGCATGCGCTGGGTTGTCTGTTATGATAGTCATCCTTTCAACA
GGACATATATCTGGAGCTCATCTTAATCCCTCCCTTACAATTGCCTTTGCTGCTCTTCGCCATTTCCCATGGAAT
CATGTCCCGGCTTATATTGCAGCACAGGTTTCTGGATCTATTTGTGCTTCTTTTGCGCTCAAAGGTGTATTCAAT
CCTTTCTTGTCAGGTGGTGTCACTGTTCCTTCTGTCAATAACGGCCAGGCATTCGCTCTTGAGTTCCTCATTACC
TTTAATCTGCTGTTTGTCGTAACTGCAGTTGCCACTGATACCCGTGCAGTAAGTAATTAATAATCAACAATATCA
TGGTCTCAATAGAATCAGACTCTAAAAAACATAAAACAAAGCAGAACACAAAATTAATATCTTGCTGATGACAGT
TTTTAATCAGGTCGGAGAATTAGCCGGATTAGCAGTTGGAGCTACAGTTATGCTCAACATTCTTGTGGCTGGGTG
AGTGTAAATTCCTTCTTGTTGCCCTCTAATTGTCCTTGTTTTCTTCTGCCATTACTTCCTCTTAGATCATCATAT
TCATAATCAGTATATACAAGTTGCCTGGCATTTAAATAGACTCTATCCATCTATTTATTCTCTATTGGATATTCT
AAAAAATTTACTAGAAAGATATTTATGATTTGGCAGGCCTACTAGTGGTGGTTCAATGAATCCTGTGCGAACTCT
GGGGCCAGCGGTTGCTGCAGGAAATTATAAGTCGATATGGATATACTTAGTGGCACCTACACTTGGAGCTCTTGC
TGGGGCAGCTATCTATGCACTTGTGAAGCTTCGAGGAGATGAAGATACGAACCTCAACGTCAGCAGGGCTAGT**TA**
G

>*Oleur*NIP5;3

ATGGCGGATTTCGAACCGGGGACACCAGGGACGCCGACAACGCCGACGCCGCTTATGTCGTCTCTGAGAGTGGAT
TCACTGTCGTACGATCGGAAGTCAATGCCGAGATGCAAGTGCTTGCCTTTGGTTTCGTCTACTTGGGCGGCACCT
CAAACTTGCCTCACCCATTTCTCTGCACCTGAAATTTCTCTCACTCGCAAGCAGGTTGCTGCAGAGTTTGTGGGA
ACATTTATTCTGATATTTGGCGCAACAGCAGGACCAATCGTGAACCAAAAATATGATCGAGCAGAAGGTCTAGTA
GGGAATGCGGCATGCGCTGGGTTGTCTGTTATGATAGTCATCCTTTCAACAGGACATATATCTGGAGCTCATCTT
AATCCCTCCCTTACAATTGCCTTTGCTGCTCTTCGCCATTTCCCATGGAATCATGTCCCGGCTTATATTGCAGCA
CAGGTTTCTGGATCTATTTGTGCTTCTTTTGCGCTCAAAGGTGTATTCAATCCTTTCTTGTCAGGTGGTGTCACT
GTTCCTTCTGTCAATAACGGCCAGGCATTCGCTCTTGAGTTCCTCATTACCTTTAATCTGCTGTTTGTCGTAACT
GCAGTTGCCACTGATACCCGTGCAGTCGGAGAATTAGCCGGATTAGCAGTTGGAGCTACAGTTATGCTCAACATT
CTTGTGGCTGGGCCTACTAGTGGTGGTTCAATGAATCCTGTGCGAACTCTGGGGCCAGCGGTTGCTGCAGGAAAT
TATAAGTCGATATGGATATACTTAGTGGCACCTACACTTGGAGCTCTTGCTGGGGCAGCTATCTATGCACTTGTG
AAGCTTCGAGGAGATGAAGATACGAACCTCAACGTCAGCAGGGCTAGT**TAG**

>*Oleur*NIP5;3

MADFEPGTPGTPTTPTPLMSSLRVDSLSYDRKSMPRCKCLPLVSSTWAAPQTCLTHFSAPEISLTRKQVAAEFVG
TFILIFGATAGPIVNQKYDRAEGLVGNAACAGLSVMIVILSTGHISGAHLNPSLTIAFAALRHFPWNHVPAYIAA
QVSGSICASFALKGVFNPFLSGGVTVPSVNNGQAFALEFLITFNLLFVVTAVATDTRAVGELAGLAVGATVMLNI
LVAGPTSGGSMNPVRTLGPAVAAGNYKSIWIYLVAPTLGALAGAAIYALVKLRGDEDTNLNVSRAS*

***Oleur*NIP6;1 Oleur061Scf9039g00011.1**

>Oleur061Scf9039:119150..124645 (+ strand) class=gene length=5496

>*Oleur*NIP6;1

ATGGATGGTGAGGATATTGCATCAGCTCCTTCAACACCTGTAACACCAGGGACTCCAGGTGCTCCTCTCTTTCGA
GATTTCAAAAGGGACAATAATTGCTTTAATGGTAGAAAATCATCTCTTCTTAAGAGCAGCAAATGCTTTAGTGCT
GAAGCTTGGGAATTGGAAGAAGGGAGTTTCCCCCCTGTCTCTTGTGCATTGCCCCCACATTCTGTCTCTCTTGCA
AGAAAGGTGTGCATGAACAACTCTACTAAATCATCTTGTCTAATTTTCGTTTTATTTTATTTTTTTATTATTTTT
TTTTCTTGAAGGCCTTTTAATAGGTGACAATGATATTTGTGTTAAGTTGTGGAACCTAATTATTGTTGAGCCATA
ATTATTGCTGCTTCGCAGAGATGGTCGCGGATCAAATTAAGGGCTATTTACGAATTTATATTTTTGTAAAATTAT
ATGAAGGAATAGGAGTCTATATAAAATTTGATTTTAACCTAATTTATCTTATAAATAGATAGCAGACCTTAGACT
TTATTTATTATCATCAAATGGAGAGAGAGAGATTGGAGAAAATATGATTGATATTTTTTCTTGTAAACCTTTTTT
CATCATAGTGAATTATTGTAGTGACAGGACAGGACGTAGTCTCATACTGAGACAAACCTCTATAAATCTTGCGTG
CTTTGTTTGTTTATTTCTTTCTATAACTTGATTGTTCTTGATGGGATAGAATTAGGGTTTATAATTCAGAACAAT
TTGAAGTTGGGATTCTCGACTGATATATATGTTTCTTACCAGATGGGAGCTGAGTTTATAGGTACCCTCATACTT
ATCTTCACTGGAACAGCCACTGCCATTGTGAACCAAAAGACAAACGGATCGGAAACCCCTTATTGGCCTGGCAGCC
TCCACCGGGCTGGCGGTGATGGTAGTCATACTCTCCACTGGTCACATTTCTGGAGCTCATCTCAATCCTGCTATC
ACCATTGCCTTTGCTGCTCTCAAGCATTTTCCATGGAAACATGTAAGCCCCTCTTTGACAGCCATTATCCAAAAA
TTATTTTTTTCTACGTACTCATTTTCAAAACATTTTTCTACTTTTAACCACACATTCTTCCAAAATTCACCATTC
AAAACTTTGTTTTTACCAAATAATAGCCATTAAATAGGGCCTAAATTTACATTCATGGCTGACTGATAGTATTAG
AAACTGTTAACAAAAAAGCTAAACTCTTGTCTGTTTCATGTCAACTATGGTGCTCTATCACATTGGATTGATTCA

ACTTTAATTTAATTTGGGTGCAGGTTCCTTCTTACATTGGAGCCCAAGTAATGGCATCATTAACTGCTTCATTTC
TGGTAAAAGGGATTTTCAATTCTATGATGGGTGGGGAGTAACAGCTCCCTCAGGGGGATATGCTCAAGCTTTTG
CTTTGGAATTTATTATCACTTTCAACCTCATGTTTGTTCTTACAGCAGTAGCCACAGATACAAGAGCTGTAAGTC
TCCCTCTCTCTAGTTACTTTCGAGATATATAATTAAATTATGAGTAAATTCCTCCATAGGTTCCAACTTTTTTCA
TAAATATGGCAACCATGTCCCCAATATTTAGTTTGTACTTCCATATTCCCAAACTTCTAATAGCGTACGCACAAT
ATCCTCACCTTATGGTAAATGTCAATTTAGACGTTAAATGTCCATGTGACACGTACAAAATGTTTGATTGAAAGG
ATAAAATTATTTTAAAAAGATTTTTTTTTATTCTTAAAGCAAAATGGTGTTTGTTCAAATTACACGAATGTGTTC
TTTGTTGAAAGATTTTACCCTTTGAGTAAGATAAAACGTGCTTGTTGCAAGACTATTTAATGATGAAATAGACAT
TTTCTATCTAGTGTAAATACTATTTACATAGTGTTAAAAGGTTGGGAACATGAATGTCACAAATAGAATATAAAG
GAGACTGATGCCATAGTTGTGAAAAGTTGAGAACCCTTGAAGAATATCCCTTTAAAATATCATGAGATTATGATA
GAAAATGGAAATATCCCATATAATTTTTCCACTAGGGATTAATTTATTTGCTTCATTGCAGCAATCAAGAGACAA
GAGTCAGTAGTAAATCTGAAATTCAAACTTCAATGAATATTAATTGTAAATACAAATTAAAAATATTATGTGTTG
AGAGACTTGTGTCACTAGCCGAAGAAAGCCAGTCAGATAAATATGTGTTAGATTAACAAGAGGAAATTGTTTAAG
ATGGATCAAATTGGAGAGAGAATGAATTCAATTTTACCTAAAATAGTCTTATTTTTGTTGATATTTCGAGTATCA
CCTACATTCACCCTCTACCTCCCCATGCCTCTTTGTCCCTTCTCCTTGTTAAGGGATAAAATTTCACATCAATTA
AAGATGAAAATATAGATCAATTTATTATAGCTTGATCTATTCCTTTTAATAAGGTCGATCTTTTTAAGATGGAAC
TCTAAATCTTAATCTAGTCTCAGAGTCTCTAACGAGTAATTCGATTAGATTAAAACTAAAAAAAATGAGCATACG
AATTTGCAGATTGGTTTGCTCAAATGAGCCACCATCTAGTGAGGGCATGTTAAGGGATAAAATTTCACATCGATT
AAAGATAAAAAAGTAGACCAATTTTTAAGAATTTGGACTACTCATTTTAATAAAGTTGACTTTTTAAGATGTAAC
CTAAAATCTTCGTAGTCCTCCCTAGATCCGGTCTCTTCCCTTAAGATCCTTAAGAGACTCTCGAGTAGTTCTAAG
CACGACTCAGGACTCCTTGATACTCTTTCGAACATAAAACAAGTAGGGGATACATGGAGCGTAGAAGACTATTAG
GGGACATTGGGCACATCAAACAATAGTATGCCGTACTATCCAGAATAATTTTTAAATTTTTGAAACAATTTCTTA
CTCCAAGGATCTCATCTTGGATCTCACTTCCAGTGCAAAATTAGTCATAGAATCTTCCAATGTGTATGCAGATTG
GTTGTAGACTATAACATATTTTGTGGAGTAACAGGTGGGCAAGCTGGCGGGAATTGCGGTAGGAGCTACTGTCAT
GCTCAATATACTGATTGCCGGGTGAGCTCAAAAGTTTCACTTTCACCTACCATTTTTCTGTTTTTGTTTTTCCAG
TCTATTAATTTGACTTGGATAAAATTCATTTGATGTGCTACATTCAAACGCACTTGCTGCAAAATACTCCATTGG
CTTTACTATTTCACAACAGACAAGTCTTCACCAATTTTTACAACAAAAACAACAGACTTAATTTCACCATTGACA
ACACAAACATCAAGAAAACATTGATAAAAGTGTGACTTCTTCTACGTTCCATTTCAGCATTTTTAACACAAACAT
TGATAAAGTGCTTGTACTTTGGTATTTCTTCTTACCTCCAATTTCCATGCAAGGTAAGATTCCATTTTCTTTTGA
GGCCCTAATAAAAATTTTTAATAATTTTTTAAAAATTGTTAAGTGATTTTTTTTTTTTTAAATTTATTGTAAGATA
TATTTTCTTGCTCATAAGTGAAATTTTTTTACTTATAAGTAATTTGTTTATATTTAAGTGAAATTTTAACTTATAA
TTAAAGTTTTAGTTATTTTTTTTTTTTGTACTTATAAATAAAATTTTAACTTAAAAGAAAAGATTTTAAGTTATAA
GTTAATTTTTTTACATAAGTGATTTTTATTTTTTTACTTTATTTAATTTTTCTATCTTATATGAATTATTTTACTT
GTAAGTGATACGTAAATTTTATAATTATAAGTCAATTTTACAGATATAAGTATTTTTTTTATAGATAAGTAGTTTT
TAGATCATTATACTAAGTAAATTTTTTTCTTATAAGTGAACTTTTAGTTATCACTAGTATTTTTTTATATAGAAGCA
ATTGTACTACATAAAAGTGAATTATATATTACAAATAACTTTTTAATTATAAAGTAAATTTTTTTTATTTATAATT
GAATTTTTAGTTTATGTAGTTATGGTCAACTTTTTACATAAAAGTGTTGTCTTATAAGCCTTTTTTTTCACTTAA
AAATAATTTTTTTAGTCCCGAAGTAATTTTTATTTATAATAGAATTTTAGTTATAATTTGCTAGATAGAGGCAGA
TTTTTCTTTATTTTTTAAGTGATTTTTTACTTATAAGTATATATTTTGTTATAAAGCGATTTTTTAACTTATAAG
TTTTTTCTCTACTTGTATAGGTATTTTTATAACTTAGAAGTTAATTTTACTCATAAGTAATTTTTTGCTCATATA
CAAATTTTGTATTTAGAAGTAATTTTTTTTTTTAATTTTTCACTTATATACATCTTTACTTATTATTAATTTTCTA
TTATGCATAAGGTTTTCCATTTAAAAATGATTTTTATCACTTATAAGTGAATATTTTTTTATTATAATTGAATTTT
TTATAAGTGACTTATAATTCTGAGCTCTTATTACTTGTAAGTAAATTTATGTACATTAATTCAACTAATATAATC
TTAGTTCATAAATTGTTGAATAAATAATATTGTCTGAACCCACCTTTACATTGCATATTAATTTTACGATTGAAT
AAAGGATCAGGTTACTTTAATCTGGTATTAGTTGCACCAAACACCATAAAAGATTAATTAAAATAAATCTAATCC
CTAGCTTGTCTAATAAGATGTGTCAAATATGTCTTAAATCAATAATGATAAACTAAACTCACATGTTTATAAAGT
TGAGAATTATCTTAAATGATTTCCTTTTTGCTATGCCTTTCAATTTGTCGTTAGAAATATTCAATCATCGTTCAC
ATCAAAAACTCCAACAAAAGTGTACGATTATTGTCAAATTACTACAACCCCTCATTTATACATGTATGAAAAACA
TCAATTAATCTACTTGTACAACGTAAGTAGACAAAGTTTGCTGACAAAATAGATGTTGGCCAGATTTAGGCCATT
GATTTGTTTGGATTTCAAAATTCGATTTTGAAATGAGTGATATGGATCTTGCCAACTCGTATTTTGTCAACCTCC
TTTGTCAACGTCCTTTGTACTCATAGCATCACTCTGAAAAACATAGACAGTTATTCAAAGCAAGGATACATTGGA
TGTTTAAAACAACATATAATTTTAGAGGTTAAAACATTGAAGGTTGCTTTACATTAATCCTAATTACATGTAAGT
CACTAAACTTGTTTTTTGTTCCAATTGAAGCATTATAAATTAATTATAGTTTCAATTAATTATGCAGGAAATCCA
CCGGAGCGTCAATGAATCCAGTGAGAACCTTAGGGCCAGCAATTGCCGCAAACAACTACAAAGCCATATGGGTAT
ACCTCACTGCACCTGTCCTTGGTGCCTTGGCTGGGGCAGCTGTTTATTCTGCAGTAAAGCTGCCTGATGAAGACC
CCCTTAACACA**TAG**

>*Oleur*NIP6;1

ATGGATGGTGAGGATATTGCATCAGCTCCTTCAACACCTGTAACACCAGGGACTCCAGGTGCTCCTCTCTTTCGA
GATTTCAAAAGGGACAATAATTGCTTTAATGGTAGAAAATCATCTCTTCTTAAGAGCAGCAAATGCTTTAGTGCT
GAAGCTTGGGAATTGGAAGAAGGGAGTTTCCCCCCTGTCTCTTGTGCATTGCCCCCACATTCTGTCTCTCTTGCA
AGAAAGTTTATAGGTACCCTCATACTTATCTTCACTGGAACAGCCACTGCCATTGTGAACCAAAAGACAAACGGA
TCGGAAACCCTTATTGGCCTGGCAGCCTCCACCGGGCTGGCGGTGATGGTAGTCATACTCTCCACTGGTCACATT
TCTGGAGCTCATCTCAATCCTGCTATCACCATTGCCTTTGCTGCTCTCAAGCATTTTCCATGGAAACATGTTCCT

TCTTACATTGGAGCCCAAGTAATGGCATCATTAACTGCTTCATTTCTGGTAAAAGGGATTTTCAATTCTATGATG
GGTGGGGGAGTAACAGCTCCCTCAGGGGGATATGCTCAAGCTTTTGCTTTGGAATTTATTATCACTTTCAACCTC
ATGTTTGTTCTTACAGCAGTGGGCAAGCTGGCGGGAATTGCGGTAGGAGCTACTGTCATGCTCAATATACTGATT
GCCGGGAAATCCACCGGAGCGTCAATGAATCCAGTGAGAACCTTAGGGCCAGCAATTGCCGCAAACAACTACAAA
GCCATATGGGTATACCTCACTGCACCTGTCCTTGGTGCCTTGGCTGGGGCAGCTGTTTATTCTGCAGTAAAGCTG
CCTGATGAAGACCCCCTTAACACA**TAG**

>*Oleur*NIP6;1

MDGEDIASAPSTPVTPGTPGAPLFRDFKRDNNCFNGRKSSLLKSSKCFSAEAWELEEGSFPPVSCALPPHSVSLA
RKFIGTLILIFTGTATAIVNQKTNGSETLIGLAASTGLAVMVVILSTGHISGAHLNPAITIAFAALKHFPWKHVP
SYIGAQVMASLTASFLVKGIFNSMMGGGVTAPSGGYAQAFALEFIITFNLMFVLTAVGKLAGIAVGATVMLNILI
AGKSTGASMNPVRTLGPAIAANNYKAIWVYLTAPVLGALAGAAVYSAVKLPDEDPLNT*

*Oleur*NIP7;1a Oleur061Scf4622g04016.1 Corrected

>Oleur061Scf4622:475742..477284 (+ strand) class=gene length=1543

>*Oleur*NIP7;1a

ATGCAGCAAAGTATGAAAGCACCATTTCGGGAACTGTCATCGACTCCTAACATTTTAGGTAGTACAGCCACAAGA
AGCCAGCCCAAAAATGACCTAGAAAATGGAGATTCAATGCTAAATGAAACTCTCTTCAACTCTCTTCATAAAATC
GACAAAAGTCTTGTGCGTAAGGTACATGTCTACAAAAGAATCTCCATATTTGCTCGTAAAATCCATCTTTTCATC
GTCTAAATCTGCAGGTATTGGCAGAGGCATTAGGGACATTCGTGCTAATGTTCTGCATTTGCGCGATCATAGCAA
TAATGCATTTAATCGGATTGGAAGCAGGCCTAATGGAATATGCAGCCACAGCAGCACTAACAGTAACTGTTATAG
TTTTCTCCATAGGAGCAATTTCTGGAGCCCATGTCAATCCTGCTGTCACAATTGCCTTCGCAACTGTCGGCCCAT
TCCCATGGTCCGAGGTAGTTCCAAGTCCAAAACTTGTTAAATTTGAAGATACGGATCAAGAAATTGAATTTCAAA
TTTATTTCCCTTTATTCTTTGAAGGTTCCACTCTATATATTGGCACAAATTGGGGGATCTACTTTGGCAACTTAT
ATTGCAAGATTAGTATATGGCATTAGATCAGAGCTTGTGATGACCAAACCACTCCAAGGATGCACTGCAGCTTTC
TGGGTGGAGCTTATTGCAACTTTCATAATTCTTTTCCTCACTGCATCCTTGTCAAATGACCCTCAATCTGTAAGA
TTCTGTCAATTCTACTCGAGTCATTTTACGTTCGACTACACTTATCCTGTGATATGTTCGGTTTAGAGTTATATC
AGACATGTTGCACATATTAGGATATTCGATTTACAGTGTTCCTAGTAATATTTCTTCTGGTATGATATAGTGCCA
TTTTTGCAGATAGGACAGGTGTCTGGCTTTGTTGTCGGGGTGGCCATTGGACTAGGAGTGCTAATTTCTGGGTAA
AGCATAAATAATTACTTATTACAAACTAACTAGAACTCACCAGATTCACTTAAGTCGATTAAATTATCAAATGCA
GGCCTGTTTCAGGTGGGTCGATGAATCCAGCGAGATCGTTAGGACCTGCACTTGTTTCATGGAAATTTGATGATC
ACTTATGGATATATTTAGTAGCACCAACTATAGGAGCCATCGCTGGTGTTTTTGTGTACCGGATTTTACGGCTAC
AAGGTTGGTCTTGTGAATCGAGTCAAGCCCGAAAAGGAAGTGATTCTTTACAT**TAG**

>*Oleur*NIP7;1a

ATGCAGCAAAGTATGAAAGCACCATTTCGGGAACTGTCATCGACTCCTAACATTTTAGGTAGTACAGCCACAAGA
AGCCAGCCCAAAAATGACCTAGAAAATGGAGATTCAATGCTAAATGAAACTCTCTTCAACTCTCTTCATAAAATC
GACAAAAGTCTTGTGCGTAAGGTATTGGCAGAGGCATTAGGGACATTCGTGCTAATGTTCTGCATTTGCGCGATC
ATAGCAATAATGCATTTAATCGGATTGGAAGCAGGCCTAATGGAATATGCAGCCACAGCAGCACTAACAGTAACT
GTTATAGTTTTCTCCATAGGAGCAATTTCTGGAGCCCATGTCAATCCTGCTGTCACAATTGCCTTCGCAACTGTC
GGCCCATTCCCATGGTCCGAGCTTGTGATGACCAAACCACTCCAAGGATGCACTGCAGCTTTCTGGGTGGAGCTT
ATTGCAACTTTCATAATTCTTTTCCTCACTGCATCCTTGTCAAATGACCCTCAATCTGTAAGATTCTGTCAATTC
TACTCGAGTCATTTTACGTTCGACTACACTTATCCTGTGATATGTTCGATAGGACAGGTGTCTGGCTTTGTTGTC
GGGGTGGCCATTGGACTAGGAGTGCTAATTTCTGGGCCTGTTTCAGGTGGGTCGATGAATCCAGCGAGATCGTTA
GGACCTGCACTTGTTTCATGGAAATTTGATGATCACTTATGGATATATTTAGTAGCACCAACTATAGGAGCCATC
GCTGGTGTTTTTGTGTACCGGATTTTACGGCTACAAGGTTGGTCTTGTGAATCGAGTCAAGCCCGAAAAGGAAGT
GATTCTTTACAT**TAG**

>*Oleur*NIP7;1a

MQQSMKAPFRELSSTPNILGSTATRSQPKNDLENGDSMLNETLFNSLHKIDKSLVRKVLAEALGTFVLMFCICAI
IAIMHLIGLEAGLMEYAATAALTVTVIVFSIGAISGAHVNPAVTIAFATVGPFPWSELVMTKPLQGCTAAFWVEL
IATFIILFLTASLSNDPQSVRFCQFYSSHFTFDYTYPVICSIGQVSGFVVGVAIGLGVLISGPVSGGSMNPARSL
GPALVSWKFDDHLWIYLVAPTIGAIAGVFVYRILRLQGWSCESSQARKGSDSLH*

*Oleur*NIP7;1b Oleur061Scf1371g00022.1

>Oleur061Scf1371:75020..76568 (- strand) class=gene length=1549

>*Oleur*NIP7;1b

ATGCAGCAAAGTATGAAAGCACCATTTCGGGAACTGTCATCGACTCCTAACATTTTAGGTAGTACAGCCTCAAGA
AGCCAGCCCAAAAATGACCTAGAAAATGGAGATTCAATGCTAAATGAAACTCTCTTCAACTCTCTTCATAAAATC
GACAAAAGTCTTGTGCGTAAGGTACATGTCTACAAAAGAATCTCCCATATTTGCTCGTAAAATCCATCTTTTCAT

CGTCTAAATCTGCAGGTATTGGCAGAGGCATTAGGGACATTCGTGCTAATGTTCTGCATTTGCGCGATCATAGCA
ATAATGCATTTAATCGGATTGGAAGCAGGCCTAATGGAATATGCAGCCACAGCAGCACTAACAGTAACTGTTATA
GTTTTCTCCATAGGAGCAATTTCTGGAGCCCATGTCAATCCTGCTGTCACAATTGCCTTCGCAACTGTCGGCCCA
TTCCCATGGTCCGAGGTAGTTCCAAGTCCAAAACTTGTTAAATTTGAAGATACGGATCAAGAAATTGAATTTCAA
ATTTATTTCCCTTTTTCTTTGAAGGTTCCACTCTATATATTGGCACAAATTGGGGGATCTACTTTGGCAACTTAT
ATTGCAAGATTAGTATATGGCATTAGATCAGAGCTTGTGATGACCAAACCACTCCAAGGATGCACTGCAGCTTTC
TGGGTGGAGCTTATTGCAACTTTCATAATTCTTTTCCTCACTGCATCCTTGTCAAATGACCCTCAATCTGTAAGA
TTCTGTCAATTCTACTCGAGTCATTTTACGTTCGACTACACTTATCCTGTGACCGCAGATATGTTCGGTTTAGAG
TTATATCAGACATGTTGCACATATTAGGATATTCGATTTACAGTGTTCCTAGTAATATTTCTTCTGGTATGATAT
AGTGCAATTTTTGCAGATAGGACAGGTGTCTGGCTTTGTTGTCGGGGTGGCCATTGGACTAGGAGTGCTAATTTC
TGGGTAAAGCATAAATAATTACTTATTACAAACTAACTAGAACTCACCAGATTCACTTAAGTCGATTAAATTATC
AAATGCAGGCCTGTTTCAGGTGGGTCGATGAATCCAGCGAGATCGTTAGGACCTGCACTTGTTTCATGGAAATTT
GATGATCACTTATGGATATATTTAGTAGCACCAACTATAGGAGCCATCGCTGGTGTTTTTGTGTACCGGATTTTA
CGCTACAAGGTTGGTCTTGTGAATCGAGTCAAGCCCGAAAAGGAAGTGATTCTTTACATTAGGTTG**TAG**

>*Oleur*NIP7;1b

ATGCAGCAAAGTATGAAAGCACCATTTCGGGAACTGTCATCGACTCCTAACATTTTAGGTAGTACAGCCTCAAGA
AGCCAGCCCAAAAATGACCTAGAAAATGGAGATTCAATGCTAAATGAAACTCTCTTCAACTCTCTTCATAAAATC
GACAAAAGTCTTGTGCGTAAGGTATTGGCAGAGGCATTAGGGACATTCGTGCTAATGTTCTGCATTTGCGCGATC
ATAGCAATAATGCATTTAATCGGATTGGAAGCAGGCCTAATGGAATATGCAGCCACAGCAGCACTAACAGTAACT
GTTATAGTTTTCTCCATAGGAGCAATTTCTGGAGCCCATGTCAATCCTGCTGTCACAATTGCCTTCGCAACTGTC
GGCCCATTCCCATGGTCCGAGCTTGTGATGACCAAACCACTCCAAGGATGCACTGCAGCTTTCTGGGTGGAGCTT
ATTGCAACTTTCATAATTCTTTTCCTCACTGCATCCTTGTCAAATGACCCTCAATCTGTAAGATTCTGTCAATTC
TACTCGAGTCATTTTACGTTCGACTACACTTATCCTGTGACCGCAGATATGTTCGTGCAATTTTTGCAGATAGGA
CAGGTGTCTGGCTTTGTTGTCGGGGTGGCCATTGGACTAGGAGTGCTAATTTCTGGGCCTGTTTCAGGTGGGTCG
ATGAATCCAGCGAGATCGTTAGGACCTGCACTTGTTTCATGGAAATTTGATGATCACTTATGGATATATTTAGTA
GCACCAACTATAGGAGCCATCGCTGGTGTTTTTGTGTACCGGATTTTACGCTACAAGGTTGGTCTTGTGAATCGA
GTCAAGCCCGAAAAGGAAGTGATTCTTTACATTAGGTTG**TAG**

>*Oleur*NIP7;1b

MQQSMKAPFRELSSTPNILGSTASRSQPKNDLENGDSMLNETLFNSLHKIDKSLVRKVLAEALGTFVLMFCICAI
IAIMHLIGLEAGLMEYAATAALTVTVIVFSIGAISGAHVNPAVTIAFATVGPFPWSELVMTKPLQGCTAAFWVEL
IATFIILFLTASLSNDPQSVRFCQFYSSHFTFDYTYPVTADMFVQFLQIGQVSGFVVGVAIGLGVLISGPVSGGS
MNPARSLGPALVSWKFDDHLWIYLVAPTIGAIAGVFVYRILRYKVGLVNRVKPEKEVILYIRL*

*Oleur*SIP1;1a Oleur061Scf4272g01002.1

>Oleur061Scf4272:214508..224653 (+ strand) class=gene length=10146

>*Oleur*SIP1;1a

ATGGGTGCTATAAAGGCAGCGGTAGCCGATGGGGTGTTGACTTTTATGTGGGTTTTTTGCGCATCGACTTTGGGC
GCACTCACTTTTGTGGTGGCTTCTGCTCTCGGAGTATCCCAAGGACTACCCACTCTTCTCGTCACTACTGTCCTT
GTTTTTGTACTGCTGTTCGTGTTCGGCATCATCGGCGATTTCTTGGGCGGCGCCACCTTCAATCCCACTGCAATC
GCTGCTTTCTATGCCGCTGGTATTGGAGGCGCCGATTCTCTCTTCTCTGCTGCCCTCCGCTTTCCTGCCCAGGTA
TGAATCATGTGATTAGTCAGATATTGATCATGTTTCTTGAGAGTATGTAATCCTACTTGCTTGATTCAAATGTAG
ATTAGTATGTGAATATTGTCAGTCGATGATATGTTTGTGAAAATCTAGATTGCTTAGGGCTAGATCTATATAATG
GAATTTAAGATTTTAAAAAAAAGGAGGTCTCCCTTGACAGATGATATAGTTGAGAAAACTAACAGATTTGAACTG
AACGACCAAATTTAAATAAAAGGCACACATGAATAATGAGTTTGCAAATAATAAACTTTGGTGACTAACACGAAA
ATTTTACCACAATTTAAGTGTATACTATGCAGGGAATGAAAATGCTAAAGTTATTACTTTTATCCTTGACAGGCG
GCTGGTGCAGTAGGTGGTGCTCTTGCAATTATGGAGGTTATGCCAATGCAGTACAAACACATGCTTGGGGGTCCT
TCATTGAAGGTTGACCTGCACACTGGAGCTATTGCTGAGGGCGTTTTGACTTTCACAATTACCTTTGCTGTCCTT
CTCATAGTCCTTAGGGGTCCAAGCAACCCACTAGTGAAGAATTGGTTGCTTGCCATGGCAACTGTTTCGGCAGTC
GTTGCCGGTTCAAGTTATACTGGACCTTCGATGAATCCTGCCAATGTAAGTGACATATCTCTTTTCCATTCTATC
GCTAGATTCTGAAATTATATGATGATCTATGTCTTATTGTGGAATACCCTAATCCTTAAAAATTGCTGAGTAATC
AAAAAATTAAAGAAACAAAATAACATGAGGTGAGAGTTTCTCTTTCCCATAGCACATAAGGACAACGTATCTGCT
GAACCTTTCTTCTAATTTTTTTTTCACATCCCAAGCCAACCTGGAATAGAGTAGTTACTTTTCTCAATATGGTA
CCCGACCGTGTTTCTTTAAAATAAAAAATGTTTATATGTAATGTGATTGAGCTTAGACATCTGAAGCACAATTTA
GAGTACTGCTCGCATTTGGCTCCCATATTTCCATTTAAGAGGTCCACGAGTCGCATCATTATTGTCCTGTTGTGG
GATGTCAAATTATCCTCAACTACCAAGTTTCTCTTTCCGTCTATGTGCCACCTTGTGATTGATCGATTAAGTGCT
GTTACCCTAATTAGGACCCACAGCAAAACAGTAGCCCTTTCCTTTACATTGACCTTAAGTGGGTTTGGAAGTTCC
ATCTATCGTAAACTTCAGATCACTTTCCACATGATTGGATTTTTCTCCAACCTTGGAATAACCAATCATTTTCGT
ATTGAGTCCTGAAAACTCCAGCTTGATCATTAACTTTCAGATTCAAAGAATATAGTTTTAGCTTTTGCTCATTTT
GACCATTATGGTCTGGTAGTGGCATTGATTTCCAGACTCAGCCATTACTTGGGAGCATTTGTTGAAATAGCATCT
GCTGTAGGAATAAGGACAATGTCTTGGGAAAGTCAATTTTGGAGAGATGAAAAGCTTGCTGGTACATCAGTTATA

TAACCGGTTGTAGAAAGTTGCATTGGAATTGAATAGCGCATGCTGAGCACGCAATAGAATGCATGCTTCAATTAA
TTAGTAGAGAAATGATAAAATTACATATGAAGAAGAATGATGAAGACTTTAAAATTTTCAGCTCAAGCTTCTAAT
ACTTAGGTTTCTTCCAACCGAAGCTACTATTTATTCACTTTGGTGCGTGGGACTTGGGCACCTATATCCAACTCA
AGTTCAATGTGTATCTTTTTCAAGTTTCACGCTATTAAACTAATGAAGTTTTCTGGGGAAAGTGTGTTAAAATTC
TGTCTGGAAGTAAACTACTGCAGAGTTTTCTTGAAAAAGTAATCTGTCTTTCTCAACTATTTTTGTGGCCATAAA
TTAGGTTCATTAATGGATTAATTATGTCATGACCATTTCGCTAATAATCTACTACACAATTGCTGAACAATTGAA
ATATGCCCGACGCAATGAAGGAATTGTGAGAATAAAAAGTTTGAGACTTGAGATGAACCAAAAAGGAAGTTTCTC
ACTTCACCACAGTGCTAGCCTGAATAAGGATGGAGATTAGAGAATAATCACAAGCAAATTAAAGGGCTTAGGATT
AAATTAAATCCAAGCTATCCAATACTTAGTCATATTAGTCAGACAAAATGTCTGATTTTTATACATGCATTTGGA
ATAAAATATGAATTTTTAGTTTTCATTTTTTTTTGAGTTCCTATTTTTTTTCCCACATGTCAAAACTTTATTATA
TTGAAAAACATAAATAGGTATCTAAAACTAATAAGTAATAATTTATAATGAGTTAGTATTTAATCCAATTAGGAA
ACCTTCTATTTTTTTGTATGTGCTTAGTTAAAAATAAAATATCCAAATATAAATACCCTCTAAAACTCAATTAAA
TCATCACTTGGCAATTCTGAGTAATTAACCACCAATGGTTTGGCCAATGACCTTAGGACGAACAAAAGTATCTAA
GCTCTAATGAGATCCCCTGAATACTGGGCAAATTATGTTGAAGTAACATTATTGTATCTTGTCCATAACCTACCA
ATAACTTTGAATGCCCTTATGGAATGAATATCCTCGACCCATAGAAATGGAAAATTATCAACCTTTTAGGTTACA
TTTTGACATTCCAATCTTCCTTGATTCACTAATTCGAACCATGTGTGTAACGCTTCGAGATCTTTTTCATCTTCA
TTATTTTATTCTTTTATAAAAATAGGCTCAAGGTGTTACTAACATCTTCTTTGTCTAATTTAAAAAGAAATATCA
GTTTCATTAAGTGTTGAAAATGCATAAAAATAACTATTAAATAATGTGCACAAAATTCAATATGAAGATGTTCTT
TAAATAAAAGTTTGACAATTATCTTTTTCAGAAAATTTACTTGGCGATCCCATAAGCTACACTCGGTCAATCTTA
TATGCCTCCAAACGACCCGTTCTCATCTGAAAGGTATTGTAAAAGTGTATTTTGAAAACATTCAGTAAGAATACG
AGCACATTTTTATTAACACGATATAAATATAAAACTGACATAATATATCGACATTTCTTAGATTTAGAGTTCAAG
GAATTTATTAAGAATTCCCATATCCCCTAAGATTTAACATTTTTGACTAAACAGTCCATCCCGTAGGATTTAACA
TTTTTGACCTAACGGTCCATCTAGAAGGATTTAACATAGAGTTCGACTGAAGGCCTCATAGCATTTTATGCCAAA
AATTATACAAACATCAAGGAATAAGAATTTACCATTTTGCACGATGTATTCTTACCTCAATGATACCAAAACAAA
ATATGAAAATATGGTTTGTAGAATAGAAAAATAAATTATTTCTAATAGATTGTGTGTATGTGAATTTATAGGGAG
TGCACCCTTTTTTTCTGGGAATTATAATCTAGTCAGATCACCTATATATATATATATATATATATATATATATAT
ATATATATATATATATATATATAGATTTAATAGGCTCGTGATATTCTTTTCAATTTCCAACGTACCAATATGAATTA
AATGAAAGTTTTGATTTATTATTTTATTACTATTTATATTTTGCTTATAAATAAAATATAAAATCTAGAATGCTT
TCTTTATAACAAAATATCTCGGGTATAACTAGTTTAAAAATTACTTTTTCTTGATTCCAAATTATATTATTTAAT
ACAAGCGATAAAACGAGTATAATATTTATATTTTATCAACTTCTAAAATACGTAAATAAATATTCGACCCAAATA
AGATTTACGATAAATTCTAAGTACACATGAGCAAAATAAATCATCAATTTTTGGACTTTTAGTGTAACGACAAAT
CTAAACATGAATTGTTTCATTTGCTAAGTGACCCATAAAAAGTTCGTGGTGTTACAATGTGACCACCAATATTGA
TGCAAGATAAAATAAGGAGAAGGGGAAAGGGAAAGGGAAAGGGAAAGGGAAAGGGAGAGAAGAGGAGAAAGGAGG
CGGAAAGATAAGAAGGGGGGAGAAAATAGAGAGAAATAGGAGAGAACATCAAGTCATTTCATAATCAAAGATTAT
TTCCAATTACATTGCATAGACCCTTTATATAGGGTTTGAACTAATTATATTTGAAGTAAGAAAATACTCAAAATA
AATAACTAATTATGTTTCTTAAAATAAACTTTAGAATGAAAACTAAATATACTAAAATCCATCCTAATTCTCCAT
CATTTCTCCTAAGCTGAGATAACTCGACTCCAGCAAAATAAATTGCAACTTCTTACCCAAGGAGGGCAATGTTTC
TTCTTCACCAAATAATGGACCCATTCACATATCAATAATTGAACCTGAAAAACATTCACAAACTTCCAATTTGAC
CAAAGGGCTCATAACCAAATAGCAATAAGGAACTTTAGGAAGCCACAACTTGTGCAACTCATTTACAAAAACTGT
TACAAGATCCACTTGAAGGGCACAAGGTACTAGCACAATCGACTAGCTTGCAGAAATGAATAAATGCAACAATGG
TGGCTCGAATGTACCCTGACAAGGCAACAATTCTGTTATATTGAAAATAAAACCCTCATTTAAGTGTGATGGCCA
ATCTAATAGATTAAATTCAAGTGAGAAATTTAATCGTTTCTCTACATGTGTAAGAAAAGTGTTTGAACGAACAAT
GACATTAGATAATGTGGTAACCCGAGAAGCATCACAAAGTAAAGGTAAAACAATATCAAAGTCAACGATAGAGTC
ATACAACAGATTAGGCAAGATCAATTTTGGCACGTAGTCGTGGATAGGTGGATTGTTTGACTCAGGTGACAATAA
TATAGATAATTCCCCATCCTCGATATTAGAATATTCAATTGACTCAACGATAGAAACCTCAATTTCTTCTTCAAG
AGCAATACAACTAGACTCAAGAGATGATTGTTGGAAATGGGTGGTAGACCTAGCTTATGACACGCTTCGCGAAGA
TGGAAGGATAAGTTCCATAGGTTGTCCTGTTGGTAAGCTTTGTCTAAGGATTGGAATGTAGGAATATCATTAGGC
AAAGTTTCAGGATTCAAACCATTGATAAACCTAGATGTTGTGATGTAGAGAGCCTTATAAATATAATAATTGTTC
AATAACTCTTCGAAGTGGGCAAAATATGCACTAGCAGTGGACGTACCTTGCCTAAAGTGCAAAAAGTATTGATTT
AACAATTGAGTTCGATAATTTACATATGCATATTTATTACTTAGGACTTGTTTCATCTCATCCCACAAGGTGACA
CTGCGTTGGCCTAGACGATTATGCAAATATGATCTCCAATAAATTTTAGTAGAATCGACCGATGATATATATACTAG
CATGACAAATTTTACATGCATCACTCAAATTGTGATAGAAAAAGAATTTTTCAAGATTATCAAGCCAATCAAGAA
CTGAATCAGATCCAGTGAGACATTAAAGTCCTATAGGTGGATTGCAACTTGCTGAGGAGAAGAATGTACCTTTTT
TGACAAAGGTATTAGACGGGGAAAATTGCGCGGATAAGTGCAAAGTGAAGGTATATGGTTGTGGTAGTGACCTAA
GGATGTGTTGTGGGCGTAACTGTTGTTACATGAAGTGTATGTAGTGTACGATGACATTTTTTTTTTTTTTTTTTTT
TTTTATTATTATAAAAATGAGGTTTGATGGGCTACGAACACCGAAATGAAAAGCAAAGCGTTCAAATAACAAGAT
GATATACCTTACAAAAGTCCAATCAAGCCGCTGGTTGAGCACCAATTTTAAGTTCCAATCTGACAGATTATACAC
TTTATTGTAAAATTAACTGCTAGCTCACGAGAGACACAAGTTGTGGCGGTGCAATTTAGTTCTGCTGCCCAATAA
TCTGAAAAATAAACTATAAGCCAGTATGTGGAATTAACCGCAAGTTGCTATAAACACTCTTGTTGGTTCGTCTAT
GTAAAAGGATGAAGCTTCTGCAGCTCAAATGTGCTCTGAAATTGAAAGAAAACTTAGTAAAACATGTGAGCTTTT
GATATAGATTGAGTGATTTTGCAGTGACCAAGCGAATTGTTGAGATGGAAACTATAGTGTAGATTAGGAATAATT
TGGATGGGAGAAAAAATCCAAAACTAGTTGGGGATCATTGTTAATTGAAATATTAGGGAGGATTGTGAGAGTGTGA
AGGAAAAGTGTTGAAGAATAGAGAGATACAGATTTGTGCAGTGACAGCGATGCGTGTTGGTGATGGAAGTGGGCC

GGCACCGAGGAATCAGGCAGTCATGGCTACAGATGTTCTGGTATTTGGTGTGTATAGGATGGACTTTTATGTGAT
GGGTACTATACGTGTATTTTAGAATTTGATGTGGGCTGGGTATGTGTTGTGTTGCTTGTGTTTTGGATAGGTGGA
TGATAATATTTGGTTAGTGGGTTGGGTTAGGGTAATTGGTTATGGGTTTCATTGGGCCATTGGATGTTGGCGAAT
GTGTGTATATGTATATGGGTTAGTGGGTTGGTGTAACAAATTAGGGTTTGGGGAAATTATCTAGTGGGCCAGGTT
GGTATGGGCTTGGTCTTGCTTGATGTATAGGCTATTGGGTTCGTATAGGTGGGTAATATAGGGTTTCTTCGGTTG
AAAGCAACCAAATTGGCCTGATCAAAAATAAATTGTACTATTTTTTTCAGAAACTGATGAAAATACTAAAATTAA
AAATTTCACGGTGTAAACAGTATCCACTAATAGTACAATGAGCAATAACTTCTTCTTTCTTTCTTTTTTTTTTTT
TTTTTTTGGTTGATGCCTGCTGCCACCTTTTTTGTGGTTTAAAGTAGGTTTTGGCCGGATATGTTGGCGGCTATG
GTTTGATTGTTGAATGATGAAACGGGTGGTTTAAATGTGGAAGTGAAATTTGTCGGTCAAGGGAAAGAAATTGAG
CTCTACTACCAAATTGATGTAGGATACAATAAGGAGAAGGGGAAAGGGGAGAGAAAAAAAAGAAATGAGGGGGGA
AAAGAGAAGGGGGAGAGAATAGAGAGAAATAGGGAGAGTATCAATTTATTTCATAATCAAAGGTTATTTCCAATT
ACATTGCATAGACCCTTTATGTAGGGTTTGAATTAATTATAATTGAAGTAAGAAAATACTCTAAATAAATCACTA
ATTGAATCCATCCTAATTCTCCATCAGATATGTATCCATATTTGAAATCCAATATCTAATATCTGTTAACTAAAA
TACAATAATCATATATTGAATGATTATACGCATGATGTCTGTTGTGAGCCTATGTTTGACTCATGTTTATAAACA
CTGAAATAATTTTTTTCCTCACATTTCATGGACCTACCAAACTACTTATTTATCCACCACTCATTTGTATCTAT
TTATGGTTTAAACCATTTATAAACAATTTTCATTCACGCTTTAAGAGATATCTGGTGCCTATTGACAATATTTAA
TTCCATAACCATAAAGATGTTTCACTCAACTTATTGAGGGTATTGGCCGTAAGTAAGATCTCTTCTGTTGATATT
TCAAAGTAATAAATCTTATGATTTGAATCTATTTTTCTTTTTAAGATTAAAGACATACATGCTTTAGTAGTTTGA
AGAAAACCAATCTAACTGATGCAATTGTATATGAATTTTCTGTCTGGGTCTTGTTCAACGCATCGAACCCCATGT
ACACACTGCATGTCAAACCCAAAATCTCATTTTGGCAACCAAGATAAATCGCCCCATCCCGTAGAGGTTGACGTG
CTACAATTTATGATGAGTTGTCATAACTCAACCTAACTGTGATCTGGTAATAAAGTTACATGGAACTCTTGATTC
TATCTTCTTAGCCAGTGTACATATCCCAAGTCATGTACAGTTTAAATTCAATGGCATTGTAAATTTCATCTAACA
AATTGCCTGCTGTAAATGTATATTGGTAATGAATTCAAAATCTACTAAAGGTAAAGAACGATTGCAGTACTTAAC
TAGTTGTATACACAAGAAGTTAACCTGATAATGCATGTAGGATCTACTGTTTGTTGTATTGGTAAGAGATTCTTG
GAGTTTCCCACGTAGGTCAAGAGGAGGTAGTTGTGTATGCCATGCAATACTGTGTCGAGAGCTGCTCAGTTTCAG
TGTCTATCTTAACTTTTAAGGAAGGAAGAAAAACCACTTCACAGATTCATGACACAGATGTTCTTTCCTCTTTGG
AAAACTCTGTCTGGATAGAACTTCCACGTGCTGGTGCCTCTGCTGTTTTTACCTTTTCTCCTCCTCTTTTTTCCC
TGTATTCTAAAGTAGGTCCTTTCAAAAAGTATTTGATGCTTTTGTTTGTATCTTTTTTCTTTGGAACTATTTGGT
CCCCTTTCTTCATCTGTTACTATGGTATGCCTTTAAGAAGATGGAATCCAATAACATTCCATCAATTGTGCCGGT
TCTTTTAACGAATCCCAATTCCACTTTGGGAGAAATTAAAATTAATTTGTATAACTGCTGAATTAATTGGATTGT
TGCAAGTGGAATGAATGGGATGCGGTCTGTTACATAGCAAATTAACTAATTTCGAATTTATTCAATTTGAGCTTA
CTAATTAGCTGATCTAAACTTCTATTTAGCTACATATTTGAATCACTAGGCTCAAATCTTGAACTCAAATAAGTG
CCGAGAACTTTTGAAGTATAACTAGCAGTTTAAAAATTGAATAAATTTATCAAAACTGTTCTTTAGATATTGGCA
GTTTTGCCGAGCAAAGAATTGTTTAATATAATAAAAATTGAGGTACCGTTTCAGGACATTATAAGAGTTCTGATC
TGTAACTGCTGTCAAATTTTTCTTTATGTGTTTTCTTGAATATGGAAAGTCAAAGTTGTTCTTTGAAGTCTTATA
TTTGCATTTAGTTTCTTGATTCTCTTTCCTCCTAATCTGACTTCTCTATCCTCAAAATTTTCAGAAGATAATTTT
GCTTAGTGATAGTTTCGATTCCTGTGTGTAATTCCATAGCTACTACGGCAAGGATTGCTGTGGTTTAGTTTAGTT
TCTTGCAAGTGTGCTAACATCCAAACATCTTGTGTAATTCCATCATTCTTAACCTGTTCTTATTATTTTTATTAG
CTATTTTCCGCAAAGCTTGTGTTAAAATTGGATGCTAATCGTGTCTAATATATTGCAGGCATTCGGGTGGGCATA
TGTATATAATAAGCACAACACATGGGAGCAATTCTATGTTTACTGGATCTGTCCATTCACTGGAGCAATATTGGC
TGCGTGGACCTTCCGCTTTCTATTCCCGCCCCCAACAAAGCAGGACAAGGCGAAGAAAGCC**TGA**

>*Oleur*SIP1;1a

ATGGGTGCTATAAAGGCAGCGGTAGCCGATGGGGTGTTGACTTTTATGTGGGTTTTTTGCGCATCGACTTTGGGC
GCACTCACTTTTGTGGTGGCTTCTGCTCTCGGAGTATCCCAAGGACTACCCACTCTTCTCGTCACTACTGTCCTT
GTTTTTGTACTGCTGTTCGTGTTCGGCATCATCGGCGATTTCTTGGGCGGCGCCACCTTCAATCCCACTGCAATC
GCTGCTTTCTATGCCGCTGGTATTGGAGGCGCCGATTCTCTCTTCTCTGCTGCCCTCCGCTTTCCTGCCCAGGCG
GCTGGTGCAGTAGGTGGTGCTCTTGCAATTATGGAGGTTATGCCAATGCAGTACAAACACATGCTTGGGGGTCCT
TCATTGAAGGTTGACCTGCACACTGGAGCTATTGCTGAGGGCGTTTTGACTTTCACAATTACCTTTGCTGTCCTT
CTCATAGTCCTTAGGGGTCCAAGCAACCCACTAGTGAAGAATTGGTTGCTTGCCATGGCAACTGTTTCGGCAGTC
GTTGCCGGTTCAAGTTATACTGGACCTTCGATGAATCCTGCCAATGCATTCGGGTGGGCATATGTATATAATAAG
CACAACACATGGGAGCAATTCTATGTTTACTGGATCTGTCCATTCACTGGAGCAATATTGGCTGCGTGGACCTTC
CGCTTTCTATTCCCGCCCCCAACAAAGCAGGACAAGGCGAAGAAAGCC**TGA**

>*Oleur*SIP1;1a

MGAIKAAVADGVLTFMWVFCASTLGALTFVVASALGVSQGLPTLLVTTVLVFVLLFVFGIIGDFLGGATFNPTAI
AAFYAAGIGGADSLFSAALRFPAQAAGAVGGALAIMEVMPMQYKHMLGGPSLKVDLHTGAIAEGVLTFTITFAVL
LIVLRGPSNPLVKNWLLAMATVSAVVAGSSYTGPSMNPANAFGWAYVYNKHNTWEQFYVYWICPFTGAILAAWTF
RFLFPPPTKQDKAKKA*

*Oleur*SIP1;1b Oleur061Scf2367g00017.1

>Oleur061Scf2367:30689..40838 (- strand) class=gene length=10150

>*Oleur*SIP1;1b

ATGGGTGCTATAAAGGCAGCGGTAGCCGATGGGGTGTTGACTTTTATGTGGGTTTTTTGCGCATCGACTTTGGGC
GCACTCACTTTTGTGGTGGCTTCTGCTCTCGGAGTATCCCAAGGACTACCCACTCTTCTCGTCACTACTGTCCTT
GTTTTTGTACTGCTGTTCGTGTTCGGCATCATCGGCGATTTCTTGGGCGGCGCCACCTTCAATCCCACTGCAATC
GCTGCTTTCTATGCCGCTGGTATTGGAGGCGCCGATTCTCTCTTCTCTGCTGCCCTCCGCTTTCCTGCCCAGGTA
TGAATCATGTGATTAGTCAGATATTGATCATGTTTCTTGAGAGTATGTAATCCTACTTGCTTGATTCAAATGTAG
ATTAGTATGTGAATATTGTCAGTCGATGATATGTTTGTGAAAATCTAGATTGCTTAGGGCTAGATCTATATAATG
GAATTTAAGATTTTAAAAAAAAGGAGGTCTCCCTTGACAGATGATATAGTTGAGAAAACTAACAGATTTGAACTG
AACGACCAAATTTAAATAAAAGGCACACATGAATAATGAGTTTGCAAATAATAAACTTTGGTGACTTACACGAAA
ATTTTACCACAATTTAAGTGTATACTATGCAGGGAATGAAAATGCTAAAGTTATTACTTTTATCCTTGACAGGCG
GCTGGTGCAGTAGGTGGTGCTCTTGCAATTATGGAGGTTATGCCAATGCAGTACAAACACATGCTTGGGGGTCCT
TCATTGAAGGTTGACCTGCACACTGGAGCTATTGCTGAGGGCGTTTTGACTTTCACAATTACCTTTGCTGTCCTT
CTCATAGTCCTTAGGGGTCCAAGCAACCCACTAGTGAAGAATTGGTTGCTTGCCATGGCAACTGTTTCGGCAGTC
GTTGCCGGTTCAAGTTATACTGGACCTTCGATGAATCCTGCCAATGTAAGTGACATATCTCTTTTCCATTCTATC
GCTAGATTCTGAAATTATATGATGATCTATGTCTTATTGTGGAATACCCTAATCCTTAAAAATTGCTGAGTAATC
AAAAAATTAAAGAAACAAAATAACATGAGGTGAGAGTTTCTCTTTCCCATAGCACATAAGGACAACGTATCTGCT
GAACCTTTCTTCTAATTTTTTTTTTCACATCCCAAGCCAACCTGGAATAGAGTAGTTACTTTTCTCAATATGGTA
CCCGACCGTGTTTCTTTAAAATAAAAAAATGTTTATATGTAATGTGATTGAGCTTAGACATCTGAAGCACAATTT
AGAGTACTGCTCGCATTTGGCTCCCATATTTCCATTTAAGAGGTCCACGAGTCGCATCATTATTGTCCTGTTGTG
GGATGTCAAATTATCCTCAACTACCAAGTTTCTCTTTCCGTCTATGTGCCACCTTGTGATTGATCGATTAAGTGC
TGTTACCCTAATTAGGACCCACAGCAAAACAGTAACCCTTTCCTTTACATTGACCTTAAGTGGGTTTGGAAGTTC
CATCTATCGTAAACTTCAGATCACTTTCCACATGATTGGATTTTTCTCCAACCTTGGAATAACCAATCATTTTCG
TATTGAGTCCTGAAAACTCCAGCTTGATCATTAACTTTCAGATTCAAAGAATATAGTTTTAGCTTTTGCTCATTT
TGACCATTATGGTCTGGTAGTGGCATTGATTTCCAGACTCAGCCATTACTTGGGAGCATTTGTTGAAATAGCATC
TGCTGTAGGAATAAGGACAATGTCTTGGGAAAGTCAATTTTGGAGAGATGAAAAGCTTGCTGGTACATCAGTTAT
ATAACCGGTTGTAGAAAGTTGCATTGGAATTGAATAGCGCATGCTGAGCACGCAATAGAATGCATGCTTCAATTA
ATTAGTAGAGAAATGATAAAATTACATATGAAGAAGAATGATGAAGACTTTAAAATTTTCAGCTCAAGCTTCTAA
TACTTAGGTTTCTTCCAACCGAAGCTACTATTTATTCACTTTGGTGCGTGGGACTTGGGCACCTATATCCAACTC
AAGTTCAATGTGTATCTTTTTCAAGTTTCACGCTATTAAACTAATGAAGTTTTCTGGGGAAAGTGTGTTAAAATT
CTGTCTGGAAGTAAACTACTGCAGAGTTTTCTTGAAAAAGTAATCTGTCTTTCTCAACTATTTTTGTGGCCATAA
ATTAGGTTCATTAATGGATTAATTATGTCATGACCATTTCGCTAATAATCTACTACACAATTGCTGAACAATTGA
AATATGCCCGACGCAATGAAGGAATTGTGAGAATAAAAAGTTTGAGACTTGAGATGAACCAAAAAGGAAGTTTCT
CACTTCACCACAGTGCTAGCCTGAATAAGGATGGAGATTAGAGAATAATCACAAGCAAATTAAAGGGCTTAGGAT
TAAATTAAATCCAAGCTATCCAATACTTAGTCATATTAGTCAGACAAAATGTCTGATTTTTATACATGCATTTGG
AATAAAATATGAATTTTTAGTTTTCATTTTTTTTTGAGTTCCTATTTTTTTTCCCACATGTCAAAACTTTATTAT
ATTGAAAAACATAAATAGGTATCTAAAACTAATAAGTAATAATTTATAATGAGTTAGTATTTAATCCAATTAGGA
AACCTTCTATTTTTTTGTATGTGCTTAGTTAAAAATAAAATATCCAAATATAAATACCCTCTAAAACTCAATTAA
ATCATCACTTGGCAATTCTGAGTAATTAACCACCAATGGTTTGGCCAATGACCTTAGGACGAACAAAAGTATCTA
AGCTCTAATGAGATCCCCTGAATACTGGGCAAATTATGTTGAAGTAACATTATTGTATCTTGTCCATAACCTACC
AATAACTTTGAATGCCCTTATGGAATGAATATCCTCGACCCATAGAAATGGAAAATTATCAACCTTTTAGGTTAC
ATTTTGACATTCCAATCTTCCTTGATTCACTAATTCGAACCATGTGTGTAACGCTTCGAGATCTTTTTCATCTTC
ATTATTTTATTCTTTTATAAAAATAGGCTCAAGGTGTTACTAACATCTTCTTTGTCTAATTTAAAAAGAAATATC
AGTTTCATTAAGTGTTGAAAATGCATAAAAATAACTATTAAATAATGTGCACAAAATTCAATATGAAGATGTTCT
TTAAATAAAAGTTTGACAATTATCTTTTTCAGAAAATTTACTTGGCGATCCCATAAGCTACACTCGGTCAATCTT
ATATGCCTCCAAACGACCCGTTCTCATCTGAAAGGTATTGTAAAAGTGTATTTTGAAAACATTCAGTAAGAATAC
GAGCACATTTTTATTAACACGATATAAATATAAAACTGACATAATATATCGACATTTCTTAGATTTAGAGTTCAA
GGAATTTATTAAGAATTCCCATATCCCCTAAGATTTAACATTTTTGACTAAACAGTCCATCCCGTAGGATTTAAC
ATTTTTGACCTAACGGTCCATCTAGAAGGATTTAACATAGAGTTCGACTGAAGGCCTCATAGCATTTTATGCCAA
AAATTATACAAACATCAAGGAATAAGAATTTACCATTTTGCACGATGTATTCTTACCTCAATGATACCAAAACAA
AATATGAAAATATGGTTTGTAGAATAGAAAAATAAATTATTTCTAATAGATTGTGTGTATGTGAATTTATAGGGA
GTGCACCCTTTTTTTCTGGGAATTATAATCTAGTCAGATCACACACACATATATATATATATATATATATATATA
TATATATTAGATTTAATAGGCTCGTGATATTCTTTTCAATTTCCAACGTACCAATATGAATTAAATGAAAGTTTT
GATTTATTATTTTATTACTATTTATATTTTGCTTATAAACAAAATATAAAATCTAGAATGCTTTCTTTATAACAA
AATATCTCGGGTATAACTAGTTTAAAAATTACTTTTTCTTGATTCCAAATTATATTATTTAATACAAGCGATAAA
ACGAGTATAATATTTATATTTTATCAACTTCTAAAATACGTAAATAAATATTCGACCCAAATAAGATTTACGATA
AATTCTAAGTACACATGAGCAAAATAAATCATCAATTTTTGGACTTTTAGTGTAACGACAAATCTAAACATGAAT
TGTTTCATTTGCTAAGTGACCCATAAAAAGTTCGTGGTGTTACAATGTGACCACCAATATTGATGCAAGATAAAA
TAAGGAGAAGGGGAAGGGGAAGGGGAAAGGGAAAGGGAAAGGGAAAGGGAAAGGGAGAGAAGAGGAGAAAGGAGG
CGGAAAGATAAGAAGGGGGGAGAAAATAGAGAGAAATAGGAGAGAACATCAAGTCATTTCATAATCAAAGATTAT
TTCCAATTACATTGCATAGACCCTTTATATAGGGTTTGAACTAATTATATTTGAAGTAAGAAAATACTCAAAATA
AATCACTAATTATGTTTCTTAAAATAAACTTTAGAATGAAAACTAAATATACTAAAATCCATCCTAATTCTCCAT
CATTTCTCCTAAGCTGAGATAACTCGACTCCAGCAAAATAAATTGCAACTTCTTACCCAAGGAGGGCAATGTTTC
TTCTTCACCAAATAATGGACCCATTCACATATCAATAATTGAACCTGAAAAACATTCACAAACTTCCAATTTGAC

CAAAGGGCTCATAACCAAATAGCAATAAGGAACTTTAGGAAGCCACAACTTGTGCAACTCATTTACAAAAACTGT
TACAAGATCCACTTGAAGGGCACAAGGTACTAGCACAATCGACTAGCTTGCAGAAATGAATAAATGCAACAATGG
TGGCTCGAATGTACCCTGACAAGGCAACAATTCTGTTATATTGAAAATAAAACCCTCATTTAAGTGTGATGGCCA
ATCTAATAGATTAAATTCAAGTGAGAAATTTAATCGTTTCTCTACATGTGTAAGAAAAGTGTTTGAACGAACAAT
GACATTAGATAATGTGGTAACCCGAGAAGCATCACAAAGTAAAGGTAAAACAATATCAAAGTCAACGATAGAGTC
ATACAACAGATTAGGCAAGATCAATTTTGGCACGTAGTCGTGGATAGGTGGATTGTTTGACTCAGGTGACAATAA
TATAGATAATTCCCCATCCTCGATATTAGAATATTCAATTGACTCAACGATAGAAACCTCAATTTCTTCTTCAAG
AGCAATACAACTAGACTCAAGAGATGATTGTTGGAAATGGGTGGTAGACCTAGCTTATGACACGCTTCGCGAAGA
TGGAAGGATAAGTTCCATAGGTTGTCCTGTTGGTAAGCTTTGTCTAAGGATTGGAATGTAGGAATATCATTAGGC
AAAGTTTCAGGATTCAAACCATTGATAAACCTAGATGTTGTGATGTAGAGAGCCTTATAAATATAATAATTGTTC
AATAACTCTTCGAAGTGGGCAAAATATGCACTAGCAGTGGACGTACCTTGCCTAAAGTGCAAAAAGTATTGATTT
AACAATTGAGTTCGATAATTTACATATGCATATTTATTACTTAGGACTTGTTTCATCTCATCCCACAAGGTGACA
CTGCGTTGGCCTAGACGATTATGCAAATATGATCTCCAATAAATTTTAGTAGAATCGACCGATGATATATACTAG
CATGACAAATTTTACATGCATCACTCAAATTGTGATAGAAAAAGAATTTTTCAAGATTATCAAGCCAATCAAGAA
CTGAATCAGATCCAGTGAGACATTAAAGTCCTATAGGTGGATTGCAACTTGCTGAGGAGAAGAATGTACCTTTTT
TGACAAAGGTATTAGACGGGGAAAATTGCGCGGATAAGTGCAAAGTGAAGGTATATGGTTGTGGTAGTGACCTAA
GGATGTGTTGTGGGCGTAACTGTTGTTACATGAAGTGTATGTAGTGTACGATGACATTTTTTTTTTTTTTTTTT
TTTTATTATTATAAAAATGAGGTTTGATGGGCTACGAACACCGAAATGAAAAGCAAAGCGTTCAAATAACAAGAT
GATATACCTTACAAAAGTCCAATCAAGCCGCTGGTTGAGCACCAATTTTAAGTTCCAATCTGACAGATTATACAC
TTTATTGTAAAATTAACTGCTAGCTCACGAGAGACACAAGTTGTGGCGGTGCAATTTAGTTCTGCTGCCCAATAA
TCTGAAAAATAAACTATAAGCCAGTATGTGGAATTAACCGCAAGTTGCTATAAACACTCTTGTTGGTTCGTCTAT
GTAAAAGGATGAAGCTTCTGCAGCTCAAATGTGCTCTGAAATTGAAAGAAAACTTAGTAAAACATGTGAGCTTTT
GATATAGATTGAGTGATTTTGCAGTGACCAAGCGAATTGTTGAGATGGAAACTATAGTGTAGATTAGGAATAATT
TGGATGGGAGAAAAATCCAAAACTAGTTGGGGATCATTGTTAATTGAAATATTAGGGAGGATTGTGAGAGTGTGA
AGGAAAAGTGTTGAAGAATAGAGAGATACAGATTTGTGCAGTGACAGCGATGCGTGTTGGTGATGGAAGTGGGCC
GGCACCGAGGAATCAGGCAGTCATGGCTACAGATGTTCTGGTATTTGGTGTGTATAGGATGGACTTTTATGTGAT
GGGTACTATACGTGTATTTTAGAATTTGATGTGGGCTGGGTATGTGTTGTGTTGCTTGTGTTTTGGATAGGTGGA
TGATAATATTTGGTTAGTGGGTTGGGTTAGGGTAATTGGTTATGGGTTTCATTGGGCCATTGGATGTTGGCGAAT
GTGTGTATATGTATATGGGTTAGTGGGTTGGTGTAACAAATTAGGGTTTGGGGAAATTATCTAGTGGGCCAGGTT
GGTATGGGCTTGGTCTTGCTTGATGTATAGGCTATTGGGTTCGTATAGGTGGGTAATATAGGGTTTCTTCGGTTG
AAAGCAACCAAATTGGCCTGATCAAAAATAAATTGTACTATTTTTTTCAGAAACTGATGAAAATACTAAAATTAA
AAATTTCACGGTGTAAACAGTATCCACTAATAGTACAATGAGCAATAACTTCTTCTTTCTTTTTTTTTTTTTTT
TTTTTTGGTTGATGCCTGCCGCCACCTTTTTTGTGGTTTAAAGTAGGTTTTGGCCGGATATGTTGGCGGCTATGG
TTTGATTGTTGAATGATGAAACGGGTGGTTTAAATGTGGAAGTGAAATTTGTCGGTCAAGGGAAAGAAATTGAGC
TCTACTACCAAATTGATGTAGGATACAATAAGGAGAAGGGGAAAGGGGAGAGAAAAAAAAGAAATGAGGGGGGAA
AAGAGAAGGGGGAGAGAATAGAGAGAAATAGGGAGAGTATCAATTTATTTCATAATCAAAGGTTATTTCCAATTA
CATTGCATAGACCCTTTATGTAGGGTTTGAATTAATTATAATTGAAGTAAGAAAATACTCTAAATAAATCACTAA
TTGAATCCATCCTAATTCTCCATCAGATATGTATCCATATTTGAAATCCAATATCTAATATCTGTTAACTAAAAT
ACAATAATCATATATTGAATGATTATACGCATGATGTCTGTTGTGAGCCTATGTTTGACTCATGTTTATAAACAC
TGAAATAATTTTTTTCCTCACATTTCATGGGACCTACCAAACTACTTATTTATCCACCACTCATTTGTATCTATT
TATGGTTTAAACCATTTATAAACAATTTTCATTCACGCTTTAAGAGATATCTGGTGCCTATTGACAATATTTAAT
TCCATAACCATAAAGATGTTTCACTCAACTTATTGAGGGTATTGGCCGTAAGTAAGATCTCTTCTGTTGATATTT
CAAAGTAATAAATCTTATGATTTGAATCTATTTTTCTTTTTAAGATTAAAGACATACATGCTTTAGTAGTTTGAA
GAAAACCAATCTAACTGATGCAATTGTATATGAATTTTCTGTCTGGGTCTTGTTCAACGCATCGAACCCCATGTA
CACACTGCATGTCAAACCCAAAATCTCATTTTGGCAACCAAGATAAATCGCCCCATCCCGTAGAGGTTGACGTGC
TACAATTTATGATGAGTTGTCATAACTCAACCTAACTGTGATCTGGTAATAAAGTTACATGGAACTCTTGATTCT
ATCTTCTTAGCCAGTGTACATATCCCAAGTCATGTACAGTTTAAATTCAATGGCATTGTAAATTTCATCTAACAA
ATTGCCTGCTGTAAATGTATATTGGTAATGAATTAAATTCAAAATCTACTAAAGGTAAAGAACGATTGCAGTACT
TAACTAGTTGTATACACAAGAAGTTAACCTGATAATGCATGTAGGATCTACTGTTTGTTGTATTGGTAAGAGATT
CTTGGAGTTTCCCACGTAGGTCAAGAGGAGGTAGTTGTGTATGCCATGCAATACTGTGTCGAGAGCTGCTCAGTT
TCAGTGTCTATCTTAACTTTTAAGGAAGGAAGAAAAACCACTTCACAGATTCATGACACAGATGTTCTTTCCTCT
TTGGAAAACTCTGTCTGGATAGAACTTCCACGTGCTGGTGCCTCTGCTGTTTTTACCTTTTCTCCTCCTCTTTTT
TCCCTGTATTCTAAAGTAGGTCCTTTCAAAAAGTATTTGATGCTTTTGTTTGTATCTTTTTTCTTTGGAACTATT
TGGTCCCCTTTCTTCATCTGTTACTATGGTATGCCTTTAAGAAGATGGAATCCAATAACATTCCATCAATTGTGC
CGGTTCTTTTAACGAATCCCAATTCCACTTTGGGAGAAATTAAAATTAATTTGTATAACTGCTGAATTAATTGGA
TTGTTGCAAGTGGAATGAATGGGATGCGGTCTGTTACATAGCAAATTAACTAATTTCGAATTTATTCAATTTGAG
CTTACTAATTAGCTGATCTAAACTTCTATTTAGCTACATATTTGAATCACTAGGCTCAAATCTTGAACTCAAATA
AGTGCCGAGAACTTTTGAAGTATAACTAGCAGTTTAAAAATTGAATAAATTTATCAAAACTGTTCTTTAGATATT
GGCAGTTTTGCCGAGCAAAGAATTGTTTAATATAATAAAAATTGAGGTACCGTTTCAGGACATTATAAGAGTTCT
GATCTGTAACTGCTGTCAAATTTTTCTTTATGTGTTTTCTTGAATATGGAAAGTCAAAGTTGTTCTTTGAAGTCT
TATATTTGCATTTAGTTTCTTGATTCTCTTTCCTCCTAATCTGACTTCTCTATCCTCAAAATTTTCAGAAGATAA
TTTTGCTTAGTGATAGTTTCGATTCCTGTGTGTAATTCCATAGCTACTACGGCAAGGATTGCTGTGGTTTAGTTT
AGTTTCTTGCAAGTGTGCTAACATCCAAACATCTTGTGTAATTCCATCATTCTTAACCTGTTCTTATTATTTTTA

TTAGCTATTTTCCGCAAAGCTTGTGTTAAAATTGGATGCTAATCGTGTCTAATATATTGCAGGCATTCGGGTGGG
CATATGTATATAATAAGCACAACACATGGGAGCAATTCTATGTTTACTGGATCTGTCCATTCACTGGAGCAATAT
TGGCTGCGTGGACCTTCCGCTTTCTATTCCCGCCCCCAACAAAGCAGGACAAGGCGAAGAAAGCC**TGA**

>*Oleur*SIP1;1b

ATGGGTGCTATAAAGGCAGCGGTAGCCGATGGGGTGTTGACTTTTATGTGGGTTTTTTGCGCATCGACTTTGGGC
GCACTCACTTTTGTGGTGGCTTCTGCTCTCGGAGTATCCCAAGGACTACCCACTCTTCTCGTCACTACTGTCCTT
GTTTTTGTACTGCTGTTCGTGTTCGGCATCATCGGCGATTTCTTGGGCGGCGCCACCTTCAATCCCACTGCAATC
GCTGCTTTCTATGCCGCTGGTATTGGAGGCGCCGATTCTCTCTTCTCTGCTGCCCTCCGCTTTCCTGCCCAGGCG
GCTGGTGCAGTAGGTGGTGCTCTTGCAATTATGGAGGTTATGCCAATGCAGTACAAACACATGCTTGGGGGTCCT
TCATTGAAGGTTGACCTGCACACTGGAGCTATTGCTGAGGGCGTTTTGACTTTCACAATTACCTTTGCTGTCCTT
CTCATAGTCCTTAGGGGTCCAAGCAACCCACTAGTGAAGAATTGGTTGCTTGCCATGGCAACTGTTTCGGCAGTC
GTTGCCGGTTCAAGTTATACTGGACCTTCGATGAATCCTGCCAATGCATTCGGGTGGGCATATGTATATAATAAG
CACAACACATGGGAGCAATTCTATGTTTACTGGATCTGTCCATTCACTGGAGCAATATTGGCTGCGTGGACCTTC
CGCTTTCTATTCCCGCCCCCAACAAAGCAGGACAAGGCGAAGAAAGCC**TGA**

>*Oleur*SIP1;1b

MGAIKAAVADGVLTFMWVFCASTLGALTFVVASALGVSQGLPTLLVTTVLVFVLLFVFGIIGDFLGGATFNPTAI
AAFYAAGIGGADSLFSAALRFPAQAAGAVGGALAIMEVMPMQYKHMLGGPSLKVDLHTGAIAEGVLTFTITFAVL
LIVLRGPSNPLVKNWLLAMATVSAVVAGSSYTGPSMNPANAFGWAYVYNKHNTWEQFYVYWICPFTGAILAAWTF
RFLFPPPTKQDKAKKA*

*Oleur*SIP1;3 Oleur061Scf0342g12031.1

>Oleur061Scf0342:1258679..1261622 (- strand) class=gene length=2944

>*Oleur*SIP1;3

ATGGGTGCTATTAAAGCAGCGGTAGCCGATGGGGTGCTGACTTTTATGTGGGTTTTTTGCACATCGACGTTGGGC
GCACTCACTTTTGTGGTGGCTTCTGCTCTCGGAGTATCCCAAGGCCTACCCACTCTTTTCATCACTACTGTTCTC
GTTTTTCTCCTGTTGATCTTGTTCGGCATCATCGGCGATGTATTGGGCGGCGCTACCTTCAATCCCACTGCCCTC
GCCGCCTTCTATGCCGCTGGTATTGGGGGCGCTGATTCGCTAGTCTCTGCTGCCCTCCGATTTCCTGCCCAGGCA
TGACTTGCCTGCTCTTCATTTTTAGATTAGTCAGATATTAAACACAGTATGCAATCATCCTACTGACTTGATTCA
AACGGAGATTAATAAGTGGAAATTGTCAGTTGATGATATGTTTGTGAAAATCTAGATTGCTCAGGGCTAGACTAG
ATATAATGAAACTCGAGATAATAAGAATAAAAAATGAACTTCCAATTTTTTTAAAAAATTCACAGATTTTAACTG
AACGACCAAATTACAACAAAAGGCAAACTTGAATAATGAGTTTGCACAAAATAAAGTTCGGTGACTAATGCGAAG
CTTTGCCATAAGTTAAGTAAAAGTTATGCAAGGAATGAGAATGGTAAAGCCGTTACTTGTATCCTTGACAGGCAG
CTGGTGCAGTAGGTGGGGCTCTTGCAATTATGGAGGTTATGCCGATGCAGTACAAACACATGCTTGGGGGTCCTT
CTCTGAAGGTTGACTTGCACACTGGAGCCATTGCTGAGGGAGTTTTGACGTTCACGATCTCTTTTGCTGTCCTTC
TCATCGTCCTTAGGGGTCCAAGCAGCCCACTTGTGAAGAATTGGTTGCTTGCCATGTCAACTGTTTCGCTAGTCG
TTGCCGGTTCAAGTTATACTGGACCTTCGATGAATCCTGCAAATGTAAGTGGTGCATCTCTTTTCCGTTCTTTTG
CTAGATTTTGAAATAGTATAAGATGATATATCTATGTCTTATTGTAGAATATTCTAAATCCTTTAAAATTGCAGA
CTAAAGAAACAAAATAACATGAGGTGAGAGTTTCTCCTTCCTATAGCACAAAATGAAAAGATATCTGCCAAACAT
TTTGTATTTAACTTTTTTATTTCATATCCCAAATGCCAACTTAGAATATAGAAGTTACTTTTCCCATTGATGGTA
CATGATTGTGTATCTTTTGCAATCAAAGTGAGATTGGTAATGTGATCGAGCTTTGACATTCGGAGCACAATCAGA
GTACTGTTCTCTTGACTTAATTTTTGGATTAGGAATGTGATTTGGTCCCCATATTTACATTTATGGGGTTCACTC
ATCCCATCATTATTGTCCCGTTATGGGGTGTCAAATTATCGAGATCCTCAACTACCAGGTTTCTATATGAGAACT
TTAGGAAAGAATGCTACTAGATTCTATTTTCCTCTATGCACCAAACTGTAACTGATCCTCAAATGCCGTCACCCT
AATTAGGACTTTGTAGCACACAGTAATACTTTCCTTTACACTCACGTTAAAATTTGTTTTGGAAGATCCATCTTT
CTTTAACTTCTGATGTCTTTCGACGTGATTGGATTTTTCTCCTTCCTTTGGAAGAACCAATCATTTTCTTATTGA
GACCCGAAAACACCAGCTTGATTAATTAACTTTCCAATTCAAAGAGCTTTAACTTTGCTCCTTTTTCTTCAATCA
TGTATGTCTTCAAAATAATGAATGGGACACAGTCTGTTACAAAGCCAAATAATAATTTAGACTTTGATTCATGTT
TAGCTTATCTTAAAGTCTCTTTAGTTACATTTGCAAATTGCTAATCTCAAATCGTGAACTAAAAATGTGCGGAGA
ACTTTTGAAGCATAACTTTAACATTTTTAAGCAGTAATTAATTAGTCAAAACTGCTCTCTAGATATTGGCTATAT
GCCAAATTTTTAGGTAGAGTTTTAGGACATTATAAGAGTTTTGATCTGTAACTGCGGTCAAATTGTTCTTTCTGT
GTATTTTCTTGAATCTGGAAAGTCAAAATTGTTCTTTGAAGTCTTATATTTGCATCTTATTTCTTGATTCTCTTC
TTCCCTAATCTGACTTATCTATTCTCAAAATTGTTAGAAGATATTTTGCAAAGTGATAGTTTTGAGCATGTGCAG
TCAGAAGTTTCTTAGTGTAGAATACTTCTGATTTTCATGCCCTTCAATTAAGTTGCCAAACATAGTTTTCGGATT
CTAGTTTTGACATTTGTAATGTGGTTTACATGGGACGGCTTAGCCTCACAGAGAAAAGCTACAACCCTGGAGTTC
AAATCACTTTTAGTTCCATAACTACTGCAAAATGGATTGGCTGTGCTTTAGTCTGGTTTCTTAGAACAAGTTTGC
TAATATCAGAACATCTTGTGCAATTCTTTCTTTCTTAATTTGATATTTCTCGCAAAACTTGTGTTATAACTGGCT
TTCAATTGTGCCTAATATCATTGCAGGCGTTTGGGTGGGCTTATGTACATAATAAGCACAACACATGGGAGCAAT
TCTATGTTTATTGGATCTGTCCCTTCGTAGGAGCAATATTGGCTGCGTGGACATTTCGCTTTCTATTCCCGCCCC
CATCAAATCAGCACAAGGCGAAGAAAGCC**TGA**

>*Oleur*SIP1;3

ATGGGTGCTATTAAAGCAGCGGTAGCCGATGGGGTGCTGACTTTTATGTGGGTTTTTTGCACATCGACGTTGGGC
GCACTCACTTTTGTGGTGGCTTCTGCTCTCGGAGTATCCCAAGGCCTACCCACTCTTTTCATCACTACTGTTCTC
GTTTTTCTCCTGTTGATCTTGTTCGGCATCATCGGCGATGTATTGGGCGGCGCTACCTTCAATCCCACTGCCCTC
GCCGCCTTCTATGCCGCTGGTATTGGGGGCGCTGATTCGCTAGTTATGCCGATGCAGTACAAACACATGCTTGGG
GGTCCTTCTCTGAAGGTTGACTTGCACACTGGAGCCATTGCTGAGGGAGTTTTGACGTTCACGATCTCTTTTGCT
GTCCTTCTCATCGTCCTTAGGGGTCCAAGCAGCCCACTTGTGAAGAATTGGTTGCTTGCCATGTCAACTGTTTCG
CTAGTCGTTGCCGGTTCAAGTTATACTGGACCTTCGATGAATCCTGCAAATGCGTTTGGGTGGGCTTATGTACAT
AATAAGCACAACACATGGGAGCAATTCTATGTTTATTGGATCTGTCCCTTCGTAGGAGCAATATTGGCTGCGTGG
ACATTTCGCTTTCTATTCCCGCCCCCATCAAATCAGCACAAGGCGAAGAAAGCC**TGA**

>*Oleur*SIP1;3
MGAIKAAVADGVLTFMWVFCTSTLGALTFVVASALGVSQGLPTLFITTVLVFLLLILFGIIGDVLGGATFNPTAL
AAFYAAGIGGADSLVMPMQYKHMLGGPSLKVDLHTGAIAEGVLTFTISFAVLLIVLRGPSSPLVKNWLLAMSTVS
LVVAGSSYTGPSMNPANAFGWAYVHNKHNTWEQFYVYWICPFVGAILAAWTFRFLFPPPSNQHKAKKA*

*Oleur*SIP1;2 Oleur061Scf0259g06030.1

>Oleur061Scf0259:705305..709156 (+ strand) class=gene length=3852

>*Oleur*SIP1;2
ATGAGGGCAATTAAGGTAGCAATCGCCGATGCGATAGTGACGTGCTTGTGGGTTTTTTGTGCATCGTCGTTGGGT
GCACTCACTTTCGTTGTGGCTTCTGCTTTTGGGGTTAGCCAAGGCCTGCCTACGCTTTTGATCACCACTTTTCTC
CTTTTTGTTCTTTTGTTTGTATTCGGATTTATTGGAGATGCGCTGGGAGGCGCCACCTTCAATCCCACAGGTCCC
GCCGCCTTTTATGCCGCCGGCGTTGGAGGCACTGAGTCTCTTGTCAGCGCCGCCGTCCGCTTTCCTGCTCAGGTG
ACCTCTTCTTGTACGATTCTCATGTTTCGTTTAATGGTCTATGTTATTGCTTTGCAAGATTATAAGTATTAGAAA
GCAAAAAATTCAGTCGTTTTGTCATAGCCGTAGGCCATACGTCAATAATTCAATGCTGAATCATTTGGATTCCAT
TGGTGACGGGACATAGAATGTGGATTTTGAAGAGAGATTCAACCACTACAAAAAGATCGATTTTTCGGGATCCTT
TCTTAAAAGGGGAGGAAAAGGTGTAAAATCAGACCAATTCGTACAATTCCTTTCTGGATATTCCTTAGTGTTCCG
GCTATTCATGAAATGTGAGATGATGATTGTAACTGAGTCCAAATCCTTGACATCAAGCCGAGTGACTTTTGATCC
TTGCAAGATGATTCTTGGAAATTGTATGACTCGTTTTTCTTCTGTACATGACGAAGATTAGTAGATCATCTTGGA
TTGTATGCAGGGCCTGGCTCTGAGATCTTGGATGTCCAGTGTGAAATTTCAAAATGGGCCCCTTTTTTCATAAAT
ATTGTGCCCTTTAACTAACATAATAATAAAAATTTTAAATTTTAAAATTACTATATATAACAAAAAATATTTTGG
TGCCCCTACAACGTTGAGGCCTTGTGCGGTTGCCCCACCTGCGCACCCTTAGGGCCACCTCTGATTGTATGACTC
GCTTGTACTTTGTAAATGACAAAGATTAGAGGATTGCTGGCATTGAAATATAGTTTAGATTTGCAATATTTAAAA
TTTGCATTGCTTAAGTTCAGGCAGTTGAACACTTGATTCATGCTAATCTGATCATGAAGAACAATGAGGTTCCAG
AAGAGCTAAACGTGCTGTGAAAGAGTGGAATGAATGGATGGAAAAAACCAATAGACTCTTTTTGTAAAAATCGTA
AATTATTTTGTGGTGTCCTTGACAGGCAGCTGGTGCAGTAGGTGGTGCACTTGGGATTCTAGAGGTTATGCCAGT
GCAATACAAGCACATGCTAGATTGTCCTTCTTTGAAAGTCGATTTGAATACAGGAGCAATTGCTGAGGGAGTATT
GACCTTCATAATTACCTTTGCTGTCCTTTACATTGTCCTGAGGGGTCCTACCAACCCATTTGTGAAGAATTGGTT
GCTAGCTATGTCAACTGTTGCCTTAGTGGTTGCAGGTTCGAGTTATACTGGACCTTCCATGAATCCTGCAAATGT
AAGTCCCATCTCTCTTTACGTTCCCTTTTTAGATAAATCATGGGCAATTTATGGTAAATTCTTGTCTTACATGCA
ATGAATTTTCCTTCAATCAAGTTATAAGAAACTTTAGAGTGTTTCTTTGCTTAGCATAATAGCAGAAATATCCAC
ACCATTATTTTTGTTTATTTATATCTTTCAAATTCCTTGTTAGATTTTATTATATTCTTTCCTAATCATGCTTCT
CTCTGCATCAAAATTCTGATTAAGATGCCAGGTATAGATTGATCTTTGACATCTATAAGCACAACGAGTGCATGC
TTGGTGTAAACATAGATGGAAGCTAAATAACAAAATTTAAGTTTCTTTTTATTCCTAAATAGTGGTCAATGTTAA
CAATTCTTTCTAGCAAACATGCCATTAGATTATTGTCCATCAATGCTGACTGAACATATGGGATTCCACGTCTTT
CTTGCTACTTAGGTCGGAATGTAAGCTAGTAATGTTTTCGGCTCTTGTACTTCACCATCTATGGTGTTGCTTCGT
TTAGCAGCACGACTATTGTATTGCCCGCCCACAACACTGTTTCACAGTTGATCTATGATGGATCTGTTAGCCGCG
CGCATAATTTTAAGGATCTCAATTCATTGCACTGTCATGTCTTAATATTGCAATCACTCTTAATAAGCCCCCGTT
TGGCCCCCATCCCTTCAAATTTTAATGGTTGGAATAATTTTTTTTGAAAAATGTGTTGTTGAAAAAGAATGAAAA
GTTTTTGATTTGCATGTAATATTATTTTATGGAAGATTATGTAGTTGAAAATAAGAAAAAAAAGAAGAAGGAGAA
GGAGAATGGGAGCCAAACGGGGCCATAGTAGCAAGCTTCTCAACCACTCAATCTTTGCCATTTTCCCTAATTGCA
AGCTTGCAAATGACCTATTAATTCCTGCAATCCAAAACTTGAACTAGAGTTCTAAAATATGATTTTCTTTAAATT
AACTTTACCTTTATGGTGCGTTTGATTGCAAGTATGAAATTTGCATAGAAAGAAAATAAATTATAGGTAATTTAA
TTGCCCTAGAACTTATTTGTATGAAAATTATCAATTAAATGTGTTTGATTGATTTAATTTTTAGCCTTAAAAATA
TTATACTTGTCCAATTTTTATTTGTGGTTAGATGAGAGAGTGGATGAATAAGAAAGTGAAAGGAGAAAATTCCGT
TGGAAAATAAAATCCTACTACACCTAAAGAAAACATTTTCCTTGATTTTGAGGAAAATATGGTCACATGACCAAA
AGTAGGAAAATGTTTTTCATGGAAAAAAGTCAATCAAACAGTATAAAAGTAGGAATTTGGGTGAAAAAATACATT
TTTCATGGAAAATATTGACAATCAAACGGACCCTTAGTTTCGATACATTCATAAGTTTGAATTTAATATCACTA
TAACAAACGAGCTAATCAGTCATTCAACTTTGCAACATATGTGCTGATTTTCAGAAAGTTTCCATACATTGTTGT

TTTTATCACTTGTTATTGTTCTCCTATCCCTTCTGCTTATTTTCGTGCTCTTTATCTATAAATGGATCATATGCT
CCTTATCATACATCTTAATCTATATCTAATATCTTTGCAGGCCTTTGGCTGGGCATATGTAAACAATACGCACAA
AACATGGGAGCAATTCTACGTCTACTGGATCTGCCCCTTCATTGGAGCAATATTAGCTGCGTGGGTTTTCCGTTT
TCTATTTCCGCCTCCTCCAGTCAAGAAGCAGAAGAAAGCC**TGA**

>*Oleur*SIP1;2

ATGAGGGCAATTAAGGTAGCAATCGCCGATGCGATAGTGACGTGCTTGTGGGTTTTTGTGCATCGTCGTTGGGT
GCACTCACTTTCGTTGTGGCTTCTGCTTTTGGGGTTAGCCAAGGCCTGCCTACGCTTTTGATCACCACTTTTCTC
CTTTTTGTTCTTTTGTTTGTATTCGGATTTATTGGAGATGCGCTGGGAGGCGCCACCTTCAATCCCACAGGTCCC
GCCGCCTTTTATGCCGCCGGCGTTGGAGGCACTGAGTCTCTTGTCAGCGCCGCCGTCCGCTTTCCTGCTCAGGCA
GCTGGTGCAGTAGGTGGTGCACTTGGGATTCTAGAGGTTATGCCAGTGCAATACAAGCACATGCTAGATTGTCCT
TCTTTGAAAGTCGATTTGAATACAGGAGCAATTGCTGAGGGAGTATTGACCTTCATAATTACCTTTGCTGTCCTT
TACATTGTCCTGAGGGGTCCTACCAACCCATTTGTGAAGAATTGGTTGCTAGCTATGTCAACTGTTGCCTTAGTG
GTTGCAGGTTCGAGTTATACTGGACCTTCCATGAATCCTGCAAATGCCTTTGGCTGGGCATATGTAAACAATACG
CACAAAACATGGGAGCAATTCTACGTCTACTGGATCTGCCCCTTCATTGGAGCAATATTAGCTGCGTGGGTTTTC
CGTTTTCTATTTCCGCCTCCTCCAGTCAAGAAGCAGAAGAAAGCC**TGA**

>*Oleur*SIP1;2

MRAIKVAIADAIVTCLWVFCASSLGALTFVVASAFGVSQGLPTLLITTFLLFVLLFVFGFIGDALGGATFNPTGP
AAFYAAGVGGTESLVSAAVRFPAQAAGAVGGALGILEVMPVQYKHMLDCPSLKVDLNTGAIAEGVLTFIITFAVL
YIVLRGPTNPFVKNWLLAMSTVALVVAGSSYTGPSMNPANAFGWAYVNNTHKTWEQFYVYWICPFIGAILAAWVF
RFLFPPPPVKKQKKA*

*Oleur*SIP2;1 Oleur061Scf0186g06043.1

>Oleur061Scf0186:651945..657348 (- strand) class=gene length=5404

>*Oleur*SIP2;1

ATGGCTGCCGTAGGAGGAAGGCTGCTGCTAGTGTCGGATTTCTTTATGTCGTTCATGTGGGTTTGGTCTAGCGTG
CTTATCAAGATTTTTGTGTATAAAATATTGGGCTATGGCACCCATGATTTGAAGGGTGAAATTCTGAGGTATGGA
ATCTCCATTGTCAACATGTTCTTCTTTGCATACTTGGCCAAAGCCACCAAGGGTGGGGCCTACAACCCCCTCACC
ATCTTGTCCTCCGCCATTTCTGGGGATTTCAGCAATTTCCTCTTCACTGTCGGGGCCAGAATTCCTATCCAGGTT
CGCTGAGTGCAAATGTTTTAACTCTTGTTTCTCTAATTATTATGCTATTTTTCGTTTTAGGAAACATTGTGGCTC
GATAAATTTGGCGATTGATGCGTGTAATTATGTGCATAAATTGACGTTTTGTTTGGTGTGACATTGAATTAGAGA
AATTCGATGATGATTCTTCTCTTTATTTTCAGGAATGCTTGCTTTTGGGTTGCTCGTAAATTCCGATTAGGGCAT
GCAATAAATCTCTGAGTTTAAGCCAAAAATTTCTATTTCTTTTTTGGAAATTGAATTGATCTAATATCTAAAACC
TTATACAGTGTAGGTAGGTAAAAGTAATTTTATTACTGAAAGAGCTTAAGAAATTTAGGGATTTGACCTTGAAAG
AAGATTATTCTCAGTTAATGTTAGTTAAGCTAAATCTTGTGGTAAAAAAACAAGTCTCATTGCCATATATTCAAG
ACTTATTCTCAGTTAATAATTTATGAAAACTGATGTGTGTTTTGTTGATTGATTTGTATGAACTAAAGATTGCAA
TATGATTAAGAGGTCTAACTTACAATACTGTACTTGTGGAAGAAGTGGGACAGTGGAGATATCTTGTTAGTTTGG
CAAATCCTTTGCTGATAATTCTGTAGAACCGAGCTCCATGTACAGGACTTTCTAATTCTGTATCTACGTGTAGGA
CTTTCTTATTCGAGGTTTCTAATTTGGAATTGTTCTCAACTAGTCGAATTCTTTTCACCACATACTTTTTGTTCC
ATTATCTTCTTATTTAGCCCCGTTTGATGGGCTTTTCTTCAAAACTCTTTTATCTATATACTCATTTTCATCCAC
ACATTCACACAATAAAATATTATGTACATAAACTCAAAACTTTTCCTTACTTTTTAATTATACATTCTCCAAAAA
TTCACTATTCAAAGCATTATTTTTCCCAAATAATGGCCATCAATCAGGGGCTTAGAGAACATCCCTTTTCCCGTG
TTGTCACTGCTGCAGTGCTTCTAGTTTCTTTATCTCTCAGAGAGGTTATGAAGAGTAGTAATTTTCAAAACTCAT
AGTTTATTCATAATCGTCTAATAGTCGTCTGGAAATAAAAGACTCGTCATGCTTTTTAAATGACTTCTGGAAGTT
GAGTTAAAGATGATTTAGGCTCTGTTTGTTTTCCATTCTTTTTCCATTAAAATGAAAAATATGACCGTTATCCAT
CACATCAAGGCACCAAATTTGAATTTCTATCTCTTGTGTGGGGTCATAACGACTATATTTTTCATTTTCATTTTT
GCAAAAATAAGAAACAAACGCCCCCTTAGTTTTTTTACCCGCGCTTACCCTTATTTTTGTTCTTCCATCTCTCTT
TTAAAGGTCTTGGGGTCTATTTATGGAGTTAGGTTCATTCTCGCGGCATTTCCTGAAATAGGACGTGGGCCACGT
CTGAATGTTGACATAGCTCGAGGTGCATTGACTGAAGGTCTTTTGACATTTGGGATTGTGATTATATCCCAAGGG
CTCTCGAGAAAAATTCCCGGAAGTTTCTTCATGAAGACTTGGATCTCCAGCGTCTCAAAGCTCACTCTTCACATA
CTCGGCTCTGATCTGACTGGAGGATGCATGAATCCAGCCTCTGTAAGTACTTAATAATCATTCTATGCATAATCT
CTGTTTACTGCCGAATTTTGTTGGATGCACACTGAGTAAAATTTTAATTGCATCTCCCAGGTAATGGGGTGGGCT
TTTGCACACGGAGAACATATAAGCAAGGAGCATTTAATTGTGTACTGGCTTGCTCCTATGGAGGCAACTTTGGCA
GCAGTGTGGGCATTTAGACTGATAGTTCAGCCCAAGAAGGACGAGACACTAAAGGCGAAGTCCGTAAAATCAGAT
TGA

>*Oleur*SIP2;1

ATGGCTGCCGTAGGAGGAAGGCTGCTGCTAGTGTCGGATTTCTTTATGTCGTTCATGTGGGTTTGGTCTAGCGTG
CTTATCAAGATTTTTGTGTATAAAATATTGGGCTATGGCACCCATGATTTGAAGGGTGAAATTCTGAGGTATGGA
ATCTCCATTGTCAACATGTTCTTCTTTGCATACTTGGCCAAAGCCACCAAGGGTGGGGCCTACAACCCCCTCACC
ATCTTGTCCTCCGCCATTTCTGGGGATTTCAGCAATTTCCTCTTCACTGTCGGGGCCAGAATTCCTATCCAGGTC

TTGGGGTCTATTTATGGAGTTAGGTTCATTCTCGCGGCATTTCCTGAAATAGGACGTGGGCCACGTCTGAATGTT
GACATAGCTCGAGGTGCATTGACTGAAGGTCTTTTGACATTTGGGATTGTGATTATATCCCAAGGGCTCTCGAGA
AAAATTCCCGGAAGTTTCTTCATGAAGACTTGGATCTCCAGCGTCTCAAAGCTCACTCTTCACATACTCGGCTCT
GATCTGACTGGAGGATGCATGAATCCAGCCTCTGTAATGGGGTGGGCTTTTGCACACGGAGAACATATAAGCAAG
GAGCATTTAATTGTGTACTGGCTTGCTCCTATGGAGGCAACTTTGGCAGCAGTGTGGGCATTTAGACTGATAGTT
CAGCCCAAGAAGGACGAGACACTAAAGGCGAAGTCCGTAAAATCAGAT**TGA**

>*Oleur*SIP2;1

MAAVGGRLLLVSDFFMSFMWVWSSVLIKIFVYKILGYGTHDLKGEILRYGISIVNMFFFAYLAKATKGGAYNPLT
ILSSAISGDFSNFLFTVGARIPIQVLGSIYGVRFILAAFPEIGRGPRLNVDIARGALTEGLLTFGIVIISQGLSR
KIPGSFFMKTWISSVSKLTLHILGSDLTGGCMNPASVMGWAFAHGEHISKEHLIVYWLAPMEATLAAVWAFRLIV
QPKKDETLKAKSVKSD*

*Oleur*SIP2;2 Oleur061Scf8348g02016.1

>Oleur061Scf8348 Oleur061Scf8348:245169..250426 (- strand) class=gene length=5258

>*Oleur*SIP2;2

ATGGCGGCCGTAAGAGGTAGGCGGCTGCTGGTGTCGGATTTCTTGATGTCTTTCATGTGGGTTTGGTCCAGTGTG
CTCATTAAGATTTTTGTTTATAAAATATTGGATTATGGCTCCCATGATATGAAGGGTGAAATTTTGAGGTATGGA
ATCTCCATTGTCAACATGTTCTTCTTTGCATTCTTGGCCAAAGCTACCAAGGGTGGGGCCTACAACCCCCTCACC
GTCTTGTCCTCGGCCATTTCTGGGAATTTCAGCAATTTCCTCTTCACTGTCGGGGCCAGAATTCCTGTCCAGGTT
CGCTAGATACAATTGTTTTAAAATCTTGTTCTTTAATTATCATGTCACCGTTTGTTTTAGGAAATATTGACAGTT
TCTTTTGTGAGGGACATTGAATTAGAGAAGTTAGATTGATGGTTCTTCTCTTTATTTAGGACTTCATGTTTTTG
GGTTAATCGTAAATTTGATTAGGGCCTGCATCTTAAATCAATCTGGGAGTTTCAGTAAATACGTTCTATTGATTA
TTTGGCAAGTGAATTGATAGATAATCTGAAACCATATACATGGTAGGCTTAGTGAAAGCAGCAATTTCAATGATT
GTCACATGTTATTGGAATATCTTAAGAATCATTCAAAAATCGAAGAAATGATCAAAACAAAATGATTTTTGAATT
GTATTCCAGAAAAATTTTATTCACAAATAAAACTATATCTCGTTATCTTGGCAAATTATTTGAGGCTAATTATAT
ATCTGCATGTAGGACTTTCTTTCTTATCCAAGATTCCCCCAATTCGACATCTTTCTCAAATATCAACTATAAAAA
ATTCTTTCCCCACTTACTTTGTGTTCCAATATCTATACTTATTTAGAGAGTTTCCCTTTTTCCCATTTTGTTAAT
GCTGACACGATGAATGGATCGTTGTTCTCCAGAAAAACCCTCAGACAGGTTGTGAAGAGGAATGACATGGTGTTC
TTAAAATAAGTGTTCCAAAACTCATAGTTTATTCATAATCATCAGCTGGAAGTTGAGTTAAGATGGTTTAGTTGT
TTACTGGAGCTTATCCTTGTTCTTCCATTTCTTTTTCAAAGGTCTTGGGGTCTATTTATGGGGTCAGGCTCATTC
TTGCGGCATTTCCAGAAATAGGACGTGGGCCACGTCTGAATGTTGACATAACTCGAGGTGCATTGACTGAAGGGT
TTTTGACGTTTGGAATTGTAATTATATCCCAAGGGCTCTCGAGAAAACTTCCTGGAAGTTTCTTCATGAAGACTT
GGATCTCAAGCGTCTCGAAGCTCACTCTTCACATGCTTGGCTCTGATCTGACTGGAGGATGCATGAATCCGGCAT
CTGTAAGTACTTATATTCAGTCTAAATGTAATCTATGCTTACTGCGGAATTGTGTTGGATACATTACACTTGAAT
GAACATCGAGTAAATTCTAATTGCGTCCCTCAGGTAATGGGGTGGGCTTTTACACGCGGGGAACATATAAGCAAG
GAACATTTAATTGTATACTGGCTTGCTCCTATGGAGGCGACTTTGGCAGCAGTGTGGGCATTTAGAGTTGTAGTT
CGACCCGAGAATGATAAGACACCTAAGGTGAAGTCCGGAAAATCAGAA**TGA**

>*Oleur*SIP2;2

ATGGCGGCCGTAAGAGGTAGGCGGCTGCTGGTGTCGGATTTCTTGATGTCTTTCATGTGGGTTTGGTCCAGTGTG
CTCATTAAGATTTTTGTTTATAAAATATTGGATTATGGCTCCCATGATATGAAGGGTGAAATTTTGAGGTATGGA
ATCTCCATTGTCAACATGTTCTTCTTTGCATTCTTGGCCAAAGCTACCAAGGGTGGGGCCTACAACCCCCTCACC
GTCTTGTCCTCGGCCATTTCTGGGAATTTCAGCAATTTCCTCTTCACTGTCGGGGCCAGAATTCCTGTCCAGGTC
TTGGGGTCTATTTATGGGGTCAGGCTCATTCTTGCGGCATTTCCAGAAATAGGACGTGGGCCACGTCTGAATGTT
GACATAACTCGAGGTGCATTGACTGAAGGGTTTTTGACGTTTGGAATTGTAATTATATCCCAAGGGCTCTCGAGA
AAACTTCCTGGAAGTTTCTTCATGAAGACTTGGATCTCAAGCGTCTCGAAGCTCACTCTTCACATGCTTGGCTCT
GATCTGACTGGAGGATGCATGAATCCGGCATCTGTAATGGGGTGGGCTTTTACACGCGGGGAACATATAAGCAAG
GAACATTTAATTGTATACTGGCTTGCTCCTATGGAGGCGACTTTGGCAGCAGTGTGGGCATTTAGAGTTGTAGTT
CGACCCGAGAATGATAAGACACCTAAGGTGAAGTCCGGAAAATCAGAA**TGA**

>*Oleur*SIP2;2

MAAVRGRRLLVSDFLMSFMWVWSSVLIKIFVYKILDYGSHDMKGEILRYGISIVNMFFFAFLAKATKGGAYNPLT
VLSSAISGNFSNFLFTVGARIPVQVLGSIYGVRLILAAFPEIGRGPRLNVDITRGALTEGFLTFGIVIISQGLSR
KLPGSFFMKTWISSVSKLTLHMLGSDLTGGCMNPASVMGWAFTRGEHISKEHLIVYWLAPMEATLAAVWAFRVVV
RPENDKTPKVKSGKSE*
