# Supplementary material for: Genome Wild Analysis and Molecular Understanding of the Aquaporin Diversity in Olive Trees (Olea Europaea L.)
Source: Int J Mol Sci. 2020 Jun 11;21(11):4183. doi: 10.3390/ijms21114183 (PMC7312470; doi:10.3390/ijms21114183)
Supplement: Supplementary file 1 [file ijms-21-04183-s001.zip › ijms-821530_Supplementary_files/Figure S3_Faize_FraexAQP_protein_sequences.pdf]

Figure S3. Deduced protein sequences of *Fraex*AQPs from *Fraxinus excelsior*. Sequences are listed according to their subfamilies. Pseudogenes and/or incomplete sequences are listed at the end of the document.

>FraexPIP1;1 FRAEX38873_v2_000016990.1
MESKEEDVRVGANKFNERQPIGTAAQSQDKDYKEPPPAPLFEPGELSSWSFYRAGIAEFIATFLFLYITVLTVMG
VSKSDSKCKTVGIQGIAWAFGGMIFALVYCTAGISGGHINPAVTFGLFLARKLSLTRAVFYMVMQCLGAICGAGV
VKGFGKTLYMTKGGGANVVAPGYTKGDGLGAEIIGTFVLVYTVFSATDAKRSARDSHVPILAPLPIGFAVFLVHL
ATIPITGTGINPARSLGAAIIYNKDHAWDDHWIFWVGPFIGAALAALYHVVVIRAIPFKKISDDTKASSICGH
>FraexPIP1;2 FRAEX38873_v2_000126390.1
MESKEEDVRVGANKFNERQPIGTAAQSQDKDYKEPPPAPLFEPGELSSWSFYRAGIAEFIATFLFLYITILTVMG
VSKSDSKCSTVGIQGIAWAFGGMIFALVYCTAGISGGHINPAVTFGLFLARKLSLTRAIFYMVMQCLGAICGAGV
VKGFGKTLYMTKGGGANVVAPGYTKGDGLGAEIVGTFVLVYTVFSATDAKRSARDSHVPILAPLPIGFAVFLVHL
ATIPITGTGINPARSLGAAIIYNKDHAWDDHWIFWVGPFVGAALAALYHVVVIRAIPFKNK
>FraexPIP1;3 FRAEX38873_v2_000177250.2 (corrected)
MAESKEEDVKLGANKFTERQPLGTAAQTDKDYKEPPPAPLFEPGELTSWSFYRAGIAEFIATFLFLYISILTVMG
VLSAKNKCASVGVQGIAWAFGGMIFVLVYCTAGISGGHINPAVTFGLFLARKLSLTRAVFYMVMQCLGAICGAGV
VKGFMKGQYGRLQGGANIVQHGYTKGDGLGAEIIGTFVLVYTVFSATDAKRNARDSHVPLLAPLPIGFAVFLVHL
ATIPITGTGINPARSLGAAIIYNKDHAWDDQWVFWVGPFIGAALAAIYHQIVIRAIPFKSRA
>FraexPIP1;4 FRAEX38873_v2_000264300.1
MAENREEDVRLGANKFAEKQPLGTAAQTDKDYKEPPPAPLFEPGELKSWSFYRAGIAEFVATFLFLYITILTVMG
VGRSQNKCASVGIQGIAWSFGGMIFALVYCTAGISGGHINPAVTFGLFLARKLSLTRAIFYIVMQCLGAICGAGV
VKGFMEGPYQRLQGGANMVQHGYTKGDGLGAEIVGTFILVYTVLSATDAKRNARDSHVPILAPLPIGFAVFLVHL
ATIPITGTGINPARSLGAAIIYNKEHAWNDHWIFWVGPFIGAALAAIYHQIVIRAIPFKSSRAC
>FraexPIP1;5 FRAEX38873_v2_000319900.1 (corrected)
MAENREEDVRLGANKFAEKQPLGTAAQTDKDYKEPPPAPLFEPGELKSWSFYRAGIAEFVATFLFLYITILTVMG
VGRSNSKCASVGIQGIAWSFGGMIFALVYCTAGISGGHINPAVTFGLFLARKLSLTRAIFYIVMQCLGAICGAGV
VKGFMEGPYQRLQGGANFMQHGYTKGDGLGAEIVGTFILVYTVFSATDAKRNARDSHVPLLAPLPIGFAVFLVHL
ATIPITGTGINPARSLGAAIIYNRDHAWDDHWIFWVGPFVGAALAAIYHQIVIRAIPFKSSQAC
>FraexPIP1;6 FRAEX38873_v2_000183090.1
MEGKEEDVRLGANKFTERQPLGTSAQSKDYKEPPPAPLFEPGELQSWSFWRAGIAEFMATFLFLYITILTVMGVS
RSGSKCASVGIQGIAWAFGGMIFALVYCTAGISGGHINPAVTFGLFLARKLSLTRAIFYIVMQCLGAICGAGVVK
GFQPSSYKANGGGANVVAHGYTKGDGLGAEIIGTFVLVYTVFSATDAKRSARDSHVPILAPLPIGFAVFLVHLAT
IPITGTGINPARSLGAAIIYNKDHAWDDHWIFWVGPFIGAALAALYHQVIIRAIPFKSGS
>FraexPIP2;1 FRAEX38873_v2_000206040.1
MTKEFESHVEPAEEYSTKDYHDPPPAPLIDFDELSSWSFYRAIIAEFVATLLFLYITVLTVIGYKSQIDPAHSGD
DCDGVGILGIAWAFGGMIFILVYCTAGISGGHINPAVTFGLFLGRKVSLIRAVLYMVAQCLGAICGVGLVKAFQK
AYFNRYGGGANELAPGYNKGVGLGAEIIGTFVLVYTVFSATDPKRNARDSHVPVLAPLPIGFAVFMVHLATIPIT
GTGINPARSFGAAVIYNKDKAWDDHWIFWVGPFVGALIAAIYHQYILRAGAIKAMGSFRSNA
>FraexPIP2;2
MAKGVGVVEHGSFTAKDYHDPPPAPLIDAEELTKWSFYRAVIAEFIATLLFLYITVLTVIGYKSQIDTTAGHGGD
QCGGVGILGIAWAFGGMIFVLVYCTAGISGGHINPAVTFGLFLARKVSMIRAVIYMVAQCLGAICGVALVKALHK
SNYKKYGGGANQLADGYSAGGGLAAEIIGTFVLVYTVFTATDPKRKARDTHVPVLAPLPIGFAVFMVHLATIPIT
GTGINPARSFGAAWIFWVGPFIGAAIAAFYHQYILRAAAIKALGSFRSNN*
>FraexPIP2;3 FRAEX38873_v2_000084870.1
MVKDVGLAEHGSFTAKDYHDPPPVPLIDAEELSKWSFYRAVIAEFIATLLFLYITVLTVIGYKSQTDTNAGHVGD
QCGGVGILGIAWAFGGMIFVLVYCTAGISGGHINPAVTFGLFLARKVSMIRAIMYMVAQCLGAICGVGLVKAFQK
SYYKKYGGGANEVADGYSNRVGLSAEIIGTFVLVYTVFSATDPKRSARDSHVPVLAPLPIGFAVFMVHLATIPIT
GTGINPARSFGAAVIYNQEKAWDDQWIFWLGPFIGAAIAAFYHQFILRAGAVKALGSFRSSSRV
>FraexPIP2;4 FRAEX38873_v2_000295860.1
MAKGVGVVEHGSFTAKDYHDPPPAPLIDAEELTKWSFYRAVIAEFIATLLFLYITVLTVIGYKSQIDTTAGHGGD
QCGGVGILGIAWAFGGMIFVLVYCTAGISGGHINPAVTFGLFLARKVSMIRAVIYMVAQCLGAICGVALVKALHK
SNYKKYGGGANQLADGYSAGGGLAAEIIGTFVLVYTVFTATDPKRKARDTHVPVLAPLPIGFAVFMVHLATIPIT
GTGINPARSLGAAVIYNQEKAWDDQWIFWVGPFIGAAIAAFYHQFILRAGAVKALGSFRSSSHV
>FraexPIP2;5 FRAEX38873_v2_000071150.1
MGKDVEVGAEYTTKDYQDPPPALLIDPEELTKWSFYRAIIAEFIATLLFLYITVLTVIGYKSQSAADECGGVGIL
GIAWAFGGMIFILVYCTAGISGGHINPAVTLGLFLARKVSLIRAIMYMVAQCLGAICGCGLVKAFQKSYYVKFGG
GANGLAEGYSNGTGLAAEIIGTFVLVYTVFSATDPKRNARDSHVPVLAPLPIGFAVFMVHLATIPITGTGINPAR
SLGAAVIYGKDEAWDDQWIFWVGPFIGAAIAAFYHQYILRAGAVKALGSFRSTAY

>FraexPIP2;6 FRAEX38873_v2_000148890.1
MTKDAGVAEHGSFTAKDYHDPPPAPLIDAKELTKWSFYRALIAEFVATLLFLYITVLTVIGYKSQIDTDLNGDQC
GGVGILGIAWAFGGMIFILVYCTAGISGGHINPAVTFGLFLARKVSLIRAVMYMVAQCLGAICGVGLVKAFQKSY
YKKYGGGANELADGISTGVGLAAEIIGTFVLVYTVFSATDPKRSARDSHVPVLAPLPIGFAVFMVHLATIPITGT
GINPARSLGAAVIYDQDKAWDDHWIFWVGPFIGAAVAAFYHQFILRAGAVKALGSFRSNASHH
>FraexPIP2;7 FRAEX38873_v2_000322410.1
MAKGIEVGDPEFTAKDYQDPPPAPLIDPEELTKWSFYRAIIAEFIATLLFLYITVLTVIGYKSQSATDECGGVGI
LGIAWAFGGMIFVLVYCTAGISGGHINPAVTFGLFLARKVSLVRAIMYMVAQCLGAICGCGLVKAFQRAYYVRYG
GGANGLEVGYSTGTGLAAEIIGTFVLVYTVFSATDPKRSARDSHVPVLAPLPIGFAVFMVHLATIPITGTGINPA
RSFGAAVIYGKDKAWDDQWIFWAGPFIGAAIAAFYHQYILRAGAVKALGSFRSSASAY
>FraexPIP2;8 FRAEX38873_v2_000236920.1
MTKEVIEEGHVHSDVKDYEDPPPAPFLDMGELMLWSFYRAIIAEFVATLLFLYVTVATVIGHKKLNAADQCDGVG
ILGIAWAFGGMIFILVYCTAGISGGHINPAVTFGLFLARKVSLIRAIMYMIAQCLGAICGVGLVKAFMKSYYNHL
GGGANSVSPGYNTGTALGAEIIGTFVLVYTVFSATDPKRSARDSHVPVLAPLPIGFAVFMVHLATIPITGTGINP
ARSFGAAVIFNNDKIWDDHWIFWVGPFVGALAAAAYHQYILRAAAIKAIGSFRSNPTN
>FraexPIP2;9 FRAEX38873_v2_000097580.1
MTKEVQVHHHGKDYVDPPPAPLLDMGELKLWSFYRALIAEFIATLLFLYVTVATVIGHKKLNAADQCDGVGLLGV
AWAFGGMIFILVYCTAGISGGHINPAVTFGLFLARKVSLIRAVMYMIAQSLGAICGVGLVKAFMKGYYNRLGGGA
NSVSPGYNTGSALGAEIIGTFVLVYTVFSATDPKRNARDSHVPVLAPLPIGFAVFMVHLATIPITGTGINPARSF
GAAVIFNNDKVWDDHWIFWVGPFVGALAAAAYHQYILRAAAIKALGSFRSNPTN
>FraexPIP2;10 FRAEX38873_v2_000121310.1
MENRIGMKKEYLDPPPVPLIGSTELTMWSFYRAVIAEFIATLLFLYITVLTVIGYKSQTDTTAGHGGNQCGGVGI
LGIAWAFGGMIFVLVYCTAGISEGHINPAVTFGLFLARKVSMIRAVIYMVAQCLGAICGVALVKALHKSNYKKYG
GGANQLADGYSAGGGLAAEIIGTFVLVYTVFTATDPKRKARDTHVPVLAPLSIGFAVFMVHLATIPITGTSINPA
RSLGPAVIYNQKKAWDDQWIFWVGPFIGAAMAALYHQCILRAKAFGSYGSSSHV
>FraexPIP2;11 FRAEX38873_v2_000347560.1
MTKDVESHVEPAGEFSAKDYHDPPPAPLIDFDELSSWSFYRAIIAEFIATLLFLYITVLTVIGYKSQIDPAHGGD
QCGGVGILGIAWAFGGMIFILVYCTAGISGGHINPAVTFGLFLGRKVSLIRAVMYMVAQCLGAICGVGFVKAFQK
SYYNQYGGGANELAPGYNKGVGLGAEIIGTFVLVYTVFSATDPKRSARDSHVPVLAPLPIGFAVFMVHLATIPIT
GTGINPARSFGAAVIYNKDKPWDDHWIFWVGPFIGAAIAAFYHQYILRAAAIKALGSFRSNA
>FraexPIP2;12 FRAEX38873_v2_000076880.1
MTKEVSEEGQVHHGGKDYVDPPPTPLLDIGELKLWSFYRALIAEFIATLLFLYVTVATVIGHKKLNNADQCDGVG
LLGIAWAFGGMIFILVYCTAGISGGHINPAVTFGLFLARKVSLFRALGYMVAQCLGAICGVGLVKAIMKSFYNDL
GGGANSVALGYNKGTALGAEIIGTFVLVYTVFSATDPKRSARDSHVPVLAPLPIGFAVFMVHLATIPITGTGINP
ARSFGAAVIFNNDKVWDDHWIFWVGPFVGALAAAIYHQYILRASNLKALGSFRSNPTN
>FraexPIP2;13 FRAEX38873_v2_000264070.1
MTKDVESHVEPAGEFSAKDYHDPPPAPLIDFDELSSWSFYRAIIAEFIATLLFLYITVLTVIGYKSQIDPAHGGD
QCGGVGILGIAWAFGGMISILVYCTTGIAGGHIKSAVTFRLFLGRKVLLIRAVTYMVAQCLGVICGVGLVKAFHK
SYYNQYGGKGNELAPGYNKGVGMGGAEIIGTFVLVYTVFSATNPRRSARDSHIPVLSRLPIGFAISMVHLATIPS
TGTGINLARSVRAAVIYNKDKPWDDHWIFWLGPFIGATIAAFYHQYILRAAAIKALGSLRSNT

>FraexXIP1;1 FRAEX38873_v2_000000210.1
MAANNWNIHGVDDEESQTGLSKIQPVPSTPMTQQLTADHDKKKQTSLTLSQMLGLNEIFSLDVWRASVGELIGSA
VLVFMIDTVVISTFETDIKMPNLLLSILIAIVVAILLLAVHPVSGGHINPVISFSAALVGLISMSRAIIYILAQC
IGAILGALALKAVVSSTIEQTFSLGGCTLTVIAPGPNGPITIGLETAQAFWLEIFCTFVFLFASIWMAYDHRQAK
RLGHVLVFSIIGLVLGLLVYISTSVTAKKGYAGAGMNPARCLGAAIVRGGHLWNGHWVFWAGPAIACVAFYLYTK
IIPRQHHHSKGYEHDFFNIMKAMLGFVDH
>FraexXIP2;1 FRAEX38873_v2_000123280.1
MAGNIGHVNVEYDEESLSDASKIQPVFSTPMPEHWTDEEKMKKKTSLSVSELLGLEDFLSLDVWRASVGELIGTA
VLVFMLDTIVISTIESDIKMPNLVLSILAAIIIAILLLAVHPVSGGHINPVISFSAALVGLISMSKAIIYIVAQC
LGAVLGALALKAVVSSTIEQTFSLGGCTLTVIAPGPNGPVMIGLETAQAIWLEIFCTFVFLFASIWMAYDQRQAK
ELGHVLVLSIVGVVLGLLVFISTTVTAKKGYAGAGMNPARCFGPAIVRGGHLWNGHWVFWVGPTIACVAFYLYTK
IIPLQHHRSKYSYDHDFFNVVKAMF
>FraexXIP3;1 FRAEX38873_v2_000228360.1
MAGDNGHVHVECDEESLSGANKIQPVFSTPMPEHWTDEEKRKKKTSLTLRELLGLEDFLSLDVWRASVGELIGTA
VLVFMLDTIVISTIETDIKMPNLVMSILAAIIVAILLLAVHPVSGGHINPAISFSAALVGLISMSKALIYIIAQC
LGAALGALALKAVVSSTIEQTFSLGGCTLTLIAPGPNGPVAIGLETAQAFWLEIFCTFVILFASVWMAYDHRQAK
ALGHVLVFSIVGVVLGLLVFISTTVTAKRGYAGAGMNPARCFGAAVVRGGHLWNGHWIFWVGPAIACVAFYLYTK
IIPRQHHRAKFYDHDFFNVVKAMF

>FraexSIP1;1 FRAEX38873_v2_000049240.1
MGAIKAAVADGVLTFMWVFCASTLGALTFVVASALGVAQGLPTLLVTTVLVFLLLFVFGIIGDFLGGATFNPTAI
AAFYAAGIGGADSLFSAALRFPAQAAGAVGGALAIMEVMPMQYKHMLGGPSLKVDLHTGAIAEGVLTFIITFAVL

LIVLKGPSNPLVKNWLLAMSTVSVVVAGSSYTGPAMNPANAFGWAYVYNKHNTWEQFYVYWICPFAGAILAAWTF
RFLFPHPTKQPKAKKA
>FraexSIP1;2 FRAEX38873_v2_000305830.1
GGAESLVTAAVRFPAQAAGAVGGALGIMECMPLQYKHMLEGPSLKVDLHTGAIAEGILTFAITFAVLYIVLRGPS
NLIVKNWLLAMSTVALVVAGSSYTGPSMNPANAFGWAYVNNTHKTWEQFYVYWICPFIGAILAAWVFRFLFPPPP
VKKQKKA
>FraexSIP1;3 FRAEX38873_v2_000020440.1
MLILGKSSIFFYCPRSLFIRPRDPILHWVLLRRWALTFVVASALGVAQGLPTLFITTVLVFILLFLFSIIGDVLG
GATFNPTAIAAFYAAGIGGPDSLVSAALRFPTQAAGAVGGALAVMEVMPIQYKHMLGGPSLKVDLHTGAIAEGVL
TFTITFFAVLLIVLRGPSSPLMKNWLLAMPTVSLVVAGSSYTGPSMNPANVSGASLSLSLDYATIR
>FraexSIP2;2 FRAEX38873_v2_000151460.2
MAAVGGRRLLVSDFLMSFMWVWSSVLIKIFVFKILGYGSHDLRGEILRYGISIVNMFFFAFLAKSTKGGAYNPLT
VLSSAISGDFSNFLFTVGARIPVQVLGSIYGVRLILAAFPEIGRGPRLNVGIARGALTEGFLTFGIVIISQGLSR
KIPGSFFMKTWISSISKLTLHMLGSDLTGGCMNPASVMGWAFARREHLSKEHLIVYWLAPIEATLAAVWAFRVTV
LPEKDAKPKVKSGKSE
>FraexSIP2;1 FRAEX38873_v2_000056860.1
MAAVGRRRLLVSDFFMSFMWVWSSVLIKIFVHKILGYGAHDLKGEILRYGIYIVNMFFFAFLAKATKGGAYNPLT
ILSSAISGDFSNFLFIVGARIPIQVLGSIYGVRLILAAFPEIGRGPRLNVDISRGALTEGILTFGIVIISQGLSR
KIPGSFFMKTWISSVSKLTLHILGSDLTGGCMNPASVMGWAFARGEHISKEHLIVYWLAPMEATLAAVWAFRVIV
RPKKDETPKVKSGKSD

>FraexNIP1;1 FRAEX38873_v2_000146020.2
MAEISSGVNGNHGMSVNIKLEEDSNHQTLTESANSSHGNSSGCSLLTLPFIQKIIAEILGTYFLIFAGCAAVVVN
ADKDKVVTHPGISIVWGLVVMVMVYSVGHISGAHFNPAVTIAFATCKRFPWKQVPAYILAQVIGSTLASGTLRLL
FTGKHDHFPGTLPAGSDMQSLVVEFIITFYLMFVISGVATDNRAIGELAGLAVGSTVLLNVMFAGPISGASMNPA
RSLGPAIVSKQYKGLWIYMVGPITGAVAGAWVYNIIRFTDKPLREITKSGSFLKTSSRNVSNR
>FraexNIP1;2 FRAEX38873_v2_000067570.2
MAEMSGANGVALNVKDDLNEKHIQNSPDWLPFIQKVIAEILGTYFLIFAGCASVVVNADKDKVITMPGIAIVWGL
VVMVMVYSVGHISGAHFNPAVTIAFASCKRFPLRQVPAYISAQMIGATLASGTLRLLFNGKHDHFGGTLPTGSNM
QSFVVEFIITFYLMFVISGVATDNRAIGELAGLAVGATILLNVMFAGPISGASMNPARSLGPAIVSNKYKGIWIY
ILAPTLGAVAGAWVYKYHQIHKQAFKRNHQECFFPQELGPEWAQLIIKLIWF
>FraexNIP1;3 FRAEX38873_v2_000371660.1
MAEMSGANGLADEKRIQNSPAWLPFIQKVIAEILGTYFLIFAGCAAVVVNADKDKVITMPGIAIVWGLVVMAMVY
SVGHISGAHFNPAVTIAFATCKRFPLRQVPAYIAAQVIGATLASGTLRLLFMGKHDHFAGTLPTGSDLQSFVVEF
IITFYLMFVISGVATDNRAIGELAGVAIGATILLNVMFAGPISGASMNPVRSLGPAIVSNRYEGIWIYIVGPTLG
AVAGAWAYNIIRFTNKPLREITKTASFLKI
>FraexNIP1;4 FRAEX38873_v2_000067580.1corrected
MAEMSGANGVALNVKDDLNEKHIQNSPDWLPFIQKVIAEILGTYFLIFAGCASVVVNADKDKVITMPGIAIVWGL
VVMVMVYSVGHISGAHFNPAVTIAFASCKRFPLRQVPAYISAQMIGATLASGTLRLLFNGKHDHFGGTLPTGSNM
QSFVVEFIITFYLMFVISGVATDNRAIGELAGLAVGATILLNVMFAGPISGASMNPARSLGPAIVSNKYKGIWIY
ILAPTLGAVAGAWVYNIIRFTNKPLREITKSASFLKSSARNGHN
>FraexNIP1;5 FRAEX38873_v2_000326700.1
MTSRVRWSSMPRVPRLQDEEDNDWIENGYEDTDGSLSADDNDEIDCRKRGPNHGSNVCADETGRIPTNHDSNTSP
ISSVSTDGDSSRNCFNAFFMQKLLAELIGTYFLVFAGCTAVVVNMDMNRVVTLPGIALVFGLVLMVLVYSVGHVS
GAHFNPAVTIALASCGKFPLMQVPGYISVQILGSILASATIRLLFEGNQDKFAGTLPTGSDLQAFLIEIITTFLF
LFVISSLTTDDRAVTKFILEECTIIVMVAGPISGGSVNPARSLGPAIVSSRYERLWIYLLGPILGAIGGAWVYNL
IRLQAKFSIENVTNHSLF
>FraexNIP2;1 FRAEX38873_v2_000215090.1
MDNRQQNFCDLNNTPGLAPVDNFKSQISLSCVGRRLKENYPSAFLNKVTAEIIATYLLVFVTCGSAALSASDEHE
VSKLGASVAGGLIVTVMIYAVGHISGAHMNPAVTLAFAAVSRFPWKQVPFYAVAQLIGATSAGFTLRVLLHPIKH
VGTTSPSGTDIQALIMEIVVTFSMVFIASAVATDTKAIGELGGVAVGSAVCISSILGGPISGGSMNPARSIGPAI
ASAYYKGIWVYIIGPVCGALLGAMSYNFIRDSDKPAHAISTQSVSFKLCRLKSNNEQIADNDTLNQL
>FraexNIP3;1 FRAEX38873_v2_000201220.1
MANSPSIISFSPKQNLPTDLSITEEAKGYSAQEFPPRDSISPSYYQQIIAEILGTYVLVFAGCGAALVDREKTLT
VVGLAMVWGLALMAMIYALGHVSGGHFNPAVTIGFAAAQKLPLIHVPLYVLSQFLGSTLACLTLKVLFNDQADIL
PTLTRYSSSTTDLEAIIWEFIITFVLMLTIRGVASDDRANNELAGVAIGATVLFNALVAGPITGASMNPARSIGP
AIVSGVYKNQWVFVVAPILGAIAASLIYSLLQPWKPGKHKESARSTYNDLYLCPEA
>FraexNIP4;1 FRAEX38873_v2_000205820.1
MAIKEMEISKMEEGVDTSTFQSNAKEGLCTSASIVTLVQKVIAEVIGTYFIIFSGCGVVVVNKLYGGTVTFPGIC
ATWGLIVMVMVYSVGHISGAHFNPAVTITFAIFRRFPWKEVPLYIMAQLIGSILASCTLAIMFDVTSEAYFGTLP
VGSNGQSLTIEIIISFLLMFNISGVATDSRAIGDLAGLAVGMTIMLNVFVAGPISGASMNPARSIGPAIVKHTYK
GLWVYIFGPLIGTIFGGFAYNLIRFTNKPLRELTKSSSVIRTISKHNTT
>FraexNIP5;1 FRAEX38873_v2_000251140.1

MAEFEPGTPGTPTTPTPLMPSLRVDSLSYDRKSMPRCKCLPLNVSTWAAPHTCLTHFSAPEISLTRKVAAEFVGT
FILIFGATAGPIVNQKYDGAEGLVGNAACAGLAVMIAILSTGHISGAHLNPSLTIAFAAFRHFPWIHVPAYIAAQ
VSASICACFALKGVFHPFLSGGVTIPSVSSGQAFGLEFLITFTLLFVVTAVATDTRAVGELAGLAVGATVMLNIL
VAGPSSGGSMNPVRTLGPAVAAGNYKSIWIYLVAPTLGALAGAAIYALVKLQGVEDDEPRHQQG
>FraexNIP5;2 FRAEX38873_v2_000203670.1
MASLKSRLEHLVTAIALRLYLAEFISTFFFVFAAVGTTMSSRKMMLDAASNSSSLVAIVVANAFALSVAVYISAN
IFGGHVNPVDTFGMAMGSHISIPMAIFYSISQMFGSVMACLLLKATTAVQHVPTHVIEHEMTGFEGAVLKGVMTF
ALVYMVYVASNLRPVSAGAIGPLAIGLVAGANVLASGPFTGRSMNPAYSFGSAIVGGSFKNQEVYWVGPLIGAAI
ARILHDNVVFPAVVPDSFRGNADGVGV
>FraexNIP6;1 FRAEX38873_v2_000011710.1
MGTEDVASVPKTPAIPGTPGCTPLFGGQFKQERGGDGRKSLFKNCKCFSVETLSLEEGTLPSVSCALIPPPFSLA
RKVGAEFIGTFILIFTGTATAIVNQKTQGSETLLSLAASTGLAVMIVIQSTSHISGAHVNPSVTIAFAALKHFPW
KHVPVYIGAQILASTCAAFLLKAIFHPIMGGGVTVPSGGFAQAFALEFIISFNLMFVVTAVATDTRAVGELAGMG
VGATVMLNILIAGETTGASMNPVRTLGPAIAANNYKAVWIYLTAPILGALAGAAAYSAVKLPDRDVNGQKKLPNA
EHSFSR
>FraexNIP6;2 FRAEX38873_v2_000070290.2
MDTEDVASAPSTPVTPGTPGAPLFGGFKRDRNSFNGRNSSLLKSCKCFSVGTWELEEGSLPPVSCALAPPSVSLA
RKVGAEFIGTLILIFTGTATAIVNQKTNGSETLIGLAASSGLAVMVIILSTGHISGAHLNPAITISFAVLKHFPW
KHVPSYIGAQVMASLTASFLLKGIFNSIMGGGVTVPSVRYAQAFALEFIITFNLMFVLTAVATDTRAVGELAGIA
VGATVMLNILIAG
>FraexNIP7;1 FRAEX38873_v2_000066660.1
MQQSIKAPFRELSSSPDISDSTATSSQPKNDLENGASTLNKTLFNSLHKIDKSLLRKVLAEALGTFMLMFCICAI
IAIMHLIGVEVGLMEYAATAALSVTVIVFSIGAISGAHVNPAVTIAFATVGPFPWSEVPLYILAQIGGSALATYI
ARLVYGIRSELVMTKPLQGCTAAFWVELIATFIILFLTASLSNDPQSIGQVSGFVVGVAIGLGVLISGPVSGGSM
NPARSLGPALVSWKFDDHLWIYLVAPTIGAIAGVFAYRILRLQGWPCESSQAQKGSDSLH

>FraexTIP1;1 FRAEX38873_v2_000139770.2
MPISRVAIGSPVEASQPDAIKAAVAEFISMLIFVFAGEGSGMAFAKLTDNGSTTPAGLIAASIAHAFALFVAVSV
GANISGGHVNPAVTFGAFVGGHITLFRSILYWIAQLLGSVVACLLLKFATGGLETSAFALSSGVTVWNAVVFEIV
MTFGLVYTVYATAVDPKKGNLGIIAPIAIGFIVGANILAGGAFDGASMNPAVSFGPAVVSWTWDSHWVYWLGPFV
GAGIAALVYEILFINQSHEQLPTSAEY
>FraexTIP1;2 FRAEX38873_v2_000060210.1
MPLSRISVGSPAEARHPDTLKAALAEFISMLIFVFAGEGSGMAFAKLTDGGSSTPAGLIAAAIAHAFALFVAVSV
AANISGGHVNPAVTFGAFVGWSHIIVEIHFVLDSPIAWICCCMLAPKVLHWWAGNVCICTFIRVDPKRGDIGTIA
PIAIGFIVGANILAGGAFDGASMNPAVSFGPAVVSWTWDSHWVYWLGPFVGAGIAALVYEIVFINQNTHEQLPTN
EY
>FraexTIP1;3 FRAEX38873_v2_000314120.1
MSSEYFNVPFRHVAIGNQNEFRQPGAIKAALAEFISTLIFVFAGEGSGMAYNKLTGNAPSSPSGLVSAAVAHAFG
LFVAVSISANISGGHVNPAVTFGLFLGGNISLIRGILYIIAQLLGSVVACLLLLFSTGGLETSAFALGADVSVWN
AFVFEIVMTFGLVYTVYATAVDPKKGELGIIAPIAIGFIVGANILAGGAFTGASMNPAVSFGPALVSFTWTHHWV
YWAGPLIGGGLAGIVYEVFFISHSHEPLPGSEY
>FraexTIP1;4 FRAEX38873_v2_000086560.1
MSSEFFNVPLRRVAFGHRDEFRQPGAIKAALAEFISTLIFVFAGQGSGMAYNKLTGNAPSSPSGLVAAAVAHGFG
LFVAVSTSANISGGHVNPAVTFGLFLGGNISLFRGVLYIIAQLLGSVVACLLLLFTTGGLETSAFALSAGVSVWN
AFVFEIVMTFGLVYTVYATAVDPKKGEVGIIAPLAIGLIVGANILAGGAFTGASMNPAVSFGPALVSFNWTHHWV
YWAGPVIGGGLAGIVYEVFFISHSHESLPSSEY
>FraexTIP1;6 FRAEX38873_v2_000091910.1
MPIYQIAIGTPGEASHPDALKAALAEFFSMLIFVFAGQGSGMAFNKLSNDGSSTPSGLLAAALAHAFALFVAVSV
SANISGGHVNPAVTFGAFIGGNITLLRGILYWLAQLLGSVVACMLLKFATGGLEISAFALSSGVSVWNALVFEIV
MTFGLVYTVYATAVDPKKGDLGSIAPIAIGFIVGANILAGGAFDGASMNPAVSFGPALVSWNFTHHWVYWFGPLI
GATIAALVYDNIYINGEITHEQLPVTDY
>FraexTIP2;1 FRAEX38873_v2_000292820.1
MPAIAFGRFHDSFSIGSLKAYVAEFISTLLFVFAGVGSAIAYNKLTADAALDPAGLVAVAICHGFALFVAVSIGA
NISGGHVNPAVTFGLALGGQITLLTGLFYWIAQLLGSIVACYLLVFVTGGLAIPTHGVGAGVGAIEGVVMEIVIT
FALVYTVYATAADPKKGSLGTIAPIAIGFIVGANILAAGPFSGGSMNPARSFGPAVASGNFSGIWIYWVGPLIGG
GLAGLIYANVFMSHEHTPLATDF
>FraexTIP2;2 FRAEX38873_v2_000028480.1
MQAIAFGRFDDSCRLGSLKAYLAEFISTLLFVFAGVGSAIAYNKLTSDAALDPAGLVAVAVCHGFALFVAVSVGA
NISGGHVNPAVTFGLAVGGQITVLTGLFYWIAQLLGSIVACFLLKVVTGGLAIPTHGVAAGVGAIEGVVMEIVIT
FALVYTVYATAADPKKGSLGTIAPIAIGFIVGANILAAGPFSGGSMNPARSFGPAVASGDFAGNWIYWVGPLIGG
GLAGLVYANVFMVHEYTPLVDEF
>FraexTIP2;3 FRAEX38873_v2_000217250.1

MPTIAFGRINDSISVGSLKAYLAEFISTLLFVFAGVGSAIAYNKLTSDAALDPAGLVAVAVCHGFALFVAVSVGA
NISGGHVNPAVTFGLAVGGQITILTGLFYWIAQLLGSIVACLLLKFVTGGLAIPIHGVAAGVGAIEGVVMEIVIT
FALVYTVYATAVDPKKGSLGTIAPIAIGFIVGANILAAGPFSGGSMNPARSFGPAVASGDFAGNWIYWVGPLIGG
GLAGLVYANVFMVHEHTPLVSEF
>FraexTIP2;4 FRAEX38873_v2_000269930.1
MVRINLGSIGDSFSVPSLKAYIAEFIATLLFVFAGVGSAIAYNKLTSDAALDPPGLVAVAVAHAFALFVGVSMAA
KASGGHLNPAVTFGLAIGGNITIITGLFYWIAQLLGSIVACLLLKFVTNGLAIPTHGVASGMNAVEGVVMEIIIT
FALVYTVYATAADPKNGPLGTIAPIAIGFIVGANILAAGPFSGGSMNPARSFGPAVVAVDFSQNWIYWVGPLIGG
GLAGLVYGDVFIGSYAPLPVQEEYP
>FraexTIP3;1 FRAEX38873_v2_000352370.1
EEATHPDSARATLAEFLSTLVFVFAGEGSVLALDKMYKDTALGASSLVVIALAHALSLFAAVASSMNVSGGHVNP
AVTFGALVGGRVSVLRAIYYWIAQLLGSVLAAILLRLATDGLRPRGFSVADGVGEWNALLMEIVLTFGLVYTVYA
TAIDPKRGSLGTIAPLAIAFIVGANVLVGGPFSGASMNPARAFGPALVGWRWRNHWVYWLGPFVGAALAGVIYEF
GMIQAETPHHSHHLHQPLTPEDY
>FraexTIP3;2 FRAEX38873_v2_000383670.1
MPRGYTFGRAEEATHPDSARATLAEFLSTFVFVFAGEGSILALDKMYRETALGASSLLVIALAHALALFAAVASS
MNVSGGHVNPAVTFGALVGGRVSVLRAVFYWIAQILGAIVASLLLWLATDGLKPQGYSVAAGVGEWNALVMEIVL
TFGLVYTVYSTAIDPKRGSLGTIAPLAIAFIFGANVLVGGPFSGASMNPARAFGPALVGGRWRDHWIYWLGPLLG
AALAGVIYEFGMIQTETPHHTHHQPLAPEDY
>FraexTIP4;1 FRAEX38873_v2_000264810.1
MAKIALGNTREAFQSDCIQALIVEFICTFLFVFAGVGAAMATDKLNGDPLVGLFFVAMAHALVVAVMISAGFRIS
GGHLNPAVTLGLLAGGHITVVRSILYWIDQLLASTAACALLKYLTGGLTVPVHTLATGVDFIQGVIMEIVLTFSL
LFTVYTTIVDPKKGFLEGMGPLLTGLVVGANVMAGGPFSGASMNPARSFGPALVSGDWTDHWVYWVGPLIGGGLA
GYIYENFFIVRSHVPLSREVVF
>FraexTIP5;1 FRAEX38873_v2_000139560.1
MASLKSRLEHTVTPTALRSYLAEFISTFFFVFASVGATMSTLKMMPDAASDSSSLVAIAVANAFALSVAVYIAAN
ISGGHVNPAVTFGMAIGGHISIPMAIFYWISQTLGSVMACVLLRVTTVGQRVPTHSIPQEMTGFGAAILEGVMTF
TLVYTVYAAADPRRGSISVIGPLAIGLVSGANVLASGPFTGGSMNPSYSFGSALVRGSFKNQAVYWIGPLIGAAI
AGILYEHVVFPAQQEPDSLRGIARGVEV
>FraexTIP5;2 FRAEX38873_v2_000166170.2
MPEFEVGTPAMSAPATPGTPTPLMSSLRIDSLSYDRKSMPRCKCLPVDAPTWGAPHTCSLTNFSVPDISLTRKLG
AEFVGTFILIFGATAGPIVNQKYNQAETLIGNAACAGLAVMIVILSTGHISGAHLNPSLTIAFATLRHFPWAHVP
AYIAAQVSASICASFALKGVFHPFMSGGVTVPSVSTGQAFALEFLITFNLLFVVTAVATDTRAVSYQLNTNIIFF
FLFFKYYKVINPCLQVGELAGIAVGATVMLNILVAGPSSGGSMNPVRTLGPAVAAGNYKAVWVYLVAPTLGALAG
AGIYTLVKLNGEEEEAPRQVRSFRR

<u>Pseudogene/incomplete *Oleur*AQP sequences</u>

>FraexPIP2-related FRAEX38873_v2_000337260.1
MVHLATIPITGTSINPARSLGPAVIYNQKKAWDDQWIFWVGPFIGAAMAAFYHQCILRAKAFGSYGSSSHV*
>FraexPIP2-related FRAEX38873_v2_000295860.1
VLAPLPIGFAVFMVHLATIPITGTGINPARSLGAAVIYNQEKAWDDQWIFWVGPFIGAAIAAFYHQFILRAGAVK
ALGSFRSSSHV*
>FraexPIP2-related FRAEX38873_v2_000121300.1
MAKGVGVVEHGSFTAKDYHDPPPAPLIDAEELTKWSFYRAVIAEFIATLLFLYITVLTVIGYKSQIDTTAGHGGD
QCGGVGILGIAWAFGGMIFVLVYCTAGISGEFCLYAHINAYKYGLITIRK*
>FraexNIP5-related FRAEX38873_v2_000192600.1
MAEFEPGTPGTPTTPTPLMPSLRVDSLSYDRKSMPRCKCLPLNVSTWAAPHTCLTHLSAPEISLTRK
