# Supplementary material for: Genome Wild Analysis and Molecular Understanding of the Aquaporin Diversity in Olive Trees (Olea Europaea L.)
Source: Int J Mol Sci. 2020 Jun 11;21(11):4183. doi: 10.3390/ijms21114183 (PMC7312470; doi:10.3390/ijms21114183)
Supplement: Supplementary file 1 [file ijms-21-04183-s001.zip › ijms-821530_Supplementary_files/Figure S4_Faize_MIP_Phylogeny.pdf]

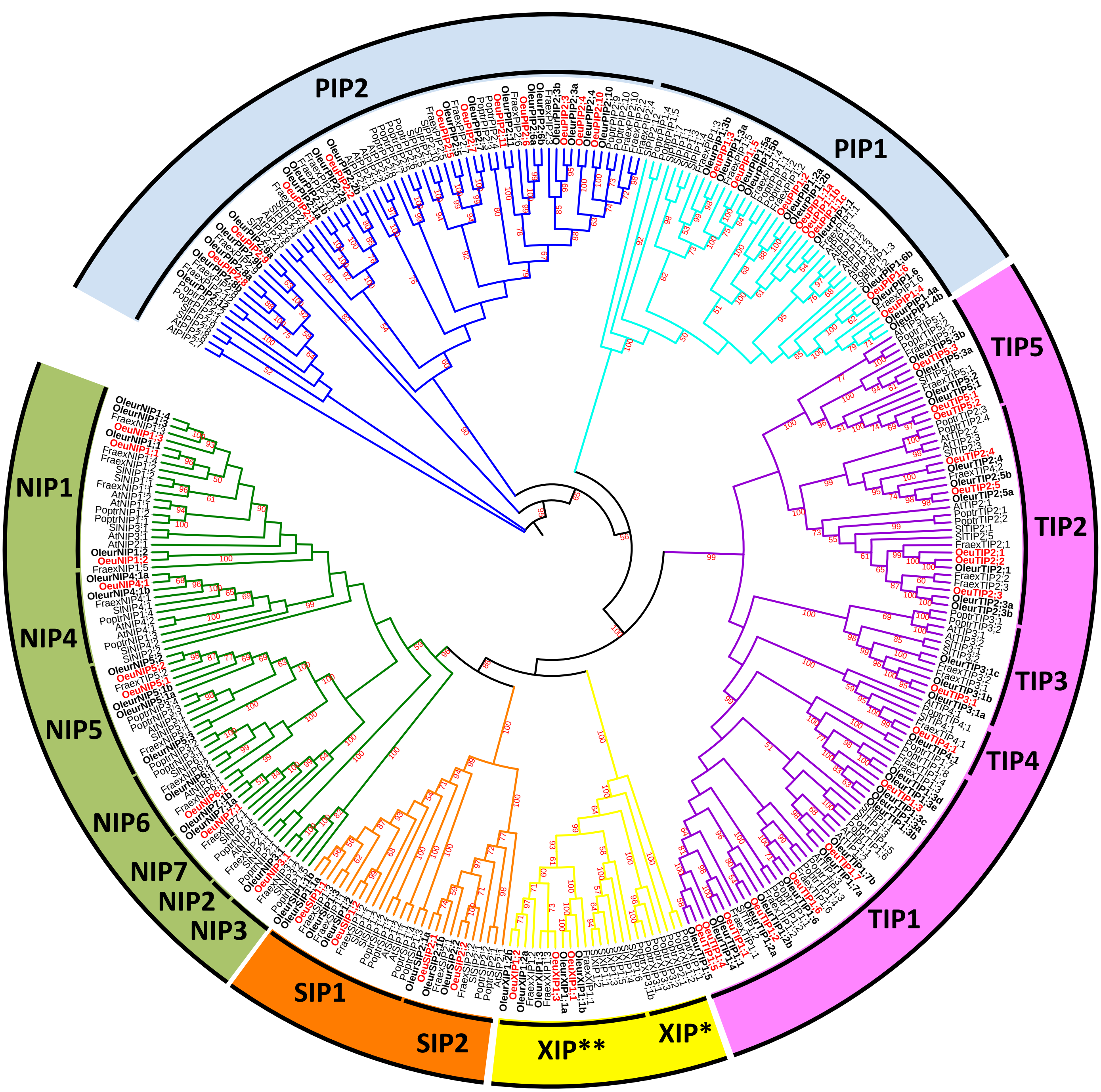

Figure S4. Phylogenetic analysis of protein sequences of the 52 *Oeu*AQPs and the 79 OleurAQPs with *Fraxinus excelsior*, *Arabidopsis thaliana* and *Populus trichocarpa* hortologs. Deduced amino acid sequences were aligned using ClustalW, and the phylogenetic tree was constructed using the Maximum Parsimony method. Maximum Parsimony analysis was conducted using the Subtree-Pruning-Regrafting algorithm. The number next to the branches represents bootstrap values ≥50% based on 5000 resampling. The distance scale denotes the number of amino acid substitutions per site. The AQP sequences from *O. europaea* var. *sylvestris*, *O. europaea* cv. Picual, *F. excelsior*, *A. thaliana* and *P. trichocarpa* are preceded by the prefixes Oeu, Oleur, Fraex, At, and Poptr, respectively. *Oeu*AQPs are mentioned in red, and *Oleur*AQP in bold. The name of each subfamily and subgroup is indicated next to the corresponding group. XIP* and XIP** correspond to XIP sequences from Rosids and Asterids, respectively. *Oeu*AQP accession numbers and sequences are listed in Table 1 and Figure S1. *Oleur*AQP, *Fraex*AQP accession numbers and sequences are listed in Figures S2 and S3, respectively.
