# Supplementary material for: Genome Wild Analysis and Molecular Understanding of the Aquaporin Diversity in Olive Trees (Olea Europaea L.)
Source: Int J Mol Sci. 2020 Jun 11;21(11):4183. doi: 10.3390/ijms21114183 (PMC7312470; doi:10.3390/ijms21114183)
Supplement: Supplementary file 1 [file ijms-21-04183-s001.zip › ijms-821530_Supplementary_files/Figure S5_Faize_OeuMIP_protein_Alignment.pdf]

Figure S5 ABCDE. Amino acid sequence alignments per subfamily of the *Oeu*AQPs members from *Olea europeae* var. *sylvestris*. 5A, PIP; 5B, TIP; 5C, XIP; 5D, NIP; 5E, SIP. Legends are detailed at the end of each subfamily alignment.

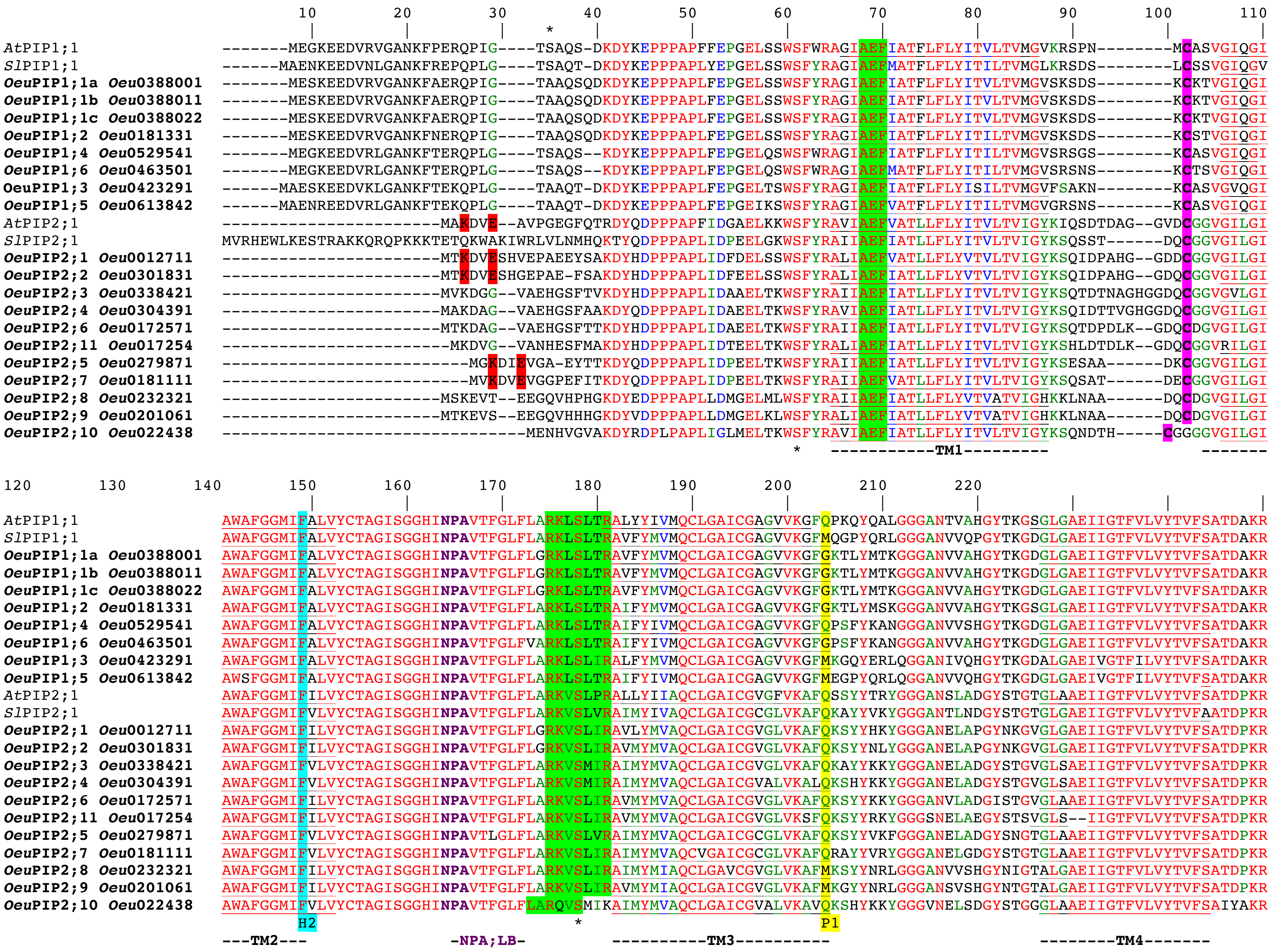

AtPIP1;1
SlPIP1;1
OeuPIP1;1a Oeu0388001
OeuPIP1;1b Oeu0388011
OeuPIP1;1c Oeu0388022
OeuPIP1;2 Oeu0181331
OeuPIP1;4 Oeu0529541
OeuPIP1;6 Oeu0463501
OeuPIP1;3 Oeu0423291
OeuPIP1;5 Oeu0613842
AtPIP2;1
SlPIP2;1
OeuPIP2;1 Oeu0012711
OeuPIP2;2 Oeu0301831
OeuPIP2;3 Oeu0338421
OeuPIP2;4 Oeu0304391
OeuPIP2;6 Oeu0172571
OeuPIP2;11 Oeu017254
OeuPIP2;5 Oeu0279871
OeuPIP2;7 Oeu0181111
OeuPIP2;8 Oeu0232321
OeuPIP2;9 Oeu0201061
OeuPIP2;10 Oeu022438
10 20 30 40 50 60 70 80 90 100 110
120 130 140 150 160 170 180 190 200 210 220
TM1
TM2
-NPA;LB-
TM3
TM4
H2
P1

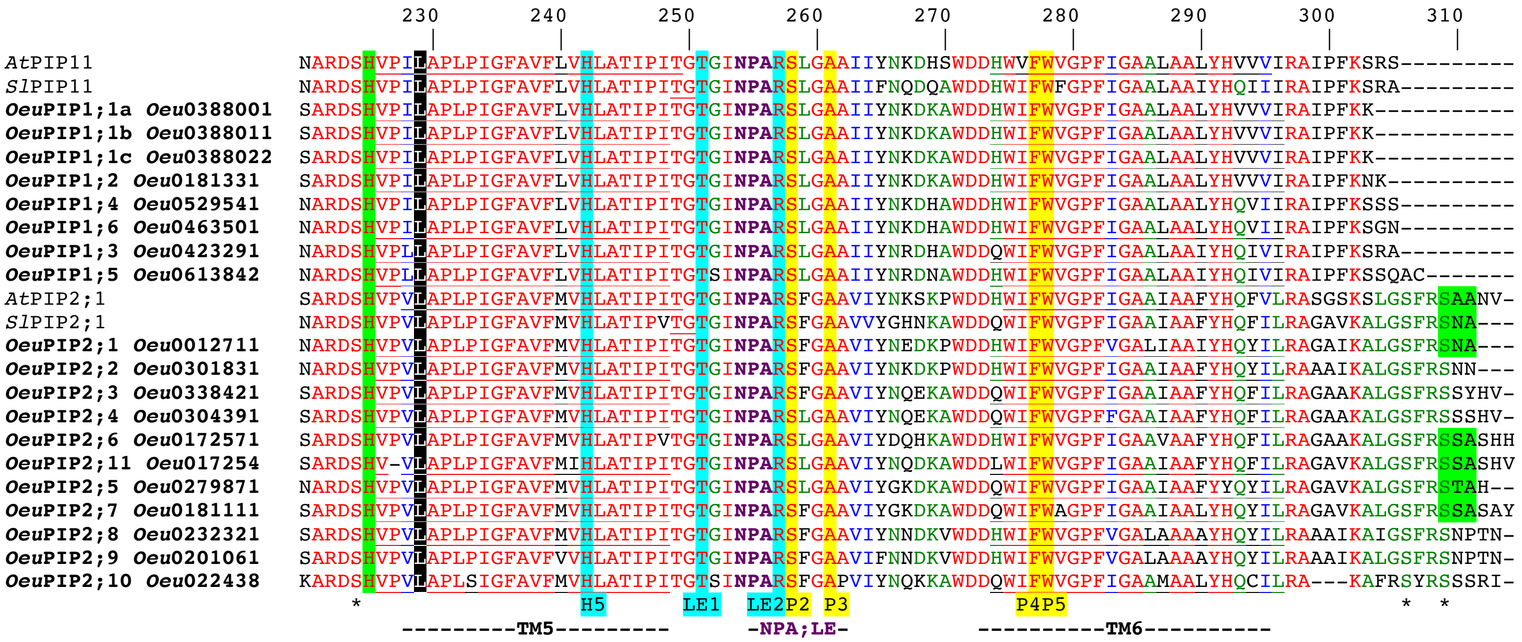

Figure S5A. Amino acid sequence alignment of the *Oeu*PIPs subfamily members from *Olea europeae* var. *sylvestris*. *Sl*PIP1;1 and *Sl*PIP2;1 (Solyc10g054840.1; Reuscher et al., 2013) from Tomato and *At*PIP1;1 (At3g61430) and *At*PIP2;1 (At3g53420) (Muries et al., 2011) from *Arabidopsis thaliana* were used as references. The highest residue similarities between MIP members are shown in red (100%), and green (>80%). Residues with similar physicochemical behaviors are shown in blue. The six transmembrane domains (TMH1-6) and the two hemi-helicoidal regions that include the «NPA» motives «NPA1» (in Loop B) and «NPA2» (in Loop E) (framed in purple) are underlined. The amino acids residues constituting the ar/R constriction region are shown with blue highlights and labeled H2, H5, LE1 and LE2, and residues constituting the Froger's positions are shown with yellow highlights and labeled P1 to P5. In addition, AEF motif, the His residue involved in gating, the RKxSxxR motif, the SxA motif for putative phosphorylation by PKC are marked in green, the KdvE motif for putative conserved methylation sites are marked in red, and the putative conserved blocking residue (L) is marked in black. Lastly, the Cysteines (C) involved in the tetramerization of PIPs are mentioned in purpled. Columns or regions with conserved putative phosphorylation sites are marked with an asterisk.

```
                                  10        20        30        40        50        60        70        80        90
                                   |         |         |         |         |         |         |         |         |
AtTIP1;2                 -------MPTRNIAIGGVQEEVYHPNALRAALAEFISTLIFVFAGSGSGIAFNKITDNGATTPSGLVAAALAHAFGLFVAVSVGANISGG
SlTIP1;1                 -------MPINQITIGS-HEELRHPGALKAALAEFISTLIFVFAGQGSGMAFNKLTDGVAT-PAGLISASIAHAFGLFVAVSVGANISGG
OeuTIP1;3 Oeu0354791     MSSEFFNVPLRRVAFGH-RDEFRQPGAIKAALAEFFSTLIFVFAGEGSGMAYNKLTGNAPTSPSGLVAAAVAHGFALFVAVAVSANISGG
OeuTIP1;1 Oeu0585311     -------MPISRVAIGS-PAEFSQLDAIKAAVAEFISMLIFVFAGQGSGMAFAKLTDNESTTPAGLIAASIAHAFALFVAVSVGANISGG
OeuTIP1;4 Oeu0402901     -------MPISRIAVGS-LAEASQPEALKATLAEFISMLIFVFAGEGSGMAFAKLTNNGSTTPTGLIAEAIAHAFALFVAVSVGANISGG
OeuTIP1;5 Oeu0402891     -------MPISRIAVGS-LAEASQPEALKATLAEFISMLIFVFAGEGSGMAFAKLTNNGSTTPTGLIAEAIAHAFALFVAVSVGANISGG
OeuTIP1;2 Oeu0410141     -------MPFSRISVGS-PAEASHPDTLKAALAEFISMVIFVFAGEGSGMAFAKLTDGGSSTPAGLIAAAIAHAFALFVAVSVAANISGG
OeuTIP1;6 Oeu0332441     -------MPIYRIAIGT-PGEASHPDALKAALAEFFSMLIFVFAGQGSGMAFNKLSNDGSPNPSGLLAAALAHAFALFVAVSVGANISGG
OeuTIP1;7 Oeu0452371     MYSNSM-RSGNQIAVGR-LEEATQQDALKAALAEFISTLIFVFAGSGSGMAFNKLTDDGATTPAGLIAAAVAHAFALFVAVSVGANISGG
OeuTIP2;1 Oeu0240741     ---------MPAIAVGR-LDDSFSIGSLKAYVAEFISTLLFVFAGVGSAIAYNKLTADAALDPAGLVAVAICHGFALFVAVSIGANISGG
OeuTIP2;2 Oeu0240761     ---------MPAIAVGR-LDDSFSIGSLKAYVAEFISTLLFVFAGVGSAIAYNKLTADAALDPAGLVAVAICHGFALFVAVSIGANISGG
OeuTIP2;3 Oeu0539881     ---------MPTIAFGR-IDDSLSVGSLKAYLAEFISTLLFVFAGVGSAIAYNKLTYDAALDPAGLVAVAVCHGFALFVAVSIGANISGG
OeuTIP2;4 Oeu0023291     ---------MVKMTLGS-IGDSFSVPSLKAYLAEFIATLLFVFAGVGSAIAYNKLTSDAALDPPGLVAVAVAHAFALFVGVSMAANVSGG
OeuTIP2;5 Oeu0402071     ---------MVKINWGS-IGDSFSVTSLKAYIAEFIATLLFVFAGVGSAIAYNKLTSDAALDPPGLVAVAVAHAFALFVGVSMAANVSGG
OeuTIP3;1 Oeu0383871     ---------MPRGYTFGRAEEATHPDSARATLAEFLSMLVFVFAGEGSVLALDKMYKDTALGASSLVVIALAHALSLFAAVASSMNVSGG
OeuTIP4;1 Oeu0302291     ---------MAKIALGN-AREAFQSDCIQALIVEFICTFLFVFAGVGAAMATDKLNGDPLV---GLFFVAMAHALVVAVMISAGFRVSGG
AtTIP5;1                 ------MRRMIPTSFSSKFQGVLSMNALRCYVSEFISTFFFVLAAVGSVMSSRKLMAGDVSGPFGVLIPAIANALALSSSVYISWNVSGG
SlTIP5;1                 -----------MASLASRLQHSVTPNALRSYLAEFLSTFFFVFAAAGASMSTRKMVPDATSDPSSLVAIAVANAFALSVAVYISANISGG
OeuTIP5;1 Oeu0217081     -----------MASLKSRLEHTVTPTALRSYLAEFISTFFFVFASVGATMSTLKMMPDAASNSSGLVAIAVANAFALSVAVYIAYNISGG
OeuTIP5;2 Oeu0217091     -----------MASLKSRLEHTVTPTALRSYLAEFISTFFFVFASVGATMSTLKMMPDAASNSSGLVAIAVANAFALSVAVYIAYNISGG
OeuTIP5;3 Oeu0224791     -----------MASLKSRLEHSVTAIALRSYLAEFISTFFFVFAAAGATMSSRKMMPDAASDSSSLVAIAVANAFALSVTVYISANISGG
                                                                                         H2
                                                -----------TM1---------         -----------TM2---------

                                 100       110       120       130       140       150       160       170       180
                                   |         |         |         |         |         |         |         |         |
AtTIP1;2                 HVNPAVTFGVLLGGNITLLRGILYWIAQLLGSVAACFLLSFATGGEPIPAFGLSAGVGSLNALVFEIVMTFGLVYTVYATAVDPKNGSLG
SlTIP1;1                 HVNPAVTFGAFVGGNITLFRGILYIIAQLLGSTAACALLEFATGGMSTGSFALSAGVSVWNAFVFEIVMTFGLVYTVYATAVDPKKGDLG
OeuTIP1;3 Oeu0354791     HVNPAVTFGLFLGGNVSLFRGILYIIAQLLGSVVACLLLLFSTGGLETSAFALTAGVSVWNAFVFEIVMTFGLVYTVYATAVDPKKGEIG
OeuTIP1;1 Oeu0585311     HVNPAVTLGAFVGGHITLFRSIMYWIAQLLGSVIACLLLKFATGGLETSAFALSSGVTVWNAVIFEIVMTFGLVYTVYATAVDPKKGNLG
OeuTIP1;4 Oeu0402901     HVNPAVTFGAFVGGHITLIRSILYWIAQLLGSVVACLLLRLATGGLETSAFALSSGVTVWNAVVFEIVMTFGLVYTVYATAVDPKRGNLG
OeuTIP1;5 Oeu0402891     HVNPAVTFGAFVGGHITLIRSILYWIAQLLGSVVACLLLRLATGGLETSAFALSSGVTVWNAVVFEIVMTFGLVYTVYATAVDPKRGNLG
OeuTIP1;2 Oeu0410141     HVNPAVTFGAFVGGHLSLFKTILYWIAQLLGAIVACLLLKFSTGGLETSAFALSTGVSSWNALVFEIVMTFGLVYTVYATAVDPKRGDIG
OeuTIP1;6 Oeu0332441     HVNPAVTFGAFIGGNITLLRSILYWIAQLLGSVVACILLKFATGGLETSAFALSSGVSVWNALVFEIVMTFGLVYTVYATAVDPKKGNLG
OeuTIP1;7 Oeu0452371     HVNPAVTFGAFVGGNISLIRGILYWIAQLLGSVVACLLLKFATGGLTTAAFSLSAGVNVWNAFVLEIVMTFGLVYTVYATAVDPKRGSLG
OeuTIP2;1 Oeu0240741     HVNPAVTFGLAVGGQITLLTGLFYWIAQLLGSIVACYLLVFVTGGLAIPIHGVGAGVGAFEGVVMEIVITFALVYTVYATAADPKKGSLG
OeuTIP2;2 Oeu0240761     HVNPAVTFGLAVGGQITLLTGLFYWIAQLLGSIVACYLLVFVTGGLAIPIHGVGAGVGAFEGVVMEIVITFALVYTVYATAADPKKGSLG
OeuTIP2;3 Oeu0539881     HVNPAVTFGLAVGGQITILTGLFYWIAQLLGSIVACFLLKFVTGGLAIPTHSMAAGVGAIEGVVMEIVITFALVYTVYATAVDPKKGSLG
OeuTIP2;4 Oeu0023291     HLNPAVTFGLAIGGNITIITGIFYWIAQLLGSTVACLLLKFVTNGLAIPTHAVASGMNAVEGVVMEIVITFALVYTVYATAADPKKGSLG
OeuTIP2;5 Oeu0402071     HLNPAVTFGLAIGGNITIITGLFYWIAQLLGSIVACLLLKFVTGGLAIPTHAVASGMNAVEGVVMEIVITFALVYTVYATAADPKKGSLG
OeuTIP3;1 Oeu0383871     HVNPAVTFGALVGGRVSVLRAIYYWIAQLLGAVLAAILLRLATDGLRPRGFSVADGVGEWNALVMEIVLTFGLVYTVYATAIDPKRGSLG
OeuTIP4;1 Oeu0302291     HINPAVTLGLLAGGHITVVRSILYWIDQLLASTAACALLKYLTGGLTIPVHTLATGVDFAQGVIMEIVLTFSLLFTVYATIVDPKKGFLE
AtTIP5;1                 HVNPAVTFAMAVAGRISVPTAMFYWTSQMIASVMACLVLKVTVMEQHVPIYKIAGEMTGFGASVLEGVLAFVLVYTVFT-ASDPRRGLPL
SlTIP5;1                 HVNPAVTFGMAVGGHISIPMSIFYWISQMIGSVTACLLLKFTN--QQVPTHGIPQEMTGFGAAVLEGVMTFGLVYTVYA-AADPRRCVHA
OeuTIP5;1 Oeu0217081     HVNPAVTFGMAIGGHISIPMAIFYWISQTLGSVIACILLKVTTVGQHVPTHSIPQEMTGFGAAILEGVMTFTLVYTVYA-AADPRRGSMS
OeuTIP5;2 Oeu0217091     HVNPAVTFGMAIGGHISIPMAIFYWISQTLGSVIACILLKVTTVGQHVPTHSIPQEMTGFGAAILEGVMTFTLVYTVYA-AADPRRGSMS
OeuTIP5;3 Oeu0224791     HVNPAVTFGMVVGGHISIPMAIFYSISQTFGSVMACLLLKATTAGQHVPTHAIEHEMTGFGGAVLEGVMTFALVYTVYV-ASNLRPVSAG
                                                                  P1
                           -NPA;LB--            ----------TM3----------         -----------TM4----------
```

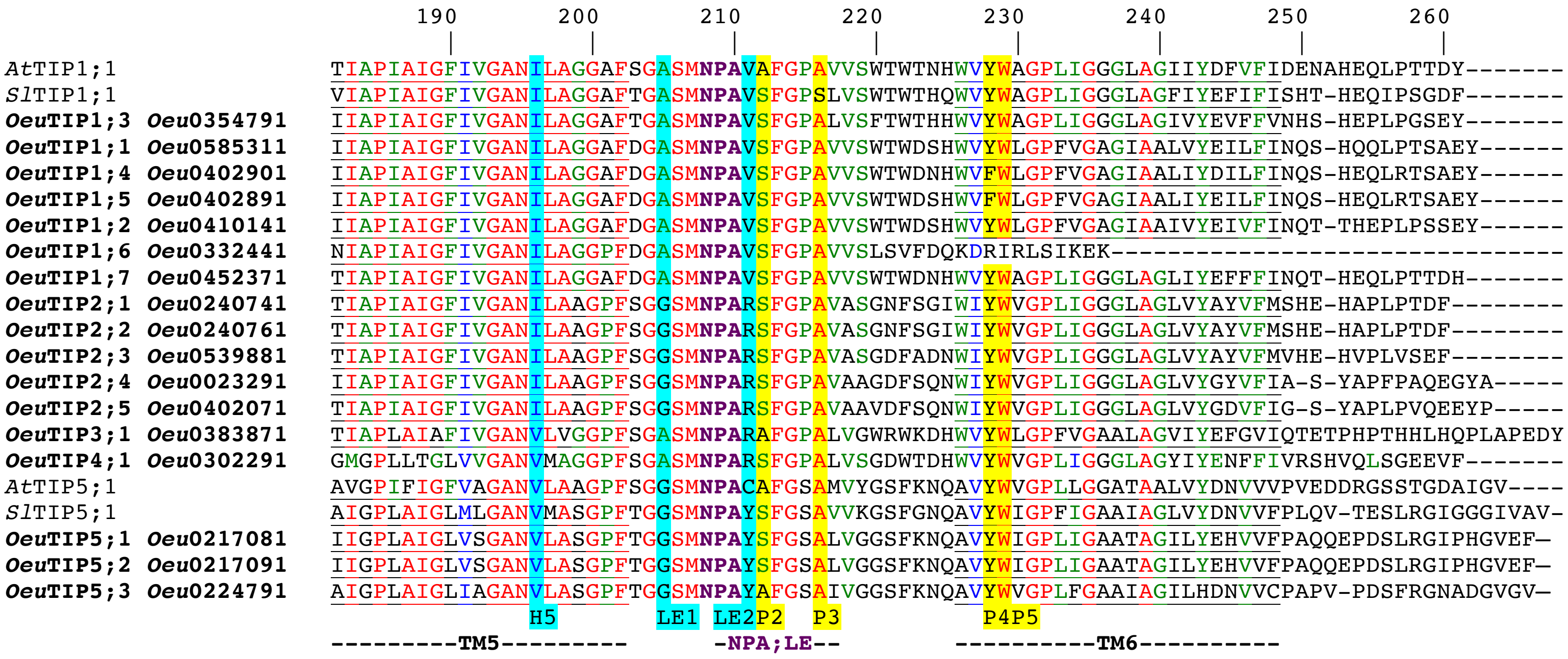

Figure S5B. Amino acid sequence alignment of the *Oeu*TIPs subfamily members from *Olea europeae* var. *sylvestris*. *Sl*TIP1;1 (Solyc06g074820.1) and *Sl*TIP5;1 (Solyc03g093230.1) (Reuscher et al., 2013) from Tomato and *At*TIP1;1 (At2g36830) and *At*TIP5;1 (At3g47440) from *Arabidopsis thaliana* were used as references. The highest residue similarities between MIP members are shown in red (100%), and green (>80%). Residues with similar physicochemical behaviors are shown in blue. The six transmembrane domains (TMH1-6) and the two hemi-helicoidal regions that include the «NPA» motives «NPA1» (in Loop B) and «NPA2» (in Loop E) (framed in purple) are underlined. The amino acids residues constituting the ar/R constriction region are shown with blue highlights and labeled H2, H5, LE1 and LE2, and residues constituting the Froger's positions are shown with yellow highlights and labeled P1 to P5.

```
                            10        20        30        40        50        60        70        80        90       100
                             |         |         |         |         |         |         |         |         |         |
Oeu0100561 OeuXIP1;1   MAANIWNVHGVDDEESQTSLSKIQPVPSTPVQVLFFLNLKFNIFSFLTQQLTADLDKKQTPLTSSQMLGLNELFSLDVWRASVGELIGSAVLVFMIDTI
Oeu0206841 OeuXIP1;2   MAGNNGHVNVEYDEEGLSGASKIQPVFSTPMQ------------------NRTDEEKMKKTTSLTLSELLGLDDFLSSDVWRASVGELVGTAVLVFMLDTI
Oeu0461221 OeuXIP1;3   MAGDNGHVHVEYDEESLSSANKIQPVFSTPMQ------------------HWTDEQKRTKNTSLTLSESLGLEDFLSLDVWRASLGELIGTAVLVFMLDTI
SlXIP1;1               MASNSNVVFGDEESQISGGTNRVQPCSSTPRKI-----------------NTIDDDEGKKHNFLSLSQRLGVADFFCLDVWRASMGELLGSAVLVFMLDTI
PoptrXIP2;1            MAGNAGVV---QDEEIGYGGNKVQPFASTPRP--------------------SKTERGKRDSSALSRILGLDELVSLNVWRASLAEVFGTAVLVFAMDTI
                                                                                         ----------------TM1-----------

                           110       120       130       140       150       160       170       180       190       200
                             |         |         |         |         |         |         |         |         |         |
Oeu0100561 OeuXIP1;1   VISTFETDIKMPNLIISILIAIVVAILLLAVHPVSGGHINPVISFSAGLVGLISMSRAIVYILAQCIGAILGALALKAVVSSTIEQNFSLGGCTLTVIAP
Oeu0206841 OeuXIP1;2   VISTIESDIKIPNLVLSILAAIIIAILLLAVHPVSGGHINPVISFSAALVGLISMSKAFIYIVAQCLGAVLGALALKAVVSSTTEETFSLGGCTLTVIAP
Oeu0461221 OeuXIP1;3   VISTIETDVKMPNLVLSILAAIIIAIVLLAVHPISGGHINPVISFSAALVGLISVSKALIYISAQCLGAALGALALKAVVSSTIEQTFSLGGCTLTVIAP
SlXIP1;1               VISTFESETKMPNLIMSILIAVVITILLLAVVPVSGGHINPVISFSAALVGIISMSRAIIYIVAQCVGAILGALALRAVVSSSIEDTFSLGGCTVTIIAP
PoptrXIP21             VISSYETQTKTPNLVMATLIAITIAILLLATFPISGGHINPAITLSAMFTGLITVSRAAIYILAQCIGAILGALALKAVVNSTIEQTFSLGGCTLKIVAP
                                           H2                                                         P1
                       ---              ------TM2-----------           --NPA1;LB--        ----------TM3------------

                           210       220       230       240       250       260       270       280       290       300
                             |         |         |         |         |         |         |         |         |         |
Oeu0100561 OeuXIP1;1   GPNGPITLGLETAQAFWLEIFCTFVFLFASIWMAYDHRQAKNLGLVLVFSIIGLVLGLLVFISTTVTAKKGYAGAGMNPARCLGAAIVRGGHLWDGHWVF
Oeu0206841 OeuXIP1;2   GLNGPATVGLETVQAFWLEIFCTFVFLFASIWMAYDPRQAKELGHVLIFSIVGVVLGLLVFISTTVTAKKGYAGAGMNPARCFGPAIVRGGHLWNVYWVF
Oeu0461221 OeuXIP1;3   GPNGLVTIGLETAQAFWLEIICTFVLLFASVWMAYDHRQAKALGRVLVFSIVGVVLGLLVFISTTVTAKKGYAGAGMNPARCFGAAVVRGGHLWNGHWIF
SlXIP1;1               GPNGPVIVGLETAQALWLEIFCTFVFLFASIWMAYDHRQAKALGHVTVLSIVGLVLGLLVFISTTVTAKKGYGGAGINPARCLGPAIIRGGHLWDGHWIF
PoptrXIP21             GPSGPVAIGLETGQALWLEIICTFVFLFSSIYIAFDRRQAIALGRVVFCSIIGLVVGLLVFISTTVTATKGYAGVGMNPARCLGPALVRGGHLWKGHWVF
                                                                        H5                           LE1  LE2 P2 P3         P4
                                 -----------TM4------------           ----------------TM5---------------     --NPA2;LE--  ---TM6---

                           310       320       330       340
                             |         |         |         |
Oeu0100561 OeuXIP1;1   WAGPAIACVAFYLYTKIIPRQHHHAKG-YGHDFFDTVKAMLGFVDH
Oeu0206841 OeuXIP1;2   WAGPTIACVAFYLYTQIIPCQHHRSKYSYDHDFYNVVKAIF-----
Oeu0461221 OeuXIP1;3   WVGPAIACVAFYLYTKIIPRQHYQAKF-YDHDFFNVVKAMF-----
SlXIP1;1               WVGPTIGCVAFYVYTKIIPTKHFLAEYGFKHDFVGVVKALSNV---
PoptrXIP21             WVGPIVASLTFSLYTKIIPREHLLGAESK-----------------
                       P5
                       -------------------
```

Figure S5C. Amino acid sequence alignment of the *Oeu*XIPs subfamily members from *Olea europeae* var. *sylvestris*. *Sl*XIP1;1 (Solyc10g054840.1; Reuscher et al., 2013) from Tomato and *Poptr*XIP2;1 (Potri.009G128500.1; Lopez et al, 2016) from Poplar were used as references. The highest residue similarities between MIP members are shown in red (100%), and green (>80%). Residues with similar physicochemical behaviors are shown in blue. The six transmembrane domains (TMH1-6) and the two hemi-helicoidal regions that include the «NPA» motives «NPA1» (in Loop B) and «NPA2» (in Loop E) (framed in purple) are underlined. The amino acids residues constituting the ar/R constriction region are shown with blue highlights and labeled H2, H5, LE1 and LE2, and residues constituting the Froger's positions are shown with yellow highlights and labeled P1 to P5.

```
                                       10        20        30        40        50        60        70        80        90
                                        |         |         |         |         |         |         |         |         |
AtTIP1;2                     -------MPTRNIAIGGVQEEVYHPNALRAALAEFISTLIFVFAGSGSGIAFNKITDNGATTPSGLVAAALAHAFGLFVAVSVGANISGG
SlTIP1;1                     -------MPINQITIGS-HEELRHPGALKAALAEFISTLIFVFAGQGSGMAFNKLTDGVAT-PAGLISASIAHAFGLFVAVSVGANISGG
OeuTIP1;3  Oeu0354791        MSSEFFNVPLRRVAFGH-RDEFRQPGAIKAALAEFFSTLIFVFAGEGSGMAYNKLTGNAPTSPSGLVAAAVAHGFALFVAVAVSANISGG
OeuTIP1;1  Oeu0585311        -------MPISRVAIGS-PAEFSQLDAIKAAVAEFISMLIFVFAGQGSGMAFAKLTDNESTTPAGLIAASIAHAFALFVAVSVGANISGG
OeuTIP1;4  Oeu0402901        -------MPISRIAVGS-LAEASQPEALKATLAEFISMLIFVFAGEGSGMAFAKLTNNGSTTPTGLIAEAIAHAFALFVAVSVGANISGG
OeuTIP1;5  Oeu0402891        -------MPISRIAVGS-LAEASQPEALKATLAEFISMLIFVFAGEGSGMAFAKLTNNGSTTPTGLIAEAIAHAFALFVAVSVGANISGG
OeuTIP1;2  Oeu0410141        -------MPFSRISVGS-PAEASHPDTLKAALAEFISMVIFVFAGEGSGMAFAKLTDGGSSTPAGLIAAAIAHAFALFVAVSVAANISGG
OeuTIP1;6  Oeu0332441        -------MPIYRIAIGT-PGEASHPDALKAALAEFFSMLIFVFAGQGSGMAFNKLSNDGSPNPSGLLAAALAHAFALFVAVSVGANISGG
OeuTIP1;7  Oeu0452371        MYSNSM-RSGNQIAVGR-LEEATQQDALKAALAEFISTLIFVFAGSGSGMAFNKLTDDGATTPAGLIAAAVAHAFALFVAVSVGANISGG
OeuTIP2;1  Oeu0240741        ---------MPAIAVGR-LDDSFSIGSLKAYVAEFISTLLFVFAGVGSAIAYNKLTADAALDPAGLVAVAICHGFALFVAVSIGANISGG
OeuTIP2;2  Oeu0240761        ---------MPAIAVGR-LDDSFSIGSLKAYVAEFISTLLFVFAGVGSAIAYNKLTADAALDPAGLVAVAICHGFALFVAVSIGANISGG
OeuTIP2;3  Oeu0539881        ---------MPTIAFGR-IDDSLSVGSLKAYLAEFISTLLFVFAGVGSAIAYNKLTYDAALDPAGLVAVAVCHGFALFVAVSIGANISGG
OeuTIP2;4  Oeu0023291        ---------MVKMTLGS-IGDSFSVPSLKAYLAEFIATLLFVFAGVGSAIAYNKLTSDAALDPPGLVAVAVAHAFALFVGVSMAANVSGG
OeuTIP2;5  Oeu0402071        ---------MVKINWGS-IGDSFSVTSLKAYIAEFIATLLFVFAGVGSAIAYNKLTSDAALDPPGLVAVAVAHAFALFVGVSMAANVSGG
OeuTIP3;1  Oeu0383871        ---------MPRGYTFGRAEEATHPDSARATLAEFLSMLVFVFAGEGSVLALDKMYKDTALGASSLVVIALAHALSLFAAVASSMNVSGG
OeuTIP4;1  Oeu0302291        ---------MAKIALGN-AREAFQSDCIQALIVEFICTFLFVFAGVGAAMATDKLNGDPLV---GLFFVAMAHALVVAVMISAGFRVSGG
AtTIP5;1                     ------MRRMIPTSFSSKFQGVLSMNALRCYVSEFISTFFFVLAAVGSVMSSRKLMAGDVSGPFGVLIPAIANALALSSSVYISWNVSGG
SlTIP5;1                     -----------MASLASRLQHSVTPNALRSYLAEFLSTFFFVFAAAGASMSTRKMVPDATSDPSSLVAIAVANAFALSVAVYISANISGG
OeuTIP5;1  Oeu0217081        -----------MASLKSRLEHTVTPTALRSYLAEFISTFFFVFASVGATMSTLKMMPDAASNSSGLVAIAVANAFALSVAVYIAYNISGG
OeuTIP5;2  Oeu0217091        -----------MASLKSRLEHTVTPTALRSYLAEFISTFFFVFASVGATMSTLKMMPDAASNSSGLVAIAVANAFALSVAVYIAYNISGG
OeuTIP5;3  Oeu0224791        -----------MASLKSRLEHSVTAIALRSYLAEFISTFFFVFAAAGATMSSRKMMPDAASDSSSLVAIAVANAFALSVTVYISANISGG
                                                                                                          H2
                                                    -----------TM1---------                -----------TM2---------
                                      100       110       120       130       140       150       160       170       180
                                        |         |         |         |         |         |         |         |         |
AtTIP1;2                     HVNPAVTFGVLLGGNITLLRGILYWIAQLLGSVAACFLLSFATGGEPIPAFGLSAGVGSLNALVFEIVMTFGLVYTVYATAVDPKNGSLG
SlTIP1;1                     HVNPAVTFGAFVGGNITLFRGILYIIAQLLGSTAACALLEFATGGMSTGSFALSAGVSVWNAFVFEIVMTFGLVYTVYATAVDPKKGDLG
OeuTIP1;3  Oeu0354791        HVNPAVTFGLFLGGNVSLFRGILYIIAQLLGSVVACLLLLFSTGGLETSAFALTAGVSVWNAFVFEIVMTFGLVYTVYATAVDPKKGEIG
OeuTIP1;1  Oeu0585311        HVNPAVTLGAFVGGHITLFRSIMYWIAQLLGSVIACLLLKFATGGLETSAFALSSGVTVWNAVIFEIVMTFGLVYTVYATAVDPKKGNLG
OeuTIP1;4  Oeu0402901        HVNPAVTFGAFVGGHITLIRSILYWIAQLLGSVVACLLLRLATGGLETSAFALSSGVTVWNAVVFEIVMTFGLVYTVYATAVDPKRGNLG
OeuTIP1;5  Oeu0402891        HVNPAVTFGAFVGGHITLIRSILYWIAQLLGSVVACLLLRLATGGLETSAFALSSGVTVWNAVVFEIVMTFGLVYTVYATAVDPKRGNLG
OeuTIP1;2  Oeu0410141        HVNPAVTFGAFVGGHLSLFKTILYWIAQLLGAIVACLLLKFSTGGLETSAFALSTGVSSWNALVFEIVMTFGLVYTVYATAVDPKRGDIG
OeuTIP1;6  Oeu0332441        HVNPAVTFGAFIGGNITLLRSILYWIAQLLGSVVACILLKFATGGLETSAFALSSGVSVWNALVFEIVMTFGLVYTVYATAVDPKKGNLG
OeuTIP1;7  Oeu0452371        HVNPAVTFGAFVGGNISLIRGILYWIAQLLGSVVACLLLKFATGGLTTAAFSLSAGVNVWNAFVLEIVMTFGLVYTVYATAVDPKRGSLG
OeuTIP2;1  Oeu0240741        HVNPAVTFGLAVGGQITLLTGLFYWIAQLLGSIVACYLLVFVTGGLAIPIHGVGAGVGAFEGVVMEIVITFALVYTVYATAADPKKGSLG
OeuTIP2;2  Oeu0240761        HVNPAVTFGLAVGGQITLLTGLFYWIAQLLGSIVACYLLVFVTGGLAIPIHGVGAGVGAFEGVVMEIVITFALVYTVYATAADPKKGSLG
OeuTIP2;3  Oeu0539881        HVNPAVTFGLAVGGQITILTGLFYWIAQLLGSIVACFLLKFVTGGLAIPTHSMAAGVGAIEGVVMEIVITFALVYTVYATAVDPKKGSLG
OeuTIP2;4  Oeu0023291        HLNPAVTFGLAIGGNITIITGIFYWIAQLLGSTVACLLLKFVTNGLAIPTHAVASGMNAVEGVVMEIVITFALVYTVYATAADPKKGSLG
OeuTIP2;5  Oeu0402071        HLNPAVTFGLAIGGNITIITGLFYWIAQLLGSIVACLLLKFVTGGLAIPTHAVASGMNAVEGVVMEIVITFALVYTVYATAADPKKGSLG
OeuTIP3;1  Oeu0383871        HVNPAVTFGALVGGRVSVLRAIYYWIAQLLGAVLAAILLRLATDGLRPRGFSVADGVGEWNALVMEIVLTFGLVYTVYATAIDPKRGSLG
OeuTIP4;1  Oeu0302291        HINPAVTLGLLAGGHITVVRSILYWIDQLLASTAACALLKYLTGGLTIPVHTLATGVDFAQGVIMEIVLTFSLLFTVYATIVDPKKGFLE
AtTIP5;1                     HVNPAVTFAMAVAGRISVPTAMFYWTSQMIASVMACLVLKVTVMEQHVPIYKIAGEMTGFGASVLEGVLAFVLVYTVFT-ASDPRRGLPL
SlTIP5;1                     HVNPAVTFGMAVGGHISIPMSIFYWISQMIGSVTACLLLKFTN--QQVPTHGIPQEMTGFGAAVLEGVMTFGLVYTVYA-AADPRRCVHA
OeuTIP5;1  Oeu0217081        HVNPAVTFGMAIGGHISIPMAIFYWISQTLGSVIACILLKVTTVGQHVPTHSIPQEMTGFGAAILEGVMTFTLVYTVYA-AADPRRGSMS
OeuTIP5;2  Oeu0217091        HVNPAVTFGMAIGGHISIPMAIFYWISQTLGSVIACILLKVTTVGQHVPTHSIPQEMTGFGAAILEGVMTFTLVYTVYA-AADPRRGSMS
OeuTIP5;3  Oeu0224791        HVNPAVTFGMVVGGHISIPMAIFYSISQTFGSVMACLLLKATTAGQHVPTHAIEHEMTGFGGAVLEGVMTFALVYTVYV-ASNLRPVSAG
                                                                           P1
                                -NPA;LB--              ----------TM3----------             -----------TM4----------
```

```
                              190       200       210       220       230       240       250       260
                                |         |         |         |         |         |         |         |
AtTIP1;1                  TIAPIAIGFIVGANILAGGAFSGASMNPAVAFGPAVVSWTWTNHWVYWAGPLIGGGLAGIIYDFVFIDENAHEQLPTTDY-------
SlTIP1;1                  VIAPIAIGFIVGANILAGGAFTGASMNPAVSFGPSLVSWTWTHQWVYWAGPLIGGGLAGFIYEFIFISHT-HEQIPSGDF-------
OeuTIP1;3 Oeu0354791      IIAPIAIGFIVGANILAGGAFTGASMNPAVSFGPALVSFTWTHHWVYWAGPLIGGGLAGIVYEVFFVNHS-HEPLPGSEY-------
OeuTIP1;1 Oeu0585311      IIAPIAIGFIVGANILAGGAFDGASMNPAVSFGPAVVSWTWDSHWVYWLGPFVGAGIAALVYEILFINQS-HQQLPTSAEY------
OeuTIP1;4 Oeu0402901      IIAPIAIGFIVGANILAGGAFDGASMNPAVSFGPAVVSWTWDNHWVFWLGPFVGAGIAALIYDILFINQS-HEQLRTSAEY------
OeuTIP1;5 Oeu0402891      IIAPIAIGFIVGANILAGGAFDGASMNPAVSFGPAVVSWTWDSHWVFWLGPFVGAGIAALIYEILFINQS-HEQLRTSAEY------
OeuTIP1;2 Oeu0410141      IIAPIAIGFIVGANILAGGAFDGASMNPAVSFGPAVVSWTWDSHWVYWLGPFVGAGIAAIVYEIVFINQT-THEPLPSSEY------
OeuTIP1;6 Oeu0332441      NIAPIAIGFIVGANILAGGPFDGASMNPAVSFGPAVVSLSVFDQKDRIRLSIKEK--------------------------------
OeuTIP1;7 Oeu0452371      TIAPIAIGFIVGANILAGGAFDGASMNPAVSFGPAVVSWTWDNHWVYWAGPLIGGGLAGLIYEFFFINQT-HEQLPTTDH-------
OeuTIP2;1 Oeu0240741      TIAPIAIGFIVGANILAAGPFSGGSMNPARSFGPAVASGNFSGIWIYWVGPLIGGGLAGLVYAYVFMSHE-HAPLPTDF--------
OeuTIP2;2 Oeu0240761      TIAPIAIGFIVGANILAAGPFSGGSMNPARSFGPAVASGNFSGIWIYWVGPLIGGGLAGLVYAYVFMSHE-HAPLPTDF--------
OeuTIP2;3 Oeu0539881      TIAPIAIGFIVGANILAAGPFSGGSMNPARSFGPAVASGDFADNWIYWVGPLIGGGLAGLVYAYVFMVHE-HVPLVSEF--------
OeuTIP2;4 Oeu0023291      IIAPIAIGFIVGANILAAGPFSGGSMNPARSFGPAVAAGDFSQNWIYWVGPLIGGGLAGLVYGYVFIA-S-YAPFPAQEGYA-----
OeuTIP2;5 Oeu0402071      TIAPIAIGFIVGANILAAGPFSGGSMNPARSFGPAVAAVDFSQNWIYWVGPLIGGGLAGLVYGDVFIG-S-YAPLPVQEEYP-----
OeuTIP3;1 Oeu0383871      TIAPLAIAFIVGANVLVGGPFSGASMNPARAFGPALVGWRWKDHWVYWLGPFVGAALAGVIYEFGVIQTETPHPTHHLHQPLAPEDY
OeuTIP4;1 Oeu0302291      GMGPLLTGLVVGANVMAGGPFSGASMNPARSFGPALVSGDWTDHWVYWVGPLIGGGLAGYIYENFFIVRSHVQLSGEEVF-------
AtTIP5;1                  AVGPIFIGFVAGANVLAAGPFSGGSMNPACAFGSAMVYGSFKNQAVYWVGPLLGGATAALVYDNVVVPVEDDRGSSTGDAIGV----
SlTIP5;1                  AIGPLAIGLMLGANVMASGPFTGGSMNPAYSFGSAVVKGSFGNQAVYWIGPFIGAAIAGLVYDNVVFPLQV-TESLRGIGGGIVAV-
OeuTIP5;1 Oeu0217081      IIGPLAIGLVSGANVLASGPFTGGSMNPAYSFGSALVGGSFKNQAVYWIGPLIGAATAGILYEHVVFPAQQEPDSLRGIPHGVEF—
OeuTIP5;2 Oeu0217091      IIGPLAIGLVSGANVLASGPFTGGSMNPAYSFGSALVGGSFKNQAVYWIGPLIGAATAGILYEHVVFPAQQEPDSLRGIPHGVEF—
OeuTIP5;3 Oeu0224791      AIGPLAIGLIAGANVLASGPFTGGSMNPAYAFGSAIVGGSFKNQAVYWVGPLFGAAIAGILHDNVVCPAPV-PDSFRGNADGVGV—
                                         H5           LE1 LE2P2  P3             P4P5
                          ---------TM5---------      -NPA;LE--          ----------TM6----------
```

Figure S5B. Amino acid sequence alignment of the *Oeu*TIPs subfamily members from *Olea europeae* var. *sylvestris*. *Sl*TIP1;1 (Solyc06g074820.1) and *Sl*TIP5;1 (Solyc03g093230.1) (Reuscher et al., 2013) from Tomato and *At*TIP1;1 (At2g36830) and *At*TIP5;1 (At3g47440) from *Arabidopsis thaliana* were used as references. The highest residue similarities between MIP members are shown in red (100%), and green (>80%). Residues with similar physicochemical behaviors are shown in blue. The six transmembrane domains (TMH1-6) and the two hemi-helicoidal regions that include the «NPA» motives «NPA1» (in Loop B) and «NPA2» (in Loop E) (framed in purple) are underlined. The amino acids residues constituting the ar/R constriction region are shown with blue highlights and labeled H2, H5, LE1 and LE2, and residues constituting the Froger's positions are shown with yellow highlights and labeled P1 to P5.

```
                              10        20        30        40        50        60        70        80        90
                               |         |         |         |         |         |         |         |         |
SlSIP1;1               ---MGVIKAAIADGLLTFLWVFCSSNIGVSTYFIASYFGIVN-EIPSLFITTLIVFVIFLMFDFLGDVLGGAGFNPTGNAAFYAAGLG-D
SlSIP1;2               ---MGAVKAAVGDFVLTLMWVFCSSTLGIFTYLIATAFGIAQ-GMASLFITTVLLFMLFFVFGIIGDALGGAAFNPAGTAAFYAAGVG-K
AtSIP1;1               ---MGVLKSAIGDMLMTFSWVVLSATFGIQTAAIISAGDFQAITWAPLVILTSLIFVYVSIFTVI---FGSASFNPTGSAAFYVAGVP-G
AtSIP1;2               ---MSAVKSALGDMVITFLWVILSATFGIQTAAIVSAVGFHGITWAPLVISTLVVFVSISIFTVIGNVLGGASFNPCGNAAFYTAGVS-S
OeuSIP1;1 Oeu0502921   ---MGAIKAAVADGVLTFMWVFCASTLGALTFVVASALGVSQ-GLPTLLVTTVLVFVLLFVFGIIGDFLGGATFNPTAIAAFYAAGIGGA
OeuSIP1;2 Oeu0147232   ---MRAIKVAIADAIVTCLWVFCASSLGALTFVVASAFGVSQ-GLPTLLITTFLLFVLLFVFGFIGDALGGATFNPTGPAAFYAAGVGGT
AtSIP2;1               ---MGRIGLVVTDLVLSFMWIWAGVLVNILVHGVL---GFSRTDPSGEIVRYLFSIISMFIFAYLQQATKGGLYNPLTALAAGVSG-GFS
SlSIP2;1               -MGVSRRSLVISDFIMSFMWVWSSVLIKMFVHKIL---GYGAHDL-------------------------KGGAYNPLTILSGAISG-DLT
OeuSIP2;1 Oeu0607491   MAAVGGRLLLVSDFFMSFMWVWSSVLIKIFVYKIL---GYGTHDLKGEILRYGISLVNMFFFAYLAKATKGGAYNPLTILSSAISG-DFS
OeuSIP2;2 OeuChr11     MAAVRGRRLLVSDFLMSFMWVWSSVLIKIFVYKIL---DYGSHDMKGEILRYGISIVNMFFFAFLAKATKGGAYNPLTVLSSAISG-NFS
                                ------------TM1---------------        ---------H2---TM2-------      -NPA;LB---  ----

100       110       120       130       140       150       160       170       180
                               |         |         |         |         |         |         |         |         |
SlSIP1;1               DSLVSAAVRCPAQVAGAVAGSLALVELIPKHYHHMLDG-PALKVDVQTGAIAEGVLTFVITFMIFVIVLRGPESVLLKNWLLTMVTLPLV
SlSIP1;2               DSLFTVATRFPAQAAGAVAGAVAILEVIPTQYKHMLGG-PSLKVDLHNGAIAEGILTFVMTFLVFIIVLKGPKSALLKNWLLAMSTVTMV
AtSIP1;1               DTLFSLAIRLPAQAIGAAGGALAIMEFIPEKYKHMIGG-PSLQVDVHTGAIAETILSFGITFAVLLIILRGPRRLLAKTFLLALATISFV
AtSIP1;2               DSLFSLAIRSPAQAIGAAGGAITIMEMIPEKYKTRIGGKPSLQFGAHNGAISEVVLSFSVTFLVLLIILRGPRKLLAKTFLLALATVSVF
OeuSIP1;1 Oeu0502921   DSLFSAALRFPAQAAGAVGGALAIMEVMPMQYKHMLGG-PSLKVDLHTGAIAEGVLTFTITFAVLLIVLRGPSNPLVKNWLLAMATVSAV
OeuSIP1;2 Oeu0147232   ESLVSAAVRFPAQAAGAVGGALGILEVMPVQYKHMLDC-PSLKVDLNTGAIAEGVLTFIITFAVLYIVLRGPTNPFVKNWLLAMSTVALV
AtSIP2;1               SFIFSVFVRIPVEVIGSILAVKHIIHVFPE-----IGKGPKLNVAIHHGALTEGILTFFIVLLSMGLTRKIPGSFFMKTWIGSLAKLTLH
SlSIP2;1               NFIFTVAARIPSQVFGSITGVRFIIAAFPN-----IGRGPVLSIDIHRGALTEGILTFAIVSISLGLSRRSRASTFMKTWISSLSKLTLH
OeuSIP2;1 Oeu0607491   NFLFTVGARIPIQVLGSIYGVRFILAAFPE-----IGRGPRLNVDIARGALTEGLLTFGIVIISQGLSRKIPGSFFMKTWISSVSKLTLH
OeuSIP2;2 OeuChr11     NFLFTVGARIPVQVLGSIYGVRLILAAFPE-----IGRGPRLNVDITRGALTEGFLTFGIVIISQGLSRKLPGSFFMKTWISSVSKLTLH
                                P1                                                                         H5
                       ---------TM3--------                       ------------TM4-----------     ------TM5----

                              190       200       210       220       230       240       250
                               |         |         |         |         |         |         |
SlSIP1;1               LAGSNFTGPSMNPANAFGWAYLSNTHKTLEHFYVYWISPFIGAILAAWIFRVLFPPPVEQ-KPQKQKRN-----
SlSIP1;2               VAGSKYTGPSMNPANAFGWAYINNMHNTWEQFYVYWICPFVGAIMAAWTFRAVFPAPAKKKKPQKKKRN-----
AtSIP1;1               VAGSKYTGPAMNPAIAFGWAYMYSSHNTWDHIYVYWISSFVGALSAALLFRSIFPPPRPQKKKQKKA-------
AtSIP1;2               VVGSKFTRPFMNPAIAFGWAYIYKSHNTWDHFYVYWISSYTGAILSAMLFRIIFPAPPLVQKKQKKA-------
OeuSIP1;1 Oeu0502921   VAGSSYTGPSMNPANAFGWAYVYNKHNTWEQFYVYWICPFTGAILAAWTFRFLFPPPTKQDKAKKA--------
OeuSIP1;2 Oeu0147232   VAGSSYTGPSMNPANAFGWAYVNNTHKTWEQFYVYWICPFIGAILAAWVFRFLFPPPPVK-KQKKA--------
AtSIP2;1               ILGSDLTGGCMNPAAVMGWAYARGEHITKEHLLVYWLGPVKATLLAVWFFKVVFKPLTEEQEKPKA-KSE----
SlSIP2;1               ILGSDLTGGCMNPASVMGWAYARGDHITKEHIHVYWLAPIQATLLAVWTFNLLVSPSKDKEAKKTEKKSE----
OeuSIP2;1 Oeu0607491   ILGSDLTGGCMNPASVMGWAFAHGEHISKEHLIVYWLAPMEATLAAVWAFRLIVRPKKDETLKAKSVKSDCKNS
OeuSIP2;2 OeuChr11     MLGSDLTGGCMNPASVMGWAFTRGEHISKEHLIVYWLAPMEATLAAVWAFRVVVRPENDKTPKVKSGKSE----
                                LE1 LE2P2  P3              P4P5
                                    --NPA;LB--           -------------TM1-------
```

Figure S5E. Amino acid sequence alignment of the *Oeu*SIPs subfamily members from *Olea europeae* var. *sylvestris*. *Sl*SIP1;1 (Solyc12g0196.1), *Sl*SIP1;2 (Solyc10g0784.1) and *Sl*SIP2;1 (Solyc01g0567.1) (Reuscher et al., 2013) from Tomato, and *At*SIP1;1 (AT3G04090), *At*SIP1;2 (AT5G18290) and *At*SIP2;1 (AT3G56950) (Reuscher et al., 2013) from *Arabidopsis thaliana* were used as references. The highest residue similarities between MIP members are shown in red (100%), and green (>80%). Residues with similar physicochemical behaviors are shown in blue. The six transmembrane domains (TMH1-6) and the two hemi-helicoidal regions that include the «NPA» motives «NPA1» (in Loop B) and «NPA2» (in Loop E) (framed in purple) are underlined. The amino acids residues constituting the ar/R constriction region are shown with blue highlights and labeled H2, H5, LE1 and LE2, and residues constituting the Froger's positions are shown with yellow highlights and labeled P1 to P5.
