# Supplementary material for: Genome Wild Analysis and Molecular Understanding of the Aquaporin Diversity in Olive Trees (Olea Europaea L.)
Source: Int J Mol Sci. 2020 Jun 11;21(11):4183. doi: 10.3390/ijms21114183 (PMC7312470; doi:10.3390/ijms21114183)

Figure S6. Structuration of the 15 motives identified in the 52 full-length *Oeu*AQP protein sequences (**Figure 2**). Motif analysis was performed by the MEME web server. Details in amino acid constituting these motives are listed in the legend, and residue occurrences are provided on page 2 of the file.

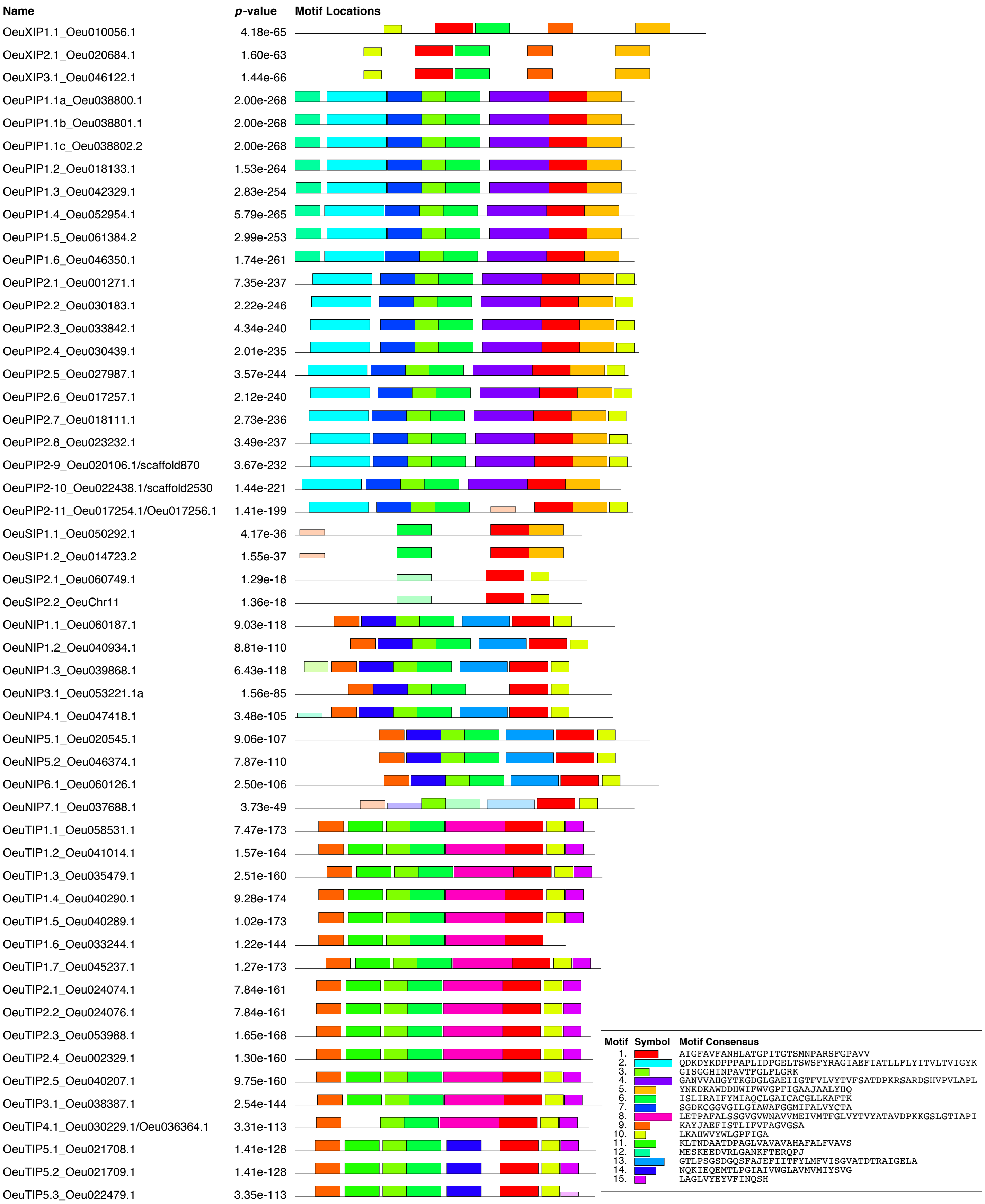

Figure S6, suite. Amino acids constituting the fifteen motifs of the 50 full-length *Oeu*AQP protein sequences (**Figure 2**). Motif analysis was performed by the MEME web server.

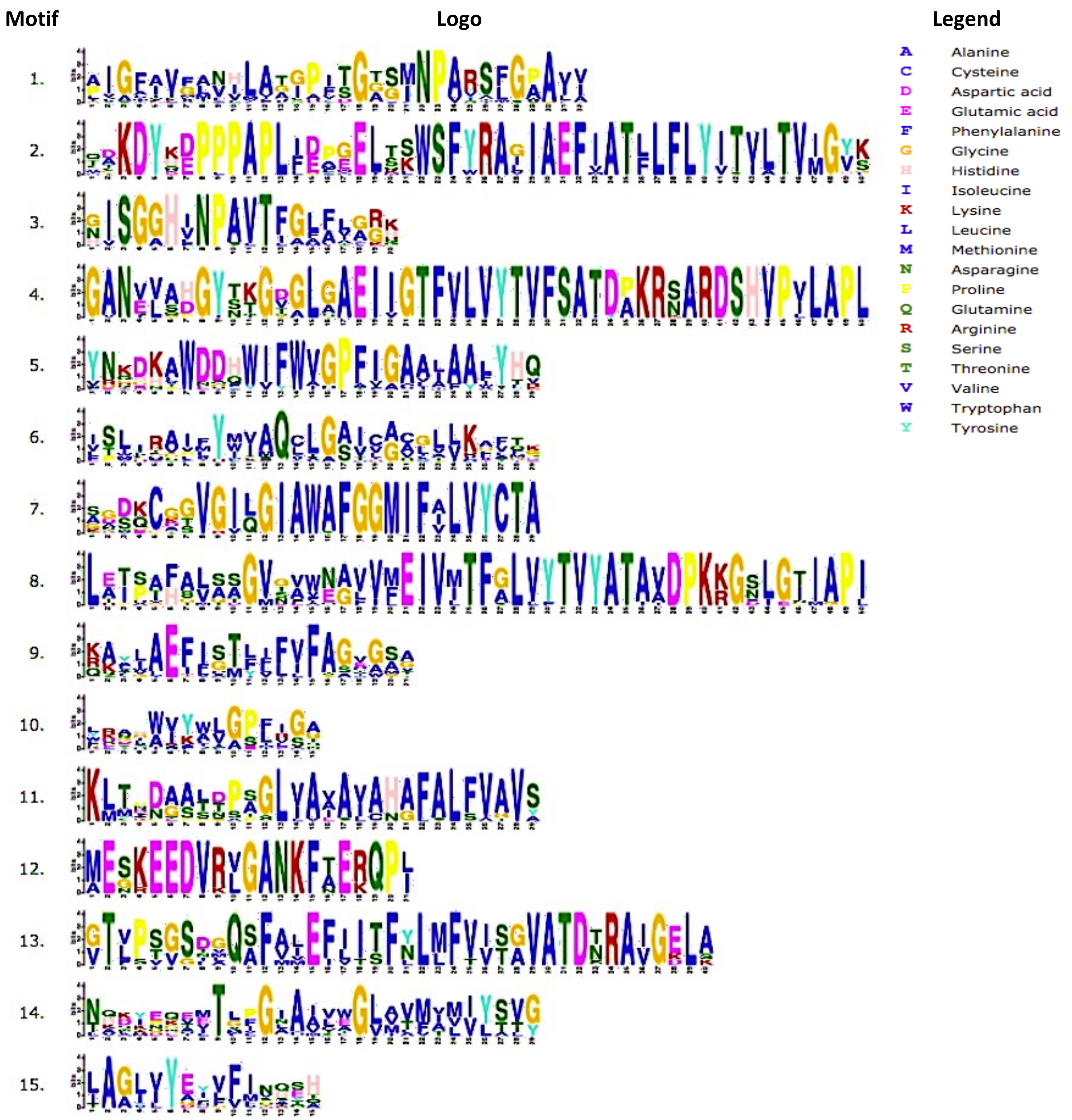

Supplement: Supplementary file 1 [file ijms-21-04183-s001.zip › ijms-821530_Supplementary_files/Figure S6_Faize_OeuAQP_protein Motifs.pdf]
