# Supplementary material for: Genome Wild Analysis and Molecular Understanding of the Aquaporin Diversity in Olive Trees (Olea Europaea L.)
Source: Int J Mol Sci. 2020 Jun 11;21(11):4183. doi: 10.3390/ijms21114183 (PMC7312470; doi:10.3390/ijms21114183)
Supplement: Supplementary file 1 [file ijms-21-04183-s001.zip › ijms-821530_Supplementary_files/Figure S7_Faize_OeuOleurAQP_Compil.pdf]

Figure S7. Comparison of protein structural features, evolution pressure, and exon-intron structures of full-length *Oeu*AQP and *Oleur*AQP genes from the wild *Olea europaea* var. *sylvestris* and the cultivated *Olea europaea* cv. Picual, respectively.

*Oeu*AQPs<br>*Oleur*AQPs	*Oeu*- & *Oleur*AQP ar/R / Froger's / NPA1-2 Motifs*	*Oeu*- & *Oleur*AQP SDP motifs**	*Oeu*/*Oleur* AQP Protein similarity (Ka/Ks)	*Oleur*AQP similarities CDS - Protein***	Ka/Ks *Oleur*AQP duplicates***
**Plasma membrane intrinsic proteins (PIPs)**					
*Oeu*PIP1;1a/b/C<br>*Oleur*PIP1;1	F-H-T-R / G-S-A-F-W / NPA-NPA	Bo CO2 H2O2 Ur	100% (0,0010)	-	-
*Oeu*PIP1;2<br>*Oleur*PIP1;2a<br>*Oleur*PIP1;2b	F-H-T-R / G-S-A-F-W / NPA-NPA	Bo CO_2 H_2O_2 Ur	100% (0,0010)<br>100% (0,0010)	99,42% - 100%	0,001
*Oeu*PIP1;3<br>*Oleur*PIP1;3a<br>*Oleur*PIP1;3b	F-H-T-R / M-S-A-F-W / NPA-NPA<br>M-S-A-F	Bo CO_2 H_2O_2 Ur	96,52% (0,5323)<br>97,329% (0,3028)	94,76% - 93,49%	0,4137
*Oeu*PIP1;4<br>*Oleur*PIP1;4a<br>*Oleur*PIP1;4b	F-H-T-R / G-S-A-F-W / NPA-NPA	Bo CO_2 H_2O_2 Ur	99,65% (0,0961)<br>99,3% (0,1237)	98,65% - 99,65%	0,0534
*Oeu*PIP1;5<br>*Oleur*PIP1;5a<br>*Oleur*PIP1;5b	F-H-T-R / G-S-A-F-W / NPA-NPA	Bo CO_2 H_2O_2 Ur	100% (0,3641)<br>94,8% (0,1383)	93,70% - 94,80%	0,1784
*Oeu*PIP1;6a<br>*Oleur*PIP1;6a<br>*Oleur*PIP1;6b	F-H-T-R / G-S-A-F-W / NPA-NPA	Bo CO_2 H_2O_2 Ur	100% (0,6987)<br>99,65% (0,6987)	99,53% - 99,65%	0,091
*Oeu*PIP2;1<br>*Oleur*PIP2;1a<br>*Oleur*PIP2;1b	F-H-T-R / Q-S-A-F-W / NPA-NPA	H_2O_2 Ur	99,65% (0,3717)<br>99,65% (0,3717)	99,31% - 100%	0,001
*Oeu*PIP2;2<br>*Oleur*PIP2;2a<br>*Oleur*PIP2;2b	F-H-T-R / Q-S-A-F-W / NPA-NPA	H_2O_2 Ur<br>Ur<br>Ur	99,64% (0,0010)<br>99,64% (0,0010)	99,98% - 100%	0,001
*Oeu*PIP2;3<br>*Oleur*PIP2;3a<br>*Oleur*PIP2;3b	F-H-T-R / Q-S-A-F-W / NPA-NPA	H_2O_2 Ur	100% (0,1361)<br>98,96% (0,1780)	98,65% - 99,96%	0,178
*Oeu*PIP2;4<br>*Oleur*PIP2;4	F-H-T-R / Q-S-A-F-W / NPA-NPA	H_2O_2 Ur	99,26% (0,5108)	-	-
*Oeu*PIP2;5<br>*Oleur*PIP2;5	F-H-T-R / Q-S-A-F-W / NPA-NPA	H_2O_2 Ur	100% (0,0010)	-	-
*Oeu*PIP2;6<br>*Oleur*PIP2;6a<br>*Oleur*PIP2;6b	F-H-T-R / Q-S-A-F-W / NPA-NPA<br>-H-T-R / Q-S-A-F-W / NPA-NPA<br>-H-T-R / Q-S-A-F-W / NPA-NPA	H_2O_2 Ur	99,31% (0,1710)<br>99,65% (0,1468)	99,19% - 99,65%	0,0666
*Oeu*PIP2;7<br>*Oleur*PIP2;7	F-H-T-R / Q-S-A-F-W / NPA-NPA	H_2O_2 Ur	99,65% (0,1799)	-	-
*Oeu*PIP2;8<br>*Oleur*PIP2;8a<br>*Oleur*PIP2;8b	F-H-T-R / M-S-A-F-W / NPA-NPA	H_2O_2 Ur	100% (0,0010)<br>99,29% (0,1073)	99,30% - 99,29%	0,1703
*Oeu*PIP2;9<br>*Oleur*PIP2;9a<br>*Oleur*PIP2;9b	F-H-T-R / M-S-A-F-W / NPA-NPA	H_2O_2 Ur	100% (0,0010)<br>99,65% (0,1388)	99,53% - 99,65%	0,1388
*Oeu*PIP2;10<br>*Oleur*PIP2;10	F-H-T-R / Q-S-A-F-W / NPA-NPA	H_2O_2 Ur	98,85% (1,2672)	-	-
*Oeu*PIP2;11	F-H-T-R / Q-S-A-F-W / NPA-NPA	H_2O_2 Ur	-	-	-
*Oleur*PIP2;12	F-H-T-R / M-S-A-F-W / NPA-NPA	H_2O_2 Ur	-	-	-
**Tonoplast intrinsic proteins (TIPs)**					
*Oeu*TIP1;1<br>*Oleur*TIP1;1	H-I-A-V / T-S-A-Y-W / NPA-NPA	Ur	100% (0,4860)	-	-
*Oeu*TIP1;2<br>*Oleur*TIP1;2a<br>*Oleur*TIP1;2b	H-I-A-V / T-S-A-Y-W / NPA-NPA	Ur	100% (0,0010)<br>99,6% (0,0520)	98,42% - 99,60%	0,0427
*Oeu*TIP1;3<br>*Oleur*TIP1;3a<br>*Oleur*TIP1;3b<br>*Oleur*TIP1;3c<br>*Oleur*TIP1;3d<br>*Oleur*TIP1;3e	H-I-A-V / T-S-A-Y-W / NPA-NPA	Ur	99,22% (0,1299)<br>99,22% (0,1147)<br>98,84% (0,1656)<br>91,47% (0,1220)<br>92,25% (0,1142)	99,87% - 99,22%*<br>99,74% - 99,61%*<br>88,93% - 92,25%*<br>89,45% - 93,02%*	0,0010*<br>0,4009*<br>0,1083*<br>0,1004*
*Oeu*TIP1;4	H-I-A-V / T-S-A-F-W / NPA-NPA	Ur	-	-	-
*Oeu*TIP1;5<br>*Oleur*TIP1;5	H-I-A-V / T-S-A-F-W / NPA-NPA	Ur	99,21% (0,1000)	-	-
*Oeu*TIP1;6<br>*Oleur*TIP1;6	H-I-A-V / T-S-A-Y-W / NPA-NPA	Ur	92,95% (1,2712)	-	-
*Oeu*TIP1;7<br>*Oleur*TIP1;7	H-I-G-R / T-S-A-Y-W / NPA-NPA	H_2O_2 Ur	97,61% (1,5809)	-	-
*Oeu*TIP2;1<br>*Oleur*TIP2;1	H-I-G-R / T-S-A-Y-W / NPA-NPA	H_2O_2 Ur	100% (0,3550)	-	-
*Oeu*TIP2;2	H-I-G-R / T-S-A-Y-W / NPA-NPA	H_2O_2 Ur	-	-	-
*Oeu*TIP2;3<br>*Oleur*TIP2;3a<br>*Oleur*TIP2;3b	H-I-G-R / T-S-A-Y-W / NPA-NPA	Ur	100% (0,0010)<br>100% (0,0010)	99,97% - 100%	0,001
*Oeu*TIP2;4<br>*Oleur*TIP2;4	H-I-G-R / T-S-A-Y-W / NPA-NPA	Ur	99,2% (0,1298)	-	-
*Oeu*TIP2;5<br>*Oleur*TIP2;5a<br>*Oleur*TIP2;5b	H-I-G-R / T-S-A-Y-W / NPA-NPA	Ur	100% (0,0010)<br>99,59% (0,1018)	99,05% - 99,59%	0,0538
*Oeu*TIP3;1<br>*Oleur*TIP3;1a<br>*Oleur*TIP3;1b<br>*Oleur*TIP3;1c	H-V-A-R / T-A-A-Y-W / NPA-NPA	Ur	100% (0,0010)<br>98,84% (0,1019)<br>89,84% (0,1433)	97,81% - 98,84%*<br>86,77% - 89,84%*	0,1019*<br>0,1433*
*Oeu*TIP4;1<br>*Oleur*TIP4;1	H-V-A-R / T-S-A-Y-W / NPA-NPA	Ur	100% (0,0010)	-	-
*Oeu*TIP5;1<br>*Oleur*TIP5;1	N-V-G-Y / T-S-A-Y-W / NPA-NPA<br>-S-A-Y-W	Ur	99,6% (0,3778)	-	-
*Oeu*TIP5;2<br>*Oleur*TIP5;2	N-V-G-Y / T-S-A-Y-W / NPA-NPA<br>-S-A-Y-W / NPS	Ur	96,76% (0,3255)	-	-
*Oeu*TIP5;3<br>*Oleur*TIP5;3a<br>*Oleur*TIP5;3b	N-V-G-Y / T-A-A-Y-W / NPA-NPA	H_2O_2 Ur	100% (0,4970)<br>99,6% (0,1041)	99,34% - 99,60%	0,1041
**Uncharacterized *X* intrinsic proteins (XIPs)**					
*Oeu*XIP1;1<br>*Oleur*XIP1;1a<br>*Oleur*XIP1;1b	I-L-A-R / V-C-A-F-W / NPV-NPA	H_2O_2	100% (0,0010)<br>99,41% (0,1263)	99,61% - 99,41%	0,2411
*Oeu*XIP1;2<br>*Oleur*XIP1;2a<br>*Oleur*XIP1;2b	A-L-A-R / V-C-P-F-W / NPV-NPA	H_2O_2 Ur	100% (0,1199)<br>99,79% (0,1973)	99,73% - 99,79%	0,325
*Oeu*XIP1;3<br>*Oleur*XIP1;3	A-L-A-R / V-C-A-F-W / NPV-NPA	H_2O_2 Ur	94,01% (1,1671)	-	-
**Nodulin-26 like intrinsic proteins (NIPs)**					
*Oeu*NIP1;1<br>*Oleur*NIP1;1	W-V-A-R / F-S-A-Y-I / NPA-NPA	H_2O_2 Ur	99,61% (0,2954)	-	-
*Oeu*NIP1;2<br>*Oleur*NIP1;2	C-I-A-R / F-S-A-Y-I / NPA-NPA	Ur	100% (0,7996)	-	-
*Oeu*NIP1;3<br>*Oleur*NIP1;3	W-V-A-R / F-S-A-Y-I / NPA-NPV	H_2O_2 Ur	89,76% (0,8785)	-	-
*Oleur*NIP1;4	W-V-A-R / F-S-A-Y-I / NPA-NPV	H_2O_2 Ur	-	-	-
*Oeu*NIP3;1<br>*Oleur*NIP3;1	W-/-A-R / F-S-A-Y-V / NPA-NPA	Ur	100% (0,0010)	-	-
*Oeu*NIP4;1<br>*Oleur*NIP4;1a<br>*Oleur*NIP4;1b	W-V-A-R / F-S-A-Y-I / NPA-NPA	Ur	99,63% (0,3973)<br>99,25% (0,1497)	98,67% - 98,88%	0,2184
*Oeu*NIP5;1<br>*Oleur*NIP5;1a<br>*Oleur*NIP5;1b	A-I-G-R / F-T-A-Y-L / NPS-NPV	Bo Ur<br>Bo Ur<br>Bo	99,33% (0,4567)<br>99,33% (0,2268)	99,77% - 100%	0,001
*Oeu*NIP5;2<br>*Oleur*NIP5;2	A-I-G-R / F-T-A-Y-L / NPS-NPV<br>F	Bo Ur	96,98% (0,3559)	-	-
*Oleur*NIP5;3	A-I-G-R / F-T-A-Y-L / NPS-NPV	Bo Ur	-	-	-
*Oeu*NIP6;1<br>*Oleur*NIP6;1	T-I-A-R / F-T-A-Y-L / NPS-NPV<br>NPA	Bo	90,67% (0,3093)	-	-
*Oeu*NIP7;1<br>*Oleur*NIP7;1a<br>*Oleur*NIP7;1b	A-V-G-R / Y-S-A-Y-L / NPA-NPA<br>I<br>I	Ur<br>-<br>-	99,22% (0,2690)<br>99,43% (1,3207)	99,52% - 92,11%	1,156
**Small basic intrinsic proteins (SIPs)**					
*Oeu*SIP1;1<br>*Oleur*SIP1;1a<br>*Oleur*SIP1;1b	V-T-P-N / F-A-A-Y-W / NPT-NPA	-	100% (0,0010)<br>100% (0,0010)	100% - 100%	0,001
*Oleur*SIP1;3	-A-A-Y-W	-	-	-	-
*Oeu*SIP1;2<br>*Oleur*SIP1;2	V-T-P-N / F-A-A-Y-W / NPT-NPA	-	100% (0,3476)	-	-
*Oeu*SIP2;1<br>*Oleur*SIP2;1	S-K-G-S / I-V-A-Y-W / NPL-NPA	-	100% (0,2534)	-	-
*Oeu*SIP2;2<br>*Oleur*SIP2;2	S-K-G-S / I-V-A-Y-W / NPL-NPA	-	100% (0,0102)	-	-

Oeu- & *Oleur*AQP Exon Intron genomic structures (Exon, red boxes; Intron, black line; Gene orientations indicated (3') in the x-axis)

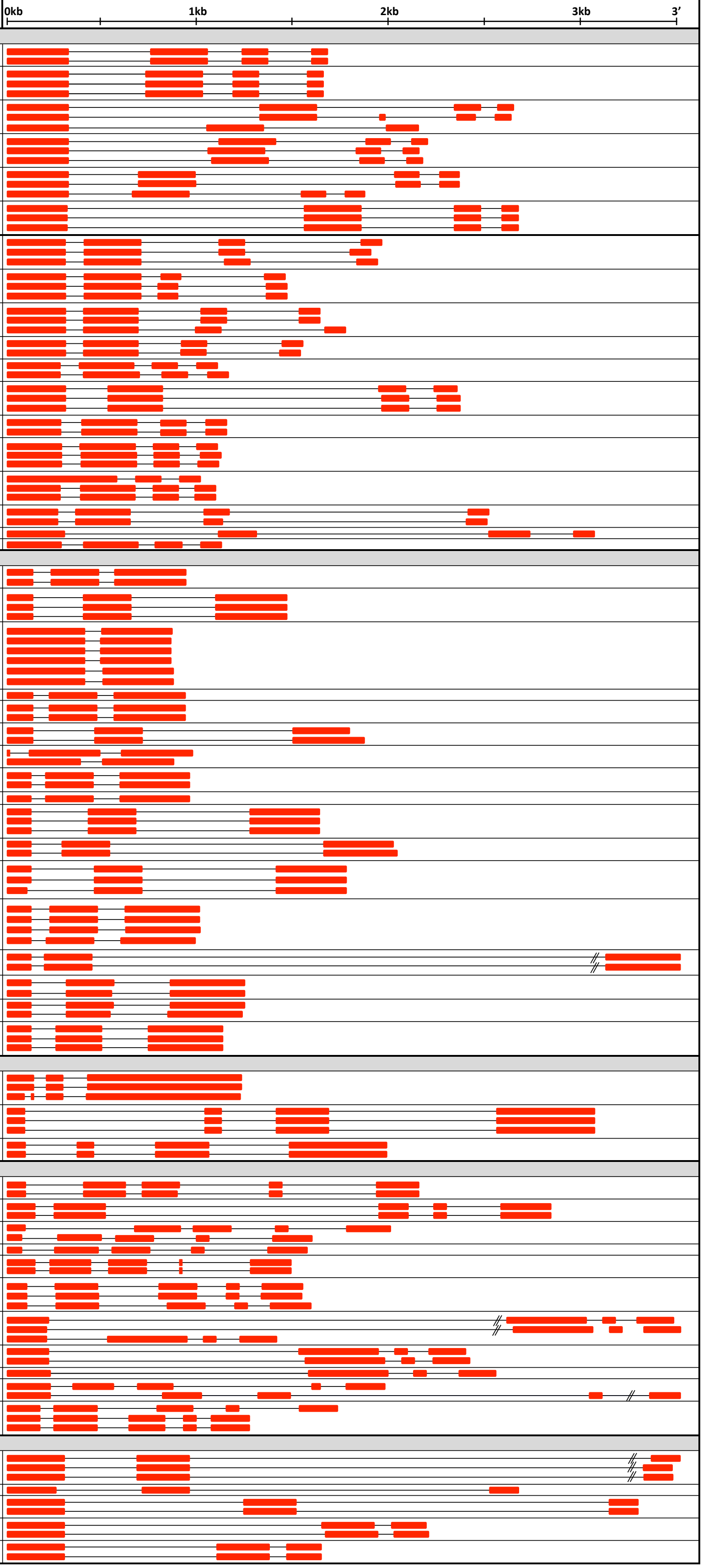

* Residue differences are in red
** Bo, Boric acid ; Ur, Urea
*** Similarities calculated with *Oleur*AQPa paralogue
