# Supplementary material for: Genome Wild Analysis and Molecular Understanding of the Aquaporin Diversity in Olive Trees (Olea Europaea L.)
Source: Int J Mol Sci. 2020 Jun 11;21(11):4183. doi: 10.3390/ijms21114183 (PMC7312470; doi:10.3390/ijms21114183)
Supplement: Supplementary file 1 [file ijms-21-04183-s001.zip › ijms-821530_Supplementary_files/Figure S8_Faize_OeuAQP protein SDPs.pdf]

Figure S8. SDP analysis of *Oeu*AQPs from the wild Olive species *Olea europaea* var. *sylvestris* from alignments with putative amino acid sequences of AQPs transporting non-aqua substrates. Multiple alignments were performed using ClustalW. Shown are the sequences containing the nine SD positions which are highlighted in yellow and the representative sequences are marked in blue. The Genbank accession numbers: *At*PIP1;2 (Q06611), *At*PIP2;1 (P43286), *At*PIP2;4 (Q9FF53), *At*TIP1;1 (P25818), *At*TIP1;2 (Q41963), *At*TIP1;3 (NP;192056), *At*TIP2;1 (Q41951), *At*TIP2;3 (Q9FGL2), *At*TIP4;1 (O82316), *At*TIP5;1 (NP;190328), *At*NIP1;1 (CAA16760), *At*NIP1;2 (Q8LFP7), *At*NIP5;1 (NP;192776), *At*NIP6;1 (NP;178191), *Cm*NIP2;1 (BAK09176), *Cp*NIP1 (CAD67694), *Gm*NOD26 (P08995), *Gm*NIP2;1 (XP;003534451), *Gm*NIP2;2 (NP;001240190), *Hv*PIP1;3 (BAA23745), *Hv*PIP1;4 (BAF33068), *Hv*PIP2;1 (BAA23744), *Hv*NIP2;1 (BAH24163), *Nt*AQP1 (O24662), *Nt*TIPa (Q9XG70), *Nt*XIP1;1α (ADO66667), *Nt*XIP1;1β (ADO66666), *Os*NIP2;1 (Q6Z2T3), *Os*NIP2;2 (Q67WJ8), *Os*NIP3;2 (Q7EYH7), *Sl*XIP1;1α (ADO66672), *Sl*XIP1;1β (ADO66671), *St*XIP1;1α (ADO66669), *St*XIP1;1β (ADO66670), *Ta*Lsi1 (ADM47602), *Ta*TIP2;1 (AAS19468), *Ta*TIP2;2 (AAS19469), *Tg*TIP1;1 (BAL41683), *Tg*TIP1;2 (BAL41684), *Zm*PIP1;1 (Q41870), *Zm*PIP1;5 (Q9AR14), *Zm*NIP2;1 (Q19KC1), *Zm*NIP2;2 (Q9ATN2).

Boric Acid Transporters

```
OeuPIP1;1a      KTVGIQQGIAWAFGGMIFALVYCTAGISGGHINPAVTFGLFLGRKLSLTRAVFYMVMQCLG
OeuPIP1;1b      KTVGIQQGIAWAFGGMIFALVYCTAGISGGHINPAVTFGLFLGRKLSLTRAVFYMVMQCLG
OeuPIP1;1c      KTVGIQQGIAWAFGGMIFALVYCTAGISGGHINPAVTFGLFLGRKLSLTRAVFYMVMQCLG
OeuPIP1;2       STVGIQQGIAWAFGGMIFALVYCTAGISGGHINPAVTFGLFLARKLSLTRAIFYMVMQCLG
OeuPIP1;3       ASVGVQQGIAWAFGGMIFALVYCTAGISGGHINPAVTFGLFLARKLSLIRALFYMVMQCLG
OeuPIP1;4       ASVGIQQGIAWAFGGMIFALVYCTAGISGGHINPAVTFGLFLARKLSLTRAIFYIVMQCLG
OeuPIP1;5       ASVGIQQGIAWSFGGMIFALVYCTAGISGGHINPAVTFGLFLARKLSLTRAIFYIVMQCLG
OeuPIP1;6       TSVGIQQGIAWAFGGMIFALVYCTAGISGGHINPAVTFGLFVARKLSLTRAIFYIVMQCLG
OeuNIP5;1       QAETLIGNAACAGLAAMIVILSTGHISGAHLNPSLTIAFAAVRHFPWAHVPGYIVAQISA
OeuNIP5;2       QAETLIGNAACAGLAVMIVILSTGHISGAHLNPSLTIAFAALRHFPWAHVPAYIAAQVSA
OeuNIP6;1       GSETLLGLAVSTGLAVMIVILSTGHISGAHVNPSVTIAFAALKHFPWKHVPVYIGAQILA
OeuXIP1;1       PNLIIS---ILIAIVVAILLLAVHPVSGGHINPVISFSAGLVGLISMSRAIVYILAQCIG
OeuXIP1;2       PNLVLS---ILAAIIIAILLLAVHPVSGGHINPVISFSAALVGLISMSKAFIYIVAQCLG
OeuXIP1;3       PNLVLS---ILAAIIIAIVLLAVHPISGGHINPVISFSAALVGLISVSKALIYISAQCLG
HvPIP1;3        GTVGIQQGIAWSFGGMIFVLVYCTAGISGGHINPAVTFGLFLARKLSLTRAVFYIVMQCLG
HvPIP1;4        GTVGIQQGIAWSFGGMIFVLVYCTAGISGGHINPAVTFGLFLARKLSLTRAVFYIVMQCLG
ZmPIP1;1        ATVGIQQGIAWSFGGMILALVYCTAGISG-HINPAVTFGLFLARKLSLTRAVFYIIMQCLG
NtXIP1;1α       PNLIMS---ILIAIVITILLLAVVPVSGGHINPVISFSAALVGIISMSRAIIYMVAQCVG
NtXIP1;1β       PNLIMS---ILIAIVITILLLAVVPVSGGHINPVISFSAALVGIISMSRAIIYMVAQCVG
StXIP1;1α       PNLIMS---ILIAVVITILLLAVVPVSGGHINPVISFSAALVGIISMSRAIIYIVAQCLG
StXIP1;1β       PNLIMS---ILIAVVITILLLAVVPVSGGHINPVISFSAALVGIISMSRAIIYIVAQCLG
AtNIP5;1        GAETLIGNAACAGLAVMIIILSTGHISGAHLNPSLTIAFAALRHFPWAHVPAYIAAQVSA
AtNIP6;1        GAETLIGCAASAGLAVMIVILSTGHISGAHLNPAVTIAFAALKHFPWKHVPVYIGAQVMA
OsNIP2;1        SRISQLGQSIAGGLIVTVMIYAVGHISGAHMNPAVTLAFAVFRHFPWIQVPFYWAAQFTG
                                      .     :: ..  :** *:** :::.      :.  :.   *    *   .


OeuPIP1;1a      AICGAGVVKGFGKTLYMT------------KGGGANVVAHGYTKGDGLGAEIIGTFVLVY
OeuPIP1;1b      AICGAGVVKGFGKTLYMT------------KGGGANVVAHGYTKGDGLGAEIIGTFVLVY
OeuPIP1;1c      AICGAGVVKGFGKTLYMT------------KGGGANVVAHGYTKGDGLGAEIIGTFVLVY
OeuPIP1;2       AICGAGVVKGFGKTLYMS------------KGGGANVVAHGYTKGSGLGAEIIGTFVLVY
OeuPIP1;3       AICGAGVVKGFMKGQYER------------LQGGANIVQHGYTKGDALGAEIVGTFILVY
OeuPIP1;4       AICGAGVVKGFQPSFYKA------------NGGGANVVSHGYTKGDGLGAEIIGTFVLVY
OeuPIP1;5       AICGAGVVKGFMEGPYQR------------LQGGANVVQHGYTKGDGLGAEIVGTFILVY
OeuPIP1;6       AICGAGVVKGFGPSFYKA------------NGGGANVVAHGYTKGDGLGAEIIGTFVLVY
OeuNIP5;1       SICASFALKGVFHPFMSG-----------------GVTVPSVSIGQAFALEFLITFNLLF
OeuNIP5;2       SICASFALKGVFHPFMSG-----------------GVTVPSVSTGQAFALEFLITFNLLF
OeuNIP6;1       STCAAFLLKAIFHPIMGG-----------------GVTVPSGGFAQAFALEFVISFNLMF
OeuXIP1;1       AILGALALKAVVSSTIEQNFSLGGCTLTVIAPGPNGPITLGLETAQAFWLEIFCTFVFLF
```

```
OeuXIP1;2        AVLGALALKAVVSSTTEETFSLGGCTLTVIAPGLNGPATVGLETVQAFWLEIFCTFVFLF
OeuXIP1;3        AALGALALKAVVSSTIEQTFSLGGCTLTVIAPGPNGLVTIGLETAQAFWLEIICTFVLLF
HvPIP1;3         AICGAGVVKGFQTTLYQG------------NGGGANSVAAGYTKGDGLGAEIVGTFVLVY
HvPIP1;4         AICGAGVVKGFQTTLYQG------------NGGGANSVAAGYTKGDGLGAEIVGTFVLVY
ZmPIP1;1         AICGRGVVKGFQQGLYMG------------NGGRRNVVAPGYTKGDGLGAEIVGTFILVY
NtXIP1;1α        AILGALALKAVVSSTIAQTFSLGGCTITVIAPGPNGPITVGLEMAQALWLEIFCTFVFLF
NtXIP1;1β        AILGALALKAVVSSTIAQTFSLGGCTITVIAPGPNGPITVGLEMAQALWLEIFCTFVFLF
StXIP1;1α        AVLGALALRAVVSSSIEDTFSLGGCTVTIIAPGPNGPVTVGLETAQALWLEIFCTFVFLF
StXIP1;1β        AVLGALALRAVVSSSIEDTFSLGGCTVTIIAPGPNGPVTVGLETAQALWLEIFCTFVFLF
AtNIP5;1         SICASFALKGVFHPFMSG-----------------GVTIPSVSLGQAFALEFIITFILLF
AtNIP6;1         SVSAAFALKAVFEPTMSG-----------------GVTVPTVGLSQAFALEFIISFNLMF
OsNIP2;1         AICASFVLKAVIHPVDVI-----------------GTTTPVGPHWHSLVVEVIVTFNMMF
                 :  .   ::..                        .          .:  *.. :* :::

OeuPIP1;1a       TVFSATDAKRSARDS---HVPILAPLPIGFAVFLVHLATIP--ITGTGINPARSLGAAII
OeuPIP1;1b       TVFSATDAKRSARDS---HVPILAPLPIGFAVFLVHLATIP--ITGTGINPARSLGAAII
OeuPIP1;1c       TVFSATDAKRSARDS---HVPILAPLPIGFAVFLVHLATIP--ITGTGINPARSLGAAII
OeuPIP1;2        TVFSATDAKRSARDS---HVPILAPLPIGFAVFLVHLATIP--ITGTGINPARSLGAAII
OeuPIP1;3        TVFSATDAKRNARDS---HVPLLAPLPIGFAVFLVHLATIP--ITGTGINPARSLGAAII
OeuPIP1;4        TVFSATDAKRNARDS---HVPILAPLPIGFAVFLVHLATIP--ITGTGINPARSLGAAII
OeuPIP1;5        TVFSATDAKRNARDS---HVPLLAPLPIGFAVFLVHLATIP--ITGTSINPARSLGAAII
OeuPIP1;6        TVFSATDAKRNARDS---HVPILAPLPIGFAVFLVHLATIP--ITGTGINPARSLGAAII
OeuNIP5;1        VVTAVATDTR--------AVGELAGIAVGATVMLNILVAGP--SSGGSMNPVRTLGPAVA
OeuNIP5;2        VVTAVATDTR--------AVGELAGIAVGATVMLNILVAGP--SSGGSMNPVRTLGPAVA
OeuNIP6;1        VVTAVATDTR--------AVGKLAGIGVGATVMLNILIAGE--TTGASMNPVRTLGPAIA
OeuXIP1;1        ASIWMAYDHRQAKNLGLVLVFSIIGLVLGLLVFISTTVTAKKGYAGAGMNPARCLGAAIV
OeuXIP1;2        ASIWMAYDPRQAKELGHVLIFSIVGVVLGLLVFISTTVTAKKGYAGAGMNPARCFGPAIV
OeuXIP1;3        ASVWMAYDHRQAKALGRVLVFSIVGVVLGLLVFISTTVTAKKGYAGAGMNPARCFGAAVV
HvPIP1;3         TVFSATDAKRSARDS---HVPILAPLPIGFAVFLVHLATIP--ITGTGINPARSLGAAII
HvPIP1;4         TVFSATDAKRSARDS---HVPILAPLPIGFAVFLVHLATIP--ITGTGINPARSLGAAII
ZmPIP1;1         TVFSATDAKRRARDS---HVPILAPLPIGFAVFLVHLATMG--ITGTGINPARSLGAAVI
NtXIP1;1α        ASIWMAYDHRQAKALGLVTVLSIVGIVLGLLVFISTTVTMKKGYAGAGMNPARCFGAAVV
NtXIP1;1β        ASIWMAYDHRQAKALGLVTVLSIVGIVLGLLVFISTTVTMKKGYAGAGMNPARCFGAAVV
StXIP1;1α        ASIWMAYDHRQAKALGHVTVLSIVGLVLGLLVFISTTVTAKKGYGGAGINPARCLGPAII
StXIP1;1β        ASIWMAYDHRQAKALGHVTVLSIVGLVLGLLVFISTTVTAKKGYGGAGINPARCLGPAII
AtNIP5;1         VVTAVATDTR--------AVGELAGIAVGATVMLNILVAGP--STGGSMNPVRTLGPAVA
AtNIP6;1         VVTAVATDTR--------AVGELAGIAVGATVMLNILIAGP--ATSASMNPVRTLGPAIA
OsNIP2;1         VTLAVATDTR--------AVGELAGLAVGSAVCITSIFAGA--ISGGSMNPARTLGPALA
                 .    :   *         :  :  : :*  * :    :      . .:**.* :*.*:
```

Carbon dioxide (CO_2) Transporters

```
OeuPIP1;4      HINPAVTFGLFLARKLSLTRAIFYIVMQCLGAICGAGVVKGFQPSFYKANGGGANVVSHG
OeuPIP1;6      HINPAVTFGLFVARKLSLTRAIFYIVMQCLGAICGAGVVKGFGPSFYKANGGGANVVAHG
AtPIP1;2       HINPAVTFGLFLARKLSLTRAVYYIVMQCLGAICGAGVVKGFQPKQYQALGGGANTIAHG
NtAQP1         HINPAVTFGLFLARKLSLTRAIFYIVMQCLGAICGAGVVKGFMVGPYQRLGGGANVVNHG
HvPIP2;1       HINPAVTFGLFLARKVSLIRALLYIIAQCLGAICGVGLVKGFQSSYYVRYGGGANELSAG
               ***********:***:** **: **: ********.*:****    *    ***** :  *

OeuPIP1;4      YTKGDGLGAEIIGTFVLVYTVFSATDAKRNARDSHVPILAPLPIGFAVFLVHLATIPITG
OeuPIP1;6      YTKGDGLGAEIIGTFVLVYTVFSATDAKRNARDSHVPILAPLPIGFAVFLVHLATIPITG
AtPIP1;2       YTKGSGLGAEIIGTFVLVYTVFSATDAKRNARDSHVPILAPLPIGFAVFLVHLATIPITG
NtAQP1         YTKGDGLGAEIIGTFVLVYTVFSATDAKRNARDSYVPILAPLPIGFAVFLVHLATIPITG
HvPIP2;1       YSKGTGLAAEIIGTFVLVYTVFSATDPKRNARDSHIPVLAPLPIGFAVFMVHLATIPITG
               *:** **.******************.*******::*:***********:**********

OeuPIP1;4      TGINPARSLGAAIIYNKDHAWDDHWIFWVGPFIGAALAALYHQVIIRAIPFKSSS----
OeuPIP1;6      TGINPARSLGAAIIYNKDHAWDDHWIFWVGPFIGAALAALYHQVIIRAIPFKSGN----
AtPIP1;2       TGINPARSLGAAIIFNKDNAWDDHWVFWVGPFIGAALAALYHVIVIRAIPFKSRS----
NtAQP1         TGINPARSLGAAIIYNTDQAWDDHWIFWVGPFIGAALAAVYHQIIIRAIPFHKSS----
HvPIP2;1       TGINPARSLGAAVIYNTDKAWDDQWIFWVGPLIGAAIAAAYHQYVLRASAAKLGSYRSN
               ************:*:*.*:****:*:*****:****:** **  ::** . :  .
```

H_2O_2 Transporters

```
OeuPIP1;1a      YMVMQCLGAICGAGVVKGFGKTLYMT------------KGGGANVVAHGYTKGDGLGAEI
OeuPIP1;1b      YMVMQCLGAICGAGVVKGFGKTLYMT------------KGGGANVVAHGYTKGDGLGAEI
OeuPIP1;1c      YMVMQCLGAICGAGVVKGFGKTLYMT------------KGGGANVVAHGYTKGDGLGAEI
OeuPIP1;2       YMVMQCLGAICGAGVVKGFGKTLYMS------------KGGGANVVAHGYTKGSGLGAEI
OeuPIP1;3       YMVMQCLGAICGAGVVKGFMKGQYER------------LQGGANIVQHGYTKGDALGAEI
OeuPIP1;4       YIVMQCLGAICGAGVVKGFQPSFYKA------------NGGGANVVSHGYTKGDGLGAEI
OeuPIP1;5       YIVMQCLGAICGAGVVKGFMEGPYQR------------LQGGANVVQHGYTKGDGLGAEI
OeuPIP1;6       YIVMQCLGAICGAGVVKGFGPSFYKA------------NGGGANVVAHGYTKGDGLGAEI
OeuPIP2;1       YMVAQCLGAICGVGLVKAFQKSYYHK------------YGGGANELAPGYNKGVGLGAEI
OeuPIP2;2       YMVAQCLGAICGVGLVKAFQKSYYNL------------YGGGANELAPGYNKGVGLGAEI
OeuPIP2;3       YMVAQCLGAICGVGLVKAFQKAYYKK------------YGGGANELADGYSTGVGLSAEI
OeuPIP2;4       YMVAQCLGAICGVALVKALQKSHYKK------------YGGGANELADGYSTGVGLSAEI
OeuPIP2;5       YMVAQCLGAICGCGLVKAFQKSYYVK------------FGGGANELADGYSNGTGLAAEI
OeuPIP2;6       YMVAQCLGAICGVGLVKAFQKSYYKK------------YGGGANVLADGISTGVGLAAEI
OeuPIP2;7       YMVAQCVGAICGCGLVKAFQRAYYVR------------YGGGANELGDGYSTGTGLAAEI
OeuPIP2;8       YMIAQCLGAVCGVGLVKAFMKSYYNR------------LGGGANVVSHGYNIGTALGAEI
OeuPIP2;9       YMIAQCLGAICGVGLVKAFMKGYYNR------------LGGGANSVSHGYNTGTALGAEI
OeuPIP2;10      YMVAQCLGAICGVALVKAVQKSHYKK------------YGGGVNELSDGYSTGGGLAAEI
OeuTIP1;7       YWIAQLLGSVVACLLLKFATGGLTTA-----------------AFSLSAGVNVWNAFVLEI
OeuTIP2;1       YWIAQLLGSIVACYLLVFVTGGLAIP-----------------IHGVGAGVGAFEGVVMEI
OeuTIP2;2       YWIAQLLGSIVACYLLVFVTGGLAIP-----------------IHGVGAGVGAFEGVVMEI
OeuTIP5;3       YSISQTFGSVMACLLLKATTAGQHVP-----------------THAIEHEMTGFGGAVLEG
OeuNIP1;1       YISAQMIGATLAGGTLRLLF-------------------QGKHDHFGGTFPTGSDMQSFV
OeuNIP1;3       YIAAQVIGATLASGTLRLLF-------------------MGKHDHFAGTLPTGSDLQSFV
OeuXIP1;1       YILAQCIGAILGALALKAVVSSTIEQNFSLGGCTLTVIAPGPNGPITLGLETAQAFWLEI
OeuXIP1;3       YISAQCLGAALGALALKAVVSSTIEQTFSLGGCTLTVIAPGQNGPVTIGLETAQAFWLEI
AtTIP1;1        YWIAQLLGSVVACLILKFATGGLAVP-----------------AFGLSAGVGVLNAFVFEI
AtTIP1;2        YWIAQLLGSVAACFLLSFATGGEPIP-----------------AFGLSAGVGSLNALVFEI
TgTIP1;1        YIIAQLLGSSVACLLLRFTTG-LGTG-----------------TFGLVAGVSVWSGLVMEI
TgTIP1;2        YIIAQLLGSSVACLLLRFTTG-LGTG-----------------TFGLVAGVSVWSGLVMEI
AtTIP2;3        YWIAQCLGSIVACLLLVFVTNGKSVP-----------------THGVSAGLGAVEGVVMEI
AtPIP2;1        YIIAQCLGAICGVGFVKAFQSSYYTR------------YGGGANSLADGYSTGTGLAAEI
AtPIP2;2        YMVAQCLGAICGVGFVKAFQSSYYDR------------YGGGANSLADGYNTGTGLAAEI
AtPIP2;4        YIVAQCLGAICGCGFVKAFQSSYYTR------------YGGGANELADGYNKGTGLGAEI
ZmPIP2;5        YIVAQCLGAICGVGLVKGFQSAFYVR------------YGGGANELSAGYSKGTGLAAEI
AtPIP2;5        YMVAQCLGAICGVALVKAFQSAYFTR------------YGGGANGLSDGYSIGTGVAAEI
AtPIP2;7        YMIAQCLGAICGVGFVKAFMKTPYNT------------LGGGANTVADGYSKGTALGAEI
NtXIP1;1α       YMVAQCVGAILGALALKAVVSSTIAQTFSLGGCTITVIAPGPNGPITVGLEMAQALWLEI
NtXIP1;1β       YMVAQCVGAILGALALKAVVSSTIAQTFSLGGCTITVIAPGPNGPITVGLEMAQALWLEI
StXIP1;1α       YIVAQCLGAVLGALALRAVVSSSIEDTFSLGGCTVTIIAPGPNGPVTVGLETAQALWLEI
StXIP1;1β       YIVAQCLGAVLGALALRAVVSSSIEDTFSLGGCTVTIIAPGPNGPVTVGLETAQALWLEI
SlXIP1;1α       YIVAQCVGAILGALALRAVVSSSIEDTFSLGGCTVTIIAPGPNGPVIVGLETAQALWLEI
SlXIP1;1β       YIVAQCVGAILGALALRAVVSSSIEDTFSLGGCTVTIIAPGPNGPVIVGLETAQALWLEI
AtNIP1;2        YVISQVIGSTLAAATLRLLFGLDQDV------------CSGKHDVFVGTLPSGSNLQSFV
                *   * .*:  .   :                             .
```

```
OeuPIP1;1a      IGTFVLVYTVFSATDAKRSARDS---HVPILAPLPIGFAVFLVHLATIP--ITGTGINPA
OeuPIP1;1b      IGTFVLVYTVFSATDAKRSARDS---HVPILAPLPIGFAVFLVHLATIP--ITGTGINPA
OeuPIP1;1c      IGTFVLVYTVFSATDAKRSARDS---HVPILAPLPIGFAVFLVHLATIP--ITGTGINPA
OeuPIP1;2       IGTFVLVYTVFSATDAKRSARDS---HVPILAPLPIGFAVFLVHLATIP--ITGTGINPA
OeuPIP1;3       VGTFILVYTVFSATDAKRNARDS---HVPLLAPLPIGFAVFLVHLATIP--ITGTGINPA
OeuPIP1;4       IGTFVLVYTVFSATDAKRNARDS---HVPILAPLPIGFAVFLVHLATIP--ITGTGINPA
OeuPIP1;5       VGTFILVYTVFSATDAKRNARDS---HVPLLAPLPIGFAVFLVHLATIP--ITGTSINPA
OeuPIP1;6       IGTFVLVYTVFSATDAKRNARDS---HVPILAPLPIGFAVFLVHLATIP--ITGTGINPA
OeuPIP2;1       IGTFVLVYTVFSATDPKRNARDS---HVPVLAPLPIGFAVFMVHLATIP--ITGTGINPA
OeuPIP2;2       IGTFVLVYTVFSATDPKRNARDS---HVPVLAPLPIGFAVFMVHLATIP--ITGTGINPA
OeuPIP2;3       IGTFVLVYTVFSATDPKRSARDS---HVPVLAPLPIGFAVFMVHLATIP--ITGTGINPA
OeuPIP2;4       IGTFVLVYTVFSATDPKRSARDS---HVPVLAPLPIGFAVFMVHLATIP--ITGTGINPA
OeuPIP2;5       IGTFVLVYTVFSATDPKRNARDS---HVPVLAPLPIGFAVFMVHLATIP--ITGTGINPA
OeuPIP2;6       IGTFVLVYTVFSATDPKRSARDS---HVPVLAPLPIGFAVFMVHLATIP--VTGTGINPA
OeuPIP2;7       IGTFVLVYTVFSATDPKRSARDS---HVPVLAPLPIGFAVFMVHLATIP--ITGTGINPA
OeuPIP2;8       IGTFVLVYTVFSATDPKRSARDS---HVPVLAPLPIGFAVFMVHLATIP--ITGTGINPA
OeuPIP2;9       IGTFVLVYTVFSATDPKRSARDS---HVPVLAPLPIGFAVFVVHLATIP--ITGTGINPA
OeuPIP2;10      IGTFVLVYTVFSAIYAKRKARDS---HVPVLAPLSIGFAVFMVHLATIP--ITGTSINPA
OeuTIP1;7       VMTFGLVYTVYATAVDPKRGSLG------TIAPIAIGFIVGANILAGGA--FDGASMNPA
OeuTIP2;1       VITFALVYTVYATAADPKKGSLG------TIAPIAIGFIVGANILAAGP--FSGGSMNPA
OeuTIP2;2       VITFALVYTVYATAADPKKGSLG------TIAPIAIGFIVGANILAAGP--FSGGSMNPA
OeuTIP5;3       VMTFALVYTVYVASN-LRPVSAG------AIGPLAIGLIAGANVLASGP--FTGGSMNPA
OeuNIP1;1       VEFIITFYLMFVISGVATDNRAIG-----ELAGLAIGATILLNVMIAGP--VSGASMNPA
OeuNIP1;3       MEFIITFYLMFVISGVATDNRAIG-----ELSGLAIGATILLNVMFAGP--VSGASMNPV
OeuXIP1;1       FCTFVFLFASIWMAYDHRQAKNLGLVLVFSIIGLVLGLLVFISTTVTAKKGYAGAGMNPA
OeuXIP1;3       ICTFVLLFASVWMAYDHRQAKALGRVLVFSIVGVVLGLLVFISTTVTAKKGYAGAGMNPA
AtTIP1;1        VMTFGLVYTVYATAIDPKNGSLG------TIAPIAIGFIVGANILAGGA--FSGASMNPA
AtTIP1;2        VMTFGLVYTVYATAVDPKNGSLG------TIAPIAIGFIVGANILAGGA--FSGASMNPA
TgTIP1;1        VMTFGLVYTVYATAVDPKKGDIG------TIAPIAIGFIVGANILVGGA--FTGASMNPA
TgTIP1;2        VMTFGLVYTVYATAVDPKKGDIG------TIAPIAIGFIVGANILVGGA--FTGASMNPA
AtTIP2;3        VVTFALVYTVYATAADPKKGSLG------TIAPIAIGFIVGANILAAGP--FSGGSMNPA
AtPIP2;1        IGTFVLVYTVFSATDPKRSARDS---HVPVLAPLPIGFAVFMVHLATIP--ITGTGINPA
AtPIP2;2        IGTFVLVYTVFSATDPKRNARDS---HVPVLAPLPIGFAVFMVHLATIP--ITGTGINPA
AtPIP2;4        IGTFVLVYTVFSATDPKRNARDS---HVPVLAPLPIGFAVFMVHLATIP--ITGTGINPA
ZmPIP2;5        IGTFVLVYTVFSATDPKRNARDS---HVPVLAPLPIGFAVFMVHLATIP--ITGTGINPA
AtPIP2;5        IGTFVLVYTVFSATDPKRSARDS---HVPVLAPLPIGFAVFIVHLATIP--ITGTGINPA
AtPIP2;7        IGTFVLVYTVFSATDPKRSARDS---HIPVLAPLPIGFAVFMVHLATIP--ITGTGINPA
NtXIP1;1α       FCTFVFLFASIWMAYDHRQAKALGLVTVLSIVGIVLGLLVFISTTVTMKKGYAGAGMNPA
NtXIP1;1β       FCTFVFLFASIWMAYDHRQAKALGLVTVLSIVGIVLGLLVFISTTVTMKKGYAGAGMNPA
StXIP1;1α       FCTFVFLFASIWMAYDHRQAKALGHVTVLSIVGLVLGLLVFISTTVTAKKGYGGAGINPA
StXIP1;1β       FCTFVFLFASIWMAYDHRQAKALGHVTVLSIVGLVLGLLVFISTTVTAKKGYGGAGINPA
SlXIP1;1α       FCTFVFLFASIWMAYDHRQAKALGHVTVLSIVGLVLGLLVFISTTVTAKKGYGGAGINPA
SlXIP1;1β       FCTFVFLFASIWMAYDHRQAKALGHVTVLSIVGLVLGLLVFISTTVTAKKGYGGAGINPA
AtNIP1;2        IEFIITFYLMFVISGVATDNRAIG-----ELAGLAVGSTVLLNVIIAGP--VSGASMNPG
                .  :  .:                      :  : :*                * .:**
```

```
OeuPIP1;1a      RSLGAAIIYNKDKAWDDHWIFWVGPFIGAALAALYHVVVIRAIPFKK-------------
OeuPIP1;1b      RSLGAAIIYNKDKAWDDHWIFWVGPFIGAALAALYHVVVIRAIPFKK-------------
OeuPIP1;1c      RSLGAAIIYNKDKAWDDHWIFWVGPFIGAALAALYHVVVIRAIPFKK-------------
OeuPIP1;2       RSLGAAIIYNKDKAWDDHWIFWVGPFIGAALAALYHVVVIRAIPFKNK------------
OeuPIP1;3       RSLGAAIIYNRDHAWDDQWIFWVGPFIGAALAAIYHQIVIRAIPFKSRA-----------
OeuPIP1;4       RSLGAAIIYNKDHAWDDHWIFWVGPFIGAALAALYHQVIIRAIPFKSSS-----------
OeuPIP1;5       RSLGAAIIYNRDNAWDDHWIFWVGPFIGAALAAIYHQIVIRAIPFKSSQAC---------
OeuPIP1;6       RSLGAAIIYNKDHAWDDHWIFWVGPFIGAALAALYHQVIIRAIPFKSGN-----------
OeuPIP2;1      RSFGAAVIYNEDKPWDDHWIFWVGPFVGALIAAIYHQYILRAGAIKALGSFRSNA-----
OeuPIP2;2      RSFGAAVIYNKDKPWDDHWIFWVGPFIGAAIAAFYHQYILRAAAIKALGSFRSNN-----
OeuPIP2;3      RSFGAAVIYNQEKAWDDQWIFWVGPFIGAAIAAFYHQFILRAGAAKALGSFRSSYHV---
OeuPIP2;4      RSLGAAVIYNQEKAWDDQWIFWVGPFFGAAIAAFYHQFILRAGAAKALGSFRSSSHV---
OeuPIP2;5      RSLGAAVIYGKDKAWDDQWIFWVGPFIGAAIAAFYYQYILRAGAVKALGSFRSTAH----
OeuPIP2;6      RSLGAAVIYDQHKAWDDHWIFWVGPFIGAAVAAFYHQFILRAGAAKALGSFRSSASHH--
OeuPIP2;7      RSFGAAVIYGKDKAWDDQWIFWAGPFIGAAIAALYHQYILRAGAVKALGSFRSSASAY--
OeuPIP2;8      RSFGAAVIYNNDKVWDDHWIFWVGPFVGALAAAAYHQYILRAAAIKAIGSFRSNPTN---
OeuPIP2;9      RSFGAAVIFNNDKVWDDHWIFWVGPFVGALAAAAYHQYILRAAAIKALGSFRSNPTN---
OeuPIP2;10     RSFGPAVIYNQKKAWDDQWIFWVGPFIGAAMAALYHQCILRA---KAFRSYRSSSRI---
OeuTIP1;7      VSFGPAVVSW---TWDNHWVYWAGPLIGGGLAGLIYEFFFINQ-THEQLPTTDH------
OeuTIP2;1      RSFGPAVASG---NFSGIWIYWVGPLIGGGLAGLVYAYVFMSH--EHAPLPTDF------
OeuTIP2;2      RSFGPAVASG---NFSGIWIYWVGPLIGGGLAGLVYAYVFMSH--EHAPLPTDF------
OeuTIP5;3      YAFGSAIVGG---SFKNQAVYWVGPLFGAAIAGILHDNVVCPAPVPDSFRGNADGVGV--
OeuNIP1;1      RSLGPAIVSN---KYKGIWIYIVGPTLGAVAGAWVYNIIRFTNKPLREITKSASFLKSSA
OeuNIP1;3      RSLGPAIVSN---RYEGIWIYIVGPTLGAIAGAWAYNIIRFTDKPLREITQTASFLKILA
OeuXIP1;1      RCLGAAIVRG-GHLWDGHWVFWAGPAIACVAFYLYTKIIPRQHHHAK-GYGHDFFDTVKA
OeuXIP1;3      RCFGAAVVRG-GHLWNGHWIFWVGPAIACVAFYLYTKIIPRQHYQAKF-YDHDFFNVVKA
AtTIP1;1       VAFGPAVVSW---TWTNHWVYWAGPLVGGGIAGLIYEVFFINT-THEQLPTTDY------
AtTIP1;2       VAFGPAVVSW---TWTNHWVYWAGPLIGGGLAGIIYDFVFIDENAHEQLPTTDY------
TgTIP1;1       IAFGPALVSW---SWTSHWVYWVGPLIGGGIAGIVYDLFFINS-THEQLPSTDY------
TgTIP1;2       IAFGPALVSW---SWASHWVYWVGPLIGGGIAAIVYDLFFISG-THEQLPTTDY------
AtTIP2;3       RSFGPAVVSG---DLSQIWIYWVGPLVGGALAGLIYGDVFIGS--YEAVETREIRV----
AtPIP2;1       RSFGAAVIYNKSKPWDDHWIFWVGPFIGAAIAAFYHQFVLRASGSKSLGSFRSAANV---
AtPIP2;2       RSFGAAVIYNKSKPWDDHWIFWVGPFIGAAIAAFYHQFVLRASGSKSLGSFRSAANV---
AtPIP2;4       RSFGAAVIYNNEKAWDDQWIFWVGPMIGAAAAAFYHQFILRAAAIKALGSFGSFGSFRSF
ZmPIP2;5       RSLGAAVIYNNDKAWDDHWIFWVGPFIGAAIAAAYHQYVLRASAAK-LGSSASFSR----
AtPIP2;5       RSLGAAIIYNKDKAWDHHWIFWVGPFAGAAIAAFYHQFVLRAGAIKALGSFRSQPHV---
AtPIP2;7       RSFGAAVIYNNEKAWDDQWIFWVGPFLGALAAAAYHQYILRASAIKALGSFRSNATN---
NtXIP1;1α      RCFGAAVVRG-GHLWDGHWIFWVGPTIACVAFYVYTKIIPPKHFHAD-GYKYDFIGVVKA
NtXIP1;1β      RCFGAAVVRG-GHLWDGHWIFWVGPTIACVAFYVYTKIIPPKHFHAD-GYKYDFIGVVKA
StXIP1;1α      RCLGPAIIRG-GHLWDGHWIFWVGPTIGCVAFYVYTKIIPPKHFLAEYGFKHDFVGVVKA
StXIP1;1β      RCLGPAIIRG-GHLWDGHWIFWVGPTIGCVAFYVYTKIIPPKHFLAEYGFKHDFVGVVKA
SlXIP1;1α      RCLGPAIIRG-GHLWDGHWIFWVGPTIGCVAFYVYTKIIPTKHFLAEYGFKHDFVGVVKA
SlXIP1;1β      RCLGPAIIRG-GHLWDGHWIFWVGPTIGCVAFYVYTKIIPTKHFLAEYGFKHDFVGVVKA
AtNIP1;2       RSLGPAMVYS---CYRGLWIYIVSPIVGAVSGAWVYNMVRYTDKPLREITKSGSFLKT-V
                .:*.*:            :: ..*  .          .
```

Urea Transporters

```
OeuPIP1;1a      GVSKSDS--------KCKTVGIQGIAWAFGGMIFALVYCTAGISGGHINPAVTFGLFLGR
OeuPIP1;1b      GVSKSDS--------KCKTVGIQGIAWAFGGMIFALVYCTAGISGGHINPAVTFGLFLGR
OeuPIP1;1c      GVSKSDS--------KCKTVGIQGIAWAFGGMIFALVYCTAGISGGHINPAVTFGLFLGR
OeuPIP1;2       GVSKSDS--------KCSTVGIQGIAWAFGGMIFALVYCTAGISGGHINPAVTFGLFLAR
OeuPIP1;3       GVFSAKN--------KCASVGVQGIAWAFGGMIFALVYCTAGISGGHINPAVTFGLFLAR
OeuPIP1;4       GVSRSGS--------KCASVGIQGIAWAFGGMIFALVYCTAGISGGHINPAVTFGLFLAR
OeuPIP1;5       GVGRSNS--------KCASVGIQGIAWSFGGMIFALVYCTAGISGGHINPAVTFGLFLAR
OeuPIP1;6       GVSRSNS--------KCTSVGIQGIAWAFGGMIFALVYCTAGISGGHINPAVTFGLFVAR
OeuPIP2;1       GYKSQIDPAHG--GDDCGGVGILGIAWAFGGMIFILVYCTAGISGGHINPAVTFGLFLGR
OeuPIP2;2       GYKSQIDPAHG--GDQCGGVGILGIAWAFGGMIFILVYCTAGISGGHINPAVTFGLFLGR
OeuPIP2;3       GYKSQTDTNAGHGGDQCGGVGVLGIAWAFGGMIFVLVYCTAGISGGHINPAVTFGLFLAR
OeuPIP2;4       GYKSQIDTTVGHGGDQCGGVGILGIAWAFGGMIFVLVYCTAGISGGHINPAVTFGLFLAR
OeuPIP2;5       GYKSESA------ADKCGGVGILGIAWAFGGMIFVLVYCTAGISGGHINPAVTLGLFLAR
OeuPIP2;6       GYKSQTDPDLK--GDQCDGVGILGIAWAFGGMIFILVYCTAGISGGHINPAVTFGLFLAR
OeuPIP2;7       GYKSQSA------TDECGGVGILGIAWAFGGMIFVLVYCTAGISGGHINPAVTFGLFLAR
OeuPIP2;8       GHKKLNA------ADQCDGVGILGIAWAFGGMIFILVYCTAGISGGHINPAVTFGLFLAR
OeuPIP2;9       GHKKLNA------ADQCDGVGILGIAWAFGGMIFILVYCTAGISGGHINPAVTFGLFLAR
OeuPIP2;10      GYKSQNDTHCG-----GGGVGILGIAWAFGGMIFVLVYCTAGISGGHINPAVTFGLFLAR
OeuPIP2;11      GYKSHLDTDLK--GDQCGGVRILGIAWAFGGMIFILVYCTAGISGGHINPAVTFGLFLAR
OeuTIP1;1       AFAKLTDNE------STTPAGLIAASIAHAFALFVAVSVGANISGGHVNPAVTLGAFVGG
OeuTIP1;2       AFAKLTDGG------SSTPAGLIAAAIAHAFALFVAVSVAANISGGHVNPAVTFGAFVGG
OeuTIP1;3       AYNKLTGNA------PTSPSGLVAAAVAHGFALFVAVAVSANISGGHVNPAVTFGLFLGG
OeuTIP1;4       AFAKLTNNG------STTPTGLIAEAIAHAFALFVAVSVGANISGGHVNPAVTFGAFVGG
OeuTIP1;5       AFAKLTNNG------STTPTGLIAEAIAHAFALFVAVSVGANISGGHVNPAVTFGAFVGG
OeuTIP1;6       AFNKLSNDG------SPNPSGLLAAALAHAFALFVAVSVGANISGGHVNPAVTFGAFIGG
OeuTIP1;7       AFNKLTDDG------ATTPAGLIAAAVAHAFALFVAVSVGANISGGHVNPAVTFGAFVGG
OeuTIP2;1       AYNKLTADA------ALDPAGLVAVAICHGFALFVAVSIGANISGGHVNPAVTFGLAVGG
OeuTIP2;2       AYNKLTADA------ALDPAGLVAVAICHGFALFVAVSIGANISGGHVNPAVTFGLAVGG
OeuTIP2;3       AYNKLTYDA------ALDPAGLVAVAVCHGFALFVAVSIGANISGGHVNPAVTFGLAVGG
OeuTIP2;4       AYNKLTSDA------ALDPPGLVAVAVAHAFALFVGVSMAANVSGGHLNPAVTFGLAIGG
OeuTIP2;5       AYNKLTSDA------ALDPPGLVAVAVAHAFALFVGVSMAANVSGGHLNPAVTFGLAIGG
OeuTIP3;1       ALDKMYKDT------ALGASSLVVIALAHALSLFAAVASSMNVSGGHVNPAVTFGALVGG
OeuTIP4;1       ATDKLNGD---------PLVGLFFVAMAHALVVAVMISAGFRVSGGHINPAVTLGLLAGG
OeuTIP5;1       STLKMMPDA------ASNSSGLVAIAVANAFALSVAVYIAYNISGGHVNPAVTFGMAIGG
OeuTIP5;2       STLKMMPDA------ASNSSGLVAIAVANAFALSVAVYIAYNISGGHVNPAVTFGMAIGG
OeuTIP5;3       SSRKMMPDA------ASDSSSLVAIAVANAFALSVTVYISANISGGHVNPAVTFGMVVGG
OeuNIP1;1       VNADKDKIIT-----------MPGIAIVWGLVVMVMIYSVGHISGAHFNPAVTIAFASCK
OeuNIP1;2       VNKNMENVVT-----------LPGIAAVCGLVVVVMIYSVGHISGAHLNPAVTIAFATFK
OeuNIP1;3       VNADKEEVIT-----------QPGIAIVWGLVVMAMVYSVGHISGAHFNPAVTIAFATCK
OeuNIP3;1       VDR--EKTLT-----------VVGLAMVWGLALMAMIYALGHVSGGHFNPAVTIGFAAAR
OeuNIP4;1       VNKLYGGTVT-----------FPGICATWGLIVMVMVYSVGHISGAHFNPAVTITFAIFR
OeuNIP5;1       VNQKYNQAET-----------LIGNAACAGLAAMIVILSTGHISGAHLNPSLTIAFAAVR
OeuNIP5;2       VNQKYNQAET-----------LIGNAACAGLAVMIVILSTGHISGAHLNPSLTIAFAALR
OeuNIP7;1       IMHLIGLEAG-----------LMEYAATAALTVTVIVFSIGAISGAHVNPAVTIAFATVG
OeuXIP1;2       STIESDI-----------KIPNLVLSILAAIIIAILLLAVHPVSGGHINPVISFSAALVG
OeuXIP1;3       STIETDV-----------KMPNLVLSILAAIIIAIVLLAVHPISGGHINPVISFSAALVG
NtTIPa          AANKLNGD---------PLVSLFFVAMAHALVVAVTISAGFRISGGHLNPAVTLGLCMGG
AtTIP4;1        ATDSLVGN---------TLVGLFAVAVAHAFVVAVMISAG-HISGGHLNPAVTLGLLLGG
AtTIP1;1        AFNKLTENG------ATTPSGLVAAAVAHAFGLFVAVSVGANISGGHVNPAVTFGAFIGG
AtTIP1;2        AFNKITDNG------ATTPSGLVAAALAHAFGLFVAVSVGANISGGHVNPAVTFGVLLGG
AtTIP1;3        AYGKLTGDG------PATPAGLVAASLSHAFALFVAVSVGANVSGGHVNPAVTFGAFIGG
AtTIP2;1        AYAKLTSDA------ALDTPGLVAIAVCHGFALFVAVAIGANISGGHVNPAVTFGLAVGG
AtTIP5;1        SSRKLMAGD------VSGPFGVLIPAIANALALSSSVYISWNVSGGHVNPAVTFAMAVAG
NtAQP1          GLKRSDS--------LCSSVGIQGVAWAFGGMIFALVYCTAGISGGHINPAVTFGLFLAR
ZmPIP1;5        GVSKSSS--------KCATVGIQGIAWSFGGMIFALVYCTAGISGGHINPAVTFGLFLAR
NtXIP1;1α       STFESDV-----------KMPNLIMSILIAIVITILLLAVVPVSGGHINPVISFSAALVG
NtXIP1;1β       STFESDV-----------KMPNLIMSILIAIVITILLLAVVPVSGGHINPVISFSAALVG
StXIP1;1α       STFESDT-----------KMPNLIMSILIAVVITILLLAVVPVSGGHINPVISFSAALVG
StXIP1;1β       STFESDT-----------KMPNLIMSILIAVVITILLLAVVPVSGGHINPVISFSAALVG
CpNIP1          LNGSDAQRVS-----------QLGASVAGGLIVTVMIYAVGHISGAHMNPAVTTAFAATR
OsNIP2;1        ISGSDLSRIS-----------QLGQSIAGGLIVTVMIYAVGHISGAHMNPAVTLAFAVFR
AtNIP6;1        VNQKTDGAET-----------LIGCAASAGLAVMIVILSTGHISGAHLNPAVTIAFAALK
                                         .   .      :     :**.*.** ::
```

```
OeuPIP1;1a      KGDGLGAEIIGTFVLVYT-VFSATDAKRSARDSHVPILAPLPIGFAVFLVHLATIPI-TG
OeuPIP1;1b      KGDGLGAEIIGTFVLVYT-VFSATDAKRSARDSHVPILAPLPIGFAVFLVHLATIPI-TG
OeuPIP1;1c      KGDGLGAEIIGTFVLVYT-VFSATDAKRSARDSHVPILAPLPIGFAVFLVHLATIPI-TG
OeuPIP1;2       KGSGLGAEIIGTFVLVYT-VFSATDAKRSARDSHVPILAPLPIGFAVFLVHLATIPI-TG
OeuPIP1;3       KGDALGAEIVGTFILVYT-VFSATDAKRNARDSHVPLLAPLPIGFAVFLVHLATIPI-TG
OeuPIP1;4       KGDGLGAEIIGTFVLVYT-VFSATDAKRNARDSHVPILAPLPIGFAVFLVHLATIPI-TG
OeuPIP1;5       KGDGLGAEIVGTFILVYT-VFSATDAKRNARDSHVPLLAPLPIGFAVFLVHLATIPI-TG
OeuPIP1;6       KGDGLGAEIIGTFVLVYT-VFSATDAKRNARDSHVPILAPLPIGFAVFLVHLATIPI-TG
OeuPIP2;1       KGVGLGAEIIGTFVLVYT-VFSATDPKRNARDSHVPVLAPLPIGFAVFMVHLATIPI-TG
OeuPIP2;2       KGVGLGAEIIGTFVLVYT-VFSATDPKRNARDSHVPVLAPLPIGFAVFMVHLATIPI-TG
OeuPIP2;3       TGVGLSAEIIGTFVLVYT-VFSATDPKRSARDSHVPVLAPLPIGFAVFMVHLATIPI-TG
OeuPIP2;4       TGVGLSAEIIGTFVLVYT-VFSATDPKRSARDSHVPVLAPLPIGFAVFMVHLATIPI-TG
OeuPIP2;5       NGTGLAAEIIGTFVLVYT-VFSATDPKRNARDSHVPVLAPLPIGFAVFMVHLATIPI-TG
OeuPIP2;6       TGVGLAAEIIGTFVLVYT-VFSATDPKRSARDSHVPVLAPLPIGFAVFMVHLATIPV-TG
OeuPIP2;7       TGTGLAAEIIGTFVLVYT-VFSATDPKRSARDSHVPVLAPLPIGFAVFMVHLATIPI-TG
OeuPIP2;8       IGTALGAEIIGTFVLVYT-VFSATDPKRSARDSHVPVLAPLPIGFAVFMVHLATIPI-TG
OeuPIP2;9       TGTALGAEIIGTFVLVYT-VFSATDPKRSARDSHVPVLAPLPIGFAVFVVHLATIPI-TG
OeuPIP2;10      TGGGLAAEIIGTFVLVYT-VFSAIYAKRKARDSHVPVLAPLSIGFAVFMVHLATIPI-TG
OeuPIP2;11      TSVGLS--IIGTFVLVYT-VFSATDPKRSARDSHV-VLAPLPIGFAVFMIHLATIPI-TG
OeuTIP1;1       VWNAVIFEIVMTFGLVYTVYATAVDPKK----GNLGIIAPIAIGFIVGANILAGGAF-DG
OeuTIP1;2       SWNALVFEIVMTFGLVYTVYATAVDPKR----GDIGIIAPIAIGFIVGANILAGGAF-DG
OeuTIP1;3       VWNAFVFEIVMTFGLVYTVYATAVDPKK----GEIGIIAPIAIGFIVGANILAGGAF-TG
OeuTIP1;4       VWNAVVFEIVMTFGLVYTVYATAVDPKR----GNLGIIAPIAIGFIVGANILAGGAF-DG
OeuTIP1;5       VWNAVVFEIVMTFGLVYTVYATAVDPKR----GNLGIIAPIAIGFIVGANILAGGAF-DG
OeuTIP1;6       VWNALVFEIVMTFGLVYTVYATAVDPKK----GNLGNIAPIAIGFIVGANILAGGPF-DG
OeuTIP1;7       VWNAFVLEIVMTFGLVYTVYATAVDPKR----GSLGTIAPIAIGFIVGANILAGGAF-DG
OeuTIP2;1       AFEGVVMEIVITFALVYTVYATAADPKK----GSLGTIAPIAIGFIVGANILAAGPF-SG
OeuTIP2;2       AFEGVVMEIVITFALVYTVYATAADPKK----GSLGTIAPIAIGFIVGANILAAGPF-SG
OeuTIP2;3       AIEGVVMEIVITFALVYTVYATAVDPKK----GSLGTIAPIAIGFIVGANILAAGPF-SG
OeuTIP2;4       AVEGVVMEIVITFALVYTVYATAADPKK----GSLGIIAPIAIGFIVGANILAAGPF-SG
OeuTIP2;5       AVEGVVMEIVITFALVYTVYATAADPKK----GSLGTIAPIAIGFIVGANILAAGPF-SG
OeuTIP3;1       EWNALVMEIVLTFGLVYTVYATAIDPKR----GSLGTIAPLAIAFIVGANVLVGGPF-SG
OeuTIP4;1       FAQGVIMEIVLTFSLLFTVYATIVDPKK----GFLEGMGPLLTGLVVGANVMAGGPF-SG
OeuTIP5;1       GFGAAILEGVMTFTLVYTVYA-AADPRR----GSMSIIGPLAIGLVSGANVLASGPF-TG
OeuTIP5;2       GFGAAILEGVMTFTLVYTVYA-AADPRR----GSMSIIGPLAIGLVSGANVLASGPF-TG
OeuTIP5;3       GFGGAVLEGVMTFALVYTVYV-ASNLRP----VSAGAIGPLAIGLIAGANVLASGPF-TG
OeuNIP1;1       DMQSFVVEFIITFYLMFVISGVATDNRA------IGELAGLAIGATILLNVMIAGPV-SG
OeuNIP1;2       DVQSFMIEFITTFYLMFVISGVATDNRA------IGELKGIAIGATLSLNILFAGPI-SG
OeuNIP1;3       DLQSFVMEFIITFYLMFVISGVATDNRA------IGELSGLAIGATILLNVMFAGPV-SG
OeuNIP3;1       DLEAITWEFVITFIFMLTIRGVASDDRA------L---------------QLELQPI-TG
OeuNIP4;1       NGQSFAIEIIISFLLMFTISGVATDSRA------IGDLAGLVVGMTIMLNVFVAGPI-SG
OeuNIP5;1       IGQAFALEFLITFNLLFVVTAVATDTRA------VGELAGIAVGATVMLNILVAGPS-SG
OeuNIP5;2       TGQAFALEFLITFNLLFVVTAVATDTRA------VGELAGIAVGATVMLNILVAGPS-SG
OeuNIP7;1       CTAAFWVELIATFIILFLTASLSNDPQS------IGQVSGFVVGVAIGLGVLISGPVSGG
OeuXIP1;2       TVQAFWLEIFCTFVFLFASIWMAYDPRQAKELGHVLIFSIVGVVLGLLVFISTTVTAKKG
OeuXIP1;3       TAQAFWLEIICTFVLLFASVWMAYDHRQAKALGRVLVFSIVGVVLGLLVFISTTVTAKKG
NtTIPa          YGQGIIMEVILTFSLLFTVYTTIVDPKK----GILEGMGPLLTGLVVGANIMAGGPF-SG
AtTIP4;1        YTQGIIWEIILTFSLLFTVYATIVDPKK----GSLDGFGPLLTGFVVGANILAGGAF-SG
AtTIP1;1        VLNAFVFEIVMTFGLVYTVYATAIDPKN----GSLGTIAPIAIGFIVGANILAGGAF-SG
AtTIP1;2        SLNALVFEIVMTFGLVYTVYATAVDPKN----GSLGTIAPIAIGFIVGANILAGGAF-SG
AtTIP1;3        PWNAVVFEIVMTFGLVYTVYATAVDPKK----GDIGIIAPLAIGLIVGANILVGGAF-DG
AtTIP2;1        SIEGVVMEIIITFALVYTVYATAADPKK----GSLGTIAPLAIGLIVGANILAAGPF-SG
AtTIP5;1        GFGASVLEGVLAFVLVYTVFT-ASDPRR----GLPLAVGPIFIGFVAGANVLAAGPF-SG
NtAQP1          KGDGLGAEIIGTFVLVYT-VFSATDAKRNARDSYVPILAPLPIGFAVFLVHLATIPI-TG
ZmPIP1;5        KGDGLGAEIVGTFVLVYT-VFSATDAKRSARDSHVPILAPLPIGFAVFLVHLATIPI-TG
NtXIP1;1α       MAQALWLEIFCTFVFLFASIWMAYDHRQAKALGLVTVLSIVGIVLGLLVFISTTVTMKKG
NtXIP1;1β       MAQALWLEIFCTFVFLFASIWMAYDHRQAKALGLVTVLSIVGIVLGLLVFISTTVTMKKG
StXIP1;1α       TAQALWLEIFCTFVFLFASIWMAYDHRQAKALGHVTVLSIVGLVLGLLVFISTTVTAKKG
StXIP1;1β       TAQALWLEIFCTFVFLFASIWMAYDHRQAKALGHVTVLSIVGLVLGLLVFISTTVTAKKG
CpNIP1          DLQALVMEIVVTFSMMFVTCAVATDTKA------VGELAGLAVGSAVCITSILAGPV-SG
OsNIP2;1        HWHSLVVEVIVTFNMMFVTLAVATDTRA------VGELAGLAVGSAVCITSIFAGAI-SG
AtNIP6;1        LSQAFALEFIISFNLMFVVTAVATDTRA------VGELAGIAVGATVMLNILIAGPA-TS
                   .     . :* ::          :                            .
```

```
OeuPIP1;1a      ---TGINPARSLGAAIIYNKDKAWDD-HWIFWVGPFIGAALAALYHVVVIRAIPFKK---
OeuPIP1;1b      ---TGINPARSLGAAIIYNKDKAWDD-HWIFWVGPFIGAALAALYHVVVIRAIPFKK---
OeuPIP1;1c      ---TGINPARSLGAAIIYNKDKAWDD-HWIFWVGPFIGAALAALYHVVVIRAIPFKK---
OeuPIP1;2       ---TGINPARSLGAAIIYNKDKAWDD-HWIFWVGPFIGAALAALYHVVVIRAIPFKNK--
OeuPIP1;3       ---TGINPARSLGAAIIYNRDHAWDD-QWIFWVGPFIGAALAAIYHQIVIRAIPFKSRA-
OeuPIP1;4       ---TGINPARSLGAAIIYNKDHAWDD-HWIFWVGPFIGAALAALYHQVIIRAIPFKSSS-
OeuPIP1;5       ---TSINPARSLGAAIIYNRDNAWDD-HWIFWVGPFIGAALAAIYHQIVIRAIPFKSSQA
OeuPIP1;6       ---TGINPARSLGAAIIYNKDHAWDD-HWIFWVGPFIGAALAALYHQVIIRAIPFKSGN-
OeuPIP2;1       ---TGINPARSFGAAVIYNEDKPWDD-HWIFWVGPFVGALIAAIYHQYILRAGAIKALGS
OeuPIP2;2       ---TGINPARSFGAAVIYNKDKPWDD-HWIFWVGPFIGAAIAAFYHQYILRAAAIKALGS
OeuPIP2;3       ---TGINPARSFGAAVIYNQEKAWDD-QWIFWVGPFIGAAIAAFYHQFILRAGAAKALGS
OeuPIP2;4       ---TGINPARSLGAAVIYNQEKAWDD-QWIFWVGPFFGAAIAAFYHQFILRAGAAKALGS
OeuPIP2;5       ---TGINPARSLGAAVIYGKDKAWDD-QWIFWVGPFIGAAIAAFYYQYILRAGAVKALGS
OeuPIP2;6       ---TGINPARSLGAAVIYDQHKAWDD-HWIFWVGPFIGAAVAAFYHQFILRAGAAKALGS
OeuPIP2;7       ---TGINPARSFGAAVIYGKDKAWDD-QWIFWAGPFIGAAIAALYHQYILRAGAVKALGS
OeuPIP2;8       ---TGINPARSFGAAVIYNNDKVWDD-HWIFWVGPFVGALAAAAYHQYILRAAAIKAIGS
OeuPIP2;9       ---TGINPARSFGAAVIFNNDKVWDD-HWIFWVGPFVGALAAAAYHQYILRAAAIKALGS
OeuPIP2;10      ---TSINPARSFGPAVIYNQKKAWDD-QWIFWVGPFIGAAMAALYHQCILRA---KAFRS
OeuPIP2;11      ---TGINPARSLGAAVIYNQDKAWDD-LWIFWVGPFIGAAIAAFYHQFILRAGAVKALGS
OeuTIP1;1       ---ASMNPAVSFGP---AVVSWTWDS-HWVYWLGPFVGAGIAALVYEILFINQS------
OeuTIP1;2       ---ASMNPAVSFGP---AVVSWTWDS-HWVYWLGPFVGAGIAAIVYEIVFINQT------
OeuTIP1;3       ---ASMNPAVSFGP---ALVSFTWTH-HWVYWAGPLIGGGLAGIVYEVFFVNH-------
OeuTIP1;4       ---ASMNPAVSFGP---AVVSWTWDN-HWVFWLGPFVGAGIAALIYDILFINQS------
OeuTIP1;5       ---ASMNPAVSFGP---AVVSWTWDS-HWVFWLGPFVGAGIAALIYEILFINQS------
OeuTIP1;6       ---ASMNPAVSFGP---AVVSLS-------------------------VFDQKD------
OeuTIP1;7       ---ASMNPAVSFGP---AVVSWTWDN-HWVYWAGPLIGGGLAGLIYEFFFINQ-------
OeuTIP2;1       ---GSMNPARSFGP---AVASGNFSG-IWIYWVGPLIGGGLAGLVYAYVFM-SH------
OeuTIP2;2       ---GSMNPARSFGP---AVASGNFSG-IWIYWVGPLIGGGLAGLVYAYVFM-SH------
OeuTIP2;3       ---GSMNPARSFGP---AVASGDFAD-NWIYWVGPLIGGGLAGLVYAYVFM-VH------
OeuTIP2;4       ---GSMNPARSFGP---AVAAGDFSQ-NWIYWVGPLIGGGLAGLVYGYVFIASY------
OeuTIP2;5       ---GSMNPARSFGP---AVAAVDFSQ-NWIYWVGPLIGGGLAGLVYGDVFIGSY------
OeuTIP3;1       ---ASMNPARAFGP---ALVGWRWKD-HWVYWLGPFVGAALAGVIYEFGVIQTETPHPTH
OeuTIP4;1       ---ASMNPARSFGP---ALVSGDWTD-HWVYWVGPLIGGGLAGYIYENFFIVRS------
OeuTIP5;1       ---GSMNPAYSFGS---ALVGGSFKN-QAVYWIGPLIGAATAGILYEHVVFPAQQ-----
OeuTIP5;2       ---GSMNPAYSFGS---ALVGGSFKN-QAVYWIGPLIGAATAGILYEHVVFPAQQ-----
OeuTIP5;3       ---GSMNPAYAFGS---AIVGGSFKN-QAVYWVGPLFGAAIAGILHDNVVCPAP------
OeuNIP1;1       ---ASMNPARSLGP---AIVSNKYKG-IWIYIVGPTLGAVAGAWVYNIIRFTNKPLREIT
OeuNIP1;3       ---ASMNPVRSLGP---AIVSNRYEG-IWIYIVGPTLGAIAGAWAYNIIRFTDKPLREIT
OeuNIP1;2       ---ASLNPARSLGP---AIVYNRYEG-LWNYILAPISGAIAGAWVYNFIRFTDKPLREII
OeuNIP3;1       ---ASMNPARSMGP---AIVSGVYKN-QWVYVVAPILGAIAASLIYSLLQPWKPEKHRES
OeuNIP4;1       ---ASMNPARSIGP---AIVKHTYKG-LWVYIFGPLIGTILGGFAYNLIRYTNKPLRELT
OeuNIP5;1       ---GSMNPVRTLGP---AVAAGNYKA-VWIYLLAPTLGALAGAGIYTLVKLNGEEEEAPS
OeuNIP5;2       ---GSMNPVRTLGP---AVAAGNYKV-IWVYLLAPTLGALAGAGIYTLVKLNGQEEEVPR
OeuNIP7;1       ---GSMNPARSLGP---ALVSWKFDDHLWIYLVAPTIGAIAGVFVYRILRLQGWSCESSQ
OeuXIP1;2       YAGAGMNPARCFGPAIVRG-GHLWNV-YWVFWAGPTIACVAFYLYTQIIPCQHHRSKYSY
OeuXIP1;3       YAGAGMNPARCFGAAVVRG-GHLWNG-HWIFWVGPAIACVAFYLYTKIIPRQHYQAKF-Y
NtTIPa          ---ASMNPARSFGP---AFVSGIWTD-HWVYWVGPLIGGGLAGFICENFFIVRT------
AtTIP4;1        ---ASMNPARSFGP---ALVSGNWTD-HWVYWVGPLIGGGLAGFIYENVLIDRP------
AtTIP1;1        ---ASMNPAVAFGP---AVVSWTWTN-HWVYWAGPLVGGGIAGLIYEVFFINT-------
AtTIP1;2        ---ASMNPAVAFGP---AVVSWTWTN-HWVYWAGPLIGGGLAGIIYDFVFIDEN------
AtTIP1;3        ---ASMNPAVSFGP---AVVSWIWTN-HWVYWVGPFIGAAIAAIVYDTIFIGSN------
AtTIP2;1        ---GSMNPARSFGP---AVAAGDFSG-HWVYWVGPLIGGGLAGLIYGNVFMGSS------
AtTIP5;1        ---GSMNPACAFGS---AMVYGSFKN-QAVYWVGPLLGGATAALVYDNVVVPVED-----
NtAQP1          ---TGINPARSLGAAIIYNTDQAWDD-HWIFWVGPFIGAALAAVYHQIIIRAIPFHKSS-
ZmPIP1;5        ---TGINPARSLGAAIVYNRSHAWND-HWIFWVGPFIGAALAAIYHVVIIRALPFKSRD-
NtXIP1;1α       YAGAGMNPARCFGAAVVRG-GHLWDG-HWIFWVGPTIACVAFYVYTKIIPPKHFHAD-GY
NtXIP1;1β       YAGAGMNPARCFGAAVVRG-GHLWDG-HWIFWVGPTIACVAFYVYTKIIPPKHFHAD-GY
StXIP1;1α       YGGAGINPARCLGPAIIRG-GHLWDG-HWIFWVGPTIGCVAFYVYTKIIPPKHFLAEYGF
StXIP1;1β       YGGAGINPARCLGPAIIRG-GHLWDG-HWIFWVGPTIGCVAFYVYTKIIPPKHFLAEYGF
CpNIP1          ---GSMNPVRTLGP---AMASDNYKG-LWVYFVGPVTGTLLGAWSYKFIRASDKPVHLIS
OsNIP2;1        ---GSMNPARTLGP---ALASNKFDG-LWIYFLGPVMGTLSGAWTYTFIRFEDTPKEGSS
AtNIP6;1        ---ASMNPVRTLGP---AIAANNYRA-IWVYLTAPILGALIGAGTYTIVKLP-EEDEAPK
                    .:**.  :*.
```
