# Supplementary material for: Genome Wild Analysis and Molecular Understanding of the Aquaporin Diversity in Olive Trees (Olea Europaea L.)
Source: Int J Mol Sci. 2020 Jun 11;21(11):4183. doi: 10.3390/ijms21114183 (PMC7312470; doi:10.3390/ijms21114183)
Supplement: Supplementary file 1 [file ijms-21-04183-s001.zip › ijms-821530_Supplementary_files/Table S1_Faize_OeuAQP_compilation.pdf]

Table S1. Nomenclatures of *Oeu*APQ sequences from the wild olive specie *Olea europaea* var. *sylvestris*.
*Oeu*AQP sequences (genomic, CDS and protein) are detailled in Supplemental FigureS1

MIP Subclasses	Phytozom loci ("Pseudogenes" in red)	UniProtKB - *Olea europaea* * Secchi et al., 2007	Location	Nucleotide location (location corrected in red, if necessary)	Remarks
	***Oeu*038800.1** *Oeu*PIP1;1a	A0A385A1M4 / A0A385A1N7 GN=PIP1.1 ABB13429.1*	chr19	7203994..7205679 forward	Exon 1 extended with 63n
	***Oeu*038801.1** *Oeu*PIP1;1b	A0A385A1M4 / A0A385A1N7 GN=PIP1.1 ABB13429.1*	chr19	7208573..7210258 reverse	Exon 1 extended with 63n
	***Oeu*038802.2** *Oeu*PIP1;1c	A0A385A1M4 / A0A385A1N7 GN=PIP1.1 ABB13429.1*	chr19	7217774..7219459 forward	Exon 1 extended with 63n
	***Oeu*018133.1** *Oeu*PIP1;2	A0A1D5AHZ6 GN=PIP1.2	scaffold17619	410..2065 forward	-
	***Oeu*042329.1** *Oeu*PIP1;3	A0A1D5AHZ7 GN=PIP1.3	chr13	11012950..11015602 forward	-
**PIP1**	***Oeu*052954.1** *Oeu*PIP1;4	-	chr3	21471493..21473698 reverse	-
	***Oeu*061384.2** *Oeu*PIP1;5	-	scaffold87	524214..526575 reverse	Third predicted Exon as part of the second intron
	***Oeu*046350.1** *Oeu*PIP1;6	-	scaffold4751	4496..7173 forward	-
	***Oeu*022396.1** *Oeu*PIP1-related	-	scaffold1994	48682..48948 forward	Incomplete sequence
	***Oeu*010785.1** *Oeu*PIP1-related	-	chr3	8511236..8513098 reverse	Incomplete sequence
	***Oeu*019177.1** *Oeu*PIP1-related	-	chr8	2118675..2120753 reverse	Incomplete sequence
	***Oeu*018831.2** *Oeu*PIP1-related	-	chr4	15354575..15359491 forward	Incomplete sequence
	***Oeu*030183.1** *Oeu*PIP2;1	Q38PG7 / ABB13430.2*	chr4	8070191..8071649 reverse	-
	***Oeu*001271.1** *Oeu*PIP2;2	-	scaffold1031	177815..179775 reverse	-
	***Oeu*033842.1** *Oeu*PIP2;3	-	chr12	7848155..7849789 reverse	-
	***Oeu*030439.1** *Oeu*PIP2;4	-	scaffold2530	66057..67614 forward	-
	***Oeu*027987.1** *Oeu*PIP2;5	-	scaffold2335	58720..59930 reverse	-
	***Oeu*017257.1** *Oeu*PIP2;6	-	chr6	11700750..11703098 forward	-
**PIP2**	***Oeu*018111.1** *Oeu*PIP2;7	-	scaffold176	1251179..1252336 forward	-
	***Oeu*023232.1** *Oeu*PIP2;8	-	scaffold20321	54..1165 reverse	Exon 1 extended in its 5'end with 84n
	***Oeu*020106.1/Scaffold870** *Oeu*PIP2;9	-	scaffold18654 scaffold870	1671..2018 forward 312817..313578 forward	linking between 2 scaffolds
	***Oeu*022438.1/scaffold2530** *Oeu*PIP2;10	-	scaffold19979 scaffold2530	1..1328 reverse	5' end extended with 66n 3' end extended with the scaffold 2530
	***Oeu*017254.1/*Oeu*017256.1** *Oeu*PIP2;11	-	chr6	11625929..11647223 forward	PIP sequence cleaved by 21268n including *Oeu*17255 gene
	***Oeu*059136.1** *Oeu*PIP2-related	-	chr10	15785080..15785518 reverse	Incomplete sequence
	***Oeu*058531.1** *Oeu*TIP1;1	Q2XVV9 / ABB76813.1* GN=TIP	chr22	3077808..3078742 forward	-
	***Oeu*041014.1** *Oeu*TIP1;2	A0A1C8V910 GN=TIP1.2	chr20	11814751..11816218 reverse	-
	***Oeu*035479.1** *Oeu*TIP1;3	A0A1D5AHZ9 GN=TIP1.3	scaffold3011	24603..25462 forward	-
	***Oeu*040290.1** *Oeu*TIP1;4	-	chr4	16254381..16255311 reverse	-
	***Oeu*040289.1** *Oeu*TIP1;5	-	chr4	16257013..16257943 reverse	-
	***Oeu*033244.1** *Oeu*TIP1;6	-	scaffold2789	55535..57322 forward	-
	***Oeu*045237.1** *Oeu*TIP1;7	-	chr11	5861757..5862731 reverse	2059n were removed at the 5' extremity
	***Oeu*024074.1** *Oeu*TIP2;1	-	chr2	21805437..21806390 reverse	-
	***Oeu*024076.1** *Oeu*TIP2;2	-	chr2	21795643..21796596 reverse	-
	***Oeu*053988.1** *Oeu*TIP2;3	-	chr22	5185229..5186864 forward	-
**TIP**	***Oeu*002329.1** *Oeu*TIP2;4	-	chr13	8623502..8625525 reverse	-
	***Oeu*040207.1** *Oeu*TIP2;5	-	chr19	9881246..9883023 forward	Intron 3 converted in exon
	***Oeu*038387.1** *Oeu*TIP3;1	-	chr10	29024107..29025120 forward	-
	***Oeu*030229.1-*Oeu*036364.1** *Oeu*TIP4;1	-	scaffold25131 scaffold311	282..785 reverse 584778..587098 forward	-
	***Oeu*021708.1** *Oeu*TIP5;1	-	chr12	21524153..21525389 reverse	-
	***Oeu*021709.1** *Oeu*TIP5;2	-	chr12	21526411..21527647 reverse	-
	***Oeu*022479.1** *Oeu*TIP5;3	-	chr1	6772133..6773251 reverse	-
	***Oeu*041020.1** *Oeu*TIP-related	-	chr20	11792223..11792531 for	Incomplete sequence
	***Oeu*007734.1** *Oeu*TIP1;3-related	-	chr4	20127443..20127820 reverse	Incomplete sequence
	***Oeu*000463.1** *Oeu*TIP2;2-related	-	chr17	6142042..6142582 forward	Incomplete sequence
	***Oeu*050612.1** *Oeu*TIP2;1-related	-	chr18	23502061..23503102 forward	Pseudogene

**XIP**	***Oeu*010056.1**<br>*Oeu*XIP1;1	-	chr3	335356..336578 forward	Exon 1 extended with 21n
	***Oeu*020684.1**<br>*Oeu*XIP1;1	-	chr5	9245927..9248997 reverse	Extended in 5' with 1455n
	***Oeu*046122.1**<br>*Oeu*XIP1;3	-	chr16	11367503..11369530 reverse	Extended in 5'
**NIP**	***Oeu*060187.1**<br>*Oeu*NIP1;1	-	chr21	7140088..7142220 forward	-
	***Oeu*040934.1**<br>*Oeu*NIP1;2	-	chr13	1731124..1733943 forward	5' extended with 2042n,<br>generating 3 exons and 3 introns.
	***Oeu*039868.1**<br>*Oeu*NIP1;3	-	chr16	12429545..12432818 reverse	5' extended with 792n<br>3' truncated of 246n<br>Intron/exon readjusted
	***Oeu*053221.1a**<br>*Oeu*NIP3;1	-	Saffold 635	392813..394288 forward	-
	***Oeu*047418.1**<br>*Oeu*NIP4;1	-	chr18	8000311..8001846 forward	-
	***Oeu*020545.1**<br>*Oeu*NIP5;1	-	chr18	6958376..6972407 forward	-
	***Oeu*046374.1**<br>*Oeu*NIP5;2	-	scaffold476	396474..398858 forward	-
	***Oeu*060126.1**<br>*Oeu*NIP6;1	-	scaffold830	250629..252602 forward	-
	***Oeu*011481.1**<br>*Oeu*NIP6-related	-	scaffold14354	165..1696 reverse	Incomplete in 3' end
	***Oeu*037688.1**<br>*Oeu*NIP7;1	-	chr4	6837162..6838876 forward	1841n removed at the 5' end<br>Intron/exon redesigned by homology with<br>Oleur061Scf4622g04016.1
	***Oeu*053221.1b**<br>*Oeu*NIP3;1-related	-	Saffold 635	402535..403011 forward	Incomplete sequence<br>End of the Scaffold635
	***Oeu*Chr8**<br>*Oeu*NIP4;2-related	-	chr8	17852182..17858508 forward	Sequence non-annoted in Phytozome database<br>Incomplete sequence
	***Oeu*053221.1b**<br>*Oeu*NIP1-related	-	scaffold635	402535..403011 forward	Incomplete sequence<br>3' end stopped by an other gene
	***Oeu*017634.1**<br>*Oeu*NIP5-related	-	scaffold1737	102936..103291 forward	Incomplete sequence
	***Oeu*005264.1**<br>*Oeu*NIP7-related	-	scaffold11917	939..1312 reverse	Incomplete sequence
**SIP**	***Oeu*060749.1**<br>*Oeu*SIP2;1	B0L1W3<br>SIP1.1	chr9	9170322..9172494 forward	CDS elongated in 3'<br>(intron + exon) with 261n
	***Oeu*Chr11**<br>*Oeu*SIP2;2	B5KGP0<br>SIP1.2	chr11	18362597..18364222 reverse	Sequence non-annoted in Phytozome database
	***Oeu*050292.1**<br>*Oeu*SIP1;1	-	chr4	9340524..9350151 reverse	-
	***Oeu*014723.2**<br>*Oeu*SIP1;2	-	scaffold1584	16236..19503 reverse	-
	***Oeu*061896.1**<br>*Oeu*SIP1-related	-	chr22	4366217..4368873 forward	Pseudogene<br>(Oleur061Scf0342g12031.1 homolog)
	***Oeu*050293.1**<br>*Oeu*SIP-related	-	chr4	9356974..9357960 reverse	Incomplete sequence<br>Intron 2 and Exon 3 truncated
