# Supplementary material for: Genome Wild Analysis and Molecular Understanding of the Aquaporin Diversity in Olive Trees (Olea Europaea L.)
Source: Int J Mol Sci. 2020 Jun 11;21(11):4183. doi: 10.3390/ijms21114183 (PMC7312470; doi:10.3390/ijms21114183)
Supplement: Supplementary file 1 [file ijms-21-04183-s001.zip › ijms-821530_Supplementary_files/Table S2_Faize_OeuAQP_OleurAQP.pdf]

Table S2. Correspondance between aquaporin gene sequences from *Olea europaea* var. *sylvestris* (*Oeu*APQ) and *Olea europaea* cv. Picual (*Oleur*AQP)

*Oeu*AQPs were extracted from Phytozome V12 portail (https://phytozome.jgi.doe.gov/pz/portal.html#!search?show=BLAST&method=Org_Oeuropaea_er)

*Oleur*AQPs were extracted from OliveTreeDB database (https://genomaolivar.dipujaen.es/db/index.php)

*Oeu*AQP sequences (genomic, CDS and protein) are detailled in Supplemental FigureS1

*Oleur*AQP sequences (genomic, CDS and protein) are listed in the Supplemental FigureS2

Proposed nomenclature was established by comparaison with orthologuous aquaporins from *A. thaliana*, *L. esculentum. F. excelsior and P. trichocarpa*

Legends :

Pseudogenes and Incomplete sequences are mentionned in red.

MIP subclasses	Phytozome V12	OliveTreeDB *Olea europaea* L.	
	Proposed Nomenclature	Proposed Nomenclature	Locus
PIP1	***Oeu*038800.1** / *Oeu*PIP1;1a<br>***Oeu*038801.1** / *Oeu*PIP1;1b<br>***Oeu*038802.2** / *Oeu*PIP1;1c	*Oleur*PIP1;1	Oleur061Scf2846g07022.1 ; 701124..702805 (- strand)
	***Oeu*018133.1** / *Oeu*PIP1;2	*Oleur*PIP1;2a	Oleur061Scf0176g00018.1 ; 63114..64769 (+ strand)
		*Oleur*PIP1;2b	Oleur061Scf0994g03003.1 ; 369686..371341 (+ strand)
	***Oeu*042329.1** / *Oeu*PIP1;3	*Oleur*PIP1;3a	Oleur061Scf1119g10041.1 ; 1019235..1022315 (- strand)
		*Oleur*PIP1;3b	Oleur061Scf3031g04003.1 ; 499618..502336 (+ strand)
	***Oeu*052954.1** / *Oeu*PIP1;4	*Oleur*PIP1;4a	Oleur061Scf0931g02045.1 ; 329811..332344 (- strand)
		*Oleur*PIP1;4b	Oleur061Scf0180g12067.1 ; 1186334..1190261
	***Oeu*061384.2** / *Oeu*PIP1;5	*Oleur*PIP1;5a	Oleur061Scf2999g01010.1 ; 181146..184008 (- strand)
		*Oleur*PIP1;5b	Oleur061Scf0451g01009.1 ; 160386..163969
	***Oeu*046350.1** / *Oeu*PIP1;6	*Oleur*PIP1;6a	Oleur061Scf6143g00002.1 ; 10938..14002 (- strand)
		*Oleur*PIP1;6b	Oleur061Scf8779g00011.1 ; 132329..136726
	***Oeu*022396.1** / *Oeu*PIP1-related	*Oleur*PIP1-related	Oleur061Scf0830g11020.1 *Pseudogene*
	***Oeu*018831.2** / *Oeu*PIP1-related	*Oleur*PIP1-related	Oleur061Scf0761g01005.1 *Pseudogene*
	-	*Oleur*PIP1-related	Oleur061Scf2581g00016.1
	-	*Oleur*PIP1-related	Oleur061Scf0830g08007.1 *Incomplete sequence*
	-	*Oleur*PIP1-related	Oleur061Scf0830g08009.1 *Incomplete sequence*
	-	*Oleur*PIP1-related	Oleur061Scf0830g09001.1 *Incomplete sequence*
	-	*Oleur*PIP1-related	Oleur061Scf0830g09023.1 *Incomplete sequence*
	-	*Oleur*PIP1-related	Oleur061Scf0830g09012.1 *Incomplete sequence*
	-	*Oleur*PIP1-related	Oleur061Scf0830g10010.1 *Incomplete sequence*
	-	*Oleur*PIP1-related	Oleur061Scf1121g01015.1 *Incomplete sequence*
	-	*Oleur*PIP1-related	Oleur061Scf1121g01024.1 *Incomplete sequence*
	-	*Oleur*PIP1-related	Oleur061Scf1121g01006.1 *Incomplete sequence*
	***Oeu*010785.1** / *Oeu*PIP1-related	No hits found	-
	***Oeu*019177.1** / *Oeu*PIP1-related	No hits found	-
PIP2	***Oeu*030183.1** / *Oeu*PIP2;1	*Oleur*PIP2;1a	Oleur061Scf0523g01011.1 ; 148832..151185 (+ strand)
		*Oleur*PIP2;1b	Oleur061Scf0872g05024.1 ; 512608..514991 (+ strand)
	***Oeu*001271.1** / *Oeu*PIP2;2	*Oleur*PIP2;2a	Oleur061Scf6622g04008.1 ; 431311..433104 (- strand)
		*Oleur*PIP2;2b	Oleur061Scf8869g00006.1 ; 18552..20344 (- strand)
	***Oeu*033842.1** / *Oeu*PIP2;3	*Oleur*PIP2;3a	Oleur061Scf0690g01039.1 ; 166917..168873 (- strand)
		*Oleur*PIP2;3b	Oleur061Scf8232g00023.1 ; 53550..55667 (+ strand)
	***Oeu*030439.1** / *Oeu*PIP2;4	*Oleur*PIP2;4	Oleur061Scf0101g01002.1 ; 84671..86479 (- strand)
	***Oeu*027987.1** / *Oeu*PIP2;5	*Oleur*PIP2;5	Oleur061Scf3195g08011.1 ; 828028..829785 (+ strand)
	***Oeu*017257.1** / *Oeu*PIP2;6	*Oleur*PIP2;6a	Oleur061Scf0106g06001.1 ; 611096..613917 (- strand)
		*Oleur*PIP2;6b	Oleur061Scf0638g02012.1 ; 213705..216526 (- strand)
	***Oeu*018111.1** / *Oeu*PIP2;7	*Oleur*PIP2;7	Oleur061Scf6451g01042.1 ; 160614..162280 (+ strand)
	***Oeu*023232.1** / *Oeu*PIP2;8	*Oleur*PIP2;8a	Oleur061Scf3978g00008.1 ; 91118..92620 (- strand)
		*Oleur*PIP2;8b	Oleur061Scf0410g01025.1 ; 167787..169203 (- strand)
	***Oeu*020106.1 / Scaffold870** / *Oeu*PIP2;9	*Oleur*PIP2;9a	Oleur061Scf7760g00005.1 ; 51176..52742 (- strand)
		*Oleur*PIP2;9b	Oleur061Scf1616g00016.1 ; 35879..37453 (+ strand)
	***Oeu*022438.1 / Scaffold2530** / *Oeu*PIP2;10	*Oleur*PIP2;10	Oleur061Scf0101g01021.1 ; 86880..93288 (- strand)
	***Oeu*017254.1 / *Oeu*017256.1** / *Oeu*PIP2;11	*Oleur*PIP2;11	Oleur061Scf0106g06008.1 ; 626873..647970 length=21098
	No hit found	*Oleur*PIP2;12	Oleur061Scf0156g01015.1 ; 141041..142902 (- strand)
	***Oeu*059136.1** / *Oeu*PIP2-related	*Oleur*PIP2-related	Oleur061Scf6854g04027.1 *Incomplete* sequence
	-	*Oleur*PIP2-related	Oleur061Scf2856g00010.1 *Incomplete sequence*
		*Oleur*PIP2;7-related	Oleur061Scf6288g00002.1 *Incomplete sequence*
		*Oleur*PIP2;10-related	Oleur061Scf0243g01010.1 *Incomplete sequence*
		*Oleur*PIP2;11-related	Oleur061Scf0638g02004.1 *Incomplete sequence*

**TIP**	***Oeu*058531.1** / *Oeu*TIP1;1	*Oleur*TIP1;1	Oleur061Scf3090g01006.1 ; 93487..94956 (- strand)
	***Oeu*041014.1** / *Oeu*TIP1;2	*Oleur*TIP1;2a<br>*Oleur*TIP1;2b	Oleur061Scf8429g00004.1 ; 141483..143267 (+ strand)<br>Oleur061Scf0417g02009.1 ; 223466..225244 (- strand)
	***Oeu*035479.1** / *Oeu*TIP1;3	*Oleur*TIP1;3a<br>*Oleur*TIP1;3b<br>*Oleur*TIP1;3c<br>*Oleur*TIP1;3d<br>*Oleur*TIP1;3e	Oleur061Scf1330g06004.1 ; 642994..644469 (- strand)<br>Oleur061Scf4360g00007.1 ; 27330..28805 (+ strand)<br>Oleur061Scf7549g01032.1 ; 241767..243178 (- strand)<br>Oleur061Scf0466g03012.1 ; 372836..374085 (- strand)<br>Oleur061Scf3806g00010.1 ; 21034..22285 (+ strand)
	***Oeu*040290.1** / *Oeu*TIP1;4	*Oleur*TIP1;4-related	Oleur061Scf5170g00024.1 Incomplete sequence
	**Oeu040289.1** / OeuTIP1;5	*Oleur*TIP1;5	Oleur061Scf7653g04013.1 ; 468560..470420 (- strand)
	***Oeu*033244.1** / *Oeu*TIP1;6	*Oleur*TIP1;6	Oleur061Scf5512g00068.1 ; 141526..147261 (- strand)
	***Oeu*045237.1** / *Oeu*TIP1;7	*Oleur*TIP1;7<br>*Oleur*TIP1;7-related	Oleur061Scf3360g04010.1 ; 442951..444443 (- strand)<br>Oleur061Scf3142g00008.1 ; 34430..35603 (+ strand)
	Oeu*024074.1** / *Oeu*TIP2;1<br>Oeu*024076.1** / *Oeu*TIP2;2	*Oleur*TIP2;1	Oleur061Scf1395g09059.1 ; 971893..975231 (+ strand)
	***Oeu*053988.1** / *Oeu*TIP2;3	*Oleur*TIP2;3a<br>*Oleur*TIP2;3b	Oleur061Scf5616g00030.1 ; 47019..49189 (- strand)<br>Oleur061Scf6190g00017.1 ; 16438..18599 (+ strand)
	***Oeu*002329.1** / *Oeu*TIP2;4	*Oleur*TIP2;4	Oleur061Scf2185g00004.1 ; 106483..109800 (- strand)
	***Oeu*040207.1** / *Oeu*TIP2;5	*Oleur*TIP2;5a<br>*Oleur*TIP2;5b	Oleur061Scf1607g00003.1 ; 86016..87968 (+ strand)<br>Oleur061Scf8992g00006.1 ; 66181..68174 (+ strand)
	***Oeu*038387.1** / *Oeu*TIP3;1	*Oleur*TIP3;1a<br>*Oleur*TIP3;1b<br>*Oleur*TIP3;1c	Oleur061Scf6254g04008.1 ; 448318..449463 (+ strand)<br>Oleur061Scf5220g00047.1 ; 6075..7220 (- strand)<br>Oleur061Scf3963g07030.1 ; 728110..729328 (+ strand)
	***Oeu*030229.1/ *Oeu*036364.1** / *Oeu*TIP4;1	*Oleur*TIP4;1	Oleur061Scf6420g00014.1 ; 44973..52364 (- strand)
	***Oeu*021708.1** / *Oeu*TIP5;1	*Oleur*TIP5;1	Oleur061Scf8528g00015.1 ; 7335..8665 (+ strand)
	***Oeu*021709.1** / *Oeu*TIP5;2	*Oleur*TIP5;2	Oleur061Scf0430g00024.1 ; 47031..48350 (- strand)
	***Oeu*022479.1** / *Oeu*TIP5;3	*Oleur*TIP5;3a<br>*Oleur*TIP5;3b	Oleur061Scf7085g01025.1 ; 145441..146632 (- strand)<br>Oleur061Scf2746g00014.1 ; 4573..5762 (- strand)
	-	*Oleur*TIP1;1-related<br>*Oleur*TIP2;4-related<br>*Oleur*TIP3;1-related	Oleur061Scf0505g01006.1 *Incomplete sequence*<br>Oleur061Scf6253g02001.1 *Pseudogen (3'end reading frame shifted)*<br>Oleur061Scf6179g00017.1 *Incomplete sequence*
	Oeu*041020.1** / *Oeu*TIP-related *Incomplete sequence*<br>Oeu*007734.1** / *Oeu*TIP1;3-related *Incomplete sequence*	Similar to *Oleur*TIP1;2a<br>Similar to *Oleur*TIP1;3e	-
	Oeu*050612.1** / *Oeu*TIP2;1-related *Pseudogene*<br>Oeu*000463.1** / *Oeu*TIP2;2-related *Incomplete sequence*	*Oleur*TIP2-related<br>*Oleur*TIP2-related	Oleur061Scf3986g06001.1 *Pseudogene*<br>Oleur061Scf2246g01050.1 *Incomplete sequence*
	-	*Oleur*TIP1-related<br>*Oleur*TIP1-related<br>*Oleur*TIP1-related<br>*Oleur*TIP2-related<br>*Oleur*TIP2-related<br>*Oleur*TIP4-related<br>*Oleur*TIP5;1-related	Oleur061Scf2451g01004.1 *Pseudogene*<br>Oleur061Scf2960g01064.1 *Pseudogene*<br>Oleur061Scf6390g00005.1 *Pseudogene*<br>Oleur061Scf6342g00008.1 *Incomplete sequence*<br>Oleur061Scf2246g01050.1 *Incomplete sequence*<br>Oleur061Scf3307g00019.1 *Incomplete sequence*<br>Oleur061Scf0238g01010.1 *Incomplete sequence*
**XIP**	***Oeu*010056.1** / *Oeu*XIP1;1	*Oleur*XIP1;1a<br>*Oleur*XIP1;1b	Oleur061Scf1407g06005.1 ; 601677..603499 (- strand)<br>Oleur061Scf4628g08012.1 ; 814896..816717 (+ strand)
	***Oeu*020684.1** / *Oeu*XIP1;2	*Oleur*XIP1;2a<br>*Oleur*XIP1;2b	Oleur061Scf0694g04001.1 ; 467273..468886 (- strand)<br>Oleur061Scf0386g00009.1 ; 689..2303 (- strand)
	***Oeu*046122.1** / *Oeu*XIP1;3	*Oleur*XIP1;3-related<br>*Oleur*XIP1;3	Oleur061Scf0258g01032.1 *Pseudogene*<br>Oleur061Scf1977g00011.1 ; 43695..44812 (- strand)
	-	*Oleur*XIP1;2-related<br>*Oleur*XIP1;2-related<br>*Oleur*XIP1;2-related<br>*Oleur*XIP1;2-related<br>*Oleur*XIP1;2-related<br>*Oleur*XIP1;2-related<br>*Oleur*XIP1;3-related	Oleur061Scf0147g01027.1 *Incomplete sequence*<br>Oleur061Scf5691g03006.1 *Incomplete sequence*<br>Oleur061Scf0694g03006.1 *Pseudogene*<br>Oleur061Scf0694g04008.1 *Pseudogene*<br>Oleur061Scf7513g00005.1 *Pseudogene*<br>Oleur061Scf9071g00003.1 *Pseudogene*<br>Oleur061Scf0258g01032.1 *Pseudogene*

NIP	***Oeu*060187.1** / *Oeu*NIP1;1	*Oleur*NIP1;1	Oleur061Scf1489g00038.1 ; 7790..10379 (+ strand)
	***Oeu*040934.1** / *Oeu*NIP1;2	*Oleur*NIP1;2	Oleur061Scf2586g05026.1 ; 547390..550593 (- strand)
	***Oeu*039868.1** / *Oeu*NIP1;3	*Oleur*NIP1;3	Oleur061Scf0038g03004.1 ; 312314..314472 (- strand)
	No hit Found	*Oleur*NIP1;4	Oleur061Scf7194g00006.1 ; 25229..27368 (- strand)
	***Oeu*053221.1** / *Oeu*NIP3;1	*Oleur*NIP3;1	Oleur061Scf1427g05001.1 ; 489963..491438 (+ strand)
	***Oeu*047418.1** / *Oeu*NIP4;1a	*Oleur*NIP4;1a	Oleur061Scf7805g00013.1 ; 30162..32022 (+ strand)
		*Oleur*NIP4;1b	Oleur061Scf3669g01008.1 ; 165510..167403 (+ strand)
	***Oeu*020545.1** / *Oeu*NIP5;1	*Oleur*NIP5;1a	Oleur061Scf0098g04035.1 ; 449548..451350 (- strand)
		*Oleur*NIP5;1b	Oleur061Scf3450g00011.1 ; 131943..133068 (- strand)
	***Oeu*046374.1** / *Oeu*NIP5;2	*Oleur*NIP5;2	Oleur061Scf1510g04020.1 ; 484673..488548 (- strand)
	**Oeu017634.1** / OeuNIP5-related *Pseudogene*	*Oleur*NIP5;3	Oleur061Scf4242g00005.1 ; 66824..76868 (- strand)
	***Oeu*060126.1** / *Oeu*NIP6;1	*Oleur*NIP6;1	Oleur061Scf9039g00011.1 ; 119150..124645 (+ strand)
	***Oeu*011481.1** / *Oeu*NIP6-related *Incomplete sequence*		
	***Oeu*037688.1** / *Oeu*NIP7;1	*Oleur*NIP7;1a	Oleur061Scf4622g04016.1 ; 475742..477284 (+ strand)
		*Oleur*NIP7;1b	Oleur061Scf1371g00022.1 ; 75020..76568 (- strand)
	***Oeu*NIP4;2-related** *Pseudogene*	*Oleur*NIP4;2-related	Oleur061Scf0296g01014.1 *Pseudogene*
	***Oeu*005264.1** / *Oeu*NIP7-related *Incomplete sequence*	No hits found	-
	-	*Oleur*NIP1-related *Pseudogene*	Oleur061Scf1489g00002.1 *Incomplete sequence*
		*Oleur*NIP1;1 related Pseudogene	Oleur061Scf0623g01004.1 *Pseudogene*
		*Oleur*NIP1;1 related Pseudogene	Oleur061Scf2586g05027.1 *Pseudogene*
		*Oleur*NIP1;1 related Pseudogene	Oleur061Scf4656g00032.1 *Pseudogene*
		*Oleur*NIP-related	Oleur061Scf9166g08006.1 *Incomplete sequence*
		*Oleur*NIP-related	Oleur061Scf2277g01043.1 *Incomplete sequence*
		*Oleur*NIP-related	Oleur061Scf2277g01009.1 *Incomplete sequence*
		*Oleur*NIP-related	Oleur061Scf2524g03003.1 *Incomplete sequence*
		*Oleur*NIP-related	Oleur061Scf4639g00001.1 *Incomplete sequence*
SIP	***Oeu*050292.1** / *Oeu*SIP1;1	*Oleur*SIP1;1a	Oleur061Scf4272g01002.1 ; 214508..224653 (+ strand)
		*Oleur*SIP1;1b	Oleur061Scf2367g00017.1 ; 30689..40838 (- strand)
	***Oeu*061896.1** / *Oeu*SIP1-related *Pseudogene*	*Oleur*SIP1;3	Oleur061Scf0342g12031.1 ; 1258679..1261622 (- strand)
	***Oeu*014723.2** / *Oeu*SIP1;2	*Oleur*SIP1;2	Oleur061Scf0259g06030.1 ; 705305..709156 (+ strand)
	***Oeu*060749.1** / *Oeu*SIP2;1	*Oleur*SIP2;1a	Oleur061Scf0186g06043.1 ; 651945..657348 (- strand)
		*Oleur*SIP2;1b	Oleur061Scf5878g07063.1 ; Pseudogene
	***Oeu*Chr11** / *Oeu*SIP2;2	OleurSIP2;2	Oleur061Scf8348g02016.1 ; 245169..250426 (- strand)
	-	*Oleur*SIP1;1-related	Oleur061Scf1072g00019.1 *Incomplete sequence*
	***Oeu*050293.1** / *Oeu*SIP-related	No hits found	-
