# Supplementary material for: Genome Wild Analysis and Molecular Understanding of the Aquaporin Diversity in Olive Trees (Olea Europaea L.)
Source: Int J Mol Sci. 2020 Jun 11;21(11):4183. doi: 10.3390/ijms21114183 (PMC7312470; doi:10.3390/ijms21114183)
Supplement: Supplementary file 1 [file ijms-21-04183-s001.zip › ijms-821530_Supplementary_files/Table S3_Faize_matrice_similarity.pdf]

Table S3. Percent Identity Matrix between the 52 full length *Oeu*AQP sequences (edited with Clustal2.1)

	1	2	3	4	5	6	7	8	9	10	11	12	13	14	15	16	17	18	19	20	21	22	23	24	25	26	27	28	29	30	31	32	33	34	35	36	37	38	39	40	41	42	43	44	45	46	47	48	49	50	51	52
1: OeuSIP2;1_Oeu060749.1	100.00	88.80	27.35	26.61																																																
2: OeuSIP2;2_OeuChr11	88.80	100.00	26.07	27.47																																																
3: OeuSIP1;1_Oeu050292.1	27.35	26.07	100.00	80.42																																																
4: OeuSIP1;2_Oeu014723.2	26.61	27.47	80.42	100.00																																																
5: OeuXIP1;1_Oeu010056.1	19.47	20.00	21.33	20.89	100.00	75.23	77.33																																													
6: OeuXIP1;2_Oeu020684.1	18.06	19.03	21.68	20.35	75.23	100.00	83.28																																													
7: OeuXIP1;3_Oeu046122.1	18.14	18.22	20.80	20.80	77.33	83.28	100.00																																													
8: OeuNIP7;1_Oeu037688.1	18.26	19.44	22.58	24.54	23.48	24.10	25.30	100.00	30.52	33.58	36.09	36.09	39.02	35.98	40.40	37.30	22.35																																			
9: OeuNIP3;1_Oeu053221.1a	17.14	16.67	21.53	24.04	21.95	23.65	21.67	30.52	100.00	36.29	33.07	33.07	37.50	41.50	41.98	39.67	28.87																																			
10: OeuNIP6;1_Oeu060126.1	18.18	17.73	20.09	18.81	22.38	21.77	24.44	33.58	36.29	100.00	60.27	60.27	41.63	41.39	44.02	42.08	23.88																																			
11: OeuNIP5;1_Oeu020545.1	18.52	17.13	18.14	15.89	20.14	24.16	24.25	36.09	33.07	60.27	100.00	91.95	40.48	42.32	43.70	41.50	22.52																																			
12: OeuNIP5;2_Oeu046374.1	19.44	18.06	18.14	16.36	20.49	23.42	23.13	36.09	33.07	60.27	91.95	100.00	40.87	43.45	44.09	43.08	22.14																																			
13: OeuNIP4;1_Oeu047418.1	17.57	15.77	18.64	18.72	23.53	23.92	24.02	39.02	37.50	41.63	40.48	40.87	100.00	53.56	56.64	56.57	25.00																																			
14: OeuNIP1;2_Oeu040934.1	18.14	17.12	19.09	19.18	18.61	19.25	19.32	35.98	41.50	41.39	42.32	43.45	53.56	100.00	63.20	63.26	25.77																																			
15: OeuNIP1;1_Oeu060187.1	14.86	14.86	20.00	19.63	24.50	25.00	25.93	40.40	41.98	44.02	43.70	44.09	56.64	63.20	100.00	78.54	25.51																																			
16: OeuNIP1;3_Oeu039868.1	15.38	14.48	19.18	18.81	23.69	24.28	24.79	37.30	39.67	42.08	41.50	43.08	56.57	63.26	78.54	100.00	25.82																																			
17: OeuPIP1;3_Oeu042329.1	16.59	17.49	24.44	25.33	27.11	26.07	27.24	22.35	28.87	23.88	22.52	22.14	25.00	25.77	25.51	25.82	100.00																																			
18: OeuPIP1;5_Oeu061384.2	17.33	17.78	24.67	24.67	25.27	25.68	25.68	22.56	26.97	24.81	23.19	22.81	24.39	25.95	26.51	26.83	90.59	100.00	88.77	88.07	85.56	85.56	85.56	85.96																												
19: OeuPIP1;4_Oeu052954.1	17.49	17.94	24.44	24.44	28.41	27.73	28.52	22.14	26.58	24.44	23.08	23.08	26.34	27.03	26.42	25.62	86.32	88.77	100.00	97.19	89.75	89.75	89.75	90.14																												
20: OeuPIP1;6_Oeu046350.1	17.94	17.94	24.00	23.56	27.68	27.34	27.73	21.37	26.16	24.44	23.08	23.08	25.93	26.25	26.42	25.62	85.26	88.07	97.19	100.00	89.75	89.75	89.75	90.14																												
21: OeuPIP1;1a_Oeu038800.1	18.10	19.46	21.97	22.87	26.37	25.78	26.95	20.99	26.58	24.06	23.28	22.52	25.51	26.25	26.02	25.21	85.21	85.56	89.75	89.75	100.00	100.00	100.00	97.19																												
22: OeuPIP1;1b_Oeu038801.1	18.10	19.46	21.97	22.87	26.37	25.78	26.95	20.99	26.58	24.06	23.28	22.52	25.51	26.25	26.02	25.21	85.21	85.56	89.75	89.75	100.00	100.00	100.00	97.19																												
23: OeuPIP1;1c_Oeu038802.2	18.10	19.46	21.97	22.87	26.37	25.78	26.95	20.99	26.58	24.06	23.28	22.52	25.51	26.25	26.02	25.21	85.21	85.56	89.75	89.75	100.00	100.00	100.00	97.19																												
24: OeuPIP1;2_Oeu018133.1	17.57	19.37	22.77	22.77	26.37	25.29	26.46	20.91	26.47	24.34	23.28	22.52	25.41	26.15	25.51	25.10	85.26	85.96	90.14	90.14	97.19	97.19	97.19	100.00																												
25: OeuPIP2;8_Oeu023232.1	18.10	18.86	22.71	23.25	28.14	27.41	29.73	19.77	26.69	25.97	24.21	23.81	23.69	25.19	25.71	25.51	69.85	68.25	71.11	70.00	71.48	71.48	71.48	71.59	100.00	95.05	81.14	82.50	74.25	79.14	79.21	77.26	79.00	78.29	77.22																	
26: OeuPIP2;9_Oeu020106.1/scaffold870	18.97	19.30	21.83	22.81	28.14	27.03	29.73	20.16	26.27	25.58	24.60	25.00	23.69	25.95	25.71	25.93	70.22	68.25	70.00	68.89	71.48	71.48	71.48	70.85	95.05	100.00	81.85	83.21	73.51	78.06	78.85	77.98	79.00	77.94	76.87																	
27: OeuPIP2;1_Oeu001271.1	19.05	19.65	21.65	20.87	27.10	27.52	28.29	19.69	24.79	23.46	24.02	23.62	24.90	25.95	24.70	25.00	69.71	68.48	71.32	70.59	72.79	72.79	72.79	71.43	81.14	81.85	100.00	95.10	77.41	83.87	83.57	84.34	83.86	83.86	83.51																	
28: OeuPIP2;2_Oeu030183.1	18.18	18.78	21.21	21.74	27.86	27.13	29.46	20.16	24.47	23.55	22.92	22.53	25.70	26.44	24.39	25.10	70.70	68.73	73.06	71.96	74.54	74.54	74.54	73.16	82.50	83.21	95.10	100.00	78.81	84.89	84.59	85.71	85.56	85.56	85.21																	
29: OeuPIP2;10_Oeu022438.1/scaffold2530	17.90	18.22	23.35	23.89	28.52	28.35	29.13	22.18	25.97	25.00	23.98	23.98	27.64	25.88	24.90	25.21	68.73	66.67	71.32	69.77	69.62	69.62	69.62	70.00	74.25	73.51	77.41	78.81	100.00	80.15	80.67	79.55	80.51	83.21	85.04																	
30: OeuPIP2;5_Oeu027987.1	18.10	18.34	21.74	23.14	27.97	26.36	28.29	21.88	26.38	24.22	24.00	23.60	26.91	27.31	27.05	26.34	71.48	69.12	73.51	72.76	73.13	73.13	73.13	73.23	79.14	78.06	83.87	84.89	80.15	100.00	91.07	85.14	86.43	86.79	86.43																	
31: OeuPIP2;7_Oeu018111.1	17.60	17.90	22.17	22.27	28.14	27.69	28.08	20.23	25.00	22.96	23.51	23.51	25.70	25.48	25.71	25.10	70.11	68.50	71.75	71.00	71.75	71.75	71.75	71.85	79.21	78.85	83.57	84.59	80.67	91.07	100.00	84.59	85.51	87.23	85.46																	
32: OeuPIP2;11_Oeu017254.1/Oeu017256.1	18.70	19.47	19.30	20.70	27.41	25.78	27.73	20.47	24.46	24.02	23.79	24.19	25.61	27.59	25.51	25.10	68.66	65.93	70.79	69.66	71.54	71.54	71.54	70.90	77.26	77.98	84.34	85.71	79.55	85.14	84.59	100.00	89.79	87.99	87.99																	
33: OeuPIP2;6_Oeu017257.1	18.45	19.21	21.21	22.17	28.52	26.92	29.23	20.93	24.05	24.42	23.81	23.81	25.30	26.14	26.42	25.93	69.12	67.15	71.85	70.74	72.22	72.22	72.22	71.59	79.00	79.00	83.86	85.56	80.51	86.43	85.51	89.79	100.00	90.94	89.90																	
34: OeuPIP2;3_Oeu033842.1	17.60	18.34	21.65	22.61	27.48	26.64	27.80	20.93	24.89	24.03	22.62	22.62	26.10	26.24	25.61	25.93	70.22	66.79	71.11	70.00	71.11	71.11	71.11	71.22	78.29	77.94	83.86	85.56	83.21	86.79	87.23	87.99	90.94	100.00	93.77																	
35: OeuPIP2;4_Oeu030439.1	17.60	18.34	21.21	21.74	27.86	26.25	27.80	21.32	24.89	24.81	23.41	23.41	26.91	26.62	25.61	25.93	69.49	66.79	71.11	70.00	70.74	70.74	70.74	70.85	77.22	76.87	83.51	85.21	85.04	86.43	85.46	87.99	89.90	93.77	100.00																	
36: OeuTIP5;1_Oeu021708.1	20.63	21.00	25.45	25.11	24.05	24.47	26.16	27.43	34.86	28.76	27.95	28.82	26.89	26.03	26.50	26.96	33.47	31.54	30.96	30.54	32.35	32.35	32.35	31.93	31.05	31.05	29.15	30.77	31.56	31.05	30.52	28.86	29.32	30.52	30.12	100.00	100.00	78.57	43.03	46.61	50.81	50.00	49.80	49.80	50.20	47.56	48.43	50.81	46.56	45.34	46.15	46.15
37: OeuTIP5;2_Oeu021709.1	20.63	21.00	25.45	25.11	24.05	24.47	26.16	27.43	34.86	28.76	27.95	28.82	26.89	26.03	26.50	26.96	33.47	31.54	30.96	30.54	32.35	32.35	32.35	31.93	31.05	31.05	29.15	30.77	31.56	31.05	30.52	28.86	29.32	30.52	30.12	100.00	100.00	78.57	43.03	46.61	50.81	50.00	49.80	49.80	50.20	47.56	48.43	50.81	46.56	45.34	46.15	46.15
38: OeuTIP5;3_Oeu022479.1	18.47	19.27	25.11	25.23	23.31	22.88	24.15	24.58	34.56	27.59	25.88	26.32	24.89	24.90	25.75	25.76	32.77	32.08	31.09	31.09	32.07	32.07	32.07	32.07	30.77	30.36	29.27	30.49	31.28	29.96	30.24	28.57	29.03	30.65	31.05	78.57	78.57	100.00	37.45	44.00	48.78	47.15	47.95	47.95	48.77	44.08	44.39	46.94	43.09	41.46	43.90	43.09
39: OeuTIP4;1_Oeu030229.1/Oeu036364.1	18.52	18.40	25.94	26.42	28.63	26.50	26.92	28.26	35.85	29.07	26.55	27.43	31.90	29.24	31.28	30.04	29.91	30.77	30.34	30.34	32.48	32.48	32.48	32.05	30.83	31.25	30.96	31.38	31.51	32.08	29.46	29.41	30.29	31.12	31.12	43.03	43.03	37.45	100.00	48.37	54.07	54.07	52.65	52.65	55.51	51.63	50.00	54.47	46.96	52.23	51.01	51.42
40: OeuTIP3;1_Oeu038387.1	17.12	18.26	23.18	22.83	26.45	26.86	26.86	27.31	30.80	28.45	26.38	26.38	29.92	26.32	28.99	29.91	33.75	33.47	32.92	33.75	34.73	34.73	34.73	34.73	34.68	34.68	33.47	33.87	33.61	33.87	33.33	33.33	34.14	34.54	34.54	46.61	46.61	44.00	48.37	100.00	50.81	50.40	51.61	51.61	53.63	55.42	57.52	59.84	57.20	54.40	57.60	58.00
41: OeuTIP2;4_Oeu002329.1	18.89	19.25	21.96	22.90	22.78	25.74	26.58	27.16	36.45	31.44	32.02	31.14	29.91	30.13	28.38	28.44	34.32	34.32	34.32	34.32	36.86	36.86	36.86	36.44	36.78	36.78	35.68	36.51	36.25	35.54	34.84	33.61	34.84	35.80	34.57	50.81	50.81	48.78	54.07	50.81	100.00	93.60	78.95	78.95	80.57	59.27	58.67	61.69	55.82	56.63	57.03	57.03
42: OeuTIP2;5_Oeu040207.1	19.82	20.19	21.96	23.36	22.78	24.89	26.58	26.72	35.51	31.00	31.58	31.14	30.77	30.54	27.95	28.44	34.75	33.90	33.47	33.47	36.44	36.44	36.44	35.59	35.54	36.36	35.27	36.10	35.83	34.71	34.02	33.61	34.84	35.39	34.16	50.00	50.00	47.15	54.07	50.40	93.60	100.00	80.57	80.57	81.78	58.87	58.67	62.50	56.22	57.03	57.43	57.43
43: OeuTIP2;1_Oeu024074.1	20.83	20.56	22.79	23.72	23.73	24.58	27.12	28.88	34.42	32.17	32.75	32.31	30.64	29.54	30.87	30.97	34.18	34.18	33.33	33.33	35.86	35.86	35.86	35.02	35.27	36.10	35.12	35.54	36.40	35.54	35.54	33.47	34.71	34.71	33.47	49.80	49.80	47.95	52.65	51.61	78.95	80.57	100.00	100.00	89.52	62.10	59.56	65.73	56.85	59.27	59.68	59.68
44: OeuTIP2;2_Oeu024076.1	20.83	20.56	22.79	23.72	23.73	24.58	27.12	28.88	34.42	32.17	32.75	32.31	30.64	29.54	30.87	30.97	34.18	34.18	33.33	33.33	35.86	35.86	35.86	35.02	35.27	36.10	35.12	35.54	36.40	35.54	35.54	33.47	34.71	34.71	33.47	49.80	49.80	47.95	52.65	51.61	78.95	80.57	100.00	100.00	89.52	62.10	59.56	65.73	56.85	59.27	59.68	59.68
45: OeuTIP2;3_Oeu053988.1	20.37	20.09	22.33	22.79	22.46	24.58	27.12	28.88	35.35	31.74	32.31	31.44	30.64	29.11	29.13	28.76	33.76	33.76	32.91	33.33	35.86	35.86	35.86	35.02	35.27	36.51	35.95	36.36	36.82	35.95	35.12	34.31	35.54	35.95	34.71	50.20	50.20	48.77	55.51	53.63	80.57	81.78	89.52	89.52	100.00	62.90	60.89	65.73	56.85	58.06	58.47	58.47
46: OeuTIP1;3_Oeu035479.1	20.28	20.09	27.44	26.51	23.67	25.41	24.18	27.27	29.91	27.62	26.05	26.47	30.33	26.72	28.76	28.38	32.65	33.88	33.61	33.61	33.33	33.33	33.33	33.74	34.26	33.86	34.66	33.86	33.73	33.73	34.52	32.13	33.73	34.13	32.94	47.56	47.56	44.08	51.63	55.42	59.27	58.87	62.10	62.10	62.90	100.00	72.25	73.93	71.31	73.31	72.11	72.51
47: OeuTIP1;6_Oeu033244.1	17.26	16.75	22.11	21.11	21.17	22.52	22.52	25.00	30.96	26.98	26.17	26.17	27.78	27.78	26.32	27.32	31.84	31.39	30.94	30.49	32.29	32.29	32.29	32.29	35.27	34.82	33.78	33.33	33.33	34.67	35.11	31.53	32.00	33.78	33.33	48.43	48.43	44.39	50.00	57.52	58.67	58.67	59.56	59.56	60.89	72.25	100.00	75.77	76.65	78.85	79.74	80.18
48: OeuTIP1;7_Oeu045237.1	17.05	17.29	27.91	26.98	26.23	26.23	27.46	26.97	30.94	27.73	26.58	27.43	29.22	26.02	25.86	26.75	32.79	31.97	32.38	32.38	33.06	33.06	33.06	33.06	35.20	35.20	33.60	34.40	34.27	34.26	34.26	33.06	34.26	34.66	33.86	50.81	50.81	46.94	54.47	59.84	61.69	62.50	65.73	65.73	65.73	73.93	75.77	100.00	75.70	77.69	80.48	80.48
49: OeuTIP1;2_Oeu041014.1	17.89	18.69	26.51	26.05	24.69	25.52	25.52	27.23	29.49	28.45	28.57	29.00	28.27	27.80	29.57	30.09	35.98	35.98	35.56	35.15	36.40	36.40	36.40	36.40	36.33	36.73	37.30	36.48	36.63	35.92	35.77	34.57	35.77	36.99	36.18	46.56	46.56	43.09	46.96	57.20	55.82	56.22	56.85	56.85	56.85	71.31	76.65	75.70	100.00	82.14	82.54	83.33
50: OeuTIP1;1_Oeu058531.1	18.35	18.69	27.91	27.91	25.94	26.36	25.52	26.38	31.34	28.45	27.71	28.14	27.00	26.97	27.83	27.43	32.64	33.05	33.05	33.47	33.89	33.89	33.89	33.89	35.51	35.10	34.43	34.43	33.74	35.10	34.55	32.51	33.74	34.55	33.33	45.34	45.34	41.46	52.23	54.40	56.63	57.03	59.27	59.27	58.06	73.31	78.85	77.69	82.14	100.00	88.49	89.29
51: OeuTIP1;4_Oeu040290.1	16.97	16.82	25.58	25.58	25.10	25.52	25.10	27.23	32.72	28.02	27.71	28.14	27.43	27.80	27.83	28.76	33.89	33.89	33.89	34.31	34.73	34.73	34.73	34.73	36.73	36.33	36.07	35.25	34.98	34.69	35.37	33.33	34.55	35.37	34.15	46.15	46.15	43.90	51.01	57.60	57.03	57.43	59.68	59.68	58.47	72.11	79.74	80.48	82.54	88.49	100.00	99.21
52: OeuTIP1;5_Oeu040289.1	16.97	16.82	25.58	25.58	25.10	25.52	25.10	27.23	32.26	28.02	27.71	28.14	27.43	27.80	27.83	28.76	33.89	33.89	33.89	34.31	34.73	34.73	34.73	34.73	36.73	36.33	36.07	35.25	34.98	34.69	35.37	33.33	34.55	35.37	34.15	46.15	46.15	43.09	51.42	58.00	57.03	57.43	59.68	59.68	58.47	72.51	80.18	80.48	83.33	89.29	99.21	100.00
