# Supplementary material for: Genome Wild Analysis and Molecular Understanding of the Aquaporin Diversity in Olive Trees (Olea Europaea L.)
Source: Int J Mol Sci. 2020 Jun 11;21(11):4183. doi: 10.3390/ijms21114183 (PMC7312470; doi:10.3390/ijms21114183)
Supplement: Supplementary file 1 [file ijms-21-04183-s001.zip › ijms-821530_Supplementary_files/Table S4_Faize_OleurAQP_RNAseq.pdf]

Table S4. Expression profile of *Oleur*AQP genes from the cultivated *Olea europaea* cv. Picual in different physiological conditions.
*Oleur*AQsP are grouped by subfamily.
*Oleu*AQP gene expressions were normalized in terms of reads per kilobase of transcript per million mapped reads (RPKM).
Expression levels were displayed by the depth of red.

			Organs/tissues						Early development (months)						Root								Leaf		
															Control	Wound (h, hours; d, days)				Verticillium dahliae			Control	Cold (days)	
AQP subclasses	*Oleur*AQP name	*Oeu*AQPs correspondance	Fruit	Flower	Leaf	Meritem	Root	Stem	1	2	3	4	5	6	0	8h	24h	48h	7d	48h	7d	15d	0	1d	10d
PIP1	*Oleur*PIP1;1	*Oeu*PIP1;1abc	576	560	328	275	88	278	295	93	616	534	512	427	2472	794	1209	1767	2761	961	420	2427	375	477	302
	*Oleur*PIP1;2a	*Oeu*PIP1;2	84	46	42	68	16	23	25	26	82	83	63	19	294	19	13	39	542	9	112	302	91	72	48
	*Oleur*PIP1;2b		129	34	25	42	20	21	50	24	60	76	41	16	665	46	23	127	1167	24	152	729	92	75	31
	*Oleur*PIP1;3a	*Oeu*PIP1;3	115	83	18	11	283	104	153	242	210	55	104	185	409	290	714	766	555	501	241	184	41	38	15
	*Oleur*PIP1;3b		93	137	22	24	266	116	48	69	58	11	37	60	454	303	652	738	505	434	213	199	44	36	19
	*Oleur*PIP1;4a	*Oeu*PIP1;4	0	44	4	16	11	5	87	32	63	75	60	15	89	21	29	9	48	3	9	53	52	22	10
	*Oleur*PIP1;4b		9	79	6	22	9	8	70	36	52	50	47	19	107	27	33	10	60	5	15	51	62	24	13
	*Oleur*PIP1;5a	*Oeu*PIP1;5	0	3	4	9	13	7	10	23	52	22	32	36	26	7	5	3	6	1	3	19	42	28	24
	*Oleur*PIP1;5b		0	45	2	8	6	5	22	17	12	3	5	3	161	33	23	34	36	24	34	141	7	12	3
	*Oleur*PIP1;6	*Oeu*PIP1;6	1	16	2	7	3	2	7	5	3	2	4	1	31	14	14	18	25	6	7	12	8	11	2
	*Oleur*PIP1;6b		1	13	0	4	3	1	31	13	14	6	7	5	37	10	13	15	26	4	4	12	7	8	2
	Pseudogen	-	0	0	0	1	1	0	0	0	0	0	0	0	0	0	0	0	0	0	0	0	0	0	0
	Incomplete sequence	-	0	0	0	0	0	0	0	0	0	0	0	0	0	0	0	0	0	1	0	0	0	0	0
	Incomplete sequence	-	0	0	0	0	0	0	0	0	0	0	0	0	0	0	0	0	0	0	0	0	0	0	0
	Incomplete sequence	-	0	0	0	0	0	0	0	0	0	0	0	0	0	0	0	0	0	0	0	0	0	0	0
	Incomplete sequence	-	0	0	0	0	0	0	1	0	0	0	0	0	0	0	0	0	0	0	0	0	0	0	0
	Incomplete sequence	-	0	0	0	0	0	0	0	0	0	0	0	0	0	0	0	0	0	0	0	0	0	0	0
	Incomplete sequence	-	0	0	0	0	0	0	0	0	0	0	0	0	0	0	0	0	0	0	0	0	0	0	0
	Incomplete sequence	-	0	0	0	0	0	0	1	1	0	0	0	0	0	0	0	1	0	0	0	0	0	0	0
	Incomplete sequence	-	0	0	0	0	0	0	0	0	0	0	0	0	0	0	0	0	0	0	0	0	0	0	0
	Incomplete sequence	-	0	0	0	0	0	0	0	0	0	0	0	0	0	0	0	0	0	0	0	0	0	0	0
	Incomplete sequence	-	0	0	0	0	0	0	0	0	0	0	0	0	0	0	0	0	0	0	0	0	0	0	0
	Incomplete sequence	-	0	0	0	0	0	0	0	0	0	0	0	0	0	0	0	0	0	0	0	0	0	0	0
	Incomplete sequence	-	0	0	0	0	0	0	0	0	0	0	0	0	0	0	0	0	0	0	0	0	0	0	0
PIP2	*Oleur*PIP2;1a	*Oeu*PIP2;1	1	0	1	0	33	5	7	2	7	6	11	9	639	166	181	276	450	139	106	656	10	1	0
	*Oleur*PIP2;1b		10	3	2	0	57	8	57	54	105	67	64	62	846	300	325	471	791	267	202	1072	48	6	0
	*Oleur*PIP2;2a	*Oeu*PIP2;2	0	0	0	0	0	0	4	1	5	0	0	0	402	10	12	38	474	11	99	374	1	0	0
	*Oleur*PIP2;2b		0	0	0	0	0	0	0	0	0	0	0	0	382	23	32	78	500	27	100	301	0	0	0
	*Oleur*PIP2.3	*Oeu*PIP2;3	0	0	0	0	1	0	1	1	1	0	0	0	24	3	2	5	33	2	10	32	0	1	0
	*Oleur*PIP2;3b		0	1	0	0	0	0	0	0	0	0	0	0	27	2	1	2	21	1	6	34	0	0	0
	*Oleur*PIP2;4	*Oeu*PIP2;4	0	2	0	0	1	0	7	3	8	3	4	1	22	8	3	2	32	1	8	113	3	2	1
	*Oleur*PIP2;5	*Oeu*PIP2;5	20	18	8	9	19	20	54	112	117	157	100	59	522	366	164	346	1047	225	242	559	142	149	25
	*Oleur*PIP2;6a	*Oeu*PIP2;6	83	44	112	93	14	127	9	5	17	16	11	13	52	31	19	7	26	1	28	157	65	118	257
	*Oleur*PIP2.6b		15	21	48	50	1	49	41	15	61	57	46	44	3	0	0	0	2	0	1	6	33	72	126
	*Oleur*PIP2;7	*Oeu*PIP2;7	75	21	2	16	1	4	1	0	15	48	22	4	110	6	5	13	80	5	26	48	34	54	27
	*Oleur*PIP2.7-related *Incomplete sequence*		17	3	0	6	0	0	0	0	2	13	6	0	24	1	0	1	12	1	3	7	9	12	3
	*Oleur*PIP2;8a	*Oeu*PIP2;8	4	130	2	13	98	39	118	122	161	30	86	56	277	232	468	416	365	318	154	168	119	60	11
	*Oleur*PIP2;8b		67	150	10	18	106	68	139	124	140	52	54	104	241	238	543	397	321	366	178	122	136	65	17
	*Oleur*PIP2;9a	*Oeu*PIP2;9	62	31	1	1	1	0	168	77	71	52	73	27	108	38	81	45	75	46	49	88	44	21	6
	*Oleur*PIP2;9b		15	26	1	3	0	0	50	26	52	12	13	17	98	31	55	29	66	22	28	65	44	22	6
	*Oleur*PIP2;10	*Oeu*PIP2;10	0	1	0	0	0	2	3	1	3	1	1	2	2	2	8	2	1	4	1	6	1	1	1
	*Oleur*PIP2.10-related *Incomplete sequence*		0	0	0	0	0	1	1	0	2	0	0	1	4	2	5	2	1	2	1	13	1	1	0
	*Oleur*PIP2.11 *Incomplete sequence*	*Oeu*PIP2;11	0	0	0	0	0	0	0	0	0	0	0	0	24	17	12	4	12	1	10	62	0	0	0
	*Oleur*PIP2.11-related *Incomplete sequence*		0	0	0	0	0	0	0	0	0	0	0	0	0	0	0	0	0	0	0	1	0	0	0
	*Oleur*PIP2;12	-	433	216	32	99	84	65	319	251	253	251	222	231	773	266	511	767	543	501	213	723	131	56	67
	*Oleur*PIP2-related *Incomplete sequence*	-	0	0	0	0	0	0	0	0	0	1	0	0	0	0	0	0	0	0	0	0	0	0	0
TIP	*Oleur*TIP1;1	*Oeu*TIP1;1	0	6	0	7	0	0	4	1	10	75	22	2	66	5	2	3	86	1	29	130	31	17	5
	*Oleur*TIP1;1-related *Incomplete sequence*		0	0	0	0	0	0	0	0	0	0	0	0	0	0	0	0	0	0	0	0	0	0	0
	*Oleur*TIP1;2a	*Oeu*TIP1;2	0	0	0	0	0	0	14	2	8	0	0	0	490	165	78	198	820	104	136	989	0	0	0
	*Oleur*TIP1;2b		0	0	0	0	1	0	2	0	2	0	0	0	505	213	93	272	883	144	147	873	0	0	0
	*Oleur*TIP1;3a	*Oeu*TIP1;3	0	522	68	162	370	601	1318	517	222	302	245	137	645	234	162	218	533	152	114	751	155	121	266
	*Oleur*TIP1;3b		0	0	0	6	0	0	0	0	0	0	0	0	2	16	11	1	31	1	9	51	8	1	15
	*Oleur*TIP1;3c		0	1	0	0	1	0	1	0	0	0	0	0	2	1	0	1	1	0	0	0	1	0	0
	*Oleur*TIP1;3d		1084	10	57	2	4	18	118	84	81	68	61	74	30	157	211	228	158	174	63	35	69	79	145
	*Oleur*TIP1;3e		94	7	38	1	13	9	23	33	11	12	14	34	92	153	190	240	243	158	68	34	15	52	69
	*Oleur*TIP1;4-related *Incomplete sequence*	*Oeu*TIP1;4	1	4	0	0	0	0	18	3	8	9	7	4	4	0	3	0	2	0	0	2	27	3	0
	*Oleur*TIP1;5	*Oeu*TIP1;5	1	4	0	0	0	0	17	3	7	9	7	4	4	0	3	0	2	0	0	2	27	3	0
	*Oleur*TIP1;6	*Oeu*TIP1;6	37	50	31	14	15	19	15	10	13	9	8	10	16	15	16	10	16	15	22	25	10	13	25
	*Oleur*TIP1;7	*Oeu*TIP1;7	0	0	0	0	0	0	2	1	1	0	0	0	8	18	41	48	49	73	38	57	1	0	0
	*Oleur*TIP1;7b-related *Pseudogen*		0	0	0	0	0	0	0	0	0	0	0	0	1	0	0	0	0	0	0	3	0	0	0
	*Oleur*TIP2;1	*Oeu*TIP2;1 - *Oeu*TIP2;2	106	35	3	2	1	1	141	37	96	48	45	11	308	113	47	54	194	34	60	546	83	16	1
	*Oleur*TIP2;3a	*Oeu*TIP2;3	53	90	3	0	0	0	3	1	8	21	17	5	82	5	0	3	88	1	39	594	60	21	22
	*Oleur*TIP2;3b		122	141	11	0	0	3	24	5	70	248	150	22	121	7	1	8	249	1	78	559	112	45	35
	*Oleur*TIP2;4	*Oeu*TIP2;4	0	0	0	0	0	0	0	0	2	0	0	0	198	10	3	32	435	4	54	483	0	0	0
	*Oleur*TIP2;4-related *Pseudogene*		0	0	0	0	0	0	0	0	1	0	0	0	102	3	1	10	153	2	22	107	0	0	0
	*Oleur*TIP2;5a	*Oeu*TIP2;5	0	0	0	0	0	0	0	0	0	0	0	0	432	5	5	37	381	10	32	130	0	0	0
	*Oleur*TIP2;5b		0	0	0	0	0	0	0	0	1	0	0	0	303	6	4	30	271	7	24	119	0	0	0
	*Oleur*TIP3;1a	*Oeu*TIP3;1	0	0	0	0	0	0	0	0	0	0	0	0	0	0	0	0	0	0	0	0	0	0	0
	*Oleur*TIP3;1b		0	0	0	0	0	0	0	0	0	0	0	0	0	0	0	0	0	0	0	0	0	0	0
	*Oleur*TIP3;1c		0	0	0	0	0	0	0	0	0	0	0	0	0	0	0	0	0	0	0	0	0	0	0
	*Oleur*TIP3;1-related Incomplete sequece		0	0	0	0	0	0	0	0	0	0	0	0	0	0	0	0	0	0	0	0	0	0	0
	*Oleur*TIP4;1	*Oeu*TIP4;1	66	5	0	0	1	0	6	0	2	0	1	1	54	7	1	3	19	0	8	54	0	0	0
	*Oleur*TIP5;1	*Oeu*TIP5;1	0	0	0	0	0	0	0	0	0	0	0	0	0	0	0	0	0	0	0	0	0	0	0
	*Oleur*TIP5;1-relatd *Incomplete sequence*		0	0	0	0	0	0	0	0	0	0	0	0	0	0	0	0	0	0	0	0	0	0	0
	*Oleur*TIP5;2	*Oeu*TIP5;2	0	0	0	0	0	0	0	0	0	0	0	0	1	0	0	0	0	0	0	0	0	0	0
	*Oleur*TIP5;3a	*Oeu*TIP5;3	0	0	0	0	0	0	0	0	0	0	0	0	0	0	0	0	0	0	0	0	0	0	0
	*Oleur*TIP5;3b		0	0	0	0	0	0	0	0	0	0	0	0	0	0	0	0	0	0	0	0	0	0	0
	*Oleur*TIP1-related *Pseudogene*	-	0	0	0	0	0	0	0	0	0	0	0	0	0	0	0	0	0	0	0	0	0	0	0
	*Oleur*TIP1-related *Pseudogene*	-	0	0	0	0	0	0	0	0	0	0	0	0	0	0	0	0	0	0	0	2	0	0	0
	*Oleur*TIP1-related *Pseudogene*	-	0	0	0	0	0	0	0	0	0	0	0	0	0	0	0	0	0	0	0	0	0	0	0
	*Oleur*TIP2-related *Incomplete sequence*	-	0	0	0	0	0	0	0	0	0	0	0	0	0	0	0	0	0	0	0	0	0	0	0
	*Oleur*TIP2-related *Incomplete sequence*	-	0	0	0	0	0	0	0	0	0	0	0	0	0	0	0	0	0	0	0	1	0	0	0
	*Oleur*TIP2-related *Pseudogene*	-	0	0	0	0	0	0	0	0	0	0	0	0	0	0	0	0	0	0	0	0	0	0	0
	*Oleur*TIP4-related *Incomplete sequence*	-	11	0	0	0	0	0	0	0	0	0	0	0	17	2	0	0	6	0	3	29	0	0	0
XIP	*Oleur*XIP1;1a	*Oeu*XIP1;1	1	42	0	16	0	1	117	25	38	38	28	11	0	0	12	0	0	0	0	0	68	12	1
	*Oleur*XIP1;1b		9	21	0	8	0	0	10	2	2	8	5	3	0	0	7	0	0	0	0	0	38	6	1
	*Oleur*XIP1;2a	*Oeu*XIP1;2	0	0	0	0	0	0	0	0	1	0	0	0	0	0	0	0	0	0	0	0	0	0	0
	*Oleur*XIP1;2b		0	0	0	0	0	0	0	0	0	0	0	0	0	0	0	0	0	0	0	0	0	0	0
	*Oleur*XIP1;2c		0	0	0	0	0	0	0	0	1	0	0	0	0	0	0	0	0	0	0	0	0	0	0
	*Oleur*XIP2-related		0	0	0	0	0	0	0	0	0	0	0	0	1	0	0	0	0	0	0	0	0	0	0
	*Oleur*XIP2-related		0	0	0	0	0	0	0	0	0	0	0	0	0	0	0	0	0	0	0	0	0	0	0
	*Oleur*XIP2-related		0	0	0	0	0	0	0	0	0	0	0	0	0	0	0	0	0	0	0	0	0	0	0
	*Oleur*XIP2-related		0	0	0	0	0	0	0	0	0	0	0	0	0	0	0	0	0	0	0	0	0	0	0
	*Oleur*XIP2-related		0	0	0	0	0	0	0	0	0	0	0	0	0	0	0	0	0	0	0	0	0	0	0
	*Oleur*XIP1;3	*Oeu*XIP1;3	0	36	0	0	0	0	0	0	0	0	0	0	0	0	0	0	0	0	0	0	0	0	0
	*Oleur*XIP3-related		0	0	0	0	0	0	0	0	0	0	0	0	0	0	0	0	0	0	0	0	0	0	0
NIP	*Oleur*NIP1;1	*Oeu*NIP1;1	2	9	6	12	5	2	9	6	11	11	10	18	0	0	0	0	0	0	0	0	12	4	9
	*Oleur*NIP1.2	*Oeu*NIP1;2	5	2	1	2	5	1	2	1	2	0	1	2	9	9	18	17	15	25	36	13	1	2	2
	*Oleur*NIP1;3	*Oeu*NIP1;3	0	0	4	1	0	0	0	0	0	0	0	0	0	1	1	0	0	0	0	0	0	0	1
	*Oleur*NIP1;4	No hit Found	0	0	3	0	0	0	0	0	0	0	0	0	0	0	0	0	0	0	0	0	0	0	1
	*Oleur*NIP3;1	*Oeu*NIP3;1	0	0	0	0	0	0	1	0	1	0	0	0	0	0	0	0	0	0	0	0	0	0	0
	*Oleur*NIP4;1a	*Oeu*NIP4;1	0	2	0	0	0	0	0	0	0	0	0	0	0	0	0	0	0	0	0	0	0	0	0
	*Oleur*NIP4;1b		0	3	0	0	0	0	0	0	0	0	0	0	0	0	0	0	0	0	0	0	0	0	0
	*Oleur*NIP4;2-related *Pseudogene*	*Oeu*NIP4;2-related *Pseudogene*	0	0	0	0	0	0	0	0	0	0	0	0	0	0	0	0	0	0	0	0	0	0	0
	*Oleur*NIP5;1a	*Oeu*NIP5;1	6	13	0	0	35	5	6	2	8	3	4	2	35	20	5	7	49	3	17	101	1	2	1
	*Oleur*NIP5;1b		5	4	0	0	7	0	2	1	2	1	2	0	16	8	2	2	17	2	6	31	0	0	0
	*Oleur*NIP5;2	*Oeu*NIP5;2	0	5	0	2	1	2	1	0	1	1	1	0	8	8	4	4	5	2	4	16	1	1	1
	*Oleur*NIP5;3	*Oeu*NIP5-related *Pseudogene*	8	10	10	40	12	18	8	8	13	6	8	12	22	39	27	38	45	33	28	70	6	5	9
	*Oleur*NIP6;1	*Oeu*NIP6;1	0	0	0	0	0	0	0	0	0	0	0	0	0	0	0	0	0	0	0	0	0	0	0
	*Oleur*NIP7-1a	*Oeu*NIP7;1	0	378	0	0	0	0	0	0	0	0	0	0	0	0	1	1	0	1	1	0	0	0	0
	*Oleur*NIP7;1b		0	142	0	0	0	0	0	0	0	0	0	0	0	0	0	0	0	0	1	0	0	0	0
	*Oleur*NIP1-related *Incomplete sequence*	-	0	0	0	0	0	0	0	0	0	0	0	0	10	3	8	6	22	4	10	1	0	0	0
	*Oleur*NIP1;1-related *Pseudogene*	-	0	1	1	1	0	0	0	0	1	0	1	1	1	0	1	0	3	0	0	0	2	1	2
	*Oleur*NIP1-1 related Pseudogene	-	1	6	1	2	0	0	3	3	5	3	4	3	5	3	6	6	5	5	3	7	4	2	1
	*Oleur*NIP1-1 related Pseudogene	-	0	0	0	0	0	0	0	0	0	0	0	0	0	0	0	0	0	0	0	0	0	0	0
	*Oleur*NIP-related *Incomplete sequence*	-	0	0	0	0	0	0	0	0	0	0	0	0	0	0	0	0	0	0	0	0	0	0	0
	*Oleur*NIP-related *Incomplete sequence*	-	0	0	0	0	0	0	0	0	0	0	0	0	0	0	0	0	0	0	0	0	0	0	0
	*Oleur*NIP-related *Incomplete sequence*	-	0	0	0	0	0	0	0	0	0	0	0	0	0	0	0	0	0	0	0	0	0	0	0
	*Oleur*NIP-related *Incomplete sequence*	-	0	0	0	0	0	0	0	0	0	0	0	0	1	0	0	0	0	0	0	0	0	0	0
	*Oleur*NIP-related *Incomplete sequence*	-	0	0	0	0	0	0	0	0	0	0	0	0	0	0	0	0	0	0	0	0	0	0	0
SIP	*Oleur*SIP1;1a	*Oeu*SIP1;1	16	108	32	25	36	26	38	34	35	15	22	33	49	65	46	48	48	43	19	30	19	28	54
	*Oleur*SIP1;1b		16	95	32	23	30	23	0	0	0	0	0	0	40	55	53	65	61	64	34	36	15	24	34
	*Oleur*SIP1;3	*Oeu*SIP1;3-related ; *Pseudogene*	4	60	9	2	4	4	3	2	3	3	3	3	4	3	4	5	5	4	2	4	2	3	5
	*Oleur*SIP1;2	OeuSIP1;2	3	1	1	2	4	2	1	4	2	1	1	2	5	20	10	14	6	15	3	4	0	0	1
	*Oleur*SIP2;1a	*Oeu*SIP2;1	1	3	5	2	2	2	8	19	9	24	11	10	10	3	3	3	4	3	1	2	22	11	3
	*Oleur*SIP2;1b	*Oeu*SIP2;1	0	0	0	0	1	0	1	2	1	5	2	1	6	1	0	1	2	1	1	0	5	2	1
	*Oleur*SIP2;2	*Oeu*SIP2;2	0	0	0	1	0	1	3	3	2	1	2	2	2	1	1	1	1	1	1	2	2	1	0
	*Oleur*SIP-related *Incomplete sequence*	No hit found	3	25	4	3	4	5	1	3	2	1	1	3	2	3	4	3	4	3	1	3	1	2	2
